# The small non-coding RNA profile of mouse oocytes is modified during aging

**DOI:** 10.18632/aging.101947

**Published:** 2019-05-24

**Authors:** Bettina P. Mihalas, Nicole J. Camlin, Miguel J. Xavier, Alexandra E. Peters, Janet E. Holt, Jessie M. Sutherland, Eileen A. McLaughlin, Andrew L. Eamens, Brett Nixon

**Affiliations:** 1 Priority Research Centre for Reproductive Science, Schools of Environmental and Life Sciences and Biomedical Science and Pharmacy, the University of Newcastle, Callaghan, New South Wales 2308, Australia; 2 Pregnancy and Reproduction Program, Hunter Medical Research Institute, New Lambton Heights, New South Wales 2305, Australia; 3 Department of Biochemistry and Molecular Biology, Johns Hopkins Bloomberg School of Public Health, Baltimore, Maryland 21218, United States; 4 School of Biological Sciences, University of Auckland, Auckland 1142, New Zealand; 5 School of Science, University of Canberra, Bruce, Australian Capital Territory 2617, Australia; 6 School of Environmental and Life Sciences, the University of Newcastle, Callaghan, New South Wales 2308, Australia

**Keywords:** oocyte, small non-coding RNA, maternal aging, aneuploidy, miRNA, endo-siRNA, meiosis, kinesin

## Abstract

Oocytes are reliant on messenger RNA (mRNA) stores to support their survival and integrity during a protracted period of transcriptional dormancy as they await ovulation. Oocytes are, however, known to experience an age-associated alteration in mRNA transcript abundance, a phenomenon that contributes to reduced developmental potential. Here we have investigated whether the expression profile of small non-protein-coding RNAs (sRNAs) is similarly altered in aged mouse oocytes. The application of high throughput sequencing revealed substantial changes to the global sRNA profile of germinal vesicle stage oocytes from young (4-6 weeks) and aged mice (14-16 months). Among these, 160 endogenous small-interfering RNAs (endo-siRNAs) and 10 microRNAs (miRNAs) were determined to differentially accumulate within young and aged oocytes. Further, we revealed decreased expression of two members of the kinesin protein family, *Kifc1* and *Kifc5b*, in aged oocytes; family members selectively targeted for expression regulation by endo-siRNAs of elevated abundance. The implications of reduced *Kifc1* and *Kifc5b* expression were explored using complementary siRNA-mediated knockdown and pharmacological inhibition strategies, both of which led to increased rates of aneuploidy in otherwise healthy young oocytes. Collectively, our data raise the prospect that altered sRNA abundance, specifically endo-siRNA abundance, could influence the quality of the aged oocyte.

## Introduction

Declining oocyte quality is a significant contributor to decreased female fertility that accompanies maternal aging in mammals [1–3]. Although the etiology of declining oocyte quality is undoubtedly complex, this is likely linked to the compromise of homeostatic cellular processes including the regulation of normal gene expression. Indeed, mammalian oocytes are uniquely vulnerable to changes in gene expression since they are rendered transcriptionally quiescent from the mature germinal vesicle (GV) stage until embryonic genome activation at the two-cell stage (mouse) or 4-8 cell stage (human) of development [4,5]. During this protracted period of dormancy, and the subsequent meiotic resumption and early embryo development, the oocyte is dependent on transcripts that have been accumulated, stored, and maintained throughout its development [4]. Global transcriptomic analyses have revealed substantial alteration of transcript profiles in both mouse [1,6] and human oocytes [7] throughout maternal aging. Importantly, many of the transcripts with altered expression encode for meiotically essential proteins, with such changes likely contributing to the decrease in oocyte quality, including the age-related aneuploidy phenotype [1]. Additionally, many of these transcriptomic changes are reflected at the protein level in mouse oocytes [1,8]. Despite this knowledge, the underlying causes of age-associated alterations to oocyte transcript abundance remain to be fully elucidated.

Modification of transcript stability via small non-protein-coding RNA (sRNAs)-directed regulation represents a mechanism by which gene expression can be altered post-transcriptionally. Endogenous small-interfering RNAs (endo-siRNAs) and microRNAs (miRNAs) are two sRNA classes that have been recently comprehensively explored in mouse oocytes through the application of transgenic models [9–11]. In this context, the oocyte specific depletion of both the endo-siRNA and miRNA biogenesis pathways in *Zp3-Dicer* conditional knockout animals (cKO), was shown to induce meiotic failure characterized by spindle malformations and chromosome compression defects [12,13]. Unsurprisingly, the oocytes of *Zp3-Dicer* cKO animals also demonstrated significant alteration to gene expression, with increased transcript abundance documented for up to one-third of all genes expressed in the oocyte [10,12,13]. Of particular note, many of the transcripts with altered abundance in the oocytes of *Zp3-Dicer* cKO animals encode protein machinery related to microtubule dynamics including microtubule-based processes, kinesin complex, motor activity, spindle formation, and microtubule-associated complexes [9]. Further, impairment of the endo-siRNA pathway via a catalytically inactive knock-in allele of Argonaute 2 (*Ago2)* [14], or a cKO of *Ago2* in *Zp3*-*Ago2* animals [15], resulted in the expression of comparable phenotypes and alterations to the oocyte transcriptome.

Notwithstanding these data, several studies have reported that miRNA activity is repressed in mature oocytes; findings that tentatively link endo-siRNA to the meiotic failure observed in conditional *Dicer* and *Ago2* knockout mice [9,10,14,16]. Consistent with this model, the mRNA transcripts upregulated in *Zp3-Dicer* cKO oocytes did not display seed matches to either canonical or non-canonical miRNAs [16,17]. Rather, the loss of either DICER or AGO2 activity in the oocytes of cKO mutants was associated with the increased abundances of protein-coding transcripts that harbor target sequences complementary to the endo-siRNAs with reduced levels of accumulation in KO oocytes [9,10,14]. Additionally, the oocytes of mice generated from conditional ablation of *Zp3-Dgcr8* (DGCR8; DiGeorge syndrome critical region8, the processing cofactor of DROSHA) showed no changes to their mRNA abundance profile, meiotic competency or pre-implantation embryo development [17]. Cumulatively, these studies suggest that despite original conclusions, to the contrary, meiotic completion in oocytes is likely dependent on the regulatory activity of endo-siRNAs rather than miRNAs. These *Zp3-Dgcr8* cKO animals did, however, experience significant reductions in litter size, suggesting that maternal miRNAs may have an important downstream role in post-implantation embryo development [17].

Despite the implications of this work, there are few reports on the regulatory role played by sRNAs in the context of aging oocytes. Accordingly, we have utilized high throughput sequencing (RNA-Seq) to profile the sRNA landscape of oocytes from young and aged mice. Moreover, we have attempted to explore the biological significance of an altered sRNA profile in aged oocytes by focusing our research attention on the putative endo-siRNA targets, *Kifc1* and *Kifc5b*, which are members of the kinesin protein family previously demonstrated as being essential for meiosis [18]. Collectively, our data raise the intriguing prospect that an altered sRNA landscape in aged oocytes, particularly an altered endo-siRNA accumulation profile, could influence the quality of the aged oocyte.

## RESULTS

### Comparison of the endo-siRNA and miRNA landscapes of oocytes from young and aged mice

**Figure 1:**
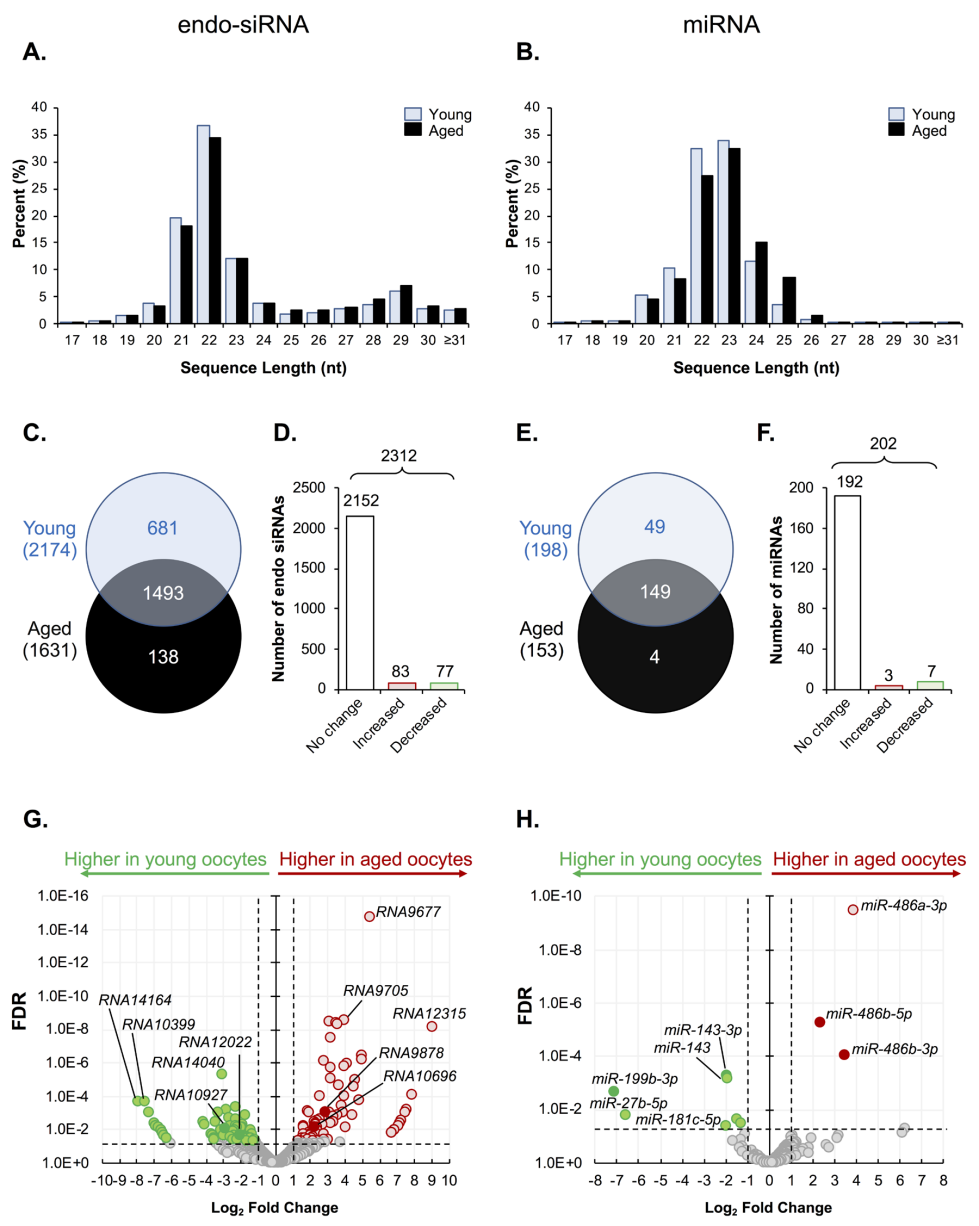
**Endo-siRNA and miRNA signatures of young and aged oocytes.** Following filtering and normalization, sRNA reads were mapped to known endo-siRNA and miRNA from RNAcentral sequence database (August 2017, RNAcentral) (https://rnacentral.org/) to explore changes in the endo-siRNA and miRNA landscape between young and aged oocytes. **(A)** Endo-siRNA and **(B)** miRNA sequence length distribution between young and aged GV oocytes. **(C)** Venn diagram illustrating the total number of endo-siRNA and **(E)** miRNA identified in young and aged oocytes. **(D)** Graphical representation of the proportion of endo-siRNAs and **(F)** miRNAs, identified as being expressed at equivalent levels (unchanged) or that were up- or down-regulated (increased or decreased, respectively) between young and aged oocytes. **(G)** Volcano plots depicting log_2_-fold changes (x-axis) and false discovery rate (FDR; y-axis) of endo-siRNAs and **(H)** miRNAs between young and aged oocytes. Solid dots represent sRNAs that were selected for RT-qPCR validation. Counts of ≥ 10 reads aligning to a specific sRNA was used as a threshold for a positive endo-siRNA or miRNA identification in this study. sRNAs experiencing a threshold of ≥ ± 2-fold change and false discovery rate of < 0.05 were considered as being differently expressed between young and aged oocytes.

Our initial studies used RNA-Seq to profile the small RNA landscape of oocytes of young and aged mice. The C57/BL6 × CBA hybrid (F1) strain was utilized as a model for these studies on the basis that they display a similar age-related decline in oocyte quality and oocyte number as that documented in humans. Owing to the low numbers of GV oocytes that can be retrieved from aged F1 females (approximately 5-10 per animal), we elected to perform sRNA profiling on only a single biological replicate that comprised of RNA extracted from pooled oocytes, specifically; 582 and 521 Germinal Vesicle (GV) oocytes from 18 young and 49 aged F1 females were pooled, respectively. Notwithstanding the limitations of this approach, sequencing returned total read numbers of 1,730,546 and 1,595,791 reads from young and aged oocytes, respectively. Of the generated young and aged oocyte libraries, 492,807 (~28%) and 284,439 (~18%) reads mapped to known sRNA species, respectively; including the endo-siRNA (~19% and ~11%), miRNA (~4% and ~3%), and piRNA (~5% and ~4%) species of sRNA (Fig. S1). The remainder of each sequenced library mapped to other known classes of non-protein-coding RNA or regulatory RNA, including the ribosomal RNA (rRNA), small nucleolar RNA (snoRNA), small nuclear RNA (snRNA), and transfer RNA (tRNAs) classes of RNA (Fig. S1). On the basis of this initial profiling exercise, we elected to focus our subsequent experimental analyses on the endo-siRNA and miRNA classes of sRNA owing to previously demonstrated roles in meiosis [12–15] and post-implantation embryo development [17], respectively. The length distribution of the sequenced and mapped endo-siRNAs and miRNAs revealed a tendency for sRNAs of between 21 to 24 nucleotides (nts) in both young and aged oocytes (Fig. 1A and B). In the case of the endo-siRNA class, sRNAs of 29 nts in length also readily accumulated in young and aged oocytes. Notably, the documented length distribution of the endo-siRNA and miRNA sRNAs identified in this study is consistent with those reported in previous studies of mouse oocytes [9,10]. After imposing a read threshold of ≥10 reads in at least one of the sample groups, a total of 2,312 unique endo-siRNAs, and 202 unique miRNAs were identified in young and aged oocytes (Tables S2 and S3).

Among the total of 2,312 unique endo-siRNAs identified in this analysis, endo-siRNAs *RNA14033, RNA14037, RNA14036, RNA14043, RNA14041,* and *RNA9780,* returned the highest read numbers in both young and aged GV oocytes, collectively accounting for ~2% of all detected endo-siRNAs. In a similar context, of the 202 unique miRNAs detected, *mmu-miR-486b-5p, mmu-miR-182-5p, mmu-miR-871-3p, mmu-miR-470-5p* and *mmu-miR-16-1* were the most abundant in both populations of oocytes, accounting for ~4.5 and ~5.5% of all miRNA that accumulated in GV oocytes of young and aged mice, respectively. A decrease in the complexity of both the endo-siRNA and miRNA landscape was observed in oocytes of aged mice with 2,174 and 1,631 endo-siRNAs, and 198 and 153 miRNAs being detected in young and aged oocytes, respectively. More specifically, 1,493 (65%) conserved endo-siRNAs were identified in both populations of GV oocytes, whilst 681 (29%) were classified as unique to young GV oocytes, and a further 138 (6%) were exclusively detected in aged GV oocytes (Fig. 1C). Similarly, 149 (74%) miRNAs were detected in both young and aged GV oocytes, whilst 49 (24%) were exclusive to young GV oocytes and only 4 (2%) were uniquely detected in aged GV oocytes (Fig. 1E). Following normalization based on the Reads Per Kilobase Million (RPKM) value of each detected sequence, sRNAs that returned a threshold of ≥ ± 2-fold change and false discovery rate of < 0.05 were considered to differentially accumulate between young and aged oocytes. Notwithstanding the diversity of the endo-siRNA sRNA population identified in young and aged GV oocytes, only 160 (7%) were classified as being differentially abundant based on the application of negative binomial exact tests, with 83 (4%) being more abundant, and 77 (3%) being less abundant in the GV oocytes recovered from aged females (Fig. 1D). A similar trend was also observed for the miRNAs, whereby only 10 miRNAs (5%) were determined to differentially accumulate in aged GV oocytes. Of these, three miRNAs were determined to have elevated abundance, while the remaining seven miRNAs were reduced in abundance in aged GV oocytes (Fig. 1F). Despite these modest numbers of differentially accumulating sRNAs, their changes in abundance between young and aged GV oocytes ranged from between 2.3- to 510-fold, and 2.5- to 114-fold, for endo-siRNAs and miRNAs, respectively (Fig. 1G and H).

### Validation of oocyte endo-siRNA and miRNA abundance

**Figure 2:**
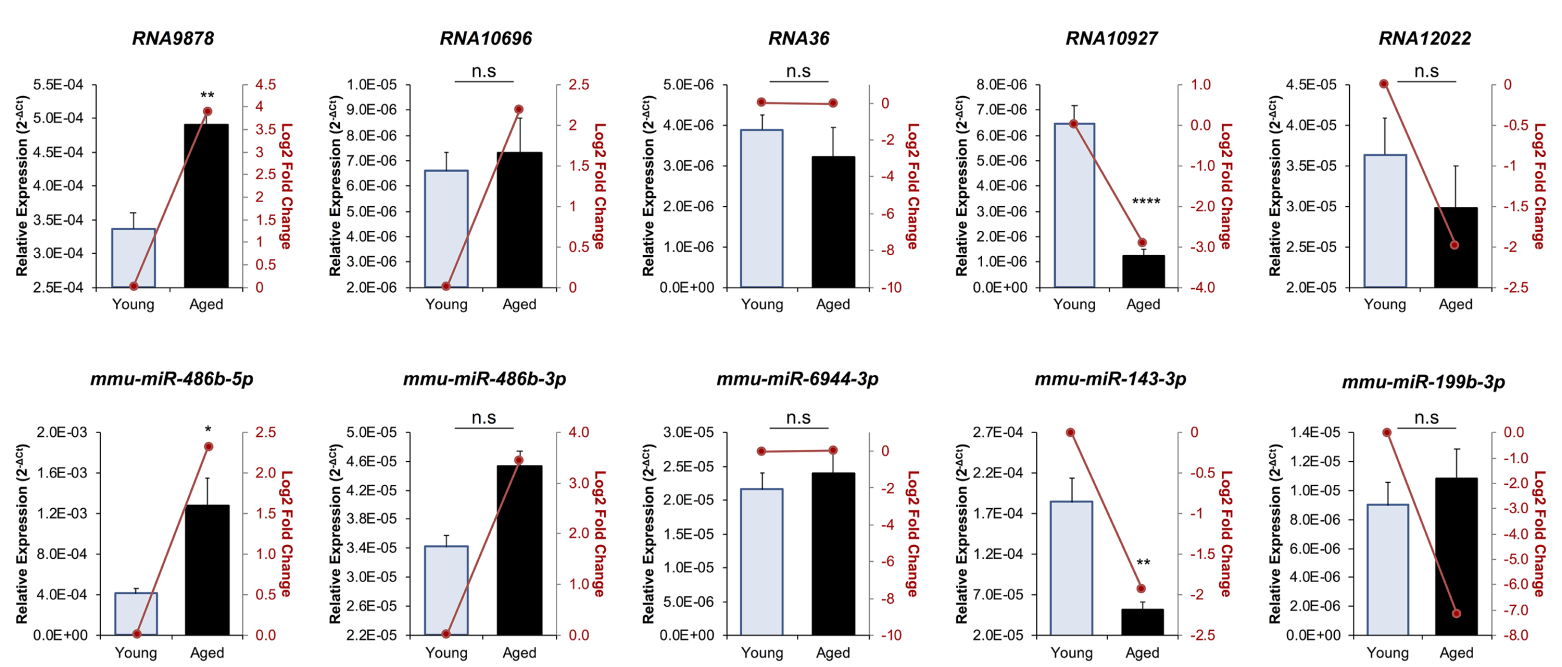
**RT-qPCR validation of miRNA and endo-siRNA abundance in young and aged oocytes.** To validate RNA-Seq data, five endo-siRNAs and five miRNAs were selected for quantification using RT-qPCR. Candidate sRNA included four representatives with increased expression in aged oocytes (*RNA9878, RNA10696, mmu-miR-486b-5p,* and *mmu-miR-486b-3p*), four with decreased expression in aged oocytes (*RNA10927, RNA12022,* mmu-miR-143-3p, and *mmu-miR-199b-3p*), and two that remained at equivalent levels (*RNA36* and *mmu-miR-6944-3p*). cDNA generation and RT-qPCR experiments were performed in technical and biological triplicate, with each biological replicate comprising 10 oocytes randomly sampled from a pool of oocytes isolated from three animals. The U6 small nuclear RNA was employed as an endogenous control to normalize the expression levels of target sRNAs. Values are shown as a mean of all replicates ± SEM. Statistical analyses were performed using Student’s t-test, * *p* < 0.05, ** *p* < 0.01, **** *p* < 0.0001. Log_2_ fold changes based on RNA-Seq are represented as dark red line graphs while the relative abundance (2^-ΔCt^) of each sRNA determined by RT-qPCR is represented by columns.

To confirm the abundance trends identified via sequencing, the relative abundance of five endo-siRNAs and five miRNAs was quantified via RT-qPCR (Fig. 2). The quantified sRNAs were either identified as having increased (*RNA9878, RNA10696, mmu-miR-486b-5p* and *mmu-miR-486b-3p*), equivalent (*RNA36* and *mmu-miR-6944-3p*), or decreased (*RNA10927, RNA12022, mmu-miR-143-3p* and *mmu-miR-199b-3p*) abundance in the aged GV oocyte. This strategy confirmed that each of the five targeted endo-siRNAs displayed the accumulation trends identified via sequencing. Indeed, the level of the *RNA9878* was demonstrated to be significantly elevated (*p* = 0.0031), whilst the level of endo-siRNA *RNA10927* was significantly reduced (*p* > 0.0001). In addition, RT-qPCR revealed that as expected, the level of endo-siRNA *RNA36* remained unchanged (*p* = 0.4944) across young and aged GV oocytes. Whilst not statistically significant, the abundance of both *RNA10696* and *RNA12022* trended in the same direction as indicated by sRNA sequencing. Of the five miRNAs quantified by RT-qPCR, four exhibited trends that agreed with the sequencing- data. Specifically, *mmu-miR-486b-5p* (*p* = 0.0109) and *mmu-miR-143-3p* (*p* = 0.0038), displayed significantly elevated and reduced abundance in aged GV oocytes respectively, and further, the levels of the *mmu-miR-6944-3p* (*p* = 0.587) did not change. In addition, *mmu-miR-486b-3p* followed the trend identified by sequencing; however, its altered abundance was not statistically significant between young and aged GV oocytes. Of the 10 sRNA experimentally validated, only *mmu-miR-199b-3p* was determined to have an opposing accumulation profile when assessed via the orthogonal approaches of RNA-Seq and RT-qPCR.

### mRNA target prediction of endo-siRNA and miRNA

**Figure 3:**
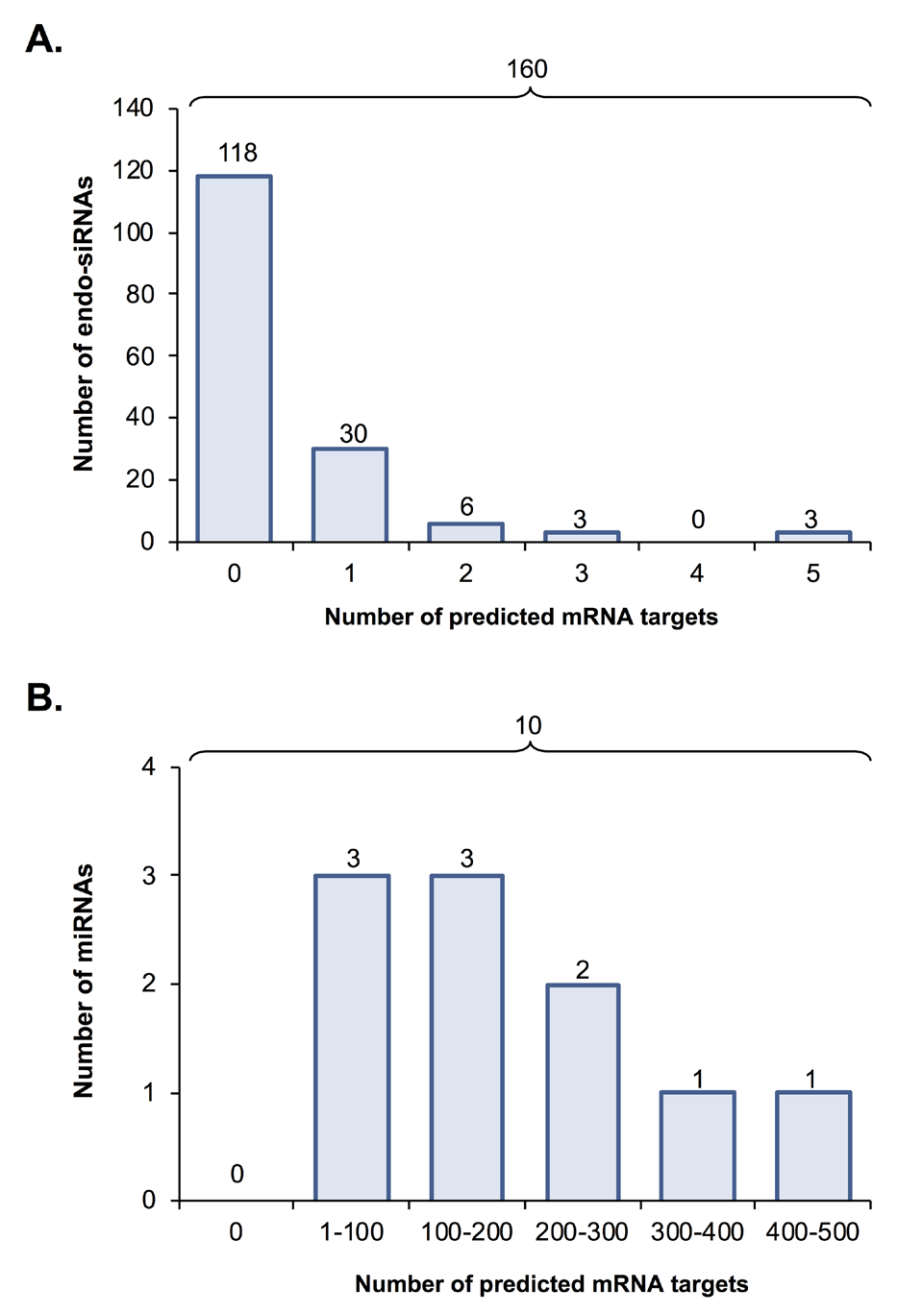
**Predicted mRNA targets of differentially expressed endo-siRNA and miRNA.** Endo-siRNA and miRNA sequences determined to be differentially expressed between young and aged oocytes (i.e. threshold of ≥ 2-fold change and false discovery rate of < 0.05) were surveyed using NCBI and miRDB target prediction algorithms, respectively to identify putative mRNA targets. The number of mRNA targets of **(A)** endo-siRNA and **(B)** miRNA predicted on the basis of this analysis are depicted.

To gain further insight into the biological consequences of sRNA dysregulation in aged oocytes, endo-siRNAs and miRNAs determined to have altered abundance across young and aged GV oocytes were curated based on target prediction algorithms. Of the 160 differentially abundant oocyte endo-siRNAs, only 42 (26%) were determined to putatively target a mRNA for expression regulation (this analysis was conducted using the standard nucleotide BLAST search function of the NCBI database and each endo-siRNA as the search query) (Fig. 3A). Amongst these 42 endo-siRNAs, 30 were identified as having only a single mRNA target, whilst the remaining 12 endo-siRNA assessed via this approach were determined to putatively target between two to five mRNA transcripts for expression regulation (Fig. 3A and Table S4). As anticipated on the basis of lower stringency of targeting complementarity, the 10 miRNA sRNAs determined to be differentially abundant in young and aged GV oocytes were revealed to potentially regulate the expression of a much larger mRNA cohort. More specifically, with the exception of three of these miRNAs, which potentially target between 1 to 100 mRNAs for expression regulation, the remaining seven s may target as many as 100 to 500 unique mRNA transcripts for expression regulation (Fig. 3B and Table S5). Since miRNA function is globally suppressed in mouse oocytes and early embryos [17], we elected to focus our subsequent attention on the endo-siRNA sRNA class and their putative targets within the oocyte.

**Figure 4:**
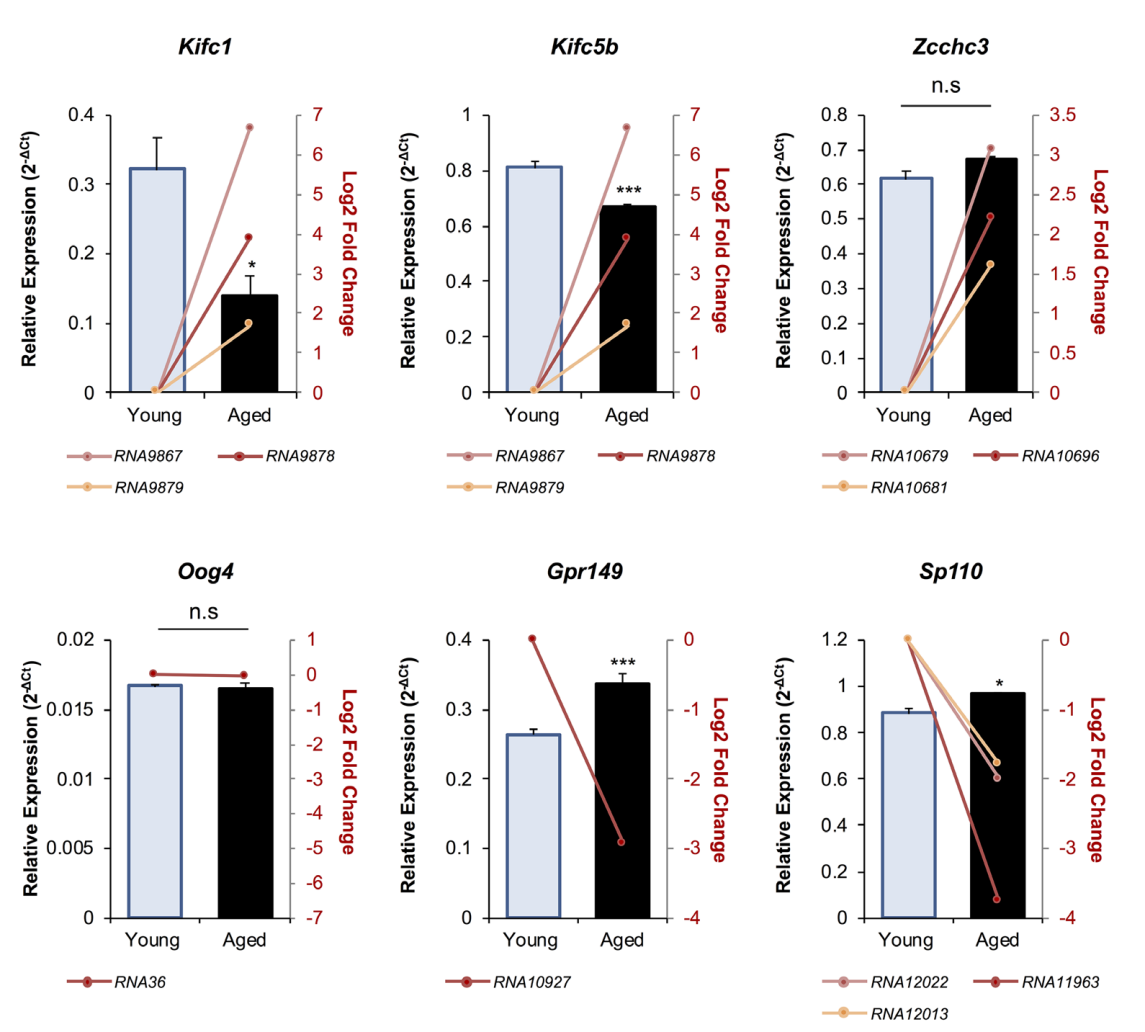
**The expression of putative mRNA target genes of endo-siRNAs differentially abundant in young and aged oocytes.** To verify that the changes in endo-siRNA abundance led to altered target gene expression in aged oocytes, six mRNAs were selected for RT-qPCR assessment. Candidate mRNAs included three representatives with targeting endo-siRNAs whose abundance was upregulated in aged oocytes (*Kifc1*, *Kifc5b*, and *Zcchc3*), two that were potentially targeted by downregulated endo-siRNAs (*Gpr149* and *Sp110*) and one mRNA potentially targeted by an endo-siRNA with unchanged abundance in mRNA potentially targeted by an endo-siRNA with unchanged abundance in between young and aged oocytes (*Oog4*). cDNA generation and RT-qPCR experiments were performed in technical and biological triplicate, with each biological replicate comprising 10 oocytes randomly sampled from a pool of oocytes isolated from three animals. *Ppia* was employed as an endogenous control to normalize the expression levels of target mRNAs. Values are shown as a mean of all replicates ± SEM. Statistical analyses were performed using Student’s t-test, * *p* < 0.05, *** *p* < 0.001. Log_2_ fold changes based on RNA-Seq are represented as pink, red, and orange line graphs while the relative abundance (2^-ΔCt^) of each sRNA determined by RT-qPCR is represented by columns.

A total of 39 unique mRNAs were identified as potential targets of the differentially abundant endo-siRNAs in the oocytes of young and aged mice (Table 1). Notably, several of these putative mRNA targets harbored multiple sequences complementary to the endo-siRNAs under assessment, with the most commonly targeted mRNAs including; *Kif4* (targeted by 7 endo-siRNAs *Rangap1* (targeted by 5 endo-siRNAs), and the *Kifc1*, *Kifc5b*, *Zcchc3*, *Sp110*, *LC677525*, and *LOC664787* transcripts which are all potential targets of three endo-siRNAs. To begin to explore the biological consequences of altered endo-siRNA abundance in aged GV oocytes, the relative level of six putative mRNA target transcripts was quantified via RT-qPCR (Fig. 4). Selected mRNAs included those with targeting endo-siRNAs that were uniformly up-regulated (*Kifc1*, *Kifc5b*, and *Zcchc3*), unchanged (*Oog4*), or down-regulated in aged oocytes (*Gpr149* and *Sp110*). Of the six mRNAs analyzed, five displayed reciprocal abundance trends to that of their putatively targeting endo-siRNAs. Indeed, the expression of *Kifc1* (*p* = 0.0113) and *Kifc5b* (*p* = 0.0009) significantly decreased, *Oog4* levels remained unchanged (*p* = 0.7809), and *Gpr149* (*p* = 0.0005) and *Sp110* (*p* = 0.0295) expression significantly increased in aged GV oocytes. The one exception was the *Zcchc3* transcript, whose abundance remained unchanged even though the levels of its putatively targeting endo-siRNAs were elevated. One possible explanation for this curious observation may be that the levels of the targeting endo-siRNAs were not altered to a degree that could influence the abundance of the *Zcchc3* transcript. Of note, a similar result has been observed with *Kif4* transcript abundance remaining unchanged in aged F1 GV oocytes despite an increase in the abundance of the targeting endo-siRNA observed here [19].

**Table 1:** mRNA targeted by differentially expressed endo-siRNA.

**Gene name**	**Expression of targeting siRNA(s)^1^**
*Rangap1*	↑↑↑↑↓
*Kif4*	↑↑↑↑↑↓↓
*Acnat1*	↑
*Rest*	↑
*Prc1*	↑↓
*Dcun1d5*	↑
*Zcchc3*	↑↑↑
*Kifc1*	↑↑↑
*Kifc5b*	↑↑↑
*Tubb4b*	↑
*Tubb2a*	↑
*Tubb2b*	↑
*Tubb3*	↑
*Tubb6*	↑
*Hmmr*	↑
*Lgalsl*	↓
*Kcnk13*	↑
*F420015M19Rik*	↓↓
*Gpr149*	↓
*Gm5148*	↑
*C330021F23Rik*	↑
*Ube2l3*	↑↓
*LOC100861784*	↓
*Gm10705*	↑↓
*LOC100041057*	↓↓
*C130026I21Rik*	↓↓
*Sp110*	↓↓↓
*LOC102638047*	↓↓
*LOC677525*	↓↓↓
*Myo1h*	↓
*LOC546061*	↓
*Snx22*	↓
*Wdr31*	↑
*Gm14149*	↑
*Gramd1c*	↑
*Esp24*	↓
*Kif2a*	↓
*Cdc5l*	↓
*Rps23*	↑

^1^ The direction of the altered abundance trend of each mRNA targeting endo-siRNA in aged, compared to young oocytes, is indicated by the up (enhanced abundance) and down (reduced abundance) arrows.

Of these putative targets, *Kifc1* and *Kifc5b* were selected for further investigation on the basis that each of their targeting endo-siRNAs (*RNA9878*, *RNA9867* and *RNA9879*) were elevated in aged GV oocytes; the uniformity of these changes in endo-siRNA abundance raised the prospect that the targeted mRNAs may be reciprocally repressed in their abundance in aged GV oocytes. The potential reduced *Kifc1* and *Kifc5b* expression in aged oocytes holds additional significance since pharmacological inhibition of KIFC1 (and other members of this family, such as KIFC5B) has been demonstrated to have a negative impact on spindle formation in mouse oocytes, a phenotype that is consistent with the age-related dysfunction witnessed in these cells [18,20]. Accordingly, the following experiments we designed to examine the impact that elevated abundance of the *Kifc1* and *Kifc5b* targeting endo-siRNAs has on the integrity of meiosis in aged GV oocytes.

### Kifc1 and Kifc5b expression in oocytes

**Figure 5:**
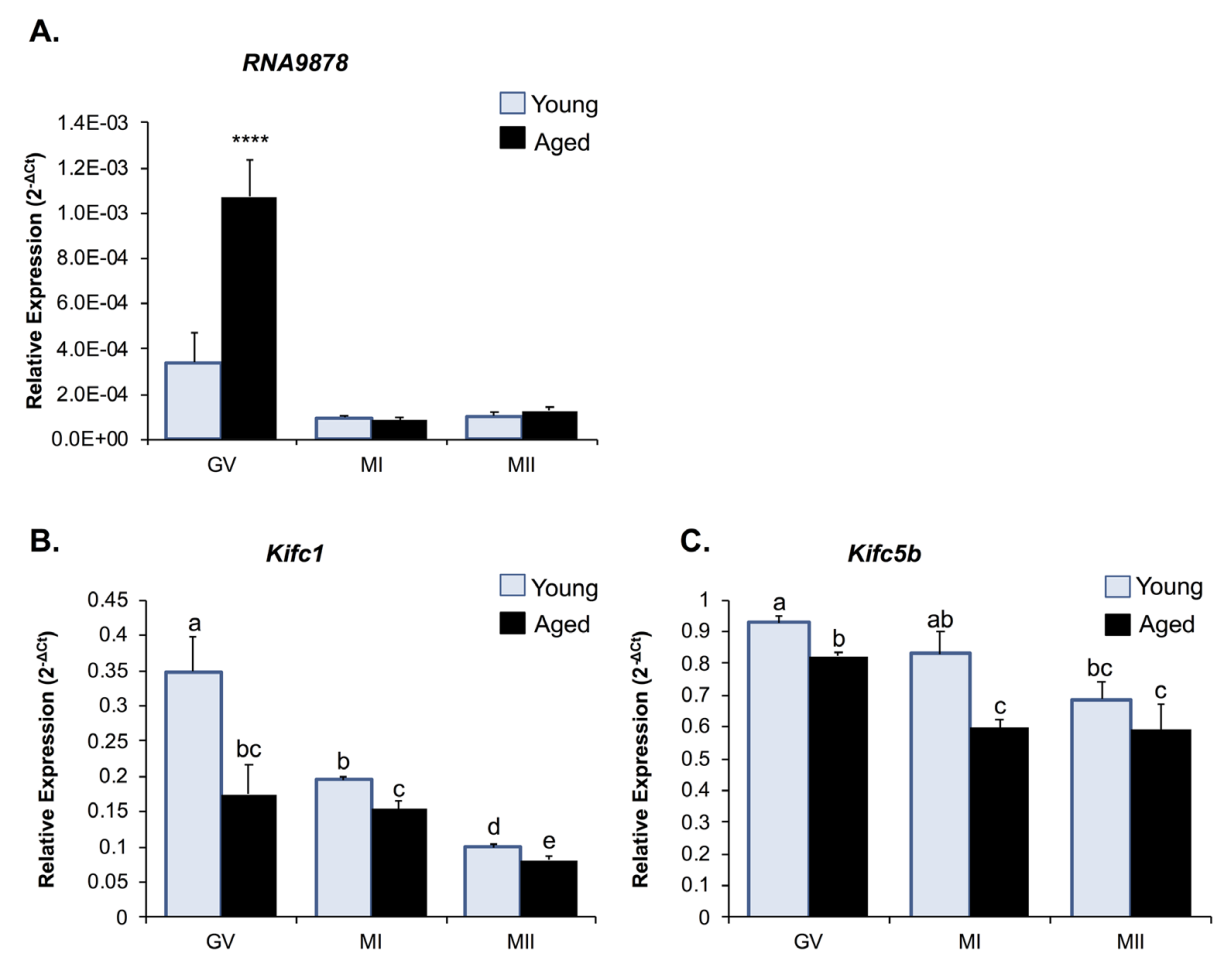
**Expression of Kifc1 and Kifc5b targeting endo-siRNA and mRNA in young and aged oocytes throughout meiosis.** RT-qPCR of young and aged GV, MI and MII stage oocytes was utilized to determine the impact of increased *Kifc1* and *Kifc5b* targeting endo-siRNAs on *Kifc1* and *Kifc5b* mRNA expression during meiosis. **(A)** RT-qPCR of *RNA9878* in young and aged GV, MI, and MII oocytes (ANOVA; *p* > 0.0001). RT-qPCR of **(B)** *Kifc1* (ANOVA; *p* ≥ 0.0266) and **(C)** *Kifc5b* (ANOVA; p ≥ 0.0128) in young and aged GV, MI, and MII oocytes. cDNA generation and RT-qPCR experiments were performed in technical and biological triplicate, with each biological replicate comprising 10 oocytes randomly sampled from a pool of oocytes isolated from three animals. The U6 small nuclear RNA and *Ppia* were employed as endogenous control to normalize the expression levels of target sRNAs and mRNAs, respectively. Values are shown as a mean of all replicates ± SEM. **** *p* < 0.0001.

As a reflection of the high level of *Kifc1* and *Kifc5b* mRNA sequence identity (i.e. 97%; Fig. S2), both transcripts are potentially targeted by the endo-siRNAs, *RNA9878*, *RNA9867*, and *RNA9879* (Table S6). Thus, to explore the potential regulatory roles of these three endo-siRNAs, we first examined the ontogeny of the levels of the *RNA9878* sRNA and its putative *Kifc1* and *Kifc5b* target transcripts during the *in vitro* maturation of young and aged oocytes (that is; GV, MI, and MII stage oocytes; Fig. 5). Consistent with our RNA-Seq data, *RNA9878* was more highly abundant in aged versus young GV oocytes (3.24-fold increase) (Fig. 5A). However, *RNA9878* abundance was significantly reduced (*p* > 0.0001), and remained low, between young and aged oocytes as they progressed through the MI and MII stages of development (Fig. 5A). Conversely, we identified a reciprocal reduction in the expression of *Kifc1*, and to a lesser degree *Kifc5b*, in aged GV oocytes compared to their younger counterparts (Fig. 5B and 5C). This profile of reduced *Kifc1* and *Kifc5b* expression persisted into MI, and in the case of *Kifc1,* MII stage oocytes (Fig. 5B and 5C). These results provide correlative support for an age-dependent endo-siRNA mediated degradation of *Kifc1* and *Kifc5b* expression in GV stage oocytes, the legacy of which persists in the latter MI and MII stages of oocyte development, which is unsurprising due to the transcriptionally silent nature of meiotically competent oocytes.

**Figure 6:**
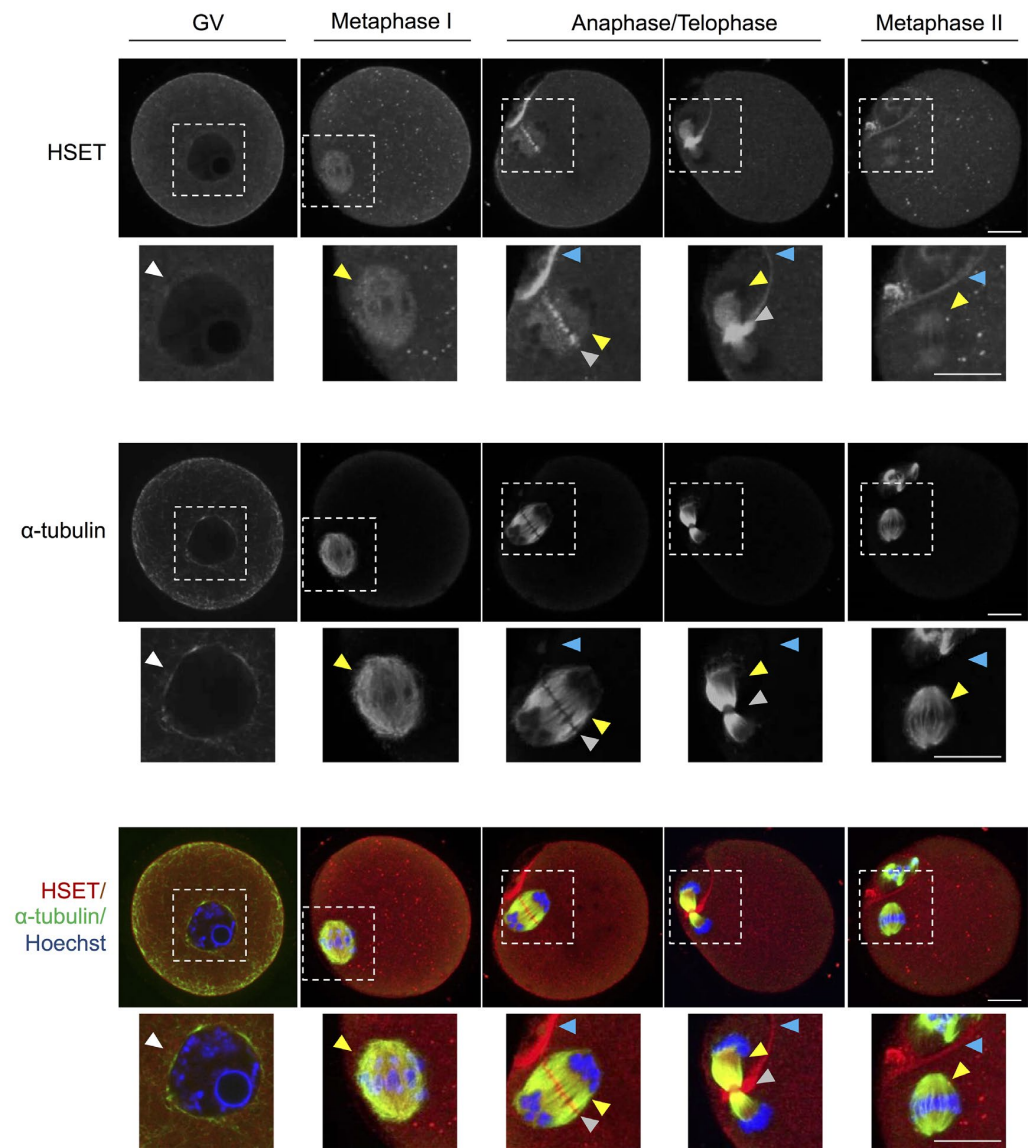
**HSET expression throughout oocyte meiosis.** Immunofluorescence analysis was utilized to track the spatial profile of HSET distribution in GV, MI, anaphase I/telophase I, and MII stage oocytes. Inserts highlight the localization of HSET to the nuclear envelope (white arrowheads), microtubules (yellow arrowheads), between the chromosomes (grey arrowheads), and at the partitioning of the polar body (blue arrowheads). Oocytes were dual labelled with anti-HSET and anti-α-tubulin antibodies followed by either appropriate goat anti-rabbit 633 Alexa Fluor (red) or goat anti-mouse 488 Alexa Fluor-conjugated (green) secondary antibodies, respectively. Oocytes were then counterstained with the nuclear stain Hoechst 33342 (blue) and viewed using confocal microscopy. Scale bar = 20 μm. These experiments were repeated using three independent biological replicates, with each comprising a minimum of 10 oocytes, and representative images are shown.

To further elucidate the impact of age-associated down-regulation of *Kifc1* and *Kifc5b* expression, we next sought to assess the abundance of the KIFC1 and KIFC5B proteins in oocytes throughout GV, MI, anaphase, telophase and MII phases of development (Fig. 6). As an important caveat for interpretation of these data, KIFC1 and KIFC5B share ~98% amino acid sequence identity (Fig. S3), and thus commercially sourced antibodies that are capable of distinguishing between these two proteins are unavailable. Accordingly, the data presented are based on the detection of both proteins, which are hereafter referred to as HSET; the alternate name for the kinesin 14 family, which encompasses both KIFC1 and KIFC5B. Immunofluorescence analysis of anti-HSET and anti-α-tubulin antibodies revealed strong co-localization in oocytes throughout their meiotic division. This finding is consistent with a functional role for HSET as a microtubule crosslinker in oocytes [20]. Additional foci of HSET localization were also detected within the vicinity of the nuclear envelope in GV stage oocytes and at the spindle of MI and MII oocytes. Notably, HSET also appeared to accumulate between α-tubulin labeling during the separation of the maternal chromosomes at anaphase and telophase and at the interface of the partitioning polar body from as early as the anaphase stage of development. The specificity of the immunofluorescence analyses was validated through the absence of fluorescent labeling in any negative control groups; consisting of either anti-mouse or anti-rabbit IgG negative controls (Fig. S4).

**Figure 7:**
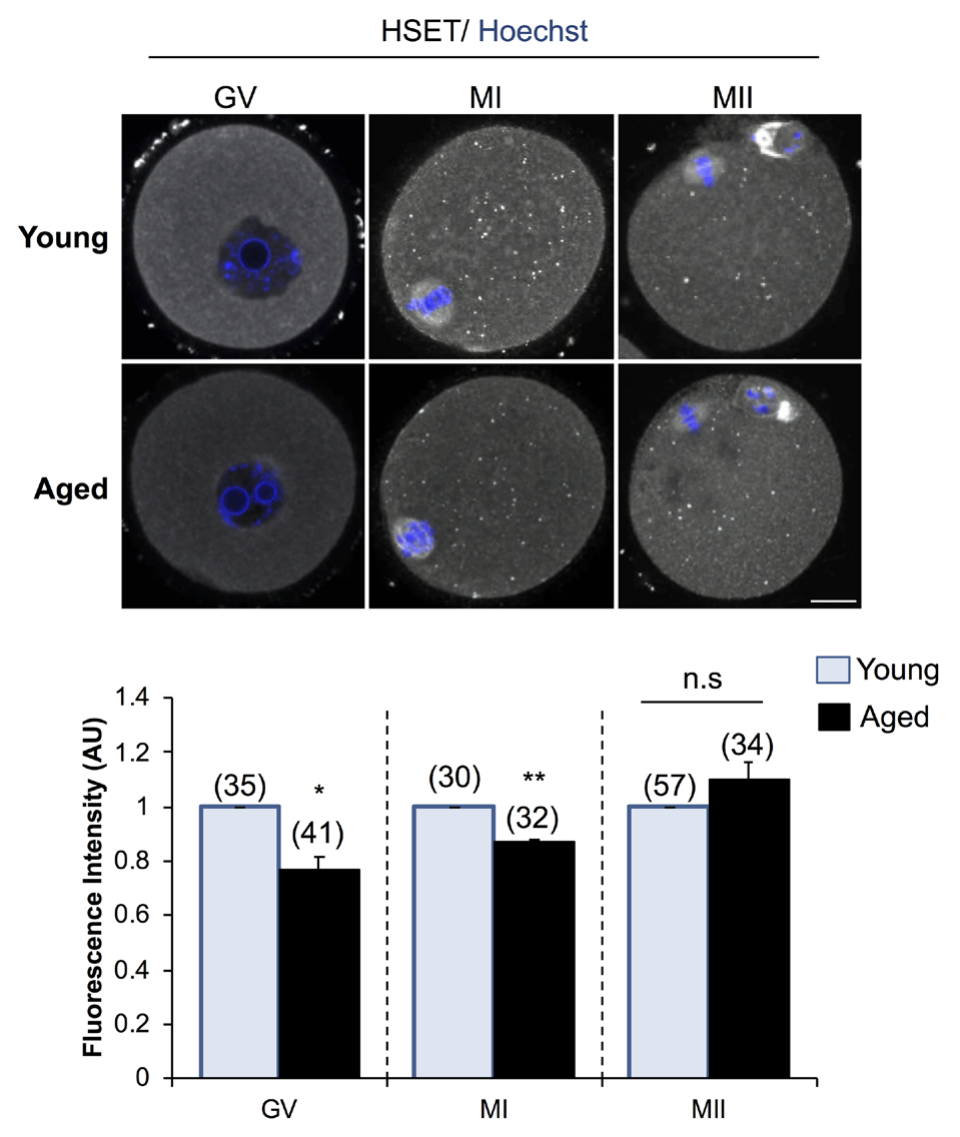
**Expression of HSET protein in young and aged oocytes.** Immunofluorescence analysis of HSET in young and aged GV, MI, and MII stage oocytes was utilized to determine whether an age-related decrease in *Kifc1* and *Kifc5b* transcript abundance presages an equivalent decrease in protein abundance. Oocytes were labelled with anti-HSET antibodies followed by goat anti-rabbit 633 Alexa Fluor-conjugated (grey) secondary antibodies. Oocytes were then counterstained with the nuclear stain Hoechst 33342 (blue) and viewed using confocal microscopy. Scale bar = 20 μm. These experiments were repeated using three independent biological replicates, with each comprising a minimum of 10 oocytes, and representative images are shown. The immunofluorescence intensity of the entire cell was calculated for each oocyte as described in the Materials and Methods, and the mean of each biological replicate values ± SEM are shown. Statistical analyses were performed using Student’s t-test, * *p* < 0.05, ** *p* < 0.01. AU, arbitrary units.

In view of these findings, we sought to determine whether decreased *Kifc1* and *Kifc5b* transcript abundance is reflected at the protein level in aged oocytes (Fig. 7). Based on immunofluorescence labeling, the HSET proteins displayed a significant age-dependent decrease in abundance, amounting to a 1.30-fold (*p =* 0.0104) and 1.15-fold (*p =* 0.0009) reduction in GV and MI stage oocytes, respectively. These trends were recapitulated in immunoblotting experiments, which confirmed a decrease in the staining intensity of the predominant band of the appropriate molecular weight for HSET (i.e. ~74 kDa) in lysates of aged GV, MI and, to a lesser extent, MII stage oocytes (Fig. S5). On the basis of these correlative data, we infer that an age-associated increase in the abundance of the *Kifc1* and *Kifc5b* targeting endo-siRNAs may contribute to the decreased expression of *Kifc1* and *Kifc5b* and the accumulation of the HSET proteins in aged oocytes; changes that may ultimately impact on the fidelity of oocyte meiosis.

### Examination of endo-siRNA-mediated HSET knockdown

**Figure 8:**
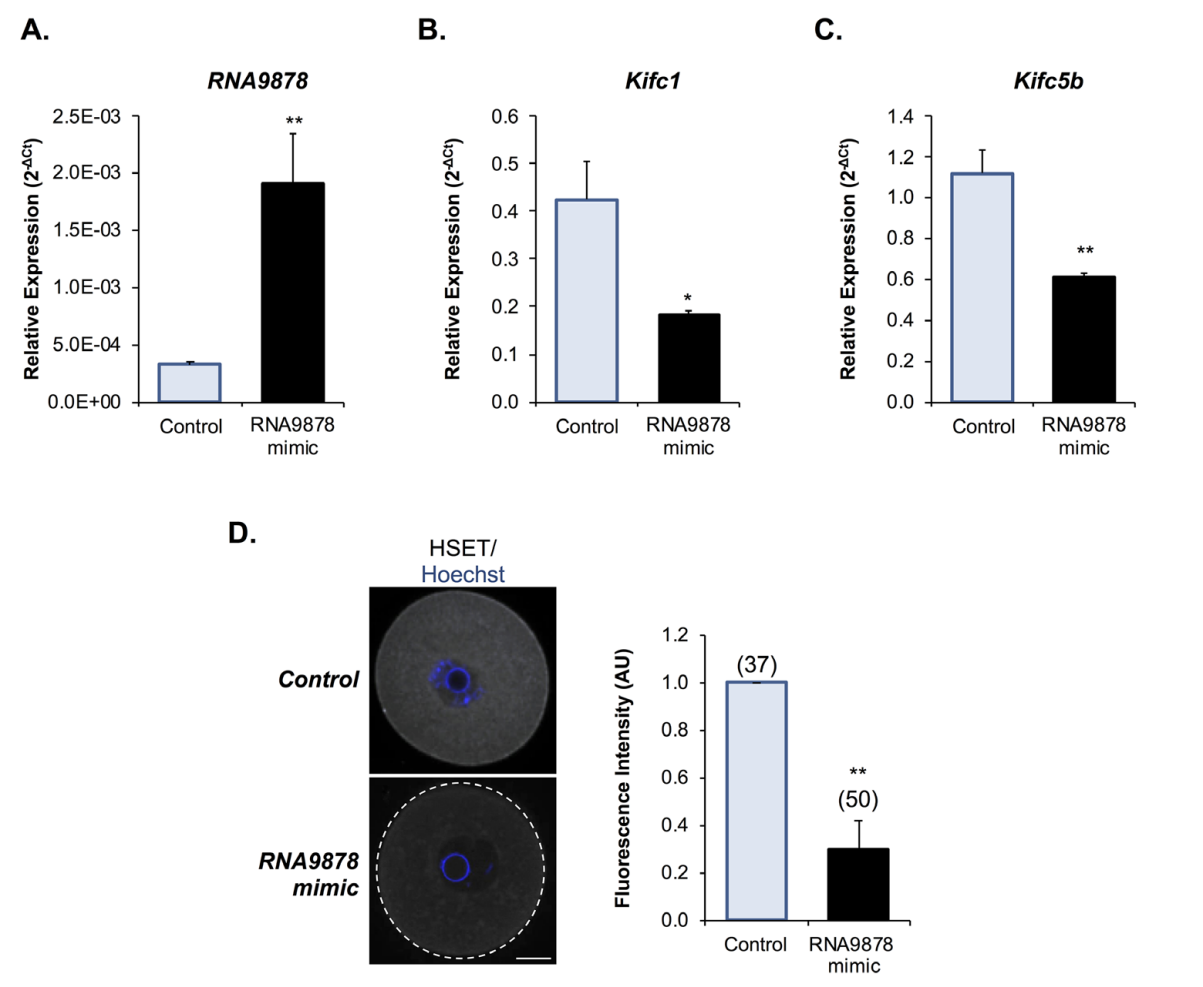
**Examination of endo-siRNA target gene knockdown.** To confirm the functional significance of the endo-siRNA, *RNA9878*, GV oocytes were microinjected with a synthetic *RNA9878* small RNA mimic or a non-targeting negative control. **(A)** To confirm successful microinjection of the *RNA9878* mimic, the expression of *RNA9878* was assessed via RT-qPCR immediately after injection. At 24 h post-injection, the relative levels of **(B)** *Kifc1* and **(C)** *Kifc5b* were assessed in non-targeting and *RNA9878* mimic injected oocytes via RT-qPCR. The U6 small nuclear RNA and *Ppia* were employed as endogenous controls to normalize the expression levels of the target endo-siRNA and mRNAs, respectively. **(D)** Non-targeting and *RNA9878* mimic injected oocytes were then fixed and the expression of HSET was examined. Oocytes were labelled with anti-HSET antibodies followed by goat anti-rabbit 633 Alexa Fluor-conjugated (grey) secondary antibodies. Oocytes were then counterstained with the nuclear stain Hoechst 33342 (blue) and viewed using confocal microscopy. Scale bar = 20 μm. RT-qPCR experiments were performed in technical and biological triplicate, with each biological replicate comprising 10 oocytes randomly sampled from a pool of oocytes isolated from three animals. Similarly, immunofluorescence experiments were repeated on three biological replicates, with each replicate comprising a minimum of 10 oocytes randomly sampled from a pool of oocytes isolated from three animals. Values are shown as a mean of each replicate ± SEM. Statistical analyses were performed using Student’s t-test, * *p* < 0.05, ** *p* < 0.01. AU, arbitrary units.

**Figure 9:**
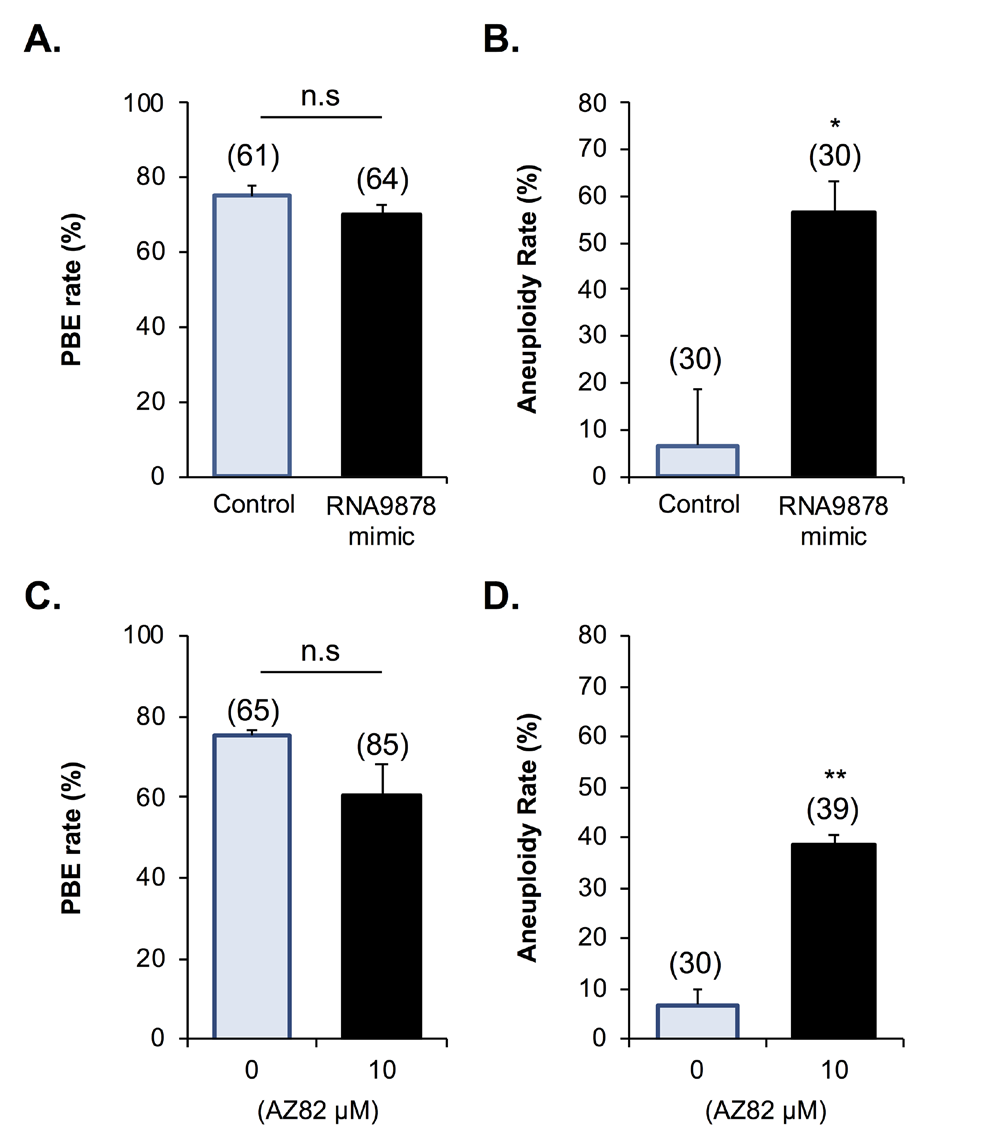
**Biological impact of endo-siRNA target gene knockdown and HSET inhibition.** To confirm the biological significance of the endo-siRNA-mediated knockdown, *RNA9878*, GV oocytes were microinjected with a synthetic *RNA9878* small RNA mimic, a non-targeting negative control, or subjected to pharmacological HSET inhibition (AZ82; 10 μM). (A) Non-targeting, RNA9878 mimic injected, and HSET inhibited oocytes were then subject to IVM to MII where **(A, C)** PBE and **(B, D)** aneuploidy rates were recorded. siRNA-mediated knockdown and HSET inhibition experiments were repeated on three biological replicates, with each replicate comprising a minimum of 20 oocytes randomly sampled from a pool of oocytes isolated from three animals. Values are shown as a mean of each replicate ± SEM. * Statistical analyses were performed using Student’s t-test, *p* < 0.05, ** *p* < 0.01. AU, arbitrary units.

Having confirmed significant changes in the *Kifc1* and *Kifc5b* targeting endo-siRNAs, the *Kifc1* and *Kifc5b* transcripts, and HSET protein within the oocytes of aged mice, we next sought to examine the functional consequences of these changes in terms of the integrity of oocyte meiosis. For the purpose of these studies, we employed a knockdown strategy in which young GV oocytes were injected with mirVana mimics of either the *RNA9878* endo-siRNA or a non-targeting negative control. At 24 h post-injection, oocytes were harvested and the relative expression of *Kifc1* and *Kifc5b* determined by RT-qPCR. As anticipated, the introduction of the *RNA9878* mirVana mimic significantly increased the detectable levels of the *RNA9878* endo-siRNA within the oocyte (5.8-fold increase; *p* = 0.0028) (Fig. 8A). In addition, this strategy proved effective in eliciting a significant reduction in the expression of both the *Kifc1* (Fig. 8B; 2.3-fold decrease; *p* = 0.0126) and *Kifc5b* (Fig. 8C; 1.8-fold decrease; *p* = 0.0016) mRNAs, as well as decreased immunofluorescence detection of HSET protein (Fig. 8D; 3.3-fold decrease; p = 0.0044). Notably, however, the degree of HSET protein knockdown achieved via this approach had no overt impact on polar body extrusion (PBE) rates witnessed during subsequent *in vitro* maturation (IVM) (Fig. 9A). By contrast, aneuploidy rates were significantly elevated by 8.5-fold (*p* = 0.0221) in oocytes injected with the *RNA9878* mimic, such that more than 50% of the injected oocytes displayed evidence of this lesion (Fig. 9B). Notwithstanding the important caveat that the magnitude of HSET knockout achieved in this experiment is much higher than that documented in naturally aged oocytes (Fig. 7), these data affirm the principle that an altered abundance of the *Kifc1* and *Kifc5b* targeting endo-siRNAs in aged GV oocytes can contribute to the increased prevalence of aneuploidy that characterizes these cells. As an additional line of evidence in support of the importance of HSET function, we were able to demonstrate a significant 5.8-fold increase in aneuploidy rates (*p =* 0.0011) in oocytes subjected to pharmacological inhibition of HSET (10 µM AZ82), without any attendant reduction in polar body extrusion rates (Figs 9C and 9D).

## DISCUSSION

This study extends previous work in confirming that maternally derived sRNAs have the potential to exert regulatory control over the developmental competence of mouse oocytes [9,10,12–17,21]. Indeed, our analysis of the global sRNA landscape of young and aged mouse oocytes has highlighted an age-dependent profile alteration to several sRNA classes that could be potentially contributing to the decline in quality of aged oocytes. Chief among these changes, we show that several endo-siRNA and miRNA sRNAs are altered in their abundance in aged oocytes. These findings take on added significance when considered in the context of putative downstream target transcripts, many of which encode proteins that are essential for the fidelity of meiotic division within the mature oocyte. Notably, these targeted proteins include members of the kinesin family, namely KIFC1 and KIFC5B, the siRNA-directed knockdown of which resulted in elevated rates of aneuploidy, equivalent to those reported in aged oocytes [22]. Taken together, these data suggest that endo-siRNAs have the potential to adversely contribute to the phenomenon of age-associated decline in oocyte quality.

The last decade has witnessed a proliferation of studies seeking to profile the sRNA landscape of the mammalian oocyte, and further, to elucidate the physiological roles directed by sRNAs in oocyte growth, maturation, and embryo development [9,10,12–17,21]. Such studies have revealed a rich and complex sRNA landscape in the oocyte and have increasingly implicated the endo-siRNA subclass as playing a central role in the regulation of genes linked to oocyte meiosis. To date, and to the best of our knowledge, studies focusing on the susceptibility of the ovarian environment to an altered global sRNA profile during maternal aging are limited. Indeed, the handful of reports in this field have tended to concentrate on the consequences of alteration to the miRNA landscape. In this context, miRNA profiling of whole ovaries from young and aged mice, identified only 54 miRNAs with altered abundance [23]. The miRNAs belonging to this cohort were determined to target genes for expression regulation belonging to FOXO, mTOR, PI3K/AKT and insulin signaling pathways; pathways that are variously implicated in the maintenance of primordial follicle quiescence, cellular senescence and ovarian cancer development [23]. However, studies of whole ovarian tissue are not entirely representative of the oocyte itself, and thus, alterations in the abundance of miRNAs specifically attributed to the oocyte may be obfuscated by the predominance of somatic tissue-specific miRNA profiles within the ovary. Among the studies that have focused on oocytes, Di Emido *et al.* (2014) reported an age-dependent decrease in *mmu-miR-132* abundance in mouse oocytes; a miRNA that lay just beyond the limit of significance imposed in our study [24]. As an extension of this work, Di Emido and colleagues also demonstrated that aged oocytes were not able to modulate the levels of miRNAs in response to oxidative stress; an insult that has been firmly implicated as a contributory agent in oocyte aging [25–27]. Thus, unlike their young counterparts, aged oocytes failed to increase the abundance of *mmu-miR-132* in response to oxidative stress [24]. Differential accumulation profiles of 12 miRNAs have also been documented in MII oocytes from women of advanced maternal age, with the majority of these miRNAs demonstrated to regulate the expression of transcripts encoding proteins implicated in pluripotency, chromatin remodeling and early embryo development [28]. Among these miRNAs with altered abundance in human MII oocytes of women of advanced maternal age, four mouse homologues were demonstrated here to be similarly upregulated in aged MII oocytes, indicating that sRNA dysregulation may similarly influence both human and mouse oocyte aging. Whilst, only one of these miRNAs, *mmu-miR-203-3p*, was detected in GV mouse oocytes in our study, it nevertheless displayed a similar trend of increased abundance (2.75-fold increase) in aged oocytes.

Building on this knowledge platform, our study has examined the extent to which changes in sRNA abundance can contribute to the pathology of maternal aging in oocytes, revealing 42 and 1613 potential mRNA targets respectively, for the endo-siRNAs and miRNAs with altered abundance in aged oocytes. Despite this relatively large number of putative miRNA target genes, we consider it unlikely that alterations to the abundance of the targeting miRNAs exert anything other than a modest influence over the phenotype of aged oocytes. Support for this conclusion rests with independent evidence that miRNAs do not overtly influence target transcript expression in mouse oocytes [9,10,14,16]. Rather, it has been proposed that suppression of miRNA activity during oocyte growth is an early reprogramming event necessary for the accumulation of maternal transcription factors involved in the downstream establishment of the pluripotent blastomeres within the embryo [16,29]. However, we currently have a limited understanding of why, or how, oocyte miRNAs are repressed prior to this stage of development. One possible explanation is the loss of processing bodies (P-bodies) in the mature oocyte, which only reappear in the blastocyst [30,31]. In this context, miRNA destabilization typically occurs in P-bodies and their loss during oocyte development could account for the inability of miRNAs to regulate the expression of their targeted transcripts [32]; however, this has yet to be experimentally confirmed. Irrespective of the potential mechanism, it is curious that an equivalent phenomenon of repression of miRNA activity has not yet been demonstrated in the oocytes of any other mammalian species. In bovine oocytes, for instance, several studies have implicated miRNAs in the regulation of oocyte-specific maternal effect genes, including *Nobox*, *Npm2*, and *Flgla* [33–35]. Nevertheless, maternally acquired miRNAs do not appear to be entirely redundant in the mouse oocyte, with putative roles in post-implantation embryo development being inferred for this class of regulatory sRNA on the basis of significantly reduced litter sizes in *Zp3-Dgcr8* cKO animals [17]. This raises the prospect that an aberrant accumulation profile of miRNAs within aged mouse oocytes could influence downstream signaling associated with the post-implantation phases of embryonic development.

One of the most striking findings of this study was that a subset of kinesin proteins was susceptible to endo-siRNA-mediated downregulation in both aged oocytes, and in oocytes injected with a targeting mirVana mimic. The kinesin superfamily of microtubule motor proteins are of interest owing to the essential role they play throughout both the mitotic and meiotic cell cycle via the regulation of chromosome condensation and alignment, spindle formation, cytokinesis and cell cycle progression [36]. Typically, the function of kinesins can be attributed to two of their functional domains, including an ATP hydrolysis domain that is responsible for powering the movement of the protein along microtubules, and a tail domain that attaches to, and enables transportation of various cargoes [36,37]. HSET belongs to the kinesin 14 family and, unlike other kinesins, the HSET motor domain is positioned at the C-terminus of the protein. This alternate motor domain positioning results in HSET acting in a minus-end directed manner [38]. However, HSET has still been demonstrated essential in the organization of the mitotic spindle owing to its ability to separate and sort antiparallel microtubules into parallel bundles [39–41], and to crosslink two parallel opposing microtubules for their subsequent transport to the spindle poles [39,42]. Consistent with the results of our own study, independent research has confirmed that HSET has a propensity to associate with the spindles of mouse oocytes, as well as the oocytes of a variety of unrelated mammalian species (e.g. bovine, human and non-human primate oocytes) [18,20,43,44].

Based on this evidence, it is not surprising that HSET inhibition at the GV stage of mouse oocyte development leads to delays in downstream spindle bipolarization in MI oocytes and accompanying catastrophic spindle abnormalities in both MI and MII oocytes [18,20]. Similarly, strategies that induce spindle disruption have also been shown to result in the anomalous distribution of HSET [43]. Moreover, the loss of HSET, or suppression of its activity, in the oocytes of model species such as *Drosophila*, manifests in abnormal spindle formation in both MI and MII oocytes [45–49]. More specifically, the spindles in these oocytes took longer to form, were unstable and continually changed shape, and had additional spindle poles as a consequence of pole splitting [46–49]. As a consequence of the pronounced spindle anomalies precipitated by inhibition or loss of HSET function, *Drosophila* oocytes experienced chromosome separation and associated aneuploidy rates at more than double the frequency of control oocytes [46,48,49]. Taken together, these observations may, at least in part, account for our findings that endo-siRNA mediated downregulation of HSET activity resulted in elevated rates of aneuploidy in mouse oocytes. In doing so, they also offer support for our hypothesis that endo-siRNA-directed downregulation of HSET activity may contribute to the age-associated loss of oocyte quality. Ultimately, establishment of the causal nature of this relationship awaits further investigation focused on the ability to improve aging phenotypes via the exogenous expression of KIFC1 and KIFC5B in aged oocytes. We note however, that the interpretation of these experiments would have to take into account the importance of a myriad of alternative mechanisms that contribute toward quality control in the aging oocyte [27].

In summary, our study has provided new insight into the complexity and dynamic nature of the sRNA landscape of aging oocytes. This study has revealed altered abundance to both the endo-siRNA and miRNA classes of sRNA within the oocytes of naturally aged mice. Among the putative targets of endo-siRNAs with elevated abundance in aged oocytes, we identified *Kifc1* and *Kifc5b*, members of the kinesin family of motor proteins implicated in the propagation of meiotic division. Further, the reduced abundance of the HSET protein that the *Kifc1* and *Kifc5b* transcripts encode, either as a consequence of aging or via its selective knockdown in young oocytes, was correlated with increased rates of aneuploidy. Taken together, these data suggest a mechanism by which an altered endo-siRNA profile may contribute to the decline in quality of aged mouse oocytes and thus provide the impetus to explore whether a similar phenomenon is operative in humans.

## MATERIALS AND METHODS

### Animal ethics

Research animals in this study were handled, monitored and euthanized in accordance with NSW Animal Research Act 1998, NSW Animal Research Regulation 2010 and the Australian Code for the Care and Use of Animals for Scientific Purposes 8^th^ Ed. as approved by the University of Newcastle Animal Care and Ethics Committee (approval number A-2011-162). C57/BL6 × CBA hybrid (F1) female mice were bred and housed at the institutes’ Central Animal House Animals under a 12 h light/12 h dark cycle at a constant temperature of 21–22°C and with food and water supplied *ad libitum.* Animals were euthanized via cervical dislocation immediately before use.

### Reagents

All chemicals and reagents used were of research grade and were supplied by Thermo Fisher Scientific (Waltham, MA, USA) or Sigma-Aldrich (St. Louis, MO, USA) unless otherwise specified. Details concerning the purchase and use of primary antibodies for immunolocalization and immunoblotting assays are reported in Table S1. Goat anti-rabbit HRP-conjugated secondary antibodies were obtained from Calbiochem (Cat # DC03L; San Diego, CA, USA). Alexa Fluor 488-conjugated goat anti-mouse (Cat # A-11001), Alexa Fluor 555-conjugated goat anti-human (Cat # A-21433) and Alexa Fluor 633-conjugated goat anti-rabbit (Cat # A-21070) antibodies were purchased from Thermo Fisher Scientific.

### Oocyte collection

Oocytes were isolated as previously described with minor modifications [50]. Briefly, ovaries were removed from non-hormonally stimulated animals immediately after being euthanized. Pre-ovulatory follicles were repeatedly punctured with a 27-gauge needle to release GV oocytes as cumulus-oocyte-complexes into pre-warmed (37°C) M2 media supplemented with 2.5 µM milrinone to maintain GV arrest. Only oocytes with an intact layer of cumulus cells were used. Cumulus cells were mechanically removed via repeated aspiration with a narrow pipette in M2 media at 37 °C. Mice between four to six weeks (young) and 14 to 16 months (aged) of age were utilized for the study of aged oocytes. Approximately half of the aged oocytes in this model exhibit aneuploidy, which is equivalent to that of a woman in her 40’s [22,51,52].

### RNA extraction, library preparation, and small RNA-Seq

Small RNA (<200bp) were extracted from a pool of GV oocytes (i.e. 582 young and 521 aged), which were isolated from 18 young and 49 aged animals. These studies were limited to a single biological replicate due to the limited number of oocytes that can be extracted from aged F1 females (~10 /mouse). Small RNAs were purified using the miRNeasy Mini Kit (Cat. # 217004, Venlo, NV, Netherlands) according to the manufacturer’s instructions. RNA quality was assessed independently at the AGRF whereby each sample was analyzed on an Agilent 2100 Bioanalyzer as per the manufacturers’ instructions (Agilent Technologies, Santa Clara, CA, USA). Upon, passing quality control, samples were subjected to Illumina TrueSeq small RNA sample preparation protocol as per the manufacturers’ instructions (Illumina Inc., San Diego, CA, USA) at the Australian Genome Research Facility (AGRF, Brisbane, QLD, Australia). The samples were again analyzed after small RNA library construction to confirm the size of the products. Finally, sequencing was performed using an Illumina Miseq platform with 50 bp single-end chemistry at AGRF.

### Bioinformatics

Raw sequence reads obtained from AGRF were processed through CutAdapt (https://cutadapt.readthedocs.io/) [53] and FastQC Babraham Bioinformatics, (projects/fastqc/) to identify and remove adapter sequences, possible contaminants, and poor-quality bases. The low-quality ends from reads were trimmed using a cutoff of < 20 Phred quality score. Clipped and quality-trimmed reads shorter than 17 bases in length were discarded. Sequenced reads were then mapped against the *Mus musculus* reference genome 9 (NCBI37/mm9) (June 2007, NCBI) and the RNAcentral sequence database (August 2017, RNAcentral) (https://rnacentral.org/) using Bowtie [54] and Bowtie 2 [55]. Bowtie was initially used with -n alignment default mode selected coupled with -best mode to align the short reads to the reference database; reads producing unique alignments were considered successfully mapped. Reads with more than one valid alignment and reads that could not be aligned were separated from uniquely mapped reads and were processed again using Bowtie 2 with default settings. Again, reads with single valid alignments were considered successfully mapped and combined with the previously mapped reads. Unaligned reads were excluded from further analyses. Mapped reads were then processed using two personalized perl algorithms. Firstly, sequences were separated into RNA type by querying the RNAcentral database to retrieve the relevant information using the individual accession numbers that each mapped read aligned to. Endo-siRNAs were aligned to a previously curated oocyte specific database [10]. The second algorithm calculated and compiled the number of each unique sRNA molecule identified in both sample groups.

To assess differential expression of sRNAs in young and aged oocytes, combined data for both sample groups were imported into R Statistical Software (http://www.R-project.org/) [56] and analyzed using the edgeR package [57,58]. A threshold value of ≥ 10 reads was used as the minimum count required, in at least one sample group, for a sRNA to be considered as present. Reads were normalized by reads per kilobase million (RPKM). A negative binomial exact test was used to determine the differential expression for each individual sRNA. Results were adjusted for multiple testing by the Benjamini and Hochberg’s approach for controlling the false discovery rate (FDR) [59]. The data discussed in this publication have been deposited in NCBI's Gene Expression Omnibus and are accessible through GEO accession number GSE125722 (http://www.ncbi.nlm.nih.gov/geo/query/acc.cgi?acc=GSE125722).

### *In silico* analysis of miRNAs and endo-siRNA target prediction

To begin to assess the function of the differentially expressed miRNAs and endo-siRNA identified in aged and young oocytes, their putative mRNA targets were identified. For miRNA target prediction, miRNA sequences were searched through miRDB (http://www.mirdb.org) and only prediction scores of ≥80 were considered. For endo-siRNA, sequences were blasted in NCBI Standard Nucleotide BLAST (blastn; http://blast.ncbi.nlm.nih.gov) against mouse genomic plus transcript (Mouse G+T) using default parameters [60]. Parameters were automatically adjusted to search for ‘short input sequences.’ Only mRNA transcripts with ≥95% identity, ≥95% query coverage, and a plus/minus strand orientation were considered for further analysis.

### RNA extraction, reverse transcription and qualitative real time PCR

RNA extraction and reverse transcription for sRNA and mRNA was performed using TaqMan MicroRNA Cells-to-CT Kit (Cat # 4391848) or TaqMan Gene Expression Cells-to-CT Kit (Cat # AM1728) as per manufactures instructions, using five oocytes per reaction. Validation of miRNA expression profiles was conducted using a quantitative real-time PCR (RT-qPCR) strategy with TaqMan miRNA assay reagents according to the manufacturer’s instructions (Thermo Fisher Scientific). The miRNAs selected for analysis were *mmu-miR-486-3p* (assay ID. 002093), *mmu-miR-486* (assay ID. 001278), *mmu-miR-199a-3p* (assay ID. 002304), *mmu-miR-143* (assay ID. 002249), and *mmu-miR-6944-3p* (assay ID. 466343_mat). The endo-siRNAs selected for analysis were *RNA9878* (Sequence: CGGGGCCCAGTAGCTAGCAG), *RNA10696* (Sequence: TCGCCATGGCCGCCGTCACCT), *RNA10927* (Sequence: TGCACAGAGACTGGAAGTAGCC), *RNA12022* (Sequence: TCTGGGCACACCTCATCCTTG), and *RNA36* (Sequence: GTTCCACAATCAATCTTCCAGT). The mRNAs selected for analysis were *Kifc1* (assay ID. Mm00835842_g1), *Kifc5b* (assay ID. Mm03011779_m1), *Zcchc3* (assay ID. Mm00613142_s1), *Oog4* (assay ID. Mm00620601_m1) *Gpr149* (assay ID. Mm00805216_m1), and *Sp110* (assay ID. Mm00841342_m1). Real-time PCR was performed using a Light Cycler 96 SW 1.1 (Roche, Castle Hill, Australia). The U6 small nuclear RNA (snRNA) (assay ID. 001973) and Ppia (assay ID. Mm02342430_g1) were used as internal controls to normalize the expression levels of target sRNA and mRNA, respectively. Relative expression levels were calculated using the 2^ΔCt^ method [61]. All RT-qPCR assays were performed in triplicate unless otherwise stated and utilized different oocytes from those employed for RNA-Seq analyses.

### *In vitro* maturation and inhibition studies

For IVM, oocytes were washed out of milrinone by aspiration through four 50 μl droplets of MEM α media (Cat. #11900024) supplemented with 20% (v/v) fetal calf serum, 50 U/ml penicillin, and 50 μg/ml streptomycin. Oocytes were then placed into a single-well IVF dish (Cat. #353653), containing 500 μl of MEM α media. Oocytes were cultured at 37°C in an atmosphere of 5% CO_2_ for 7.5 h for MI, 8.5 h for anaphase/telophase or 16 h for MII stage oocytes. All media and mineral oil were equilibrated at 37°C in an atmosphere of 5% CO_2_ for a minimum of 3 h before use. Following IVM, oocyte maturation was scored, with GV oocytes being identified by the presence of a nuclear envelope and nucleolus and MII oocytes identified via the presence of the first polar body [62,63]. For HSET inhibition studies, the inhibitor, AZ82 (Cat. # 5339160001, Merck) was included in MEM α media during the entirety of IVM at final concentrations of 10 μM as previously described [18]. AZ82 is a selective inhibitor of microtubule-bound HSET motor domain ATPase activity (IC_50_ = 300 nM *in vitro*) [38]. Control oocytes were exposed to equivalent concentrations of the vehicle, dimethyl sulfoxide.

### Knockdown of endo-siRNA targets

In order to assess the biological consequence of endo-siRNA knockdown, GV oocytes were microinjected with the following endo-siRNA mimic sequence; 5' CGGGGCCCAGTAGCTAGCAG 3' (mirVana; Thermo Fisher Scientific). Alternatively, control oocytes were injected with a non-targeting oligo (Cat. # 4464060, Thermo Fisher Scientific). Microinjection of oocytes was performed according to a previously published protocol [64]. Briefly, oocytes were suspended into a 10 µl droplet of M2 media supplemented with 2.5 µM milrinone and held under mineral oil pre-warmed to 37°C. The oocytes were then placed on the heated stage (37°C) of a Nikon TE300-inverted microscope and injected with endo-siRNA mimic or non-targeting oligonucleotide (~5-10 pl at 50 µg/ml) control using micropipettes fabricated from borosilicate glass capillaries (1.5 mm outside diameter; 0.84 mm inside diameter; World Precision Instruments, Sarasota, FL, USA) and attached to a PV820 pneumatic picopump (World Precision Instruments). Injected oocytes were either immediately processed for RT-qPCR analysis to confirm the introduction of the mimic or were allowed to recover for 1 h before being transferred into MEM α media with 2.5 µM milrinone for 24 h at 37°C in 5% CO_2_ to allow time for mRNA and protein degradation. Following recovery, oocytes were processed for RT-qPCR to confirm the degradation of target mRNA. All RT-qPCR assays were performed in triplicate. Alternatively, following 24 h recovery, the aneuploidy status of the oocytes was assessed as previously described [65,66]. Briefly, following IVM to MII, oocytes were incubated with 200 μM monastrol in MEM α for 2 h at 37°C in 5% CO_2_. Oocytes were fixed, and kinetochores were immunostained with anti-CREST antibodies as described below.

### Immunocytochemistry

Live oocytes were washed in phosphate buffered saline (PBS) containing 3 mg/ml polyvinylpyrrolidone (PVP) prior to being fixed and permeabilized in 2% paraformaldehyde (w/v) diluted in PBS with 0.5% Triton X-100 (v/v) for 30 min. Fixed oocytes were blocked in 7% goat serum (v/v) and 1% BSA (w/v) prepared in PBS with 0.1% Tween-20 (PBST) for 1 h at room temperature. Cells were then incubated with appropriate antibodies diluted to appropriate concentrations (Table S1) in 1% BSA (w/v)/ PBST overnight at 4 °C. After washing for 1 h in 1% BSA (w/v)/ PBST, oocytes were incubated with appropriate Alexa Fluor-conjugated secondary antibodies (diluted 1:1000 in 1% BSA (w/v)/ PBST) for 1 h at room temperature. All experiments included negative control groups in which the primary antibody was substituted with the appropriate concentration of either anti-mouse or anti-rabbit IgG (Cat # sc-2027; sc-2025; Santa Cruz Biotechnology, Dallas, TX, USA) in 1% BSA (w/v)/ PBST. Oocytes were counterstained with Hoechst 33258 (20 µg/ml) diluted in PBS/ PVP for 15 min at room temperature. Finally, oocytes were mounted on Menzel Glӓser microscope slides (Thermo Fisher Scientific) in Citifluor Glycerol Solution AF2 (Cat # AGR1321, Citifluor Ltd., London, UK). To ensure accurate fluorescence quantification, all oocytes used in a single experimental replicate were immunostained concurrently with all treatment groups simultaneously with equivalent antibody concentrations, volumes and incubation times. Oocytes were then mounted in 1 µl of Citifluor to minimize quenching of fluorescence signals during image capture using an Olympus FV1000 confocal microscope.

### Confocal imaging

All oocyte images were captured using high-resolution confocal microscopy on an FV1000 confocal microscope (Olympus, Shinjuku, Tokyo, Japan). Oocytes were imaged using a 60 × oil immersion lens with a z-resolution of 0.5 μm (CREST) or 1 μm (HSET and α-tubulin). Fluorochromes were imaged sequentially to avoid bleed-through. Oocytes from each treatment group were imaged with identical parameters on the same day to minimize fluorescence fading.

### Quantification of fluorescence intensity

For GV oocytes, images chosen for quantification were those captured through the mid-section of the oocyte; positioned to incorporate the center of the nucleolus and thus encompass the center of the nucleus as well as the cytoplasm. The entire area within the oocyte was used for immunocytochemical quantification. Fluorescence intensity for immunocytochemistry was measured using ImageJ (National Institutes of Health, Bethesda, MD, USA) as previously described [19]. The integrated fluorescence intensity of a mid-section (encompassing the DNA) of the whole oocyte, was determined and the background fluorescence was measured at four locations on the image and averaged. For determination of fluorescence intensity in captured images, the corrected total cell fluorescence (CTCF), or normalized fluorescence, was used as described in the following equation; CTCF = Integrated fluorescence intensity - (area of selected cell × average background fluorescence). This measurement considers differences in the size of oocytes via correction of the background staining intensity for the size of the cell. Data collected from individual experimental replicates were normalized to appropriate untreated controls.

### SDS-polyacrylamide gel electrophoresis (SDS-PAGE) and immunoblotting

To confirm the specificity of the anti-HSET antibodies, a standard immunoblotting procedure was performed as previously described with minor alternations [67]. Briefly, protein was extracted from isolated oocytes via direct incubation in SDS extraction buffer comprising 2% SDS (w/v), 10% sucrose (w/v) in 0.1875 M Tris, pH 6.8 and supplemented with ProteCEASE protease inhibitors (Cat # 786‐326, G-Biosciences, MO, USA) and boiling (100°C for 5 min). The resultant protein lysates were diluted into SDS-PAGE loading buffer containing 2% β-mercaptoethanol and bromophenol blue before being resolved on NuPage 10% Bis-Tris gels (Cat # NP0301BOX, Thermo Fisher Scientific) and transferred using an XCell Blot Module (Cat # EI9051, Thermo Fisher Scientific) onto nitrocellulose membranes (Cat # 10600002, GE Healthcare, Buckinghamshire, UK). Membranes were blocked by incubation in 3% BSA (w/v) / Tris-buffered saline (TBS; pH 7.4) and 0.1% Tween-20 (TBST) for 2 h at room temperature before being incubated with anti-HSET antibodies, diluted in 1% BSA (w/v) / TBST overnight at 4 °C. Membranes were washed three times with TBST and incubated with horseradish peroxidase-conjugated secondary antibody diluted into 1% BSA (w/v) / TBST for 1 h. Following three washes in TBST, labeled proteins were detected using an enhanced chemiluminescence kit (Cat # RPN2106, GE Healthcare) and visualized using ImageQuant LAS 4000 (Fujifilm, Tempe, AZ, USA).

### Statistical analysis

Statistical analysis was performed using two-tailed unpaired Student’s *t*-tests and one-way analysis of variances (ANOVA) with Tukey’s post-hoc multiple comparison using GraphPad Prism 7 software (San Diego, CA, USA). A p-value of < 0.05 was considered significant. RT-qPCR experiments were performed using three biological and three technical replicates. Each biological replicate comprised 10 oocytes randomly sampled from a pool of oocytes isolated from three animals. Immunofluorescence experiments were repeated on three individual biological replicates, with each replicate comprising a minimum of 10 oocytes randomly sampled from a pool of oocytes isolated from three animals. siRNA-mediated HSET knockdown and pharmacological inhibition experiments were repeated using three biological replicates, with each replicate comprising a minimum of 20 oocytes randomly sampled from a pool of oocytes isolated from three animals. Statistical analyses were performed using the mean of all biological replicates ± S.E.M. RNA-Seq was performed on a single biological replicate comprising of 582 young (4 -6 weeks) and 521 aged (14 – 16 months), which were pooled after isolation from 18 and 49 animals, respectively.

## SUPPLEMENTARY MATERIAL

Supplementary Figures

Supplementary Table 1

Supplementary Table 2

Supplementary Table 3

Supplementary Table 4

Supplementary Table 5

Supplementary Table 6
