# Supplementary material for: The small non-coding RNA profile of mouse oocytes is modified during aging
Source: Aging (Albany NY). 2019 May 24;11(10):2968–97. doi: 10.18632/aging.101947 (PMC6555462; doi:10.18632/aging.101947)
Supplement: Supplementary Figures [file aging-11-101947-s001.docx]

**Supplementary Table S1. Antibodies used for immunocytochemistry (ICC) and immunoblotting (IB).**

| Antibody | Species | Concentration | Dilution | Catalogue Number (Cat. #) and Source |
| --- | --- | --- | --- | --- |
| α-tubulin | Mouse monoclonal | 1 mg/ ml | 1:400 (ICC) | Cat. # A11126, Thermo Fisher Scientific |
|  |  |  |  |  |
| HSET | Rabbit monoclonal | 1 mg/ ml | 1:100 (ICC) 1:500 (IB) | Cat. # 20709-1-AP, United Bioreasearch |
| CREST | Human monoclonal | 1 mg/ ml | 1:400 (ICC) | Cat. # 90C-CS1058, Fitzgerald |
