# Supplementary material for: The small non-coding RNA profile of mouse oocytes is modified during aging
Source: Aging (Albany NY). 2019 May 24;11(10):2968–97. doi: 10.18632/aging.101947 (PMC6555462; doi:10.18632/aging.101947)
Supplement: Supplementary Table 1 [file aging-11-101947-s002.docx]

| Supplementary Table S6. Accession numbers (Accession No), read numbers (read No), log2 fold change (Log2 FC), false discovery rate (FDR), sequence, sequence size (BP), target site, and endo-siRNA-target orientation of the three Kifc1 and Kifc5b targeting endo-siRNAs. | | | | | | | | | | |
| --- | --- | --- | --- | --- | --- | --- | --- | --- | --- | --- |
| Accession No | Name | Young (Read No) | Aged (Read No) | Log2 FC | FDR | Sequence | BP | mRNA Target | Target Site | Target Orientation |
| URS00000682CA | RNA9878 | 2 | 21 | 3.88 | 0.0010188 | CGGGGCCCAGTAGCTAGCAG | 20 | Kifc1 | Coding Region | Plus/Minus |
|  |  |  |  |  |  |  |  | Kifc5b | Coding Region | Plus/Minus |
| URS0000131954 | RNA9867 | 0 | 10 | 6.66 | 0.0171119 | TGCAGCTGGTTGTGGAGTCGC | 21 | Kifc1 | Coding Region | Plus/Minus |
|  |  |  |  |  |  |  |  | Kifc5b | Coding Region | Plus/Minus |
| URS000033242E | RNA9879 | 20 | 43 | 1.68 | 0.0371872 | TTGCGGGGCCCAGTAGCTAGCAG | 23 | Kifc1 | Coding Region | Plus/Minus |
|  |  |  |  |  |  |  |  | Kifc5b | Coding Region | Plus/Minus |
