# Supplementary material for: The small non-coding RNA profile of mouse oocytes is modified during aging
Source: Aging (Albany NY). 2019 May 24;11(10):2968–97. doi: 10.18632/aging.101947 (PMC6555462; doi:10.18632/aging.101947)
Supplement: Supplementary Table 2 [file aging-11-101947-s003.docx]

| Supplementary Table S3. Accession numbers (Accession No) Read numbers (read No), log2 fold change (Log2 FC), and false discovery rate (FDR) of miRNAs identified from RNA-Seq. Counts of ≥ 10 reads were used as a threshold for positive miRNA identification. | | | | | | |
| --- | --- | --- | --- | --- | --- | --- |
| Type | Accession No | Name | Young (Read No) | Aged (Read No) | Log2 FC | FDR |
| miRNA | URS0000377E71 | mmu-miR-486a-3p | 21 | 204 | 3.852668783 | 3.36E-10 |
| miRNA | URS00004BF1DC | mmu-miR-486b-5p | 3004 | 9999 | 2.316954717 | 5.20E-06 |
| miRNA | URS00002F1F3B | mmu-miR-486b-3p | 5 | 37 | 3.430925907 | 8.43E-05 |
| miRNA | URS000055F35D | mmu-miR-143 | 524 | 89 | -1.974366909 | 0.00047948 |
| miRNA | URS00005C2A6D | mmu-miR-143-3p | 518 | 91 | -1.925731394 | 0.000723365 |
| miRNA | URS00003F2D94 | mmu-miR-199b-3p | 21 | 0 | -7.140683645 | 0.001989789 |
| miRNA | URS0000330617 | mmu-miR-27b-5p | 14 | 0 | -6.560824035 | 0.017111944 |
| miRNA | URS000040C1ED | mmu-miR-322-5p | 168 | 40 | -1.48595611 | 0.025027984 |
| miRNA | URS0000537D7C | mmu-miR-351-5p | 312 | 83 | -1.327203232 | 0.034052119 |
| miRNA | URS000018C928 | mmu-miR-181c-5p | 41 | 7 | -1.952860016 | 0.043943988 |
| miRNA | URS00005C1B5F | mmu-miR-132 | 11 | 0 | -6.217062441 | 0.054256875 |
| miRNA | URS00002529AE | mmu-miR-141 | 11 | 0 | -6.217062441 | 0.054256875 |
| miRNA | URS00002B277E | mmu-miR-674-3p | 10 | 0 | -6.081496947 | 0.079048176 |
| miRNA | URS00004B2A47 | mmu-miR-30d-3p | 10 | 0 | -6.081496947 | 0.079048176 |
| miRNA | URS000028C76E | mmu-miR-199-as | 10 | 0 | -6.081496947 | 0.079048176 |
| miRNA | URS000034798A | mmu-miR-5097 | 174 | 248 | 1.09270618 | 0.08352491 |
| miRNA | URS000032FACE | mmu-miR-151 | 221 | 69 | -1.096151132 | 0.099808628 |
| miRNA | URS00004449AE | mmu-miR-106b-5p | 15 | 1 | -3.201462396 | 0.099808628 |
| miRNA | URS000058760A | mmu-miR-10b-5p | 297 | 97 | -1.031532597 | 0.113565109 |
| miRNA | URS00003E3515 | mmu-miR-872-3p | 23 | 4 | -1.915154042 | 0.124082356 |
| miRNA | URS0000307DF8 | mmu-miR-664-5p | 14 | 1 | -3.102945857 | 0.130300569 |
| miRNA | URS000028D142 | mmu-let-7d-3p | 117 | 36 | -1.116166769 | 0.133528957 |
| miRNA | URS00000ED600 | mmu-miR-375-3p | 19 | 32 | 1.327356408 | 0.146525292 |
| miRNA | URS000039CE41 | mmu-miR-881-3p | 205 | 69 | -0.987804984 | 0.151703032 |
| miRNA | URS000014B746 | mmu-miR-203 | 7 | 15 | 1.660681888 | 0.151790022 |
| miRNA | URS0000209905 | mmu-miR-125b-5p | 53 | 15 | -1.233393355 | 0.177593158 |
| miRNA | URS0000237AF9 | mmu-miR-335-5p | 55 | 16 | -1.19417323 | 0.188336755 |
| miRNA | URS0000155642 | mmu-miR-192-5p | 74 | 23 | -1.100430886 | 0.192391654 |
| miRNA | URS0000190DC2 | mmu-let-7e | 10 | 1 | -2.62361877 | 0.216318601 |
| miRNA | URS00000BE495 | mmu-miR-423-3p | 106 | 36 | -0.973914035 | 0.219521704 |
| miRNA | URS00003BBF48 | mmu-miR-148a-3p | 735 | 281 | -0.804841106 | 0.223341019 |
| miRNA | URS0000237FB8 | mmu-miR-30c-1-3p | 94 | 32 | -0.970267408 | 0.232222871 |
| miRNA | URS000019B0F7 | mmu-miR-26a-5p | 774 | 302 | -0.775472914 | 0.239800262 |
| miRNA | URS00001E6362 | mmu-miR-L-28 | 12 | 19 | 1.234752597 | 0.239800262 |
| miRNA | URS00002DFEB9 | mmu-miR-295-3p | 281 | 108 | -0.796858087 | 0.258351613 |
| miRNA | URS00005FDE70 | mmu-miR-328-3p | 185 | 71 | -0.79865651 | 0.270936881 |
| miRNA | URS0000527228 | mmu-miR-30c-2-3p | 62 | 21 | -0.976387291 | 0.273094715 |
| miRNA | URS00001FFA8C | mmu-miR-381-3p | 16 | 3 | -1.799015053 | 0.279219595 |
| miRNA | URS0000096022 | mmu-miR-22-3p | 1310 | 534 | -0.71243651 | 0.283171821 |
| miRNA | URS000050CF2F | mmu-miR-1983 | 10 | 17 | 1.33465669 | 0.287755781 |
| miRNA | URS00004BF607 | mmu-miR-871-3p | 8070 | 3339 | -0.69102287 | 0.290850579 |
| miRNA | URS0000170CF4 | mmu-miR-221-3p | 30 | 9 | -1.146081844 | 0.290850579 |
| miRNA | URS000075C08B | mmu-miR-129b-3p. | 11 | 1 | -2.759184263 | 0.290850579 |
| miRNA | URS0000597BED | mmu-miR-484 | 221 | 87 | -0.762164635 | 0.291220907 |
| miRNA | URS000042B744 | mmu-let-7i | 36 | 11 | -1.12130259 | 0.291664225 |
| miRNA | URS000075A5B0 | mmu-miR-126b-3p | 22 | 5 | -1.536583054 | 0.297359358 |
| miRNA | URS0000361AEA | mmu-miR-30a | 373 | 151 | -0.722137189 | 0.299421714 |
| miRNA | URS000015EBFE | mmu-miR-741-3p | 184 | 72 | -0.770693686 | 0.299740674 |
| miRNA | URS00004C0C6B | mmu-miR-743b-3p | 159 | 62 | -0.775604909 | 0.301351875 |
| miRNA | URS00000C5BAA | mmu-miR-411-5p | 78 | 28 | -0.893554223 | 0.301535819 |
| miRNA | URS00002AABED | mmu-let-7d | 172 | 67 | -0.777164721 | 0.303648806 |
| miRNA | URS000040C192 | mmu-miR-871-5p | 901 | 374 | -0.686229409 | 0.309717564 |
| miRNA | URS00004DC6A5 | mmu-miR-30e-3p | 142 | 56 | -0.759223926 | 0.32018087 |
| miRNA | URS000000B1C9 | mmu-let-7e-5p | 61 | 22 | -0.886182349 | 0.326348218 |
| miRNA | URS0000543D82 | mmu-miR-184-3p | 40 | 47 | 0.812448208 | 0.345471017 |
| miRNA | URS0000192F9C | mmu-miR-200c-3p | 59 | 22 | -0.838207925 | 0.348612112 |
| miRNA | URS00001B9705 | mmu-miR-465a-5p | 94 | 36 | -0.800842458 | 0.349327625 |
| miRNA | URS00001DE669 | mmu-miR-30e-5p | 15 | 21 | 1.060057842 | 0.358222433 |
| miRNA | URS00005A4DCF | mmu-miR-125a-5p | 304 | 129 | -0.65417887 | 0.366497178 |
| miRNA | URS00002DABEA | mmu-miR-125b-1-3p | 18 | 5 | -1.249244202 | 0.368708004 |
| miRNA | URS0000521626 | mmu-miR-148b-3p | 55 | 20 | -0.87404053 | 0.369536088 |
| miRNA | URS00000565C2 | mmu-miR-322-3p | 49 | 18 | -0.859075547 | 0.380590484 |
| miRNA | URS0000181EB6 | mmu-miR-320 | 52 | 19 | -0.866969137 | 0.382269873 |
| miRNA | URS00005F9596 | mmu-miR-3073b-5p | 16 | 4 | -1.395672812 | 0.387946147 |
| miRNA | URS000035F92F | mmu-miR-191 | 7 | 11 | 1.216697095 | 0.387946147 |
| miRNA | URS00003D5044 | mmu-miR-182-5p | 12898 | 5731 | -0.588166888 | 0.387966173 |
| miRNA | URS000043D1A9 | mmu-miR-30a-5p | 211 | 90 | -0.646553282 | 0.396847807 |
| miRNA | URS000016B3AD | mmu-miR-182 | 2075 | 925 | -0.583420881 | 0.399524491 |
| miRNA | URS000008E301 | mmu-miR-125a | 172 | 73 | -0.653606291 | 0.403794302 |
| miRNA | URS0000476BE1 | mmu-miR-103-3p | 311 | 136 | -0.610828127 | 0.40692769 |
| miRNA | URS00002CC426 | mmu-miR-101 | 13 | 3 | -1.502541273 | 0.412434152 |
| miRNA | URS00001230A0 | mmu-miR-101a-3p | 13 | 3 | -1.502541273 | 0.412434152 |
| miRNA | URS00005AF7FD | mmu-miR-181c-3p | 13 | 3 | -1.502541273 | 0.412434152 |
| miRNA | URS00004AB1CD | mmu-miR-465b-3p | 279 | 122 | -0.610862096 | 0.417514205 |
| miRNA | URS0000236310 | mmu-miR-181d-5p | 72 | 29 | -0.727858749 | 0.430277392 |
| miRNA | URS000048BA36 | mmu-miR-425-5p | 17 | 5 | -1.167482681 | 0.430277392 |
| miRNA | URS00003E7283 | mmu-miR-365-3p | 11 | 2 | -1.826372474 | 0.43937236 |
| miRNA | URS0000528CBC | mmu-miR-183-5p | 360 | 163 | -0.560726352 | 0.450998149 |
| miRNA | URS00000E73CB | mmu-miR-26b | 238 | 107 | -0.570796507 | 0.454895864 |
| miRNA | URS00000E4947 | mmu-miR-470 | 219 | 98 | -0.577477867 | 0.456446868 |
| miRNA | URS00005916FD | mmu-miR-1839-5p | 14 | 4 | -1.204939204 | 0.459074573 |
| miRNA | URS00000386E7 | mmu-miR-872-5p | 72 | 30 | -0.679114611 | 0.462108386 |
| miRNA | URS000059311D | mmu-miR-27b-3p | 1916 | 893 | -0.519203348 | 0.467070817 |
| miRNA | URS000075C034 | mmu-miR-7068-3p. | 1432 | 672 | -0.509321176 | 0.483049545 |
| miRNA | URS000016D2D4 | mmu-miR-10a-5p | 54 | 56 | 0.633158885 | 0.487591968 |
| miRNA | URS00003B7674 | mmu-let-7f-5p | 1416 | 669 | -0.499566713 | 0.494732883 |
| miRNA | URS00002C7B2B | mmu-miR-34c-5p | 148 | 67 | -0.560557006 | 0.495336469 |
| miRNA | URS000057DF36 | mmu-miR-1247-5p | 39 | 15 | -0.79233482 | 0.495336469 |
| miRNA | URS00005165DA | mmu-miR-30b-5p | 127 | 57 | -0.572851225 | 0.495336469 |
| miRNA | URS00005092C2 | mmu-miR-335-3p | 104 | 46 | -0.593705586 | 0.507874589 |
| miRNA | URS00002C0FCB | mmu-miR-130b-3p | 28 | 10 | -0.896643379 | 0.512945353 |
| miRNA | URS00001E3DAA | mmu-miR-127-3p | 770 | 371 | -0.471217533 | 0.527284032 |
| miRNA | URS000075D9C3 | mmu-miR-3547-3p. | 21 | 7 | -0.99262132 | 0.527284032 |
| miRNA | URS0000506FB9 | mmu-miR-292a-5p | 9 | 12 | 0.985309464 | 0.534229207 |
| miRNA | URS0000065D58 | mmu-miR-30a-3p | 257 | 122 | -0.492431641 | 0.535536822 |
| miRNA | URS0000394886 | mmu-miR-183 | 921 | 450 | -0.451076727 | 0.54479998 |
| miRNA | URS000039ED8D | mmu-miR-21a-5p | 130 | 122 | 0.490003849 | 0.546961185 |
| miRNA | URS0000329B12 | mmu-miR-92a-3p | 1925 | 946 | -0.442792758 | 0.547406958 |
| miRNA | URS0000520A95 | mmu-miR-151-3p | 40 | 17 | -0.649276158 | 0.560354975 |
| miRNA | URS00000FC90D | mmu-miR-204 | 74 | 34 | -0.538554387 | 0.566943601 |
| miRNA | URS000040D674 | mmu-miR-100-5p | 14 | 5 | -0.890074043 | 0.566943601 |
| miRNA | URS00003D1AE3 | mmu-miR-15a-5p | 28 | 11 | -0.760440521 | 0.571042657 |
| miRNA | URS00002075FA | mmu-miR-17-5p | 18 | 19 | 0.65573471 | 0.571042657 |
| miRNA | URS000003E1A9 | mmu-miR-141-3p | 477 | 237 | -0.426832884 | 0.573480641 |
| miRNA | URS00001F4670 | mmu-miR-296-3p | 11 | 3 | -1.264519851 | 0.574993816 |
| miRNA | URS00004AFF8D | mmu-let-7g-5p | 1066 | 535 | -0.412416269 | 0.577324218 |
| miRNA | URS00004BCD9C | mmu-miR-16-5p | 768 | 389 | -0.399133171 | 0.59962218 |
| miRNA | URS00004023EA | mmu-let-7i-5p | 1371 | 700 | -0.387639879 | 0.606115357 |
| miRNA | URS0000561F50 | mmu-miR-298-5p | 2433 | 1250 | -0.378668303 | 0.615032149 |
| miRNA | URS00000A4E70 | mmu-miR-465c-5p | 569 | 290 | -0.390144494 | 0.615949951 |
| miRNA | URS00005AEC2E | mmu-miR-149 | 12 | 4 | -0.985091475 | 0.617282502 |
| miRNA | URS0000416056 | mmu-let-7a-5p | 791 | 406 | -0.380002341 | 0.619385747 |
| miRNA | URS0000316FA5 | mmu-miR-26b-5p | 63 | 30 | -0.486897612 | 0.624808199 |
| miRNA | URS00000DAC94 | mmu-miR-547-3p | 22 | 9 | -0.701228492 | 0.625254148 |
| miRNA | URS00004F9744 | mmu-miR-25-3p | 484 | 249 | -0.376614787 | 0.629772509 |
| miRNA | URS00005743AE | mmu-miR-107-3p | 38 | 17 | -0.575559004 | 0.63134244 |
| miRNA | URS00004C5B22 | mmu-let-7g | 251 | 128 | -0.389163537 | 0.639461075 |
| miRNA | URS00003CF9BA | mmu-miR-27b | 1088 | 567 | -0.358088677 | 0.644600941 |
| miRNA | URS00003841F3 | mmu-miRNA317 | 18 | 7 | -0.771931868 | 0.648917382 |
| miRNA | URS0000420D03 | mmu-miR-378a-3p | 13 | 5 | -0.784333871 | 0.648917382 |
| miRNA | URS000036225D | mmu-miR-16-1 | 3619 | 1911 | -0.339134249 | 0.656688287 |
| miRNA | URS000057311A | mmu-miR-471 | 12 | 13 | 0.69075087 | 0.6711294 |
| miRNA | URS0000149452 | mmu-miR-93-5p | 150 | 77 | -0.379485106 | 0.673556447 |
| miRNA | URS00003DA300 | mmu-miR-181a-5p | 175 | 150 | 0.359448351 | 0.674345534 |
| miRNA | URS00004AD914 | mmu-miR-15b-5p | 110 | 56 | -0.391278972 | 0.67507066 |
| miRNA | URS00000A07C1 | mmu-let-7d-5p | 530 | 282 | -0.328079771 | 0.682411878 |
| miRNA | URS000061D3E1 | mmu-miR-470-5p | 4407 | 2367 | -0.314611988 | 0.683595041 |
| miRNA | URS000050B527 | mmu-miR-146a-5p | 10 | 11 | 0.711225446 | 0.699594908 |
| miRNA | URS0000384021 | mmu-miR-106b-3p | 10 | 11 | 0.711225446 | 0.699594908 |
| miRNA | URS0000148B91 | mmu-miR-342-3p | 50 | 44 | 0.396649488 | 0.703253759 |
| miRNA | URS00004E57E7 | mmu-miR-23b | 15 | 15 | 0.577366613 | 0.703253759 |
| miRNA | URS00004E0808 | mmu-miR-98-5p | 140 | 74 | -0.337309523 | 0.707594634 |
| miRNA | URS000013D17D | mmu-miR-19b-3p | 11 | 4 | -0.861177609 | 0.707594634 |
| miRNA | URS00001C8A86 | mmu-miR-423-5p | 1307 | 721 | -0.276039843 | 0.733822007 |
| miRNA | URS00004505E4 | mmu-miR-421-3p | 25 | 12 | -0.473417176 | 0.742301327 |
| miRNA | URS00001C770D | mmu-miR-149-5p | 27 | 13 | -0.469252789 | 0.755142758 |
| miRNA | URS000050DE77 | mmu-let-7c-5p | 347 | 193 | -0.264096383 | 0.755142758 |
| miRNA | URS00005C2E31 | mmu-miR-191-5p | 209 | 115 | -0.279534615 | 0.755142758 |
| miRNA | URS0000324096 | mmu-let-7b-5p | 171 | 138 | 0.272567511 | 0.765467116 |
| miRNA | URS00002FCA7A | mmu-miR-292a-3p | 21 | 10 | -0.48416411 | 0.76691261 |
| miRNA | URS00001A755A | mmu-miR-1843b-5p | 15 | 6 | -0.730284082 | 0.776984055 |
| miRNA | URS000055128B | mmu-miR-210-3p | 14 | 6 | -0.631767544 | 0.776984055 |
| miRNA | URS000022074F | mmu-miR-300-3p | 10 | 10 | 0.575022588 | 0.776984055 |
| miRNA | URS00002D7227 | mmu-miR-291a-5p | 46 | 24 | -0.355173455 | 0.786498605 |
| miRNA | URS00003F97C5 | mmu-miR-1981-5p | 74 | 60 | 0.279036696 | 0.791074328 |
| miRNA | URS00002B452B | mmu-miR-200b | 16 | 7 | -0.603495318 | 0.795198638 |
| miRNA | URS00001C11BC | mmu-miR-301a-3p | 123 | 69 | -0.251541903 | 0.812781904 |
| miRNA | URS00004FB43D | mmu-miR-29a | 27 | 23 | 0.3490676 | 0.814425667 |
| miRNA | URS00001FE271 | mmu-miR-541-5p | 286 | 164 | -0.220081482 | 0.814974249 |
| miRNA | URS000044CC4C | mmu-miR-471-5p | 10 | 4 | -0.725612116 | 0.814974249 |
| miRNA | URS00003C6588 | mmu-miRNA206 | 111 | 63 | -0.234678543 | 0.822573164 |
| miRNA | URS00004E8341 | mmu-miR-532-5p | 27 | 14 | -0.363125364 | 0.822573164 |
| miRNA | URS000015CB3A | mmu-miR-186 | 46 | 25 | -0.29651952 | 0.828241404 |
| miRNA | URS000008BB79 | mmu-miR-423 | 11 | 5 | -0.546312449 | 0.834881262 |
| miRNA | URS00003BD810 | mmu-miR-130b-5p | 11 | 5 | -0.546312449 | 0.834881262 |
| miRNA | URS0000030638 | mmu-miR-465b-5p | 29 | 15 | -0.366816898 | 0.836840923 |
| miRNA | URS00002ED61F | mmu-miR-744-5p | 236 | 137 | -0.202359747 | 0.840741969 |
| miRNA | URS0000007170 | mmu-miR-676-3p | 200 | 117 | -0.191224979 | 0.848618114 |
| miRNA | URS000016FF9C | mmu-miR-96-5p | 22 | 11 | -0.414610652 | 0.853593923 |
| miRNA | URS00001390DD | mmu-miR-341-3p | 15 | 7 | -0.511278248 | 0.876597449 |
| miRNA | URS00001539CF | mmu-miR-434-5p | 14 | 7 | -0.41276171 | 0.876597449 |
| miRNA | URS000025A0B7 | mmu-miR-435 | 41 | 24 | -0.189734787 | 0.894015374 |
| miRNA | URS00002F4D78 | mmu-miR-29a-3p | 30 | 17 | -0.23603276 | 0.901902999 |
| miRNA | URS00001F0427 | mmu-miR-15a | 119 | 71 | -0.1627404 | 0.908556806 |
| miRNA | URS000032555E | mmu-miR-501-3p | 46 | 27 | -0.185914535 | 0.909357918 |
| miRNA | URS000040DCFF | mmu-miR-186-5p | 28 | 22 | 0.233038505 | 0.912519555 |
| miRNA | URS0000007FBA | mmu-miR-340-5p | 65 | 38 | -0.191853822 | 0.91904569 |
| miRNA | URS000025576D | mmu-miR-92b-3p | 71 | 42 | -0.174923812 | 0.929382611 |
| miRNA | URS00000D812E | mmu-miR-672-5p | 386 | 239 | -0.109432837 | 0.940449511 |
| miRNA | URS0000326EC7 | mmu-miR-3471 | 41 | 30 | 0.130994139 | 0.943899746 |
| miRNA | URS00003CF1AD | mmu-miR-320-3p | 164 | 101 | -0.117115773 | 0.946281813 |
| miRNA | URS0000021B51 | mmu-miR-155-5p | 43 | 32 | 0.155335274 | 0.946281813 |
| miRNA | URS00004DFCB1 | mmu-miR-miR-130 | 11 | 6 | -0.28800595 | 0.946281813 |
| miRNA | URS000019C8F1 | mmu-miR-434-3p | 131 | 81 | -0.111327329 | 0.946508306 |
| miRNA | URS00000DE5AF | mmu-miR-31-5p | 56 | 34 | -0.137392977 | 0.95999881 |
| miRNA | URS000034CD58 | mmu-miR-293-5p | 13 | 7 | -0.307021537 | 0.95999881 |
| miRNA | URS000043908D | mmu-miR-431-5p | 15 | 8 | -0.321178338 | 0.964206536 |
| miRNA | URS00000887D8 | mmu-miR-miR-27a | 15 | 8 | -0.321178338 | 0.964206536 |
| miRNA | URS000029D9F1 | mmu-miR-204-5p | 14 | 11 | 0.231898359 | 0.965461396 |
| miRNA | URS00000FB635 | mmu-miR-3535 | 19 | 14 | 0.140485073 | 0.977897443 |
| miRNA | URS000019907A | mmu-miR-30c-5p | 53 | 33 | -0.101116845 | 0.979267202 |
| miRNA | URS000061F139 | mmu-miR-246 | 23 | 14 | -0.133184271 | 0.98374376 |
| miRNA | URS00002C10B3 | mmu-miR-99b-5p | 176 | 113 | -0.057089816 | 0.989949364 |
| miRNA | URS000006B2AB | mmu-miR-060 | 172 | 119 | 0.050622908 | 1 |
| miRNA | URS0000013DD8 | mmu-miR-652-3p | 68 | 44 | -0.045815368 | 1 |
| miRNA | URS00000CF1D2 | mmu-miR-361-5p | 67 | 47 | 0.070459083 | 1 |
| miRNA | URS000075A908 | mmu-miR-7035-3p. | 14 | 8 | -0.2226618 | 1 |
| miRNA | URS000042EA31 | mmu-miR-155 | 15 | 9 | -0.15323602 | 1 |
| miRNA | URS00004996E9 | mmu-miR-873a-5p | 10 | 6 | -0.152440456 | 1 |
| miRNA | URS000025440F | mmu-miR-1843a-5p | 15 | 11 | 0.13338182 | 1 |
| miRNA | URS0000315338 | mmu-miR-130a-3p | 18 | 11 | -0.1272718 | 1 |
| miRNA | URS000061CA23 | mmu-miR-128 | 13 | 8 | -0.116921627 | 1 |
| miRNA | URS00000821E0 | mmu-miR-140-3p | 17 | 12 | 0.078935819 | 1 |
| miRNA | URS0000605E00 | mmu-miR-181b-5p | 20 | 14 | 0.067049183 | 1 |
| miRNA | URS00005F8E5B | mmu-miR-151-5p | 25 | 16 | -0.061367046 | 1 |
| miRNA | URS000005CF5F | mmu-miR-30d-5p | 68 | 47 | 0.049132789 | 1 |
| miRNA | URS0000298002 | mmu-miR-296 | 20 | 13 | -0.039078242 | 1 |
| miRNA | URS000075EEA3 | mmu-miR-6944-3p. | 64 | 43 | 0.008358483 | 1 |
| miRNA | URS00004C43E8 | mmu-miR-34b-3p | 18 | 12 | -0.002825702 | 1 |
| miRNA | URS0000584A54 | mmu-miR-877-3p | 15 | 10 | -0.002821038 | 1 |
| miRNA | URS000024A59E | mmu-miR-128-3p | 15 | 10 | -0.002821038 | 1 |
| miRNA | URS000025E0BB | mmu-miR-98-3p | 12 | 8 | -0.002814071 | 1 |
