# Supplementary material for: The small non-coding RNA profile of mouse oocytes is modified during aging
Source: Aging (Albany NY). 2019 May 24;11(10):2968–97. doi: 10.18632/aging.101947 (PMC6555462; doi:10.18632/aging.101947)
Supplement: Supplementary Table 3 [file aging-11-101947-s004.docx]

| Supplementary Table S4. mRNA target prediction of differentially expressed endo-siRNA between young and aged oocytes using NCBI’s nucleotide blast sequence alignment. Table includes target mRNA gene symbol, gene description, maximum score, total score, query coverage, E value, identity, target, and target orientation. | | | | | | | | | |
| --- | --- | --- | --- | --- | --- | --- | --- | --- | --- |
| endo-siRNA Name | Gene Symbol | Gene Description | Maximum score | Total score | Query coverage | E value | Identity | Target | Orientation |
| RNA9677 | *Rangap1* | Mus musculus RAN GTPase activating protein 1 (Rangap1), transcript variant 1, mRNA | 32.2 | 32.2 | 100% | 1.9 | 95% | Coding Region | Plus/Minus |
| RNA14343 | *Kif4* | Mus musculus kinesin family member 4 (Kif4), mRNA | 40.1 | 40.1 | 100% | 0.008 | 100% | 5' UTR | Plus/Minus |
| RNA9672 | *Rangap1* | Mus musculus RAN GTPase activating protein 1 (Rangap1), transcript variant 1, mRNA | 36.2 | 36.2 | 100% | 0.2 | 95% | Coding Region | Plus/Minus |
| RNA14342 | *Kif4* | Mus musculus kinesin family member 4 (Kif4), mRNA | 42.1 | 68.4 | 100% | 0.003 | 100% | 5' UTR | Plus/Minus |
| RNA12422 | *Acnat1* | Mus musculus acyl-coenzyme A amino acid N-acyltransferase 1 (Acnat1), mRNA | 44.1 | 44.1 | 100% | 8.00E-04 | 100% | 3' URT | Plus/Minus |
| RNA9674 | *Rangap1* | Mus musculus RAN GTPase activating protein 1 (Rangap1), transcript variant 1, mRNA | 32.2 | 32.2 | 100% | 1.9 | 95% | Coding Region | Plus/Minus |
| RNA14300 | *Kif4* | Mus musculus kinesin family member 4 (Kif4), mRNA | 42.1 | 42.1 | 100% | 0.003 | 100% | 5' UTR | Plus/Minus |
| RNA14348 | *Kif4* | Mus musculus kinesin family member 4 (Kif4), mRNA | 42.1 | 42.1 | 100% | 0.003 | 100% | 5' UTR | Plus/Minus |
| RNA14304 | *Kif4* | Mus musculus kinesin family member 4 (Kif4), mRNA | 42.1 | 42.1 | 100% | 0.003 | 100% | 5' UTR | Plus/Minus |
| RNA11666 | *Rest* | Mus musculus RE1-silencing transcription factor (Rest), mRNA | 42.1 | 42.1 | 100% | 0.003 | 100% | Coding Region | Plus/Minus |
| RNA10399 | *Prc1* | Mus musculus protein regulator of cytokinesis 1 (Prc1), transcript variant 3, mRNA | 40.1 | 40.1 | 100% | 0.008 | 100% | Coding Region | Plus/Minus |
| RNA13419 | *Dcun1d5* | Mus musculus DCN1, defective in cullin neddylation 1, domain containing 5 (S. cerevisiae) (Dcun1d5), mRNA | 42.1 | 66.4 | 100% | 0.003 | 100% | 5' UTR | Plus/Minus |
| RNA10679 | *Zcchc3* | Mus musculus zinc finger, CCHC domain containing 3 (Zcchc3), mRNA | 40.1 | 40.1 | 100% | 0.008 | 100% | Coding Region | Plus/Minus |
| RNA9878 | *Kifc1* | Mus musculus kinesin family member C1 (Kifc1), mRNA | 40.1 | 40.1 | 100% | 0.008 | 100% | Coding Region | Plus/Minus |
|  | *Kifc5b* | Mus musculus kinesin family member C5B (Kifc5b), mRNA | 40.1 | 40.1 | 100% | 0.008 | 100% | Coding Region | Plus/Minus |
| RNA12469 | *Tubb4b* | Mus musculus tubulin, beta 4B class IVB (Tubb4b), mRNA | 42.1 | 42.1 | 100% | 0.003 | 100% | Coding Region | Plus/Minus |
|  | *Tubb2a* | Mus musculus tubulin, beta 2A class IIA (Tubb2a), mRNA | 42.1 | 42.1 | 100% | 0.003 | 100% | Coding Region | Plus/Minus |
|  | *Tubb2b* | Mus musculus tubulin, beta 2B class IIB (Tubb2b), mRNA | 42.1 | 42.1 | 100% | 0.003 | 100% | Coding Region | Plus/Minus |
|  | *Tubb3* | Mus musculus tubulin, beta 3 class III (Tubb3), mRNA | 42.1 | 42.1 | 100% | 0.003 | 100% | Coding Region | Plus/Minus |
|  | *Tubb6* | Mus musculus tubulin, beta 6 class V (Tubb6), mRNA | 34.2 | 34.2 | 100% | 0.64 | 95% | Coding Region | Plus/Minus |
| RNA6661 | *Hmmr* | Mus musculus hyaluronan mediated motility receptor (RHAMM) (Hmmr), mRNA | 46.1 | 46.1 | 100% | 7.00E-03 | 100% | 5' UTR | Plus/Minus |
| RNA6611 | *Lgalsl* | Mus musculus lectin, galactoside binding-like (Lgalsl), mRNA | 42.1 | 42.1 | 100% | 0.003 | 100% | 3' UTR | Plus/Minus |
| RNA8503 | *Kcnk13* | Mus musculus potassium channel, subfamily K, member 13 (Kcnk13), transcript variant 1, mRNA | 42.1 | 42.1 | 100% | 0.003 | 100% | Coding Region | Plus/Minus |
| RNA4292 | *F420015M19Rik* | PREDICTED: Mus musculus RIKEN cDNA F420015M19 gene (F420015M19Rik), mRNA | 38.2 | 38.2 | 95% | 0.031 | 100% | 3' UTR | Plus/Minus |
| RNA10696 | *Zcchc3* | Mus musculus zinc finger, CCHC domain containing 3 (Zcchc3), mRNA | 42.1 | 42.1 | 100% | 0.003 | 100% | Coding Region | Plus/Minus |
| RNA10927 | *Gpr149* | Mus musculus G protein-coupled receptor 149 (Gpr149), mRNA | 44.1 | 44.1 | 100% | 8.00E-04 | 100% | Coding Region | Plus/Minus |
| RNA9673 | *Rangap1* | Mus musculus RAN GTPase activating protein 1 (Rangap1), transcript variant 1, mRNA | 34.2 | 34.2 | 100% | 0.64 | 95% | Coding Region | Plus/Minus |
| RNA10415 | *Prc1* | Mus musculus protein regulator of cytokinesis 1 (Prc1), transcript variant 1, mRNA | 34.2 | 34.2 | 100% | 0.64 | 95% | 5' UTR | Plus/Minus |
| RNA13058 | *Gm5148* | [Mus musculus predicted gene 5148 (Gm5148), mRNA](https://blast.ncbi.nlm.nih.gov/Blast.cgi#alnHdr_142348074) | 44.1 | 44.1 | 100% | 8.00E-04 | 100% | Coding Region | Plus/Minus |
|  | *C330021F23Rik* | Mus musculus RIKEN cDNA C330021F23 gene (C330021F23Rik), mRNA | 44.1 | 44.1 | 100% | 8.00E-04 | 100% | Coding Region | Plus/Minus |
| RNA14370 | *Kif4* | Mus musculus kinesin family member 4 (Kif4), mRNA | 38.2 | 38.2 | 100% | 0.021 | 100% | Coding Region | Plus/Minus |
| RNA9867 | *Kifc5b* | Mus musculus kinesin family member C5B (Kifc5b), mRNA | 42.1 | 42.1 | 100% | 0.003 | 100% | Coding Region | Plus/Minus |
|  | *Kifc1* | Mus musculus kinesin family member C1 (Kifc1), mRNA | 40.1 | 40.1 | 95% | 0.01 | 100% | Coding Region | Plus/Minus |
| RNA6556 | *Ube2l3* | [Mus musculus ubiquitin-conjugating enzyme E2L 3 (Ube2l3), mRNA](https://blast.ncbi.nlm.nih.gov/Blast.cgi#alnHdr_133892277) | 34.2 | 34.2 | 100% | 0.64 | 95% | Coding Region | Plus/Minus |
|  | *LOC100861784* | PREDICTED: Mus musculus ubiquitin-conjugating enzyme E2 L3-like (LOC100861784), mRNA | 34.2 | 34.2 | 100% | 0.64 | 95% | Coding Region | Plus/Minus |
|  | *Gm10705* | PREDICTED: Mus musculus predicted gene 10705 (Gm10705), mRNA | 34.2 | 34.2 | 100% | 0.64 | 95% | Coding Region | Plus/Minus |
| RNA11963 | *LOC100041057* | [PREDICTED: Mus musculus sp110 nuclear body protein-like (LOC100041057), transcript variant X11, mRNA](https://blast.ncbi.nlm.nih.gov/Blast.cgi#alnHdr_1039727079) | 42.1 | 42.1 | 100% | 0.003 | 100% | 3' UTR | Plus/Minus |
|  | *C130026I21Rik* | PREDICTED: Mus musculus RIKEN cDNA C130026I21 gene (C130026I21Rik), transcript variant X2, mRNA | 42.1 | 42.1 | 100% | 0.003 | 100% | Coding Region | Plus/Minus |
|  | *Sp110* | PREDICTED: Mus musculus Sp110 nuclear body protein (Sp110), transcript variant X6, mRNA | 42.1 | 42.1 | 100% | 0.003 | 100% | 3' UTR | Plus/Minus |
|  | *LOC102638047* | PREDICTED: Mus musculus sp110 nuclear body protein-like (LOC102638047), transcript variant X5, mRNA | 42.1 | 42.1 | 100% | 0.003 | 100% | 3' UTR | Plus/Minus |
|  | *LOC677525* | PREDICTED: Mus musculus sp110 nuclear body protein-like (LOC677525), mRNA | 34.2 | 34.2 | 100% | 0.64 | 95% | 3' UTR | Plus/Minus |
| RNA9630 | *Rangap1* | [Mus musculus RAN GTPase activating protein 1 (Rangap1), transcript variant 1, mRNA](https://blast.ncbi.nlm.nih.gov/Blast.cgi#alnHdr_226062634) | 34.2 | 34.2 | 100% | 0.64 | 95% | Coding Region | Plus/Minus |
| RNA10681 | *Zcchc3* | [Mus musculus zinc finger, CCHC domain containing 3 (Zcchc3), mRNA](https://blast.ncbi.nlm.nih.gov/Blast.cgi#alnHdr_227500549) | 42.1 | 42.1 | 100% | 0.003 | 100% | Coding Region | Plus/Minus |
| RNA11793 | *Myo1h* | [Mus musculus myosin 1H (Myo1h), mRNA](https://blast.ncbi.nlm.nih.gov/Blast.cgi#alnHdr_257196126) | 52 | 52 | 100% | 5.00E-06 | 100% | 3' UTR | Plus/Minus |
| RNA12022 | *LOC677525* | PREDICTED: Mus musculus sp110 nuclear body protein-like (LOC677525), mRNA | 42.1 | 42.1 | 100% | 0.003 | 100% | Coding Region | Plus/Minus |
|  | *LOC546061* | [Mus musculus sp110 nuclear body protein-like (LOC546061), mRNA](https://blast.ncbi.nlm.nih.gov/Blast.cgi#alnHdr_1039727058) | 42.1 | 42.1 | 100% | 0.003 | 100% | Coding Region | Plus/Minus |
|  | *Sp110* | [Mus musculus Sp110 nuclear body protein (Sp110), transcript variant 1, mRNA](https://blast.ncbi.nlm.nih.gov/Blast.cgi#alnHdr_133893021) | 34.2 | 34.2 | 100% | 0.64 | 95% | 5' UTR | Plus/Minus |
| RNA13574 | *Snx22* | [Mus musculus sorting nexin 22 (Snx22), mRNA](https://blast.ncbi.nlm.nih.gov/Blast.cgi#alnHdr_118131181) | 50.1 | 50.1 | 100% | 2.00E-05 | 100% | 3' UTR | Plus/Minus |
| RNA4172 | *F420015M19Rik* | PREDICTED: Mus musculus RIKEN cDNA F420015M19 gene (F420015M19Rik), mRNA | 48.1 | 48.1 | 100% | 7.00E-05 | 100% | 3' UTR | Plus/Minus |
| RNA3355 | *Wdr31* | [Mus musculus WD repeat domain 31 (Wdr31), transcript variant X2, mRNA](https://blast.ncbi.nlm.nih.gov/Blast.cgi#alnHdr_1039768672) | 36.2 | 36.2 | 100% | 0.2 | 95% | 3' UTR | Plus/Minus |
|  | *Gm14149* | [Mus musculus predicted gene 14149 (Gm14149), mRNA](https://blast.ncbi.nlm.nih.gov/Blast.cgi#alnHdr_1039763463) | 36.2 | 36.2 | 100% | 0.2 | 95% | 5' UTR | Plus/Minus |
|  | *Gramd1c* | [Mus musculus GRAM domain containing 1C (Gramd1c), transcript variant X4, mRNA](https://blast.ncbi.nlm.nih.gov/Blast.cgi#alnHdr_755531145) | 36.2 | 36.2 | 100% | 0.2 | 95% | 5' UTR | Plus/Minus |
| RNA6569 | *Ube2l3* | [Mus musculus ubiquitin-conjugating enzyme E2L 3 (Ube2l3), mRNA](https://blast.ncbi.nlm.nih.gov/Blast.cgi#alnHdr_133892277) | 36.2 | 36.2 | 100% | 0.2 | 95% | 3' UTR | Plus/Minus |
|  | *Gm10705* | PREDICTED: Mus musculus predicted gene 10705 (Gm10705), mRNA | 36.2 | 36.2 | 100% | 0.2 | 95% | 3' UTR | Plus/Minus |
| RNA5204 | *Esp24* | [Mus musculus exocrine gland secreted peptide 24 (Esp24), mRNA](https://blast.ncbi.nlm.nih.gov/Blast.cgi#alnHdr_365812497) | 44.1 | 44.1 | 100% | 0.001 | 96% | 5' UTR | Plus/Minus |
|  | *Kif2a* | [Mus musculus kinesin family member 2A (Kif2a), transcript variant 1, mRNA](https://blast.ncbi.nlm.nih.gov/Blast.cgi#alnHdr_224809370) | 44.1 | 44.1 | 100% | 0.001 | 96% | 5' UTR | Plus/Minus |
| RNA5121 | *Cdc5l* | [Mus musculus cell division cycle 5-like (S. pombe) (Cdc5l), mRNA](https://blast.ncbi.nlm.nih.gov/Blast.cgi#alnHdr_133892345) | 42.1 | 42.1 | 100% | 0.003 | 100% | Coding Region | Plus/Minus |
| RNA12013 | *Sp110* | [PREDICTED: Mus musculus Sp110 nuclear body protein (Sp110), transcript variant X6, mRNA](https://blast.ncbi.nlm.nih.gov/Blast.cgi#alnHdr_1039727291) | 42.1 | 42.1 | 100% | 0.003 | 100% | Coding Region | Plus/Minus |
|  | *LOC100041057* | PREDICTED: Mus musculus sp110 nuclear body protein-like (LOC100041057), transcript variant X11, mRNA | 42.1 | 42.1 | 100% | 0.003 | 100% | 3' UTR | Plus/Minus |
|  | *LOC102638047* | PREDICTED: Mus musculus sp110 nuclear body protein-like (LOC102638047), transcript variant X5, mRNA | 34.2 | 34.2 | 100% | 0.64 | 95% | 3' UTR | Plus/Minus |
|  | *C130026I21Rik* | PREDICTED: Mus musculus RIKEN cDNA C130026I21 gene (C130026I21Rik), transcript variant X1, mRNA | 42.1 | 42.1 | 100% | 0.003 | 100% | Coding Region | Plus/Minus |
|  | *LOC677525* | [PREDICTED: Mus musculus sp110 nuclear body protein-like (LOC677525), mRNA](https://blast.ncbi.nlm.nih.gov/Blast.cgi#alnHdr_1039727082) | 40.1 | 40.1 | 95% | 0.01 | 100% | 3' UTR | Plus/Minus |
| RNA9879 | *Kifc1* | [Mus musculus kinesin family member C1 (Kifc1), mRNA](https://blast.ncbi.nlm.nih.gov/Blast.cgi#alnHdr_305678349) | 46.1 | 46.1 | 100% | 3.00E-04 | 100% | Coding Region | Plus/Minus |
|  | *Kifc5b* | [Mus musculus kinesin family member C5B (Kifc5b), mRNA](https://blast.ncbi.nlm.nih.gov/Blast.cgi#alnHdr_116642892) | 46.1 | 46.1 | 100% | 3.00E-04 | 100% | Coding Region | Plus/Minus |
| RNA14340 | *Kif4* | [Mus musculus kinesin family member 4 (Kif4), mRNA](https://blast.ncbi.nlm.nih.gov/Blast.cgi#alnHdr_162287088) | 40.1 | 40.1 | 100% | 0.008 | 100% | 5' UTR | Plus/Minus |
| RNA13073 | *Rps23* | [Mus musculus ribosomal protein S23 (Rps23), mRNA](https://blast.ncbi.nlm.nih.gov/Blast.cgi#alnHdr_160333335) | 40.1 | 40.1 | 100% | 0.008 | 100% | Coding Region | Plus/Minus |
