# Supplementary material for: The small non-coding RNA profile of mouse oocytes is modified during aging
Source: Aging (Albany NY). 2019 May 24;11(10):2968–97. doi: 10.18632/aging.101947 (PMC6555462; doi:10.18632/aging.101947)
Supplement: Supplementary Table 4 [file aging-11-101947-s005.docx]

| Supplementary Table S2. Accession numbers (Accession No), read numbers (read No), log2 fold change (Log2 FC), and false discovery rate (FDR) of endo-siRNAs identified from RNA-Seq. Counts of ≥ 10 reads were used as a threshold for positive endo-siRNA identification. | | | | | | |
| --- | --- | --- | --- | --- | --- | --- |
| Type | Accession No | Name | Young (Read No) | Aged (Read No) | Log2 FC | FDR |
| endo-siRNA | URS00005F868B | RNA9677 | 8 | 226 | 5.376152106 | 1.85E-15 |
| endo-siRNA | URS0000260742 | RNA9705 | 13 | 134 | 3.932302057 | 2.78E-09 |
| endo-siRNA | URS0000053DFB | RNA9680 | 155 | 881 | 3.087756612 | 3.70E-09 |
| endo-siRNA | URS00002D071A | RNA14343 | 27 | 203 | 3.485286523 | 4.20E-09 |
| endo-siRNA | URS000007EC44 | RNA9715 | 23 | 180 | 3.541835665 | 5.23E-09 |
| endo-siRNA | URS000057C334 | RNA12315 | 0 | 51 | 8.99547395 | 7.61E-09 |
| endo-siRNA | URS00003AC5DF | RNA9672 | 39 | 232 | 3.149780661 | 3.36E-08 |
| endo-siRNA | URS0000032CB1 | RNA8522 | 2 | 45 | 4.972915438 | 3.98E-07 |
| endo-siRNA | URS00005C5507 | RNA10703 | 2 | 43 | 4.90747605 | 6.35E-07 |
| endo-siRNA | URS000034CAC8 | RNA14342 | 66 | 293 | 2.729696321 | 7.83E-07 |
| endo-siRNA | URS000014BEAE | RNA9716 | 4 | 47 | 4.086701898 | 1.13E-06 |
| endo-siRNA | URS00001C8F84 | RNA955 | 5 | 51 | 3.892828749 | 1.68E-06 |
| endo-siRNA | URS00000002B4 | RNA12422 | 16 | 94 | 3.124786209 | 2.01E-06 |
| endo-siRNA | URS00005839CB | RNA14040 | 133 | 10 | -3.13849377 | 5.67E-06 |
| endo-siRNA | URS00000F2B8E | RNA9674 | 12 | 72 | 3.151172637 | 8.87E-06 |
| endo-siRNA | URS00002785DF | RNA9518 | 2 | 34 | 4.569560967 | 1.15E-05 |
| endo-siRNA | URS0000216E38 | RNA14300 | 5 | 41 | 3.578644666 | 2.38E-05 |
| endo-siRNA | URS00000D062E | RNA9517 | 2 | 31 | 4.436704292 | 2.64E-05 |
| endo-siRNA | URS00005E4C8A | RNA14348 | 0 | 23 | 7.850047566 | 8.25E-05 |
| endo-siRNA | URS00000D1EDE | RNA1951 | 3 | 32 | 3.93132242 | 0.000104453 |
| endo-siRNA | URS000039119E | RNA14304 | 21 | 83 | 2.556314202 | 0.000114971 |
| endo-siRNA | URS00002F85A7 | RNA11666 | 1 | 21 | 4.779807277 | 0.000168772 |
| endo-siRNA | URS00001FE5A9 | RNA14164 | 36 | 0 | -7.914024983 | 0.00023024 |
| endo-siRNA | URS000059E7A0 | RNA10399 | 28 | 0 | -7.553162914 | 0.000234996 |
| endo-siRNA | URS00005C5F71 | RNA13944 | 3 | 28 | 3.739320012 | 0.000423221 |
| endo-siRNA | URS0000048C12 | RNA4147 | 158 | 21 | -2.32385622 | 0.000451605 |
| endo-siRNA | URS0000079E2A | RNA9150 | 0 | 18 | 7.498146457 | 0.000680861 |
| endo-siRNA | URS000028EC49 | RNA11525 | 76 | 7 | -2.840813532 | 0.000696533 |
| endo-siRNA | URS000045DAD2 | RNA9679 | 327 | 780 | 1.835820791 | 0.000757115 |
| endo-siRNA | URS0000290A55 | RNA9520 | 9 | 43 | 2.817980122 | 0.000769131 |
| endo-siRNA | URS0000491F8B | RNA13419 | 0 | 17 | 7.416153637 | 0.000955389 |
| endo-siRNA | URS00005FE21D | RNA138 | 24 | 0 | -7.332050175 | 0.000955389 |
| endo-siRNA | URS0000460BAD | RNA10679 | 5 | 29 | 3.08052714 | 0.000976847 |
| endo-siRNA | URS000009FDAE | RNA9787 | 131 | 319 | 1.864905887 | 0.000982696 |
| endo-siRNA | URS000019A8C3 | RNA11095 | 46 | 3 | -3.313818419 | 0.000985268 |
| endo-siRNA | URS00000682CA | RNA9878 | 2 | 21 | 3.877035316 | 0.001018791 |
| endo-siRNA | URS0000304F29 | RNA13781 | 2 | 21 | 3.877035316 | 0.001018791 |
| endo-siRNA | URS0000579F73 | RNA12179 | 1 | 16 | 4.389627483 | 0.001351886 |
| endo-siRNA | URS00001E8FD9 | RNA14032 | 1119 | 221 | -1.757518311 | 0.001460196 |
| endo-siRNA | URS0000228C8F | RNA9717 | 3 | 24 | 3.517784038 | 0.001583708 |
| endo-siRNA | URS00004E7A19 | RNA12469 | 4 | 27 | 3.289270369 | 0.001592618 |
| endo-siRNA | URS000050176E | RNA9906 | 59 | 6 | -2.695352853 | 0.00212765 |
| endo-siRNA | URS0000082A9E | RNA1257 | 9 | 38 | 2.640083771 | 0.002370023 |
| endo-siRNA | URS00001E3C38 | RNA13860 | 10 | 38 | 2.490445851 | 0.002370023 |
| endo-siRNA | URS00003E9B07 | RNA7012 | 90 | 12 | -2.31519117 | 0.002374711 |
| endo-siRNA | URS00000B3EC9 | RNA14380 | 0 | 15 | 7.236707186 | 0.002921264 |
| endo-siRNA | URS00002F6628 | RNA9385 | 12 | 42 | 2.374995301 | 0.003303153 |
| endo-siRNA | URS00005E9AC2 | RNA5033 | 35 | 2 | -3.482863924 | 0.003620857 |
| endo-siRNA | URS00005B8C2C | RNA6661 | 16 | 49 | 2.186316176 | 0.003686969 |
| endo-siRNA | URS00000F073D | RNA14178 | 30 | 1 | -4.194308219 | 0.003756156 |
| endo-siRNA | URS0000112B33 | RNA6611 | 30 | 1 | -4.194308219 | 0.003756156 |
| endo-siRNA | URS0000437EB1 | RNA766 | 3 | 21 | 3.325994899 | 0.003756156 |
| endo-siRNA | URS00000B256B | RNA8503 | 0 | 14 | 7.137854602 | 0.004127039 |
| endo-siRNA | URS00001910FC | RNA3634 | 19 | 0 | -6.997369529 | 0.004127039 |
| endo-siRNA | URS00004FBF62 | RNA4292 | 19 | 0 | -6.997369529 | 0.004127039 |
| endo-siRNA | URS0000238E68 | RNA13739 | 4 | 24 | 3.120011449 | 0.004526316 |
| endo-siRNA | URS000035BFA5 | RNA4249 | 14 | 45 | 2.254454502 | 0.004718354 |
| endo-siRNA | URS0000259B2A | RNA14108 | 29 | 1 | -4.145645906 | 0.004952621 |
| endo-siRNA | URS00001E4EC9 | RNA4817 | 116 | 20 | -1.948598522 | 0.004960226 |
| endo-siRNA | URS0000286487 | RNA14006 | 6 | 26 | 2.667539092 | 0.00511459 |
| endo-siRNA | URS00005A6413 | RNA14271 | 11 | 37 | 2.316508668 | 0.005218236 |
| endo-siRNA | URS00002C67CC | RNA13554 | 0 | 13 | 7.031727177 | 0.005961877 |
| endo-siRNA | URS0000057AEF | RNA11703 | 18 | 0 | -6.919994193 | 0.005961877 |
| endo-siRNA | URS00005794E4 | RNA6682 | 48 | 5 | -2.656816321 | 0.005961877 |
| endo-siRNA | URS00005164A7 | RNA2456 | 62 | 8 | -2.357623471 | 0.006285124 |
| endo-siRNA | URS0000267D29 | RNA10696 | 15 | 46 | 2.187577199 | 0.006285124 |
| endo-siRNA | URS000000D23B | RNA9496 | 108 | 219 | 1.600671427 | 0.007014669 |
| endo-siRNA | URS00002B7D46 | RNA10468 | 100 | 17 | -1.967969238 | 0.007322085 |
| endo-siRNA | URS00003183D4 | RNA838 | 93 | 16 | -1.950369378 | 0.007460566 |
| endo-siRNA | URS0000315592 | RNA9030 | 90 | 15 | -1.995652475 | 0.007580889 |
| endo-siRNA | URS000015429F | RNA10927 | 35 | 3 | -2.921011301 | 0.007992789 |
| endo-siRNA | URS0000112E5B | RNA1138 | 64 | 9 | -2.235376113 | 0.008409812 |
| endo-siRNA | URS00005F4357 | RNA9682 | 84 | 172 | 1.614328193 | 0.008409812 |
| endo-siRNA | URS00005DDDFD | RNA4238 | 84 | 14 | -1.995140704 | 0.008409812 |
| endo-siRNA | URS00001CC9AA | RNA13467 | 16 | 0 | -6.751557643 | 0.008409812 |
| endo-siRNA | URS00001E3C20 | RNA11559 | 16 | 0 | -6.751557643 | 0.008409812 |
| endo-siRNA | URS00000A4881 | RNA6198 | 1 | 12 | 3.977577353 | 0.008409812 |
| endo-siRNA | URS00002D036D | RNA9673 | 10 | 34 | 2.330427119 | 0.008409812 |
| endo-siRNA | URS000057721B | RNA10415 | 11 | 34 | 2.194861625 | 0.008409812 |
| endo-siRNA | URS00001EEBE4 | RNA14007 | 11 | 34 | 2.194861625 | 0.008409812 |
| endo-siRNA | URS0000047CD4 | RNA14035 | 402 | 95 | -1.498101004 | 0.011772171 |
| endo-siRNA | URS00001E3449 | RNA10456 | 29 | 2 | -3.212834117 | 0.011772171 |
| endo-siRNA | URS0000075343 | RNA600 | 47 | 6 | -2.368231719 | 0.011772171 |
| endo-siRNA | URS0000518611 | RNA13058 | 0 | 11 | 6.792722394 | 0.011772171 |
| endo-siRNA | URS0000599235 | RNA8833 | 0 | 11 | 6.792722394 | 0.011772171 |
| endo-siRNA | URS00001B6AFF | RNA9541 | 0 | 11 | 6.792722394 | 0.011772171 |
| endo-siRNA | URS0000112D6A | RNA11679 | 15 | 0 | -6.659340573 | 0.011772171 |
| endo-siRNA | URS000015DE8B | RNA7498 | 15 | 0 | -6.659340573 | 0.011772171 |
| endo-siRNA | URS00000C7D1F | RNA14370 | 15 | 0 | -6.659340573 | 0.011772171 |
| endo-siRNA | URS00005EEC87 | RNA7304 | 15 | 0 | -6.659340573 | 0.011772171 |
| endo-siRNA | URS000037EE7F | RNA13751 | 8 | 28 | 2.367838478 | 0.011939895 |
| endo-siRNA | URS00000313C6 | RNA13951 | 7 | 25 | 2.393816191 | 0.011979848 |
| endo-siRNA | URS00003C8644 | RNA3635 | 42 | 5 | -2.464812649 | 0.014292302 |
| endo-siRNA | URS00005A561C | RNA13940 | 19 | 47 | 1.880508474 | 0.016298867 |
| endo-siRNA | URS000028A47B | RNA9487 | 0 | 10 | 6.656519535 | 0.017111944 |
| endo-siRNA | URS00000D7E4A | RNA11956 | 0 | 10 | 6.656519535 | 0.017111944 |
| endo-siRNA | URS0000131954 | RNA9867 | 0 | 10 | 6.656519535 | 0.017111944 |
| endo-siRNA | URS000029357C | RNA9497 | 0 | 10 | 6.656519535 | 0.017111944 |
| endo-siRNA | URS000000CC73 | RNA858 | 14 | 0 | -6.560824035 | 0.017111944 |
| endo-siRNA | URS00000D960D | RNA7490 | 14 | 0 | -6.560824035 | 0.017111944 |
| endo-siRNA | URS00000B8250 | RNA2897 | 14 | 0 | -6.560824035 | 0.017111944 |
| endo-siRNA | URS00002F138F | RNA6556 | 14 | 0 | -6.560824035 | 0.017111944 |
| endo-siRNA | URS0000105441 | RNA9406 | 22 | 1 | -3.749454868 | 0.018510408 |
| endo-siRNA | URS000006C2B9 | RNA11963 | 22 | 1 | -3.749454868 | 0.018510408 |
| endo-siRNA | URS000026DD30 | RNA9630 | 57 | 9 | -2.06868002 | 0.018510408 |
| endo-siRNA | URS00002C14D4 | RNA490 | 553 | 141 | -1.388838 | 0.018510408 |
| endo-siRNA | URS00001E65FD | RNA9507 | 6 | 21 | 2.360733837 | 0.018787141 |
| endo-siRNA | URS000018381E | RNA5696 | 4 | 19 | 2.784552798 | 0.018811139 |
| endo-siRNA | URS000028DC04 | RNA1256 | 14 | 37 | 1.972747073 | 0.019226455 |
| endo-siRNA | URS000059FC86 | RNA10681 | 41 | 83 | 1.596075506 | 0.019310184 |
| endo-siRNA | URS0000362935 | RNA12006 | 52 | 8 | -2.104535805 | 0.022809454 |
| endo-siRNA | URS000059F59A | RNA11793 | 32 | 4 | -2.388962654 | 0.022910908 |
| endo-siRNA | URS00003B74BD | RNA3540 | 48 | 8 | -1.989404078 | 0.024533743 |
| endo-siRNA | URS00002FCC71 | RNA9612 | 21 | 1 | -3.682805467 | 0.024825663 |
| endo-siRNA | URS000056B808 | RNA5924 | 25 | 2 | -2.999895701 | 0.024825663 |
| endo-siRNA | URS00001CC18B | RNA12022 | 54 | 9 | -1.990887693 | 0.024928083 |
| endo-siRNA | URS00004C8AA8 | RNA1209 | 13 | 0 | -6.455083863 | 0.025027984 |
| endo-siRNA | URS0000433375 | RNA10182 | 13 | 0 | -6.455083863 | 0.025027984 |
| endo-siRNA | URS0000148D55 | RNA2185 | 13 | 0 | -6.455083863 | 0.025027984 |
| endo-siRNA | URS00002439FD | RNA13574 | 13 | 0 | -6.455083863 | 0.025027984 |
| endo-siRNA | URS0000101CFC | RNA4172 | 13 | 0 | -6.455083863 | 0.025027984 |
| endo-siRNA | URS00005E7B7A | RNA10197 | 13 | 0 | -6.455083863 | 0.025027984 |
| endo-siRNA | URS0000539CE3 | RNA3355 | 12 | 31 | 1.938091084 | 0.026039681 |
| endo-siRNA | URS000027B3D9 | RNA9731 | 51 | 8 | -2.076602728 | 0.026550332 |
| endo-siRNA | URS00005B1D49 | RNA970 | 8 | 24 | 2.146302504 | 0.026630723 |
| endo-siRNA | URS00002CB103 | RNA6569 | 8 | 24 | 2.146302504 | 0.026630723 |
| endo-siRNA | URS0000489BC3 | RNA11979 | 77 | 16 | -1.678479385 | 0.027745572 |
| endo-siRNA | URS000009ACA0 | RNA883 | 86 | 18 | -1.668736421 | 0.028727002 |
| endo-siRNA | URS00004B5FD1 | RNA679 | 9 | 26 | 2.094344644 | 0.030603288 |
| endo-siRNA | URS00001FAD29 | RNA5204 | 62 | 12 | -1.778617215 | 0.030662794 |
| endo-siRNA | URS00005B76EA | RNA11685 | 28 | 3 | -2.600620324 | 0.030662794 |
| endo-siRNA | URS0000420785 | RNA2990 | 33 | 64 | 1.533436559 | 0.03248979 |
| endo-siRNA | URS00004F517F | RNA10273 | 20 | 1 | -3.612927241 | 0.03357886 |
| endo-siRNA | URS00004AFE15 | RNA5121 | 20 | 1 | -3.612927241 | 0.03357886 |
| endo-siRNA | URS00005713E5 | RNA12013 | 67 | 13 | -1.775691742 | 0.03424073 |
| endo-siRNA | URS00003CD7AB | RNA11618 | 115 | 27 | -1.505028613 | 0.034932276 |
| endo-siRNA | URS00001D4927 | RNA2457 | 54 | 10 | -1.840472711 | 0.036173824 |
| endo-siRNA | URS00003960A3 | RNA1008 | 120 | 29 | -1.463625045 | 0.036795392 |
| endo-siRNA | URS000033242E | RNA9879 | 20 | 43 | 1.67903373 | 0.037187161 |
| endo-siRNA | URS00001FD56A | RNA9921 | 36 | 5 | -2.243274992 | 0.037204243 |
| endo-siRNA | URS00005E64CF | RNA5634 | 9 | 25 | 2.037982462 | 0.037204243 |
| endo-siRNA | URS00002BA668 | RNA14340 | 12 | 0 | -6.340976307 | 0.037762292 |
| endo-siRNA | URS000047ADDF | RNA897 | 12 | 0 | -6.340976307 | 0.037762292 |
| endo-siRNA | URS00003DEB12 | RNA433 | 26 | 3 | -2.494295884 | 0.03922985 |
| endo-siRNA | URS000025FEA1 | RNA13073 | 13 | 31 | 1.823983528 | 0.039954153 |
| endo-siRNA | URS0000046070 | RNA6805 | 605 | 173 | -1.223572549 | 0.043070447 |
| endo-siRNA | URS0000380356 | RNA10183 | 19 | 1 | -3.539491352 | 0.043943988 |
| endo-siRNA | URS0000147D35 | RNA6464 | 3 | 14 | 2.744451086 | 0.043943988 |
| endo-siRNA | URS00001BFF3D | RNA14276 | 3 | 14 | 2.744451086 | 0.043943988 |
| endo-siRNA | URS00002AA858 | RNA8353 | 2 | 11 | 2.950359295 | 0.043943988 |
| endo-siRNA | URS00002C0BA7 | RNA11292 | 2 | 11 | 2.950359295 | 0.043943988 |
| endo-siRNA | URS00001D0B48 | RNA13989 | 29 | 4 | -2.247639252 | 0.043943988 |
| endo-siRNA | URS00002733EF | RNA614 | 29 | 4 | -2.247639252 | 0.043943988 |
| endo-siRNA | URS00000A4427 | RNA4827 | 34 | 62 | 1.444828697 | 0.046757375 |
| endo-siRNA | URS00002F7978 | RNA9398 | 63 | 13 | -1.687086915 | 0.04828782 |
| endo-siRNA | URS00002EFDAC | RNA4265 | 85 | 138 | 1.279751377 | 0.048880222 |
| endo-siRNA | URS00002D18A1 | RNA9862 | 5 | 18 | 2.395501255 | 0.048880222 |
| endo-siRNA | URS00000DEDAD | RNA2664 | 5 | 18 | 2.395501255 | 0.048880222 |
| endo-siRNA | URS00000E1149 | RNA9861 | 6 | 18 | 2.139481879 | 0.048880222 |
| endo-siRNA | URS0000139E85 | RNA13991 | 145 | 38 | -1.347562757 | 0.049149743 |
| endo-siRNA | URS0000224368 | RNA5942 | 48 | 9 | -1.82146176 | 0.051312399 |
| endo-siRNA | URS0000099FC9 | RNA10423 | 81 | 19 | -1.504894151 | 0.052481103 |
| endo-siRNA | URS000041D767 | RNA14365 | 33 | 5 | -2.118287787 | 0.053833605 |
| endo-siRNA | URS00002FB927 | RNA14268 | 22 | 2 | -2.816643079 | 0.053869583 |
| endo-siRNA | URS0000269AF5 | RNA8884 | 21 | 2 | -2.749993678 | 0.053869583 |
| endo-siRNA | URS000010D03D | RNA10033 | 21 | 2 | -2.749993678 | 0.053869583 |
| endo-siRNA | URS00005D00BE | RNA10181 | 21 | 2 | -2.749993678 | 0.053869583 |
| endo-siRNA | URS00004F1CAD | RNA862 | 61 | 13 | -1.640656592 | 0.053869583 |
| endo-siRNA | URS000032EC9B | RNA10443 | 28 | 4 | -2.197278083 | 0.054256875 |
| endo-siRNA | URS000022619C | RNA6083 | 27 | 4 | -2.145095135 | 0.054256875 |
| endo-siRNA | URS00001AB1F7 | RNA5363 | 27 | 4 | -2.145095135 | 0.054256875 |
| endo-siRNA | URS000057CD09 | RNA3573 | 11 | 0 | -6.217062441 | 0.054256875 |
| endo-siRNA | URS00004031BC | RNA5389 | 11 | 0 | -6.217062441 | 0.054256875 |
| endo-siRNA | URS000054CCF2 | RNA9538 | 11 | 0 | -6.217062441 | 0.054256875 |
| endo-siRNA | URS00001253E8 | RNA11985 | 11 | 0 | -6.217062441 | 0.054256875 |
| endo-siRNA | URS000030F2D3 | RNA3317 | 11 | 0 | -6.217062441 | 0.054256875 |
| endo-siRNA | URS00002ED837 | RNA2531 | 11 | 0 | -6.217062441 | 0.054256875 |
| endo-siRNA | URS000059F8DD | RNA11983 | 11 | 0 | -6.217062441 | 0.054256875 |
| endo-siRNA | URS00005F1659 | RNA2182 | 11 | 0 | -6.217062441 | 0.054256875 |
| endo-siRNA | URS000032B789 | RNA6505 | 11 | 0 | -6.217062441 | 0.054256875 |
| endo-siRNA | URS00000E0C41 | RNA13003 | 11 | 0 | -6.217062441 | 0.054256875 |
| endo-siRNA | URS000043BA6C | RNA1211 | 11 | 0 | -6.217062441 | 0.054256875 |
| endo-siRNA | URS00005151DA | RNA3271 | 11 | 0 | -6.217062441 | 0.054256875 |
| endo-siRNA | URS0000168F80 | RNA8918 | 55 | 12 | -1.60622336 | 0.056100069 |
| endo-siRNA | URS000031A3BE | RNA992 | 141 | 38 | -1.30724728 | 0.057077117 |
| endo-siRNA | URS00000E5425 | RNA10001 | 69 | 16 | -1.520542223 | 0.05709384 |
| endo-siRNA | URS0000137638 | RNA7794 | 82 | 20 | -1.448941239 | 0.057176992 |
| endo-siRNA | URS000041E26F | RNA5934 | 175 | 48 | -1.282364411 | 0.057176992 |
| endo-siRNA | URS000054F2B7 | RNA4348 | 18 | 1 | -3.462116016 | 0.057176992 |
| endo-siRNA | URS0000391E6E | RNA8241 | 18 | 1 | -3.462116016 | 0.057176992 |
| endo-siRNA | URS0000374743 | RNA2835 | 3 | 13 | 2.63832366 | 0.057176992 |
| endo-siRNA | URS00005F84EC | RNA9913 | 39 | 7 | -1.880979681 | 0.059010557 |
| endo-siRNA | URS00000F441D | RNA11998 | 38 | 7 | -1.843650316 | 0.059010557 |
| endo-siRNA | URS00003A57A3 | RNA10388 | 60 | 13 | -1.616868784 | 0.059071908 |
| endo-siRNA | URS00000C41F9 | RNA7064 | 1 | 10 | 3.716928397 | 0.059159749 |
| endo-siRNA | URS000025CD17 | RNA5519 | 2 | 10 | 2.814156436 | 0.059159749 |
| endo-siRNA | URS00003E86E3 | RNA6003 | 2 | 10 | 2.814156436 | 0.059159749 |
| endo-siRNA | URS0000188AB8 | RNA4274 | 24 | 3 | -2.379507585 | 0.059437197 |
| endo-siRNA | URS000028D498 | RNA818 | 422 | 127 | -1.149676687 | 0.062131011 |
| endo-siRNA | URS00000998CD | RNA3374 | 26 | 4 | -2.090953642 | 0.066768258 |
| endo-siRNA | URS0000451FBD | RNA4797 | 26 | 4 | -2.090953642 | 0.066768258 |
| endo-siRNA | URS00003DFD1A | RNA9645 | 182 | 52 | -1.22365425 | 0.067128118 |
| endo-siRNA | URS00005623CA | RNA9548 | 215 | 62 | -1.210560872 | 0.067839115 |
| endo-siRNA | URS0000238EBB | RNA2725 | 20 | 2 | -2.680115452 | 0.067839115 |
| endo-siRNA | URS00002C1A52 | RNA3359 | 20 | 2 | -2.680115452 | 0.067839115 |
| endo-siRNA | URS0000218B5C | RNA2828 | 4 | 14 | 2.346678497 | 0.067839115 |
| endo-siRNA | URS00002F7278 | RNA14010 | 4 | 14 | 2.346678497 | 0.067839115 |
| endo-siRNA | URS00003D828A | RNA10424 | 80 | 20 | -1.413383132 | 0.068036513 |
| endo-siRNA | URS00000108ED | RNA14000 | 48 | 10 | -1.671046778 | 0.068168277 |
| endo-siRNA | URS00002AAFE0 | RNA13852 | 17 | 33 | 1.530751023 | 0.068168277 |
| endo-siRNA | URS000024E53B | RNA5393 | 17 | 33 | 1.530751023 | 0.068168277 |
| endo-siRNA | URS00003CD8A8 | RNA9571 | 12 | 26 | 1.685227364 | 0.069059405 |
| endo-siRNA | URS000008979B | RNA5661 | 12 | 26 | 1.685227364 | 0.069059405 |
| endo-siRNA | URS0000593E56 | RNA1344 | 108 | 29 | -1.311821904 | 0.069602352 |
| endo-siRNA | URS0000098E86 | RNA9668 | 110 | 30 | -1.289513623 | 0.070182104 |
| endo-siRNA | URS0000154983 | RNA10156 | 58 | 13 | -1.56808314 | 0.074373296 |
| endo-siRNA | URS000022428C | RNA2920 | 9 | 22 | 1.854342392 | 0.075472033 |
| endo-siRNA | URS00005387AA | RNA14341 | 5 | 16 | 2.22657342 | 0.075472033 |
| endo-siRNA | URS00004A6212 | RNA6647 | 17 | 1 | -3.380354495 | 0.075472033 |
| endo-siRNA | URS000041067E | RNA6034 | 3 | 12 | 2.523764975 | 0.075472033 |
| endo-siRNA | URS000043EBAB | RNA4067 | 197 | 58 | -1.180566585 | 0.076886825 |
| endo-siRNA | URS00002E03E7 | RNA12140 | 182 | 53 | -1.196225823 | 0.079048176 |
| endo-siRNA | URS0000539E07 | RNA895 | 10 | 0 | -6.081496947 | 0.079048176 |
| endo-siRNA | URS000024B6B1 | RNA8664 | 10 | 0 | -6.081496947 | 0.079048176 |
| endo-siRNA | URS00002E7C8F | RNA2478 | 10 | 0 | -6.081496947 | 0.079048176 |
| endo-siRNA | URS00000FC9EA | RNA5425 | 10 | 0 | -6.081496947 | 0.079048176 |
| endo-siRNA | URS000000AD85 | RNA3572 | 10 | 0 | -6.081496947 | 0.079048176 |
| endo-siRNA | URS00005E4467 | RNA5259 | 10 | 0 | -6.081496947 | 0.079048176 |
| endo-siRNA | URS00005C7A02 | RNA2962 | 10 | 0 | -6.081496947 | 0.079048176 |
| endo-siRNA | URS0000344013 | RNA10680 | 45 | 72 | 1.257392677 | 0.079151933 |
| endo-siRNA | URS00001B1CCB | RNA13576 | 25 | 4 | -2.034700836 | 0.080915519 |
| endo-siRNA | URS00004B6971 | RNA10853 | 25 | 4 | -2.034700836 | 0.080915519 |
| endo-siRNA | URS00002C48F7 | RNA7331 | 39 | 8 | -1.690879771 | 0.083094521 |
| endo-siRNA | URS00002C0D42 | RNA5568 | 62 | 15 | -1.45907852 | 0.084137394 |
| endo-siRNA | URS00005BAAFB | RNA13577 | 8 | 19 | 1.810843853 | 0.086599985 |
| endo-siRNA | URS00000E2289 | RNA5530 | 19 | 2 | -2.606679563 | 0.086887296 |
| endo-siRNA | URS000014F013 | RNA5938 | 168 | 50 | -1.164748594 | 0.089519804 |
| endo-siRNA | URS000044181A | RNA13906 | 51 | 12 | -1.497596472 | 0.091495295 |
| endo-siRNA | URS000055D267 | RNA11380 | 51 | 12 | -1.497596472 | 0.091495295 |
| endo-siRNA | URS000057DFE9 | RNA892 | 128 | 37 | -1.206223197 | 0.093092945 |
| endo-siRNA | URS000005E891 | RNA14075 | 56 | 13 | -1.517589867 | 0.093315687 |
| endo-siRNA | URS00005A7EEF | RNA6072 | 157 | 47 | -1.156225342 | 0.093602038 |
| endo-siRNA | URS00001E7A59 | RNA826 | 457 | 147 | -1.05375559 | 0.095671289 |
| endo-siRNA | URS00001229C8 | RNA11684 | 22 | 3 | -2.254790455 | 0.095671289 |
| endo-siRNA | URS00003E3FBE | RNA14120 | 22 | 3 | -2.254790455 | 0.095671289 |
| endo-siRNA | URS000033F70F | RNA2079 | 22 | 3 | -2.254790455 | 0.095671289 |
| endo-siRNA | URS0000316A9C | RNA1165 | 21 | 3 | -2.188141055 | 0.095671289 |
| endo-siRNA | URS00003CF38B | RNA1255 | 30 | 50 | 1.314774932 | 0.096266314 |
| endo-siRNA | URS00003D83FF | RNA14096 | 144 | 43 | -1.159692662 | 0.097673476 |
| endo-siRNA | URS000015FFF6 | RNA607 | 57 | 86 | 1.173377964 | 0.097673476 |
| endo-siRNA | URS00005100B7 | RNA3322 | 37 | 8 | -1.615229439 | 0.097673476 |
| endo-siRNA | URS0000290EA1 | RNA9498 | 98 | 139 | 1.085178465 | 0.099808628 |
| endo-siRNA | URS00003DBF00 | RNA1243 | 42 | 9 | -1.629458088 | 0.099808628 |
| endo-siRNA | URS000016B925 | RNA5894 | 16 | 1 | -3.293679466 | 0.099808628 |
| endo-siRNA | URS000035B35C | RNA2421 | 16 | 1 | -3.293679466 | 0.099808628 |
| endo-siRNA | URS000020D799 | RNA2373 | 15 | 1 | -3.201462396 | 0.099808628 |
| endo-siRNA | URS00004AA461 | RNA3600 | 15 | 1 | -3.201462396 | 0.099808628 |
| endo-siRNA | URS000047F68B | RNA13004 | 15 | 1 | -3.201462396 | 0.099808628 |
| endo-siRNA | URS00000501DA | RNA2708 | 3 | 11 | 2.399318877 | 0.099808628 |
| endo-siRNA | URS00004D5F63 | RNA8617 | 3 | 11 | 2.399318877 | 0.099808628 |
| endo-siRNA | URS00000733CF | RNA4271 | 3 | 11 | 2.399318877 | 0.099808628 |
| endo-siRNA | URS000036DC90 | RNA5930 | 24 | 4 | -1.976165343 | 0.100319845 |
| endo-siRNA | URS00006030E1 | RNA9038 | 24 | 4 | -1.976165343 | 0.100319845 |
| endo-siRNA | URS00002C2B84 | RNA4308 | 169 | 52 | -1.11683036 | 0.100436986 |
| endo-siRNA | URS0000204FD9 | RNA10154 | 113 | 33 | -1.191213539 | 0.10290317 |
| endo-siRNA | URS00002DA245 | RNA9905 | 54 | 13 | -1.465265069 | 0.10290317 |
| endo-siRNA | URS00003E1E4E | RNA10471 | 546 | 180 | -1.018384551 | 0.10290317 |
| endo-siRNA | URS0000176C3A | RNA11945 | 59 | 15 | -1.387702158 | 0.103343574 |
| endo-siRNA | URS000036DC94 | RNA4234 | 74 | 20 | -1.301127132 | 0.103948365 |
| endo-siRNA | URS00005FFFA9 | RNA625 | 9 | 20 | 1.717492294 | 0.108387945 |
| endo-siRNA | URS000005272A | RNA10340 | 219 | 70 | -1.062315863 | 0.110381731 |
| endo-siRNA | URS000031D94D | RNA8677 | 31 | 6 | -1.770204249 | 0.111452535 |
| endo-siRNA | URS00003531F1 | RNA5032 | 95 | 27 | -1.229789452 | 0.112428212 |
| endo-siRNA | URS00006117FE | RNA7045 | 4 | 13 | 2.240551072 | 0.112428212 |
| endo-siRNA | URS00005BA5E3 | RNA12471 | 4 | 13 | 2.240551072 | 0.112428212 |
| endo-siRNA | URS000059AE1D | RNA1367 | 41 | 63 | 1.198867686 | 0.112507622 |
| endo-siRNA | URS00005C64BF | RNA1288 | 34 | 7 | -1.683852312 | 0.112507622 |
| endo-siRNA | URS00000A4B78 | RNA9036 | 34 | 7 | -1.683852312 | 0.112507622 |
| endo-siRNA | URS0000352E5D | RNA1335 | 36 | 8 | -1.575862748 | 0.113565109 |
| endo-siRNA | URS000052E559 | RNA3547 | 13 | 25 | 1.514757627 | 0.113565109 |
| endo-siRNA | URS00002FE054 | RNA13851 | 13 | 25 | 1.514757627 | 0.113565109 |
| endo-siRNA | URS000006E282 | RNA10472 | 41 | 9 | -1.594817788 | 0.114932675 |
| endo-siRNA | URS000009FD5E | RNA5704 | 15 | 28 | 1.473382955 | 0.114932675 |
| endo-siRNA | URS00001925F8 | RNA3473 | 44 | 10 | -1.545924096 | 0.115258943 |
| endo-siRNA | URS000033D602 | RNA11826 | 113 | 34 | -1.148273168 | 0.115769562 |
| endo-siRNA | URS00004E7BD4 | RNA4711 | 214 | 69 | -1.049747542 | 0.117199485 |
| endo-siRNA | URS0000197336 | RNA5828 | 20 | 3 | -2.118262829 | 0.119072217 |
| endo-siRNA | URS0000347A7F | RNA1811 | 5 | 14 | 2.035209401 | 0.119072217 |
| endo-siRNA | URS0000227629 | RNA8233 | 235 | 310 | 0.981266719 | 0.121823622 |
| endo-siRNA | URS00002A69DA | RNA9744 | 267 | 88 | -1.018320265 | 0.121932993 |
| endo-siRNA | URS00005B622F | RNA3955 | 6 | 16 | 1.970554043 | 0.124082356 |
| endo-siRNA | URS00004ADDCE | RNA9959 | 23 | 4 | -1.915154042 | 0.124082356 |
| endo-siRNA | URS0000153679 | RNA8347 | 23 | 4 | -1.915154042 | 0.124082356 |
| endo-siRNA | URS0000342DBB | RNA11600 | 23 | 4 | -1.915154042 | 0.124082356 |
| endo-siRNA | URS0000248BB8 | RNA13673 | 23 | 4 | -1.915154042 | 0.124082356 |
| endo-siRNA | URS00005B5699 | RNA6052 | 7 | 16 | 1.753193323 | 0.124082356 |
| endo-siRNA | URS000038E5F4 | RNA9296 | 7 | 16 | 1.753193323 | 0.124082356 |
| endo-siRNA | URS000014A416 | RNA14001 | 143 | 191 | 0.998915444 | 0.125541321 |
| endo-siRNA | URS00002AC877 | RNA10475 | 67 | 18 | -1.309272462 | 0.125541321 |
| endo-siRNA | URS00001CF172 | RNA4812 | 67 | 18 | -1.309272462 | 0.125541321 |
| endo-siRNA | URS00005AFE6A | RNA10474 | 195 | 63 | -1.04675472 | 0.127925936 |
| endo-siRNA | URS00004A548C | RNA11939 | 209 | 68 | -1.03669472 | 0.129674235 |
| endo-siRNA | URS000057DA0A | RNA1351 | 27 | 5 | -1.830229974 | 0.129674235 |
| endo-siRNA | URS00005418B3 | RNA1017 | 9 | 19 | 1.643869876 | 0.129674235 |
| endo-siRNA | URS0000470FCB | RNA13993 | 47 | 12 | -1.380119719 | 0.130300569 |
| endo-siRNA | URS00001998D0 | RNA3571 | 51 | 73 | 1.097256095 | 0.130300569 |
| endo-siRNA | URS00000F8015 | RNA725 | 42 | 10 | -1.479043105 | 0.130300569 |
| endo-siRNA | URS00001E2130 | RNA885 | 33 | 7 | -1.640975453 | 0.130300569 |
| endo-siRNA | URS0000068B0B | RNA3461 | 33 | 7 | -1.640975453 | 0.130300569 |
| endo-siRNA | URS00000401B8 | RNA5996 | 33 | 7 | -1.640975453 | 0.130300569 |
| endo-siRNA | URS0000418D32 | RNA3293 | 11 | 23 | 1.632985126 | 0.130300569 |
| endo-siRNA | URS0000250D2E | RNA9586 | 11 | 23 | 1.632985126 | 0.130300569 |
| endo-siRNA | URS00003EA90E | RNA6910 | 32 | 7 | -1.59678516 | 0.130300569 |
| endo-siRNA | URS00001AE921 | RNA3546 | 32 | 7 | -1.59678516 | 0.130300569 |
| endo-siRNA | URS000008F2A7 | RNA6822 | 40 | 9 | -1.559325242 | 0.130300569 |
| endo-siRNA | URS00003F1394 | RNA4658 | 80 | 23 | -1.212686887 | 0.130300569 |
| endo-siRNA | URS00002F53CE | RNA6474 | 14 | 26 | 1.465379636 | 0.130300569 |
| endo-siRNA | URS00004F751D | RNA11900 | 3 | 10 | 2.263116019 | 0.130300569 |
| endo-siRNA | URS0000293921 | RNA5416 | 3 | 10 | 2.263116019 | 0.130300569 |
| endo-siRNA | URS00001FFDE7 | RNA13167 | 3 | 10 | 2.263116019 | 0.130300569 |
| endo-siRNA | URS000042EEA0 | RNA10391 | 78 | 22 | -1.240076332 | 0.130300569 |
| endo-siRNA | URS00005975A0 | RNA8576 | 77 | 22 | -1.221496587 | 0.130300569 |
| endo-siRNA | URS000000CF3A | RNA2693 | 92 | 126 | 1.034628663 | 0.131644839 |
| endo-siRNA | URS00002CB3B0 | RNA928 | 96 | 29 | -1.142146804 | 0.13305548 |
| endo-siRNA | URS00002B9393 | RNA861 | 409 | 140 | -0.964058011 | 0.133528957 |
| endo-siRNA | URS00006059CC | RNA13994 | 109 | 34 | -1.096348658 | 0.137027946 |
| endo-siRNA | URS000007ED4D | RNA5780 | 58 | 16 | -1.270591696 | 0.140225023 |
| endo-siRNA | URS00003B0BF8 | RNA3543 | 81 | 24 | -1.1694355 | 0.140677349 |
| endo-siRNA | URS000060C64B | RNA913 | 826 | 291 | -0.922774868 | 0.142381249 |
| endo-siRNA | URS00005B8292 | RNA1339 | 17 | 2 | -2.447542706 | 0.142381249 |
| endo-siRNA | URS00000E26BA | RNA8905 | 17 | 2 | -2.447542706 | 0.142381249 |
| endo-siRNA | URS000040AEE1 | RNA14146 | 17 | 2 | -2.447542706 | 0.142381249 |
| endo-siRNA | URS000033EC6F | RNA10865 | 17 | 2 | -2.447542706 | 0.142381249 |
| endo-siRNA | URS000006EF72 | RNA14336 | 4 | 12 | 2.125992387 | 0.142381249 |
| endo-siRNA | URS00000DC896 | RNA10441 | 132 | 42 | -1.068165685 | 0.143227961 |
| endo-siRNA | URS0000040280 | RNA8899 | 21 | 35 | 1.31293957 | 0.143569241 |
| endo-siRNA | URS000049445B | RNA2692 | 82 | 110 | 1.00459887 | 0.144275434 |
| endo-siRNA | URS00004A54B3 | RNA1246 | 48 | 13 | -1.295839136 | 0.145031412 |
| endo-siRNA | URS000004918C | RNA1183 | 4400 | 1580 | -0.895423447 | 0.14536715 |
| endo-siRNA | URS00004F2747 | RNA6804 | 61 | 84 | 1.041871378 | 0.146525292 |
| endo-siRNA | URS00001C0444 | RNA6084 | 43 | 11 | -1.376668269 | 0.148147247 |
| endo-siRNA | URS00001852B4 | RNA5213 | 67 | 19 | -1.231690016 | 0.148851604 |
| endo-siRNA | URS00004E3932 | RNA825 | 610 | 215 | -0.922042114 | 0.149877215 |
| endo-siRNA | URS00002F8156 | RNA14003 | 116 | 152 | 0.971146842 | 0.149877215 |
| endo-siRNA | URS000053473C | RNA837 | 178 | 59 | -1.009745348 | 0.15074634 |
| endo-siRNA | URS000007CB0B | RNA10009 | 175 | 58 | -1.009863754 | 0.151647044 |
| endo-siRNA | URS0000562A01 | RNA7658 | 27 | 42 | 1.214991641 | 0.151790022 |
| endo-siRNA | URS00002D851D | RNA6762 | 128 | 41 | -1.058504438 | 0.151790022 |
| endo-siRNA | URS000018C260 | RNA915 | 21 | 4 | -1.784798813 | 0.151790022 |
| endo-siRNA | URS00002580C3 | RNA2716 | 21 | 4 | -1.784798813 | 0.151790022 |
| endo-siRNA | URS0000595C7E | RNA13917 | 21 | 4 | -1.784798813 | 0.151790022 |
| endo-siRNA | URS00003CD352 | RNA920 | 21 | 4 | -1.784798813 | 0.151790022 |
| endo-siRNA | URS00003401C7 | RNA12183 | 7 | 15 | 1.660681888 | 0.151790022 |
| endo-siRNA | URS00004AEFF3 | RNA8678 | 78 | 23 | -1.176230185 | 0.152314123 |
| endo-siRNA | URS00003864BF | RNA463 | 36 | 53 | 1.136837411 | 0.152314123 |
| endo-siRNA | URS00004F32D0 | RNA9508 | 11 | 22 | 1.569138979 | 0.152722359 |
| endo-siRNA | URS000040D83D | RNA5896 | 31 | 7 | -1.551198415 | 0.152722359 |
| endo-siRNA | URS000048116D | RNA12426 | 8 | 17 | 1.651268587 | 0.152847185 |
| endo-siRNA | URS0000384B84 | RNA12122 | 29 | 6 | -1.674467592 | 0.153172681 |
| endo-siRNA | URS0000562AA2 | RNA13553 | 10 | 20 | 1.567854374 | 0.153172681 |
| endo-siRNA | URS0000509BD0 | RNA5051 | 42 | 60 | 1.093952508 | 0.156008225 |
| endo-siRNA | URS000009AAE8 | RNA10151 | 242 | 83 | -0.96086461 | 0.156563595 |
| endo-siRNA | URS0000358902 | RNA12130 | 236 | 81 | -0.959813637 | 0.1584963 |
| endo-siRNA | URS0000380218 | RNA2726 | 47 | 66 | 1.069511961 | 0.16117399 |
| endo-siRNA | URS00003890B9 | RNA9818 | 128 | 42 | -1.023822698 | 0.169077671 |
| endo-siRNA | URS000023D393 | RNA5072 | 150 | 50 | -1.001404168 | 0.171073993 |
| endo-siRNA | URS000008A2D9 | RNA450 | 40 | 10 | -1.40891026 | 0.173868684 |
| endo-siRNA | URS0000108266 | RNA4819 | 110 | 35 | -1.067806889 | 0.173931841 |
| endo-siRNA | URS00003B3EC0 | RNA914 | 575 | 208 | -0.884546932 | 0.174621382 |
| endo-siRNA | URS00004EDD57 | RNA4217 | 56 | 16 | -1.220098422 | 0.175147916 |
| endo-siRNA | URS00002C54A3 | RNA14352 | 40 | 56 | 1.064721336 | 0.175543603 |
| endo-siRNA | URS00004174AD | RNA6066 | 37 | 9 | -1.447287121 | 0.176440418 |
| endo-siRNA | URS00001C15E6 | RNA945 | 37 | 9 | -1.447287121 | 0.176440418 |
| endo-siRNA | URS00001737CB | RNA9920 | 94 | 30 | -1.063076864 | 0.176704162 |
| endo-siRNA | URS000035A233 | RNA3421 | 94 | 30 | -1.063076864 | 0.176704162 |
| endo-siRNA | URS0000519FE5 | RNA5999 | 80 | 25 | -1.092892964 | 0.177593158 |
| endo-siRNA | URS000036FBF4 | RNA715 | 80 | 25 | -1.092892964 | 0.177593158 |
| endo-siRNA | URS000054DE47 | RNA2506 | 91 | 29 | -1.065102505 | 0.178342792 |
| endo-siRNA | URS000024F22C | RNA11399 | 13 | 1 | -2.997205685 | 0.178342792 |
| endo-siRNA | URS00004E9CE9 | RNA6643 | 13 | 1 | -2.997205685 | 0.178342792 |
| endo-siRNA | URS00003F0C6E | RNA9618 | 13 | 1 | -2.997205685 | 0.178342792 |
| endo-siRNA | URS00001953DC | RNA7536 | 13 | 1 | -2.997205685 | 0.178342792 |
| endo-siRNA | URS00000BF334 | RNA8504 | 22 | 35 | 1.246290169 | 0.180015126 |
| endo-siRNA | URS0000562707 | RNA7017 | 44 | 60 | 1.027071518 | 0.180502208 |
| endo-siRNA | URS00002E524B | RNA758 | 21 | 33 | 1.228300051 | 0.183338625 |
| endo-siRNA | URS000000FAC5 | RNA13934 | 12 | 21 | 1.378422109 | 0.183338625 |
| endo-siRNA | URS00003ED9A3 | RNA435 | 997 | 368 | -0.855598911 | 0.185274789 |
| endo-siRNA | URS0000549BAB | RNA13683 | 28 | 6 | -1.624106423 | 0.185378896 |
| endo-siRNA | URS000017A5D4 | RNA10498 | 16 | 2 | -2.360867677 | 0.185378896 |
| endo-siRNA | URS0000216D5B | RNA5498 | 16 | 2 | -2.360867677 | 0.185378896 |
| endo-siRNA | URS00004A541C | RNA5978 | 16 | 2 | -2.360867677 | 0.185378896 |
| endo-siRNA | URS0000488952 | RNA505 | 16 | 2 | -2.360867677 | 0.185378896 |
| endo-siRNA | URS000020F314 | RNA14203 | 15 | 2 | -2.268650607 | 0.185378896 |
| endo-siRNA | URS00004291E0 | RNA449 | 4 | 11 | 2.001546289 | 0.185378896 |
| endo-siRNA | URS00002E98BA | RNA5937 | 25 | 5 | -1.719835676 | 0.1864436 |
| endo-siRNA | URS00002A0CA9 | RNA9864 | 25 | 5 | -1.719835676 | 0.1864436 |
| endo-siRNA | URS00002034DE | RNA11706 | 101 | 33 | -1.029473095 | 0.187062119 |
| endo-siRNA | URS0000173CC2 | RNA3870 | 39 | 54 | 1.048737061 | 0.187062119 |
| endo-siRNA | URS00001BB392 | RNA6091 | 18 | 3 | -1.967451603 | 0.187062119 |
| endo-siRNA | URS0000572163 | RNA7488 | 18 | 3 | -1.967451603 | 0.187062119 |
| endo-siRNA | URS00005D48B2 | RNA7343 | 18 | 3 | -1.967451603 | 0.187062119 |
| endo-siRNA | URS00001E090B | RNA575 | 18 | 3 | -1.967451603 | 0.187062119 |
| endo-siRNA | URS0000619549 | RNA11539 | 18 | 3 | -1.967451603 | 0.187062119 |
| endo-siRNA | URS00000CC07D | RNA4521 | 5 | 13 | 1.929081976 | 0.187062119 |
| endo-siRNA | URS00000E0E9F | RNA770 | 7 | 14 | 1.561829304 | 0.187062119 |
| endo-siRNA | URS0000551727 | RNA5706 | 7 | 14 | 1.561829304 | 0.187062119 |
| endo-siRNA | URS0000264D89 | RNA9464 | 199 | 70 | -0.924252586 | 0.187272381 |
| endo-siRNA | URS0000307CF3 | RNA4197 | 54 | 16 | -1.167773625 | 0.188336755 |
| endo-siRNA | URS00002A04C8 | RNA14095 | 127 | 43 | -0.978653129 | 0.189609513 |
| endo-siRNA | URS00000B7159 | RNA9798 | 494 | 182 | -0.858103587 | 0.192582088 |
| endo-siRNA | URS00002718BF | RNA12014 | 39 | 10 | -1.372522471 | 0.192870685 |
| endo-siRNA | URS0000435D79 | RNA754 | 17 | 27 | 1.242213802 | 0.192870685 |
| endo-siRNA | URS0000369896 | RNA4169 | 139 | 48 | -0.950414079 | 0.195986162 |
| endo-siRNA | URS00004C573B | RNA10429 | 36 | 9 | -1.40792043 | 0.1974431 |
| endo-siRNA | URS000002357E | RNA12883 | 218 | 78 | -0.8998098 | 0.197455775 |
| endo-siRNA | URS00001F1B1E | RNA5302 | 89 | 29 | -1.033094604 | 0.199395484 |
| endo-siRNA | URS00002F3401 | RNA8234 | 250 | 301 | 0.849563754 | 0.200561443 |
| endo-siRNA | URS0000234750 | RNA5764 | 14 | 23 | 1.289223531 | 0.202117716 |
| endo-siRNA | URS000040A02B | RNA13697 | 132 | 45 | -0.968858755 | 0.202714755 |
| endo-siRNA | URS0000497046 | RNA5080 | 121 | 41 | -0.977465287 | 0.205374751 |
| endo-siRNA | URS00001AB673 | RNA14034 | 109 | 37 | -0.974701616 | 0.207921706 |
| endo-siRNA | URS0000407EC0 | RNA1242 | 29 | 7 | -1.455461759 | 0.21244989 |
| endo-siRNA | URS00002CB036 | RNA10465 | 29 | 7 | -1.455461759 | 0.21244989 |
| endo-siRNA | URS000052478E | RNA4086 | 29 | 7 | -1.455461759 | 0.21244989 |
| endo-siRNA | URS0000290F6F | RNA13695 | 29 | 7 | -1.455461759 | 0.21244989 |
| endo-siRNA | URS0000598110 | RNA8486 | 87 | 29 | -1.000360427 | 0.213661415 |
| endo-siRNA | URS000046D5C4 | RNA9700 | 171 | 206 | 0.850198288 | 0.214043551 |
| endo-siRNA | URS00005FCEFB | RNA436 | 1138 | 434 | -0.808477589 | 0.215327086 |
| endo-siRNA | URS000054779D | RNA10155 | 530 | 200 | -0.823565021 | 0.216131726 |
| endo-siRNA | URS00003AC99E | RNA744 | 59 | 18 | -1.126262887 | 0.216318601 |
| endo-siRNA | URS00005DE381 | RNA733 | 26 | 6 | -1.517781983 | 0.216318601 |
| endo-siRNA | URS0000434BFB | RNA700 | 26 | 6 | -1.517781983 | 0.216318601 |
| endo-siRNA | URS00005DF3FD | RNA580 | 26 | 6 | -1.517781983 | 0.216318601 |
| endo-siRNA | URS000010C9F7 | RNA619 | 10 | 1 | -2.62361877 | 0.216318601 |
| endo-siRNA | URS00004619DE | RNA3804 | 10 | 1 | -2.62361877 | 0.216318601 |
| endo-siRNA | URS00002E7F44 | RNA5081 | 10 | 1 | -2.62361877 | 0.216318601 |
| endo-siRNA | URS00004DE200 | RNA4739 | 10 | 1 | -2.62361877 | 0.216318601 |
| endo-siRNA | URS000044361B | RNA4703 | 10 | 1 | -2.62361877 | 0.216318601 |
| endo-siRNA | URS00000879FF | RNA9391 | 10 | 1 | -2.62361877 | 0.216318601 |
| endo-siRNA | URS0000020FE3 | RNA5637 | 10 | 1 | -2.62361877 | 0.216318601 |
| endo-siRNA | URS0000552D97 | RNA776 | 10 | 1 | -2.62361877 | 0.216318601 |
| endo-siRNA | URS000025ED59 | RNA81 | 10 | 1 | -2.62361877 | 0.216318601 |
| endo-siRNA | URS00002BABD8 | RNA5145 | 10 | 1 | -2.62361877 | 0.216318601 |
| endo-siRNA | URS00001496AC | RNA6575 | 10 | 1 | -2.62361877 | 0.216318601 |
| endo-siRNA | URS000017A6FA | RNA5759 | 10 | 1 | -2.62361877 | 0.216318601 |
| endo-siRNA | URS00001DA1A6 | RNA5917 | 48 | 14 | -1.189711711 | 0.216796385 |
| endo-siRNA | URS000050EF43 | RNA10791 | 131 | 46 | -0.926260892 | 0.217500144 |
| endo-siRNA | URS00000CD5D8 | RNA9655 | 636 | 241 | -0.817621108 | 0.218288956 |
| endo-siRNA | URS000011DA30 | RNA14275 | 66 | 83 | 0.91122521 | 0.218288956 |
| endo-siRNA | URS00003117FF | RNA5034 | 9 | 17 | 1.48429461 | 0.219521704 |
| endo-siRNA | URS00002B6D6F | RNA14014 | 127 | 153 | 0.850058529 | 0.220129447 |
| endo-siRNA | URS0000034722 | RNA9768 | 281 | 104 | -0.851254481 | 0.220129447 |
| endo-siRNA | URS00004BFD12 | RNA9500 | 783 | 905 | 0.790902192 | 0.221991691 |
| endo-siRNA | URS00004F6F77 | RNA9656 | 537 | 204 | -0.813934434 | 0.222593268 |
| endo-siRNA | URS00000D6AC0 | RNA3502 | 72 | 23 | -1.060983524 | 0.222593268 |
| endo-siRNA | URS00001E1921 | RNA3452 | 36 | 49 | 1.023848836 | 0.222593268 |
| endo-siRNA | URS00001D1A72 | RNA9813 | 164 | 59 | -0.891667533 | 0.222593268 |
| endo-siRNA | URS0000182926 | RNA4165 | 54 | 17 | -1.080838609 | 0.222835194 |
| endo-siRNA | URS0000468C2C | RNA9800 | 35 | 9 | -1.367449338 | 0.223341019 |
| endo-siRNA | URS00003B82F7 | RNA5820 | 35 | 9 | -1.367449338 | 0.223341019 |
| endo-siRNA | URS00002DCC14 | RNA707 | 8 | 15 | 1.471822136 | 0.223341019 |
| endo-siRNA | URS000054E122 | RNA601 | 8 | 15 | 1.471822136 | 0.223341019 |
| endo-siRNA | URS000026BA6F | RNA14358 | 78 | 26 | -1.000074081 | 0.224980541 |
| endo-siRNA | URS0000586412 | RNA10146 | 43 | 13 | -1.137663486 | 0.226834705 |
| endo-siRNA | URS000040387A | RNA1241 | 107 | 37 | -0.948021307 | 0.227433935 |
| endo-siRNA | URS0000275B44 | RNA13871 | 12 | 1 | -2.883098129 | 0.228785408 |
| endo-siRNA | URS00003473A6 | RNA3158 | 12 | 1 | -2.883098129 | 0.228785408 |
| endo-siRNA | URS0000283A9F | RNA1337 | 12 | 1 | -2.883098129 | 0.228785408 |
| endo-siRNA | URS0000146B8C | RNA8848 | 33 | 9 | -1.282933225 | 0.228785408 |
| endo-siRNA | URS00005ADDBD | RNA5675 | 33 | 9 | -1.282933225 | 0.228785408 |
| endo-siRNA | URS000025E666 | RNA11654 | 32 | 9 | -1.238742932 | 0.228785408 |
| endo-siRNA | URS0000138C83 | RNA13106 | 17 | 3 | -1.885690082 | 0.229170259 |
| endo-siRNA | URS00001ACBE3 | RNA6001 | 17 | 3 | -1.885690082 | 0.229170259 |
| endo-siRNA | URS0000585847 | RNA565 | 17 | 3 | -1.885690082 | 0.229170259 |
| endo-siRNA | URS00002A361F | RNA14011 | 5 | 12 | 1.81452329 | 0.229170259 |
| endo-siRNA | URS00003FDC93 | RNA4332 | 5 | 12 | 1.81452329 | 0.229170259 |
| endo-siRNA | URS00002DBF93 | RNA14375 | 5 | 12 | 1.81452329 | 0.229170259 |
| endo-siRNA | URS0000525E84 | RNA11821 | 146 | 53 | -0.878548426 | 0.229170259 |
| endo-siRNA | URS0000553FDD | RNA681 | 19 | 28 | 1.135353999 | 0.229170259 |
| endo-siRNA | URS00003DD6FB | RNA10742 | 14 | 2 | -2.170134068 | 0.229170259 |
| endo-siRNA | URS000061C42D | RNA11940 | 14 | 2 | -2.170134068 | 0.229170259 |
| endo-siRNA | URS00003E57E4 | RNA13369 | 14 | 2 | -2.170134068 | 0.229170259 |
| endo-siRNA | URS00001798B7 | RNA7348 | 14 | 2 | -2.170134068 | 0.229170259 |
| endo-siRNA | URS00002F50CF | RNA9848 | 4 | 10 | 1.86534343 | 0.229170259 |
| endo-siRNA | URS00003D33DA | RNA1350 | 4 | 10 | 1.86534343 | 0.229170259 |
| endo-siRNA | URS0000547903 | RNA11021 | 4 | 10 | 1.86534343 | 0.229170259 |
| endo-siRNA | URS00002B5610 | RNA1336 | 4 | 10 | 1.86534343 | 0.229170259 |
| endo-siRNA | URS000012EB20 | RNA5294 | 50 | 15 | -1.149573171 | 0.229170259 |
| endo-siRNA | URS000011EC50 | RNA815 | 774 | 299 | -0.789871182 | 0.229939602 |
| endo-siRNA | URS00002DBD87 | RNA8368 | 108 | 38 | -0.923051447 | 0.231983046 |
| endo-siRNA | URS0000304D74 | RNA14054 | 64 | 21 | -1.022082251 | 0.231983046 |
| endo-siRNA | URS00005EC190 | RNA548 | 64 | 21 | -1.022082251 | 0.231983046 |
| endo-siRNA | URS0000521C6A | RNA14235 | 64 | 21 | -1.022082251 | 0.231983046 |
| endo-siRNA | URS000053DC3A | RNA13077 | 93 | 32 | -0.954862063 | 0.232222871 |
| endo-siRNA | URS000023BC6B | RNA908 | 216 | 80 | -0.850042516 | 0.234662606 |
| endo-siRNA | URS0000240FC9 | RNA5935 | 243 | 91 | -0.834207806 | 0.234691974 |
| endo-siRNA | URS0000171E09 | RNA13943 | 47 | 14 | -1.159433608 | 0.234953928 |
| endo-siRNA | URS00004CE5D4 | RNA1248 | 47 | 14 | -1.159433608 | 0.234953928 |
| endo-siRNA | URS0000448196 | RNA3447 | 39 | 11 | -1.236319612 | 0.234953928 |
| endo-siRNA | URS00001AD9E3 | RNA8877 | 76 | 94 | 0.887467995 | 0.237254391 |
| endo-siRNA | URS0000226026 | RNA1001 | 28 | 7 | -1.405100589 | 0.239800262 |
| endo-siRNA | URS000045A625 | RNA14263 | 28 | 7 | -1.405100589 | 0.239800262 |
| endo-siRNA | URS00005C3685 | RNA9749 | 1067 | 419 | -0.766282736 | 0.240743669 |
| endo-siRNA | URS000036C195 | RNA9653 | 161 | 59 | -0.865056952 | 0.243142191 |
| endo-siRNA | URS00000D74F9 | RNA2694 | 105 | 125 | 0.832749179 | 0.245168974 |
| endo-siRNA | URS00004A266E | RNA9894 | 288 | 110 | -0.805890158 | 0.245168974 |
| endo-siRNA | URS000047F23D | RNA14031 | 237 | 89 | -0.830186645 | 0.245168974 |
| endo-siRNA | URS000028763F | RNA9745 | 524 | 204 | -0.778589145 | 0.245168974 |
| endo-siRNA | URS000058C994 | RNA13954 | 89 | 107 | 0.846768538 | 0.24602557 |
| endo-siRNA | URS000059E262 | RNA823 | 993 | 391 | -0.762360239 | 0.246765188 |
| endo-siRNA | URS00000C22BE | RNA9621 | 98 | 35 | -0.901397377 | 0.247634749 |
| endo-siRNA | URS000013E365 | RNA11393 | 25 | 6 | -1.461529176 | 0.247646202 |
| endo-siRNA | URS0000575B0B | RNA8357 | 25 | 6 | -1.461529176 | 0.247646202 |
| endo-siRNA | URS000053664E | RNA7409 | 25 | 6 | -1.461529176 | 0.247646202 |
| endo-siRNA | URS0000346CD3 | RNA8915 | 25 | 6 | -1.461529176 | 0.247646202 |
| endo-siRNA | URS00005974F3 | RNA95 | 25 | 6 | -1.461529176 | 0.247646202 |
| endo-siRNA | URS00000AF1DB | RNA10834 | 34 | 9 | -1.325810085 | 0.250993904 |
| endo-siRNA | URS00001EE37E | RNA4828 | 69 | 23 | -0.999713278 | 0.253479557 |
| endo-siRNA | URS000025F8B6 | RNA5079 | 114 | 41 | -0.891601656 | 0.253479557 |
| endo-siRNA | URS0000041C18 | RNA9751 | 157 | 58 | -0.853414926 | 0.254969441 |
| endo-siRNA | URS00004837A2 | RNA4791 | 175 | 66 | -0.823752002 | 0.254979851 |
| endo-siRNA | URS000031E233 | RNA11705 | 218 | 83 | -0.810284004 | 0.254979851 |
| endo-siRNA | URS00000ADBFD | RNA10405 | 23 | 5 | -1.600288882 | 0.256068751 |
| endo-siRNA | URS0000497ABB | RNA5868 | 23 | 5 | -1.600288882 | 0.256068751 |
| endo-siRNA | URS0000332DEE | RNA5529 | 23 | 5 | -1.600288882 | 0.256068751 |
| endo-siRNA | URS00000AC86E | RNA10672 | 23 | 5 | -1.600288882 | 0.256068751 |
| endo-siRNA | URS00002FBBF5 | RNA2751 | 9 | 16 | 1.397359594 | 0.256068751 |
| endo-siRNA | URS0000086D2D | RNA12662 | 327 | 127 | -0.781873126 | 0.259625889 |
| endo-siRNA | URS00001072D6 | RNA14027 | 31 | 9 | -1.193156187 | 0.259859499 |
| endo-siRNA | URS000018253F | RNA802 | 31 | 9 | -1.193156187 | 0.259859499 |
| endo-siRNA | URS000050E84A | RNA995 | 79 | 27 | -0.964174782 | 0.260787802 |
| endo-siRNA | URS00002E9B35 | RNA4170 | 116 | 42 | -0.881978236 | 0.261343905 |
| endo-siRNA | URS000047A4E3 | RNA13604 | 20 | 5 | -1.400055427 | 0.266019627 |
| endo-siRNA | URS00002D541C | RNA9034 | 20 | 5 | -1.400055427 | 0.266019627 |
| endo-siRNA | URS000021DBC9 | RNA9273 | 8 | 14 | 1.372969552 | 0.266019627 |
| endo-siRNA | URS000005C052 | RNA9380 | 8 | 14 | 1.372969552 | 0.266019627 |
| endo-siRNA | URS0000615A2B | RNA11267 | 37 | 11 | -1.160669281 | 0.266019627 |
| endo-siRNA | URS00003BA876 | RNA10430 | 70 | 24 | -0.959287178 | 0.266019627 |
| endo-siRNA | URS0000556795 | RNA3409 | 70 | 24 | -0.959287178 | 0.266019627 |
| endo-siRNA | URS000042B008 | RNA1343 | 263 | 102 | -0.783786958 | 0.268517922 |
| endo-siRNA | URS00002B774D | RNA845 | 29 | 8 | -1.265361848 | 0.269329084 |
| endo-siRNA | URS00000FFCCA | RNA1972 | 13 | 20 | 1.194267458 | 0.269329084 |
| endo-siRNA | URS00003D3365 | RNA9895 | 261 | 102 | -0.772780261 | 0.273889822 |
| endo-siRNA | URS00000C0733 | RNA4686 | 171 | 65 | -0.812414972 | 0.273889822 |
| endo-siRNA | URS00003B7040 | RNA11999 | 6 | 13 | 1.673062599 | 0.273889822 |
| endo-siRNA | URS000015701A | RNA14331 | 6 | 13 | 1.673062599 | 0.273889822 |
| endo-siRNA | URS0000522F69 | RNA7203 | 18 | 4 | -1.564109362 | 0.273889822 |
| endo-siRNA | URS000026CE12 | RNA6435 | 18 | 4 | -1.564109362 | 0.273889822 |
| endo-siRNA | URS0000342CF6 | RNA1009 | 18 | 4 | -1.564109362 | 0.273889822 |
| endo-siRNA | URS000039B29C | RNA4513 | 18 | 4 | -1.564109362 | 0.273889822 |
| endo-siRNA | URS000028C1AE | RNA5188 | 18 | 4 | -1.564109362 | 0.273889822 |
| endo-siRNA | URS00005AC523 | RNA864 | 18 | 4 | -1.564109362 | 0.273889822 |
| endo-siRNA | URS00001A49B2 | RNA1292 | 7 | 13 | 1.455701879 | 0.273889822 |
| endo-siRNA | URS0000153692 | RNA6158 | 7 | 13 | 1.455701879 | 0.273889822 |
| endo-siRNA | URS0000300819 | RNA14211 | 35 | 10 | -1.217034356 | 0.273889822 |
| endo-siRNA | URS00001C1A84 | RNA5583 | 16 | 24 | 1.159629911 | 0.273889822 |
| endo-siRNA | URS000006EC13 | RNA7612 | 291 | 114 | -0.769348495 | 0.276307809 |
| endo-siRNA | URS000011338E | RNA4675 | 20 | 28 | 1.061918109 | 0.276884152 |
| endo-siRNA | URS00005D8292 | RNA5633 | 39 | 49 | 0.908831814 | 0.276884152 |
| endo-siRNA | URS00001702C2 | RNA9403 | 134 | 50 | -0.838846612 | 0.276884152 |
| endo-siRNA | URS00005D1EA7 | RNA12679 | 288 | 113 | -0.767105739 | 0.276884152 |
| endo-siRNA | URS0000522D55 | RNA5977 | 27 | 7 | -1.352917641 | 0.276884152 |
| endo-siRNA | URS00001CC42C | RNA880 | 27 | 7 | -1.352917641 | 0.276884152 |
| endo-siRNA | URS0000002AAE | RNA12632 | 27 | 7 | -1.352917641 | 0.276884152 |
| endo-siRNA | URS000005FB1C | RNA13869 | 26 | 7 | -1.298776149 | 0.276884152 |
| endo-siRNA | URS0000479AB7 | RNA12370 | 26 | 7 | -1.298776149 | 0.276884152 |
| endo-siRNA | URS000025655A | RNA9185 | 26 | 7 | -1.298776149 | 0.276884152 |
| endo-siRNA | URS00003E377C | RNA10447 | 336 | 133 | -0.754479538 | 0.277422267 |
| endo-siRNA | URS0000050D09 | RNA1260 | 144 | 54 | -0.831752744 | 0.278233413 |
| endo-siRNA | URS00003901F3 | RNA10264 | 116 | 43 | -0.848110694 | 0.279219595 |
| endo-siRNA | URS000051A9E0 | RNA4684 | 175 | 67 | -0.802089545 | 0.279219595 |
| endo-siRNA | URS00002526E1 | RNA11795 | 16 | 3 | -1.799015053 | 0.279219595 |
| endo-siRNA | URS00002A5CD5 | RNA7792 | 16 | 3 | -1.799015053 | 0.279219595 |
| endo-siRNA | URS00000909FD | RNA8257 | 16 | 3 | -1.799015053 | 0.279219595 |
| endo-siRNA | URS00005ED0D1 | RNA8908 | 16 | 3 | -1.799015053 | 0.279219595 |
| endo-siRNA | URS000004BD3A | RNA12428 | 16 | 3 | -1.799015053 | 0.279219595 |
| endo-siRNA | URS000014BA79 | RNA882 | 16 | 3 | -1.799015053 | 0.279219595 |
| endo-siRNA | URS000055FC60 | RNA3459 | 15 | 3 | -1.706797983 | 0.279219595 |
| endo-siRNA | URS00004050B0 | RNA9912 | 15 | 3 | -1.706797983 | 0.279219595 |
| endo-siRNA | URS0000599EA0 | RNA126 | 15 | 3 | -1.706797983 | 0.279219595 |
| endo-siRNA | URS000050005F | RNA5927 | 15 | 3 | -1.706797983 | 0.279219595 |
| endo-siRNA | URS0000225331 | RNA13616 | 15 | 3 | -1.706797983 | 0.279219595 |
| endo-siRNA | URS00001BCF47 | RNA14379 | 5 | 11 | 1.690077192 | 0.279219595 |
| endo-siRNA | URS0000395192 | RNA817 | 1060 | 432 | -0.712717466 | 0.285109948 |
| endo-siRNA | URS00004C4B40 | RNA7799 | 419 | 168 | -0.736032233 | 0.285109948 |
| endo-siRNA | URS00003F670F | RNA9661 | 203 | 79 | -0.778679515 | 0.285562636 |
| endo-siRNA | URS000020919B | RNA14042 | 6963 | 2866 | -0.698537824 | 0.286576053 |
| endo-siRNA | URS0000570261 | RNA4743 | 10 | 17 | 1.33465669 | 0.287755781 |
| endo-siRNA | URS00002F5C19 | RNA6646 | 10 | 17 | 1.33465669 | 0.287755781 |
| endo-siRNA | URS00002AE038 | RNA9511 | 10 | 17 | 1.33465669 | 0.287755781 |
| endo-siRNA | URS000007C47D | RNA9876 | 289 | 115 | -0.746814945 | 0.287755781 |
| endo-siRNA | URS00001849EA | RNA5882 | 13 | 2 | -2.064393896 | 0.287809929 |
| endo-siRNA | URS0000200A98 | RNA13613 | 13 | 2 | -2.064393896 | 0.287809929 |
| endo-siRNA | URS00001D980F | RNA506 | 13 | 2 | -2.064393896 | 0.287809929 |
| endo-siRNA | URS00003BF66D | RNA11976 | 13 | 2 | -2.064393896 | 0.287809929 |
| endo-siRNA | URS000030B0FF | RNA9817 | 650 | 264 | -0.717574151 | 0.290644664 |
| endo-siRNA | URS00003E9B64 | RNA10421 | 265 | 105 | -0.752930561 | 0.290644664 |
| endo-siRNA | URS00005DDA8B | RNA4167 | 52 | 63 | 0.857091988 | 0.290850579 |
| endo-siRNA | URS000059E679 | RNA9746 | 175 | 68 | -0.780747551 | 0.290850579 |
| endo-siRNA | URS0000583657 | RNA9666 | 1326 | 545 | -0.700537452 | 0.290850579 |
| endo-siRNA | URS00004F33D2 | RNA3143 | 14 | 21 | 1.15857438 | 0.290850579 |
| endo-siRNA | URS00001DE21F | RNA10662 | 14 | 21 | 1.15857438 | 0.290850579 |
| endo-siRNA | URS00005162D3 | RNA6696 | 11 | 1 | -2.759184263 | 0.290850579 |
| endo-siRNA | URS00004140CD | RNA9286 | 11 | 1 | -2.759184263 | 0.290850579 |
| endo-siRNA | URS0000111458 | RNA4535 | 11 | 1 | -2.759184263 | 0.290850579 |
| endo-siRNA | URS0000026DE8 | RNA1002 | 11 | 1 | -2.759184263 | 0.290850579 |
| endo-siRNA | URS0000342BA4 | RNA4540 | 25 | 33 | 0.978398028 | 0.290850579 |
| endo-siRNA | URS0000109F46 | RNA5316 | 36 | 11 | -1.12130259 | 0.291664225 |
| endo-siRNA | URS0000298901 | RNA8676 | 36 | 11 | -1.12130259 | 0.291664225 |
| endo-siRNA | URS00003388E9 | RNA8305 | 36 | 11 | -1.12130259 | 0.291664225 |
| endo-siRNA | URS000035A797 | RNA9625 | 167 | 65 | -0.77829701 | 0.295627006 |
| endo-siRNA | URS0000619756 | RNA10411 | 58 | 20 | -0.950458996 | 0.296762377 |
| endo-siRNA | URS0000086B01 | RNA14295 | 22 | 5 | -1.536583054 | 0.297359358 |
| endo-siRNA | URS00004B7AD9 | RNA5185 | 22 | 5 | -1.536583054 | 0.297359358 |
| endo-siRNA | URS0000278278 | RNA5238 | 22 | 5 | -1.536583054 | 0.297359358 |
| endo-siRNA | URS0000246ACD | RNA9880 | 22 | 5 | -1.536583054 | 0.297359358 |
| endo-siRNA | URS0000116DF9 | RNA5057 | 9 | 15 | 1.304848159 | 0.297359358 |
| endo-siRNA | URS000000EE45 | RNA6868 | 9 | 15 | 1.304848159 | 0.297359358 |
| endo-siRNA | URS00003D655E | RNA814 | 390 | 158 | -0.721076766 | 0.301535819 |
| endo-siRNA | URS000061424E | RNA9043 | 78 | 28 | -0.893554223 | 0.301535819 |
| endo-siRNA | URS00000A90F8 | RNA11966 | 40 | 13 | -1.033702618 | 0.303648806 |
| endo-siRNA | URS0000462896 | RNA591 | 34 | 10 | -1.175395102 | 0.304249511 |
| endo-siRNA | URS000043D38F | RNA9493 | 16 | 23 | 1.098489923 | 0.304249511 |
| endo-siRNA | URS00004DCEA7 | RNA8273 | 30 | 38 | 0.919756401 | 0.305537401 |
| endo-siRNA | URS0000260EF2 | RNA13075 | 987 | 409 | -0.688701948 | 0.307211841 |
| endo-siRNA | URS000000C10F | RNA10589 | 343 | 139 | -0.720601961 | 0.307211841 |
| endo-siRNA | URS00004C83C2 | RNA7709 | 120 | 46 | -0.799879614 | 0.307211841 |
| endo-siRNA | URS00004A8E0D | RNA812 | 663 | 273 | -0.69779179 | 0.308257152 |
| endo-siRNA | URS000007F34A | RNA14033 | 28150 | 11807 | -0.671374682 | 0.30849418 |
| endo-siRNA | URS00002F0CA5 | RNA5582 | 26 | 34 | 0.965085592 | 0.308719435 |
| endo-siRNA | URS000008A04F | RNA5291 | 75 | 27 | -0.889358421 | 0.308719435 |
| endo-siRNA | URS00001B2725 | RNA714 | 187 | 205 | 0.714247438 | 0.310939097 |
| endo-siRNA | URS0000496FEF | RNA9701 | 87 | 99 | 0.767501327 | 0.311004266 |
| endo-siRNA | URS00000AF1A8 | RNA5962 | 19 | 5 | -1.326619538 | 0.311004266 |
| endo-siRNA | URS00000148B3 | RNA5391 | 19 | 5 | -1.326619538 | 0.311004266 |
| endo-siRNA | URS00001EA500 | RNA9654 | 19 | 5 | -1.326619538 | 0.311004266 |
| endo-siRNA | URS000032AD08 | RNA925 | 19 | 5 | -1.326619538 | 0.311004266 |
| endo-siRNA | URS000020C9C8 | RNA8499 | 69 | 25 | -0.879919356 | 0.311206068 |
| endo-siRNA | URS000046EE58 | RNA14045 | 277 | 112 | -0.723765341 | 0.313996364 |
| endo-siRNA | URS00001D5B12 | RNA5303 | 178 | 71 | -0.743054407 | 0.314653577 |
| endo-siRNA | URS00001019DE | RNA9597 | 677 | 723 | 0.676833462 | 0.315158073 |
| endo-siRNA | URS0000485549 | RNA5590 | 15 | 22 | 1.126860846 | 0.316755221 |
| endo-siRNA | URS000000D11C | RNA4795 | 25 | 7 | -1.242523342 | 0.316919334 |
| endo-siRNA | URS000027D53D | RNA9482 | 161 | 176 | 0.710105999 | 0.320193102 |
| endo-siRNA | URS00000DCAE2 | RNA11955 | 31 | 39 | 0.910059553 | 0.320193102 |
| endo-siRNA | URS000023EADA | RNA14015 | 131 | 145 | 0.727945441 | 0.32073773 |
| endo-siRNA | URS000016AF03 | RNA7800 | 366 | 151 | -0.69481629 | 0.321448468 |
| endo-siRNA | URS000008E5E8 | RNA10448 | 651 | 272 | -0.676738829 | 0.322899412 |
| endo-siRNA | URS000005352D | RNA872 | 104 | 40 | -0.794869545 | 0.324874745 |
| endo-siRNA | URS0000255432 | RNA5255 | 6 | 12 | 1.558503913 | 0.327037583 |
| endo-siRNA | URS00002C70E5 | RNA3254 | 6 | 12 | 1.558503913 | 0.327037583 |
| endo-siRNA | URS000005E403 | RNA10271 | 17 | 4 | -1.482347841 | 0.327037583 |
| endo-siRNA | URS00000AAE2B | RNA2219 | 17 | 4 | -1.482347841 | 0.327037583 |
| endo-siRNA | URS00000215B5 | RNA923 | 17 | 4 | -1.482347841 | 0.327037583 |
| endo-siRNA | URS00001ACA60 | RNA11276 | 17 | 4 | -1.482347841 | 0.327037583 |
| endo-siRNA | URS00001038FC | RNA9187 | 17 | 4 | -1.482347841 | 0.327037583 |
| endo-siRNA | URS000056DBCB | RNA5196 | 7 | 12 | 1.341143193 | 0.327037583 |
| endo-siRNA | URS0000228801 | RNA9636 | 35 | 11 | -1.080831497 | 0.327189322 |
| endo-siRNA | URS000004C759 | RNA10013 | 35 | 11 | -1.080831497 | 0.327189322 |
| endo-siRNA | URS0000473B59 | RNA12665 | 35 | 11 | -1.080831497 | 0.327189322 |
| endo-siRNA | URS000021B764 | RNA1825 | 42 | 50 | 0.831398688 | 0.327225992 |
| endo-siRNA | URS00003A77CA | RNA3118 | 30 | 37 | 0.881384711 | 0.329599815 |
| endo-siRNA | URS00001B86F5 | RNA12319 | 45 | 15 | -0.998049081 | 0.329599815 |
| endo-siRNA | URS00004255FA | RNA9839 | 45 | 15 | -0.998049081 | 0.329599815 |
| endo-siRNA | URS0000614434 | RNA4233 | 45 | 15 | -0.998049081 | 0.329599815 |
| endo-siRNA | URS0000319E7C | RNA991 | 29 | 9 | -1.097419531 | 0.330699499 |
| endo-siRNA | URS00004871D2 | RNA6678 | 14 | 20 | 1.088527286 | 0.330699499 |
| endo-siRNA | URS000012AACD | RNA6077 | 172 | 69 | -0.734791851 | 0.331594841 |
| endo-siRNA | URS00004403EA | RNA3527 | 23 | 6 | -1.341982382 | 0.331755755 |
| endo-siRNA | URS000039FEFA | RNA12180 | 23 | 6 | -1.341982382 | 0.331755755 |
| endo-siRNA | URS000034D7B9 | RNA9807 | 10 | 16 | 1.247721674 | 0.331755755 |
| endo-siRNA | URS0000271E32 | RNA9896 | 2483 | 1058 | -0.648581955 | 0.33526144 |
| endo-siRNA | URS000008374C | RNA1007 | 39 | 13 | -0.997314829 | 0.33526144 |
| endo-siRNA | URS00002E7C01 | RNA3410 | 49 | 17 | -0.941068367 | 0.33526144 |
| endo-siRNA | URS000003C272 | RNA9793 | 143 | 57 | -0.743847584 | 0.336776339 |
| endo-siRNA | URS000008F62A | RNA9688 | 35 | 42 | 0.842417717 | 0.338485059 |
| endo-siRNA | URS000015DB73 | RNA790 | 194 | 79 | -0.713305818 | 0.338485059 |
| endo-siRNA | URS00003A2530 | RNA10135 | 123 | 49 | -0.744503581 | 0.342533373 |
| endo-siRNA | URS0000377A91 | RNA6427 | 33 | 10 | -1.132518243 | 0.342533373 |
| endo-siRNA | URS000033BC6A | RNA9662 | 2882 | 1235 | -0.640404123 | 0.342935389 |
| endo-siRNA | URS0000188000 | RNA12118 | 14 | 3 | -1.608281445 | 0.342935389 |
| endo-siRNA | URS00004D37FF | RNA12139 | 14 | 3 | -1.608281445 | 0.342935389 |
| endo-siRNA | URS000030A41B | RNA10991 | 14 | 3 | -1.608281445 | 0.342935389 |
| endo-siRNA | URS000002B34C | RNA9613 | 14 | 3 | -1.608281445 | 0.342935389 |
| endo-siRNA | URS0000009BA5 | RNA6773 | 14 | 3 | -1.608281445 | 0.342935389 |
| endo-siRNA | URS00001C01F8 | RNA7040 | 5 | 10 | 1.553874334 | 0.342935389 |
| endo-siRNA | URS00003E7BAA | RNA6081 | 216 | 89 | -0.696419543 | 0.344768361 |
| endo-siRNA | URS0000269D0E | RNA14240 | 56 | 20 | -0.899965723 | 0.345415245 |
| endo-siRNA | URS0000622856 | RNA6074 | 313 | 130 | -0.68511781 | 0.345833077 |
| endo-siRNA | URS000013103C | RNA8563 | 26 | 8 | -1.108676238 | 0.347297816 |
| endo-siRNA | URS0000216829 | RNA3417 | 46 | 16 | -0.937142387 | 0.347780893 |
| endo-siRNA | URS000054527A | RNA4063 | 255 | 106 | -0.683805457 | 0.348453433 |
| endo-siRNA | URS00001E24D3 | RNA14157 | 75 | 84 | 0.744450251 | 0.348612112 |
| endo-siRNA | URS00000975DC | RNA9822 | 581 | 246 | -0.657551691 | 0.348978815 |
| endo-siRNA | URS0000268ED2 | RNA8889 | 21 | 5 | -1.469933653 | 0.349032931 |
| endo-siRNA | URS0000453619 | RNA8238 | 21 | 5 | -1.469933653 | 0.349032931 |
| endo-siRNA | URS00001CA93E | RNA5200 | 21 | 5 | -1.469933653 | 0.349032931 |
| endo-siRNA | URS000037715C | RNA843 | 21 | 5 | -1.469933653 | 0.349032931 |
| endo-siRNA | URS00002ADC0D | RNA2244 | 21 | 5 | -1.469933653 | 0.349032931 |
| endo-siRNA | URS000014537F | RNA10119 | 36 | 43 | 0.835814166 | 0.350576759 |
| endo-siRNA | URS00001B8AFF | RNA2525 | 50 | 18 | -0.8881339 | 0.3511815 |
| endo-siRNA | URS000026244B | RNA6041 | 456 | 193 | -0.658045646 | 0.354220078 |
| endo-siRNA | URS00000D7B8B | RNA14043 | 7606 | 3295 | -0.624730821 | 0.355108129 |
| endo-siRNA | URS00001248E0 | RNA9599 | 68 | 76 | 0.741300799 | 0.359388429 |
| endo-siRNA | URS00004F16B0 | RNA9756 | 161 | 66 | -0.703565117 | 0.359869197 |
| endo-siRNA | URS0000220FDA | RNA3384 | 186 | 76 | -0.708380845 | 0.359869197 |
| endo-siRNA | URS0000398DA8 | RNA9747 | 185 | 76 | -0.700609774 | 0.359869197 |
| endo-siRNA | URS000017E85E | RNA9723 | 57 | 21 | -0.855386157 | 0.359869197 |
| endo-siRNA | URS0000605EEE | RNA14267 | 12 | 2 | -1.95028634 | 0.359869197 |
| endo-siRNA | URS000014494A | RNA504 | 12 | 2 | -1.95028634 | 0.359869197 |
| endo-siRNA | URS0000321E3F | RNA2298 | 12 | 2 | -1.95028634 | 0.359869197 |
| endo-siRNA | URS000011B0CD | RNA9728 | 12 | 2 | -1.95028634 | 0.359869197 |
| endo-siRNA | URS0000381BBC | RNA19 | 12 | 2 | -1.95028634 | 0.359869197 |
| endo-siRNA | URS00004EC226 | RNA6476 | 24 | 30 | 0.899866306 | 0.359897912 |
| endo-siRNA | URS000050E69B | RNA11415 | 432 | 184 | -0.648927963 | 0.362798076 |
| endo-siRNA | URS00004FE6A0 | RNA10263 | 64 | 24 | -0.830293112 | 0.363936452 |
| endo-siRNA | URS0000239A3F | RNA3486 | 34 | 11 | -1.039192244 | 0.363936452 |
| endo-siRNA | URS0000500B2E | RNA7559 | 24 | 7 | -1.18398785 | 0.363936452 |
| endo-siRNA | URS00005A0987 | RNA12653 | 24 | 7 | -1.18398785 | 0.363936452 |
| endo-siRNA | URS000009A7D1 | RNA5076 | 24 | 7 | -1.18398785 | 0.363936452 |
| endo-siRNA | URS0000038926 | RNA807 | 68 | 26 | -0.802541543 | 0.366338922 |
| endo-siRNA | URS00003F1E01 | RNA3393 | 29 | 35 | 0.850099131 | 0.367697195 |
| endo-siRNA | URS000022D3D7 | RNA11529 | 18 | 5 | -1.249244202 | 0.368708004 |
| endo-siRNA | URS000007BD6D | RNA13530 | 18 | 5 | -1.249244202 | 0.368708004 |
| endo-siRNA | URS00002394F1 | RNA8906 | 18 | 5 | -1.249244202 | 0.368708004 |
| endo-siRNA | URS00005F6728 | RNA6672 | 18 | 5 | -1.249244202 | 0.368708004 |
| endo-siRNA | URS00003582F7 | RNA9527 | 18 | 5 | -1.249244202 | 0.368708004 |
| endo-siRNA | URS000048B109 | RNA7467 | 18 | 5 | -1.249244202 | 0.368708004 |
| endo-siRNA | URS0000178BD0 | RNA6487 | 38 | 13 | -0.959985464 | 0.369233899 |
| endo-siRNA | URS000059238B | RNA208 | 55 | 20 | -0.87404053 | 0.369536088 |
| endo-siRNA | URS0000113347 | RNA11297 | 245 | 103 | -0.667504826 | 0.369813493 |
| endo-siRNA | URS000046642C | RNA13923 | 178 | 74 | -0.683430522 | 0.371001691 |
| endo-siRNA | URS000015EDC8 | RNA12019 | 86 | 34 | -0.754958811 | 0.373357316 |
| endo-siRNA | URS00000F22A1 | RNA5997 | 328 | 140 | -0.645783489 | 0.373357316 |
| endo-siRNA | URS00004B8DB3 | RNA753 | 14 | 19 | 1.014904868 | 0.373357316 |
| endo-siRNA | URS000055EB76 | RNA11819 | 41 | 14 | -0.963067739 | 0.373357316 |
| endo-siRNA | URS0000005E8E | RNA9730 | 272 | 116 | -0.646918716 | 0.375508495 |
| endo-siRNA | URS00003C7B9B | RNA3457 | 66 | 25 | -0.815931149 | 0.37553472 |
| endo-siRNA | URS00003D4242 | RNA7364 | 66 | 25 | -0.815931149 | 0.37553472 |
| endo-siRNA | URS0000019728 | RNA5300 | 97 | 39 | -0.730926009 | 0.37553472 |
| endo-siRNA | URS00001537FD | RNA1235 | 77 | 30 | -0.775781526 | 0.380590484 |
| endo-siRNA | URS00005AA036 | RNA5572 | 46 | 52 | 0.757072958 | 0.380590484 |
| endo-siRNA | URS00002F6A2E | RNA8581 | 27 | 33 | 0.868003729 | 0.380590484 |
| endo-siRNA | URS00006086F7 | RNA498 | 48 | 18 | -0.829419856 | 0.380590484 |
| endo-siRNA | URS00002A41AE | RNA884 | 32 | 10 | -1.08832795 | 0.380636066 |
| endo-siRNA | URS0000087C21 | RNA14066 | 31 | 10 | -1.042741205 | 0.380636066 |
| endo-siRNA | URS00001C8F5B | RNA2737 | 16 | 22 | 1.034643776 | 0.380636066 |
| endo-siRNA | URS0000429DFC | RNA14041 | 7150 | 3154 | -0.598631782 | 0.380664365 |
| endo-siRNA | URS000040AA23 | RNA4236 | 220 | 228 | 0.633294375 | 0.381649922 |
| endo-siRNA | URS0000419612 | RNA9908 | 52 | 19 | -0.866969137 | 0.382269873 |
| endo-siRNA | URS000015EAAA | RNA4285 | 22 | 6 | -1.278276554 | 0.382842159 |
| endo-siRNA | URS000058377F | RNA4141 | 10 | 15 | 1.155210239 | 0.382842159 |
| endo-siRNA | URS00004A258E | RNA5243 | 10 | 15 | 1.155210239 | 0.382842159 |
| endo-siRNA | URS00005173DC | RNA3652 | 63 | 69 | 0.712021405 | 0.384225331 |
| endo-siRNA | URS00003184CC | RNA4240 | 35 | 12 | -0.956385399 | 0.384475739 |
| endo-siRNA | URS00002359A1 | RNA4394 | 418 | 181 | -0.625119417 | 0.384837555 |
| endo-siRNA | URS00005C2863 | RNA5205 | 124 | 51 | -0.698571095 | 0.387016748 |
| endo-siRNA | URS000007CDDA | RNA12880 | 132 | 54 | -0.706358225 | 0.387551729 |
| endo-siRNA | URS000043C3D7 | RNA3495 | 71 | 27 | -0.810448946 | 0.387946147 |
| endo-siRNA | URS000036A182 | RNA9783 | 658 | 288 | -0.609732668 | 0.387946147 |
| endo-siRNA | URS00001CC954 | RNA5705 | 6 | 11 | 1.434057815 | 0.387946147 |
| endo-siRNA | URS00003628CE | RNA3154 | 6 | 11 | 1.434057815 | 0.387946147 |
| endo-siRNA | URS0000455AEE | RNA5068 | 16 | 4 | -1.395672812 | 0.387946147 |
| endo-siRNA | URS0000214E1B | RNA943 | 16 | 4 | -1.395672812 | 0.387946147 |
| endo-siRNA | URS00003998D7 | RNA5235 | 16 | 4 | -1.395672812 | 0.387946147 |
| endo-siRNA | URS000045B750 | RNA5761 | 7 | 11 | 1.216697095 | 0.387946147 |
| endo-siRNA | URS0000003EE6 | RNA5093 | 7 | 11 | 1.216697095 | 0.387946147 |
| endo-siRNA | URS000060E39D | RNA13941 | 43 | 15 | -0.932683477 | 0.388811075 |
| endo-siRNA | URS00002A1309 | RNA14337 | 24 | 29 | 0.851122167 | 0.388811075 |
| endo-siRNA | URS00003CE3F9 | RNA10668 | 25 | 8 | -1.052423432 | 0.389923786 |
| endo-siRNA | URS000007405F | RNA5733 | 25 | 8 | -1.052423432 | 0.389923786 |
| endo-siRNA | URS000026B662 | RNA4547 | 71 | 77 | 0.697984971 | 0.390376894 |
| endo-siRNA | URS00000AB704 | RNA9646 | 122 | 50 | -0.703653296 | 0.390704799 |
| endo-siRNA | URS00005CC30E | RNA11953 | 28 | 34 | 0.858761152 | 0.392131276 |
| endo-siRNA | URS00002A3633 | RNA4127 | 61 | 23 | -0.822336202 | 0.396499693 |
| endo-siRNA | URS00004278F8 | RNA14049 | 411 | 179 | -0.616784874 | 0.396733001 |
| endo-siRNA | URS000039ECAF | RNA13857 | 83 | 33 | -0.746764578 | 0.398995425 |
| endo-siRNA | URS00001092A8 | RNA810 | 671 | 296 | -0.598436671 | 0.398995425 |
| endo-siRNA | URS00003FD077 | RNA5175 | 116 | 48 | -0.689762081 | 0.398995425 |
| endo-siRNA | URS00005BDD00 | RNA821 | 264 | 114 | -0.628943226 | 0.399608504 |
| endo-siRNA | URS0000040BCD | RNA11921 | 205 | 88 | -0.637349209 | 0.401981691 |
| endo-siRNA | URS000027B260 | RNA499 | 33 | 11 | -0.996315385 | 0.401981691 |
| endo-siRNA | URS0000588682 | RNA9555 | 18 | 22 | 0.866207226 | 0.401981691 |
| endo-siRNA | URS000028E883 | RNA14036 | 13123 | 5887 | -0.574371729 | 0.404228178 |
| endo-siRNA | URS00000399FB | RNA3360 | 20 | 25 | 0.89903607 | 0.404529922 |
| endo-siRNA | URS000059B033 | RNA4152 | 36 | 13 | -0.882297806 | 0.404529922 |
| endo-siRNA | URS0000271EAD | RNA10428 | 36 | 13 | -0.882297806 | 0.404529922 |
| endo-siRNA | URS000047CA76 | RNA12215 | 9 | 13 | 1.09986815 | 0.404579697 |
| endo-siRNA | URS000049B5BE | RNA13072 | 9 | 13 | 1.09986815 | 0.404579697 |
| endo-siRNA | URS0000608636 | RNA9565 | 9 | 13 | 1.09986815 | 0.404579697 |
| endo-siRNA | URS0000389B55 | RNA4159 | 9 | 13 | 1.09986815 | 0.404579697 |
| endo-siRNA | URS00002D96A8 | RNA11701 | 40 | 14 | -0.927575193 | 0.406069308 |
| endo-siRNA | URS000054B7E1 | RNA5995 | 22 | 27 | 0.873113429 | 0.406069308 |
| endo-siRNA | URS0000423E60 | RNA4393 | 192 | 82 | -0.64466263 | 0.406383596 |
| endo-siRNA | URS0000226985 | RNA9632 | 44 | 16 | -0.87322501 | 0.407233665 |
| endo-siRNA | URS000051631F | RNA5210 | 44 | 16 | -0.87322501 | 0.407233665 |
| endo-siRNA | URS0000169854 | RNA3500 | 84 | 34 | -0.721071239 | 0.408099279 |
| endo-siRNA | URS000023ED1A | RNA2501 | 30 | 35 | 0.801436818 | 0.408298536 |
| endo-siRNA | URS0000368020 | RNA438 | 55 | 21 | -0.803993436 | 0.408298536 |
| endo-siRNA | URS00005C230A | RNA9602 | 42 | 47 | 0.742315362 | 0.408298536 |
| endo-siRNA | URS00002ADA9F | RNA3424 | 62 | 24 | -0.784598152 | 0.408298536 |
| endo-siRNA | URS000015CCF6 | RNA9659 | 213 | 92 | -0.628480089 | 0.412434152 |
| endo-siRNA | URS000040762E | RNA3418 | 23 | 7 | -1.122976548 | 0.412434152 |
| endo-siRNA | URS00001A4A83 | RNA9584 | 23 | 7 | -1.122976548 | 0.412434152 |
| endo-siRNA | URS000052D80F | RNA5596 | 13 | 3 | -1.502541273 | 0.412434152 |
| endo-siRNA | URS00000D27B8 | RNA5571 | 13 | 3 | -1.502541273 | 0.412434152 |
| endo-siRNA | URS0000056234 | RNA12375 | 13 | 3 | -1.502541273 | 0.412434152 |
| endo-siRNA | URS00000703B7 | RNA9539 | 13 | 3 | -1.502541273 | 0.412434152 |
| endo-siRNA | URS00003BFB93 | RNA5130 | 13 | 3 | -1.502541273 | 0.412434152 |
| endo-siRNA | URS0000308073 | RNA9322 | 13 | 3 | -1.502541273 | 0.412434152 |
| endo-siRNA | URS000022F3F3 | RNA1191 | 13 | 3 | -1.502541273 | 0.412434152 |
| endo-siRNA | URS00002C2D62 | RNA14356 | 27 | 9 | -0.994875413 | 0.417514205 |
| endo-siRNA | URS00001FA311 | RNA11782 | 27 | 9 | -0.994875413 | 0.417514205 |
| endo-siRNA | URS0000258CD0 | RNA10731 | 27 | 9 | -0.994875413 | 0.417514205 |
| endo-siRNA | URS00005DDBDA | RNA13946 | 27 | 9 | -0.994875413 | 0.417514205 |
| endo-siRNA | URS00000AE501 | RNA11397 | 26 | 9 | -0.940733921 | 0.417514205 |
| endo-siRNA | URS000052668E | RNA14046 | 656 | 294 | -0.575604789 | 0.419255878 |
| endo-siRNA | URS000006CD1D | RNA7381 | 1768 | 799 | -0.563679153 | 0.421517261 |
| endo-siRNA | URS00001601FF | RNA10476 | 857 | 385 | -0.572200683 | 0.421749658 |
| endo-siRNA | URS000023DA21 | RNA841 | 34 | 12 | -0.914746146 | 0.423073637 |
| endo-siRNA | URS00005397F7 | RNA5292 | 292 | 129 | -0.596105132 | 0.423073637 |
| endo-siRNA | URS00004C66AB | RNA5932 | 52 | 20 | -0.793346719 | 0.423197826 |
| endo-siRNA | URS00004FD53A | RNA9306 | 26 | 31 | 0.832228917 | 0.423847147 |
| endo-siRNA | URS00003F69F6 | RNA8497 | 26 | 31 | 0.832228917 | 0.423847147 |
| endo-siRNA | URS00000B1B63 | RNA8827 | 41 | 15 | -0.864215156 | 0.423847147 |
| endo-siRNA | URS00003FBCE2 | RNA6803 | 57 | 61 | 0.678537919 | 0.42642747 |
| endo-siRNA | URS00005E2F02 | RNA14037 | 14556 | 6631 | -0.552195422 | 0.427371888 |
| endo-siRNA | URS0000459A93 | RNA4330 | 79 | 32 | -0.719895319 | 0.430141782 |
| endo-siRNA | URS000020121B | RNA979 | 89 | 92 | 0.62908338 | 0.430277392 |
| endo-siRNA | URS0000096F5F | RNA9667 | 1663 | 756 | -0.555155756 | 0.430277392 |
| endo-siRNA | URS00005F56B6 | RNA13985 | 349 | 156 | -0.579261604 | 0.430277392 |
| endo-siRNA | URS00001284A3 | RNA3155 | 17 | 5 | -1.167482681 | 0.430277392 |
| endo-siRNA | URS00001256FC | RNA8566 | 17 | 5 | -1.167482681 | 0.430277392 |
| endo-siRNA | URS00004EAF38 | RNA3531 | 17 | 5 | -1.167482681 | 0.430277392 |
| endo-siRNA | URS00003478CF | RNA14281 | 17 | 5 | -1.167482681 | 0.430277392 |
| endo-siRNA | URS0000593734 | RNA11112 | 8 | 12 | 1.152283441 | 0.430277392 |
| endo-siRNA | URS000002D8F1 | RNA690 | 8 | 12 | 1.152283441 | 0.430277392 |
| endo-siRNA | URS0000437FF8 | RNA12457 | 8 | 12 | 1.152283441 | 0.430277392 |
| endo-siRNA | URS0000261348 | RNA4506 | 8 | 12 | 1.152283441 | 0.430277392 |
| endo-siRNA | URS00004D27AB | RNA13578 | 42 | 46 | 0.711355132 | 0.43176238 |
| endo-siRNA | URS00004EE41B | RNA752 | 564 | 254 | -0.568568209 | 0.431898951 |
| endo-siRNA | URS00005A9C7B | RNA6831 | 129 | 131 | 0.603735917 | 0.432319489 |
| endo-siRNA | URS00004DFF3D | RNA1198 | 397 | 178 | -0.57488185 | 0.432698063 |
| endo-siRNA | URS00002D20A9 | RNA993 | 95 | 40 | -0.664482112 | 0.432698063 |
| endo-siRNA | URS00002C3514 | RNA4359 | 40 | 44 | 0.717500051 | 0.432698063 |
| endo-siRNA | URS00005B3485 | RNA6763 | 306 | 136 | -0.587456555 | 0.437438201 |
| endo-siRNA | URS000005076B | RNA549 | 53 | 21 | -0.750702125 | 0.438132042 |
| endo-siRNA | URS00004E3B94 | RNA9792 | 32 | 36 | 0.749303401 | 0.438132042 |
| endo-siRNA | URS00000F4066 | RNA6674 | 30 | 34 | 0.759737669 | 0.439092317 |
| endo-siRNA | URS00003A0A7F | RNA508 | 21 | 6 | -1.211627154 | 0.439092317 |
| endo-siRNA | URS000053D89E | RNA12481 | 20 | 6 | -1.141748928 | 0.439092317 |
| endo-siRNA | URS00002A3226 | RNA14124 | 20 | 6 | -1.141748928 | 0.439092317 |
| endo-siRNA | URS00002A1359 | RNA4695 | 20 | 6 | -1.141748928 | 0.439092317 |
| endo-siRNA | URS0000361C44 | RNA2522 | 10 | 14 | 1.056357655 | 0.439092317 |
| endo-siRNA | URS00001BA383 | RNA9029 | 27 | 31 | 0.778087425 | 0.43937236 |
| endo-siRNA | URS00001A6FA9 | RNA501 | 202 | 89 | -0.599812855 | 0.43937236 |
| endo-siRNA | URS0000202C72 | RNA8097 | 132 | 133 | 0.592449971 | 0.43937236 |
| endo-siRNA | URS00003ADAE1 | RNA5487 | 43 | 16 | -0.840172041 | 0.43937236 |
| endo-siRNA | URS00003B3612 | RNA3117 | 25 | 29 | 0.792586674 | 0.43937236 |
| endo-siRNA | URS000000A603 | RNA7373 | 587 | 266 | -0.559646427 | 0.43937236 |
| endo-siRNA | URS00002A6CEA | RNA9890 | 13 | 17 | 0.961069775 | 0.43937236 |
| endo-siRNA | URS000017160E | RNA9433 | 11 | 2 | -1.826372474 | 0.43937236 |
| endo-siRNA | URS0000031A41 | RNA5564 | 11 | 2 | -1.826372474 | 0.43937236 |
| endo-siRNA | URS00003172BF | RNA3188 | 11 | 2 | -1.826372474 | 0.43937236 |
| endo-siRNA | URS00004F4AE2 | RNA4346 | 11 | 2 | -1.826372474 | 0.43937236 |
| endo-siRNA | URS00004431CB | RNA5899 | 11 | 2 | -1.826372474 | 0.43937236 |
| endo-siRNA | URS000011A145 | RNA9524 | 11 | 2 | -1.826372474 | 0.43937236 |
| endo-siRNA | URS0000322802 | RNA10431 | 11 | 2 | -1.826372474 | 0.43937236 |
| endo-siRNA | URS0000171511 | RNA12348 | 11 | 2 | -1.826372474 | 0.43937236 |
| endo-siRNA | URS00005F8B47 | RNA4362 | 35 | 13 | -0.841826714 | 0.439385524 |
| endo-siRNA | URS00004BBE26 | RNA13069 | 332 | 149 | -0.573439749 | 0.439385524 |
| endo-siRNA | URS00000CA10D | RNA12024 | 32 | 11 | -0.952125092 | 0.439385524 |
| endo-siRNA | URS00001D45E8 | RNA7497 | 32 | 11 | -0.952125092 | 0.439385524 |
| endo-siRNA | URS00001F5761 | RNA1268 | 32 | 11 | -0.952125092 | 0.439385524 |
| endo-siRNA | URS000016792E | RNA1011 | 31 | 11 | -0.906538347 | 0.439385524 |
| endo-siRNA | URS000008EAB4 | RNA840 | 31 | 11 | -0.906538347 | 0.439385524 |
| endo-siRNA | URS00004BC85F | RNA13866 | 108 | 46 | -0.648076473 | 0.439990803 |
| endo-siRNA | URS00001DE973 | RNA14302 | 70 | 73 | 0.641546326 | 0.439990803 |
| endo-siRNA | URS000059DFFF | RNA13117 | 121 | 52 | -0.635321187 | 0.443771918 |
| endo-siRNA | URS0000621DA7 | RNA11813 | 136 | 59 | -0.621849551 | 0.444554185 |
| endo-siRNA | URS000037779C | RNA14048 | 296 | 133 | -0.571702406 | 0.447533026 |
| endo-siRNA | URS0000014D40 | RNA8502 | 71 | 29 | -0.707723078 | 0.452219625 |
| endo-siRNA | URS00001009B9 | RNA4066 | 79 | 33 | -0.675637561 | 0.459074573 |
| endo-siRNA | URS0000567E96 | RNA5298 | 112 | 112 | 0.581472954 | 0.459074573 |
| endo-siRNA | URS00005D32DA | RNA14098 | 1241 | 574 | -0.530189841 | 0.459074573 |
| endo-siRNA | URS000047634C | RNA10630 | 60 | 24 | -0.737408407 | 0.459074573 |
| endo-siRNA | URS000022C93E | RNA2189 | 15 | 4 | -1.303455742 | 0.459074573 |
| endo-siRNA | URS0000143F27 | RNA10422 | 15 | 4 | -1.303455742 | 0.459074573 |
| endo-siRNA | URS000053929F | RNA1741 | 6 | 10 | 1.297854957 | 0.459074573 |
| endo-siRNA | URS000043482D | RNA3550 | 6 | 10 | 1.297854957 | 0.459074573 |
| endo-siRNA | URS000009BDA6 | RNA12579 | 6 | 10 | 1.297854957 | 0.459074573 |
| endo-siRNA | URS00001A4C28 | RNA566 | 14 | 4 | -1.204939204 | 0.459074573 |
| endo-siRNA | URS00004B2CDB | RNA10665 | 14 | 4 | -1.204939204 | 0.459074573 |
| endo-siRNA | URS000000763C | RNA9803 | 14 | 4 | -1.204939204 | 0.459074573 |
| endo-siRNA | URS000034A908 | RNA10832 | 14 | 4 | -1.204939204 | 0.459074573 |
| endo-siRNA | URS0000407F6B | RNA1259 | 14 | 4 | -1.204939204 | 0.459074573 |
| endo-siRNA | URS00001A2C92 | RNA9553 | 1669 | 774 | -0.526408186 | 0.459142218 |
| endo-siRNA | URS00002D70CC | RNA494 | 52 | 21 | -0.723299625 | 0.465318132 |
| endo-siRNA | URS000012B004 | RNA14338 | 52 | 21 | -0.723299625 | 0.465318132 |
| endo-siRNA | URS0000524E6A | RNA9633 | 68 | 28 | -0.696021686 | 0.465318132 |
| endo-siRNA | URS000042E2A2 | RNA7367 | 813 | 376 | -0.53029129 | 0.465940631 |
| endo-siRNA | URS00004E9E45 | RNA574 | 121 | 53 | -0.607892761 | 0.466806978 |
| endo-siRNA | URS00001C3BCE | RNA5818 | 30 | 33 | 0.716797299 | 0.467070817 |
| endo-siRNA | URS0000189387 | RNA35 | 30 | 33 | 0.716797299 | 0.467070817 |
| endo-siRNA | URS0000097426 | RNA9766 | 22 | 7 | -1.05927072 | 0.467070817 |
| endo-siRNA | URS000007F87E | RNA4672 | 22 | 7 | -1.05927072 | 0.467070817 |
| endo-siRNA | URS00002B9CA4 | RNA13902 | 11 | 15 | 1.019644745 | 0.467070817 |
| endo-siRNA | URS0000173536 | RNA14195 | 150 | 67 | -0.57990287 | 0.47043784 |
| endo-siRNA | URS0000478C90 | RNA4792 | 1146 | 535 | -0.516801717 | 0.47548693 |
| endo-siRNA | URS00000B9A19 | RNA5049 | 36 | 39 | 0.69529531 | 0.47548693 |
| endo-siRNA | URS000028AEB9 | RNA9694 | 41 | 16 | -0.77170372 | 0.47622857 |
| endo-siRNA | URS00000BF01E | RNA4324 | 41 | 16 | -0.77170372 | 0.47622857 |
| endo-siRNA | URS00005E945E | RNA2414 | 25 | 28 | 0.742137861 | 0.47622857 |
| endo-siRNA | URS00004A1A66 | RNA9647 | 135 | 60 | -0.587007259 | 0.480651873 |
| endo-siRNA | URS00001ECFA5 | RNA5454 | 50 | 53 | 0.664582036 | 0.481092914 |
| endo-siRNA | URS00004EA389 | RNA11383 | 37 | 14 | -0.815537072 | 0.481092914 |
| endo-siRNA | URS0000578DF2 | RNA10392 | 37 | 14 | -0.815537072 | 0.481092914 |
| endo-siRNA | URS0000048E97 | RNA10046 | 111 | 49 | -0.596594751 | 0.481781258 |
| endo-siRNA | URS00004A197C | RNA33 | 186 | 84 | -0.564171712 | 0.482147778 |
| endo-siRNA | URS00002DA685 | RNA10477 | 1030 | 483 | -0.510346899 | 0.484787854 |
| endo-siRNA | URS00004FAB81 | RNA1852 | 48 | 50 | 0.639395016 | 0.484787854 |
| endo-siRNA | URS0000217255 | RNA8276 | 50 | 20 | -0.736929036 | 0.484787854 |
| endo-siRNA | URS00005A30ED | RNA726 | 34 | 13 | -0.800187461 | 0.485880213 |
| endo-siRNA | URS0000350248 | RNA724 | 83 | 36 | -0.621597387 | 0.487591968 |
| endo-siRNA | URS00004544B2 | RNA11526 | 2430 | 1146 | -0.502198972 | 0.487757865 |
| endo-siRNA | URS00005CCC14 | RNA9732 | 1363 | 641 | -0.506209371 | 0.488649202 |
| endo-siRNA | URS00004F913F | RNA894 | 30 | 11 | -0.859464003 | 0.490848135 |
| endo-siRNA | URS0000585890 | RNA8002 | 18 | 21 | 0.799404222 | 0.490848135 |
| endo-siRNA | URS000032FE8E | RNA4068 | 1480 | 698 | -0.502124164 | 0.49285948 |
| endo-siRNA | URS000005D0B7 | RNA1200 | 717 | 336 | -0.51127037 | 0.493708732 |
| endo-siRNA | URS00000F23A1 | RNA11416 | 96 | 42 | -0.609347538 | 0.494732883 |
| endo-siRNA | URS00002709E7 | RNA5989 | 43 | 17 | -0.753237026 | 0.494732883 |
| endo-siRNA | URS00002CFACC | RNA3372 | 172 | 78 | -0.558155298 | 0.494732883 |
| endo-siRNA | URS00000EE63F | RNA10615 | 134 | 60 | -0.576292792 | 0.494732883 |
| endo-siRNA | URS000028EE1A | RNA2277 | 15 | 18 | 0.838805884 | 0.494824086 |
| endo-siRNA | URS000022B77C | RNA8649 | 15 | 18 | 0.838805884 | 0.494824086 |
| endo-siRNA | URS00001823B4 | RNA1240 | 377 | 175 | -0.524844666 | 0.495336469 |
| endo-siRNA | URS00005DB261 | RNA8274 | 231 | 221 | 0.517984422 | 0.495336469 |
| endo-siRNA | URS000002F04F | RNA7795 | 72 | 31 | -0.6319637 | 0.495336469 |
| endo-siRNA | URS00003C889A | RNA9551 | 182 | 83 | -0.55009037 | 0.495336469 |
| endo-siRNA | URS00003A70DF | RNA850 | 12 | 3 | -1.388433717 | 0.495336469 |
| endo-siRNA | URS00005C3D18 | RNA6561 | 12 | 3 | -1.388433717 | 0.495336469 |
| endo-siRNA | URS00003D23AC | RNA4237 | 12 | 3 | -1.388433717 | 0.495336469 |
| endo-siRNA | URS00000FF30B | RNA12018 | 12 | 3 | -1.388433717 | 0.495336469 |
| endo-siRNA | URS000001A6A0 | RNA6477 | 12 | 3 | -1.388433717 | 0.495336469 |
| endo-siRNA | URS000009C4E6 | RNA828 | 12 | 3 | -1.388433717 | 0.495336469 |
| endo-siRNA | URS00000BC02C | RNA9531 | 12 | 3 | -1.388433717 | 0.495336469 |
| endo-siRNA | URS00003AAD8F | RNA4248 | 12 | 3 | -1.388433717 | 0.495336469 |
| endo-siRNA | URS0000220425 | RNA5988 | 12 | 3 | -1.388433717 | 0.495336469 |
| endo-siRNA | URS00004498CD | RNA5237 | 12 | 3 | -1.388433717 | 0.495336469 |
| endo-siRNA | URS0000441A38 | RNA9772 | 23 | 8 | -0.932876638 | 0.495336469 |
| endo-siRNA | URS00004F395F | RNA3385 | 23 | 8 | -0.932876638 | 0.495336469 |
| endo-siRNA | URS000046FD56 | RNA13131 | 23 | 8 | -0.932876638 | 0.495336469 |
| endo-siRNA | URS00004FFDA3 | RNA682 | 13 | 16 | 0.874134759 | 0.495336469 |
| endo-siRNA | URS00002EA9D2 | RNA5017 | 13 | 16 | 0.874134759 | 0.495336469 |
| endo-siRNA | URS0000345833 | RNA14058 | 259 | 120 | -0.527435913 | 0.495983323 |
| endo-siRNA | URS000004802E | RNA12071 | 59 | 25 | -0.654567855 | 0.497439986 |
| endo-siRNA | URS000007CAD2 | RNA5787 | 19 | 6 | -1.068313038 | 0.49815383 |
| endo-siRNA | URS000055B605 | RNA5936 | 19 | 6 | -1.068313038 | 0.49815383 |
| endo-siRNA | URS0000277B27 | RNA5653 | 10 | 13 | 0.95023023 | 0.49815383 |
| endo-siRNA | URS000023356D | RNA1379 | 10 | 13 | 0.95023023 | 0.49815383 |
| endo-siRNA | URS00003600E7 | RNA1903 | 10 | 13 | 0.95023023 | 0.49815383 |
| endo-siRNA | URS0000061C69 | RNA14272 | 16 | 5 | -1.080807652 | 0.49815383 |
| endo-siRNA | URS00005826E5 | RNA9614 | 16 | 5 | -1.080807652 | 0.49815383 |
| endo-siRNA | URS0000229ADC | RNA7379 | 16 | 5 | -1.080807652 | 0.49815383 |
| endo-siRNA | URS0000135E72 | RNA4322 | 16 | 5 | -1.080807652 | 0.49815383 |
| endo-siRNA | URS0000195272 | RNA7625 | 16 | 5 | -1.080807652 | 0.49815383 |
| endo-siRNA | URS00004B47F2 | RNA12631 | 16 | 5 | -1.080807652 | 0.49815383 |
| endo-siRNA | URS0000091BA5 | RNA11937 | 16 | 5 | -1.080807652 | 0.49815383 |
| endo-siRNA | URS000013073C | RNA863 | 16 | 5 | -1.080807652 | 0.49815383 |
| endo-siRNA | URS00004F896B | RNA6764 | 282 | 131 | -0.523679507 | 0.498534085 |
| endo-siRNA | URS00003E87E9 | RNA154 | 21 | 24 | 0.770503909 | 0.498757896 |
| endo-siRNA | URS00000AACD9 | RNA9762 | 35 | 14 | -0.735699289 | 0.498757896 |
| endo-siRNA | URS00005CD498 | RNA13999 | 398 | 376 | 0.499918379 | 0.498757896 |
| endo-siRNA | URS00000066B0 | RNA5931 | 119 | 54 | -0.55696061 | 0.498757896 |
| endo-siRNA | URS0000209DED | RNA9821 | 56 | 23 | -0.699269477 | 0.499291222 |
| endo-siRNA | URS00002AD34D | RNA11263 | 36 | 38 | 0.657917815 | 0.499291222 |
| endo-siRNA | URS0000513F65 | RNA1004 | 99 | 44 | -0.586715268 | 0.503215463 |
| endo-siRNA | URS000048D28E | RNA12881 | 147 | 67 | -0.550785914 | 0.505897726 |
| endo-siRNA | URS00001CEE60 | RNA4344 | 32 | 12 | -0.827678994 | 0.50660631 |
| endo-siRNA | URS00001A1CFD | RNA759 | 32 | 12 | -0.827678994 | 0.50660631 |
| endo-siRNA | URS00003E8AB0 | RNA4101 | 31 | 12 | -0.782092249 | 0.50660631 |
| endo-siRNA | URS000056779F | RNA3485 | 31 | 12 | -0.782092249 | 0.50660631 |
| endo-siRNA | URS00002BF37C | RNA5209 | 61 | 26 | -0.646180098 | 0.508351322 |
| endo-siRNA | URS00005AD3BB | RNA3805 | 58 | 58 | 0.580878102 | 0.508506762 |
| endo-siRNA | URS00003610A3 | RNA9469 | 1340 | 641 | -0.481659533 | 0.512088379 |
| endo-siRNA | URS0000040490 | RNA3189 | 340 | 160 | -0.505082964 | 0.512505794 |
| endo-siRNA | URS00000E24B6 | RNA12875 | 240 | 112 | -0.517034046 | 0.512561851 |
| endo-siRNA | URS00000DD97D | RNA9292 | 74 | 32 | -0.625752516 | 0.512945353 |
| endo-siRNA | URS00001E9760 | RNA4722 | 74 | 32 | -0.625752516 | 0.512945353 |
| endo-siRNA | URS000054B49F | RNA3370 | 49 | 20 | -0.707870683 | 0.512945353 |
| endo-siRNA | URS00005CE73F | RNA1247 | 48 | 20 | -0.678214992 | 0.512945353 |
| endo-siRNA | URS00001C1082 | RNA3369 | 57 | 24 | -0.663597018 | 0.512945353 |
| endo-siRNA | URS00005D5950 | RNA2717 | 28 | 10 | -0.896643379 | 0.512945353 |
| endo-siRNA | URS00004C1DC2 | RNA13913 | 16 | 19 | 0.82417126 | 0.512945353 |
| endo-siRNA | URS000057CE6D | RNA860 | 114 | 51 | -0.577417573 | 0.512945353 |
| endo-siRNA | URS0000007540 | RNA10442 | 114 | 51 | -0.577417573 | 0.512945353 |
| endo-siRNA | URS000043CC68 | RNA608 | 197 | 91 | -0.53165476 | 0.513568456 |
| endo-siRNA | URS000036D5A0 | RNA13986 | 443 | 210 | -0.494604505 | 0.513594631 |
| endo-siRNA | URS00004A636A | RNA12661 | 1531 | 735 | -0.476489607 | 0.515672469 |
| endo-siRNA | URS0000431811 | RNA6462 | 91 | 89 | 0.549299817 | 0.519058698 |
| endo-siRNA | URS00001C704F | RNA873 | 71 | 31 | -0.611828029 | 0.519499558 |
| endo-siRNA | URS00001545C4 | RNA523 | 375 | 178 | -0.492665379 | 0.520752648 |
| endo-siRNA | URS000011944E | RNA3497 | 93 | 41 | -0.598298133 | 0.520752648 |
| endo-siRNA | URS00004F9E55 | RNA11996 | 159 | 73 | -0.540327186 | 0.520752648 |
| endo-siRNA | URS000012717A | RNA11009 | 24 | 9 | -0.825945622 | 0.520893179 |
| endo-siRNA | URS0000238D3F | RNA6036 | 24 | 9 | -0.825945622 | 0.520893179 |
| endo-siRNA | URS000002D1FF | RNA12007 | 24 | 9 | -0.825945622 | 0.520893179 |
| endo-siRNA | URS00004A97CF | RNA912 | 448 | 213 | -0.490336673 | 0.521006671 |
| endo-siRNA | URS000012A1B3 | RNA6828 | 76 | 33 | -0.619892162 | 0.522314853 |
| endo-siRNA | URS000010AB96 | RNA3507 | 76 | 33 | -0.619892162 | 0.522314853 |
| endo-siRNA | URS000026584E | RNA1239 | 44 | 45 | 0.612834906 | 0.524296822 |
| endo-siRNA | URS000047B738 | RNA9697 | 244 | 115 | -0.502764512 | 0.524414497 |
| endo-siRNA | URS00003E71CB | RNA14245 | 89 | 40 | -0.570513133 | 0.524607157 |
| endo-siRNA | URS00000D8E41 | RNA12882 | 214 | 100 | -0.51506395 | 0.526776965 |
| endo-siRNA | URS0000484FEE | RNA4825 | 255 | 239 | 0.48838312 | 0.527016776 |
| endo-siRNA | URS00001C8C4C | RNA9526 | 50 | 21 | -0.666881942 | 0.527284032 |
| endo-siRNA | URS000056EF7B | RNA10176 | 129 | 59 | -0.545700144 | 0.527284032 |
| endo-siRNA | URS000030D04C | RNA14016 | 64 | 63 | 0.558309362 | 0.527284032 |
| endo-siRNA | URS0000280348 | RNA14310 | 41 | 17 | -0.684768704 | 0.527284032 |
| endo-siRNA | URS00004B26A1 | RNA13945 | 21 | 7 | -0.99262132 | 0.527284032 |
| endo-siRNA | URS00004C8183 | RNA3376 | 327 | 156 | -0.485366869 | 0.528536679 |
| endo-siRNA | URS00005FDB62 | RNA9763 | 405 | 193 | -0.486993422 | 0.529819002 |
| endo-siRNA | URS00000F86E3 | RNA4282 | 55 | 23 | -0.673344285 | 0.53190235 |
| endo-siRNA | URS00000FF29F | RNA9875 | 104 | 47 | -0.562745356 | 0.532014128 |
| endo-siRNA | URS00001751BB | RNA7380 | 131 | 60 | -0.543663516 | 0.532014128 |
| endo-siRNA | URS000037ABF0 | RNA10025 | 46 | 19 | -0.690632106 | 0.534229207 |
| endo-siRNA | URS000029531F | RNA9605 | 901 | 436 | -0.464994449 | 0.534229207 |
| endo-siRNA | URS0000125E36 | RNA13818 | 17 | 20 | 0.811118649 | 0.534229207 |
| endo-siRNA | URS000029C290 | RNA6004 | 17 | 20 | 0.811118649 | 0.534229207 |
| endo-siRNA | URS00004370C8 | RNA2291 | 18 | 20 | 0.729357128 | 0.534229207 |
| endo-siRNA | URS00004A9DC0 | RNA5069 | 23 | 26 | 0.755164797 | 0.534229207 |
| endo-siRNA | URS00001576D0 | RNA14019 | 38 | 15 | -0.755005455 | 0.534229207 |
| endo-siRNA | URS00005C6C57 | RNA774 | 926 | 448 | -0.465311333 | 0.534229207 |
| endo-siRNA | URS00004279C9 | RNA564 | 9 | 12 | 0.985309464 | 0.534229207 |
| endo-siRNA | URS00004F6F20 | RNA6458 | 86 | 39 | -0.557562584 | 0.535252768 |
| endo-siRNA | URS00001A8443 | RNA12120 | 139 | 131 | 0.496142557 | 0.535252768 |
| endo-siRNA | URS00000DD019 | RNA8674 | 1026 | 498 | -0.460620563 | 0.535252768 |
| endo-siRNA | URS000056FC57 | RNA2622 | 142 | 134 | 0.498010863 | 0.535252768 |
| endo-siRNA | URS00002F1C2E | RNA5128 | 10 | 2 | -1.69080698 | 0.535252768 |
| endo-siRNA | URS000009E852 | RNA3031 | 10 | 2 | -1.69080698 | 0.535252768 |
| endo-siRNA | URS000043F28D | RNA5055 | 10 | 2 | -1.69080698 | 0.535252768 |
| endo-siRNA | URS0000344121 | RNA8220 | 10 | 2 | -1.69080698 | 0.535252768 |
| endo-siRNA | URS00000511AD | RNA11988 | 10 | 2 | -1.69080698 | 0.535252768 |
| endo-siRNA | URS000059E29C | RNA5985 | 10 | 2 | -1.69080698 | 0.535252768 |
| endo-siRNA | URS000015AAE4 | RNA1841 | 10 | 2 | -1.69080698 | 0.535252768 |
| endo-siRNA | URS00005DEC8B | RNA5165 | 10 | 2 | -1.69080698 | 0.535252768 |
| endo-siRNA | URS00003C7961 | RNA14 | 13 | 4 | -1.099199031 | 0.53536909 |
| endo-siRNA | URS0000496446 | RNA808 | 13 | 4 | -1.099199031 | 0.53536909 |
| endo-siRNA | URS000028BAE6 | RNA6092 | 13 | 4 | -1.099199031 | 0.53536909 |
| endo-siRNA | URS00003B15FD | RNA14129 | 13 | 4 | -1.099199031 | 0.53536909 |
| endo-siRNA | URS000054F1BE | RNA14118 | 13 | 4 | -1.099199031 | 0.53536909 |
| endo-siRNA | URS000003D964 | RNA134 | 13 | 4 | -1.099199031 | 0.53536909 |
| endo-siRNA | URS000045A31E | RNA11822 | 13 | 4 | -1.099199031 | 0.53536909 |
| endo-siRNA | URS000006A717 | RNA827 | 96 | 44 | -0.5423893 | 0.535425448 |
| endo-siRNA | URS000022E484 | RNA832 | 67 | 65 | 0.537388563 | 0.535425448 |
| endo-siRNA | URS0000567361 | RNA3368 | 27 | 29 | 0.682192376 | 0.53544966 |
| endo-siRNA | URS00004A1910 | RNA1006 | 43 | 18 | -0.671244206 | 0.53544966 |
| endo-siRNA | URS00004783A8 | RNA10452 | 42 | 18 | -0.637416184 | 0.53544966 |
| endo-siRNA | URS000052B28B | RNA14079 | 42 | 18 | -0.637416184 | 0.53544966 |
| endo-siRNA | URS000052F91E | RNA14130 | 42 | 18 | -0.637416184 | 0.53544966 |
| endo-siRNA | URS00000C3091 | RNA7576 | 83 | 37 | -0.582177165 | 0.535536822 |
| endo-siRNA | URS00003D7EE9 | RNA3488 | 133 | 62 | -0.518269971 | 0.535961849 |
| endo-siRNA | URS000021C14D | RNA32 | 266 | 127 | -0.484160302 | 0.537060054 |
| endo-siRNA | URS00002F3FB9 | RNA9467 | 584 | 283 | -0.462912984 | 0.537146914 |
| endo-siRNA | URS00005A5BF5 | RNA9604 | 2905 | 1422 | -0.448476313 | 0.537146914 |
| endo-siRNA | URS00001F8651 | RNA14373 | 21 | 23 | 0.709363922 | 0.537146914 |
| endo-siRNA | URS000002F367 | RNA10622 | 2378 | 1165 | -0.447272464 | 0.54027583 |
| endo-siRNA | URS00000F3523 | RNA5592 | 95 | 90 | 0.503440391 | 0.541317579 |
| endo-siRNA | URS00002EDBE5 | RNA14047 | 759 | 370 | -0.454355906 | 0.541317579 |
| endo-siRNA | URS000020D6B5 | RNA7328 | 684 | 333 | -0.456243307 | 0.54479998 |
| endo-siRNA | URS0000249E6A | RNA2559 | 52 | 23 | -0.592650474 | 0.545521586 |
| endo-siRNA | URS00003E73D7 | RNA151 | 406 | 197 | -0.460970333 | 0.546961185 |
| endo-siRNA | URS00003A1CD1 | RNA10446 | 75 | 34 | -0.557880829 | 0.547831488 |
| endo-siRNA | URS0000223B38 | RNA8571 | 75 | 34 | -0.557880829 | 0.547831488 |
| endo-siRNA | URS00000E06E4 | RNA14231 | 57 | 25 | -0.604943083 | 0.547831488 |
| endo-siRNA | URS0000085278 | RNA7357 | 30 | 12 | -0.735017905 | 0.547831488 |
| endo-siRNA | URS00003CEB5C | RNA6504 | 12 | 15 | 0.895730879 | 0.547831488 |
| endo-siRNA | URS00006141BF | RNA9509 | 12 | 15 | 0.895730879 | 0.547831488 |
| endo-siRNA | URS00002CC7AA | RNA4816 | 22 | 8 | -0.86917081 | 0.547831488 |
| endo-siRNA | URS0000553F9D | RNA5383 | 28 | 30 | 0.678753566 | 0.548263472 |
| endo-siRNA | URS000000A1A0 | RNA738 | 28 | 30 | 0.678753566 | 0.548263472 |
| endo-siRNA | URS0000477877 | RNA5295 | 240 | 115 | -0.478932531 | 0.548629568 |
| endo-siRNA | URS0000080B59 | RNA12129 | 189 | 90 | -0.487815717 | 0.554600725 |
| endo-siRNA | URS00003B1398 | RNA10398 | 72 | 32 | -0.586305154 | 0.554932559 |
| endo-siRNA | URS0000213417 | RNA11395 | 72 | 32 | -0.586305154 | 0.554932559 |
| endo-siRNA | URS0000413AEF | RNA441 | 22 | 24 | 0.703854509 | 0.555205753 |
| endo-siRNA | URS00004C3F56 | RNA5972 | 27 | 10 | -0.844460431 | 0.560354975 |
| endo-siRNA | URS0000105202 | RNA11948 | 27 | 10 | -0.844460431 | 0.560354975 |
| endo-siRNA | URS000019DD97 | RNA9401 | 27 | 10 | -0.844460431 | 0.560354975 |
| endo-siRNA | URS00000B1B06 | RNA6255 | 16 | 18 | 0.746588813 | 0.560354975 |
| endo-siRNA | URS0000054524 | RNA11002 | 16 | 18 | 0.746588813 | 0.560354975 |
| endo-siRNA | URS0000115F8A | RNA6089 | 18 | 6 | -0.990937702 | 0.560719675 |
| endo-siRNA | URS00005E58DC | RNA1185 | 18 | 6 | -0.990937702 | 0.560719675 |
| endo-siRNA | URS00005FE12F | RNA3931 | 18 | 6 | -0.990937702 | 0.560719675 |
| endo-siRNA | URS0000574996 | RNA18 | 18 | 6 | -0.990937702 | 0.560719675 |
| endo-siRNA | URS0000052B8C | RNA2496 | 18 | 6 | -0.990937702 | 0.560719675 |
| endo-siRNA | URS00000D8E63 | RNA5521 | 68 | 31 | -0.549677823 | 0.562249234 |
| endo-siRNA | URS00004CA739 | RNA983 | 78 | 36 | -0.532126865 | 0.565045123 |
| endo-siRNA | URS00001C78A0 | RNA842 | 50 | 22 | -0.600078938 | 0.566553125 |
| endo-siRNA | URS000051D994 | RNA14002 | 277 | 252 | 0.445443652 | 0.566671954 |
| endo-siRNA | URS00001B69C8 | RNA11997 | 93 | 43 | -0.52974885 | 0.566943601 |
| endo-siRNA | URS000029C782 | RNA10478 | 311 | 152 | -0.450475173 | 0.566943601 |
| endo-siRNA | URS000058FFC4 | RNA4264 | 106 | 98 | 0.468337271 | 0.566943601 |
| endo-siRNA | URS00006227D1 | RNA10420 | 156 | 75 | -0.473931138 | 0.566943601 |
| endo-siRNA | URS000011D108 | RNA11109 | 37 | 15 | -0.716684488 | 0.566943601 |
| endo-siRNA | URS00003672A3 | RNA14077 | 36 | 15 | -0.677317797 | 0.566943601 |
| endo-siRNA | URS00001ADD33 | RNA8463 | 36 | 15 | -0.677317797 | 0.566943601 |
| endo-siRNA | URS00001B7D0A | RNA2326 | 24 | 25 | 0.637791314 | 0.566943601 |
| endo-siRNA | URS000052005A | RNA9752 | 707 | 350 | -0.432135232 | 0.566943601 |
| endo-siRNA | URS0000384D88 | RNA9534 | 205 | 99 | -0.467606386 | 0.566943601 |
| endo-siRNA | URS00005C465A | RNA4087 | 278 | 136 | -0.449080921 | 0.566943601 |
| endo-siRNA | URS000027C959 | RNA10438 | 23 | 9 | -0.76493432 | 0.566943601 |
| endo-siRNA | URS000057A49A | RNA4219 | 23 | 9 | -0.76493432 | 0.566943601 |
| endo-siRNA | URS0000193438 | RNA2829 | 7 | 10 | 1.080494237 | 0.566943601 |
| endo-siRNA | URS000011E753 | RNA11669 | 7 | 10 | 1.080494237 | 0.566943601 |
| endo-siRNA | URS00000A9403 | RNA2592 | 7 | 10 | 1.080494237 | 0.566943601 |
| endo-siRNA | URS00002BC01D | RNA3639 | 7 | 10 | 1.080494237 | 0.566943601 |
| endo-siRNA | URS0000458326 | RNA5403 | 7 | 10 | 1.080494237 | 0.566943601 |
| endo-siRNA | URS00005D191C | RNA13012 | 7 | 10 | 1.080494237 | 0.566943601 |
| endo-siRNA | URS0000142E73 | RNA5275 | 15 | 5 | -0.988590582 | 0.566943601 |
| endo-siRNA | URS000052699E | RNA1205 | 15 | 5 | -0.988590582 | 0.566943601 |
| endo-siRNA | URS0000352F15 | RNA13764 | 15 | 5 | -0.988590582 | 0.566943601 |
| endo-siRNA | URS00000E35DD | RNA1254 | 15 | 5 | -0.988590582 | 0.566943601 |
| endo-siRNA | URS00003D54A2 | RNA547 | 15 | 5 | -0.988590582 | 0.566943601 |
| endo-siRNA | URS000054E52A | RNA6113 | 15 | 5 | -0.988590582 | 0.566943601 |
| endo-siRNA | URS0000233104 | RNA5880 | 15 | 5 | -0.988590582 | 0.566943601 |
| endo-siRNA | URS0000159F1F | RNA769 | 15 | 5 | -0.988590582 | 0.566943601 |
| endo-siRNA | URS00000EC818 | RNA9579 | 8 | 10 | 0.891634485 | 0.566943601 |
| endo-siRNA | URS000031E69D | RNA5254 | 8 | 10 | 0.891634485 | 0.566943601 |
| endo-siRNA | URS00005EF98B | RNA1310 | 8 | 10 | 0.891634485 | 0.566943601 |
| endo-siRNA | URS000052B909 | RNA8692 | 8 | 10 | 0.891634485 | 0.566943601 |
| endo-siRNA | URS000050C66B | RNA12527 | 14 | 5 | -0.890074043 | 0.566943601 |
| endo-siRNA | URS000027AE2B | RNA7638 | 14 | 5 | -0.890074043 | 0.566943601 |
| endo-siRNA | URS000039FF3E | RNA4316 | 14 | 5 | -0.890074043 | 0.566943601 |
| endo-siRNA | URS0000389C7D | RNA4089 | 14 | 5 | -0.890074043 | 0.566943601 |
| endo-siRNA | URS00004DF82B | RNA7560 | 46 | 20 | -0.617009688 | 0.569291596 |
| endo-siRNA | URS00003A8BEA | RNA9734 | 2953 | 1479 | -0.415421623 | 0.570715132 |
| endo-siRNA | URS00004BFE77 | RNA1264 | 34 | 35 | 0.621708577 | 0.570715132 |
| endo-siRNA | URS000054D491 | RNA4807 | 34 | 35 | 0.621708577 | 0.570715132 |
| endo-siRNA | URS00002AB951 | RNA9660 | 306 | 150 | -0.446199762 | 0.570715132 |
| endo-siRNA | URS00005CEDD1 | RNA14330 | 75 | 35 | -0.51618168 | 0.571042657 |
| endo-siRNA | URS000021E1BB | RNA14073 | 90 | 42 | -0.516388054 | 0.571042657 |
| endo-siRNA | URS0000476198 | RNA734 | 95 | 45 | -0.494957388 | 0.571042657 |
| endo-siRNA | URS0000408BC0 | RNA829 | 28 | 11 | -0.760440521 | 0.571042657 |
| endo-siRNA | URS00002087B8 | RNA772 | 28 | 11 | -0.760440521 | 0.571042657 |
| endo-siRNA | URS00001EEB6C | RNA8882 | 308 | 152 | -0.436497712 | 0.57315223 |
| endo-siRNA | URS0000229DE7 | RNA3430 | 175 | 85 | -0.459243719 | 0.574480309 |
| endo-siRNA | URS00004352ED | RNA849 | 11 | 3 | -1.264519851 | 0.574993816 |
| endo-siRNA | URS000024CF7C | RNA3387 | 11 | 3 | -1.264519851 | 0.574993816 |
| endo-siRNA | URS000029B130 | RNA12843 | 11 | 3 | -1.264519851 | 0.574993816 |
| endo-siRNA | URS00002184A0 | RNA3498 | 11 | 3 | -1.264519851 | 0.574993816 |
| endo-siRNA | URS00001E57D4 | RNA9454 | 11 | 3 | -1.264519851 | 0.574993816 |
| endo-siRNA | URS0000126680 | RNA13515 | 11 | 3 | -1.264519851 | 0.574993816 |
| endo-siRNA | URS0000106DA8 | RNA12429 | 11 | 3 | -1.264519851 | 0.574993816 |
| endo-siRNA | URS000050EBCC | RNA11989 | 11 | 3 | -1.264519851 | 0.574993816 |
| endo-siRNA | URS0000346566 | RNA7389 | 11 | 3 | -1.264519851 | 0.574993816 |
| endo-siRNA | URS0000273E1A | RNA3371 | 91 | 43 | -0.498435697 | 0.577340115 |
| endo-siRNA | URS0000043E21 | RNA11954 | 166 | 81 | -0.452596089 | 0.577340115 |
| endo-siRNA | URS00000821CE | RNA9468 | 2718 | 1369 | -0.407283813 | 0.577340115 |
| endo-siRNA | URS00002169D6 | RNA11278 | 61 | 58 | 0.508304651 | 0.577340115 |
| endo-siRNA | URS000054A9B5 | RNA6800 | 28 | 29 | 0.630009428 | 0.577340115 |
| endo-siRNA | URS000038F48A | RNA8485 | 29 | 29 | 0.579648258 | 0.577340115 |
| endo-siRNA | URS00004E4AC4 | RNA9911 | 62 | 28 | -0.563062178 | 0.577983549 |
| endo-siRNA | URS000054CD77 | RNA9569 | 11 | 13 | 0.814664736 | 0.577983549 |
| endo-siRNA | URS00002C0A53 | RNA2838 | 11 | 13 | 0.814664736 | 0.577983549 |
| endo-siRNA | URS000010438D | RNA5570 | 88 | 41 | -0.518702829 | 0.585838651 |
| endo-siRNA | URS00002958DB | RNA13694 | 199 | 98 | -0.43941459 | 0.586965039 |
| endo-siRNA | URS00003D58B5 | RNA10625 | 301 | 149 | -0.43208704 | 0.587030711 |
| endo-siRNA | URS000048B3BC | RNA9409 | 24 | 10 | -0.67553064 | 0.587518747 |
| endo-siRNA | URS00002DCF2B | RNA5050 | 44 | 43 | 0.547395518 | 0.589693728 |
| endo-siRNA | URS0000391099 | RNA2491 | 63 | 29 | -0.535641751 | 0.589693728 |
| endo-siRNA | URS0000465B5E | RNA8562 | 59 | 27 | -0.54396287 | 0.590472048 |
| endo-siRNA | URS000043625A | RNA4128 | 58 | 27 | -0.519363843 | 0.590472048 |
| endo-siRNA | URS0000529E96 | RNA921 | 405 | 202 | -0.421272304 | 0.590472048 |
| endo-siRNA | URS0000167756 | RNA503 | 29 | 12 | -0.686355592 | 0.590521253 |
| endo-siRNA | URS000009DB2F | RNA9773 | 29 | 12 | -0.686355592 | 0.590521253 |
| endo-siRNA | URS0000238D44 | RNA822 | 54 | 24 | -0.585804692 | 0.590556935 |
| endo-siRNA | URS0000582626 | RNA5184 | 49 | 22 | -0.571020584 | 0.591510167 |
| endo-siRNA | URS0000235EFF | RNA14086 | 155 | 76 | -0.445578742 | 0.591717411 |
| endo-siRNA | URS00001DCEC1 | RNA13116 | 26 | 26 | 0.579365197 | 0.591717411 |
| endo-siRNA | URS00001D9121 | RNA5260 | 26 | 26 | 0.579365197 | 0.591717411 |
| endo-siRNA | URS000055414F | RNA7447 | 248 | 123 | -0.429266368 | 0.594344088 |
| endo-siRNA | URS000018D7C1 | RNA5595 | 96 | 88 | 0.455972127 | 0.597099694 |
| endo-siRNA | URS0000218843 | RNA8888 | 425 | 214 | -0.407572695 | 0.598809224 |
| endo-siRNA | URS0000197D8D | RNA9568 | 9 | 11 | 0.860863366 | 0.599928168 |
| endo-siRNA | URS00003D9AAF | RNA14270 | 9 | 11 | 0.860863366 | 0.599928168 |
| endo-siRNA | URS000046E878 | RNA2413 | 116 | 56 | -0.467798712 | 0.600369612 |
| endo-siRNA | URS000058C137 | RNA9392 | 158 | 77 | -0.454374245 | 0.60156919 |
| endo-siRNA | URS00004B3B6C | RNA10460 | 127 | 62 | -0.451748944 | 0.604440652 |
| endo-siRNA | URS000024E296 | RNA6631 | 81 | 38 | -0.508680255 | 0.604724061 |
| endo-siRNA | URS0000466B7F | RNA958 | 197 | 176 | 0.419216284 | 0.60552908 |
| endo-siRNA | URS00002F15DC | RNA9769 | 50 | 23 | -0.536232791 | 0.60552908 |
| endo-siRNA | URS0000442BD7 | RNA830 | 853 | 434 | -0.392658154 | 0.606115357 |
| endo-siRNA | URS000037E6C0 | RNA8985 | 27 | 27 | 0.579466508 | 0.606115357 |
| endo-siRNA | URS00003595CF | RNA14071 | 40 | 18 | -0.567283338 | 0.606115357 |
| endo-siRNA | URS0000496A62 | RNA5473 | 40 | 18 | -0.567283338 | 0.606115357 |
| endo-siRNA | URS00001994EF | RNA9186 | 20 | 8 | -0.732643184 | 0.606115357 |
| endo-siRNA | URS0000013D2E | RNA5468 | 20 | 8 | -0.732643184 | 0.606115357 |
| endo-siRNA | URS00004B0CA8 | RNA5208 | 20 | 8 | -0.732643184 | 0.606115357 |
| endo-siRNA | URS00003E83A9 | RNA14321 | 13 | 14 | 0.68277074 | 0.606115357 |
| endo-siRNA | URS00005B7079 | RNA14368 | 13 | 14 | 0.68277074 | 0.606115357 |
| endo-siRNA | URS00004568FD | RNA5027 | 66 | 31 | -0.506705247 | 0.606115357 |
| endo-siRNA | URS000005BA84 | RNA9720 | 35 | 15 | -0.636846705 | 0.606115357 |
| endo-siRNA | URS0000094C8D | RNA11971 | 24 | 24 | 0.579137379 | 0.606115357 |
| endo-siRNA | URS00000B132B | RNA9823 | 24 | 24 | 0.579137379 | 0.606115357 |
| endo-siRNA | URS00005C4228 | RNA4738 | 26 | 10 | -0.790318939 | 0.606115357 |
| endo-siRNA | URS000010B366 | RNA4085 | 26 | 10 | -0.790318939 | 0.606115357 |
| endo-siRNA | URS000003C1EB | RNA5958 | 26 | 10 | -0.790318939 | 0.606115357 |
| endo-siRNA | URS00002EEEC5 | RNA9407 | 25 | 10 | -0.734066132 | 0.606115357 |
| endo-siRNA | URS000019D155 | RNA6810 | 25 | 10 | -0.734066132 | 0.606115357 |
| endo-siRNA | URS000011F147 | RNA5293 | 410 | 207 | -0.403709586 | 0.609066265 |
| endo-siRNA | URS000027B9B7 | RNA10343 | 56 | 26 | -0.523113373 | 0.610434741 |
| endo-siRNA | URS000051A303 | RNA9504 | 230 | 204 | 0.408816567 | 0.610434741 |
| endo-siRNA | URS00005198D9 | RNA6075 | 289 | 145 | -0.412655566 | 0.610434741 |
| endo-siRNA | URS000057A429 | RNA492 | 205 | 103 | -0.410519083 | 0.616105916 |
| endo-siRNA | URS00004E23BA | RNA12664 | 110 | 54 | -0.443651036 | 0.617282502 |
| endo-siRNA | URS00001ED3DD | RNA3448 | 1320 | 678 | -0.379018285 | 0.617282502 |
| endo-siRNA | URS00001AA4D5 | RNA4325 | 62 | 29 | -0.512613364 | 0.617282502 |
| endo-siRNA | URS00002170A4 | RNA8895 | 218 | 109 | -0.417553774 | 0.617282502 |
| endo-siRNA | URS000048B315 | RNA5018 | 12 | 4 | -0.985091475 | 0.617282502 |
| endo-siRNA | URS00005F9C91 | RNA13539 | 12 | 4 | -0.985091475 | 0.617282502 |
| endo-siRNA | URS00004F1D4F | RNA9903 | 12 | 4 | -0.985091475 | 0.617282502 |
| endo-siRNA | URS000001B4A9 | RNA4207 | 12 | 4 | -0.985091475 | 0.617282502 |
| endo-siRNA | URS00000CD67A | RNA5606 | 12 | 4 | -0.985091475 | 0.617282502 |
| endo-siRNA | URS00000A5AB0 | RNA844 | 12 | 4 | -0.985091475 | 0.617282502 |
| endo-siRNA | URS0000328E79 | RNA3335 | 12 | 4 | -0.985091475 | 0.617282502 |
| endo-siRNA | URS0000276BF9 | RNA9200 | 12 | 4 | -0.985091475 | 0.617282502 |
| endo-siRNA | URS000004FDF7 | RNA8583 | 12 | 4 | -0.985091475 | 0.617282502 |
| endo-siRNA | URS00000D2793 | RNA663 | 12 | 4 | -0.985091475 | 0.617282502 |
| endo-siRNA | URS00005D4A23 | RNA2913 | 12 | 4 | -0.985091475 | 0.617282502 |
| endo-siRNA | URS00001EAD39 | RNA2804 | 12 | 4 | -0.985091475 | 0.617282502 |
| endo-siRNA | URS000000A2C1 | RNA4201 | 12 | 4 | -0.985091475 | 0.617282502 |
| endo-siRNA | URS00005E79CC | RNA2728 | 12 | 4 | -0.985091475 | 0.617282502 |
| endo-siRNA | URS00005A8660 | RNA12002 | 37 | 16 | -0.624173053 | 0.619708759 |
| endo-siRNA | URS000058A847 | RNA5054 | 37 | 16 | -0.624173053 | 0.619708759 |
| endo-siRNA | URS000032BAD7 | RNA3156 | 25 | 25 | 0.579255822 | 0.619708759 |
| endo-siRNA | URS000049AEE6 | RNA10839 | 94 | 85 | 0.436315109 | 0.62017674 |
| endo-siRNA | URS000031ADC9 | RNA9641 | 366 | 186 | -0.394242108 | 0.620785548 |
| endo-siRNA | URS00001F6AAC | RNA12663 | 626 | 321 | -0.381371592 | 0.620785548 |
| endo-siRNA | URS00004BE766 | RNA9638 | 84 | 41 | -0.451705439 | 0.620785548 |
| endo-siRNA | URS000035D0C2 | RNA14080 | 938 | 483 | -0.375382977 | 0.622077038 |
| endo-siRNA | URS00001F6179 | RNA10022 | 32 | 14 | -0.606992883 | 0.622295759 |
| endo-siRNA | URS000033D2DF | RNA4821 | 32 | 14 | -0.606992883 | 0.622295759 |
| endo-siRNA | URS00002470D3 | RNA9689 | 106 | 52 | -0.444630954 | 0.623215526 |
| endo-siRNA | URS0000378F99 | RNA14242 | 63 | 30 | -0.486897612 | 0.624808199 |
| endo-siRNA | URS00003A1087 | RNA3526 | 27 | 11 | -0.708257573 | 0.624808199 |
| endo-siRNA | URS000013DB77 | RNA9757 | 27 | 11 | -0.708257573 | 0.624808199 |
| endo-siRNA | URS0000407265 | RNA13603 | 27 | 11 | -0.708257573 | 0.624808199 |
| endo-siRNA | URS0000044D0B | RNA6114 | 17 | 18 | 0.659913785 | 0.624808199 |
| endo-siRNA | URS000059A440 | RNA6830 | 17 | 18 | 0.659913785 | 0.624808199 |
| endo-siRNA | URS000060A87E | RNA1244 | 47 | 22 | -0.511086791 | 0.625003401 |
| endo-siRNA | URS00004C761B | RNA11528 | 17 | 6 | -0.909176181 | 0.625090622 |
| endo-siRNA | URS0000540749 | RNA3570 | 17 | 6 | -0.909176181 | 0.625090622 |
| endo-siRNA | URS00004C6C17 | RNA891 | 10 | 12 | 0.835671544 | 0.625090622 |
| endo-siRNA | URS00005143A4 | RNA4336 | 10 | 12 | 0.835671544 | 0.625090622 |
| endo-siRNA | URS000059F3D8 | RNA966 | 54 | 50 | 0.469969083 | 0.625254148 |
| endo-siRNA | URS00005541CD | RNA14076 | 2373 | 1229 | -0.367087536 | 0.625254148 |
| endo-siRNA | URS000043DAF2 | RNA5146 | 22 | 9 | -0.701228492 | 0.625254148 |
| endo-siRNA | URS000044A399 | RNA7446 | 156 | 78 | -0.417421585 | 0.625899195 |
| endo-siRNA | URS000024D832 | RNA9639 | 167 | 84 | -0.408849347 | 0.6260081 |
| endo-siRNA | URS00003AEEAC | RNA9698 | 178 | 90 | -0.401377368 | 0.626060025 |
| endo-siRNA | URS00005BA221 | RNA1204 | 278 | 141 | -0.397030044 | 0.626534288 |
| endo-siRNA | URS000028A551 | RNA612 | 43 | 19 | -0.59366176 | 0.627431492 |
| endo-siRNA | URS00004A7867 | RNA12672 | 30 | 29 | 0.530985945 | 0.627431492 |
| endo-siRNA | URS0000452067 | RNA10014 | 91 | 45 | -0.432996309 | 0.629091272 |
| endo-siRNA | URS00003C898B | RNA809 | 53 | 25 | -0.500259052 | 0.630038171 |
| endo-siRNA | URS00003C99A2 | RNA156 | 53 | 25 | -0.500259052 | 0.630038171 |
| endo-siRNA | URS00004FDA68 | RNA13997 | 64 | 31 | -0.462413275 | 0.63134244 |
| endo-siRNA | URS00000679C2 | RNA9761 | 87 | 42 | -0.467561161 | 0.63138447 |
| endo-siRNA | URS00002D5A2D | RNA14293 | 97 | 48 | -0.43205854 | 0.63138447 |
| endo-siRNA | URS000030265B | RNA11309 | 49 | 23 | -0.507174437 | 0.634766611 |
| endo-siRNA | URS0000536A00 | RNA10669 | 35 | 33 | 0.495429805 | 0.634766611 |
| endo-siRNA | URS0000185B90 | RNA9549 | 71 | 34 | -0.478971353 | 0.635932618 |
| endo-siRNA | URS00000BA372 | RNA2415 | 22 | 22 | 0.578868374 | 0.636372043 |
| endo-siRNA | URS000032909C | RNA11435 | 22 | 22 | 0.578868374 | 0.636372043 |
| endo-siRNA | URS00004269EE | RNA994 | 22 | 22 | 0.578868374 | 0.636372043 |
| endo-siRNA | URS0000417431 | RNA9784 | 2093 | 1091 | -0.357778886 | 0.638254382 |
| endo-siRNA | URS0000369293 | RNA9877 | 301 | 154 | -0.384500473 | 0.638573068 |
| endo-siRNA | URS000023FBB5 | RNA7384 | 222 | 113 | -0.391819684 | 0.639461075 |
| endo-siRNA | URS0000220AEC | RNA12565 | 31 | 30 | 0.53265574 | 0.640566017 |
| endo-siRNA | URS00002BD39F | RNA4171 | 66 | 32 | -0.461046701 | 0.641131067 |
| endo-siRNA | URS000029D333 | RNA5182 | 28 | 12 | -0.635994423 | 0.642615893 |
| endo-siRNA | URS0000142E21 | RNA5533 | 28 | 12 | -0.635994423 | 0.642615893 |
| endo-siRNA | URS000025C003 | RNA4281 | 19 | 19 | 0.578359374 | 0.642615893 |
| endo-siRNA | URS00004A402A | RNA723 | 133 | 116 | 0.384431547 | 0.645317536 |
| endo-siRNA | URS00000FAEF9 | RNA14333 | 72 | 35 | -0.457407876 | 0.645406525 |
| endo-siRNA | URS00002243A7 | RNA13995 | 401 | 344 | 0.360800265 | 0.64576857 |
| endo-siRNA | URS0000430B43 | RNA12874 | 179 | 91 | -0.393529006 | 0.646678103 |
| endo-siRNA | URS000049660C | RNA773 | 722 | 376 | -0.35906829 | 0.647277695 |
| endo-siRNA | URS0000044F3C | RNA7365 | 454 | 235 | -0.367779991 | 0.647396034 |
| endo-siRNA | URS0000098E41 | RNA1215 | 15 | 16 | 0.669878048 | 0.647945211 |
| endo-siRNA | URS0000421005 | RNA5900 | 23 | 10 | -0.614519338 | 0.647945211 |
| endo-siRNA | URS00005BA56D | RNA9331 | 23 | 10 | -0.614519338 | 0.647945211 |
| endo-siRNA | URS0000273292 | RNA5591 | 23 | 10 | -0.614519338 | 0.647945211 |
| endo-siRNA | URS000032EACA | RNA6118 | 49 | 45 | 0.458056534 | 0.648917382 |
| endo-siRNA | URS0000355B16 | RNA7524 | 49 | 45 | 0.458056534 | 0.648917382 |
| endo-siRNA | URS00004FA525 | RNA794 | 56 | 27 | -0.46887057 | 0.648917382 |
| endo-siRNA | URS0000379BE8 | RNA9786 | 556 | 290 | -0.356809608 | 0.648917382 |
| endo-siRNA | URS00005468BE | RNA6070 | 142 | 72 | -0.397226113 | 0.648917382 |
| endo-siRNA | URS00000F8989 | RNA6071 | 2791 | 1466 | -0.346762724 | 0.648917382 |
| endo-siRNA | URS00005C947C | RNA13933 | 18 | 7 | -0.771931868 | 0.648917382 |
| endo-siRNA | URS000012C2C9 | RNA7235 | 18 | 7 | -0.771931868 | 0.648917382 |
| endo-siRNA | URS00000E84FE | RNA11300 | 18 | 7 | -0.771931868 | 0.648917382 |
| endo-siRNA | URS00005B8BF6 | RNA1116 | 18 | 7 | -0.771931868 | 0.648917382 |
| endo-siRNA | URS000028FE57 | RNA7386 | 45 | 21 | -0.515357851 | 0.648917382 |
| endo-siRNA | URS0000364A84 | RNA10621 | 45 | 21 | -0.515357851 | 0.648917382 |
| endo-siRNA | URS000055339C | RNA5101 | 34 | 15 | -0.595207452 | 0.648917382 |
| endo-siRNA | URS00005F222D | RNA14144 | 13 | 5 | -0.784333871 | 0.648917382 |
| endo-siRNA | URS00003D703E | RNA9719 | 13 | 5 | -0.784333871 | 0.648917382 |
| endo-siRNA | URS00001EEAC5 | RNA3937 | 13 | 5 | -0.784333871 | 0.648917382 |
| endo-siRNA | URS00003EFCEA | RNA13766 | 13 | 5 | -0.784333871 | 0.648917382 |
| endo-siRNA | URS00003F7E5B | RNA5788 | 13 | 5 | -0.784333871 | 0.648917382 |
| endo-siRNA | URS000039CC59 | RNA1203 | 13 | 5 | -0.784333871 | 0.648917382 |
| endo-siRNA | URS00000F34EC | RNA9814 | 13 | 5 | -0.784333871 | 0.648917382 |
| endo-siRNA | URS00000D804B | RNA9758 | 13 | 5 | -0.784333871 | 0.648917382 |
| endo-siRNA | URS00002F8B84 | RNA11129 | 13 | 5 | -0.784333871 | 0.648917382 |
| endo-siRNA | URS0000151F13 | RNA3496 | 13 | 5 | -0.784333871 | 0.648917382 |
| endo-siRNA | URS000047F647 | RNA13836 | 13 | 5 | -0.784333871 | 0.648917382 |
| endo-siRNA | URS000016D33C | RNA4685 | 171 | 87 | -0.39240046 | 0.649265934 |
| endo-siRNA | URS0000350FF4 | RNA9465 | 62 | 30 | -0.463869226 | 0.649265934 |
| endo-siRNA | URS0000545D3C | RNA4179 | 62 | 30 | -0.463869226 | 0.649265934 |
| endo-siRNA | URS00004AD32C | RNA10021 | 79 | 39 | -0.435300963 | 0.649265934 |
| endo-siRNA | URS0000329DFC | RNA3380 | 59 | 53 | 0.42645305 | 0.649265934 |
| endo-siRNA | URS000016D570 | RNA5971 | 137 | 69 | -0.406880039 | 0.649265934 |
| endo-siRNA | URS00004965D2 | RNA1201 | 513 | 268 | -0.354495168 | 0.649265934 |
| endo-siRNA | URS0000081232 | RNA922 | 787 | 412 | -0.351530241 | 0.649265934 |
| endo-siRNA | URS0000155029 | RNA9402 | 102 | 51 | -0.417175741 | 0.650553357 |
| endo-siRNA | URS0000410403 | RNA1202 | 749 | 392 | -0.351919061 | 0.651752855 |
| endo-siRNA | URS000032507A | RNA12876 | 225 | 116 | -0.373403027 | 0.652886698 |
| endo-siRNA | URS000002DF54 | RNA8230 | 29 | 13 | -0.571796907 | 0.655040921 |
| endo-siRNA | URS0000528D3B | RNA887 | 29 | 13 | -0.571796907 | 0.655040921 |
| endo-siRNA | URS000052EA5D | RNA3403 | 29 | 13 | -0.571796907 | 0.655040921 |
| endo-siRNA | URS0000559874 | RNA1349 | 74 | 66 | 0.416321718 | 0.655403156 |
| endo-siRNA | URS00003726BC | RNA14068 | 127 | 64 | -0.406017941 | 0.656688287 |
| endo-siRNA | URS00002F8A7A | RNA5897 | 46 | 22 | -0.48015959 | 0.659788981 |
| endo-siRNA | URS000024B50E | RNA9750 | 151 | 77 | -0.389061618 | 0.660900756 |
| endo-siRNA | URS000016F6B9 | RNA609 | 299 | 156 | -0.356283612 | 0.661646744 |
| endo-siRNA | URS00001466D0 | RNA9609 | 35 | 16 | -0.544335269 | 0.662375869 |
| endo-siRNA | URS000056DECC | RNA3398 | 38 | 35 | 0.461910574 | 0.662375869 |
| endo-siRNA | URS00005B35C2 | RNA4096 | 287 | 245 | 0.353680462 | 0.662375869 |
| endo-siRNA | URS00005D10BB | RNA3509 | 585 | 494 | 0.338110442 | 0.6711294 |
| endo-siRNA | URS000060E9FB | RNA3696 | 35 | 32 | 0.451172046 | 0.6711294 |
| endo-siRNA | URS000002A45C | RNA6554 | 21 | 21 | 0.578714771 | 0.6711294 |
| endo-siRNA | URS000023D9BC | RNA6080 | 30 | 14 | -0.514331795 | 0.6711294 |
| endo-siRNA | URS00004A03C5 | RNA8679 | 30 | 14 | -0.514331795 | 0.6711294 |
| endo-siRNA | URS000061D2B8 | RNA5961 | 30 | 14 | -0.514331795 | 0.6711294 |
| endo-siRNA | URS00004BC7ED | RNA4164 | 53 | 26 | -0.44389687 | 0.6711294 |
| endo-siRNA | URS00004B3935 | RNA11950 | 53 | 26 | -0.44389687 | 0.6711294 |
| endo-siRNA | URS000020EA6D | RNA6867 | 12 | 13 | 0.69075087 | 0.6711294 |
| endo-siRNA | URS00001941DE | RNA3419 | 19 | 8 | -0.659207294 | 0.6711294 |
| endo-siRNA | URS00000EE641 | RNA4263 | 13 | 13 | 0.576643314 | 0.6711294 |
| endo-siRNA | URS000036DE03 | RNA5202 | 13 | 13 | 0.576643314 | 0.6711294 |
| endo-siRNA | URS000024FCE3 | RNA616 | 114 | 58 | -0.392203103 | 0.672114949 |
| endo-siRNA | URS000020C174 | RNA11959 | 113 | 58 | -0.379508815 | 0.672114949 |
| endo-siRNA | URS0000039F6D | RNA1329 | 507 | 268 | -0.337527076 | 0.672114949 |
| endo-siRNA | URS000018BD34 | RNA6078 | 223 | 116 | -0.360530349 | 0.672114949 |
| endo-siRNA | URS00001639A0 | RNA5848 | 44 | 40 | 0.443310182 | 0.672114949 |
| endo-siRNA | URS0000418600 | RNA9521 | 126 | 109 | 0.372627065 | 0.673896688 |
| endo-siRNA | URS00004E8147 | RNA9782 | 1131 | 601 | -0.329999648 | 0.674309928 |
| endo-siRNA | URS0000374BD4 | RNA8911 | 37 | 17 | -0.537238037 | 0.674309928 |
| endo-siRNA | URS000025633D | RNA12475 | 37 | 17 | -0.537238037 | 0.674309928 |
| endo-siRNA | URS000025EDE4 | RNA5594 | 98 | 85 | 0.376287862 | 0.67507066 |
| endo-siRNA | URS00005C3C87 | RNA10451 | 164 | 85 | -0.365667415 | 0.67507066 |
| endo-siRNA | URS00004BA5CE | RNA12466 | 9 | 10 | 0.724660508 | 0.67507066 |
| endo-siRNA | URS0000620706 | RNA9535 | 9 | 10 | 0.724660508 | 0.67507066 |
| endo-siRNA | URS0000488BA4 | RNA4487 | 9 | 10 | 0.724660508 | 0.67507066 |
| endo-siRNA | URS00001C8566 | RNA9600 | 201 | 105 | -0.354393345 | 0.67507066 |
| endo-siRNA | URS000020EDDF | RNA13998 | 599 | 502 | 0.327171364 | 0.67507066 |
| endo-siRNA | URS000042801D | RNA5969 | 26 | 11 | -0.65411608 | 0.67507066 |
| endo-siRNA | URS00000EAF10 | RNA14084 | 26 | 11 | -0.65411608 | 0.67507066 |
| endo-siRNA | URS000000B6EE | RNA11302 | 25 | 11 | -0.597863274 | 0.67507066 |
| endo-siRNA | URS000056DBC4 | RNA13905 | 25 | 11 | -0.597863274 | 0.67507066 |
| endo-siRNA | URS000019878D | RNA528 | 17 | 17 | 0.577920965 | 0.67507066 |
| endo-siRNA | URS000018D278 | RNA12017 | 17 | 17 | 0.577920965 | 0.67507066 |
| endo-siRNA | URS000014F408 | RNA4331 | 146 | 76 | -0.359364807 | 0.67507066 |
| endo-siRNA | URS00001B387D | RNA5226 | 104 | 53 | -0.389760965 | 0.67507066 |
| endo-siRNA | URS00001F1B7B | RNA4829 | 98 | 50 | -0.388059263 | 0.67507066 |
| endo-siRNA | URS00004C2224 | RNA3487 | 91 | 46 | -0.401357074 | 0.67507066 |
| endo-siRNA | URS00004FFA5C | RNA12153 | 10 | 3 | -1.128954357 | 0.67507066 |
| endo-siRNA | URS0000571564 | RNA5829 | 10 | 3 | -1.128954357 | 0.67507066 |
| endo-siRNA | URS0000586677 | RNA12123 | 10 | 3 | -1.128954357 | 0.67507066 |
| endo-siRNA | URS00005767E3 | RNA13247 | 10 | 3 | -1.128954357 | 0.67507066 |
| endo-siRNA | URS0000210C6F | RNA7636 | 10 | 3 | -1.128954357 | 0.67507066 |
| endo-siRNA | URS000059FFDA | RNA10663 | 10 | 3 | -1.128954357 | 0.67507066 |
| endo-siRNA | URS00003BA3FB | RNA9991 | 10 | 3 | -1.128954357 | 0.67507066 |
| endo-siRNA | URS00000718F7 | RNA3414 | 10 | 3 | -1.128954357 | 0.67507066 |
| endo-siRNA | URS000010A831 | RNA9436 | 10 | 3 | -1.128954357 | 0.67507066 |
| endo-siRNA | URS00001E7261 | RNA11824 | 10 | 3 | -1.128954357 | 0.67507066 |
| endo-siRNA | URS00005059BA | RNA4801 | 10 | 3 | -1.128954357 | 0.67507066 |
| endo-siRNA | URS00002B067B | RNA14055 | 261 | 138 | -0.337050245 | 0.683595041 |
| endo-siRNA | URS0000095A2F | RNA6759 | 56 | 50 | 0.417644286 | 0.683595041 |
| endo-siRNA | URS00003B9AC9 | RNA14072 | 44 | 21 | -0.483045216 | 0.683595041 |
| endo-siRNA | URS000059E04D | RNA901 | 50 | 24 | -0.475092803 | 0.683595041 |
| endo-siRNA | URS00000C8D39 | RNA12004 | 200 | 105 | -0.347203219 | 0.686569362 |
| endo-siRNA | URS000037AAC6 | RNA5435 | 21 | 9 | -0.634579092 | 0.686569362 |
| endo-siRNA | URS000047BC7E | RNA5215 | 21 | 9 | -0.634579092 | 0.686569362 |
| endo-siRNA | URS00000E3515 | RNA5517 | 21 | 9 | -0.634579092 | 0.686569362 |
| endo-siRNA | URS0000250F22 | RNA11603 | 21 | 9 | -0.634579092 | 0.686569362 |
| endo-siRNA | URS000039A896 | RNA9335 | 14 | 14 | 0.577030567 | 0.686569362 |
| endo-siRNA | URS00005E3E43 | RNA755 | 14 | 14 | 0.577030567 | 0.686569362 |
| endo-siRNA | URS0000402334 | RNA5781 | 14 | 14 | 0.577030567 | 0.686569362 |
| endo-siRNA | URS00000C168E | RNA5229 | 176 | 92 | -0.35341534 | 0.686851428 |
| endo-siRNA | URS00003700DF | RNA9552 | 429 | 229 | -0.323390148 | 0.686851428 |
| endo-siRNA | URS00001F0553 | RNA11277 | 98 | 84 | 0.359234555 | 0.688118132 |
| endo-siRNA | URS000047F506 | RNA8452 | 18 | 18 | 0.578152263 | 0.692141946 |
| endo-siRNA | URS00005350A0 | RNA9476 | 19 | 18 | 0.500776928 | 0.692141946 |
| endo-siRNA | URS00004EDA8B | RNA8488 | 45 | 22 | -0.448554847 | 0.693045148 |
| endo-siRNA | URS00002DB372 | RNA3492 | 45 | 22 | -0.448554847 | 0.693045148 |
| endo-siRNA | URS00002672AB | RNA5203 | 33 | 15 | -0.552330592 | 0.693045148 |
| endo-siRNA | URS00003549A7 | RNA13698 | 39 | 19 | -0.453313103 | 0.693045148 |
| endo-siRNA | URS0000215606 | RNA1181 | 327 | 174 | -0.327921316 | 0.693805796 |
| endo-siRNA | URS0000078BD8 | RNA5225 | 836 | 449 | -0.314611222 | 0.69430286 |
| endo-siRNA | URS0000398276 | RNA14326 | 85 | 73 | 0.36198247 | 0.695095777 |
| endo-siRNA | URS00005923D7 | RNA7026 | 102 | 53 | -0.361787297 | 0.696719211 |
| endo-siRNA | URS00000027A1 | RNA7385 | 225 | 119 | -0.336597623 | 0.696820851 |
| endo-siRNA | URS000030EA5D | RNA5263 | 107 | 91 | 0.348007828 | 0.699594908 |
| endo-siRNA | URS00001EE15E | RNA3525 | 84 | 43 | -0.383156156 | 0.699594908 |
| endo-siRNA | URS00004457E0 | RNA9670 | 16 | 6 | -0.822501152 | 0.699594908 |
| endo-siRNA | URS00004B8D63 | RNA9360 | 16 | 6 | -0.822501152 | 0.699594908 |
| endo-siRNA | URS00004B4063 | RNA1003 | 16 | 6 | -0.822501152 | 0.699594908 |
| endo-siRNA | URS00000D575B | RNA7455 | 10 | 11 | 0.711225446 | 0.699594908 |
| endo-siRNA | URS0000228953 | RNA11591 | 10 | 11 | 0.711225446 | 0.699594908 |
| endo-siRNA | URS000058BA74 | RNA6093 | 10 | 11 | 0.711225446 | 0.699594908 |
| endo-siRNA | URS00002CE7CD | RNA7333 | 10 | 11 | 0.711225446 | 0.699594908 |
| endo-siRNA | URS0000200068 | RNA11592 | 39 | 35 | 0.424581209 | 0.700505979 |
| endo-siRNA | URS000044B085 | RNA14244 | 456 | 245 | -0.314023514 | 0.700572449 |
| endo-siRNA | URS00004DB729 | RNA4536 | 464 | 249 | -0.315751795 | 0.701306117 |
| endo-siRNA | URS00005FEDD9 | RNA8306 | 34 | 31 | 0.447152753 | 0.701558578 |
| endo-siRNA | URS00001D1FCE | RNA629 | 35 | 31 | 0.4055135 | 0.701558578 |
| endo-siRNA | URS0000191CB8 | RNA13585 | 1001 | 825 | 0.303096365 | 0.702517551 |
| endo-siRNA | URS0000538EB0 | RNA9362 | 40 | 19 | -0.489700892 | 0.703253759 |
| endo-siRNA | URS000052C8F8 | RNA14018 | 51 | 44 | 0.368164883 | 0.703253759 |
| endo-siRNA | URS00002F946B | RNA2626 | 15 | 15 | 0.577366613 | 0.703253759 |
| endo-siRNA | URS00004DE3B7 | RNA3365 | 15 | 15 | 0.577366613 | 0.703253759 |
| endo-siRNA | URS000018A866 | RNA13253 | 22 | 10 | -0.55081351 | 0.703253759 |
| endo-siRNA | URS0000449835 | RNA5973 | 22 | 10 | -0.55081351 | 0.703253759 |
| endo-siRNA | URS00002125F2 | RNA5666 | 28 | 13 | -0.521435737 | 0.703270938 |
| endo-siRNA | URS00000A1AC7 | RNA10396 | 28 | 13 | -0.521435737 | 0.703270938 |
| endo-siRNA | URS000005BEC5 | RNA2973 | 20 | 19 | 0.504923484 | 0.703270938 |
| endo-siRNA | URS00003B789C | RNA4126 | 20 | 19 | 0.504923484 | 0.703270938 |
| endo-siRNA | URS00001B8A9A | RNA1252 | 60 | 30 | -0.41667948 | 0.703438773 |
| endo-siRNA | URS0000164153 | RNA6046 | 46 | 40 | 0.379392805 | 0.703438773 |
| endo-siRNA | URS000009529B | RNA10620 | 54 | 27 | -0.416545772 | 0.706043576 |
| endo-siRNA | URS00003E4767 | RNA4533 | 73 | 37 | -0.397318485 | 0.706043576 |
| endo-siRNA | URS0000169F43 | RNA902 | 36 | 32 | 0.410700954 | 0.707594634 |
| endo-siRNA | URS0000203EA0 | RNA5189 | 281 | 151 | -0.3137214 | 0.707594634 |
| endo-siRNA | URS00005E980A | RNA10160 | 11 | 4 | -0.861177609 | 0.707594634 |
| endo-siRNA | URS00003E5651 | RNA9440 | 11 | 4 | -0.861177609 | 0.707594634 |
| endo-siRNA | URS00004650C2 | RNA14067 | 11 | 4 | -0.861177609 | 0.707594634 |
| endo-siRNA | URS000005BDC7 | RNA5628 | 11 | 4 | -0.861177609 | 0.707594634 |
| endo-siRNA | URS00004EEDDB | RNA8304 | 11 | 4 | -0.861177609 | 0.707594634 |
| endo-siRNA | URS00002E165C | RNA429 | 11 | 4 | -0.861177609 | 0.707594634 |
| endo-siRNA | URS00005FD789 | RNA5150 | 11 | 4 | -0.861177609 | 0.707594634 |
| endo-siRNA | URS000036724C | RNA13930 | 11 | 4 | -0.861177609 | 0.707594634 |
| endo-siRNA | URS00003C0C11 | RNA4837 | 11 | 4 | -0.861177609 | 0.707594634 |
| endo-siRNA | URS000014E5E4 | RNA10038 | 11 | 4 | -0.861177609 | 0.707594634 |
| endo-siRNA | URS00000D9FE5 | RNA4309 | 11 | 4 | -0.861177609 | 0.707594634 |
| endo-siRNA | URS000032C277 | RNA5301 | 108 | 56 | -0.364843114 | 0.707926004 |
| endo-siRNA | URS00005905F9 | RNA722 | 115 | 60 | -0.355959938 | 0.70990161 |
| endo-siRNA | URS0000311FC8 | RNA5593 | 102 | 86 | 0.335512845 | 0.70990161 |
| endo-siRNA | URS00001E7D06 | RNA1289 | 29 | 14 | -0.465669482 | 0.714722622 |
| endo-siRNA | URS0000221DFE | RNA10698 | 144 | 77 | -0.320651421 | 0.71651885 |
| endo-siRNA | URS0000292E88 | RNA526 | 16 | 16 | 0.577660978 | 0.71651885 |
| endo-siRNA | URS00004388EE | RNA13916 | 11 | 12 | 0.700106051 | 0.71651885 |
| endo-siRNA | URS00003485BF | RNA786 | 11 | 12 | 0.700106051 | 0.71651885 |
| endo-siRNA | URS0000148367 | RNA9282 | 11 | 12 | 0.700106051 | 0.71651885 |
| endo-siRNA | URS00001032D4 | RNA401 | 11 | 12 | 0.700106051 | 0.71651885 |
| endo-siRNA | URS0000527BAB | RNA5230 | 17 | 7 | -0.690170347 | 0.71651885 |
| endo-siRNA | URS00003C3A0A | RNA1281 | 17 | 7 | -0.690170347 | 0.71651885 |
| endo-siRNA | URS00002CCE06 | RNA8258 | 17 | 7 | -0.690170347 | 0.71651885 |
| endo-siRNA | URS00003F021C | RNA757 | 434 | 236 | -0.296681178 | 0.717330346 |
| endo-siRNA | URS00000C4A50 | RNA1014 | 43 | 21 | -0.449992247 | 0.717330346 |
| endo-siRNA | URS000058EBD5 | RNA11642 | 54 | 47 | 0.380885757 | 0.717330346 |
| endo-siRNA | URS00004406BC | RNA7448 | 409 | 222 | -0.299306025 | 0.719632179 |
| endo-siRNA | URS000030BBAF | RNA5192 | 312 | 169 | -0.302248 | 0.720187539 |
| endo-siRNA | URS0000418D14 | RNA9733 | 1525 | 837 | -0.283365695 | 0.721848453 |
| endo-siRNA | URS000058567E | RNA7394 | 37 | 18 | -0.455245217 | 0.722149136 |
| endo-siRNA | URS0000365EB0 | RNA9453 | 36 | 18 | -0.415878526 | 0.722149136 |
| endo-siRNA | URS00004B39C7 | RNA10107 | 38 | 34 | 0.420211425 | 0.722149136 |
| endo-siRNA | URS0000471E4B | RNA9738 | 167 | 90 | -0.309428176 | 0.722149136 |
| endo-siRNA | URS000003CD18 | RNA9779 | 57 | 29 | -0.391612231 | 0.727259285 |
| endo-siRNA | URS000000C409 | RNA9616 | 44 | 22 | -0.416242212 | 0.728842619 |
| endo-siRNA | URS0000343F3B | RNA9415 | 67 | 57 | 0.348222133 | 0.729155121 |
| endo-siRNA | URS00004D7D7C | RNA14102 | 615 | 337 | -0.285647585 | 0.729155121 |
| endo-siRNA | URS000054A767 | RNA9505 | 103 | 86 | 0.321458212 | 0.73192767 |
| endo-siRNA | URS000058EA98 | RNA3508 | 115 | 61 | -0.332152596 | 0.733822007 |
| endo-siRNA | URS00002E955F | RNA775 | 24 | 11 | -0.539327781 | 0.733822007 |
| endo-siRNA | URS00002C83D6 | RNA9754 | 58 | 30 | -0.367893837 | 0.733822007 |
| endo-siRNA | URS00004086A4 | RNA3462 | 58 | 30 | -0.367893837 | 0.733822007 |
| endo-siRNA | URS000036F714 | RNA9755 | 222 | 121 | -0.293219848 | 0.734583789 |
| endo-siRNA | URS00001C5966 | RNA9693 | 230 | 125 | -0.297377297 | 0.734903367 |
| endo-siRNA | URS00002E833F | RNA9279 | 116 | 97 | 0.323670526 | 0.735402328 |
| endo-siRNA | URS00001E5EC1 | RNA11398 | 66 | 34 | -0.373848571 | 0.735402328 |
| endo-siRNA | URS000059344A | RNA14329 | 66 | 34 | -0.373848571 | 0.735402328 |
| endo-siRNA | URS0000616C97 | RNA5400 | 18 | 8 | -0.581831958 | 0.735402328 |
| endo-siRNA | URS000011BDD9 | RNA9627 | 18 | 8 | -0.581831958 | 0.735402328 |
| endo-siRNA | URS0000473E95 | RNA26 | 18 | 8 | -0.581831958 | 0.735402328 |
| endo-siRNA | URS00004B275D | RNA2856 | 12 | 12 | 0.576192185 | 0.735402328 |
| endo-siRNA | URS00001E1763 | RNA6032 | 12 | 12 | 0.576192185 | 0.735402328 |
| endo-siRNA | URS00001A243A | RNA5617 | 12 | 12 | 0.576192185 | 0.735402328 |
| endo-siRNA | URS000017A05E | RNA9490 | 13 | 12 | 0.462084629 | 0.735402328 |
| endo-siRNA | URS000044E0BE | RNA3294 | 88 | 46 | -0.353074923 | 0.735402328 |
| endo-siRNA | URS0000578812 | RNA5920 | 12 | 5 | -0.670226315 | 0.735402328 |
| endo-siRNA | URS000031C4DF | RNA13040 | 12 | 5 | -0.670226315 | 0.735402328 |
| endo-siRNA | URS000019B6CB | RNA8582 | 12 | 5 | -0.670226315 | 0.735402328 |
| endo-siRNA | URS000018189F | RNA14070 | 12 | 5 | -0.670226315 | 0.735402328 |
| endo-siRNA | URS000024E143 | RNA3523 | 12 | 5 | -0.670226315 | 0.735402328 |
| endo-siRNA | URS000031B5E7 | RNA14177 | 12 | 5 | -0.670226315 | 0.735402328 |
| endo-siRNA | URS0000377A71 | RNA10147 | 12 | 5 | -0.670226315 | 0.735402328 |
| endo-siRNA | URS00002E0CAD | RNA6069 | 110 | 59 | -0.316121767 | 0.736480769 |
| endo-siRNA | URS0000210E17 | RNA13531 | 32 | 15 | -0.508140299 | 0.736480769 |
| endo-siRNA | URS00000AF9B3 | RNA6045 | 47 | 40 | 0.348465603 | 0.737159924 |
| endo-siRNA | URS00004E45F8 | RNA9692 | 139 | 75 | -0.307639344 | 0.737690078 |
| endo-siRNA | URS0000195103 | RNA11271 | 198 | 162 | 0.292406288 | 0.738712654 |
| endo-siRNA | URS0000486DB8 | RNA9887 | 338 | 185 | -0.287243783 | 0.739123357 |
| endo-siRNA | URS0000434DAF | RNA5241 | 36 | 31 | 0.365042408 | 0.742301327 |
| endo-siRNA | URS00005FB0B2 | RNA5534 | 227 | 184 | 0.278959249 | 0.742301327 |
| endo-siRNA | URS00003A504D | RNA14074 | 26 | 12 | -0.529669982 | 0.742301327 |
| endo-siRNA | URS000042491B | RNA6008 | 26 | 12 | -0.529669982 | 0.742301327 |
| endo-siRNA | URS000061D0E5 | RNA3058 | 18 | 17 | 0.496159444 | 0.742301327 |
| endo-siRNA | URS0000326F9D | RNA9684 | 18 | 17 | 0.496159444 | 0.742301327 |
| endo-siRNA | URS00003C8A32 | RNA5231 | 18 | 17 | 0.496159444 | 0.742301327 |
| endo-siRNA | URS00004B828D | RNA10861 | 18 | 17 | 0.496159444 | 0.742301327 |
| endo-siRNA | URS00005C60BD | RNA13255 | 25 | 12 | -0.473417176 | 0.742301327 |
| endo-siRNA | URS0000130AB3 | RNA5087 | 25 | 12 | -0.473417176 | 0.742301327 |
| endo-siRNA | URS00003B6F29 | RNA5970 | 66 | 56 | 0.344378494 | 0.742301327 |
| endo-siRNA | URS000006A371 | RNA9471 | 188 | 103 | -0.285723143 | 0.747474603 |
| endo-siRNA | URS00002FBDE7 | RNA9797 | 91 | 49 | -0.310401027 | 0.748441522 |
| endo-siRNA | URS00002B71C6 | RNA5163 | 77 | 41 | -0.32640814 | 0.748936979 |
| endo-siRNA | URS00004431FE | RNA833 | 407 | 225 | -0.272879919 | 0.748936979 |
| endo-siRNA | URS00001D2C8C | RNA11951 | 48 | 24 | -0.416378759 | 0.749341896 |
| endo-siRNA | URS000017E71E | RNA12320 | 47 | 24 | -0.386100656 | 0.749341896 |
| endo-siRNA | URS000059703D | RNA5332 | 20 | 9 | -0.564700866 | 0.750515692 |
| endo-siRNA | URS00004684AE | RNA5028 | 20 | 9 | -0.564700866 | 0.750515692 |
| endo-siRNA | URS000048BB54 | RNA6087 | 20 | 9 | -0.564700866 | 0.750515692 |
| endo-siRNA | URS000047551F | RNA2272 | 19 | 9 | -0.491264976 | 0.750515692 |
| endo-siRNA | URS000010BDD6 | RNA6012 | 19 | 9 | -0.491264976 | 0.750515692 |
| endo-siRNA | URS000060C292 | RNA5984 | 19 | 9 | -0.491264976 | 0.750515692 |
| endo-siRNA | URS000014D194 | RNA3896 | 19 | 9 | -0.491264976 | 0.750515692 |
| endo-siRNA | URS000051CF5F | RNA10699 | 14 | 13 | 0.470903142 | 0.750515692 |
| endo-siRNA | URS0000314AEA | RNA9740 | 138 | 75 | -0.297233988 | 0.751012034 |
| endo-siRNA | URS000000CFED | RNA615 | 161 | 88 | -0.289074016 | 0.75222921 |
| endo-siRNA | URS0000309689 | RNA12329 | 27 | 13 | -0.469252789 | 0.755142758 |
| endo-siRNA | URS0000003BD3 | RNA7358 | 27 | 13 | -0.469252789 | 0.755142758 |
| endo-siRNA | URS0000488951 | RNA8574 | 20 | 18 | 0.427341038 | 0.755142758 |
| endo-siRNA | URS00001F6FA1 | RNA5983 | 20 | 18 | 0.427341038 | 0.755142758 |
| endo-siRNA | URS000007E8D0 | RNA150 | 20 | 18 | 0.427341038 | 0.755142758 |
| endo-siRNA | URS00001C1CB8 | RNA14012 | 2390 | 1901 | 0.251846668 | 0.755142758 |
| endo-siRNA | URS000038A341 | RNA9607 | 49 | 25 | -0.387380515 | 0.755142758 |
| endo-siRNA | URS000022F39A | RNA1224 | 13 | 6 | -0.526027371 | 0.757664683 |
| endo-siRNA | URS00005F48EE | RNA4157 | 13 | 6 | -0.526027371 | 0.757664683 |
| endo-siRNA | URS00001ADBC2 | RNA7849 | 13 | 6 | -0.526027371 | 0.757664683 |
| endo-siRNA | URS00001273D6 | RNA1190 | 13 | 6 | -0.526027371 | 0.757664683 |
| endo-siRNA | URS0000353F7C | RNA3193 | 13 | 6 | -0.526027371 | 0.757664683 |
| endo-siRNA | URS000015A833 | RNA5319 | 13 | 6 | -0.526027371 | 0.757664683 |
| endo-siRNA | URS0000314089 | RNA9396 | 188 | 104 | -0.271797423 | 0.759114538 |
| endo-siRNA | URS00004D8FE3 | RNA836 | 81 | 43 | -0.330783905 | 0.762282876 |
| endo-siRNA | URS00001AC9C4 | RNA12343 | 81 | 43 | -0.330783905 | 0.762282876 |
| endo-siRNA | URS000015E056 | RNA8987 | 80 | 43 | -0.312895304 | 0.762282876 |
| endo-siRNA | URS00000B6CB7 | RNA756 | 606 | 339 | -0.255850035 | 0.762656906 |
| endo-siRNA | URS00003007F6 | RNA10462 | 53 | 44 | 0.312829305 | 0.76691261 |
| endo-siRNA | URS00000B74DE | RNA4793 | 21 | 10 | -0.48416411 | 0.76691261 |
| endo-siRNA | URS000011B481 | RNA11788 | 21 | 10 | -0.48416411 | 0.76691261 |
| endo-siRNA | URS00003118C1 | RNA4794 | 21 | 10 | -0.48416411 | 0.76691261 |
| endo-siRNA | URS00002C0985 | RNA5980 | 21 | 10 | -0.48416411 | 0.76691261 |
| endo-siRNA | URS00003D2345 | RNA155 | 21 | 10 | -0.48416411 | 0.76691261 |
| endo-siRNA | URS000051C33C | RNA8609 | 201 | 111 | -0.274297286 | 0.76691261 |
| endo-siRNA | URS00000A7E66 | RNA14020 | 21 | 19 | 0.435045258 | 0.76691261 |
| endo-siRNA | URS00001E0AF2 | RNA11266 | 28 | 14 | -0.415308312 | 0.76691261 |
| endo-siRNA | URS00004E42FE | RNA7504 | 28 | 14 | -0.415308312 | 0.76691261 |
| endo-siRNA | URS000020F4AD | RNA3471 | 90 | 49 | -0.294485841 | 0.76883916 |
| endo-siRNA | URS000004F51B | RNA13672 | 44 | 23 | -0.352396065 | 0.773628325 |
| endo-siRNA | URS00002A1E5B | RNA9338 | 60 | 32 | -0.323870024 | 0.77485672 |
| endo-siRNA | URS00001E4373 | RNA7327 | 1091 | 617 | -0.240159562 | 0.776650417 |
| endo-siRNA | URS00001084BE | RNA5776 | 37 | 19 | -0.377662771 | 0.776984055 |
| endo-siRNA | URS000026424F | RNA4345 | 37 | 19 | -0.377662771 | 0.776984055 |
| endo-siRNA | URS0000608554 | RNA1189 | 84 | 46 | -0.286077533 | 0.776984055 |
| endo-siRNA | URS00004FD848 | RNA12460 | 22 | 20 | 0.442018276 | 0.776984055 |
| endo-siRNA | URS0000303748 | RNA617 | 110 | 60 | -0.291914955 | 0.776984055 |
| endo-siRNA | URS00003855E2 | RNA3429 | 167 | 93 | -0.262174176 | 0.776984055 |
| endo-siRNA | URS00005A9EE7 | RNA10686 | 15 | 6 | -0.730284082 | 0.776984055 |
| endo-siRNA | URS00001CE94B | RNA1308 | 15 | 6 | -0.730284082 | 0.776984055 |
| endo-siRNA | URS000056B73C | RNA5470 | 15 | 6 | -0.730284082 | 0.776984055 |
| endo-siRNA | URS00005B851A | RNA14030 | 15 | 6 | -0.730284082 | 0.776984055 |
| endo-siRNA | URS00005BC3BB | RNA502 | 15 | 6 | -0.730284082 | 0.776984055 |
| endo-siRNA | URS00001767E6 | RNA1086 | 15 | 6 | -0.730284082 | 0.776984055 |
| endo-siRNA | URS000024153E | RNA3939 | 15 | 6 | -0.730284082 | 0.776984055 |
| endo-siRNA | URS000038D648 | RNA5876 | 15 | 6 | -0.730284082 | 0.776984055 |
| endo-siRNA | URS00001157F7 | RNA5573 | 15 | 6 | -0.730284082 | 0.776984055 |
| endo-siRNA | URS0000137B4F | RNA13056 | 15 | 6 | -0.730284082 | 0.776984055 |
| endo-siRNA | URS0000457EC4 | RNA9285 | 14 | 6 | -0.631767544 | 0.776984055 |
| endo-siRNA | URS0000297E46 | RNA9452 | 14 | 6 | -0.631767544 | 0.776984055 |
| endo-siRNA | URS00003A43DE | RNA9901 | 14 | 6 | -0.631767544 | 0.776984055 |
| endo-siRNA | URS00003925DF | RNA6772 | 14 | 6 | -0.631767544 | 0.776984055 |
| endo-siRNA | URS000021CDF5 | RNA4125 | 14 | 6 | -0.631767544 | 0.776984055 |
| endo-siRNA | URS000032CC9F | RNA5875 | 14 | 6 | -0.631767544 | 0.776984055 |
| endo-siRNA | URS00001308DA | RNA3627 | 14 | 6 | -0.631767544 | 0.776984055 |
| endo-siRNA | URS000013F29D | RNA5566 | 14 | 6 | -0.631767544 | 0.776984055 |
| endo-siRNA | URS00001C723A | RNA8979 | 14 | 6 | -0.631767544 | 0.776984055 |
| endo-siRNA | URS000042C08E | RNA2918 | 14 | 6 | -0.631767544 | 0.776984055 |
| endo-siRNA | URS00002FC6B7 | RNA3056 | 10 | 10 | 0.575022588 | 0.776984055 |
| endo-siRNA | URS000033258A | RNA7611 | 36 | 30 | 0.317891497 | 0.776984055 |
| endo-siRNA | URS000056E284 | RNA12221 | 16 | 15 | 0.485149543 | 0.77825476 |
| endo-siRNA | URS000027102A | RNA3548 | 56 | 47 | 0.328560959 | 0.77858464 |
| endo-siRNA | URS00005D31F8 | RNA6039 | 46 | 24 | -0.355173455 | 0.786498605 |
| endo-siRNA | URS000059514B | RNA8223 | 65 | 53 | 0.287065774 | 0.786498605 |
| endo-siRNA | URS000057E4D3 | RNA4095 | 31 | 15 | -0.462553554 | 0.786866805 |
| endo-siRNA | URS00000AA340 | RNA1301 | 31 | 15 | -0.462553554 | 0.786866805 |
| endo-siRNA | URS00001CDAA2 | RNA9776 | 31 | 15 | -0.462553554 | 0.786866805 |
| endo-siRNA | URS0000465E79 | RNA12586 | 23 | 21 | 0.448359542 | 0.786866805 |
| endo-siRNA | URS000058F582 | RNA13230 | 23 | 21 | 0.448359542 | 0.786866805 |
| endo-siRNA | URS000023E3D0 | RNA13418 | 30 | 15 | -0.415479211 | 0.786866805 |
| endo-siRNA | URS0000300468 | RNA11305 | 72 | 39 | -0.301710798 | 0.789736493 |
| endo-siRNA | URS0000013C08 | RNA4235 | 1162 | 908 | 0.226240372 | 0.791169479 |
| endo-siRNA | URS0000116E79 | RNA10985 | 31 | 26 | 0.326942931 | 0.791169479 |
| endo-siRNA | URS00003AB868 | RNA9718 | 31 | 26 | 0.326942931 | 0.791169479 |
| endo-siRNA | URS0000581585 | RNA8652 | 17 | 16 | 0.490985949 | 0.792201324 |
| endo-siRNA | URS00003C3B0A | RNA8671 | 23 | 11 | -0.47831648 | 0.792201324 |
| endo-siRNA | URS000047050F | RNA5375 | 23 | 11 | -0.47831648 | 0.792201324 |
| endo-siRNA | URS0000121095 | RNA5455 | 46 | 38 | 0.305581789 | 0.792917581 |
| endo-siRNA | URS0000095340 | RNA493 | 46 | 38 | 0.305581789 | 0.792917581 |
| endo-siRNA | URS000006F971 | RNA3493 | 73 | 40 | -0.28513578 | 0.793845071 |
| endo-siRNA | URS0000245F3D | RNA854 | 16 | 7 | -0.603495318 | 0.795198638 |
| endo-siRNA | URS0000361476 | RNA14327 | 16 | 7 | -0.603495318 | 0.795198638 |
| endo-siRNA | URS00001E11B7 | RNA12524 | 16 | 7 | -0.603495318 | 0.795198638 |
| endo-siRNA | URS00001DD587 | RNA788 | 16 | 7 | -0.603495318 | 0.795198638 |
| endo-siRNA | URS0000577550 | RNA14294 | 16 | 7 | -0.603495318 | 0.795198638 |
| endo-siRNA | URS00000B209D | RNA2406 | 16 | 7 | -0.603495318 | 0.795198638 |
| endo-siRNA | URS000010B3EC | RNA5919 | 11 | 11 | 0.575659953 | 0.795198638 |
| endo-siRNA | URS000023818C | RNA11994 | 11 | 11 | 0.575659953 | 0.795198638 |
| endo-siRNA | URS00001183B6 | RNA4594 | 11 | 11 | 0.575659953 | 0.795198638 |
| endo-siRNA | URS0000397350 | RNA2537 | 11 | 11 | 0.575659953 | 0.795198638 |
| endo-siRNA | URS00005DF6AF | RNA9704 | 32 | 16 | -0.415628864 | 0.795198638 |
| endo-siRNA | URS000051DA4B | RNA5774 | 25 | 21 | 0.328812748 | 0.795198638 |
| endo-siRNA | URS00002B4DFE | RNA592 | 83 | 46 | -0.268830501 | 0.796513736 |
| endo-siRNA | URS000044059C | RNA9193 | 40 | 21 | -0.34603138 | 0.796616665 |
| endo-siRNA | URS00005D9F7B | RNA610 | 159 | 126 | 0.246296796 | 0.797121156 |
| endo-siRNA | URS00002D036C | RNA12870 | 164 | 92 | -0.251625544 | 0.802074099 |
| endo-siRNA | URS000034A5EF | RNA12869 | 63 | 51 | 0.276659858 | 0.802656016 |
| endo-siRNA | URS000038B46B | RNA5211 | 63 | 51 | 0.276659858 | 0.802656016 |
| endo-siRNA | URS0000493C43 | RNA2412 | 103 | 57 | -0.271063272 | 0.803823258 |
| endo-siRNA | URS00001DD21D | RNA4358 | 58 | 32 | -0.275084381 | 0.803823258 |
| endo-siRNA | URS000029D25A | RNA4103 | 24 | 12 | -0.414881683 | 0.803823258 |
| endo-siRNA | URS00003AEC47 | RNA5214 | 24 | 12 | -0.414881683 | 0.803823258 |
| endo-siRNA | URS000025CEC7 | RNA6665 | 40 | 34 | 0.346494271 | 0.804794917 |
| endo-siRNA | URS00000D809A | RNA2417 | 50 | 27 | -0.305833884 | 0.804794917 |
| endo-siRNA | URS000053EA84 | RNA939 | 26 | 22 | 0.339362946 | 0.805755388 |
| endo-siRNA | URS0000410938 | RNA1184 | 56 | 46 | 0.297600729 | 0.809052661 |
| endo-siRNA | URS000042B889 | RNA1251 | 57 | 46 | 0.2721332 | 0.809052661 |
| endo-siRNA | URS00004E71B3 | RNA2526 | 78 | 43 | -0.276438602 | 0.812788149 |
| endo-siRNA | URS00004528EB | RNA9703 | 51 | 28 | -0.282041435 | 0.812788149 |
| endo-siRNA | URS00005D7EED | RNA7410 | 51 | 28 | -0.282041435 | 0.812788149 |
| endo-siRNA | URS00001ED148 | RNA3549 | 179 | 101 | -0.243269154 | 0.813047816 |
| endo-siRNA | URS00005F3995 | RNA8272 | 34 | 29 | 0.351257704 | 0.813543921 |
| endo-siRNA | URS00004B0E8C | RNA14374 | 35 | 29 | 0.309618451 | 0.813543921 |
| endo-siRNA | URS00005D23C6 | RNA6660 | 17 | 8 | -0.500070437 | 0.813543921 |
| endo-siRNA | URS00000AB593 | RNA5173 | 17 | 8 | -0.500070437 | 0.813543921 |
| endo-siRNA | URS000009C57E | RNA2562 | 17 | 8 | -0.500070437 | 0.813543921 |
| endo-siRNA | URS0000527D45 | RNA649 | 17 | 8 | -0.500070437 | 0.813543921 |
| endo-siRNA | URS00001E8A2A | RNA8739 | 17 | 8 | -0.500070437 | 0.813543921 |
| endo-siRNA | URS00005AE6F8 | RNA594 | 17 | 8 | -0.500070437 | 0.813543921 |
| endo-siRNA | URS000016C240 | RNA2587 | 17 | 8 | -0.500070437 | 0.813543921 |
| endo-siRNA | URS000039E2AA | RNA10041 | 27 | 23 | 0.3490676 | 0.814425667 |
| endo-siRNA | URS000060AE47 | RNA4275 | 34 | 18 | -0.333768181 | 0.814425667 |
| endo-siRNA | URS000022EFC7 | RNA9788 | 34 | 18 | -0.333768181 | 0.814425667 |
| endo-siRNA | URS0000341F20 | RNA676 | 66 | 53 | 0.265089756 | 0.814425667 |
| endo-siRNA | URS00006163B6 | RNA7383 | 79 | 44 | -0.26169141 | 0.814425667 |
| endo-siRNA | URS0000554951 | RNA9764 | 339 | 194 | -0.223008743 | 0.814425667 |
| endo-siRNA | URS00002D1815 | RNA4082 | 26 | 13 | -0.415111297 | 0.814425667 |
| endo-siRNA | URS0000524971 | RNA13705 | 25 | 13 | -0.35885849 | 0.814425667 |
| endo-siRNA | URS00003D235F | RNA14291 | 25 | 13 | -0.35885849 | 0.814425667 |
| endo-siRNA | URS000039D46E | RNA1315 | 20 | 17 | 0.345348218 | 0.814425667 |
| endo-siRNA | URS000026ECA7 | RNA10145 | 220 | 126 | -0.221803189 | 0.814974249 |
| endo-siRNA | URS0000005A74 | RNA9499 | 10 | 4 | -0.725612116 | 0.814974249 |
| endo-siRNA | URS000002C2C0 | RNA5929 | 10 | 4 | -0.725612116 | 0.814974249 |
| endo-siRNA | URS00000032D6 | RNA3923 | 10 | 4 | -0.725612116 | 0.814974249 |
| endo-siRNA | URS00004F581C | RNA4448 | 10 | 4 | -0.725612116 | 0.814974249 |
| endo-siRNA | URS000031AB67 | RNA11704 | 10 | 4 | -0.725612116 | 0.814974249 |
| endo-siRNA | URS0000109E7D | RNA6172 | 10 | 4 | -0.725612116 | 0.814974249 |
| endo-siRNA | URS00005BCB77 | RNA8564 | 10 | 4 | -0.725612116 | 0.814974249 |
| endo-siRNA | URS00001822D0 | RNA5444 | 10 | 4 | -0.725612116 | 0.814974249 |
| endo-siRNA | URS000015855A | RNA10496 | 10 | 4 | -0.725612116 | 0.814974249 |
| endo-siRNA | URS00000AFB26 | RNA2970 | 10 | 4 | -0.725612116 | 0.814974249 |
| endo-siRNA | URS00002A0826 | RNA6562 | 10 | 4 | -0.725612116 | 0.814974249 |
| endo-siRNA | URS00005FA80E | RNA13867 | 10 | 4 | -0.725612116 | 0.814974249 |
| endo-siRNA | URS00004F5EE4 | RNA2627 | 28 | 24 | 0.35802464 | 0.819798859 |
| endo-siRNA | URS000013FC75 | RNA7239 | 35 | 19 | -0.297824988 | 0.819798859 |
| endo-siRNA | URS00001E753A | RNA1186 | 35 | 19 | -0.297824988 | 0.819798859 |
| endo-siRNA | URS00004EA742 | RNA4116 | 35 | 19 | -0.297824988 | 0.819798859 |
| endo-siRNA | URS00004E7952 | RNA8914 | 27 | 14 | -0.363125364 | 0.822573164 |
| endo-siRNA | URS0000314ACD | RNA5678 | 27 | 14 | -0.363125364 | 0.822573164 |
| endo-siRNA | URS0000368D09 | RNA9816 | 27 | 14 | -0.363125364 | 0.822573164 |
| endo-siRNA | URS000053369C | RNA7370 | 21 | 18 | 0.357462812 | 0.822573164 |
| endo-siRNA | URS000054C100 | RNA1206 | 21 | 18 | 0.357462812 | 0.822573164 |
| endo-siRNA | URS0000586F2A | RNA14056 | 179 | 102 | -0.229069302 | 0.822573164 |
| endo-siRNA | URS000010296E | RNA11936 | 45 | 24 | -0.323568713 | 0.822858751 |
| endo-siRNA | URS00001D1C05 | RNA2080 | 18 | 9 | -0.41388964 | 0.823197935 |
| endo-siRNA | URS0000010898 | RNA4156 | 14 | 12 | 0.356344456 | 0.823197935 |
| endo-siRNA | URS000015C5C8 | RNA12144 | 14 | 12 | 0.356344456 | 0.823197935 |
| endo-siRNA | URS00002ABC29 | RNA14013 | 1332 | 1020 | 0.19706759 | 0.824171286 |
| endo-siRNA | URS00000DF333 | RNA959 | 63 | 50 | 0.248147237 | 0.825446161 |
| endo-siRNA | URS000032993D | RNA10115 | 63 | 50 | 0.248147237 | 0.825446161 |
| endo-siRNA | URS00002B3AEA | RNA7376 | 114 | 64 | -0.250417186 | 0.826750513 |
| endo-siRNA | URS00000F04F9 | RNA9648 | 263 | 152 | -0.208750221 | 0.829995382 |
| endo-siRNA | URS000037725C | RNA1338 | 22 | 19 | 0.368395858 | 0.831469998 |
| endo-siRNA | URS00001FDB83 | RNA10616 | 97 | 55 | -0.236043567 | 0.833751234 |
| endo-siRNA | URS0000462429 | RNA495 | 188 | 145 | 0.207279564 | 0.834030939 |
| endo-siRNA | URS00005B716C | RNA9685 | 57 | 45 | 0.240493965 | 0.834201444 |
| endo-siRNA | URS0000014E79 | RNA10144 | 48 | 26 | -0.301362642 | 0.834201444 |
| endo-siRNA | URS000016E693 | RNA9581 | 39 | 32 | 0.295683931 | 0.834201444 |
| endo-siRNA | URS0000589B6F | RNA3940 | 47 | 26 | -0.27108454 | 0.834201444 |
| endo-siRNA | URS000057DE7D | RNA4520 | 47 | 26 | -0.27108454 | 0.834201444 |
| endo-siRNA | URS000052B49E | RNA9780 | 6876 | 4033 | -0.187598525 | 0.834260968 |
| endo-siRNA | URS00003C2A78 | RNA6671 | 15 | 13 | 0.372386604 | 0.834881262 |
| endo-siRNA | URS00000F23C5 | RNA3316 | 15 | 13 | 0.372386604 | 0.834881262 |
| endo-siRNA | URS00003FA5A0 | RNA4830 | 19 | 10 | -0.340849994 | 0.834881262 |
| endo-siRNA | URS000009954E | RNA974 | 11 | 5 | -0.546312449 | 0.834881262 |
| endo-siRNA | URS0000343EA1 | RNA3938 | 11 | 5 | -0.546312449 | 0.834881262 |
| endo-siRNA | URS0000442DBA | RNA13246 | 11 | 5 | -0.546312449 | 0.834881262 |
| endo-siRNA | URS00005DDC65 | RNA7369 | 11 | 5 | -0.546312449 | 0.834881262 |
| endo-siRNA | URS00001F9E0A | RNA1942 | 11 | 5 | -0.546312449 | 0.834881262 |
| endo-siRNA | URS0000358BC2 | RNA8569 | 11 | 5 | -0.546312449 | 0.834881262 |
| endo-siRNA | URS000020C2DE | RNA952 | 11 | 5 | -0.546312449 | 0.834881262 |
| endo-siRNA | URS00002391F0 | RNA5616 | 11 | 5 | -0.546312449 | 0.834881262 |
| endo-siRNA | URS00002B3FB0 | RNA7520 | 11 | 5 | -0.546312449 | 0.834881262 |
| endo-siRNA | URS000016778F | RNA3464 | 11 | 5 | -0.546312449 | 0.834881262 |
| endo-siRNA | URS0000192233 | RNA7797 | 84 | 66 | 0.233804866 | 0.836426582 |
| endo-siRNA | URS0000025843 | RNA12133 | 40 | 33 | 0.3035539 | 0.836840923 |
| endo-siRNA | URS000004AA6E | RNA9332 | 23 | 20 | 0.378312448 | 0.836840923 |
| endo-siRNA | URS0000549CFC | RNA5194 | 29 | 15 | -0.366816898 | 0.836840923 |
| endo-siRNA | URS00000EE530 | RNA3375 | 29 | 15 | -0.366816898 | 0.836840923 |
| endo-siRNA | URS0000437EAD | RNA13 | 24 | 20 | 0.317301146 | 0.836840923 |
| endo-siRNA | URS000050FEDA | RNA6825 | 32 | 26 | 0.281356186 | 0.837035839 |
| endo-siRNA | URS0000201157 | RNA11262 | 533 | 312 | -0.190419015 | 0.838241498 |
| endo-siRNA | URS00000C1077 | RNA14100 | 669 | 392 | -0.188994069 | 0.838995395 |
| endo-siRNA | URS00005A25C5 | RNA3396 | 60 | 33 | -0.279612265 | 0.840741969 |
| endo-siRNA | URS000012F597 | RNA9570 | 50 | 40 | 0.259473456 | 0.840741969 |
| endo-siRNA | URS00000059C7 | RNA10426 | 195 | 113 | -0.204868899 | 0.840741969 |
| endo-siRNA | URS000013775E | RNA3415 | 102 | 58 | -0.231961272 | 0.841627098 |
| endo-siRNA | URS0000350125 | RNA529 | 87 | 68 | 0.226271853 | 0.841627098 |
| endo-siRNA | URS00003B818D | RNA9886 | 50 | 28 | -0.253556829 | 0.842169317 |
| endo-siRNA | URS000005D88E | RNA14063 | 124 | 71 | -0.222045768 | 0.844304874 |
| endo-siRNA | URS0000481FC9 | RNA906 | 16 | 14 | 0.386296959 | 0.845214027 |
| endo-siRNA | URS000025477E | RNA7550 | 31 | 16 | -0.370042119 | 0.845214027 |
| endo-siRNA | URS00003726CB | RNA11972 | 30 | 16 | -0.322967776 | 0.845214027 |
| endo-siRNA | URS00004D98CF | RNA7449 | 443 | 260 | -0.186614424 | 0.845463404 |
| endo-siRNA | URS00004C5717 | RNA7525 | 61 | 48 | 0.235803993 | 0.846903136 |
| endo-siRNA | URS000023A08E | RNA5178 | 35 | 28 | 0.259169637 | 0.849900744 |
| endo-siRNA | URS0000593A50 | RNA5120 | 41 | 23 | -0.250874775 | 0.849900744 |
| endo-siRNA | URS000058B698 | RNA9819 | 41 | 23 | -0.250874775 | 0.849900744 |
| endo-siRNA | URS00001ED66F | RNA5249 | 1391 | 1049 | 0.174987748 | 0.850815758 |
| endo-siRNA | URS000031A431 | RNA9815 | 95 | 55 | -0.206033191 | 0.850815758 |
| endo-siRNA | URS00004B4ED9 | RNA4395 | 44 | 35 | 0.251179584 | 0.850815758 |
| endo-siRNA | URS00005D3B7F | RNA6038 | 32 | 17 | -0.328693848 | 0.850815758 |
| endo-siRNA | URS00003A4D6A | RNA12467 | 26 | 21 | 0.272559942 | 0.850815758 |
| endo-siRNA | URS0000314B19 | RNA806 | 26 | 21 | 0.272559942 | 0.850815758 |
| endo-siRNA | URS000042B34B | RNA12119 | 1510 | 894 | -0.174070203 | 0.850815758 |
| endo-siRNA | URS0000479394 | RNA10449 | 64 | 36 | -0.24732978 | 0.850815758 |
| endo-siRNA | URS00005CFBA5 | RNA5162 | 12 | 6 | -0.411919816 | 0.850815758 |
| endo-siRNA | URS00001381F8 | RNA12050 | 12 | 6 | -0.411919816 | 0.850815758 |
| endo-siRNA | URS0000366288 | RNA6663 | 12 | 6 | -0.411919816 | 0.850815758 |
| endo-siRNA | URS0000204FC0 | RNA11319 | 12 | 6 | -0.411919816 | 0.850815758 |
| endo-siRNA | URS0000578C83 | RNA1824 | 12 | 6 | -0.411919816 | 0.850815758 |
| endo-siRNA | URS00005B7EC0 | RNA8984 | 12 | 6 | -0.411919816 | 0.850815758 |
| endo-siRNA | URS000010CBF7 | RNA6086 | 12 | 6 | -0.411919816 | 0.850815758 |
| endo-siRNA | URS00005D9FCE | RNA6938 | 12 | 6 | -0.411919816 | 0.850815758 |
| endo-siRNA | URS00005E5EEF | RNA3501 | 12 | 6 | -0.411919816 | 0.850815758 |
| endo-siRNA | URS00000730D2 | RNA6031 | 22 | 11 | -0.414610652 | 0.853593923 |
| endo-siRNA | URS00001EAD9B | RNA5224 | 17 | 15 | 0.398474514 | 0.853593923 |
| endo-siRNA | URS00000D5744 | RNA916 | 33 | 18 | -0.290891322 | 0.859734766 |
| endo-siRNA | URS00000DB877 | RNA1249 | 77 | 44 | -0.224768161 | 0.86002642 |
| endo-siRNA | URS0000292152 | RNA9472 | 190 | 111 | -0.193163437 | 0.860147384 |
| endo-siRNA | URS00004FEA64 | RNA13263 | 44 | 24 | -0.291256077 | 0.860147384 |
| endo-siRNA | URS000035ED4E | RNA12756 | 58 | 45 | 0.21546822 | 0.863113335 |
| endo-siRNA | URS000009A5E9 | RNA9015 | 18 | 16 | 0.409224428 | 0.863113335 |
| endo-siRNA | URS0000409449 | RNA6618 | 18 | 16 | 0.409224428 | 0.863113335 |
| endo-siRNA | URS00001D0136 | RNA5460 | 18 | 16 | 0.409224428 | 0.863113335 |
| endo-siRNA | URS0000347384 | RNA13928 | 18 | 16 | 0.409224428 | 0.863113335 |
| endo-siRNA | URS00003F371D | RNA3524 | 23 | 12 | -0.353870382 | 0.863113335 |
| endo-siRNA | URS000058BB67 | RNA13671 | 23 | 12 | -0.353870382 | 0.863113335 |
| endo-siRNA | URS0000290DC9 | RNA8517 | 23 | 12 | -0.353870382 | 0.863113335 |
| endo-siRNA | URS000020BE37 | RNA5242 | 19 | 16 | 0.331849092 | 0.863113335 |
| endo-siRNA | URS00001BCBF0 | RNA9691 | 56 | 32 | -0.224591107 | 0.863189369 |
| endo-siRNA | URS00005764B8 | RNA14087 | 38 | 30 | 0.240203839 | 0.863906246 |
| endo-siRNA | URS0000053E50 | RNA12430 | 34 | 19 | -0.256185734 | 0.863906246 |
| endo-siRNA | URS000005A8CC | RNA7263 | 10 | 9 | 0.424607606 | 0.866196539 |
| endo-siRNA | URS00002E3C7E | RNA6002 | 10 | 9 | 0.424607606 | 0.866196539 |
| endo-siRNA | URS00003412CA | RNA2497 | 10 | 9 | 0.424607606 | 0.866196539 |
| endo-siRNA | URS00000FEF49 | RNA1199 | 234 | 138 | -0.179604503 | 0.867583902 |
| endo-siRNA | URS0000210D1A | RNA12126 | 46 | 26 | -0.240157338 | 0.868074148 |
| endo-siRNA | URS00002948B7 | RNA9466 | 70 | 40 | -0.224720919 | 0.868585556 |
| endo-siRNA | URS0000167F4C | RNA13260 | 105 | 61 | -0.201086894 | 0.869353088 |
| endo-siRNA | URS000004019E | RNA5775 | 29 | 24 | 0.30766347 | 0.869741749 |
| endo-siRNA | URS00002C37E8 | RNA5031 | 30 | 24 | 0.259001157 | 0.869741749 |
| endo-siRNA | URS00001BB55D | RNA7548 | 58 | 33 | -0.230826622 | 0.870022092 |
| endo-siRNA | URS0000052270 | RNA5442 | 58 | 33 | -0.230826622 | 0.870022092 |
| endo-siRNA | URS000014D50E | RNA987 | 24 | 13 | -0.300322998 | 0.870190738 |
| endo-siRNA | URS00001CA234 | RNA12135 | 48 | 27 | -0.247119839 | 0.873037916 |
| endo-siRNA | URS00004C1A57 | RNA9580 | 41 | 32 | 0.223803595 | 0.873037916 |
| endo-siRNA | URS00003D0A40 | RNA14082 | 167 | 99 | -0.172070397 | 0.873610351 |
| endo-siRNA | URS00003E9FF6 | RNA10617 | 36 | 20 | -0.264673662 | 0.876597449 |
| endo-siRNA | URS00006100C7 | RNA1272 | 36 | 20 | -0.264673662 | 0.876597449 |
| endo-siRNA | URS0000077A27 | RNA1231 | 121 | 71 | -0.186755877 | 0.876597449 |
| endo-siRNA | URS00001D3376 | RNA2427 | 42 | 33 | 0.233421055 | 0.876597449 |
| endo-siRNA | URS000032E24D | RNA9550 | 307 | 183 | -0.164196855 | 0.876597449 |
| endo-siRNA | URS000001F478 | RNA9739 | 61 | 35 | -0.218760554 | 0.876597449 |
| endo-siRNA | URS0000025C7B | RNA611 | 97 | 74 | 0.191376861 | 0.876597449 |
| endo-siRNA | URS00005B1259 | RNA7708 | 21 | 17 | 0.275469992 | 0.876597449 |
| endo-siRNA | URS0000086E6E | RNA12142 | 820 | 492 | -0.154822474 | 0.876597449 |
| endo-siRNA | URS00000D8F76 | RNA11105 | 15 | 7 | -0.511278248 | 0.876597449 |
| endo-siRNA | URS00000F2502 | RNA10159 | 15 | 7 | -0.511278248 | 0.876597449 |
| endo-siRNA | URS000049367B | RNA586 | 15 | 7 | -0.511278248 | 0.876597449 |
| endo-siRNA | URS0000500E42 | RNA7586 | 15 | 7 | -0.511278248 | 0.876597449 |
| endo-siRNA | URS000051F34F | RNA1850 | 15 | 7 | -0.511278248 | 0.876597449 |
| endo-siRNA | URS000023502A | RNA11560 | 11 | 10 | 0.439457094 | 0.876597449 |
| endo-siRNA | URS00004A079E | RNA2919 | 11 | 10 | 0.439457094 | 0.876597449 |
| endo-siRNA | URS00005ABF8E | RNA3484 | 11 | 10 | 0.439457094 | 0.876597449 |
| endo-siRNA | URS000033366F | RNA7423 | 14 | 7 | -0.41276171 | 0.876597449 |
| endo-siRNA | URS000000D306 | RNA524 | 109 | 83 | 0.188725123 | 0.877349028 |
| endo-siRNA | URS00002A2711 | RNA5315 | 154 | 116 | 0.173148911 | 0.87797591 |
| endo-siRNA | URS0000197342 | RNA6555 | 99 | 58 | -0.188956695 | 0.87797591 |
| endo-siRNA | URS000057C33B | RNA6839 | 38 | 21 | -0.272314226 | 0.878555381 |
| endo-siRNA | URS000039E7A0 | RNA6059 | 43 | 34 | 0.242533403 | 0.879087779 |
| endo-siRNA | URS00000E0703 | RNA9393 | 101 | 76 | 0.171589223 | 0.882787414 |
| endo-siRNA | URS00004B2440 | RNA13591 | 459 | 341 | 0.15334333 | 0.88343852 |
| endo-siRNA | URS00002061A9 | RNA1245 | 51 | 29 | -0.231592621 | 0.884289129 |
| endo-siRNA | URS00001CF125 | RNA4160 | 27 | 15 | -0.26427278 | 0.884426973 |
| endo-siRNA | URS00000772EF | RNA8920 | 33 | 26 | 0.237165892 | 0.884688219 |
| endo-siRNA | URS00004302A2 | RNA740 | 359 | 216 | -0.150772929 | 0.884899013 |
| endo-siRNA | URS000036E44E | RNA9405 | 104 | 61 | -0.187300867 | 0.888210101 |
| endo-siRNA | URS000043245B | RNA9796 | 53 | 30 | -0.23818406 | 0.888210101 |
| endo-siRNA | URS000012870C | RNA1840 | 16 | 8 | -0.413395408 | 0.888210101 |
| endo-siRNA | URS0000329F0F | RNA14255 | 16 | 8 | -0.413395408 | 0.888210101 |
| endo-siRNA | URS0000576D1C | RNA14134 | 16 | 8 | -0.413395408 | 0.888210101 |
| endo-siRNA | URS00005FD7F2 | RNA4328 | 16 | 8 | -0.413395408 | 0.888210101 |
| endo-siRNA | URS00000DF5EE | RNA5542 | 16 | 8 | -0.413395408 | 0.888210101 |
| endo-siRNA | URS0000539E1B | RNA4403 | 16 | 8 | -0.413395408 | 0.888210101 |
| endo-siRNA | URS00004F33CD | RNA4387 | 34 | 27 | 0.248531836 | 0.888210101 |
| endo-siRNA | URS00001310EF | RNA879 | 40 | 23 | -0.215382229 | 0.888210101 |
| endo-siRNA | URS0000541E02 | RNA11929 | 66 | 38 | -0.213829839 | 0.888210101 |
| endo-siRNA | URS000060F355 | RNA3458 | 66 | 38 | -0.213829839 | 0.888210101 |
| endo-siRNA | URS000061393A | RNA711 | 47 | 36 | 0.196862676 | 0.891007726 |
| endo-siRNA | URS000011CFFF | RNA13112 | 161 | 96 | -0.163679765 | 0.893817493 |
| endo-siRNA | URS0000357E1A | RNA13918 | 36 | 28 | 0.218698545 | 0.894015374 |
| endo-siRNA | URS0000047DEB | RNA5870 | 205 | 123 | -0.154732923 | 0.897787074 |
| endo-siRNA | URS0000457E44 | RNA3412 | 29 | 16 | -0.274305462 | 0.897787074 |
| endo-siRNA | URS000044E3CA | RNA6669 | 25 | 20 | 0.258765654 | 0.897787074 |
| endo-siRNA | URS00001274D0 | RNA14024 | 98 | 74 | 0.176602571 | 0.89790155 |
| endo-siRNA | URS00005C6866 | RNA13082 | 165 | 99 | -0.154704 | 0.89790155 |
| endo-siRNA | URS00001D9CD0 | RNA5871 | 61 | 47 | 0.205494234 | 0.897908468 |
| endo-siRNA | URS0000286CF0 | RNA6079 | 277 | 167 | -0.147840387 | 0.897908468 |
| endo-siRNA | URS00003CB920 | RNA14160 | 346 | 209 | -0.145089728 | 0.897908468 |
| endo-siRNA | URS00004822ED | RNA14113 | 17 | 9 | -0.332128119 | 0.897908468 |
| endo-siRNA | URS000020E701 | RNA582 | 17 | 9 | -0.332128119 | 0.897908468 |
| endo-siRNA | URS0000227734 | RNA8573 | 17 | 9 | -0.332128119 | 0.897908468 |
| endo-siRNA | URS0000134584 | RNA12121 | 17 | 9 | -0.332128119 | 0.897908468 |
| endo-siRNA | URS00005D8D83 | RNA4080 | 17 | 9 | -0.332128119 | 0.897908468 |
| endo-siRNA | URS00003A5E06 | RNA5104 | 17 | 9 | -0.332128119 | 0.897908468 |
| endo-siRNA | URS00000A7B26 | RNA9561 | 84 | 50 | -0.166033977 | 0.899862966 |
| endo-siRNA | URS0000484DE9 | RNA3478 | 44 | 25 | -0.232602142 | 0.901902999 |
| endo-siRNA | URS000047AD39 | RNA11288 | 44 | 25 | -0.232602142 | 0.901902999 |
| endo-siRNA | URS000023A050 | RNA5324 | 44 | 25 | -0.232602142 | 0.901902999 |
| endo-siRNA | URS00003B443F | RNA1167 | 30 | 17 | -0.23603276 | 0.901902999 |
| endo-siRNA | URS0000140169 | RNA3560 | 285 | 210 | 0.141469789 | 0.903695731 |
| endo-siRNA | URS0000485F25 | RNA6675 | 52 | 39 | 0.166622253 | 0.904694012 |
| endo-siRNA | URS0000179608 | RNA10023 | 15 | 12 | 0.257827918 | 0.907325534 |
| endo-siRNA | URS00000B728D | RNA5024 | 32 | 18 | -0.246701028 | 0.907325534 |
| endo-siRNA | URS00004BAC70 | RNA12136 | 75 | 44 | -0.186875049 | 0.907780695 |
| endo-siRNA | URS00004D1544 | RNA10473 | 119 | 71 | -0.1627404 | 0.908556806 |
| endo-siRNA | URS00004A6A62 | RNA2500 | 40 | 31 | 0.213637596 | 0.909357918 |
| endo-siRNA | URS000015A38B | RNA6461 | 106 | 64 | -0.145590584 | 0.912519555 |
| endo-siRNA | URS00002B2492 | RNA14332 | 33 | 19 | -0.213308875 | 0.912519555 |
| endo-siRNA | URS0000576E5C | RNA3395 | 55 | 42 | 0.192579756 | 0.913248962 |
| endo-siRNA | URS000046CCBD | RNA11974 | 62 | 37 | -0.162214598 | 0.913248962 |
| endo-siRNA | URS00002E3C99 | RNA13589 | 375 | 274 | 0.129349096 | 0.913852728 |
| endo-siRNA | URS0000570296 | RNA2550 | 948 | 691 | 0.125892838 | 0.914450787 |
| endo-siRNA | URS00001DF824 | RNA1219 | 2298 | 1410 | -0.122562046 | 0.914804128 |
| endo-siRNA | URS0000302261 | RNA6486 | 16 | 13 | 0.280169534 | 0.914934797 |
| endo-siRNA | URS00002BF17C | RNA5392 | 56 | 43 | 0.200522106 | 0.916048938 |
| endo-siRNA | URS0000146FCE | RNA1013 | 84 | 63 | 0.166794722 | 0.916459387 |
| endo-siRNA | URS0000560C08 | RNA10480 | 29 | 23 | 0.246523483 | 0.917184904 |
| endo-siRNA | URS00005C6802 | RNA6670 | 30 | 23 | 0.19786117 | 0.917184904 |
| endo-siRNA | URS0000224355 | RNA12143 | 2121 | 1304 | -0.119678687 | 0.91904569 |
| endo-siRNA | URS0000466921 | RNA14105 | 82 | 49 | -0.16041874 | 0.922918336 |
| endo-siRNA | URS00005CD023 | RNA7387 | 31 | 24 | 0.211926814 | 0.922918336 |
| endo-siRNA | URS00003FCD7D | RNA9889 | 21 | 11 | -0.347961251 | 0.922918336 |
| endo-siRNA | URS0000037F13 | RNA7305 | 21 | 11 | -0.347961251 | 0.922918336 |
| endo-siRNA | URS00002BF7E4 | RNA7657 | 21 | 11 | -0.347961251 | 0.922918336 |
| endo-siRNA | URS00001D9081 | RNA10677 | 17 | 14 | 0.29962193 | 0.922918336 |
| endo-siRNA | URS000025CA83 | RNA749 | 177 | 130 | 0.136765023 | 0.923897137 |
| endo-siRNA | URS00005D2AA5 | RNA9888 | 51 | 30 | -0.182848483 | 0.923897137 |
| endo-siRNA | URS000026C0B5 | RNA5067 | 45 | 34 | 0.1771678 | 0.923897137 |
| endo-siRNA | URS00000AD6C9 | RNA4131 | 195 | 142 | 0.124438418 | 0.925599326 |
| endo-siRNA | URS00005D8DDF | RNA10822 | 37 | 21 | -0.233993259 | 0.926556773 |
| endo-siRNA | URS0000119343 | RNA13110 | 32 | 25 | 0.224994004 | 0.926556773 |
| endo-siRNA | URS000029AC0A | RNA4798 | 32 | 25 | 0.224994004 | 0.926556773 |
| endo-siRNA | URS00001B39AB | RNA14026 | 69 | 41 | -0.168470978 | 0.926617483 |
| endo-siRNA | URS0000251363 | RNA14311 | 18 | 15 | 0.316712993 | 0.928326723 |
| endo-siRNA | URS000020979F | RNA5109 | 18 | 15 | 0.316712993 | 0.928326723 |
| endo-siRNA | URS00004D8F73 | RNA5037 | 22 | 12 | -0.290164554 | 0.928326723 |
| endo-siRNA | URS000035CA73 | RNA904 | 22 | 12 | -0.290164554 | 0.928326723 |
| endo-siRNA | URS00003E1D5E | RNA2540 | 19 | 15 | 0.239337657 | 0.928326723 |
| endo-siRNA | URS00004CFEF4 | RNA5070 | 19 | 15 | 0.239337657 | 0.928326723 |
| endo-siRNA | URS00005CBA12 | RNA9925 | 33 | 25 | 0.180803711 | 0.928402154 |
| endo-siRNA | URS0000460C2F | RNA11279 | 33 | 25 | 0.180803711 | 0.928402154 |
| endo-siRNA | URS0000399048 | RNA7506 | 33 | 25 | 0.180803711 | 0.928402154 |
| endo-siRNA | URS000057CDA9 | RNA5077 | 33 | 25 | 0.180803711 | 0.928402154 |
| endo-siRNA | URS00002BDCE4 | RNA7349 | 80 | 59 | 0.142573883 | 0.93029845 |
| endo-siRNA | URS000051CB19 | RNA1345 | 39 | 23 | -0.17899444 | 0.932894173 |
| endo-siRNA | URS0000237667 | RNA14060 | 227 | 140 | -0.11507313 | 0.933840903 |
| endo-siRNA | URS00001D76C3 | RNA4804 | 23 | 13 | -0.239311696 | 0.934015844 |
| endo-siRNA | URS0000349737 | RNA795 | 299 | 184 | -0.118264689 | 0.934673087 |
| endo-siRNA | URS00004ABF3D | RNA12841 | 36 | 27 | 0.166421491 | 0.936380335 |
| endo-siRNA | URS000053C8FB | RNA5431 | 40 | 24 | -0.154242241 | 0.936380335 |
| endo-siRNA | URS00000A7ED5 | RNA3510 | 411 | 297 | 0.113398014 | 0.936380335 |
| endo-siRNA | URS00004C42BC | RNA10121 | 117 | 86 | 0.137844881 | 0.938050849 |
| endo-siRNA | URS00004C9453 | RNA7796 | 53 | 39 | 0.139219753 | 0.9381411 |
| endo-siRNA | URS0000330415 | RNA3511 | 25 | 14 | -0.252731065 | 0.9381411 |
| endo-siRNA | URS00000F7C39 | RNA3569 | 24 | 14 | -0.194195573 | 0.9381411 |
| endo-siRNA | URS00003EFD77 | RNA12020 | 24 | 14 | -0.194195573 | 0.9381411 |
| endo-siRNA | URS0000179096 | RNA760 | 21 | 16 | 0.188534977 | 0.9381411 |
| endo-siRNA | URS00003E0F77 | RNA527 | 37 | 28 | 0.179331854 | 0.938250321 |
| endo-siRNA | URS000016F0CA | RNA5176 | 37 | 28 | 0.179331854 | 0.938250321 |
| endo-siRNA | URS000044C853 | RNA5893 | 37 | 28 | 0.179331854 | 0.938250321 |
| endo-siRNA | URS00000DD2F4 | RNA569 | 96 | 59 | -0.120010809 | 0.941762651 |
| endo-siRNA | URS00001C2F20 | RNA6633 | 80 | 48 | -0.154546692 | 0.942172714 |
| endo-siRNA | URS000042499F | RNA7350 | 26 | 15 | -0.210131288 | 0.942172714 |
| endo-siRNA | URS000027C320 | RNA14249 | 26 | 15 | -0.210131288 | 0.942172714 |
| endo-siRNA | URS0000319063 | RNA5432 | 44 | 26 | -0.17623996 | 0.942172714 |
| endo-siRNA | URS0000228471 | RNA5354 | 119 | 73 | -0.122718619 | 0.942172714 |
| endo-siRNA | URS0000020B37 | RNA9735 | 119 | 73 | -0.122718619 | 0.942172714 |
| endo-siRNA | URS0000018694 | RNA3642 | 10 | 5 | -0.410746955 | 0.942172714 |
| endo-siRNA | URS000023C890 | RNA2704 | 10 | 5 | -0.410746955 | 0.942172714 |
| endo-siRNA | URS0000256A78 | RNA1309 | 10 | 5 | -0.410746955 | 0.942172714 |
| endo-siRNA | URS00001DF1CB | RNA9760 | 10 | 5 | -0.410746955 | 0.942172714 |
| endo-siRNA | URS00004CAA24 | RNA1837 | 10 | 5 | -0.410746955 | 0.942172714 |
| endo-siRNA | URS00005D015E | RNA12358 | 10 | 5 | -0.410746955 | 0.942172714 |
| endo-siRNA | URS00005D129E | RNA5801 | 10 | 5 | -0.410746955 | 0.942172714 |
| endo-siRNA | URS000000AFF3 | RNA11315 | 10 | 5 | -0.410746955 | 0.942172714 |
| endo-siRNA | URS00003AB646 | RNA8214 | 10 | 5 | -0.410746955 | 0.942172714 |
| endo-siRNA | URS00002BBE77 | RNA404 | 10 | 5 | -0.410746955 | 0.942172714 |
| endo-siRNA | URS00001B32C2 | RNA10481 | 10 | 5 | -0.410746955 | 0.942172714 |
| endo-siRNA | URS0000463532 | RNA4517 | 10 | 5 | -0.410746955 | 0.942172714 |
| endo-siRNA | URS00003FA0C6 | RNA5286 | 10 | 5 | -0.410746955 | 0.942172714 |
| endo-siRNA | URS0000597EB0 | RNA4210 | 10 | 5 | -0.410746955 | 0.942172714 |
| endo-siRNA | URS000024FB7C | RNA14145 | 10 | 5 | -0.410746955 | 0.942172714 |
| endo-siRNA | URS00001D3585 | RNA89 | 10 | 5 | -0.410746955 | 0.942172714 |
| endo-siRNA | URS000046A8DB | RNA9615 | 10 | 5 | -0.410746955 | 0.942172714 |
| endo-siRNA | URS00005BBD97 | RNA14236 | 82 | 50 | -0.131331231 | 0.942740116 |
| endo-siRNA | URS000014D312 | RNA4832 | 64 | 38 | -0.169537868 | 0.94342826 |
| endo-siRNA | URS000006A4E7 | RNA13936 | 63 | 38 | -0.146871294 | 0.94342826 |
| endo-siRNA | URS000046CF91 | RNA7454 | 45 | 27 | -0.154309793 | 0.943899746 |
| endo-siRNA | URS0000529887 | RNA5765 | 23 | 18 | 0.227107583 | 0.943899746 |
| endo-siRNA | URS00001182AB | RNA6459 | 65 | 39 | -0.154476327 | 0.945251182 |
| endo-siRNA | URS0000262854 | RNA9736 | 263 | 189 | 0.105378769 | 0.946281813 |
| endo-siRNA | URS000024ECAB | RNA27 | 24 | 19 | 0.243678728 | 0.946281813 |
| endo-siRNA | URS00002AC98A | RNA804 | 28 | 16 | -0.223944293 | 0.946281813 |
| endo-siRNA | URS0000017AA4 | RNA9713 | 28 | 16 | -0.223944293 | 0.946281813 |
| endo-siRNA | URS00004387A2 | RNA4511 | 25 | 19 | 0.185143236 | 0.946281813 |
| endo-siRNA | URS0000504FAC | RNA6044 | 61 | 45 | 0.142894769 | 0.946281813 |
| endo-siRNA | URS00004F3AF8 | RNA1196 | 271 | 169 | -0.099099622 | 0.946281813 |
| endo-siRNA | URS000030006D | RNA5305 | 11 | 6 | -0.28800595 | 0.946281813 |
| endo-siRNA | URS00000410AA | RNA4259 | 11 | 6 | -0.28800595 | 0.946281813 |
| endo-siRNA | URS0000100317 | RNA4353 | 11 | 6 | -0.28800595 | 0.946281813 |
| endo-siRNA | URS00002E8808 | RNA13524 | 11 | 6 | -0.28800595 | 0.946281813 |
| endo-siRNA | URS000051ED18 | RNA5638 | 11 | 6 | -0.28800595 | 0.946281813 |
| endo-siRNA | URS000054220D | RNA8373 | 11 | 6 | -0.28800595 | 0.946281813 |
| endo-siRNA | URS00004430B4 | RNA9608 | 11 | 6 | -0.28800595 | 0.946281813 |
| endo-siRNA | URS000013D905 | RNA4820 | 11 | 6 | -0.28800595 | 0.946281813 |
| endo-siRNA | URS00000686A3 | RNA11027 | 11 | 6 | -0.28800595 | 0.946281813 |
| endo-siRNA | URS000000696C | RNA1177 | 11 | 6 | -0.28800595 | 0.946281813 |
| endo-siRNA | URS000055EE38 | RNA8668 | 11 | 6 | -0.28800595 | 0.946281813 |
| endo-siRNA | URS0000005236 | RNA14325 | 11 | 6 | -0.28800595 | 0.946281813 |
| endo-siRNA | URS0000079748 | RNA442 | 11 | 6 | -0.28800595 | 0.946281813 |
| endo-siRNA | URS0000457597 | RNA3644 | 26 | 20 | 0.202512847 | 0.946281813 |
| endo-siRNA | URS0000280C02 | RNA8375 | 29 | 17 | -0.187370447 | 0.946281813 |
| endo-siRNA | URS000038F127 | RNA14305 | 29 | 17 | -0.187370447 | 0.946281813 |
| endo-siRNA | URS000013D143 | RNA11594 | 29 | 17 | -0.187370447 | 0.946281813 |
| endo-siRNA | URS000034054A | RNA10859 | 29 | 17 | -0.187370447 | 0.946281813 |
| endo-siRNA | URS0000328CE6 | RNA12868 | 70 | 42 | -0.154503125 | 0.946620536 |
| endo-siRNA | URS00004C24FC | RNA6051 | 30 | 18 | -0.15403994 | 0.950819004 |
| endo-siRNA | URS00001F705B | RNA6042 | 47 | 34 | 0.114635856 | 0.951812191 |
| endo-siRNA | URS000034DD1E | RNA2431 | 52 | 32 | -0.117972103 | 0.954915999 |
| endo-siRNA | URS00000C78C5 | RNA5089 | 28 | 21 | 0.166235501 | 0.954948353 |
| endo-siRNA | URS00000FFC1B | RNA9320 | 10 | 8 | 0.256665288 | 0.954948353 |
| endo-siRNA | URS000035F387 | RNA7254 | 10 | 8 | 0.256665288 | 0.954948353 |
| endo-siRNA | URS00002C9642 | RNA848 | 10 | 8 | 0.256665288 | 0.954948353 |
| endo-siRNA | URS00000E3D2E | RNA9699 | 10 | 8 | 0.256665288 | 0.954948353 |
| endo-siRNA | URS0000504445 | RNA13861 | 10 | 8 | 0.256665288 | 0.954948353 |
| endo-siRNA | URS00005CD9C4 | RNA4433 | 10 | 8 | 0.256665288 | 0.954948353 |
| endo-siRNA | URS00002B05A3 | RNA705 | 29 | 22 | 0.182677336 | 0.957660404 |
| endo-siRNA | URS000053B709 | RNA4534 | 30 | 22 | 0.134015022 | 0.957660404 |
| endo-siRNA | URS000009C5EA | RNA5029 | 30 | 22 | 0.134015022 | 0.957660404 |
| endo-siRNA | URS000021B9BB | RNA13132 | 30 | 22 | 0.134015022 | 0.957660404 |
| endo-siRNA | URS000061A973 | RNA4191 | 50 | 37 | 0.147290751 | 0.958012173 |
| endo-siRNA | URS00004E4E15 | RNA6832 | 136 | 97 | 0.094462482 | 0.958754341 |
| endo-siRNA | URS0000017254 | RNA11281 | 420 | 264 | -0.087705882 | 0.958999621 |
| endo-siRNA | URS00000BB113 | RNA491 | 94 | 68 | 0.114811277 | 0.95999881 |
| endo-siRNA | URS00003C3F17 | RNA13922 | 74 | 53 | 0.100383941 | 0.95999881 |
| endo-siRNA | URS000053D47F | RNA1234 | 57 | 35 | -0.121161358 | 0.95999881 |
| endo-siRNA | URS00000C34DA | RNA13254 | 13 | 7 | -0.307021537 | 0.95999881 |
| endo-siRNA | URS00001D9989 | RNA12494 | 13 | 7 | -0.307021537 | 0.95999881 |
| endo-siRNA | URS0000198B08 | RNA1237 | 13 | 7 | -0.307021537 | 0.95999881 |
| endo-siRNA | URS00002C009B | RNA10599 | 11 | 9 | 0.289042112 | 0.95999881 |
| endo-siRNA | URS000011A671 | RNA14106 | 11 | 9 | 0.289042112 | 0.95999881 |
| endo-siRNA | URS0000064AE9 | RNA9640 | 32 | 24 | 0.166340069 | 0.961246752 |
| endo-siRNA | URS00001C4E24 | RNA7341 | 35 | 21 | -0.154155475 | 0.961246752 |
| endo-siRNA | URS00001EA14A | RNA5280 | 130 | 81 | -0.100284823 | 0.961246752 |
| endo-siRNA | URS0000597D6B | RNA4516 | 54 | 39 | 0.112328047 | 0.961246752 |
| endo-siRNA | URS000048E57F | RNA4510 | 409 | 258 | -0.082585383 | 0.964206536 |
| endo-siRNA | URS0000416C63 | RNA3139 | 15 | 8 | -0.321178338 | 0.964206536 |
| endo-siRNA | URS000054BEA4 | RNA924 | 15 | 8 | -0.321178338 | 0.964206536 |
| endo-siRNA | URS00005156E5 | RNA3574 | 15 | 8 | -0.321178338 | 0.964206536 |
| endo-siRNA | URS00005FFB1C | RNA4213 | 15 | 8 | -0.321178338 | 0.964206536 |
| endo-siRNA | URS00004FD843 | RNA3630 | 12 | 10 | 0.315543228 | 0.964206536 |
| endo-siRNA | URS0000377353 | RNA13817 | 12 | 10 | 0.315543228 | 0.964206536 |
| endo-siRNA | URS00001CB491 | RNA712 | 12 | 10 | 0.315543228 | 0.964206536 |
| endo-siRNA | URS00005F3E0C | RNA2278 | 12 | 10 | 0.315543228 | 0.964206536 |
| endo-siRNA | URS000003A02F | RNA5151 | 12 | 10 | 0.315543228 | 0.964206536 |
| endo-siRNA | URS000052A0C0 | RNA940 | 12 | 10 | 0.315543228 | 0.964206536 |
| endo-siRNA | URS0000209822 | RNA7542 | 12 | 10 | 0.315543228 | 0.964206536 |
| endo-siRNA | URS0000262BEF | RNA14292 | 12 | 10 | 0.315543228 | 0.964206536 |
| endo-siRNA | URS0000016482 | RNA10676 | 12 | 10 | 0.315543228 | 0.964206536 |
| endo-siRNA | URS00003864C1 | RNA3628 | 12 | 10 | 0.315543228 | 0.964206536 |
| endo-siRNA | URS000056B556 | RNA9483 | 81 | 58 | 0.100065366 | 0.964206536 |
| endo-siRNA | URS00001144DF | RNA9554 | 38 | 23 | -0.141665075 | 0.964206536 |
| endo-siRNA | URS00004C3EC0 | RNA4161 | 34 | 25 | 0.137926852 | 0.964206536 |
| endo-siRNA | URS000024C02B | RNA3428 | 141 | 88 | -0.097898806 | 0.965461396 |
| endo-siRNA | URS000020DA0D | RNA11814 | 35 | 26 | 0.15264978 | 0.965461396 |
| endo-siRNA | URS000018E13E | RNA14175 | 39 | 24 | -0.117854452 | 0.965461396 |
| endo-siRNA | URS0000514654 | RNA10408 | 36 | 26 | 0.112178687 | 0.965461396 |
| endo-siRNA | URS0000420598 | RNA9474 | 92 | 57 | -0.108375324 | 0.965461396 |
| endo-siRNA | URS0000519EE0 | RNA12459 | 13 | 11 | 0.337638531 | 0.965461396 |
| endo-siRNA | URS000045C308 | RNA5558 | 13 | 11 | 0.337638531 | 0.965461396 |
| endo-siRNA | URS00000E80D6 | RNA7798 | 16 | 9 | -0.24545309 | 0.965461396 |
| endo-siRNA | URS00001C9B91 | RNA11258 | 16 | 9 | -0.24545309 | 0.965461396 |
| endo-siRNA | URS00002651F0 | RNA11984 | 16 | 9 | -0.24545309 | 0.965461396 |
| endo-siRNA | URS00004F9036 | RNA11992 | 16 | 9 | -0.24545309 | 0.965461396 |
| endo-siRNA | URS00003B02DB | RNA2362 | 14 | 11 | 0.231898359 | 0.965461396 |
| endo-siRNA | URS00001F3ECD | RNA9634 | 14 | 11 | 0.231898359 | 0.965461396 |
| endo-siRNA | URS000057D438 | RNA13074 | 14 | 11 | 0.231898359 | 0.965461396 |
| endo-siRNA | URS0000303A70 | RNA1258 | 14 | 11 | 0.231898359 | 0.965461396 |
| endo-siRNA | URS000018673D | RNA13089 | 42 | 25 | -0.165721151 | 0.96831676 |
| endo-siRNA | URS00002EFF55 | RNA9695 | 39 | 29 | 0.154130336 | 0.97034735 |
| endo-siRNA | URS0000248683 | RNA4323 | 43 | 26 | -0.143186992 | 0.97034735 |
| endo-siRNA | URS000005DCEA | RNA3315 | 17 | 10 | -0.181713137 | 0.97034735 |
| endo-siRNA | URS00004960B1 | RNA132 | 17 | 10 | -0.181713137 | 0.97034735 |
| endo-siRNA | URS00001D4434 | RNA5253 | 17 | 10 | -0.181713137 | 0.97034735 |
| endo-siRNA | URS000029B7C9 | RNA2376 | 17 | 10 | -0.181713137 | 0.97034735 |
| endo-siRNA | URS000051BF98 | RNA9824 | 121 | 86 | 0.089407382 | 0.973130255 |
| endo-siRNA | URS0000391329 | RNA5164 | 68 | 49 | 0.109128606 | 0.974604042 |
| endo-siRNA | URS000043C636 | RNA6006 | 70 | 50 | 0.096486597 | 0.975955764 |
| endo-siRNA | URS0000155032 | RNA903 | 71 | 51 | 0.10457853 | 0.976887547 |
| endo-siRNA | URS00002AB002 | RNA5891 | 20 | 11 | -0.278083025 | 0.976887547 |
| endo-siRNA | URS000012B9BF | RNA12037 | 20 | 11 | -0.278083025 | 0.976887547 |
| endo-siRNA | URS000039B2F2 | RNA5654 | 19 | 11 | -0.204647136 | 0.976887547 |
| endo-siRNA | URS00005C8081 | RNA3402 | 19 | 11 | -0.204647136 | 0.976887547 |
| endo-siRNA | URS000034A6F3 | RNA4154 | 19 | 11 | -0.204647136 | 0.976887547 |
| endo-siRNA | URS00003C8DE0 | RNA7372 | 19 | 11 | -0.204647136 | 0.976887547 |
| endo-siRNA | URS000011B10F | RNA3734 | 17 | 13 | 0.193494505 | 0.976887547 |
| endo-siRNA | URS00003DE769 | RNA2972 | 17 | 13 | 0.193494505 | 0.976887547 |
| endo-siRNA | URS0000270B13 | RNA13958 | 17 | 13 | 0.193494505 | 0.976887547 |
| endo-siRNA | URS000052F2D9 | RNA6119 | 17 | 13 | 0.193494505 | 0.976887547 |
| endo-siRNA | URS000053522D | RNA735 | 170 | 108 | -0.072324795 | 0.976887547 |
| endo-siRNA | URS00001892FE | RNA797 | 45 | 33 | 0.134227429 | 0.977484893 |
| endo-siRNA | URS0000464649 | RNA7608 | 50 | 31 | -0.107212966 | 0.977897443 |
| endo-siRNA | URS000041DFD8 | RNA9737 | 107 | 75 | 0.069370082 | 0.977897443 |
| endo-siRNA | URS000048E392 | RNA3643 | 18 | 14 | 0.217860409 | 0.977897443 |
| endo-siRNA | URS0000289FD3 | RNA5290 | 18 | 14 | 0.217860409 | 0.977897443 |
| endo-siRNA | URS0000213FC5 | RNA6560 | 18 | 14 | 0.217860409 | 0.977897443 |
| endo-siRNA | URS00000509C3 | RNA4081 | 18 | 14 | 0.217860409 | 0.977897443 |
| endo-siRNA | URS00004B1929 | RNA6742 | 19 | 14 | 0.140485073 | 0.977897443 |
| endo-siRNA | URS0000548A9E | RNA3631 | 19 | 14 | 0.140485073 | 0.977897443 |
| endo-siRNA | URS000049BD90 | RNA5387 | 22 | 13 | -0.175605868 | 0.980668313 |
| endo-siRNA | URS00002DB4C7 | RNA5078 | 22 | 13 | -0.175605868 | 0.980668313 |
| endo-siRNA | URS0000073D82 | RNA4064 | 20 | 15 | 0.165901767 | 0.980668313 |
| endo-siRNA | URS00005EC242 | RNA11547 | 51 | 37 | 0.118806145 | 0.982216596 |
| endo-siRNA | URS000027C2E9 | RNA5865 | 23 | 14 | -0.133184271 | 0.98374376 |
| endo-siRNA | URS000006D5F2 | RNA816 | 58 | 36 | -0.105659431 | 0.98502288 |
| endo-siRNA | URS00001EF6D2 | RNA14025 | 127 | 81 | -0.066640943 | 0.98502288 |
| endo-siRNA | URS000009215B | RNA5461 | 25 | 15 | -0.153878481 | 0.98591646 |
| endo-siRNA | URS000033180D | RNA11045 | 24 | 15 | -0.095342989 | 0.98591646 |
| endo-siRNA | URS00005683F2 | RNA5773 | 126 | 88 | 0.064190261 | 0.986936132 |
| endo-siRNA | URS00003EE5CD | RNA9795 | 57 | 41 | 0.106505294 | 0.9885842 |
| endo-siRNA | URS00002AC1E1 | RNA9478 | 23 | 17 | 0.145114764 | 0.988825158 |
| endo-siRNA | URS00002D9FF3 | RNA5195 | 23 | 17 | 0.145114764 | 0.988825158 |
| endo-siRNA | URS00003FC57A | RNA9397 | 212 | 136 | -0.058302303 | 0.989400489 |
| endo-siRNA | URS00004F6E61 | RNA5334 | 25 | 18 | 0.10756079 | 0.989949364 |
| endo-siRNA | URS00004A2E20 | RNA5274 | 65 | 41 | -0.082506753 | 0.99053254 |
| endo-siRNA | URS000060D869 | RNA4065 | 28 | 17 | -0.137009277 | 0.992043613 |
| endo-siRNA | URS000019E0E7 | RNA8454 | 28 | 17 | -0.137009277 | 0.992043613 |
| endo-siRNA | URS00004D50E2 | RNA7018 | 219 | 152 | 0.055216625 | 0.992395172 |
| endo-siRNA | URS00000E4E34 | RNA12873 | 227 | 146 | -0.054573958 | 0.992395172 |
| endo-siRNA | URS000017470E | RNA3617 | 27 | 20 | 0.148371355 | 0.994185443 |
| endo-siRNA | URS00004F051D | RNA13248 | 31 | 19 | -0.123531837 | 0.995238241 |
| endo-siRNA | URS000041AD9B | RNA2700 | 30 | 19 | -0.076457494 | 0.995238241 |
| endo-siRNA | URS00001A715B | RNA3361 | 33 | 20 | -0.139686457 | 0.99881559 |
| endo-siRNA | URS000000F0B4 | RNA500 | 31 | 22 | 0.086940679 | 0.99881559 |
| endo-siRNA | URS000060ABCB | RNA855 | 124 | 79 | -0.068217876 | 0.999145623 |
| endo-siRNA | URS000061FF9E | RNA13528 | 76 | 53 | 0.061986536 | 0.999145623 |
| endo-siRNA | URS0000246D13 | RNA12134 | 34 | 21 | -0.112516222 | 0.999145623 |
| endo-siRNA | URS0000311900 | RNA2524 | 34 | 21 | -0.112516222 | 0.999145623 |
| endo-siRNA | URS000036B4EB | RNA3394 | 34 | 21 | -0.112516222 | 0.999145623 |
| endo-siRNA | URS00001BF48F | RNA9566 | 32 | 23 | 0.105200081 | 0.999145623 |
| endo-siRNA | URS0000284AD7 | RNA5187 | 32 | 23 | 0.105200081 | 0.999145623 |
| endo-siRNA | URS0000289F35 | RNA5082 | 32 | 23 | 0.105200081 | 0.999145623 |
| endo-siRNA | URS00003CDB1D | RNA938 | 32 | 23 | 0.105200081 | 0.999145623 |
| endo-siRNA | URS00003791E2 | RNA5171 | 36 | 22 | -0.127823564 | 1 |
| endo-siRNA | URS000034A6AA | RNA13105 | 35 | 22 | -0.087352471 | 1 |
| endo-siRNA | URS0000143E77 | RNA11548 | 80 | 56 | 0.067416678 | 1 |
| endo-siRNA | URS00001ED8C7 | RNA13220 | 37 | 23 | -0.103344108 | 1 |
| endo-siRNA | URS00004E756C | RNA4273 | 37 | 23 | -0.103344108 | 1 |
| endo-siRNA | URS00005BA253 | RNA7503 | 35 | 25 | 0.096287598 | 1 |
| endo-siRNA | URS000013FB6D | RNA5273 | 35 | 25 | 0.096287598 | 1 |
| endo-siRNA | URS00000C0CDF | RNA4363 | 38 | 24 | -0.080525087 | 1 |
| endo-siRNA | URS00005BCF32 | RNA8277 | 37 | 26 | 0.072811997 | 1 |
| endo-siRNA | URS0000299500 | RNA3695 | 37 | 26 | 0.072811997 | 1 |
| endo-siRNA | URS00004499BD | RNA5642 | 40 | 25 | -0.095588306 | 1 |
| endo-siRNA | URS00002E869E | RNA2968 | 43 | 27 | -0.088944189 | 1 |
| endo-siRNA | URS00003C53F6 | RNA9388 | 46 | 33 | 0.102622687 | 1 |
| endo-siRNA | URS00005AC0A9 | RNA7505 | 50 | 32 | -0.061554421 | 1 |
| endo-siRNA | URS00000A0CFE | RNA5097 | 53 | 37 | 0.063470568 | 1 |
| endo-siRNA | URS00002BE016 | RNA9558 | 123 | 84 | 0.031877746 | 1 |
| endo-siRNA | URS000026B222 | RNA12141 | 58 | 37 | -0.066239209 | 1 |
| endo-siRNA | URS0000520711 | RNA3413 | 57 | 37 | -0.041213464 | 1 |
| endo-siRNA | URS00004E6D58 | RNA13590 | 497 | 326 | -0.026252651 | 1 |
| endo-siRNA | URS000035CCB4 | RNA2420 | 191 | 130 | 0.027031217 | 1 |
| endo-siRNA | URS0000158E63 | RNA13696 | 197 | 134 | 0.026130916 | 1 |
| endo-siRNA | URS00001BB13C | RNA750 | 95 | 65 | 0.034571557 | 1 |
| endo-siRNA | URS000022F76F | RNA6073 | 490 | 331 | 0.0161578 | 1 |
| endo-siRNA | URS000045D533 | RNA613 | 116 | 79 | 0.027877326 | 1 |
| endo-siRNA | URS00001128E2 | RNA9775 | 125 | 85 | 0.025689368 | 1 |
| endo-siRNA | URS00005D92B5 | RNA13588 | 339 | 225 | -0.009242926 | 1 |
| endo-siRNA | URS00003BD7ED | RNA10120 | 1187 | 796 | 0.005632938 | 1 |
| endo-siRNA | URS00003A52C0 | RNA1218 | 4178 | 2785 | -0.00302181 | 1 |
| endo-siRNA | URS00003613AA | RNA86 | 14 | 8 | -0.2226618 | 1 |
| endo-siRNA | URS000032DCB2 | RNA2475 | 14 | 8 | -0.2226618 | 1 |
| endo-siRNA | URS000043CEFD | RNA3468 | 14 | 8 | -0.2226618 | 1 |
| endo-siRNA | URS0000579F1A | RNA3416 | 14 | 8 | -0.2226618 | 1 |
| endo-siRNA | URS00002E5202 | RNA5982 | 13 | 10 | 0.201435673 | 1 |
| endo-siRNA | URS00005C1768 | RNA6494 | 13 | 10 | 0.201435673 | 1 |
| endo-siRNA | URS000023A257 | RNA13618 | 13 | 10 | 0.201435673 | 1 |
| endo-siRNA | URS000053DBA3 | RNA13868 | 12 | 7 | -0.192913982 | 1 |
| endo-siRNA | URS000025A5BF | RNA10450 | 12 | 7 | -0.192913982 | 1 |
| endo-siRNA | URS0000382974 | RNA4811 | 12 | 7 | -0.192913982 | 1 |
| endo-siRNA | URS00004D7977 | RNA11320 | 12 | 7 | -0.192913982 | 1 |
| endo-siRNA | URS0000272DE7 | RNA2180 | 12 | 7 | -0.192913982 | 1 |
| endo-siRNA | URS000008E735 | RNA14376 | 12 | 7 | -0.192913982 | 1 |
| endo-siRNA | URS000021FF4D | RNA6115 | 12 | 7 | -0.192913982 | 1 |
| endo-siRNA | URS00005C2793 | RNA5500 | 12 | 9 | 0.165128246 | 1 |
| endo-siRNA | URS00003ED1DB | RNA1902 | 12 | 9 | 0.165128246 | 1 |
| endo-siRNA | URS000029B06E | RNA13055 | 12 | 9 | 0.165128246 | 1 |
| endo-siRNA | URS00002C66A1 | RNA9560 | 20 | 12 | -0.153636928 | 1 |
| endo-siRNA | URS00001C12A1 | RNA10005 | 15 | 9 | -0.15323602 | 1 |
| endo-siRNA | URS00000BF389 | RNA2495 | 15 | 9 | -0.15323602 | 1 |
| endo-siRNA | URS0000237AA5 | RNA5380 | 15 | 9 | -0.15323602 | 1 |
| endo-siRNA | URS0000554EE4 | RNA803 | 15 | 9 | -0.15323602 | 1 |
| endo-siRNA | URS00005FA718 | RNA13108 | 10 | 6 | -0.152440456 | 1 |
| endo-siRNA | URS00000DFC55 | RNA9838 | 10 | 6 | -0.152440456 | 1 |
| endo-siRNA | URS000010CC8C | RNA578 | 10 | 6 | -0.152440456 | 1 |
| endo-siRNA | URS00000973A9 | RNA8211 | 10 | 6 | -0.152440456 | 1 |
| endo-siRNA | URS0000303BD6 | RNA11527 | 10 | 6 | -0.152440456 | 1 |
| endo-siRNA | URS00004236EC | RNA5493 | 10 | 6 | -0.152440456 | 1 |
| endo-siRNA | URS00000CB3FB | RNA10521 | 10 | 6 | -0.152440456 | 1 |
| endo-siRNA | URS000013E5C2 | RNA9567 | 10 | 6 | -0.152440456 | 1 |
| endo-siRNA | URS0000265D0A | RNA4818 | 15 | 11 | 0.13338182 | 1 |
| endo-siRNA | URS0000604896 | RNA13765 | 18 | 11 | -0.1272718 | 1 |
| endo-siRNA | URS000045832E | RNA3390 | 18 | 11 | -0.1272718 | 1 |
| endo-siRNA | URS0000343627 | RNA1168 | 11 | 8 | 0.121099795 | 1 |
| endo-siRNA | URS00000674F7 | RNA10269 | 11 | 8 | 0.121099795 | 1 |
| endo-siRNA | URS00005FBAFF | RNA3354 | 11 | 8 | 0.121099795 | 1 |
| endo-siRNA | URS00005BE115 | RNA6574 | 11 | 8 | 0.121099795 | 1 |
| endo-siRNA | URS0000369952 | RNA7543 | 11 | 8 | 0.121099795 | 1 |
| endo-siRNA | URS000027AE3A | RNA1012 | 26 | 16 | -0.117619852 | 1 |
| endo-siRNA | URS00002B850F | RNA4356 | 13 | 8 | -0.116921627 | 1 |
| endo-siRNA | URS00000464D0 | RNA12327 | 13 | 8 | -0.116921627 | 1 |
| endo-siRNA | URS000003C4D5 | RNA10407 | 21 | 13 | -0.108956468 | 1 |
| endo-siRNA | URS000024E92C | RNA85 | 21 | 13 | -0.108956468 | 1 |
| endo-siRNA | URS000047C9AD | RNA1188 | 21 | 15 | 0.096023541 | 1 |
| endo-siRNA | URS0000394E52 | RNA13114 | 21 | 15 | 0.096023541 | 1 |
| endo-siRNA | URS0000499666 | RNA5116 | 14 | 10 | 0.0956955 | 1 |
| endo-siRNA | URS00000D875A | RNA6491 | 32 | 20 | -0.095496164 | 1 |
| endo-siRNA | URS00004AA0A6 | RNA2416 | 16 | 10 | -0.095038108 | 1 |
| endo-siRNA | URS000056BD0A | RNA391 | 27 | 17 | -0.084826329 | 1 |
| endo-siRNA | URS000018311B | RNA8446 | 24 | 17 | 0.084103462 | 1 |
| endo-siRNA | URS00003A782E | RNA6488 | 24 | 17 | 0.084103462 | 1 |
| endo-siRNA | URS00003E85D4 | RNA8883 | 19 | 12 | -0.080201038 | 1 |
| endo-siRNA | URS00003BDC49 | RNA6005 | 19 | 12 | -0.080201038 | 1 |
| endo-siRNA | URS0000361F18 | RNA9643 | 17 | 12 | 0.078935819 | 1 |
| endo-siRNA | URS00002916A2 | RNA5066 | 17 | 12 | 0.078935819 | 1 |
| endo-siRNA | URS00002F2D31 | RNA7801 | 17 | 12 | 0.078935819 | 1 |
| endo-siRNA | URS0000427DFE | RNA6662 | 27 | 19 | 0.074748937 | 1 |
| endo-siRNA | URS0000081646 | RNA8370 | 33 | 21 | -0.069639363 | 1 |
| endo-siRNA | URS00002A0CE6 | RNA5299 | 33 | 21 | -0.069639363 | 1 |
| endo-siRNA | URS0000536B47 | RNA11091 | 22 | 14 | -0.069478443 | 1 |
| endo-siRNA | URS0000160ED6 | RNA7330 | 22 | 14 | -0.069478443 | 1 |
| endo-siRNA | URS0000500353 | RNA3539 | 11 | 7 | -0.069000116 | 1 |
| endo-siRNA | URS00005F15BC | RNA12131 | 11 | 7 | -0.069000116 | 1 |
| endo-siRNA | URS00002DBC3C | RNA5651 | 11 | 7 | -0.069000116 | 1 |
| endo-siRNA | URS00002982BD | RNA14346 | 11 | 7 | -0.069000116 | 1 |
| endo-siRNA | URS00004765A9 | RNA7276 | 40 | 28 | 0.067293733 | 1 |
| endo-siRNA | URS0000061DC1 | RNA6040 | 40 | 28 | 0.067293733 | 1 |
| endo-siRNA | URS000046A956 | RNA4206 | 20 | 14 | 0.067049183 | 1 |
| endo-siRNA | URS00000853CA | RNA5522 | 20 | 14 | 0.067049183 | 1 |
| endo-siRNA | URS0000433F10 | RNA799 | 10 | 7 | 0.066565378 | 1 |
| endo-siRNA | URS0000014C84 | RNA10664 | 10 | 7 | 0.066565378 | 1 |
| endo-siRNA | URS000007D83D | RNA4307 | 10 | 7 | 0.066565378 | 1 |
| endo-siRNA | URS0000440A64 | RNA9307 | 47 | 30 | -0.06537173 | 1 |
| endo-siRNA | URS000019EAA7 | RNA1238 | 33 | 23 | 0.061009788 | 1 |
| endo-siRNA | URS0000546255 | RNA2988 | 39 | 25 | -0.059200517 | 1 |
| endo-siRNA | URS00001A35B4 | RNA1302 | 23 | 16 | 0.058179748 | 1 |
| endo-siRNA | URS000003D2B8 | RNA11294 | 23 | 16 | 0.058179748 | 1 |
| endo-siRNA | URS00000EF37B | RNA12115 | 23 | 16 | 0.058179748 | 1 |
| endo-siRNA | URS00003AF7A1 | RNA10318 | 53 | 34 | -0.058176474 | 1 |
| endo-siRNA | URS00001727CF | RNA5779 | 42 | 27 | -0.055116167 | 1 |
| endo-siRNA | URS00000AD0C7 | RNA7388 | 42 | 27 | -0.055116167 | 1 |
| endo-siRNA | URS0000596764 | RNA11949 | 42 | 27 | -0.055116167 | 1 |
| endo-siRNA | URS000015EF21 | RNA10439 | 14 | 9 | -0.054719482 | 1 |
| endo-siRNA | URS00000D0F73 | RNA13083 | 14 | 9 | -0.054719482 | 1 |
| endo-siRNA | URS00000C2C47 | RNA5918 | 14 | 9 | -0.054719482 | 1 |
| endo-siRNA | URS000030C4F2 | RNA8851 | 14 | 9 | -0.054719482 | 1 |
| endo-siRNA | URS00001FFBF7 | RNA12461 | 14 | 9 | -0.054719482 | 1 |
| endo-siRNA | URS00002FDC31 | RNA1075 | 45 | 29 | -0.051583925 | 1 |
| endo-siRNA | URS00004CA00C | RNA5652 | 45 | 29 | -0.051583925 | 1 |
| endo-siRNA | URS00000E1B88 | RNA5885 | 26 | 18 | 0.051307983 | 1 |
| endo-siRNA | URS00004772F6 | RNA7620 | 13 | 9 | 0.05102069 | 1 |
| endo-siRNA | URS00003A150F | RNA4088 | 13 | 9 | 0.05102069 | 1 |
| endo-siRNA | URS0000468B1A | RNA14039 | 13 | 9 | 0.05102069 | 1 |
| endo-siRNA | URS00000138DD | RNA4326 | 13 | 9 | 0.05102069 | 1 |
| endo-siRNA | URS000058BBA6 | RNA2854 | 13 | 9 | 0.05102069 | 1 |
| endo-siRNA | URS0000228D50 | RNA11825 | 31 | 20 | -0.049909419 | 1 |
| endo-siRNA | URS0000023754 | RNA3566 | 31 | 20 | -0.049909419 | 1 |
| endo-siRNA | URS000037A8CB | RNA6497 | 31 | 20 | -0.049909419 | 1 |
| endo-siRNA | URS000031BAFE | RNA4094 | 31 | 20 | -0.049909419 | 1 |
| endo-siRNA | URS000018301F | RNA11820 | 42 | 29 | 0.047609701 | 1 |
| endo-siRNA | URS0000355EDB | RNA11299 | 29 | 20 | 0.045827237 | 1 |
| endo-siRNA | URS00001B0032 | RNA10455 | 34 | 22 | -0.045713218 | 1 |
| endo-siRNA | URS000024547F | RNA9774 | 17 | 11 | -0.045510279 | 1 |
| endo-siRNA | URS00005EC8E0 | RNA12879 | 17 | 11 | -0.045510279 | 1 |
| endo-siRNA | URS00001200B2 | RNA2241 | 17 | 11 | -0.045510279 | 1 |
| endo-siRNA | URS0000042B54 | RNA5913 | 17 | 11 | -0.045510279 | 1 |
| endo-siRNA | URS0000383C94 | RNA2509 | 17 | 11 | -0.045510279 | 1 |
| endo-siRNA | URS00003D6A92 | RNA11303 | 54 | 35 | -0.043369032 | 1 |
| endo-siRNA | URS000001A405 | RNA11289 | 48 | 33 | 0.041417383 | 1 |
| endo-siRNA | URS00000CF6A1 | RNA3363 | 32 | 22 | 0.041353934 | 1 |
| endo-siRNA | URS000010C495 | RNA13948 | 16 | 11 | 0.04116475 | 1 |
| endo-siRNA | URS00000D0537 | RNA7549 | 16 | 11 | 0.04116475 | 1 |
| endo-siRNA | URS0000409865 | RNA4262 | 16 | 11 | 0.04116475 | 1 |
| endo-siRNA | URS000025FAE5 | RNA10387 | 16 | 11 | 0.04116475 | 1 |
| endo-siRNA | URS000049F96D | RNA9395 | 67 | 46 | 0.039498853 | 1 |
| endo-siRNA | URS000061D445 | RNA11960 | 60 | 39 | -0.039275667 | 1 |
| endo-siRNA | URS00005608F4 | RNA10149 | 20 | 13 | -0.039078242 | 1 |
| endo-siRNA | URS0000163CF8 | RNA3632 | 20 | 13 | -0.039078242 | 1 |
| endo-siRNA | URS000038CD3B | RNA9595 | 35 | 24 | 0.037633663 | 1 |
| endo-siRNA | URS00002B1688 | RNA13107 | 35 | 24 | 0.037633663 | 1 |
| endo-siRNA | URS000052D5D8 | RNA10137 | 38 | 26 | 0.03449103 | 1 |
| endo-siRNA | URS0000364688 | RNA697 | 19 | 13 | 0.034357648 | 1 |
| endo-siRNA | URS0000041726 | RNA898 | 23 | 15 | -0.034331687 | 1 |
| endo-siRNA | URS0000393E91 | RNA11111 | 23 | 15 | -0.034331687 | 1 |
| endo-siRNA | URS0000218BB9 | RNA5550 | 23 | 15 | -0.034331687 | 1 |
| endo-siRNA | URS000043056A | RNA496 | 75 | 49 | -0.031931075 | 1 |
| endo-siRNA | URS00004A242B | RNA5228 | 26 | 17 | -0.030684837 | 1 |
| endo-siRNA | URS0000543CAC | RNA3364 | 22 | 15 | 0.029374141 | 1 |
| endo-siRNA | URS0000285E71 | RNA1159 | 55 | 36 | -0.029240965 | 1 |
| endo-siRNA | URS00004EB79B | RNA7344 | 113 | 74 | -0.028574072 | 1 |
| endo-siRNA | URS0000398D66 | RNA36 | 29 | 19 | -0.02779518 | 1 |
| endo-siRNA | URS000036DF82 | RNA10003 | 29 | 19 | -0.02779518 | 1 |
| endo-siRNA | URS0000420283 | RNA9702 | 50 | 34 | 0.025643709 | 1 |
| endo-siRNA | URS00005AA06C | RNA5030 | 25 | 17 | 0.02556797 | 1 |
| endo-siRNA | URS00002DD771 | RNA5976 | 32 | 21 | -0.02544907 | 1 |
| endo-siRNA | URS00005BDA9D | RNA8892 | 32 | 21 | -0.02544907 | 1 |
| endo-siRNA | URS000040E00D | RNA7340 | 106 | 72 | 0.024084022 | 1 |
| endo-siRNA | URS000035B3A7 | RNA11667 | 35 | 23 | -0.023506324 | 1 |
| endo-siRNA | URS0000424927 | RNA2539 | 28 | 19 | 0.022565989 | 1 |
| endo-siRNA | URS0000584959 | RNA1275 | 31 | 21 | 0.020137675 | 1 |
| endo-siRNA | URS000030BD86 | RNA981 | 85 | 56 | -0.019887456 | 1 |
| endo-siRNA | URS0000030208 | RNA9575 | 47 | 31 | -0.018220819 | 1 |
| endo-siRNA | URS00005E0472 | RNA9778 | 68 | 46 | 0.018172558 | 1 |
| endo-siRNA | URS000003121F | RNA4389 | 68 | 46 | 0.018172558 | 1 |
| endo-siRNA | URS00005BA1B3 | RNA927 | 56 | 37 | -0.015745935 | 1 |
| endo-siRNA | URS00005834E7 | RNA7522 | 117 | 79 | 0.015509513 | 1 |
| endo-siRNA | URS000035444E | RNA9644 | 124 | 82 | -0.014513428 | 1 |
| endo-siRNA | URS00003C4806 | RNA531 | 62 | 41 | -0.014495839 | 1 |
| endo-siRNA | URS00003DC53F | RNA831 | 46 | 31 | 0.012706382 | 1 |
| endo-siRNA | URS00000F137B | RNA3453 | 46 | 31 | 0.012706382 | 1 |
| endo-siRNA | URS000037DDE5 | RNA5118 | 46 | 31 | 0.012706382 | 1 |
| endo-siRNA | URS0000231CD7 | RNA4745 | 98 | 66 | 0.01177958 | 1 |
| endo-siRNA | URS00002ECC89 | RNA9882 | 125 | 84 | 0.008636061 | 1 |
| endo-siRNA | URS000030E0EF | RNA10626 | 595 | 396 | -0.00527466 | 1 |
| endo-siRNA | URS0000207450 | RNA4509 | 342 | 229 | 0.003463004 | 1 |
| endo-siRNA | URS00000EF300 | RNA708 | 66 | 44 | -0.002842792 | 1 |
| endo-siRNA | URS000056CBC3 | RNA11298 | 36 | 24 | -0.002837429 | 1 |
| endo-siRNA | URS0000265BDE | RNA6495 | 36 | 24 | -0.002837429 | 1 |
| endo-siRNA | URS00005BFCD8 | RNA792 | 33 | 22 | -0.002836359 | 1 |
| endo-siRNA | URS000017B41B | RNA14308 | 33 | 22 | -0.002836359 | 1 |
| endo-siRNA | URS0000230EAC | RNA9502 | 24 | 16 | -0.002831553 | 1 |
| endo-siRNA | URS00004D7DF7 | RNA8844 | 21 | 14 | -0.002829043 | 1 |
| endo-siRNA | URS0000515625 | RNA9204 | 21 | 14 | -0.002829043 | 1 |
| endo-siRNA | URS00004E79B5 | RNA5098 | 18 | 12 | -0.002825702 | 1 |
| endo-siRNA | URS0000224EF0 | RNA4151 | 18 | 12 | -0.002825702 | 1 |
| endo-siRNA | URS000053FDB0 | RNA3731 | 18 | 12 | -0.002825702 | 1 |
| endo-siRNA | URS00003980FE | RNA5543 | 18 | 12 | -0.002825702 | 1 |
| endo-siRNA | URS0000006DC0 | RNA4139 | 18 | 12 | -0.002825702 | 1 |
| endo-siRNA | URS00001BF028 | RNA4149 | 15 | 10 | -0.002821038 | 1 |
| endo-siRNA | URS00003791D1 | RNA13104 | 12 | 8 | -0.002814071 | 1 |
| endo-siRNA | URS000051D9B9 | RNA11546 | 12 | 8 | -0.002814071 | 1 |
| endo-siRNA | URS000049FEAC | RNA5251 | 12 | 8 | -0.002814071 | 1 |
| endo-siRNA | URS000024260F | RNA5344 | 12 | 8 | -0.002814071 | 1 |
| endo-siRNA | URS000010F7B9 | RNA9707 | 12 | 8 | -0.002814071 | 1 |
| endo-siRNA | URS000004A3FB | RNA7434 | 12 | 8 | -0.002814071 | 1 |
| endo-siRNA | URS0000412CBC | RNA4815 | 12 | 8 | -0.002814071 | 1 |
