# Supplementary material for: The small non-coding RNA profile of mouse oocytes is modified during aging
Source: Aging (Albany NY). 2019 May 24;11(10):2968–97. doi: 10.18632/aging.101947 (PMC6555462; doi:10.18632/aging.101947)
Supplement: Supplementary Table 5 [file aging-11-101947-s006.docx]

| Supplementary Table S5. mRNA target prediction of differentially expressed miRNA between young and aged oocytes using miRDB. Table includes target mRNA gene symbol, gene description, target rank, and target score. | | | | |
| --- | --- | --- | --- | --- |
| miRNA Name | Gene Symbol | Gene Description | Target Rank | Target Score |
| mmu-miR-486a-3p | *Scn2b* | sodium channel, voltage-gated, type II, beta subunit | 1 | 100 |
| mmu-miR-486a-3p | *Naa60* | N(alpha)-acetyltransferase 60, NatF catalytic subunit | 2 | 100 |
| mmu-miR-486a-3p | *Tmem132e* | transmembrane protein 132E | 3 | 100 |
| mmu-miR-486a-3p | *Mark2* | MAP/microtubule affinity-regulating kinase 2 | 4 | 100 |
| mmu-miR-486a-3p | *Stim1* | stromal interaction molecule 1 | 5 | 100 |
| mmu-miR-486a-3p | *Ppard* | peroxisome proliferator-activated receptor delta | 6 | 100 |
| mmu-miR-486a-3p | *Kirrel* | kin of IRRE like (Drosophila) | 7 | 100 |
| mmu-miR-486a-3p | *Plcb1* | phospholipase C, beta 1 (phosphoinositide-specific) | 8 | 100 |
| mmu-miR-486a-3p | *Cacna2d2* | calcium channel, voltage-dependent, alpha 2/delta subunit 2 | 9 | 100 |
| mmu-miR-486a-3p | *Nacc2* | NACC family member 2, BEN and BTB (POZ) domain containing | 10 | 100 |
| mmu-miR-486a-3p | *Plekho2* | pleckstrin homology domain containing, family O member 2 | 11 | 100 |
| mmu-miR-486a-3p | *Flot2* | flotillin 2 | 12 | 99 |
| mmu-miR-486a-3p | *Atxn7l3* | ataxin 7-like 3 | 13 | 99 |
| mmu-miR-486a-3p | *Rnf41* | ring finger protein 41, E3 ubiquitin protein ligase | 14 | 99 |
| mmu-miR-486a-3p | *Pou2f2* | POU class 2 homeobox 2 | 15 | 99 |
| mmu-miR-486a-3p | *Fbxo41* | F-box protein 41 | 16 | 99 |
| mmu-miR-486a-3p | *Pacs2* | phosphofurin acidic cluster sorting protein 2 | 17 | 99 |
| mmu-miR-486a-3p | *Dab2ip* | DAB2 interacting protein | 18 | 99 |
| mmu-miR-486a-3p | *Hrh3* | histamine receptor H3 | 19 | 99 |
| mmu-miR-486a-3p | *Luzp1* | leucine zipper protein 1 | 20 | 98 |
| mmu-miR-486a-3p | *Dnmbp* | dynamin binding protein | 21 | 98 |
| mmu-miR-486a-3p | *Nsd1* | nuclear receptor binding SET domain protein 1 | 22 | 98 |
| mmu-miR-486a-3p | *Tbc1d22b* | TBC1 domain family, member 22B | 23 | 98 |
| mmu-miR-486a-3p | *Dmbx1* | diencephalon/mesencephalon homeobox 1 | 24 | 98 |
| mmu-miR-486a-3p | *Trabd2b* | TraB domain containing 2B | 25 | 98 |
| mmu-miR-486a-3p | *Fam102a* | family with sequence similarity 102, member A | 26 | 98 |
| mmu-miR-486a-3p | *Tmem63c* | transmembrane protein 63C | 27 | 98 |
| mmu-miR-486a-3p | *Csrnp1* | cysteine-serine-rich nuclear protein 1 | 28 | 98 |
| mmu-miR-486a-3p | *Capn6* | calpain 6 | 29 | 98 |
| mmu-miR-486a-3p | *Map1lc3a* | microtubule-associated protein 1 light chain 3 alpha | 30 | 98 |
| mmu-miR-486a-3p | *Tmem104* | transmembrane protein 104 | 31 | 97 |
| mmu-miR-486a-3p | *Pak6* | p21 protein (Cdc42/Rac)-activated kinase 6 | 32 | 97 |
| mmu-miR-486a-3p | *Cnih2* | cornichon family AMPA receptor auxiliary protein 2 | 33 | 97 |
| mmu-miR-486a-3p | *Crtc1* | CREB regulated transcription coactivator 1 | 34 | 97 |
| mmu-miR-486a-3p | *Cd276* | CD276 molecule | 35 | 97 |
| mmu-miR-486a-3p | *Vamp2* | vesicle-associated membrane protein 2 (synaptobrevin 2) | 36 | 97 |
| mmu-miR-486a-3p | *Arid1b* | AT rich interactive domain 1B (SWI1-like) | 37 | 97 |
| mmu-miR-486a-3p | *Rab11fip4* | RAB11 family interacting protein 4 (class II) | 38 | 97 |
| mmu-miR-486a-3p | *Adarb1* | adenosine deaminase, RNA-specific, B1 | 39 | 97 |
| mmu-miR-486a-3p | *Chst1* | carbohydrate (keratan sulfate Gal-6) sulfotransferase 1 | 40 | 96 |
| mmu-miR-486a-3p | *Tbc1d13* | TBC1 domain family, member 13 | 41 | 96 |
| mmu-miR-486a-3p | *Arhgap1* | Rho GTPase activating protein 1 | 42 | 96 |
| mmu-miR-486a-3p | *Sbk1* | SH3 domain binding kinase 1 | 43 | 96 |
| mmu-miR-486a-3p | *Atf7* | activating transcription factor 7 | 44 | 96 |
| mmu-miR-486a-3p | *Cntnap1* | contactin associated protein 1 | 45 | 96 |
| mmu-miR-486a-3p | *Aatk* | apoptosis-associated tyrosine kinase | 46 | 96 |
| mmu-miR-486a-3p | *Dot1l* | DOT1-like histone H3K79 methyltransferase | 47 | 96 |
| mmu-miR-486a-3p | *Wnt4* | wingless-type MMTV integration site family, member 4 | 48 | 96 |
| mmu-miR-486a-3p | *Clip3* | CAP-GLY domain containing linker protein 3 | 49 | 96 |
| mmu-miR-486a-3p | *Nfic* | nuclear factor I/C (CCAAT-binding transcription factor) | 50 | 95 |
| mmu-miR-486a-3p | *Pvrl1* | poliovirus receptor-related 1 (herpesvirus entry mediator C) | 51 | 95 |
| mmu-miR-486a-3p | *Elfn2* | extracellular leucine-rich repeat and fibronectin type III domain containing 2 | 52 | 95 |
| mmu-miR-486a-3p | *Tcf12* | transcription factor 12 | 53 | 95 |
| mmu-miR-486a-3p | *Mcm5* | minichromosome maintenance complex component 5 | 54 | 95 |
| mmu-miR-486a-3p | *Nptx1* | neuronal pentraxin I | 55 | 95 |
| mmu-miR-486a-3p | *Caln1* | calneuron 1 | 56 | 95 |
| mmu-miR-486a-3p | *Sh3kbp1* | SH3-domain kinase binding protein 1 | 57 | 95 |
| mmu-miR-486a-3p | *Vps9d1* | VPS9 domain containing 1 | 58 | 95 |
| mmu-miR-486a-3p | *Kif21b* | kinesin family member 21B | 59 | 95 |
| mmu-miR-486a-3p | *Ap1b1* | adaptor-related protein complex 1, beta 1 subunit | 60 | 95 |
| mmu-miR-486a-3p | *Cemip* | cell migration inducing protein, hyaluronan binding | 61 | 95 |
| mmu-miR-486a-3p | *Cecr6* | cat eye syndrome chromosome region, candidate 6 | 62 | 95 |
| mmu-miR-486a-3p | *Agpat1* | 1-acylglycerol-3-phosphate O-acyltransferase 1 | 63 | 95 |
| mmu-miR-486a-3p | *C8orf46* | chromosome 8 open reading frame 46 | 64 | 95 |
| mmu-miR-486a-3p | *Csnk1g1* | casein kinase 1, gamma 1 | 65 | 95 |
| mmu-miR-486a-3p | *Vash1* | vasohibin 1 | 66 | 95 |
| mmu-miR-486a-3p | *Dcaf7* | DDB1 and CUL4 associated factor 7 | 67 | 95 |
| mmu-miR-486a-3p | *Grm2* | glutamate receptor, metabotropic 2 | 68 | 95 |
| mmu-miR-486a-3p | *Lrrc55* | leucine rich repeat containing 55 | 69 | 94 |
| mmu-miR-486a-3p | *Ephb2* | EPH receptor B2 | 70 | 94 |
| mmu-miR-486a-3p | *Scrib* | scribbled planar cell polarity protein | 71 | 94 |
| mmu-miR-486a-3p | *Fam212b* | family with sequence similarity 212, member B | 72 | 94 |
| mmu-miR-486a-3p | *Arhgap26* | Rho GTPase activating protein 26 | 73 | 94 |
| mmu-miR-486a-3p | *Sdc3* | syndecan 3 | 74 | 94 |
| mmu-miR-486a-3p | *Gdi1* | GDP dissociation inhibitor 1 | 75 | 94 |
| mmu-miR-486a-3p | *Vopp1* | vesicular, overexpressed in cancer, prosurvival protein 1 | 76 | 94 |
| mmu-miR-486a-3p | *Nutf2* | nuclear transport factor 2 | 77 | 94 |
| mmu-miR-486a-3p | *Nefh* | neurofilament, heavy polypeptide | 78 | 94 |
| mmu-miR-486a-3p | *Cnp* | 2',3'-cyclic nucleotide 3' phosphodiesterase | 79 | 94 |
| mmu-miR-486a-3p | *Cyb5rl* | cytochrome b5 reductase-like | 80 | 93 |
| mmu-miR-486a-3p | *Gm2a* | GM2 ganglioside activator | 81 | 93 |
| mmu-miR-486a-3p | *Slc9a8* | solute carrier family 9, subfamily A (NHE8, cation proton antiporter 8), member 8 | 82 | 93 |
| mmu-miR-486a-3p | *Pdik1l* | PDLIM1 interacting kinase 1 like | 83 | 93 |
| mmu-miR-486a-3p | *Fam178a* | family with sequence similarity 178, member A | 84 | 93 |
| mmu-miR-486a-3p | *Pafah1b2* | platelet-activating factor acetylhydrolase 1b, catalytic subunit 2 (30kDa) | 85 | 93 |
| mmu-miR-486a-3p | *Sox10* | SRY (sex determining region Y)-box 10 | 86 | 93 |
| mmu-miR-486a-3p | *Dtx3* | deltex 3, E3 ubiquitin ligase | 87 | 93 |
| mmu-miR-486a-3p | *Pxn* | paxillin | 88 | 93 |
| mmu-miR-486a-3p | *Brf1* | BRF1, RNA polymerase III transcription initiation factor 90 kDa subunit | 89 | 93 |
| mmu-miR-486a-3p | *Gata4* | GATA binding protein 4 | 90 | 93 |
| mmu-miR-486a-3p | *Tsc1* | tuberous sclerosis 1 | 91 | 93 |
| mmu-miR-486a-3p | *Slc19a1* | solute carrier family 19 (folate transporter), member 1 | 92 | 93 |
| mmu-miR-486a-3p | *Cyfip2* | cytoplasmic FMR1 interacting protein 2 | 93 | 93 |
| mmu-miR-486a-3p | *Fxyd6* | FXYD domain containing ion transport regulator 6 | 94 | 92 |
| mmu-miR-486a-3p | *Triobp* | TRIO and F-actin binding protein | 95 | 92 |
| mmu-miR-486a-3p | *Adam19* | ADAM metallopeptidase domain 19 | 96 | 92 |
| mmu-miR-486a-3p | *Cpeb2* | cytoplasmic polyadenylation element binding protein 2 | 97 | 92 |
| mmu-miR-486a-3p | *Stx6* | syntaxin 6 | 98 | 92 |
| mmu-miR-486a-3p | *Tmem150a* | transmembrane protein 150A | 99 | 92 |
| mmu-miR-486a-3p | *Pgap3* | post-GPI attachment to proteins 3 | 100 | 92 |
| mmu-miR-486a-3p | *Znf512b* | zinc finger protein 512B | 101 | 92 |
| mmu-miR-486a-3p | *Pex26* | peroxisomal biogenesis factor 26 | 102 | 92 |
| mmu-miR-486a-3p | *Rap1gap2* | RAP1 GTPase activating protein 2 | 103 | 92 |
| mmu-miR-486a-3p | *Zc3h12a* | zinc finger CCCH-type containing 12A | 104 | 92 |
| mmu-miR-486a-3p | *Rims4* | regulating synaptic membrane exocytosis 4 | 105 | 92 |
| mmu-miR-486a-3p | *Cplx2* | complexin 2 | 106 | 92 |
| mmu-miR-486a-3p | *Sort1* | sortilin 1 | 107 | 92 |
| mmu-miR-486a-3p | *Ccdc97* | coiled-coil domain containing 97 | 108 | 92 |
| mmu-miR-486a-3p | *Cbx6* | chromobox homolog 6 | 109 | 92 |
| mmu-miR-486a-3p | *Cbfa2t3* | core-binding factor, runt domain, alpha subunit 2; translocated to, 3 | 110 | 92 |
| mmu-miR-486a-3p | *Dchs1* | dachsous cadherin-related 1 | 111 | 92 |
| mmu-miR-486a-3p | *Ppapdc1b* | phosphatidic acid phosphatase type 2 domain containing 1B | 112 | 91 |
| mmu-miR-486a-3p | *Rfwd3* | ring finger and WD repeat domain 3 | 113 | 91 |
| mmu-miR-486a-3p | *Micall1* | MICAL-like 1 | 114 | 91 |
| mmu-miR-486a-3p | *Dll4* | delta-like 4 (Drosophila) | 115 | 91 |
| mmu-miR-486a-3p | *Pogk* | pogo transposable element with KRAB domain | 116 | 91 |
| mmu-miR-486a-3p | *Ndor1* | NADPH dependent diflavin oxidoreductase 1 | 117 | 91 |
| mmu-miR-486a-3p | *Tub* | tubby bipartite transcription factor | 118 | 91 |
| mmu-miR-486a-3p | *Znf710* | zinc finger protein 710 | 119 | 91 |
| mmu-miR-486a-3p | *Klf12* | Kruppel-like factor 12 | 120 | 91 |
| mmu-miR-486a-3p | *Ulk1* | unc-51 like autophagy activating kinase 1 | 121 | 91 |
| mmu-miR-486a-3p | *Spdef* | SAM pointed domain containing ETS transcription factor | 122 | 91 |
| mmu-miR-486a-3p | *Ppp1r14b* | protein phosphatase 1, regulatory (inhibitor) subunit 14B | 123 | 90 |
| mmu-miR-486a-3p | *Dbf4b* | DBF4 zinc finger B | 124 | 90 |
| mmu-miR-486a-3p | *Clcf1* | cardiotrophin-like cytokine factor 1 | 125 | 90 |
| mmu-miR-486a-3p | *Uap1l1* | UDP-N-acteylglucosamine pyrophosphorylase 1-like 1 | 126 | 90 |
| mmu-miR-486a-3p | *Kcna2* | potassium voltage-gated channel, shaker-related subfamily, member 2 | 127 | 90 |
| mmu-miR-486a-3p | *Lphn1* | latrophilin 1 | 128 | 90 |
| mmu-miR-486a-3p | *Pdgfrb* | platelet-derived growth factor receptor, beta polypeptide | 129 | 90 |
| mmu-miR-486a-3p | *Flnc* | filamin C, gamma | 130 | 90 |
| mmu-miR-486a-3p | *Tspan11* | tetraspanin 11 | 131 | 90 |
| mmu-miR-486a-3p | *Rnf165* | ring finger protein 165 | 132 | 90 |
| mmu-miR-486a-3p | *Mdga1* | MAM domain containing glycosylphosphatidylinositol anchor 1 | 133 | 90 |
| mmu-miR-486a-3p | *Foxp4* | forkhead box P4 | 134 | 90 |
| mmu-miR-486a-3p | *Zdhhc8* | zinc finger, DHHC-type containing 8 | 135 | 89 |
| mmu-miR-486a-3p | *Tanc2* | tetratricopeptide repeat, ankyrin repeat and coiled-coil containing 2 | 136 | 89 |
| mmu-miR-486a-3p | *Chd3* | chromodomain helicase DNA binding protein 3 | 137 | 89 |
| mmu-miR-486a-3p | *Mknk2* | MAP kinase interacting serine/threonine kinase 2 | 138 | 89 |
| mmu-miR-486a-3p | *C17orf62* | chromosome 17 open reading frame 62 | 139 | 89 |
| mmu-miR-486a-3p | *Kmt2d* | lysine (K)-specific methyltransferase 2D | 140 | 89 |
| mmu-miR-486a-3p | *Tspan5* | tetraspanin 5 | 141 | 89 |
| mmu-miR-486a-3p | *Nkiras2* | NFKB inhibitor interacting Ras-like 2 | 142 | 89 |
| mmu-miR-486a-3p | *Zcchc24* | zinc finger, CCHC domain containing 24 | 143 | 89 |
| mmu-miR-486a-3p | *Mdm4* | MDM4, p53 regulator | 144 | 89 |
| mmu-miR-486a-3p | *Bace1* | beta-site APP-cleaving enzyme 1 | 145 | 89 |
| mmu-miR-486a-3p | *Syt2* | synaptotagmin II | 146 | 89 |
| mmu-miR-486a-3p | *Fam155b* | family with sequence similarity 155, member B | 147 | 89 |
| mmu-miR-486a-3p | *Sv2c* | synaptic vesicle glycoprotein 2C | 148 | 89 |
| mmu-miR-486a-3p | *Nkd1* | naked cuticle homolog 1 (Drosophila) | 149 | 89 |
| mmu-miR-486a-3p | *Hspb6* | heat shock protein, alpha-crystallin-related, B6 | 150 | 88 |
| mmu-miR-486a-3p | *Kcnq4* | potassium voltage-gated channel, KQT-like subfamily, member 4 | 151 | 88 |
| mmu-miR-486a-3p | *Tm9sf4* | transmembrane 9 superfamily protein member 4 | 152 | 88 |
| mmu-miR-486a-3p | *Mesdc1* | mesoderm development candidate 1 | 153 | 88 |
| mmu-miR-486a-3p | *Rab2b* | RAB2B, member RAS oncogene family | 154 | 88 |
| mmu-miR-486a-3p | *Wnt5b* | wingless-type MMTV integration site family, member 5B | 155 | 88 |
| mmu-miR-486a-3p | *Gpc6* | glypican 6 | 156 | 88 |
| mmu-miR-486a-3p | *R3hdm4* | R3H domain containing 4 | 157 | 88 |
| mmu-miR-486a-3p | *Prr12* | proline rich 12 | 158 | 88 |
| mmu-miR-486a-3p | *Szrd1* | SUZ RNA binding domain containing 1 | 159 | 88 |
| mmu-miR-486a-3p | *Specc1l* | sperm antigen with calponin homology and coiled-coil domains 1-like | 160 | 88 |
| mmu-miR-486a-3p | *Cbx2* | chromobox homolog 2 | 161 | 88 |
| mmu-miR-486a-3p | *Spock2* | sparc/osteonectin, cwcv and kazal-like domains proteoglycan (testican) 2 | 162 | 88 |
| mmu-miR-486a-3p | *Hes7* | hes family bHLH transcription factor 7 | 163 | 88 |
| mmu-miR-486a-3p | *Rai14* | retinoic acid induced 14 | 164 | 88 |
| mmu-miR-486a-3p | *Vat1* | vesicle amine transport 1 | 165 | 87 |
| mmu-miR-486a-3p | *Gimap4* | GTPase, IMAP family member 4 | 166 | 87 |
| mmu-miR-486a-3p | *Dll3* | delta-like 3 (Drosophila) | 167 | 87 |
| mmu-miR-486a-3p | *Gas7* | growth arrest-specific 7 | 168 | 87 |
| mmu-miR-486a-3p | *Cables1* | Cdk5 and Abl enzyme substrate 1 | 169 | 87 |
| mmu-miR-486a-3p | *Diexf* | digestive organ expansion factor homolog (zebrafish) | 170 | 87 |
| mmu-miR-486a-3p | *Dusp7* | dual specificity phosphatase 7 | 171 | 87 |
| mmu-miR-486a-3p | *Rnf4* | ring finger protein 4 | 172 | 87 |
| mmu-miR-486a-3p | *Kcnab2* | potassium voltage-gated channel, shaker-related subfamily, beta member 2 | 173 | 87 |
| mmu-miR-486a-3p | *Wfikkn2* | WAP, follistatin/kazal, immunoglobulin, kunitz and netrin domain containing 2 | 174 | 87 |
| mmu-miR-486a-3p | *Shisa5* | shisa family member 5 | 175 | 87 |
| mmu-miR-486a-3p | *Stx1b* | syntaxin 1B | 176 | 87 |
| mmu-miR-486a-3p | *Prrt2* | proline-rich transmembrane protein 2 | 177 | 87 |
| mmu-miR-486a-3p | *Dvl3* | dishevelled segment polarity protein 3 | 178 | 87 |
| mmu-miR-486a-3p | *Mgat4b* | mannosyl (alpha-1,3-)-glycoprotein beta-1,4-N-acetylglucosaminyltransferase, isozyme B | 179 | 86 |
| mmu-miR-486a-3p | *Grm4* | glutamate receptor, metabotropic 4 | 180 | 86 |
| mmu-miR-486a-3p | *Tesk2* | testis-specific kinase 2 | 181 | 86 |
| mmu-miR-486a-3p | *Tbc1d20* | TBC1 domain family, member 20 | 182 | 86 |
| mmu-miR-486a-3p | *Sptbn4* | spectrin, beta, non-erythrocytic 4 | 183 | 86 |
| mmu-miR-486a-3p | *Arhgap35* | Rho GTPase activating protein 35 | 184 | 86 |
| mmu-miR-486a-3p | *Dbndd1* | dysbindin (dystrobrevin binding protein 1) domain containing 1 | 185 | 86 |
| mmu-miR-486a-3p | *Crb2* | crumbs family member 2 | 186 | 86 |
| mmu-miR-486a-3p | *Impdh1* | IMP (inosine 5'-monophosphate) dehydrogenase 1 | 187 | 86 |
| mmu-miR-486a-3p | *Dpysl5* | dihydropyrimidinase-like 5 | 188 | 86 |
| mmu-miR-486a-3p | *Celsr3* | cadherin, EGF LAG seven-pass G-type receptor 3 | 189 | 86 |
| mmu-miR-486a-3p | *Ppm1f* | protein phosphatase, Mg2+/Mn2+ dependent, 1F | 190 | 86 |
| mmu-miR-486a-3p | *Smtn* | smoothelin | 191 | 86 |
| mmu-miR-486a-3p | *Ecm1* | extracellular matrix protein 1 | 192 | 86 |
| mmu-miR-486a-3p | *S100a10* | S100 calcium binding protein A10 | 193 | 85 |
| mmu-miR-486a-3p | *Glis2* | GLIS family zinc finger 2 | 194 | 85 |
| mmu-miR-486a-3p | *Ankrd54* | ankyrin repeat domain 54 | 195 | 85 |
| mmu-miR-486a-3p | *Snai3* | snail family zinc finger 3 | 196 | 85 |
| mmu-miR-486a-3p | *Prx* | periaxin | 197 | 85 |
| mmu-miR-486a-3p | *C17orf97* | chromosome 17 open reading frame 97 | 198 | 85 |
| mmu-miR-486a-3p | *Lrp1* | low density lipoprotein receptor-related protein 1 | 199 | 85 |
| mmu-miR-486a-3p | *Mef2d* | myocyte enhancer factor 2D | 200 | 85 |
| mmu-miR-486a-3p | *L1cam* | L1 cell adhesion molecule | 201 | 85 |
| mmu-miR-486a-3p | *Snx32* | sorting nexin 32 | 202 | 85 |
| mmu-miR-486a-3p | *Map3k11* | mitogen-activated protein kinase kinase kinase 11 | 203 | 85 |
| mmu-miR-486a-3p | *Mpp2* | membrane protein, palmitoylated 2 (MAGUK p55 subfamily member 2) | 204 | 85 |
| mmu-miR-486a-3p | *Ksr1* | kinase suppressor of ras 1 | 205 | 85 |
| mmu-miR-486a-3p | *Znf496* | zinc finger protein 496 | 206 | 85 |
| mmu-miR-486a-3p | *Scnn1g* | sodium channel, non-voltage-gated 1, gamma subunit | 207 | 85 |
| mmu-miR-486a-3p | *Src* | v-src avian sarcoma (Schmidt-Ruppin A-2) viral oncogene homolog | 208 | 84 |
| mmu-miR-486a-3p | *Nuf2* | NUF2, NDC80 kinetochore complex component | 209 | 84 |
| mmu-miR-486a-3p | *Tubb* | tubulin, beta class I | 210 | 84 |
| mmu-miR-486a-3p | *Mllt6* | myeloid/lymphoid or mixed-lineage leukemia (trithorax homolog, Drosophila); translocated to, 6 | 211 | 84 |
| mmu-miR-486a-3p | *Prkcg* | protein kinase C, gamma | 212 | 84 |
| mmu-miR-486a-3p | *Cdc25b* | cell division cycle 25B | 213 | 84 |
| mmu-miR-486a-3p | *Coro6* | coronin 6 | 214 | 84 |
| mmu-miR-486a-3p | *Jph1* | junctophilin 1 | 215 | 84 |
| mmu-miR-486a-3p | *Dis3* | DIS3 exosome endoribonuclease and 3'-5' exoribonuclease | 216 | 84 |
| mmu-miR-486a-3p | *Traf3* | TNF receptor-associated factor 3 | 217 | 84 |
| mmu-miR-486a-3p | *Snw1* | SNW domain containing 1 | 218 | 84 |
| mmu-miR-486a-3p | *Ctnnbip1* | catenin, beta interacting protein 1 | 219 | 84 |
| mmu-miR-486a-3p | *Cdc42se1* | CDC42 small effector 1 | 220 | 84 |
| mmu-miR-486a-3p | *Mep1a* | meprin A, alpha (PABA peptide hydrolase) | 221 | 84 |
| mmu-miR-486a-3p | *Kcnq2* | potassium voltage-gated channel, KQT-like subfamily, member 2 | 222 | 84 |
| mmu-miR-486a-3p | *Ctsb* | cathepsin B | 223 | 84 |
| mmu-miR-486a-3p | *Map3k10* | mitogen-activated protein kinase kinase kinase 10 | 224 | 84 |
| mmu-miR-486a-3p | *Il31ra* | interleukin 31 receptor A | 225 | 84 |
| mmu-miR-486a-3p | *Upb1* | ureidopropionase, beta | 226 | 84 |
| mmu-miR-486a-3p | *Bcl11a* | B-cell CLL/lymphoma 11A (zinc finger protein) | 227 | 84 |
| mmu-miR-486a-3p | *Cldn19* | claudin 19 | 228 | 84 |
| mmu-miR-486a-3p | *E2f4* | E2F transcription factor 4, p107/p130-binding | 229 | 84 |
| mmu-miR-486a-3p | *Srgap3* | SLIT-ROBO Rho GTPase activating protein 3 | 230 | 84 |
| mmu-miR-486a-3p | *Phox2a* | paired-like homeobox 2a | 231 | 83 |
| mmu-miR-486a-3p | *Ctdsp1* | CTD (carboxy-terminal domain, RNA polymerase II, polypeptide A) small phosphatase 1 | 232 | 83 |
| mmu-miR-486a-3p | *Tenm4* | teneurin transmembrane protein 4 | 233 | 83 |
| mmu-miR-486a-3p | *Ncan* | neurocan | 234 | 83 |
| mmu-miR-486a-3p | *Palm* | paralemmin | 235 | 83 |
| mmu-miR-486a-3p | *Fosl2* | FOS-like antigen 2 | 236 | 83 |
| mmu-miR-486a-3p | *Slc44a2* | solute carrier family 44 (choline transporter), member 2 | 237 | 83 |
| mmu-miR-486a-3p | *Dnhd1* | dynein heavy chain domain 1 | 238 | 83 |
| mmu-miR-486a-3p | *Gfod1* | glucose-fructose oxidoreductase domain containing 1 | 239 | 83 |
| mmu-miR-486a-3p | *Relt* | RELT tumor necrosis factor receptor | 240 | 83 |
| mmu-miR-486a-3p | *Nfasc* | neurofascin | 241 | 83 |
| mmu-miR-486a-3p | *Tspear* | thrombospondin-type laminin G domain and EAR repeats | 242 | 83 |
| mmu-miR-486a-3p | *Trafd1* | TRAF-type zinc finger domain containing 1 | 243 | 83 |
| mmu-miR-486a-3p | *Rgag4* | retrotransposon gag domain containing 4 | 244 | 83 |
| mmu-miR-486a-3p | *Fgfr1* | fibroblast growth factor receptor 1 | 245 | 82 |
| mmu-miR-486a-3p | *Igsf9* | immunoglobulin superfamily, member 9 | 246 | 82 |
| mmu-miR-486a-3p | *Fam131b* | family with sequence similarity 131, member B | 247 | 82 |
| mmu-miR-486a-3p | *Hif1an* | hypoxia inducible factor 1, alpha subunit inhibitor | 248 | 82 |
| mmu-miR-486a-3p | *Tp53inp2* | tumor protein p53 inducible nuclear protein 2 | 249 | 82 |
| mmu-miR-486a-3p | *C20orf194* | chromosome 20 open reading frame 194 | 250 | 82 |
| mmu-miR-486a-3p | *Sult1b1* | sulfotransferase family, cytosolic, 1B, member 1 | 251 | 82 |
| mmu-miR-486a-3p | *H6pd* | hexose-6-phosphate dehydrogenase (glucose 1-dehydrogenase) | 252 | 82 |
| mmu-miR-486a-3p | *Bmpr2* | bone morphogenetic protein receptor, type II (serine/threonine kinase) | 253 | 82 |
| mmu-miR-486a-3p | *Grik3* | glutamate receptor, ionotropic, kainate 3 | 254 | 82 |
| mmu-miR-486a-3p | *Klhdc3* | kelch domain containing 3 | 255 | 82 |
| mmu-miR-486a-3p | *Mgat5b* | mannosyl (alpha-1,6-)-glycoprotein beta-1,6-N-acetyl-glucosaminyltransferase, isozyme B | 256 | 82 |
| mmu-miR-486a-3p | *Lhfpl4* | lipoma HMGIC fusion partner-like 4 | 257 | 82 |
| mmu-miR-486a-3p | *Usf1* | upstream transcription factor 1 | 258 | 81 |
| mmu-miR-486a-3p | *Gbas* | glioblastoma amplified sequence | 259 | 81 |
| mmu-miR-486a-3p | *Baiap2* | BAI1-associated protein 2 | 260 | 81 |
| mmu-miR-486a-3p | *Sh3glb2* | SH3-domain GRB2-like endophilin B2 | 261 | 81 |
| mmu-miR-486a-3p | *Sec22c* | SEC22 vesicle trafficking protein homolog C (S. cerevisiae) | 262 | 81 |
| mmu-miR-486a-3p | *Doc2a* | double C2-like domains, alpha | 263 | 81 |
| mmu-miR-486a-3p | *Stra6* | stimulated by retinoic acid 6 | 264 | 81 |
| mmu-miR-486a-3p | *Tp73* | tumor protein p73 | 265 | 81 |
| mmu-miR-486a-3p | *Rpl3l* | ribosomal protein L3-like | 266 | 81 |
| mmu-miR-486a-3p | *Soga1* | suppressor of glucose, autophagy associated 1 | 267 | 81 |
| mmu-miR-486a-3p | *Rabl6* | RAB, member RAS oncogene family-like 6 | 268 | 81 |
| mmu-miR-486a-3p | *Dlgap4* | discs, large (Drosophila) homolog-associated protein 4 | 269 | 81 |
| mmu-miR-486a-3p | *Ptk2b* | protein tyrosine kinase 2 beta | 270 | 81 |
| mmu-miR-486a-3p | *Rela* | v-rel avian reticuloendotheliosis viral oncogene homolog A | 271 | 80 |
| mmu-miR-486a-3p | *Snph* | syntaphilin | 272 | 80 |
| mmu-miR-486a-3p | *Vsx1* | visual system homeobox 1 | 273 | 80 |
| mmu-miR-486a-3p | *Stk32b* | serine/threonine kinase 32B | 274 | 80 |
| mmu-miR-486a-3p | *Slc39a13* | solute carrier family 39 (zinc transporter), member 13 | 275 | 80 |
| mmu-miR-486a-3p | *Il2rb* | interleukin 2 receptor, beta | 276 | 80 |
| mmu-miR-486a-3p | *Nhlh1* | nescient helix loop helix 1 | 277 | 80 |
| mmu-miR-486a-3p | *Pax2* | paired box 2 | 278 | 80 |
| mmu-miR-486a-3p | *Anp32a* | acidic (leucine-rich) nuclear phosphoprotein 32 family, member A | 279 | 80 |
| mmu-miR-486a-3p | *Bcl7b* | B-cell CLL/lymphoma 7B | 280 | 80 |
| mmu-miR-486a-3p | *Gmeb2* | glucocorticoid modulatory element binding protein 2 | 281 | 80 |
| mmu-miR-486a-3p | *Rhof* | ras homolog family member F (in filopodia) | 282 | 80 |
| mmu-miR-486a-3p | *Cxcl14* | chemokine (C-X-C motif) ligand 14 | 283 | 80 |
| mmu-miR-486a-3p | *Ctdsp2* | CTD (carboxy-terminal domain, RNA polymerase II, polypeptide A) small phosphatase 2 | 284 | 80 |
| mmu-miR-486a-3p | *Idh3a* | isocitrate dehydrogenase 3 (NAD+) alpha | 285 | 80 |
| mmu-miR-486a-3p | *Fndc5* | fibronectin type III domain containing 5 | 286 | 80 |
| mmu-miR-486a-3p | *Mrvi1* | murine retrovirus integration site 1 homolog | 287 | 80 |
| mmu-miR-486a-3p | *Adamts4* | ADAM metallopeptidase with thrombospondin type 1 motif, 4 | 288 | 80 |
| mmu-miR-486b-5p | [*Abhd17b*](http://www.ncbi.nlm.nih.gov/entrez/query.fcgi?db=gene&cmd=Retrieve&dopt=full_report&list_uids=226016) | abhydrolase domain containing 17B | 1 | 100 |
| mmu-miR-486b-5p | [*Snrpd1*](http://www.ncbi.nlm.nih.gov/entrez/query.fcgi?db=gene&cmd=Retrieve&dopt=full_report&list_uids=20641) | small nuclear ribonucleoprotein D1 | 2 | 99 |
| mmu-miR-486b-5p | [*Srsf3*](http://www.ncbi.nlm.nih.gov/entrez/query.fcgi?db=gene&cmd=Retrieve&dopt=full_report&list_uids=20383) | serine/arginine-rich splicing factor 3 | 3 | 99 |
| mmu-miR-486b-5p | [*Arhgap5*](http://www.ncbi.nlm.nih.gov/entrez/query.fcgi?db=gene&cmd=Retrieve&dopt=full_report&list_uids=11855) | Rho GTPase activating protein 5 | 4 | 99 |
| mmu-miR-486b-5p | [*Sp5*](http://www.ncbi.nlm.nih.gov/entrez/query.fcgi?db=gene&cmd=Retrieve&dopt=full_report&list_uids=64406) | trans-acting transcription factor 5 | 5 | 98 |
| mmu-miR-486b-5p | [*Cadm1*](http://www.ncbi.nlm.nih.gov/entrez/query.fcgi?db=gene&cmd=Retrieve&dopt=full_report&list_uids=54725) | cell adhesion molecule 1 | 6 | 96 |
| mmu-miR-486b-5p | [*Crebrf*](http://www.ncbi.nlm.nih.gov/entrez/query.fcgi?db=gene&cmd=Retrieve&dopt=full_report&list_uids=77128) | CREB3 regulatory factor | 7 | 96 |
| mmu-miR-486b-5p | [*Cops7b*](http://www.ncbi.nlm.nih.gov/entrez/query.fcgi?db=gene&cmd=Retrieve&dopt=full_report&list_uids=26895) | COP9 (constitutive photomorphogenic) homolog, subunit 7b (Arabidopsis thaliana) | 8 | 96 |
| mmu-miR-486b-5p | [*Rab11fip4*](http://www.ncbi.nlm.nih.gov/entrez/query.fcgi?db=gene&cmd=Retrieve&dopt=full_report&list_uids=268451) | RAB11 family interacting protein 4 (class II) | 9 | 96 |
| mmu-miR-486b-5p | [*Gpr153*](http://www.ncbi.nlm.nih.gov/entrez/query.fcgi?db=gene&cmd=Retrieve&dopt=full_report&list_uids=100129) | G protein-coupled receptor 153 | 10 | 96 |
| mmu-miR-486b-5p | [*Slc12a5*](http://www.ncbi.nlm.nih.gov/entrez/query.fcgi?db=gene&cmd=Retrieve&dopt=full_report&list_uids=57138) | solute carrier family 12, member 5 | 11 | 95 |
| mmu-miR-486b-5p | [*Bahcc1*](http://www.ncbi.nlm.nih.gov/entrez/query.fcgi?db=gene&cmd=Retrieve&dopt=full_report&list_uids=268515) | BAH domain and coiled-coil containing 1 | 12 | 94 |
| mmu-miR-486b-5p | [*Tob1*](http://www.ncbi.nlm.nih.gov/entrez/query.fcgi?db=gene&cmd=Retrieve&dopt=full_report&list_uids=22057) | transducer of ErbB-2.1 | 13 | 94 |
| mmu-miR-486b-5p | [*Dlx3*](http://www.ncbi.nlm.nih.gov/entrez/query.fcgi?db=gene&cmd=Retrieve&dopt=full_report&list_uids=13393) | distal-less homeobox 3 | 14 | 94 |
| mmu-miR-486b-5p | [*Bcorl1*](http://www.ncbi.nlm.nih.gov/entrez/query.fcgi?db=gene&cmd=Retrieve&dopt=full_report&list_uids=320376) | BCL6 co-repressor-like 1 | 15 | 94 |
| mmu-miR-486b-5p | [*Yipf5*](http://www.ncbi.nlm.nih.gov/entrez/query.fcgi?db=gene&cmd=Retrieve&dopt=full_report&list_uids=67180) | Yip1 domain family, member 5 | 16 | 93 |
| mmu-miR-486b-5p | [*Mark1*](http://www.ncbi.nlm.nih.gov/entrez/query.fcgi?db=gene&cmd=Retrieve&dopt=full_report&list_uids=226778) | MAP/microtubule affinity-regulating kinase 1 | 17 | 93 |
| mmu-miR-486b-5p | [*Neurod6*](http://www.ncbi.nlm.nih.gov/entrez/query.fcgi?db=gene&cmd=Retrieve&dopt=full_report&list_uids=11922) | neurogenic differentiation 6 | 18 | 93 |
| mmu-miR-486b-5p | [*Atg2b*](http://www.ncbi.nlm.nih.gov/entrez/query.fcgi?db=gene&cmd=Retrieve&dopt=full_report&list_uids=76559) | autophagy related 2B | 19 | 91 |
| mmu-miR-486b-5p | [*Gpr33*](http://www.ncbi.nlm.nih.gov/entrez/query.fcgi?db=gene&cmd=Retrieve&dopt=full_report&list_uids=14762) | G protein-coupled receptor 33 | 20 | 91 |
| mmu-miR-486b-5p | [*Ptpn12*](http://www.ncbi.nlm.nih.gov/entrez/query.fcgi?db=gene&cmd=Retrieve&dopt=full_report&list_uids=19248) | protein tyrosine phosphatase, non-receptor type 12 | 21 | 91 |
| mmu-miR-486b-5p | [*Atxn7l3*](http://www.ncbi.nlm.nih.gov/entrez/query.fcgi?db=gene&cmd=Retrieve&dopt=full_report&list_uids=217218) | ataxin 7-like 3 | 22 | 90 |
| mmu-miR-486b-5p | [*Dcc*](http://www.ncbi.nlm.nih.gov/entrez/query.fcgi?db=gene&cmd=Retrieve&dopt=full_report&list_uids=13176) | deleted in colorectal carcinoma | 23 | 90 |
| mmu-miR-486b-5p | [*Btbd3*](http://www.ncbi.nlm.nih.gov/entrez/query.fcgi?db=gene&cmd=Retrieve&dopt=full_report&list_uids=228662) | BTB (POZ) domain containing 3 | 24 | 90 |
| mmu-miR-486b-5p | [*Smoc1*](http://www.ncbi.nlm.nih.gov/entrez/query.fcgi?db=gene&cmd=Retrieve&dopt=full_report&list_uids=64075) | SPARC related modular calcium binding 1 | 25 | 90 |
| mmu-miR-486b-5p | [*Hgf*](http://www.ncbi.nlm.nih.gov/entrez/query.fcgi?db=gene&cmd=Retrieve&dopt=full_report&list_uids=15234) | hepatocyte growth factor | 26 | 89 |
| mmu-miR-486b-5p | [*Golga3*](http://www.ncbi.nlm.nih.gov/entrez/query.fcgi?db=gene&cmd=Retrieve&dopt=full_report&list_uids=269682) | golgi autoantigen, golgin subfamily a, 3 | 27 | 87 |
| mmu-miR-486b-5p | [*Rpgrip1l*](http://www.ncbi.nlm.nih.gov/entrez/query.fcgi?db=gene&cmd=Retrieve&dopt=full_report&list_uids=244585) | Rpgrip1-like | 28 | 87 |
| mmu-miR-486b-5p | [*Btaf1*](http://www.ncbi.nlm.nih.gov/entrez/query.fcgi?db=gene&cmd=Retrieve&dopt=full_report&list_uids=107182) | BTAF1 RNA polymerase II, B-TFIID transcription factor-associated, (Mot1 homolog, S. cerevisiae) | 29 | 87 |
| mmu-miR-486b-5p | [*Glis1*](http://www.ncbi.nlm.nih.gov/entrez/query.fcgi?db=gene&cmd=Retrieve&dopt=full_report&list_uids=230587) | GLIS family zinc finger 1 | 30 | 87 |
| mmu-miR-486b-5p | [*Slc10a7*](http://www.ncbi.nlm.nih.gov/entrez/query.fcgi?db=gene&cmd=Retrieve&dopt=full_report&list_uids=76775) | solute carrier family 10 (sodium/bile acid cotransporter family), member 7 | 31 | 85 |
| mmu-miR-486b-5p | [*Rin1*](http://www.ncbi.nlm.nih.gov/entrez/query.fcgi?db=gene&cmd=Retrieve&dopt=full_report&list_uids=225870) | Ras and Rab interactor 1 | 32 | 85 |
| mmu-miR-486b-5p | [*Gabra1*](http://www.ncbi.nlm.nih.gov/entrez/query.fcgi?db=gene&cmd=Retrieve&dopt=full_report&list_uids=14394) | gamma-aminobutyric acid (GABA) A receptor, subunit alpha 1 | 33 | 85 |
| mmu-miR-486b-5p | [*Nalcn*](http://www.ncbi.nlm.nih.gov/entrez/query.fcgi?db=gene&cmd=Retrieve&dopt=full_report&list_uids=338370) | sodium leak channel, non-selective | 34 | 84 |
| mmu-miR-486b-5p | [*Nr2c2*](http://www.ncbi.nlm.nih.gov/entrez/query.fcgi?db=gene&cmd=Retrieve&dopt=full_report&list_uids=22026) | nuclear receptor subfamily 2, group C, member 2 | 35 | 84 |
| mmu-miR-486b-5p | [*Tmub2*](http://www.ncbi.nlm.nih.gov/entrez/query.fcgi?db=gene&cmd=Retrieve&dopt=full_report&list_uids=72053) | transmembrane and ubiquitin-like domain containing 2 | 36 | 84 |
| mmu-miR-486b-5p | [*Ncoa6*](http://www.ncbi.nlm.nih.gov/entrez/query.fcgi?db=gene&cmd=Retrieve&dopt=full_report&list_uids=56406) | nuclear receptor coactivator 6 | 37 | 84 |
| mmu-miR-486b-5p | [*Dock3*](http://www.ncbi.nlm.nih.gov/entrez/query.fcgi?db=gene&cmd=Retrieve&dopt=full_report&list_uids=208869) | dedicator of cyto-kinesis 3 | 38 | 84 |
| mmu-miR-486b-5p | [*Mta3*](http://www.ncbi.nlm.nih.gov/entrez/query.fcgi?db=gene&cmd=Retrieve&dopt=full_report&list_uids=116871) | metastasis associated 3 | 39 | 84 |
| mmu-miR-486b-5p | [*Klhl14*](http://www.ncbi.nlm.nih.gov/entrez/query.fcgi?db=gene&cmd=Retrieve&dopt=full_report&list_uids=225266) | kelch-like 14 | 40 | 84 |
| mmu-miR-486b-5p | [*S1pr3*](http://www.ncbi.nlm.nih.gov/entrez/query.fcgi?db=gene&cmd=Retrieve&dopt=full_report&list_uids=13610) | sphingosine-1-phosphate receptor 3 | 41 | 83 |
| mmu-miR-486b-5p | [*Osbpl8*](http://www.ncbi.nlm.nih.gov/entrez/query.fcgi?db=gene&cmd=Retrieve&dopt=full_report&list_uids=237542) | oxysterol binding protein-like 8 | 42 | 83 |
| mmu-miR-486b-5p | [*Ccs*](http://www.ncbi.nlm.nih.gov/entrez/query.fcgi?db=gene&cmd=Retrieve&dopt=full_report&list_uids=12460) | copper chaperone for superoxide dismutase | 43 | 83 |
| mmu-miR-486b-5p | [*Twistnb*](http://www.ncbi.nlm.nih.gov/entrez/query.fcgi?db=gene&cmd=Retrieve&dopt=full_report&list_uids=28071) | TWIST neighbor | 44 | 83 |
| mmu-miR-486b-5p | [*Txlng*](http://www.ncbi.nlm.nih.gov/entrez/query.fcgi?db=gene&cmd=Retrieve&dopt=full_report&list_uids=353170) | taxilin gamma | 45 | 83 |
| mmu-miR-486b-5p | [*Slco2b1*](http://www.ncbi.nlm.nih.gov/entrez/query.fcgi?db=gene&cmd=Retrieve&dopt=full_report&list_uids=101488) | solute carrier organic anion transporter family, member 2b1 | 46 | 82 |
| mmu-miR-486b-5p | [*Pik3r1*](http://www.ncbi.nlm.nih.gov/entrez/query.fcgi?db=gene&cmd=Retrieve&dopt=full_report&list_uids=18708) | phosphatidylinositol 3-kinase, regulatory subunit, polypeptide 1 (p85 alpha) | 47 | 82 |
| mmu-miR-486b-5p | [*Tanc2*](http://www.ncbi.nlm.nih.gov/entrez/query.fcgi?db=gene&cmd=Retrieve&dopt=full_report&list_uids=77097) | tetratricopeptide repeat, ankyrin repeat and coiled-coil containing 2 | 48 | 82 |
| mmu-miR-486b-5p | [*Draxin*](http://www.ncbi.nlm.nih.gov/entrez/query.fcgi?db=gene&cmd=Retrieve&dopt=full_report&list_uids=70433) | dorsal inhibitory axon guidance protein | 49 | 80 |
| mmu-miR-486b-5p | [*Celf2*](http://www.ncbi.nlm.nih.gov/entrez/query.fcgi?db=gene&cmd=Retrieve&dopt=full_report&list_uids=14007) | CUGBP, Elav-like family member 2 | 50 | 80 |
| mmu-miR-486b-5p | [*Pirt*](http://www.ncbi.nlm.nih.gov/entrez/query.fcgi?db=gene&cmd=Retrieve&dopt=full_report&list_uids=193003) | phosphoinositide-interacting regulator of transient receptor potential channels | 51 | 80 |
| mmu-miR-486b-5p | [*Exoc3*](http://www.ncbi.nlm.nih.gov/entrez/query.fcgi?db=gene&cmd=Retrieve&dopt=full_report&list_uids=211446) | exocyst complex component 3 | 52 | 80 |
| mmu-miR-486b-3p | [*Dtx3*](http://www.ncbi.nlm.nih.gov/entrez/query.fcgi?db=gene&cmd=Retrieve&dopt=full_report&list_uids=80904) | deltex 3 homolog (Drosophila) | 1 | 100 |
| mmu-miR-486b-3p | [*Tsc1*](http://www.ncbi.nlm.nih.gov/entrez/query.fcgi?db=gene&cmd=Retrieve&dopt=full_report&list_uids=64930) | tuberous sclerosis 1 | 2 | 100 |
| mmu-miR-486b-3p | [*4930402H24Rik*](http://www.ncbi.nlm.nih.gov/entrez/query.fcgi?db=gene&cmd=Retrieve&dopt=full_report&list_uids=228602) | RIKEN cDNA 4930402H24 gene | 3 | 100 |
| mmu-miR-486b-3p | [*Tulp3*](http://www.ncbi.nlm.nih.gov/entrez/query.fcgi?db=gene&cmd=Retrieve&dopt=full_report&list_uids=22158) | tubby-like protein 3 | 4 | 100 |
| mmu-miR-486b-3p | [*Abcg4*](http://www.ncbi.nlm.nih.gov/entrez/query.fcgi?db=gene&cmd=Retrieve&dopt=full_report&list_uids=192663) | ATP-binding cassette, sub-family G (WHITE), member 4 | 5 | 100 |
| mmu-miR-486b-3p | [*1810041L15Rik*](http://www.ncbi.nlm.nih.gov/entrez/query.fcgi?db=gene&cmd=Retrieve&dopt=full_report&list_uids=72301) | RIKEN cDNA 1810041L15 gene | 6 | 100 |
| mmu-miR-486b-3p | [*Ccdc97*](http://www.ncbi.nlm.nih.gov/entrez/query.fcgi?db=gene&cmd=Retrieve&dopt=full_report&list_uids=52132) | coiled-coil domain containing 97 | 7 | 99 |
| mmu-miR-486b-3p | [*Slc25a42*](http://www.ncbi.nlm.nih.gov/entrez/query.fcgi?db=gene&cmd=Retrieve&dopt=full_report&list_uids=73095) | solute carrier family 25, member 42 | 8 | 99 |
| mmu-miR-486b-3p | [*Gigyf1*](http://www.ncbi.nlm.nih.gov/entrez/query.fcgi?db=gene&cmd=Retrieve&dopt=full_report&list_uids=57330) | GRB10 interacting GYF protein 1 | 9 | 99 |
| mmu-miR-486b-3p | [*Traf3*](http://www.ncbi.nlm.nih.gov/entrez/query.fcgi?db=gene&cmd=Retrieve&dopt=full_report&list_uids=22031) | TNF receptor-associated factor 3 | 10 | 99 |
| mmu-miR-486b-3p | [*Mfn2*](http://www.ncbi.nlm.nih.gov/entrez/query.fcgi?db=gene&cmd=Retrieve&dopt=full_report&list_uids=170731) | mitofusin 2 | 11 | 99 |
| mmu-miR-486b-3p | [*Phb*](http://www.ncbi.nlm.nih.gov/entrez/query.fcgi?db=gene&cmd=Retrieve&dopt=full_report&list_uids=18673) | prohibitin | 12 | 99 |
| mmu-miR-486b-3p | [*Kcna2*](http://www.ncbi.nlm.nih.gov/entrez/query.fcgi?db=gene&cmd=Retrieve&dopt=full_report&list_uids=16490) | potassium voltage-gated channel, shaker-related subfamily, member 2 | 13 | 99 |
| mmu-miR-486b-3p | [*Hmga1-rs1*](http://www.ncbi.nlm.nih.gov/entrez/query.fcgi?db=gene&cmd=Retrieve&dopt=full_report&list_uids=111241) | high mobility group AT-hook I, related sequence 1 | 14 | 99 |
| mmu-miR-486b-3p | [*Itpkb*](http://www.ncbi.nlm.nih.gov/entrez/query.fcgi?db=gene&cmd=Retrieve&dopt=full_report&list_uids=320404) | inositol 1,4,5-trisphosphate 3-kinase B | 15 | 99 |
| mmu-miR-486b-3p | [*Dcakd*](http://www.ncbi.nlm.nih.gov/entrez/query.fcgi?db=gene&cmd=Retrieve&dopt=full_report&list_uids=68087) | dephospho-CoA kinase domain containing | 16 | 99 |
| mmu-miR-486b-3p | [*Anpep*](http://www.ncbi.nlm.nih.gov/entrez/query.fcgi?db=gene&cmd=Retrieve&dopt=full_report&list_uids=16790) | alanyl (membrane) aminopeptidase | 17 | 99 |
| mmu-miR-486b-3p | [*Arid1b*](http://www.ncbi.nlm.nih.gov/entrez/query.fcgi?db=gene&cmd=Retrieve&dopt=full_report&list_uids=239985) | AT rich interactive domain 1B (SWI-like) | 18 | 99 |
| mmu-miR-486b-3p | [*Tmem132e*](http://www.ncbi.nlm.nih.gov/entrez/query.fcgi?db=gene&cmd=Retrieve&dopt=full_report&list_uids=270893) | transmembrane protein 132E | 19 | 98 |
| mmu-miR-486b-3p | [*Ttyh3*](http://www.ncbi.nlm.nih.gov/entrez/query.fcgi?db=gene&cmd=Retrieve&dopt=full_report&list_uids=78339) | tweety homolog 3 (Drosophila) | 20 | 98 |
| mmu-miR-486b-3p | [*Hapln4*](http://www.ncbi.nlm.nih.gov/entrez/query.fcgi?db=gene&cmd=Retrieve&dopt=full_report&list_uids=330790) | hyaluronan and proteoglycan link protein 4 | 21 | 98 |
| mmu-miR-486b-3p | [*Ndor1*](http://www.ncbi.nlm.nih.gov/entrez/query.fcgi?db=gene&cmd=Retrieve&dopt=full_report&list_uids=78797) | NADPH dependent diflavin oxidoreductase 1 | 22 | 98 |
| mmu-miR-486b-3p | [*Aifm2*](http://www.ncbi.nlm.nih.gov/entrez/query.fcgi?db=gene&cmd=Retrieve&dopt=full_report&list_uids=71361) | apoptosis-inducing factor, mitochondrion-associated 2 | 23 | 98 |
| mmu-miR-486b-3p | [*Plcb1*](http://www.ncbi.nlm.nih.gov/entrez/query.fcgi?db=gene&cmd=Retrieve&dopt=full_report&list_uids=18795) | phospholipase C, beta 1 | 24 | 98 |
| mmu-miR-486b-3p | [*Scn2b*](http://www.ncbi.nlm.nih.gov/entrez/query.fcgi?db=gene&cmd=Retrieve&dopt=full_report&list_uids=72821) | sodium channel, voltage-gated, type II, beta | 25 | 98 |
| mmu-miR-486b-3p | [*Rhog*](http://www.ncbi.nlm.nih.gov/entrez/query.fcgi?db=gene&cmd=Retrieve&dopt=full_report&list_uids=56212) | ras homolog gene family, member G | 26 | 98 |
| mmu-miR-486b-3p | [*Idh3a*](http://www.ncbi.nlm.nih.gov/entrez/query.fcgi?db=gene&cmd=Retrieve&dopt=full_report&list_uids=67834) | isocitrate dehydrogenase 3 (NAD+) alpha | 27 | 98 |
| mmu-miR-486b-3p | [*Maf*](http://www.ncbi.nlm.nih.gov/entrez/query.fcgi?db=gene&cmd=Retrieve&dopt=full_report&list_uids=17132) | avian musculoaponeurotic fibrosarcoma (v-maf) AS42 oncogene homolog | 28 | 98 |
| mmu-miR-486b-3p | [*Trak1*](http://www.ncbi.nlm.nih.gov/entrez/query.fcgi?db=gene&cmd=Retrieve&dopt=full_report&list_uids=67095) | trafficking protein, kinesin binding 1 | 29 | 98 |
| mmu-miR-486b-3p | [*Bgn*](http://www.ncbi.nlm.nih.gov/entrez/query.fcgi?db=gene&cmd=Retrieve&dopt=full_report&list_uids=12111) | biglycan | 30 | 98 |
| mmu-miR-486b-3p | [*Tada2b*](http://www.ncbi.nlm.nih.gov/entrez/query.fcgi?db=gene&cmd=Retrieve&dopt=full_report&list_uids=231151) | transcriptional adaptor 2B | 31 | 98 |
| mmu-miR-486b-3p | [*Pcsk4*](http://www.ncbi.nlm.nih.gov/entrez/query.fcgi?db=gene&cmd=Retrieve&dopt=full_report&list_uids=18551) | proprotein convertase subtilisin/kexin type 4 | 32 | 97 |
| mmu-miR-486b-3p | [*Nr5a1*](http://www.ncbi.nlm.nih.gov/entrez/query.fcgi?db=gene&cmd=Retrieve&dopt=full_report&list_uids=26423) | nuclear receptor subfamily 5, group A, member 1 | 33 | 97 |
| mmu-miR-486b-3p | [*Stim1*](http://www.ncbi.nlm.nih.gov/entrez/query.fcgi?db=gene&cmd=Retrieve&dopt=full_report&list_uids=20866) | stromal interaction molecule 1 | 34 | 97 |
| mmu-miR-486b-3p | [*Fbxo41*](http://www.ncbi.nlm.nih.gov/entrez/query.fcgi?db=gene&cmd=Retrieve&dopt=full_report&list_uids=330369) | F-box protein 41 | 35 | 97 |
| mmu-miR-486b-3p | [*Cbfa2t3*](http://www.ncbi.nlm.nih.gov/entrez/query.fcgi?db=gene&cmd=Retrieve&dopt=full_report&list_uids=12398) | core-binding factor, runt domain, alpha subunit 2, translocated to, 3 (human) | 36 | 97 |
| mmu-miR-486b-3p | [*Snx1*](http://www.ncbi.nlm.nih.gov/entrez/query.fcgi?db=gene&cmd=Retrieve&dopt=full_report&list_uids=56440) | sorting nexin 1 | 37 | 97 |
| mmu-miR-486b-3p | [*Flt4*](http://www.ncbi.nlm.nih.gov/entrez/query.fcgi?db=gene&cmd=Retrieve&dopt=full_report&list_uids=14257) | FMS-like tyrosine kinase 4 | 38 | 97 |
| mmu-miR-486b-3p | [*Tmem63c*](http://www.ncbi.nlm.nih.gov/entrez/query.fcgi?db=gene&cmd=Retrieve&dopt=full_report&list_uids=217733) | transmembrane protein 63c | 39 | 97 |
| mmu-miR-486b-3p | [*Kcna1*](http://www.ncbi.nlm.nih.gov/entrez/query.fcgi?db=gene&cmd=Retrieve&dopt=full_report&list_uids=16485) | potassium voltage-gated channel, shaker-related subfamily, member 1 | 40 | 97 |
| mmu-miR-486b-3p | [*Ep300*](http://www.ncbi.nlm.nih.gov/entrez/query.fcgi?db=gene&cmd=Retrieve&dopt=full_report&list_uids=328572) | E1A binding protein p300 | 41 | 97 |
| mmu-miR-486b-3p | [*Syt7*](http://www.ncbi.nlm.nih.gov/entrez/query.fcgi?db=gene&cmd=Retrieve&dopt=full_report&list_uids=54525) | synaptotagmin VII | 42 | 96 |
| mmu-miR-486b-3p | [*Arhgap26*](http://www.ncbi.nlm.nih.gov/entrez/query.fcgi?db=gene&cmd=Retrieve&dopt=full_report&list_uids=71302) | Rho GTPase activating protein 26 | 43 | 96 |
| mmu-miR-486b-3p | [*Mllt6*](http://www.ncbi.nlm.nih.gov/entrez/query.fcgi?db=gene&cmd=Retrieve&dopt=full_report&list_uids=246198) | myeloid/lymphoid or mixed-lineage leukemia (trithorax homolog, Drosophila); translocated to, 6 | 44 | 96 |
| mmu-miR-486b-3p | [*Slc19a1*](http://www.ncbi.nlm.nih.gov/entrez/query.fcgi?db=gene&cmd=Retrieve&dopt=full_report&list_uids=20509) | solute carrier family 19 (folate transporter), member 1 | 45 | 96 |
| mmu-miR-486b-3p | [*Znrf3*](http://www.ncbi.nlm.nih.gov/entrez/query.fcgi?db=gene&cmd=Retrieve&dopt=full_report&list_uids=407821) | zinc and ring finger 3 | 46 | 96 |
| mmu-miR-486b-3p | [*4921536K21Rik*](http://www.ncbi.nlm.nih.gov/entrez/query.fcgi?db=gene&cmd=Retrieve&dopt=full_report&list_uids=67430) | RIKEN cDNA 4921536K21 gene | 47 | 96 |
| mmu-miR-486b-3p | [*Wasf2*](http://www.ncbi.nlm.nih.gov/entrez/query.fcgi?db=gene&cmd=Retrieve&dopt=full_report&list_uids=242687) | WAS protein family, member 2 | 48 | 96 |
| mmu-miR-486b-3p | [*Adarb1*](http://www.ncbi.nlm.nih.gov/entrez/query.fcgi?db=gene&cmd=Retrieve&dopt=full_report&list_uids=110532) | adenosine deaminase, RNA-specific, B1 | 49 | 96 |
| mmu-miR-486b-3p | [*Itga5*](http://www.ncbi.nlm.nih.gov/entrez/query.fcgi?db=gene&cmd=Retrieve&dopt=full_report&list_uids=16402) | integrin alpha 5 (fibronectin receptor alpha) | 50 | 96 |
| mmu-miR-486b-3p | [*Flnc*](http://www.ncbi.nlm.nih.gov/entrez/query.fcgi?db=gene&cmd=Retrieve&dopt=full_report&list_uids=68794) | filamin C, gamma | 51 | 96 |
| mmu-miR-486b-3p | [*Rph3a*](http://www.ncbi.nlm.nih.gov/entrez/query.fcgi?db=gene&cmd=Retrieve&dopt=full_report&list_uids=19894) | rabphilin 3A | 52 | 96 |
| mmu-miR-486b-3p | [*Zfp787*](http://www.ncbi.nlm.nih.gov/entrez/query.fcgi?db=gene&cmd=Retrieve&dopt=full_report&list_uids=67109) | zinc finger protein 787 | 53 | 96 |
| mmu-miR-486b-3p | [*Pvrl1*](http://www.ncbi.nlm.nih.gov/entrez/query.fcgi?db=gene&cmd=Retrieve&dopt=full_report&list_uids=58235) | poliovirus receptor-related 1 | 54 | 96 |
| mmu-miR-486b-3p | [*Map3k11*](http://www.ncbi.nlm.nih.gov/entrez/query.fcgi?db=gene&cmd=Retrieve&dopt=full_report&list_uids=26403) | mitogen-activated protein kinase kinase kinase 11 | 55 | 96 |
| mmu-miR-486b-3p | [*Ccnd2*](http://www.ncbi.nlm.nih.gov/entrez/query.fcgi?db=gene&cmd=Retrieve&dopt=full_report&list_uids=12444) | cyclin D2 | 56 | 96 |
| mmu-miR-486b-3p | [*Klhdc3*](http://www.ncbi.nlm.nih.gov/entrez/query.fcgi?db=gene&cmd=Retrieve&dopt=full_report&list_uids=71765) | kelch domain containing 3 | 57 | 96 |
| mmu-miR-486b-3p | [*Col5a3*](http://www.ncbi.nlm.nih.gov/entrez/query.fcgi?db=gene&cmd=Retrieve&dopt=full_report&list_uids=53867) | collagen, type V, alpha 3 | 58 | 96 |
| mmu-miR-486b-3p | [*Ky*](http://www.ncbi.nlm.nih.gov/entrez/query.fcgi?db=gene&cmd=Retrieve&dopt=full_report&list_uids=16716) | kyphoscoliosis peptidase | 59 | 96 |
| mmu-miR-486b-3p | [*Sgcd*](http://www.ncbi.nlm.nih.gov/entrez/query.fcgi?db=gene&cmd=Retrieve&dopt=full_report&list_uids=24052) | sarcoglycan, delta (dystrophin-associated glycoprotein) | 60 | 96 |
| mmu-miR-486b-3p | [*Nat8l*](http://www.ncbi.nlm.nih.gov/entrez/query.fcgi?db=gene&cmd=Retrieve&dopt=full_report&list_uids=269642) | N-acetyltransferase 8-like | 61 | 95 |
| mmu-miR-486b-3p | [*Bsn*](http://www.ncbi.nlm.nih.gov/entrez/query.fcgi?db=gene&cmd=Retrieve&dopt=full_report&list_uids=12217) | bassoon | 62 | 95 |
| mmu-miR-486b-3p | [*Vamp2*](http://www.ncbi.nlm.nih.gov/entrez/query.fcgi?db=gene&cmd=Retrieve&dopt=full_report&list_uids=22318) | vesicle-associated membrane protein 2 | 63 | 95 |
| mmu-miR-486b-3p | [*Cdc42ep1*](http://www.ncbi.nlm.nih.gov/entrez/query.fcgi?db=gene&cmd=Retrieve&dopt=full_report&list_uids=104445) | CDC42 effector protein (Rho GTPase binding) 1 | 64 | 95 |
| mmu-miR-486b-3p | [*Mark2*](http://www.ncbi.nlm.nih.gov/entrez/query.fcgi?db=gene&cmd=Retrieve&dopt=full_report&list_uids=13728) | MAP/microtubule affinity-regulating kinase 2 | 65 | 95 |
| mmu-miR-486b-3p | [*Kcnq4*](http://www.ncbi.nlm.nih.gov/entrez/query.fcgi?db=gene&cmd=Retrieve&dopt=full_report&list_uids=60613) | potassium voltage-gated channel, subfamily Q, member 4 | 66 | 95 |
| mmu-miR-486b-3p | [*Aptx*](http://www.ncbi.nlm.nih.gov/entrez/query.fcgi?db=gene&cmd=Retrieve&dopt=full_report&list_uids=66408) | aprataxin | 67 | 95 |
| mmu-miR-486b-3p | [*Aif1l*](http://www.ncbi.nlm.nih.gov/entrez/query.fcgi?db=gene&cmd=Retrieve&dopt=full_report&list_uids=108897) | allograft inflammatory factor 1-like | 68 | 95 |
| mmu-miR-486b-3p | [*Glis2*](http://www.ncbi.nlm.nih.gov/entrez/query.fcgi?db=gene&cmd=Retrieve&dopt=full_report&list_uids=83396) | GLIS family zinc finger 2 | 69 | 95 |
| mmu-miR-486b-3p | [*Luzp1*](http://www.ncbi.nlm.nih.gov/entrez/query.fcgi?db=gene&cmd=Retrieve&dopt=full_report&list_uids=269593) | leucine zipper protein 1 | 70 | 95 |
| mmu-miR-486b-3p | [*Fndc5*](http://www.ncbi.nlm.nih.gov/entrez/query.fcgi?db=gene&cmd=Retrieve&dopt=full_report&list_uids=384061) | fibronectin type III domain containing 5 | 71 | 95 |
| mmu-miR-486b-3p | [*Fxyd6*](http://www.ncbi.nlm.nih.gov/entrez/query.fcgi?db=gene&cmd=Retrieve&dopt=full_report&list_uids=59095) | FXYD domain-containing ion transport regulator 6 | 72 | 95 |
| mmu-miR-486b-3p | [*Tcf7l1*](http://www.ncbi.nlm.nih.gov/entrez/query.fcgi?db=gene&cmd=Retrieve&dopt=full_report&list_uids=21415) | transcription factor 7 like 1 (T cell specific, HMG box) | 73 | 94 |
| mmu-miR-486b-3p | [*Cyth4*](http://www.ncbi.nlm.nih.gov/entrez/query.fcgi?db=gene&cmd=Retrieve&dopt=full_report&list_uids=72318) | cytohesin 4 | 74 | 94 |
| mmu-miR-486b-3p | [*Leng8*](http://www.ncbi.nlm.nih.gov/entrez/query.fcgi?db=gene&cmd=Retrieve&dopt=full_report&list_uids=232798) | leukocyte receptor cluster (LRC) member 8 | 75 | 94 |
| mmu-miR-486b-3p | [*Adamts4*](http://www.ncbi.nlm.nih.gov/entrez/query.fcgi?db=gene&cmd=Retrieve&dopt=full_report&list_uids=240913) | a disintegrin-like and metallopeptidase (reprolysin type) with thrombospondin type 1 motif, 4 | 76 | 94 |
| mmu-miR-486b-3p | [*Gdnf*](http://www.ncbi.nlm.nih.gov/entrez/query.fcgi?db=gene&cmd=Retrieve&dopt=full_report&list_uids=14573) | glial cell line derived neurotrophic factor | 77 | 94 |
| mmu-miR-486b-3p | [*Plekha6*](http://www.ncbi.nlm.nih.gov/entrez/query.fcgi?db=gene&cmd=Retrieve&dopt=full_report&list_uids=240753) | pleckstrin homology domain containing, family A member 6 | 78 | 94 |
| mmu-miR-486b-3p | [*Trim47*](http://www.ncbi.nlm.nih.gov/entrez/query.fcgi?db=gene&cmd=Retrieve&dopt=full_report&list_uids=217333) | tripartite motif-containing 47 | 79 | 94 |
| mmu-miR-486b-3p | [*Trabd2b*](http://www.ncbi.nlm.nih.gov/entrez/query.fcgi?db=gene&cmd=Retrieve&dopt=full_report&list_uids=666048) | TraB domain containing 2B | 80 | 94 |
| mmu-miR-486b-3p | [*Psme3*](http://www.ncbi.nlm.nih.gov/entrez/query.fcgi?db=gene&cmd=Retrieve&dopt=full_report&list_uids=19192) | proteaseome (prosome, macropain) activator subunit 3 (PA28 gamma, Ki) | 81 | 94 |
| mmu-miR-486b-3p | [*Vav2*](http://www.ncbi.nlm.nih.gov/entrez/query.fcgi?db=gene&cmd=Retrieve&dopt=full_report&list_uids=22325) | vav 2 oncogene | 82 | 94 |
| mmu-miR-486b-3p | [*Rgma*](http://www.ncbi.nlm.nih.gov/entrez/query.fcgi?db=gene&cmd=Retrieve&dopt=full_report&list_uids=244058) | repulsive guidance molecule family member A | 83 | 94 |
| mmu-miR-486b-3p | [*Snph*](http://www.ncbi.nlm.nih.gov/entrez/query.fcgi?db=gene&cmd=Retrieve&dopt=full_report&list_uids=241727) | syntaphilin | 84 | 94 |
| mmu-miR-486b-3p | [*Rabl6*](http://www.ncbi.nlm.nih.gov/entrez/query.fcgi?db=gene&cmd=Retrieve&dopt=full_report&list_uids=227624) | RAB, member of RAS oncogene family-like 6 | 85 | 93 |
| mmu-miR-486b-3p | [*Il2rb*](http://www.ncbi.nlm.nih.gov/entrez/query.fcgi?db=gene&cmd=Retrieve&dopt=full_report&list_uids=16185) | interleukin 2 receptor, beta chain | 86 | 93 |
| mmu-miR-486b-3p | [*Dgkd*](http://www.ncbi.nlm.nih.gov/entrez/query.fcgi?db=gene&cmd=Retrieve&dopt=full_report&list_uids=227333) | diacylglycerol kinase, delta | 87 | 93 |
| mmu-miR-486b-3p | [*Rerg*](http://www.ncbi.nlm.nih.gov/entrez/query.fcgi?db=gene&cmd=Retrieve&dopt=full_report&list_uids=232441) | RAS-like, estrogen-regulated, growth-inhibitor | 88 | 93 |
| mmu-miR-486b-3p | [*Zfp651*](http://www.ncbi.nlm.nih.gov/entrez/query.fcgi?db=gene&cmd=Retrieve&dopt=full_report&list_uids=270210) | zinc finger protein 651 | 89 | 93 |
| mmu-miR-486b-3p | [*Ctsd*](http://www.ncbi.nlm.nih.gov/entrez/query.fcgi?db=gene&cmd=Retrieve&dopt=full_report&list_uids=13033) | cathepsin D | 90 | 93 |
| mmu-miR-486b-3p | [*Syp*](http://www.ncbi.nlm.nih.gov/entrez/query.fcgi?db=gene&cmd=Retrieve&dopt=full_report&list_uids=20977) | synaptophysin | 91 | 93 |
| mmu-miR-486b-3p | [*Src*](http://www.ncbi.nlm.nih.gov/entrez/query.fcgi?db=gene&cmd=Retrieve&dopt=full_report&list_uids=20779) | Rous sarcoma oncogene | 92 | 93 |
| mmu-miR-486b-3p | [*Agpat1*](http://www.ncbi.nlm.nih.gov/entrez/query.fcgi?db=gene&cmd=Retrieve&dopt=full_report&list_uids=55979) | 1-acylglycerol-3-phosphate O-acyltransferase 1 (lysophosphatidic acid acyltransferase, alpha) | 93 | 92 |
| mmu-miR-486b-3p | [*Kcnq2*](http://www.ncbi.nlm.nih.gov/entrez/query.fcgi?db=gene&cmd=Retrieve&dopt=full_report&list_uids=16536) | potassium voltage-gated channel, subfamily Q, member 2 | 94 | 92 |
| mmu-miR-486b-3p | [*Elfn2*](http://www.ncbi.nlm.nih.gov/entrez/query.fcgi?db=gene&cmd=Retrieve&dopt=full_report&list_uids=207393) | leucine rich repeat and fibronectin type III, extracellular 2 | 95 | 92 |
| mmu-miR-486b-3p | [*Pax2*](http://www.ncbi.nlm.nih.gov/entrez/query.fcgi?db=gene&cmd=Retrieve&dopt=full_report&list_uids=18504) | paired box 2 | 96 | 92 |
| mmu-miR-486b-3p | [*Sp7*](http://www.ncbi.nlm.nih.gov/entrez/query.fcgi?db=gene&cmd=Retrieve&dopt=full_report&list_uids=170574) | Sp7 transcription factor 7 | 97 | 92 |
| mmu-miR-486b-3p | [*Mknk2*](http://www.ncbi.nlm.nih.gov/entrez/query.fcgi?db=gene&cmd=Retrieve&dopt=full_report&list_uids=17347) | MAP kinase-interacting serine/threonine kinase 2 | 98 | 92 |
| mmu-miR-486b-3p | [*Ciita*](http://www.ncbi.nlm.nih.gov/entrez/query.fcgi?db=gene&cmd=Retrieve&dopt=full_report&list_uids=12265) | class II transactivator | 99 | 92 |
| mmu-miR-486b-3p | [*Cpeb2*](http://www.ncbi.nlm.nih.gov/entrez/query.fcgi?db=gene&cmd=Retrieve&dopt=full_report&list_uids=231207) | cytoplasmic polyadenylation element binding protein 2 | 100 | 92 |
| mmu-miR-486b-3p | [*Pknox1*](http://www.ncbi.nlm.nih.gov/entrez/query.fcgi?db=gene&cmd=Retrieve&dopt=full_report&list_uids=18771) | Pbx/knotted 1 homeobox | 101 | 92 |
| mmu-miR-486b-3p | [*Gas7*](http://www.ncbi.nlm.nih.gov/entrez/query.fcgi?db=gene&cmd=Retrieve&dopt=full_report&list_uids=14457) | growth arrest specific 7 | 102 | 92 |
| mmu-miR-486b-3p | [*Tmem150a*](http://www.ncbi.nlm.nih.gov/entrez/query.fcgi?db=gene&cmd=Retrieve&dopt=full_report&list_uids=232086) | transmembrane protein 150A | 103 | 92 |
| mmu-miR-486b-3p | [*Col23a1*](http://www.ncbi.nlm.nih.gov/entrez/query.fcgi?db=gene&cmd=Retrieve&dopt=full_report&list_uids=237759) | collagen, type XXIII, alpha 1 | 104 | 92 |
| mmu-miR-486b-3p | [*Soga1*](http://www.ncbi.nlm.nih.gov/entrez/query.fcgi?db=gene&cmd=Retrieve&dopt=full_report&list_uids=320706) | suppressor of glucose, autophagy associated 1 | 105 | 92 |
| mmu-miR-486b-3p | [*Masp1*](http://www.ncbi.nlm.nih.gov/entrez/query.fcgi?db=gene&cmd=Retrieve&dopt=full_report&list_uids=17174) | mannan-binding lectin serine peptidase 1 | 106 | 91 |
| mmu-miR-486b-3p | [*Slc7a8*](http://www.ncbi.nlm.nih.gov/entrez/query.fcgi?db=gene&cmd=Retrieve&dopt=full_report&list_uids=50934) | solute carrier family 7 (cationic amino acid transporter, y+ system), member 8 | 107 | 91 |
| mmu-miR-486b-3p | [*R3hdml*](http://www.ncbi.nlm.nih.gov/entrez/query.fcgi?db=gene&cmd=Retrieve&dopt=full_report&list_uids=100043899) | R3H domain containing-like | 108 | 91 |
| mmu-miR-486b-3p | [*Ap1b1*](http://www.ncbi.nlm.nih.gov/entrez/query.fcgi?db=gene&cmd=Retrieve&dopt=full_report&list_uids=11764) | adaptor protein complex AP-1, beta 1 subunit | 109 | 91 |
| mmu-miR-486b-3p | [*Bhlha15*](http://www.ncbi.nlm.nih.gov/entrez/query.fcgi?db=gene&cmd=Retrieve&dopt=full_report&list_uids=17341) | basic helix-loop-helix family, member a15 | 110 | 91 |
| mmu-miR-486b-3p | [*Iqce*](http://www.ncbi.nlm.nih.gov/entrez/query.fcgi?db=gene&cmd=Retrieve&dopt=full_report&list_uids=74239) | IQ motif containing E | 111 | 91 |
| mmu-miR-486b-3p | [*Crtc1*](http://www.ncbi.nlm.nih.gov/entrez/query.fcgi?db=gene&cmd=Retrieve&dopt=full_report&list_uids=382056) | CREB regulated transcription coactivator 1 | 112 | 91 |
| mmu-miR-486b-3p | [*Relt*](http://www.ncbi.nlm.nih.gov/entrez/query.fcgi?db=gene&cmd=Retrieve&dopt=full_report&list_uids=320100) | RELT tumor necrosis factor receptor | 113 | 91 |
| mmu-miR-486b-3p | [*Ppard*](http://www.ncbi.nlm.nih.gov/entrez/query.fcgi?db=gene&cmd=Retrieve&dopt=full_report&list_uids=19015) | peroxisome proliferator activator receptor delta | 114 | 91 |
| mmu-miR-486b-3p | [*Fam212b*](http://www.ncbi.nlm.nih.gov/entrez/query.fcgi?db=gene&cmd=Retrieve&dopt=full_report&list_uids=109050) | family with sequence similarity 212, member B | 115 | 91 |
| mmu-miR-486b-3p | [*Carhsp1*](http://www.ncbi.nlm.nih.gov/entrez/query.fcgi?db=gene&cmd=Retrieve&dopt=full_report&list_uids=52502) | calcium regulated heat stable protein 1 | 116 | 91 |
| mmu-miR-486b-3p | [*Lss*](http://www.ncbi.nlm.nih.gov/entrez/query.fcgi?db=gene&cmd=Retrieve&dopt=full_report&list_uids=16987) | lanosterol synthase | 117 | 91 |
| mmu-miR-486b-3p | [*Rorc*](http://www.ncbi.nlm.nih.gov/entrez/query.fcgi?db=gene&cmd=Retrieve&dopt=full_report&list_uids=19885) | RAR-related orphan receptor gamma | 118 | 91 |
| mmu-miR-486b-3p | [*Psenen*](http://www.ncbi.nlm.nih.gov/entrez/query.fcgi?db=gene&cmd=Retrieve&dopt=full_report&list_uids=66340) | presenilin enhancer 2 homolog (C. elegans) | 119 | 91 |
| mmu-miR-486b-3p | [*Bdkrb2*](http://www.ncbi.nlm.nih.gov/entrez/query.fcgi?db=gene&cmd=Retrieve&dopt=full_report&list_uids=12062) | bradykinin receptor, beta 2 | 120 | 91 |
| mmu-miR-486b-3p | [*Arl8a*](http://www.ncbi.nlm.nih.gov/entrez/query.fcgi?db=gene&cmd=Retrieve&dopt=full_report&list_uids=68724) | ADP-ribosylation factor-like 8A | 121 | 90 |
| mmu-miR-486b-3p | [*Rtn4rl2*](http://www.ncbi.nlm.nih.gov/entrez/query.fcgi?db=gene&cmd=Retrieve&dopt=full_report&list_uids=269295) | reticulon 4 receptor-like 2 | 122 | 90 |
| mmu-miR-486b-3p | [*Impdh1*](http://www.ncbi.nlm.nih.gov/entrez/query.fcgi?db=gene&cmd=Retrieve&dopt=full_report&list_uids=23917) | inosine 5'-phosphate dehydrogenase 1 | 123 | 90 |
| mmu-miR-486b-3p | [*Espn*](http://www.ncbi.nlm.nih.gov/entrez/query.fcgi?db=gene&cmd=Retrieve&dopt=full_report&list_uids=56226) | espin | 124 | 90 |
| mmu-miR-486b-3p | [*Pgpep1*](http://www.ncbi.nlm.nih.gov/entrez/query.fcgi?db=gene&cmd=Retrieve&dopt=full_report&list_uids=66522) | pyroglutamyl-peptidase I | 125 | 90 |
| mmu-miR-486b-3p | [*Nefh*](http://www.ncbi.nlm.nih.gov/entrez/query.fcgi?db=gene&cmd=Retrieve&dopt=full_report&list_uids=380684) | neurofilament, heavy polypeptide | 126 | 90 |
| mmu-miR-486b-3p | [*Spdef*](http://www.ncbi.nlm.nih.gov/entrez/query.fcgi?db=gene&cmd=Retrieve&dopt=full_report&list_uids=30051) | SAM pointed domain containing ets transcription factor | 127 | 90 |
| mmu-miR-486b-3p | [*Aatk*](http://www.ncbi.nlm.nih.gov/entrez/query.fcgi?db=gene&cmd=Retrieve&dopt=full_report&list_uids=11302) | apoptosis-associated tyrosine kinase | 128 | 90 |
| mmu-miR-486b-3p | [*Lasp1*](http://www.ncbi.nlm.nih.gov/entrez/query.fcgi?db=gene&cmd=Retrieve&dopt=full_report&list_uids=16796) | LIM and SH3 protein 1 | 129 | 90 |
| mmu-miR-486b-3p | [*Cplx2*](http://www.ncbi.nlm.nih.gov/entrez/query.fcgi?db=gene&cmd=Retrieve&dopt=full_report&list_uids=12890) | complexin 2 | 130 | 90 |
| mmu-miR-486b-3p | [*Bet1*](http://www.ncbi.nlm.nih.gov/entrez/query.fcgi?db=gene&cmd=Retrieve&dopt=full_report&list_uids=12068) | blocked early in transport 1 homolog (S. cerevisiae) | 131 | 90 |
| mmu-miR-486b-3p | [*Sh3kbp1*](http://www.ncbi.nlm.nih.gov/entrez/query.fcgi?db=gene&cmd=Retrieve&dopt=full_report&list_uids=58194) | SH3-domain kinase binding protein 1 | 132 | 90 |
| mmu-miR-486b-3p | [*Lhfpl4*](http://www.ncbi.nlm.nih.gov/entrez/query.fcgi?db=gene&cmd=Retrieve&dopt=full_report&list_uids=269788) | lipoma HMGIC fusion partner-like protein 4 | 133 | 90 |
| mmu-miR-486b-3p | [*Chsy1*](http://www.ncbi.nlm.nih.gov/entrez/query.fcgi?db=gene&cmd=Retrieve&dopt=full_report&list_uids=269941) | chondroitin sulfate synthase 1 | 134 | 90 |
| mmu-miR-486b-3p | [*Fcgr4*](http://www.ncbi.nlm.nih.gov/entrez/query.fcgi?db=gene&cmd=Retrieve&dopt=full_report&list_uids=246256) | Fc receptor, IgG, low affinity IV | 135 | 90 |
| mmu-miR-486b-3p | [*Sec22c*](http://www.ncbi.nlm.nih.gov/entrez/query.fcgi?db=gene&cmd=Retrieve&dopt=full_report&list_uids=215474) | SEC22 vesicle trafficking protein homolog C (S. cerevisiae) | 136 | 90 |
| mmu-miR-486b-3p | [*Sdc3*](http://www.ncbi.nlm.nih.gov/entrez/query.fcgi?db=gene&cmd=Retrieve&dopt=full_report&list_uids=20970) | syndecan 3 | 137 | 90 |
| mmu-miR-486b-3p | [*Ppm1f*](http://www.ncbi.nlm.nih.gov/entrez/query.fcgi?db=gene&cmd=Retrieve&dopt=full_report&list_uids=68606) | protein phosphatase 1F (PP2C domain containing) | 138 | 90 |
| mmu-miR-486b-3p | [*Gnpda1*](http://www.ncbi.nlm.nih.gov/entrez/query.fcgi?db=gene&cmd=Retrieve&dopt=full_report&list_uids=26384) | glucosamine-6-phosphate deaminase 1 | 139 | 90 |
| mmu-miR-486b-3p | [*Cbln3*](http://www.ncbi.nlm.nih.gov/entrez/query.fcgi?db=gene&cmd=Retrieve&dopt=full_report&list_uids=56410) | cerebellin 3 precursor protein | 140 | 90 |
| mmu-miR-486b-3p | [*Dusp7*](http://www.ncbi.nlm.nih.gov/entrez/query.fcgi?db=gene&cmd=Retrieve&dopt=full_report&list_uids=235584) | dual specificity phosphatase 7 | 141 | 90 |
| mmu-miR-486b-3p | [*Clk2*](http://www.ncbi.nlm.nih.gov/entrez/query.fcgi?db=gene&cmd=Retrieve&dopt=full_report&list_uids=12748) | CDC-like kinase 2 | 142 | 89 |
| mmu-miR-486b-3p | [*Fbxo31*](http://www.ncbi.nlm.nih.gov/entrez/query.fcgi?db=gene&cmd=Retrieve&dopt=full_report&list_uids=76454) | F-box protein 31 | 143 | 89 |
| mmu-miR-486b-3p | [*Nr1h2*](http://www.ncbi.nlm.nih.gov/entrez/query.fcgi?db=gene&cmd=Retrieve&dopt=full_report&list_uids=22260) | nuclear receptor subfamily 1, group H, member 2 | 144 | 89 |
| mmu-miR-486b-3p | [*Epn3*](http://www.ncbi.nlm.nih.gov/entrez/query.fcgi?db=gene&cmd=Retrieve&dopt=full_report&list_uids=71889) | epsin 3 | 145 | 89 |
| mmu-miR-486b-3p | [*Dnal4*](http://www.ncbi.nlm.nih.gov/entrez/query.fcgi?db=gene&cmd=Retrieve&dopt=full_report&list_uids=54152) | dynein, axonemal, light chain 4 | 146 | 89 |
| mmu-miR-486b-3p | [*Usp20*](http://www.ncbi.nlm.nih.gov/entrez/query.fcgi?db=gene&cmd=Retrieve&dopt=full_report&list_uids=74270) | ubiquitin specific peptidase 20 | 147 | 89 |
| mmu-miR-486b-3p | [*Zbtb44*](http://www.ncbi.nlm.nih.gov/entrez/query.fcgi?db=gene&cmd=Retrieve&dopt=full_report&list_uids=235132) | zinc finger and BTB domain containing 44 | 148 | 89 |
| mmu-miR-486b-3p | [*Mgat4b*](http://www.ncbi.nlm.nih.gov/entrez/query.fcgi?db=gene&cmd=Retrieve&dopt=full_report&list_uids=103534) | mannoside acetylglucosaminyltransferase 4, isoenzyme B | 149 | 89 |
| mmu-miR-486b-3p | [*Ppig*](http://www.ncbi.nlm.nih.gov/entrez/query.fcgi?db=gene&cmd=Retrieve&dopt=full_report&list_uids=228005) | peptidyl-prolyl isomerase G (cyclophilin G) | 150 | 89 |
| mmu-miR-486b-3p | [*BC037034*](http://www.ncbi.nlm.nih.gov/entrez/query.fcgi?db=gene&cmd=Retrieve&dopt=full_report&list_uids=231807) | cDNA sequence BC037034 | 151 | 89 |
| mmu-miR-486b-3p | [*Tspan9*](http://www.ncbi.nlm.nih.gov/entrez/query.fcgi?db=gene&cmd=Retrieve&dopt=full_report&list_uids=109246) | tetraspanin 9 | 152 | 89 |
| mmu-miR-486b-3p | [*Plbd2*](http://www.ncbi.nlm.nih.gov/entrez/query.fcgi?db=gene&cmd=Retrieve&dopt=full_report&list_uids=71772) | phospholipase B domain containing 2 | 153 | 89 |
| mmu-miR-486b-3p | [*Zc4h2*](http://www.ncbi.nlm.nih.gov/entrez/query.fcgi?db=gene&cmd=Retrieve&dopt=full_report&list_uids=245522) | zinc finger, C4H2 domain containing | 154 | 89 |
| mmu-miR-486b-3p | [*Ppp1ca*](http://www.ncbi.nlm.nih.gov/entrez/query.fcgi?db=gene&cmd=Retrieve&dopt=full_report&list_uids=19045) | protein phosphatase 1, catalytic subunit, alpha isoform | 155 | 89 |
| mmu-miR-486b-3p | [*Tusc2*](http://www.ncbi.nlm.nih.gov/entrez/query.fcgi?db=gene&cmd=Retrieve&dopt=full_report&list_uids=80385) | tumor suppressor candidate 2 | 156 | 88 |
| mmu-miR-486b-3p | [*Spata2*](http://www.ncbi.nlm.nih.gov/entrez/query.fcgi?db=gene&cmd=Retrieve&dopt=full_report&list_uids=263876) | spermatogenesis associated 2 | 157 | 88 |
| mmu-miR-486b-3p | [*Extl3*](http://www.ncbi.nlm.nih.gov/entrez/query.fcgi?db=gene&cmd=Retrieve&dopt=full_report&list_uids=54616) | exostoses (multiple)-like 3 | 158 | 88 |
| mmu-miR-486b-3p | [*Gab1*](http://www.ncbi.nlm.nih.gov/entrez/query.fcgi?db=gene&cmd=Retrieve&dopt=full_report&list_uids=14388) | growth factor receptor bound protein 2-associated protein 1 | 159 | 88 |
| mmu-miR-486b-3p | [*Cd34*](http://www.ncbi.nlm.nih.gov/entrez/query.fcgi?db=gene&cmd=Retrieve&dopt=full_report&list_uids=12490) | CD34 antigen | 160 | 88 |
| mmu-miR-486b-3p | [*Pgap3*](http://www.ncbi.nlm.nih.gov/entrez/query.fcgi?db=gene&cmd=Retrieve&dopt=full_report&list_uids=320655) | post-GPI attachment to proteins 3 | 161 | 88 |
| mmu-miR-486b-3p | [*Usp36*](http://www.ncbi.nlm.nih.gov/entrez/query.fcgi?db=gene&cmd=Retrieve&dopt=full_report&list_uids=72344) | ubiquitin specific peptidase 36 | 162 | 88 |
| mmu-miR-486b-3p | [*Cpsf3l*](http://www.ncbi.nlm.nih.gov/entrez/query.fcgi?db=gene&cmd=Retrieve&dopt=full_report&list_uids=71957) | cleavage and polyadenylation specific factor 3-like | 163 | 88 |
| mmu-miR-486b-3p | [*Stx1b*](http://www.ncbi.nlm.nih.gov/entrez/query.fcgi?db=gene&cmd=Retrieve&dopt=full_report&list_uids=56216) | syntaxin 1B | 164 | 88 |
| mmu-miR-486b-3p | [*Arhgdia*](http://www.ncbi.nlm.nih.gov/entrez/query.fcgi?db=gene&cmd=Retrieve&dopt=full_report&list_uids=192662) | Rho GDP dissociation inhibitor (GDI) alpha | 165 | 88 |
| mmu-miR-486b-3p | [*Ino80d*](http://www.ncbi.nlm.nih.gov/entrez/query.fcgi?db=gene&cmd=Retrieve&dopt=full_report&list_uids=227195) | INO80 complex subunit D | 166 | 88 |
| mmu-miR-486b-3p | [*Zdhhc22*](http://www.ncbi.nlm.nih.gov/entrez/query.fcgi?db=gene&cmd=Retrieve&dopt=full_report&list_uids=238331) | zinc finger, DHHC-type containing 22 | 167 | 87 |
| mmu-miR-486b-3p | [*Cdc25b*](http://www.ncbi.nlm.nih.gov/entrez/query.fcgi?db=gene&cmd=Retrieve&dopt=full_report&list_uids=12531) | cell division cycle 25B | 168 | 87 |
| mmu-miR-486b-3p | [*Zfp710*](http://www.ncbi.nlm.nih.gov/entrez/query.fcgi?db=gene&cmd=Retrieve&dopt=full_report&list_uids=209225) | zinc finger protein 710 | 169 | 87 |
| mmu-miR-486b-3p | [*Rnf44*](http://www.ncbi.nlm.nih.gov/entrez/query.fcgi?db=gene&cmd=Retrieve&dopt=full_report&list_uids=105239) | ring finger protein 44 | 170 | 87 |
| mmu-miR-486b-3p | [*Atp6v0d1*](http://www.ncbi.nlm.nih.gov/entrez/query.fcgi?db=gene&cmd=Retrieve&dopt=full_report&list_uids=11972) | ATPase, H+ transporting, lysosomal V0 subunit D1 | 171 | 87 |
| mmu-miR-486b-3p | [*Szrd1*](http://www.ncbi.nlm.nih.gov/entrez/query.fcgi?db=gene&cmd=Retrieve&dopt=full_report&list_uids=213491) | SUZ RNA binding domain containing 1 | 172 | 87 |
| mmu-miR-486b-3p | [*Mrpl35*](http://www.ncbi.nlm.nih.gov/entrez/query.fcgi?db=gene&cmd=Retrieve&dopt=full_report&list_uids=66223) | mitochondrial ribosomal protein L35 | 173 | 87 |
| mmu-miR-486b-3p | [*Zfp593*](http://www.ncbi.nlm.nih.gov/entrez/query.fcgi?db=gene&cmd=Retrieve&dopt=full_report&list_uids=68040) | zinc finger protein 593 | 174 | 87 |
| mmu-miR-486b-3p | [*Vat1*](http://www.ncbi.nlm.nih.gov/entrez/query.fcgi?db=gene&cmd=Retrieve&dopt=full_report&list_uids=26949) | vesicle amine transport protein 1 homolog (T californica) | 175 | 87 |
| mmu-miR-486b-3p | [*Nkx2-2*](http://www.ncbi.nlm.nih.gov/entrez/query.fcgi?db=gene&cmd=Retrieve&dopt=full_report&list_uids=18088) | NK2 homeobox 2 | 176 | 87 |
| mmu-miR-486b-3p | [*Tanc2*](http://www.ncbi.nlm.nih.gov/entrez/query.fcgi?db=gene&cmd=Retrieve&dopt=full_report&list_uids=77097) | tetratricopeptide repeat, ankyrin repeat and coiled-coil containing 2 | 177 | 87 |
| mmu-miR-486b-3p | [*Spock2*](http://www.ncbi.nlm.nih.gov/entrez/query.fcgi?db=gene&cmd=Retrieve&dopt=full_report&list_uids=94214) | sparc/osteonectin, cwcv and kazal-like domains proteoglycan 2 | 178 | 87 |
| mmu-miR-486b-3p | [*Cd3e*](http://www.ncbi.nlm.nih.gov/entrez/query.fcgi?db=gene&cmd=Retrieve&dopt=full_report&list_uids=12501) | CD3 antigen, epsilon polypeptide | 179 | 87 |
| mmu-miR-486b-3p | [*Wfikkn2*](http://www.ncbi.nlm.nih.gov/entrez/query.fcgi?db=gene&cmd=Retrieve&dopt=full_report&list_uids=278507) | WAP, follistatin/kazal, immunoglobulin, kunitz and netrin domain containing 2 | 180 | 87 |
| mmu-miR-486b-3p | [*Asic1*](http://www.ncbi.nlm.nih.gov/entrez/query.fcgi?db=gene&cmd=Retrieve&dopt=full_report&list_uids=11419) | acid-sensing (proton-gated) ion channel 1 | 181 | 87 |
| mmu-miR-486b-3p | [*Rasgrp3*](http://www.ncbi.nlm.nih.gov/entrez/query.fcgi?db=gene&cmd=Retrieve&dopt=full_report&list_uids=240168) | RAS, guanyl releasing protein 3 | 182 | 87 |
| mmu-miR-486b-3p | [*Sep-09*](http://www.ncbi.nlm.nih.gov/entrez/query.fcgi?db=gene&cmd=Retrieve&dopt=full_report&list_uids=53860) | septin 9 | 183 | 87 |
| mmu-miR-486b-3p | [*Adamts2*](http://www.ncbi.nlm.nih.gov/entrez/query.fcgi?db=gene&cmd=Retrieve&dopt=full_report&list_uids=216725) | a disintegrin-like and metallopeptidase (reprolysin type) with thrombospondin type 1 motif, 2 | 184 | 87 |
| mmu-miR-486b-3p | [*Stra6*](http://www.ncbi.nlm.nih.gov/entrez/query.fcgi?db=gene&cmd=Retrieve&dopt=full_report&list_uids=20897) | stimulated by retinoic acid gene 6 | 185 | 87 |
| mmu-miR-486b-3p | [*Elmo1*](http://www.ncbi.nlm.nih.gov/entrez/query.fcgi?db=gene&cmd=Retrieve&dopt=full_report&list_uids=140580) | engulfment and cell motility 1 | 186 | 86 |
| mmu-miR-486b-3p | [*Cnga2*](http://www.ncbi.nlm.nih.gov/entrez/query.fcgi?db=gene&cmd=Retrieve&dopt=full_report&list_uids=12789) | cyclic nucleotide gated channel alpha 2 | 187 | 86 |
| mmu-miR-486b-3p | [*Hapln3*](http://www.ncbi.nlm.nih.gov/entrez/query.fcgi?db=gene&cmd=Retrieve&dopt=full_report&list_uids=67666) | hyaluronan and proteoglycan link protein 3 | 188 | 86 |
| mmu-miR-486b-3p | [*Grin1*](http://www.ncbi.nlm.nih.gov/entrez/query.fcgi?db=gene&cmd=Retrieve&dopt=full_report&list_uids=14810) | glutamate receptor, ionotropic, NMDA1 (zeta 1) | 189 | 86 |
| mmu-miR-486b-3p | [*Cyb5r3*](http://www.ncbi.nlm.nih.gov/entrez/query.fcgi?db=gene&cmd=Retrieve&dopt=full_report&list_uids=109754) | cytochrome b5 reductase 3 | 190 | 86 |
| mmu-miR-486b-3p | [*Scmh1*](http://www.ncbi.nlm.nih.gov/entrez/query.fcgi?db=gene&cmd=Retrieve&dopt=full_report&list_uids=29871) | sex comb on midleg homolog 1 | 191 | 86 |
| mmu-miR-486b-3p | [*Trappc3*](http://www.ncbi.nlm.nih.gov/entrez/query.fcgi?db=gene&cmd=Retrieve&dopt=full_report&list_uids=27096) | trafficking protein particle complex 3 | 192 | 86 |
| mmu-miR-486b-3p | [*Clmn*](http://www.ncbi.nlm.nih.gov/entrez/query.fcgi?db=gene&cmd=Retrieve&dopt=full_report&list_uids=94040) | calmin | 193 | 86 |
| mmu-miR-486b-3p | [*2010003K11Rik*](http://www.ncbi.nlm.nih.gov/entrez/query.fcgi?db=gene&cmd=Retrieve&dopt=full_report&list_uids=69861) | RIKEN cDNA 2010003K11 gene | 194 | 86 |
| mmu-miR-486b-3p | [*Nol10*](http://www.ncbi.nlm.nih.gov/entrez/query.fcgi?db=gene&cmd=Retrieve&dopt=full_report&list_uids=217431) | nucleolar protein 10 | 195 | 86 |
| mmu-miR-486b-3p | [*Mapre1*](http://www.ncbi.nlm.nih.gov/entrez/query.fcgi?db=gene&cmd=Retrieve&dopt=full_report&list_uids=13589) | microtubule-associated protein, RP/EB family, member 1 | 196 | 86 |
| mmu-miR-486b-3p | [*Tcte1*](http://www.ncbi.nlm.nih.gov/entrez/query.fcgi?db=gene&cmd=Retrieve&dopt=full_report&list_uids=21645) | t-complex-associated testis expressed 1 | 197 | 85 |
| mmu-miR-486b-3p | [*Tubb5*](http://www.ncbi.nlm.nih.gov/entrez/query.fcgi?db=gene&cmd=Retrieve&dopt=full_report&list_uids=22154) | tubulin, beta 5 class I | 198 | 85 |
| mmu-miR-486b-3p | [*Mecp2*](http://www.ncbi.nlm.nih.gov/entrez/query.fcgi?db=gene&cmd=Retrieve&dopt=full_report&list_uids=17257) | methyl CpG binding protein 2 | 199 | 85 |
| mmu-miR-486b-3p | [*Fibcd1*](http://www.ncbi.nlm.nih.gov/entrez/query.fcgi?db=gene&cmd=Retrieve&dopt=full_report&list_uids=98970) | fibrinogen C domain containing 1 | 200 | 85 |
| mmu-miR-486b-3p | [*Cftr*](http://www.ncbi.nlm.nih.gov/entrez/query.fcgi?db=gene&cmd=Retrieve&dopt=full_report&list_uids=12638) | cystic fibrosis transmembrane conductance regulator | 201 | 85 |
| mmu-miR-486b-3p | [*Gatsl2*](http://www.ncbi.nlm.nih.gov/entrez/query.fcgi?db=gene&cmd=Retrieve&dopt=full_report&list_uids=80909) | GATS protein-like 2 | 202 | 85 |
| mmu-miR-486b-3p | [*Ap1s1*](http://www.ncbi.nlm.nih.gov/entrez/query.fcgi?db=gene&cmd=Retrieve&dopt=full_report&list_uids=11769) | adaptor protein complex AP-1, sigma 1 | 203 | 85 |
| mmu-miR-486b-3p | [*Fosl2*](http://www.ncbi.nlm.nih.gov/entrez/query.fcgi?db=gene&cmd=Retrieve&dopt=full_report&list_uids=14284) | fos-like antigen 2 | 204 | 85 |
| mmu-miR-486b-3p | [*Vps9d1*](http://www.ncbi.nlm.nih.gov/entrez/query.fcgi?db=gene&cmd=Retrieve&dopt=full_report&list_uids=72325) | VPS9 domain containing 1 | 205 | 85 |
| mmu-miR-486b-3p | [*Pomt2*](http://www.ncbi.nlm.nih.gov/entrez/query.fcgi?db=gene&cmd=Retrieve&dopt=full_report&list_uids=217734) | protein-O-mannosyltransferase 2 | 206 | 85 |
| mmu-miR-486b-3p | [*Pom121*](http://www.ncbi.nlm.nih.gov/entrez/query.fcgi?db=gene&cmd=Retrieve&dopt=full_report&list_uids=107939) | nuclear pore membrane protein 121 | 207 | 85 |
| mmu-miR-486b-3p | [*Rab11fip3*](http://www.ncbi.nlm.nih.gov/entrez/query.fcgi?db=gene&cmd=Retrieve&dopt=full_report&list_uids=215445) | RAB11 family interacting protein 3 (class II) | 208 | 85 |
| mmu-miR-486b-3p | [*AI597468*](http://www.ncbi.nlm.nih.gov/entrez/query.fcgi?db=gene&cmd=Retrieve&dopt=full_report&list_uids=103266) | expressed sequence AI597468 | 209 | 85 |
| mmu-miR-486b-3p | [*Fkbp5*](http://www.ncbi.nlm.nih.gov/entrez/query.fcgi?db=gene&cmd=Retrieve&dopt=full_report&list_uids=14229) | FK506 binding protein 5 | 210 | 85 |
| mmu-miR-486b-3p | [*Capn6*](http://www.ncbi.nlm.nih.gov/entrez/query.fcgi?db=gene&cmd=Retrieve&dopt=full_report&list_uids=12338) | calpain 6 | 211 | 85 |
| mmu-miR-486b-3p | [*Tm9sf4*](http://www.ncbi.nlm.nih.gov/entrez/query.fcgi?db=gene&cmd=Retrieve&dopt=full_report&list_uids=99237) | transmembrane 9 superfamily protein member 4 | 212 | 85 |
| mmu-miR-486b-3p | [*Clec16a*](http://www.ncbi.nlm.nih.gov/entrez/query.fcgi?db=gene&cmd=Retrieve&dopt=full_report&list_uids=74374) | C-type lectin domain family 16, member A | 213 | 85 |
| mmu-miR-486b-3p | [*Cnn1*](http://www.ncbi.nlm.nih.gov/entrez/query.fcgi?db=gene&cmd=Retrieve&dopt=full_report&list_uids=12797) | calponin 1 | 214 | 85 |
| mmu-miR-486b-3p | [*Csrnp1*](http://www.ncbi.nlm.nih.gov/entrez/query.fcgi?db=gene&cmd=Retrieve&dopt=full_report&list_uids=215418) | cysteine-serine-rich nuclear protein 1 | 215 | 84 |
| mmu-miR-486b-3p | [*Sowaha*](http://www.ncbi.nlm.nih.gov/entrez/query.fcgi?db=gene&cmd=Retrieve&dopt=full_report&list_uids=237761) | sosondowah ankyrin repeat domain family member A | 216 | 84 |
| mmu-miR-486b-3p | [*Suv420h2*](http://www.ncbi.nlm.nih.gov/entrez/query.fcgi?db=gene&cmd=Retrieve&dopt=full_report&list_uids=232811) | suppressor of variegation 4-20 homolog 2 (Drosophila) | 217 | 84 |
| mmu-miR-486b-3p | [*Faim2*](http://www.ncbi.nlm.nih.gov/entrez/query.fcgi?db=gene&cmd=Retrieve&dopt=full_report&list_uids=72393) | Fas apoptotic inhibitory molecule 2 | 218 | 84 |
| mmu-miR-486b-3p | [*Wscd2*](http://www.ncbi.nlm.nih.gov/entrez/query.fcgi?db=gene&cmd=Retrieve&dopt=full_report&list_uids=320916) | WSC domain containing 2 | 219 | 84 |
| mmu-miR-486b-3p | [*Phka1*](http://www.ncbi.nlm.nih.gov/entrez/query.fcgi?db=gene&cmd=Retrieve&dopt=full_report&list_uids=18679) | phosphorylase kinase alpha 1 | 220 | 84 |
| mmu-miR-486b-3p | [*Sod3*](http://www.ncbi.nlm.nih.gov/entrez/query.fcgi?db=gene&cmd=Retrieve&dopt=full_report&list_uids=20657) | superoxide dismutase 3, extracellular | 221 | 84 |
| mmu-miR-486b-3p | [*Slc6a9*](http://www.ncbi.nlm.nih.gov/entrez/query.fcgi?db=gene&cmd=Retrieve&dopt=full_report&list_uids=14664) | solute carrier family 6 (neurotransmitter transporter, glycine), member 9 | 222 | 84 |
| mmu-miR-486b-3p | [*Tomm34*](http://www.ncbi.nlm.nih.gov/entrez/query.fcgi?db=gene&cmd=Retrieve&dopt=full_report&list_uids=67145) | translocase of outer mitochondrial membrane 34 | 223 | 84 |
| mmu-miR-486b-3p | [*Ptk2b*](http://www.ncbi.nlm.nih.gov/entrez/query.fcgi?db=gene&cmd=Retrieve&dopt=full_report&list_uids=19229) | PTK2 protein tyrosine kinase 2 beta | 224 | 84 |
| mmu-miR-486b-3p | [*Syndig1l*](http://www.ncbi.nlm.nih.gov/entrez/query.fcgi?db=gene&cmd=Retrieve&dopt=full_report&list_uids=627191) | synapse differentiation inducing 1 like | 225 | 83 |
| mmu-miR-486b-3p | [*Ndrg4*](http://www.ncbi.nlm.nih.gov/entrez/query.fcgi?db=gene&cmd=Retrieve&dopt=full_report&list_uids=234593) | N-myc downstream regulated gene 4 | 226 | 83 |
| mmu-miR-486b-3p | [*Rhpn1*](http://www.ncbi.nlm.nih.gov/entrez/query.fcgi?db=gene&cmd=Retrieve&dopt=full_report&list_uids=14787) | rhophilin, Rho GTPase binding protein 1 | 227 | 83 |
| mmu-miR-486b-3p | [*Plxna1*](http://www.ncbi.nlm.nih.gov/entrez/query.fcgi?db=gene&cmd=Retrieve&dopt=full_report&list_uids=18844) | plexin A1 | 228 | 83 |
| mmu-miR-486b-3p | [*Cyth2*](http://www.ncbi.nlm.nih.gov/entrez/query.fcgi?db=gene&cmd=Retrieve&dopt=full_report&list_uids=19158) | cytohesin 2 | 229 | 83 |
| mmu-miR-486b-3p | [*Igdcc4*](http://www.ncbi.nlm.nih.gov/entrez/query.fcgi?db=gene&cmd=Retrieve&dopt=full_report&list_uids=56741) | immunoglobulin superfamily, DCC subclass, member 4 | 230 | 83 |
| mmu-miR-486b-3p | [*S100a14*](http://www.ncbi.nlm.nih.gov/entrez/query.fcgi?db=gene&cmd=Retrieve&dopt=full_report&list_uids=66166) | S100 calcium binding protein A14 | 231 | 83 |
| mmu-miR-486b-3p | [*Krt73*](http://www.ncbi.nlm.nih.gov/entrez/query.fcgi?db=gene&cmd=Retrieve&dopt=full_report&list_uids=223915) | keratin 73 | 232 | 83 |
| mmu-miR-486b-3p | [*Surf4*](http://www.ncbi.nlm.nih.gov/entrez/query.fcgi?db=gene&cmd=Retrieve&dopt=full_report&list_uids=20932) | surfeit gene 4 | 233 | 83 |
| mmu-miR-486b-3p | [*Degs2*](http://www.ncbi.nlm.nih.gov/entrez/query.fcgi?db=gene&cmd=Retrieve&dopt=full_report&list_uids=70059) | degenerative spermatocyte homolog 2 (Drosophila), lipid desaturase | 234 | 83 |
| mmu-miR-486b-3p | [*Vipr2*](http://www.ncbi.nlm.nih.gov/entrez/query.fcgi?db=gene&cmd=Retrieve&dopt=full_report&list_uids=22355) | vasoactive intestinal peptide receptor 2 | 235 | 83 |
| mmu-miR-486b-3p | [*Nfyc*](http://www.ncbi.nlm.nih.gov/entrez/query.fcgi?db=gene&cmd=Retrieve&dopt=full_report&list_uids=18046) | nuclear transcription factor-Y gamma | 236 | 83 |
| mmu-miR-486b-3p | [*Pip4k2b*](http://www.ncbi.nlm.nih.gov/entrez/query.fcgi?db=gene&cmd=Retrieve&dopt=full_report&list_uids=108083) | phosphatidylinositol-5-phosphate 4-kinase, type II, beta | 237 | 83 |
| mmu-miR-486b-3p | [*Ace*](http://www.ncbi.nlm.nih.gov/entrez/query.fcgi?db=gene&cmd=Retrieve&dopt=full_report&list_uids=11421) | angiotensin I converting enzyme (peptidyl-dipeptidase A) 1 | 238 | 83 |
| mmu-miR-486b-3p | [*Capn9*](http://www.ncbi.nlm.nih.gov/entrez/query.fcgi?db=gene&cmd=Retrieve&dopt=full_report&list_uids=73647) | calpain 9 | 239 | 83 |
| mmu-miR-486b-3p | [*Pitpnm2*](http://www.ncbi.nlm.nih.gov/entrez/query.fcgi?db=gene&cmd=Retrieve&dopt=full_report&list_uids=19679) | phosphatidylinositol transfer protein, membrane-associated 2 | 240 | 83 |
| mmu-miR-486b-3p | [*Wbp2*](http://www.ncbi.nlm.nih.gov/entrez/query.fcgi?db=gene&cmd=Retrieve&dopt=full_report&list_uids=22378) | WW domain binding protein 2 | 241 | 83 |
| mmu-miR-486b-3p | [*Scly*](http://www.ncbi.nlm.nih.gov/entrez/query.fcgi?db=gene&cmd=Retrieve&dopt=full_report&list_uids=50880) | selenocysteine lyase | 242 | 82 |
| mmu-miR-486b-3p | [*Ifitm1*](http://www.ncbi.nlm.nih.gov/entrez/query.fcgi?db=gene&cmd=Retrieve&dopt=full_report&list_uids=68713) | interferon induced transmembrane protein 1 | 243 | 82 |
| mmu-miR-486b-3p | [*S100a10*](http://www.ncbi.nlm.nih.gov/entrez/query.fcgi?db=gene&cmd=Retrieve&dopt=full_report&list_uids=20194) | S100 calcium binding protein A10 (calpactin) | 244 | 82 |
| mmu-miR-486b-3p | [*Hlcs*](http://www.ncbi.nlm.nih.gov/entrez/query.fcgi?db=gene&cmd=Retrieve&dopt=full_report&list_uids=110948) | holocarboxylase synthetase (biotin- [propriony-Coenzyme A-carboxylase (ATP-hydrolysing)] ligase) | 245 | 82 |
| mmu-miR-486b-3p | [*Zcchc24*](http://www.ncbi.nlm.nih.gov/entrez/query.fcgi?db=gene&cmd=Retrieve&dopt=full_report&list_uids=71918) | zinc finger, CCHC domain containing 24 | 246 | 82 |
| mmu-miR-486b-3p | [*Wipf3*](http://www.ncbi.nlm.nih.gov/entrez/query.fcgi?db=gene&cmd=Retrieve&dopt=full_report&list_uids=330319) | WAS/WASL interacting protein family, member 3 | 247 | 82 |
| mmu-miR-486b-3p | [*Atp13a1*](http://www.ncbi.nlm.nih.gov/entrez/query.fcgi?db=gene&cmd=Retrieve&dopt=full_report&list_uids=170759) | ATPase type 13A1 | 248 | 82 |
| mmu-miR-486b-3p | [*Rab3il1*](http://www.ncbi.nlm.nih.gov/entrez/query.fcgi?db=gene&cmd=Retrieve&dopt=full_report&list_uids=74760) | RAB3A interacting protein (rabin3)-like 1 | 249 | 82 |
| mmu-miR-486b-3p | [*Camk2a*](http://www.ncbi.nlm.nih.gov/entrez/query.fcgi?db=gene&cmd=Retrieve&dopt=full_report&list_uids=12322) | calcium/calmodulin-dependent protein kinase II alpha | 250 | 82 |
| mmu-miR-486b-3p | [*Pard3b*](http://www.ncbi.nlm.nih.gov/entrez/query.fcgi?db=gene&cmd=Retrieve&dopt=full_report&list_uids=72823) | par-3 family cell polarity regulator beta | 251 | 82 |
| mmu-miR-486b-3p | [*Bcl11a*](http://www.ncbi.nlm.nih.gov/entrez/query.fcgi?db=gene&cmd=Retrieve&dopt=full_report&list_uids=14025) | B cell CLL/lymphoma 11A (zinc finger protein) | 252 | 82 |
| mmu-miR-486b-3p | [*Pfkfb4*](http://www.ncbi.nlm.nih.gov/entrez/query.fcgi?db=gene&cmd=Retrieve&dopt=full_report&list_uids=270198) | 6-phosphofructo-2-kinase/fructose-2,6-biphosphatase 4 | 253 | 82 |
| mmu-miR-486b-3p | [*Cdk14*](http://www.ncbi.nlm.nih.gov/entrez/query.fcgi?db=gene&cmd=Retrieve&dopt=full_report&list_uids=18647) | cyclin-dependent kinase 14 | 254 | 82 |
| mmu-miR-486b-3p | [*Nrip1*](http://www.ncbi.nlm.nih.gov/entrez/query.fcgi?db=gene&cmd=Retrieve&dopt=full_report&list_uids=268903) | nuclear receptor interacting protein 1 | 255 | 82 |
| mmu-miR-486b-3p | [*Sppl2b*](http://www.ncbi.nlm.nih.gov/entrez/query.fcgi?db=gene&cmd=Retrieve&dopt=full_report&list_uids=73218) | signal peptide peptidase like 2B | 256 | 82 |
| mmu-miR-486b-3p | [*Acaca*](http://www.ncbi.nlm.nih.gov/entrez/query.fcgi?db=gene&cmd=Retrieve&dopt=full_report&list_uids=107476) | acetyl-Coenzyme A carboxylase alpha | 257 | 81 |
| mmu-miR-486b-3p | [*Smad2*](http://www.ncbi.nlm.nih.gov/entrez/query.fcgi?db=gene&cmd=Retrieve&dopt=full_report&list_uids=17126) | SMAD family member 2 | 258 | 81 |
| mmu-miR-486b-3p | [*Tbc1d22b*](http://www.ncbi.nlm.nih.gov/entrez/query.fcgi?db=gene&cmd=Retrieve&dopt=full_report&list_uids=381085) | TBC1 domain family, member 22B | 259 | 81 |
| mmu-miR-486b-3p | [*Dlx3*](http://www.ncbi.nlm.nih.gov/entrez/query.fcgi?db=gene&cmd=Retrieve&dopt=full_report&list_uids=13393) | distal-less homeobox 3 | 260 | 81 |
| mmu-miR-486b-3p | [*Srsf1*](http://www.ncbi.nlm.nih.gov/entrez/query.fcgi?db=gene&cmd=Retrieve&dopt=full_report&list_uids=110809) | serine/arginine-rich splicing factor 1 | 261 | 81 |
| mmu-miR-486b-3p | [*Edc3*](http://www.ncbi.nlm.nih.gov/entrez/query.fcgi?db=gene&cmd=Retrieve&dopt=full_report&list_uids=353190) | enhancer of mRNA decapping 3 homolog (S. cerevisiae) | 262 | 81 |
| mmu-miR-486b-3p | [*Gprin2*](http://www.ncbi.nlm.nih.gov/entrez/query.fcgi?db=gene&cmd=Retrieve&dopt=full_report&list_uids=432839) | G protein regulated inducer of neurite outgrowth 2 | 263 | 81 |
| mmu-miR-486b-3p | [*Fzd7*](http://www.ncbi.nlm.nih.gov/entrez/query.fcgi?db=gene&cmd=Retrieve&dopt=full_report&list_uids=14369) | frizzled homolog 7 (Drosophila) | 264 | 81 |
| mmu-miR-486b-3p | [*Unk*](http://www.ncbi.nlm.nih.gov/entrez/query.fcgi?db=gene&cmd=Retrieve&dopt=full_report&list_uids=217331) | unkempt homolog (Drosophila) | 265 | 81 |
| mmu-miR-486b-3p | [*Tnks1bp1*](http://www.ncbi.nlm.nih.gov/entrez/query.fcgi?db=gene&cmd=Retrieve&dopt=full_report&list_uids=228140) | tankyrase 1 binding protein 1 | 266 | 81 |
| mmu-miR-486b-3p | [*Pcbp2*](http://www.ncbi.nlm.nih.gov/entrez/query.fcgi?db=gene&cmd=Retrieve&dopt=full_report&list_uids=18521) | poly(rC) binding protein 2 | 267 | 81 |
| mmu-miR-486b-3p | [*Mdga1*](http://www.ncbi.nlm.nih.gov/entrez/query.fcgi?db=gene&cmd=Retrieve&dopt=full_report&list_uids=74762) | MAM domain containing glycosylphosphatidylinositol anchor 1 | 268 | 81 |
| mmu-miR-486b-3p | [*Pemt*](http://www.ncbi.nlm.nih.gov/entrez/query.fcgi?db=gene&cmd=Retrieve&dopt=full_report&list_uids=18618) | phosphatidylethanolamine N-methyltransferase | 269 | 81 |
| mmu-miR-486b-3p | [*Sh3bp5l*](http://www.ncbi.nlm.nih.gov/entrez/query.fcgi?db=gene&cmd=Retrieve&dopt=full_report&list_uids=79566) | SH3 binding domain protein 5 like | 270 | 81 |
| mmu-miR-486b-3p | [*0610030E20Rik*](http://www.ncbi.nlm.nih.gov/entrez/query.fcgi?db=gene&cmd=Retrieve&dopt=full_report&list_uids=68364) | RIKEN cDNA 0610030E20 gene | 271 | 81 |
| mmu-miR-486b-3p | [*Rfwd3*](http://www.ncbi.nlm.nih.gov/entrez/query.fcgi?db=gene&cmd=Retrieve&dopt=full_report&list_uids=234736) | ring finger and WD repeat domain 3 | 272 | 81 |
| mmu-miR-486b-3p | [*Thop1*](http://www.ncbi.nlm.nih.gov/entrez/query.fcgi?db=gene&cmd=Retrieve&dopt=full_report&list_uids=50492) | thimet oligopeptidase 1 | 273 | 81 |
| mmu-miR-486b-3p | [*Pfn2*](http://www.ncbi.nlm.nih.gov/entrez/query.fcgi?db=gene&cmd=Retrieve&dopt=full_report&list_uids=18645) | profilin 2 | 274 | 81 |
| mmu-miR-486b-3p | [*Sox10*](http://www.ncbi.nlm.nih.gov/entrez/query.fcgi?db=gene&cmd=Retrieve&dopt=full_report&list_uids=20665) | SRY (sex determining region Y)-box 10 | 275 | 81 |
| mmu-miR-486b-3p | [*Slc36a1*](http://www.ncbi.nlm.nih.gov/entrez/query.fcgi?db=gene&cmd=Retrieve&dopt=full_report&list_uids=215335) | solute carrier family 36 (proton/amino acid symporter), member 1 | 276 | 80 |
| mmu-miR-486b-3p | [*2310011J03Rik*](http://www.ncbi.nlm.nih.gov/entrez/query.fcgi?db=gene&cmd=Retrieve&dopt=full_report&list_uids=66374) | RIKEN cDNA 2310011J03 gene | 277 | 80 |
| mmu-miR-486b-3p | [*Capn15*](http://www.ncbi.nlm.nih.gov/entrez/query.fcgi?db=gene&cmd=Retrieve&dopt=full_report&list_uids=50817) | calpain 15 | 278 | 80 |
| mmu-miR-486b-3p | [*Smarcc2*](http://www.ncbi.nlm.nih.gov/entrez/query.fcgi?db=gene&cmd=Retrieve&dopt=full_report&list_uids=68094) | SWI/SNF related, matrix associated, actin dependent regulator of chromatin, subfamily c, member 2 | 279 | 80 |
| mmu-miR-486b-3p | [*Tmem119*](http://www.ncbi.nlm.nih.gov/entrez/query.fcgi?db=gene&cmd=Retrieve&dopt=full_report&list_uids=231633) | transmembrane protein 119 | 280 | 80 |
| mmu-miR-486b-3p | [*Zfp609*](http://www.ncbi.nlm.nih.gov/entrez/query.fcgi?db=gene&cmd=Retrieve&dopt=full_report&list_uids=214812) | zinc finger protein 609 | 281 | 80 |
| mmu-miR-486b-3p | [*Trim30b*](http://www.ncbi.nlm.nih.gov/entrez/query.fcgi?db=gene&cmd=Retrieve&dopt=full_report&list_uids=244183) | tripartite motif-containing 30B | 282 | 80 |
| mmu-miR-486b-3p | [*Fam222b*](http://www.ncbi.nlm.nih.gov/entrez/query.fcgi?db=gene&cmd=Retrieve&dopt=full_report&list_uids=216971) | family with sequence similarity 222, member B | 283 | 80 |
| mmu-miR-486b-3p | [*Begain*](http://www.ncbi.nlm.nih.gov/entrez/query.fcgi?db=gene&cmd=Retrieve&dopt=full_report&list_uids=380785) | brain-enriched guanylate kinase-associated | 284 | 80 |
| mmu-miR-486b-3p | [*LOC102640171*](http://www.ncbi.nlm.nih.gov/entrez/query.fcgi?db=gene&cmd=Retrieve&dopt=full_report&list_uids=102640171) | tripartite motif-containing protein 30A-like | 285 | 80 |
| mmu-miR-486b-3p | [*Dcaf7*](http://www.ncbi.nlm.nih.gov/entrez/query.fcgi?db=gene&cmd=Retrieve&dopt=full_report&list_uids=71833) | DDB1 and CUL4 associated factor 7 | 286 | 80 |
| mmu-miR-143 | [*Abl2*](http://www.ncbi.nlm.nih.gov/entrez/query.fcgi?db=gene&cmd=Retrieve&dopt=full_report&list_uids=11352) | v-abl Abelson murine leukemia viral oncogene 2 (arg, Abelson-related gene) | 1 | 100 |
| mmu-miR-143 | [*Igfbp5*](http://www.ncbi.nlm.nih.gov/entrez/query.fcgi?db=gene&cmd=Retrieve&dopt=full_report&list_uids=16011) | insulin-like growth factor binding protein 5 | 2 | 99 |
| mmu-miR-143 | [*Creld1*](http://www.ncbi.nlm.nih.gov/entrez/query.fcgi?db=gene&cmd=Retrieve&dopt=full_report&list_uids=171508) | cysteine-rich with EGF-like domains 1 | 3 | 99 |
| mmu-miR-143 | [*Ahcyl1*](http://www.ncbi.nlm.nih.gov/entrez/query.fcgi?db=gene&cmd=Retrieve&dopt=full_report&list_uids=229709) | S-adenosylhomocysteine hydrolase-like 1 | 4 | 99 |
| mmu-miR-143 | [*Elmod1*](http://www.ncbi.nlm.nih.gov/entrez/query.fcgi?db=gene&cmd=Retrieve&dopt=full_report&list_uids=270162) | ELMO/CED-12 domain containing 1 | 5 | 98 |
| mmu-miR-143 | [*Add3*](http://www.ncbi.nlm.nih.gov/entrez/query.fcgi?db=gene&cmd=Retrieve&dopt=full_report&list_uids=27360) | adducin 3 (gamma) | 6 | 98 |
| mmu-miR-143 | [*Rsrc2*](http://www.ncbi.nlm.nih.gov/entrez/query.fcgi?db=gene&cmd=Retrieve&dopt=full_report&list_uids=208606) | arginine/serine-rich coiled-coil 2 | 7 | 98 |
| mmu-miR-143 | [*Atp10a*](http://www.ncbi.nlm.nih.gov/entrez/query.fcgi?db=gene&cmd=Retrieve&dopt=full_report&list_uids=11982) | ATPase, class V, type 10A | 8 | 98 |
| mmu-miR-143 | [*Lrp12*](http://www.ncbi.nlm.nih.gov/entrez/query.fcgi?db=gene&cmd=Retrieve&dopt=full_report&list_uids=239393) | low density lipoprotein-related protein 12 | 9 | 98 |
| mmu-miR-143 | [*Appl2*](http://www.ncbi.nlm.nih.gov/entrez/query.fcgi?db=gene&cmd=Retrieve&dopt=full_report&list_uids=216190) | adaptor protein, phosphotyrosine interaction, PH domain and leucine zipper containing 2 | 10 | 98 |
| mmu-miR-143 | [*Ptprb*](http://www.ncbi.nlm.nih.gov/entrez/query.fcgi?db=gene&cmd=Retrieve&dopt=full_report&list_uids=19263) | protein tyrosine phosphatase, receptor type, B | 11 | 98 |
| mmu-miR-143 | [*Myo6*](http://www.ncbi.nlm.nih.gov/entrez/query.fcgi?db=gene&cmd=Retrieve&dopt=full_report&list_uids=17920) | myosin VI | 12 | 98 |
| mmu-miR-143 | [*Dennd1b*](http://www.ncbi.nlm.nih.gov/entrez/query.fcgi?db=gene&cmd=Retrieve&dopt=full_report&list_uids=329260) | DENN/MADD domain containing 1B | 13 | 97 |
| mmu-miR-143 | [*Vapb*](http://www.ncbi.nlm.nih.gov/entrez/query.fcgi?db=gene&cmd=Retrieve&dopt=full_report&list_uids=56491) | vesicle-associated membrane protein, associated protein B and C | 14 | 97 |
| mmu-miR-143 | [*Itm2b*](http://www.ncbi.nlm.nih.gov/entrez/query.fcgi?db=gene&cmd=Retrieve&dopt=full_report&list_uids=16432) | integral membrane protein 2B | 15 | 96 |
| mmu-miR-143 | [*Pvrl3*](http://www.ncbi.nlm.nih.gov/entrez/query.fcgi?db=gene&cmd=Retrieve&dopt=full_report&list_uids=58998) | poliovirus receptor-related 3 | 16 | 96 |
| mmu-miR-143 | [*Hlcs*](http://www.ncbi.nlm.nih.gov/entrez/query.fcgi?db=gene&cmd=Retrieve&dopt=full_report&list_uids=110948) | holocarboxylase synthetase (biotin- [propriony-Coenzyme A-carboxylase (ATP-hydrolysing)] ligase) | 17 | 96 |
| mmu-miR-143 | [*Gxylt1*](http://www.ncbi.nlm.nih.gov/entrez/query.fcgi?db=gene&cmd=Retrieve&dopt=full_report&list_uids=223827) | glucoside xylosyltransferase 1 | 18 | 96 |
| mmu-miR-143 | [*Pcx*](http://www.ncbi.nlm.nih.gov/entrez/query.fcgi?db=gene&cmd=Retrieve&dopt=full_report&list_uids=18563) | pyruvate carboxylase | 19 | 96 |
| mmu-miR-143 | [*Psme4*](http://www.ncbi.nlm.nih.gov/entrez/query.fcgi?db=gene&cmd=Retrieve&dopt=full_report&list_uids=103554) | proteasome (prosome, macropain) activator subunit 4 | 20 | 95 |
| mmu-miR-143 | [*A1cf*](http://www.ncbi.nlm.nih.gov/entrez/query.fcgi?db=gene&cmd=Retrieve&dopt=full_report&list_uids=69865) | APOBEC1 complementation factor | 21 | 94 |
| mmu-miR-143 | [*Ttpa*](http://www.ncbi.nlm.nih.gov/entrez/query.fcgi?db=gene&cmd=Retrieve&dopt=full_report&list_uids=50500) | tocopherol (alpha) transfer protein | 22 | 94 |
| mmu-miR-143 | [*Cryz*](http://www.ncbi.nlm.nih.gov/entrez/query.fcgi?db=gene&cmd=Retrieve&dopt=full_report&list_uids=12972) | crystallin, zeta | 23 | 94 |
| mmu-miR-143 | [*Galnt7*](http://www.ncbi.nlm.nih.gov/entrez/query.fcgi?db=gene&cmd=Retrieve&dopt=full_report&list_uids=108150) | UDP-N-acetyl-alpha-D-galactosamine: polypeptide N-acetylgalactosaminyltransferase 7 | 24 | 94 |
| mmu-miR-143 | [*Atp6v1a*](http://www.ncbi.nlm.nih.gov/entrez/query.fcgi?db=gene&cmd=Retrieve&dopt=full_report&list_uids=11964) | ATPase, H+ transporting, lysosomal V1 subunit A | 25 | 93 |
| mmu-miR-143 | [*St8sia3*](http://www.ncbi.nlm.nih.gov/entrez/query.fcgi?db=gene&cmd=Retrieve&dopt=full_report&list_uids=20451) | ST8 alpha-N-acetyl-neuraminide alpha-2,8-sialyltransferase 3 | 26 | 93 |
| mmu-miR-143 | [*Tmem41b*](http://www.ncbi.nlm.nih.gov/entrez/query.fcgi?db=gene&cmd=Retrieve&dopt=full_report&list_uids=233724) | transmembrane protein 41B | 27 | 93 |
| mmu-miR-143 | [*Gabarapl1*](http://www.ncbi.nlm.nih.gov/entrez/query.fcgi?db=gene&cmd=Retrieve&dopt=full_report&list_uids=57436) | gamma-aminobutyric acid (GABA) A receptor-associated protein-like 1 | 28 | 93 |
| mmu-miR-143 | [*Ddr2*](http://www.ncbi.nlm.nih.gov/entrez/query.fcgi?db=gene&cmd=Retrieve&dopt=full_report&list_uids=18214) | discoidin domain receptor family, member 2 | 29 | 92 |
| mmu-miR-143 | [*Cops7a*](http://www.ncbi.nlm.nih.gov/entrez/query.fcgi?db=gene&cmd=Retrieve&dopt=full_report&list_uids=26894) | COP9 (constitutive photomorphogenic) homolog, subunit 7a (Arabidopsis thaliana) | 30 | 92 |
| mmu-miR-143 | [*Rbm24*](http://www.ncbi.nlm.nih.gov/entrez/query.fcgi?db=gene&cmd=Retrieve&dopt=full_report&list_uids=666794) | RNA binding motif protein 24 | 31 | 92 |
| mmu-miR-143 | [*Kras*](http://www.ncbi.nlm.nih.gov/entrez/query.fcgi?db=gene&cmd=Retrieve&dopt=full_report&list_uids=16653) | v-Ki-ras2 Kirsten rat sarcoma viral oncogene homolog | 32 | 92 |
| mmu-miR-143 | [*Atp12a*](http://www.ncbi.nlm.nih.gov/entrez/query.fcgi?db=gene&cmd=Retrieve&dopt=full_report&list_uids=192113) | ATPase, H+/K+ transporting, nongastric, alpha polypeptide | 33 | 92 |
| mmu-miR-143 | [*Zfp583*](http://www.ncbi.nlm.nih.gov/entrez/query.fcgi?db=gene&cmd=Retrieve&dopt=full_report&list_uids=213011) | zinc finger protein 583 | 34 | 91 |
| mmu-miR-143 | [*Klhl20*](http://www.ncbi.nlm.nih.gov/entrez/query.fcgi?db=gene&cmd=Retrieve&dopt=full_report&list_uids=226541) | kelch-like 20 | 35 | 91 |
| mmu-miR-143 | [*Map1b*](http://www.ncbi.nlm.nih.gov/entrez/query.fcgi?db=gene&cmd=Retrieve&dopt=full_report&list_uids=17755) | microtubule-associated protein 1B | 36 | 91 |
| mmu-miR-143 | [*Ppm1e*](http://www.ncbi.nlm.nih.gov/entrez/query.fcgi?db=gene&cmd=Retrieve&dopt=full_report&list_uids=320472) | protein phosphatase 1E (PP2C domain containing) | 37 | 91 |
| mmu-miR-143 | [*Slc7a11*](http://www.ncbi.nlm.nih.gov/entrez/query.fcgi?db=gene&cmd=Retrieve&dopt=full_report&list_uids=26570) | solute carrier family 7 (cationic amino acid transporter, y+ system), member 11 | 38 | 91 |
| mmu-miR-143 | [*Necap1*](http://www.ncbi.nlm.nih.gov/entrez/query.fcgi?db=gene&cmd=Retrieve&dopt=full_report&list_uids=67602) | NECAP endocytosis associated 1 | 39 | 90 |
| mmu-miR-143 | [*Gigyf2*](http://www.ncbi.nlm.nih.gov/entrez/query.fcgi?db=gene&cmd=Retrieve&dopt=full_report&list_uids=227331) | GRB10 interacting GYF protein 2 | 40 | 90 |
| mmu-miR-143 | [*Rdh19*](http://www.ncbi.nlm.nih.gov/entrez/query.fcgi?db=gene&cmd=Retrieve&dopt=full_report&list_uids=216453) | retinol dehydrogenase 19 | 41 | 90 |
| mmu-miR-143 | [*Brd2*](http://www.ncbi.nlm.nih.gov/entrez/query.fcgi?db=gene&cmd=Retrieve&dopt=full_report&list_uids=14312) | bromodomain containing 2 | 42 | 90 |
| mmu-miR-143 | [*Ifih1*](http://www.ncbi.nlm.nih.gov/entrez/query.fcgi?db=gene&cmd=Retrieve&dopt=full_report&list_uids=71586) | interferon induced with helicase C domain 1 | 43 | 90 |
| mmu-miR-143 | [*Larp4*](http://www.ncbi.nlm.nih.gov/entrez/query.fcgi?db=gene&cmd=Retrieve&dopt=full_report&list_uids=207214) | La ribonucleoprotein domain family, member 4 | 44 | 90 |
| mmu-miR-143 | [*Arhgap28*](http://www.ncbi.nlm.nih.gov/entrez/query.fcgi?db=gene&cmd=Retrieve&dopt=full_report&list_uids=268970) | Rho GTPase activating protein 28 | 45 | 90 |
| mmu-miR-143 | [*Dip2b*](http://www.ncbi.nlm.nih.gov/entrez/query.fcgi?db=gene&cmd=Retrieve&dopt=full_report&list_uids=239667) | DIP2 disco-interacting protein 2 homolog B (Drosophila) | 46 | 90 |
| mmu-miR-143 | [*Phf11d*](http://www.ncbi.nlm.nih.gov/entrez/query.fcgi?db=gene&cmd=Retrieve&dopt=full_report&list_uids=219132) | PHD finger protein 11D | 47 | 90 |
| mmu-miR-143 | [*Epm2aip1*](http://www.ncbi.nlm.nih.gov/entrez/query.fcgi?db=gene&cmd=Retrieve&dopt=full_report&list_uids=77781) | EPM2A (laforin) interacting protein 1 | 48 | 89 |
| mmu-miR-143 | [*Nxph1*](http://www.ncbi.nlm.nih.gov/entrez/query.fcgi?db=gene&cmd=Retrieve&dopt=full_report&list_uids=18231) | neurexophilin 1 | 49 | 89 |
| mmu-miR-143 | [*Scamp4*](http://www.ncbi.nlm.nih.gov/entrez/query.fcgi?db=gene&cmd=Retrieve&dopt=full_report&list_uids=56214) | secretory carrier membrane protein 4 | 50 | 89 |
| mmu-miR-143 | [*4930524B15Rik*](http://www.ncbi.nlm.nih.gov/entrez/query.fcgi?db=gene&cmd=Retrieve&dopt=full_report&list_uids=67592) | RIKEN cDNA 4930524B15 gene | 51 | 89 |
| mmu-miR-143 | [*Atp8a2*](http://www.ncbi.nlm.nih.gov/entrez/query.fcgi?db=gene&cmd=Retrieve&dopt=full_report&list_uids=50769) | ATPase, aminophospholipid transporter-like, class I, type 8A, member 2 | 52 | 89 |
| mmu-miR-143 | [*Zfp148*](http://www.ncbi.nlm.nih.gov/entrez/query.fcgi?db=gene&cmd=Retrieve&dopt=full_report&list_uids=22661) | zinc finger protein 148 | 53 | 89 |
| mmu-miR-143 | [*Etv6*](http://www.ncbi.nlm.nih.gov/entrez/query.fcgi?db=gene&cmd=Retrieve&dopt=full_report&list_uids=14011) | ets variant gene 6 (TEL oncogene) | 54 | 89 |
| mmu-miR-143 | [*Tmem121*](http://www.ncbi.nlm.nih.gov/entrez/query.fcgi?db=gene&cmd=Retrieve&dopt=full_report&list_uids=69195) | transmembrane protein 121 | 55 | 88 |
| mmu-miR-143 | [*Whsc1*](http://www.ncbi.nlm.nih.gov/entrez/query.fcgi?db=gene&cmd=Retrieve&dopt=full_report&list_uids=107823) | Wolf-Hirschhorn syndrome candidate 1 (human) | 56 | 88 |
| mmu-miR-143 | [*C77370*](http://www.ncbi.nlm.nih.gov/entrez/query.fcgi?db=gene&cmd=Retrieve&dopt=full_report&list_uids=245555) | expressed sequence C77370 | 57 | 88 |
| mmu-miR-143 | [*Fam134b*](http://www.ncbi.nlm.nih.gov/entrez/query.fcgi?db=gene&cmd=Retrieve&dopt=full_report&list_uids=66270) | family with sequence similarity 134, member B | 58 | 87 |
| mmu-miR-143 | [*Psd3*](http://www.ncbi.nlm.nih.gov/entrez/query.fcgi?db=gene&cmd=Retrieve&dopt=full_report&list_uids=234353) | pleckstrin and Sec7 domain containing 3 | 59 | 87 |
| mmu-miR-143 | [*Tmem170b*](http://www.ncbi.nlm.nih.gov/entrez/query.fcgi?db=gene&cmd=Retrieve&dopt=full_report&list_uids=621976) | transmembrane protein 170B | 60 | 87 |
| mmu-miR-143 | [*Mar-03*](http://www.ncbi.nlm.nih.gov/entrez/query.fcgi?db=gene&cmd=Retrieve&dopt=full_report&list_uids=320253) | membrane-associated ring finger (C3HC4) 3 | 61 | 87 |
| mmu-miR-143 | [*Uqcrfs1*](http://www.ncbi.nlm.nih.gov/entrez/query.fcgi?db=gene&cmd=Retrieve&dopt=full_report&list_uids=66694) | ubiquinol-cytochrome c reductase, Rieske iron-sulfur polypeptide 1 | 62 | 87 |
| mmu-miR-143 | [*Erbb4*](http://www.ncbi.nlm.nih.gov/entrez/query.fcgi?db=gene&cmd=Retrieve&dopt=full_report&list_uids=13869) | v-erb-a erythroblastic leukemia viral oncogene homolog 4 (avian) | 63 | 87 |
| mmu-miR-143 | [*Bmp5*](http://www.ncbi.nlm.nih.gov/entrez/query.fcgi?db=gene&cmd=Retrieve&dopt=full_report&list_uids=12160) | bone morphogenetic protein 5 | 64 | 87 |
| mmu-miR-143 | [*G2e3*](http://www.ncbi.nlm.nih.gov/entrez/query.fcgi?db=gene&cmd=Retrieve&dopt=full_report&list_uids=217558) | G2/M-phase specific E3 ubiquitin ligase | 65 | 86 |
| mmu-miR-143 | [*Ppp3r2*](http://www.ncbi.nlm.nih.gov/entrez/query.fcgi?db=gene&cmd=Retrieve&dopt=full_report&list_uids=19059) | protein phosphatase 3, regulatory subunit B, alpha isoform (calcineurin B, type II) | 66 | 86 |
| mmu-miR-143 | [*Naa30*](http://www.ncbi.nlm.nih.gov/entrez/query.fcgi?db=gene&cmd=Retrieve&dopt=full_report&list_uids=70646) | N(alpha)-acetyltransferase 30, NatC catalytic subunit | 67 | 86 |
| mmu-miR-143 | [*Tmx4*](http://www.ncbi.nlm.nih.gov/entrez/query.fcgi?db=gene&cmd=Retrieve&dopt=full_report&list_uids=52837) | thioredoxin-related transmembrane protein 4 | 68 | 86 |
| mmu-miR-143 | [*Gbas*](http://www.ncbi.nlm.nih.gov/entrez/query.fcgi?db=gene&cmd=Retrieve&dopt=full_report&list_uids=14467) | glioblastoma amplified sequence | 69 | 86 |
| mmu-miR-143 | [*Mab21l1*](http://www.ncbi.nlm.nih.gov/entrez/query.fcgi?db=gene&cmd=Retrieve&dopt=full_report&list_uids=17116) | mab-21-like 1 (C. elegans) | 70 | 85 |
| mmu-miR-143 | [*Itga6*](http://www.ncbi.nlm.nih.gov/entrez/query.fcgi?db=gene&cmd=Retrieve&dopt=full_report&list_uids=16403) | integrin alpha 6 | 71 | 85 |
| mmu-miR-143 | [*Six4*](http://www.ncbi.nlm.nih.gov/entrez/query.fcgi?db=gene&cmd=Retrieve&dopt=full_report&list_uids=20474) | sine oculis-related homeobox 4 | 72 | 85 |
| mmu-miR-143 | [*Cyfip2*](http://www.ncbi.nlm.nih.gov/entrez/query.fcgi?db=gene&cmd=Retrieve&dopt=full_report&list_uids=76884) | cytoplasmic FMR1 interacting protein 2 | 73 | 85 |
| mmu-miR-143 | [*Patl1*](http://www.ncbi.nlm.nih.gov/entrez/query.fcgi?db=gene&cmd=Retrieve&dopt=full_report&list_uids=225929) | protein associated with topoisomerase II homolog 1 (yeast) | 74 | 85 |
| mmu-miR-143 | [*Mcf2*](http://www.ncbi.nlm.nih.gov/entrez/query.fcgi?db=gene&cmd=Retrieve&dopt=full_report&list_uids=109904) | mcf.2 transforming sequence | 75 | 85 |
| mmu-miR-143 | [*Strn3*](http://www.ncbi.nlm.nih.gov/entrez/query.fcgi?db=gene&cmd=Retrieve&dopt=full_report&list_uids=94186) | striatin, calmodulin binding protein 3 | 76 | 85 |
| mmu-miR-143 | [*Strip2*](http://www.ncbi.nlm.nih.gov/entrez/query.fcgi?db=gene&cmd=Retrieve&dopt=full_report&list_uids=320609) | striatin interacting protein 2 | 77 | 85 |
| mmu-miR-143 | [*Gfpt1*](http://www.ncbi.nlm.nih.gov/entrez/query.fcgi?db=gene&cmd=Retrieve&dopt=full_report&list_uids=14583) | glutamine fructose-6-phosphate transaminase 1 | 78 | 85 |
| mmu-miR-143 | [*Ube2e3*](http://www.ncbi.nlm.nih.gov/entrez/query.fcgi?db=gene&cmd=Retrieve&dopt=full_report&list_uids=22193) | ubiquitin-conjugating enzyme E2E 3 | 79 | 85 |
| mmu-miR-143 | [*Egln1*](http://www.ncbi.nlm.nih.gov/entrez/query.fcgi?db=gene&cmd=Retrieve&dopt=full_report&list_uids=112405) | EGL nine homolog 1 (C. elegans) | 80 | 84 |
| mmu-miR-143 | [*Msi2*](http://www.ncbi.nlm.nih.gov/entrez/query.fcgi?db=gene&cmd=Retrieve&dopt=full_report&list_uids=76626) | musashi RNA-binding protein 2 | 81 | 84 |
| mmu-miR-143 | [*Cd226*](http://www.ncbi.nlm.nih.gov/entrez/query.fcgi?db=gene&cmd=Retrieve&dopt=full_report&list_uids=225825) | CD226 antigen | 82 | 84 |
| mmu-miR-143 | [*Slc4a8*](http://www.ncbi.nlm.nih.gov/entrez/query.fcgi?db=gene&cmd=Retrieve&dopt=full_report&list_uids=59033) | solute carrier family 4 (anion exchanger), member 8 | 83 | 83 |
| mmu-miR-143 | [*Ssh2*](http://www.ncbi.nlm.nih.gov/entrez/query.fcgi?db=gene&cmd=Retrieve&dopt=full_report&list_uids=237860) | slingshot homolog 2 (Drosophila) | 84 | 83 |
| mmu-miR-143 | [*Fam117a*](http://www.ncbi.nlm.nih.gov/entrez/query.fcgi?db=gene&cmd=Retrieve&dopt=full_report&list_uids=215512) | family with sequence similarity 117, member A | 85 | 83 |
| mmu-miR-143 | [*Crebzf*](http://www.ncbi.nlm.nih.gov/entrez/query.fcgi?db=gene&cmd=Retrieve&dopt=full_report&list_uids=233490) | CREB/ATF bZIP transcription factor | 86 | 82 |
| mmu-miR-143 | [*Slc16a2*](http://www.ncbi.nlm.nih.gov/entrez/query.fcgi?db=gene&cmd=Retrieve&dopt=full_report&list_uids=20502) | solute carrier family 16 (monocarboxylic acid transporters), member 2 | 87 | 82 |
| mmu-miR-143 | [*Kcnj12*](http://www.ncbi.nlm.nih.gov/entrez/query.fcgi?db=gene&cmd=Retrieve&dopt=full_report&list_uids=16515) | potassium inwardly-rectifying channel, subfamily J, member 12 | 88 | 82 |
| mmu-miR-143 | [*Fgf1*](http://www.ncbi.nlm.nih.gov/entrez/query.fcgi?db=gene&cmd=Retrieve&dopt=full_report&list_uids=14164) | fibroblast growth factor 1 | 89 | 82 |
| mmu-miR-143 | [*Lrrc34*](http://www.ncbi.nlm.nih.gov/entrez/query.fcgi?db=gene&cmd=Retrieve&dopt=full_report&list_uids=71827) | leucine rich repeat containing 34 | 90 | 82 |
| mmu-miR-143 | [*Hoxa5*](http://www.ncbi.nlm.nih.gov/entrez/query.fcgi?db=gene&cmd=Retrieve&dopt=full_report&list_uids=15402) | homeobox A5 | 91 | 82 |
| mmu-miR-143 | [*Frem1*](http://www.ncbi.nlm.nih.gov/entrez/query.fcgi?db=gene&cmd=Retrieve&dopt=full_report&list_uids=329872) | Fras1 related extracellular matrix protein 1 | 92 | 82 |
| mmu-miR-143 | [*Lrrc30*](http://www.ncbi.nlm.nih.gov/entrez/query.fcgi?db=gene&cmd=Retrieve&dopt=full_report&list_uids=240131) | leucine rich repeat containing 30 | 93 | 82 |
| mmu-miR-143 | [*Phf6*](http://www.ncbi.nlm.nih.gov/entrez/query.fcgi?db=gene&cmd=Retrieve&dopt=full_report&list_uids=70998) | PHD finger protein 6 | 94 | 81 |
| mmu-miR-143 | [*Prr3*](http://www.ncbi.nlm.nih.gov/entrez/query.fcgi?db=gene&cmd=Retrieve&dopt=full_report&list_uids=75210) | proline-rich polypeptide 3 | 95 | 81 |
| mmu-miR-143 | [*Orc3*](http://www.ncbi.nlm.nih.gov/entrez/query.fcgi?db=gene&cmd=Retrieve&dopt=full_report&list_uids=50793) | origin recognition complex, subunit 3 | 96 | 81 |
| mmu-miR-143 | [*Smndc1*](http://www.ncbi.nlm.nih.gov/entrez/query.fcgi?db=gene&cmd=Retrieve&dopt=full_report&list_uids=76479) | survival motor neuron domain containing 1 | 97 | 81 |
| mmu-miR-143 | [*Clec7a*](http://www.ncbi.nlm.nih.gov/entrez/query.fcgi?db=gene&cmd=Retrieve&dopt=full_report&list_uids=56644) | C-type lectin domain family 7, member a | 98 | 80 |
| mmu-miR-143 | [*Dgkg*](http://www.ncbi.nlm.nih.gov/entrez/query.fcgi?db=gene&cmd=Retrieve&dopt=full_report&list_uids=110197) | diacylglycerol kinase, gamma | 99 | 80 |
| mmu-miR-143 | [*Fndc3b*](http://www.ncbi.nlm.nih.gov/entrez/query.fcgi?db=gene&cmd=Retrieve&dopt=full_report&list_uids=72007) | fibronectin type III domain containing 3B | 100 | 80 |
| mmu-miR-143 | [*Map9*](http://www.ncbi.nlm.nih.gov/entrez/query.fcgi?db=gene&cmd=Retrieve&dopt=full_report&list_uids=213582) | microtubule-associated protein 9 | 101 | 80 |
| mmu-miR-143 | [*Otud4*](http://www.ncbi.nlm.nih.gov/entrez/query.fcgi?db=gene&cmd=Retrieve&dopt=full_report&list_uids=73945) | OTU domain containing 4 | 102 | 80 |
| mmu-miR-143 | [*Zdhhc21*](http://www.ncbi.nlm.nih.gov/entrez/query.fcgi?db=gene&cmd=Retrieve&dopt=full_report&list_uids=68268) | zinc finger, DHHC domain containing 21 | 103 | 80 |
| mmu-miR-143-3p | [*Abl2*](http://www.ncbi.nlm.nih.gov/entrez/query.fcgi?db=gene&cmd=Retrieve&dopt=full_report&list_uids=11352) | v-abl Abelson murine leukemia viral oncogene 2 (arg, Abelson-related gene) | 1 | 100 |
| mmu-miR-143-3p | [*Igfbp5*](http://www.ncbi.nlm.nih.gov/entrez/query.fcgi?db=gene&cmd=Retrieve&dopt=full_report&list_uids=16011) | insulin-like growth factor binding protein 5 | 2 | 99 |
| mmu-miR-143-3p | [*Creld1*](http://www.ncbi.nlm.nih.gov/entrez/query.fcgi?db=gene&cmd=Retrieve&dopt=full_report&list_uids=171508) | cysteine-rich with EGF-like domains 1 | 3 | 99 |
| mmu-miR-143-3p | [*Ahcyl1*](http://www.ncbi.nlm.nih.gov/entrez/query.fcgi?db=gene&cmd=Retrieve&dopt=full_report&list_uids=229709) | S-adenosylhomocysteine hydrolase-like 1 | 4 | 99 |
| mmu-miR-143-3p | [*Elmod1*](http://www.ncbi.nlm.nih.gov/entrez/query.fcgi?db=gene&cmd=Retrieve&dopt=full_report&list_uids=270162) | ELMO/CED-12 domain containing 1 | 5 | 98 |
| mmu-miR-143-3p | [*Add3*](http://www.ncbi.nlm.nih.gov/entrez/query.fcgi?db=gene&cmd=Retrieve&dopt=full_report&list_uids=27360) | adducin 3 (gamma) | 6 | 98 |
| mmu-miR-143-3p | [*Rsrc2*](http://www.ncbi.nlm.nih.gov/entrez/query.fcgi?db=gene&cmd=Retrieve&dopt=full_report&list_uids=208606) | arginine/serine-rich coiled-coil 2 | 7 | 98 |
| mmu-miR-143-3p | [*Atp10a*](http://www.ncbi.nlm.nih.gov/entrez/query.fcgi?db=gene&cmd=Retrieve&dopt=full_report&list_uids=11982) | ATPase, class V, type 10A | 8 | 98 |
| mmu-miR-143-3p | [*Lrp12*](http://www.ncbi.nlm.nih.gov/entrez/query.fcgi?db=gene&cmd=Retrieve&dopt=full_report&list_uids=239393) | low density lipoprotein-related protein 12 | 9 | 98 |
| mmu-miR-143-3p | [*Appl2*](http://www.ncbi.nlm.nih.gov/entrez/query.fcgi?db=gene&cmd=Retrieve&dopt=full_report&list_uids=216190) | adaptor protein, phosphotyrosine interaction, PH domain and leucine zipper containing 2 | 10 | 98 |
| mmu-miR-143-3p | [*Ptprb*](http://www.ncbi.nlm.nih.gov/entrez/query.fcgi?db=gene&cmd=Retrieve&dopt=full_report&list_uids=19263) | protein tyrosine phosphatase, receptor type, B | 11 | 98 |
| mmu-miR-143-3p | [*Myo6*](http://www.ncbi.nlm.nih.gov/entrez/query.fcgi?db=gene&cmd=Retrieve&dopt=full_report&list_uids=17920) | myosin VI | 12 | 98 |
| mmu-miR-143-3p | [*Dennd1b*](http://www.ncbi.nlm.nih.gov/entrez/query.fcgi?db=gene&cmd=Retrieve&dopt=full_report&list_uids=329260) | DENN/MADD domain containing 1B | 13 | 97 |
| mmu-miR-143-3p | [*Vapb*](http://www.ncbi.nlm.nih.gov/entrez/query.fcgi?db=gene&cmd=Retrieve&dopt=full_report&list_uids=56491) | vesicle-associated membrane protein, associated protein B and C | 14 | 97 |
| mmu-miR-143-3p | [*Itm2b*](http://www.ncbi.nlm.nih.gov/entrez/query.fcgi?db=gene&cmd=Retrieve&dopt=full_report&list_uids=16432) | integral membrane protein 2B | 15 | 96 |
| mmu-miR-143-3p | [*Pvrl3*](http://www.ncbi.nlm.nih.gov/entrez/query.fcgi?db=gene&cmd=Retrieve&dopt=full_report&list_uids=58998) | poliovirus receptor-related 3 | 16 | 96 |
| mmu-miR-143-3p | [*Hlcs*](http://www.ncbi.nlm.nih.gov/entrez/query.fcgi?db=gene&cmd=Retrieve&dopt=full_report&list_uids=110948) | holocarboxylase synthetase (biotin- [propriony-Coenzyme A-carboxylase (ATP-hydrolysing)] ligase) | 17 | 96 |
| mmu-miR-143-3p | [*Gxylt1*](http://www.ncbi.nlm.nih.gov/entrez/query.fcgi?db=gene&cmd=Retrieve&dopt=full_report&list_uids=223827) | glucoside xylosyltransferase 1 | 18 | 96 |
| mmu-miR-143-3p | [*Pcx*](http://www.ncbi.nlm.nih.gov/entrez/query.fcgi?db=gene&cmd=Retrieve&dopt=full_report&list_uids=18563) | pyruvate carboxylase | 19 | 96 |
| mmu-miR-143-3p | [*Psme4*](http://www.ncbi.nlm.nih.gov/entrez/query.fcgi?db=gene&cmd=Retrieve&dopt=full_report&list_uids=103554) | proteasome (prosome, macropain) activator subunit 4 | 20 | 95 |
| mmu-miR-143-3p | [*A1cf*](http://www.ncbi.nlm.nih.gov/entrez/query.fcgi?db=gene&cmd=Retrieve&dopt=full_report&list_uids=69865) | APOBEC1 complementation factor | 21 | 94 |
| mmu-miR-143-3p | [*Ttpa*](http://www.ncbi.nlm.nih.gov/entrez/query.fcgi?db=gene&cmd=Retrieve&dopt=full_report&list_uids=50500) | tocopherol (alpha) transfer protein | 22 | 94 |
| mmu-miR-143-3p | [*Cryz*](http://www.ncbi.nlm.nih.gov/entrez/query.fcgi?db=gene&cmd=Retrieve&dopt=full_report&list_uids=12972) | crystallin, zeta | 23 | 94 |
| mmu-miR-143-3p | [*Galnt7*](http://www.ncbi.nlm.nih.gov/entrez/query.fcgi?db=gene&cmd=Retrieve&dopt=full_report&list_uids=108150) | UDP-N-acetyl-alpha-D-galactosamine: polypeptide N-acetylgalactosaminyltransferase 7 | 24 | 94 |
| mmu-miR-143-3p | [*Atp6v1a*](http://www.ncbi.nlm.nih.gov/entrez/query.fcgi?db=gene&cmd=Retrieve&dopt=full_report&list_uids=11964) | ATPase, H+ transporting, lysosomal V1 subunit A | 25 | 93 |
| mmu-miR-143-3p | [*St8sia3*](http://www.ncbi.nlm.nih.gov/entrez/query.fcgi?db=gene&cmd=Retrieve&dopt=full_report&list_uids=20451) | ST8 alpha-N-acetyl-neuraminide alpha-2,8-sialyltransferase 3 | 26 | 93 |
| mmu-miR-143-3p | [*Tmem41b*](http://www.ncbi.nlm.nih.gov/entrez/query.fcgi?db=gene&cmd=Retrieve&dopt=full_report&list_uids=233724) | transmembrane protein 41B | 27 | 93 |
| mmu-miR-143-3p | [*Gabarapl1*](http://www.ncbi.nlm.nih.gov/entrez/query.fcgi?db=gene&cmd=Retrieve&dopt=full_report&list_uids=57436) | gamma-aminobutyric acid (GABA) A receptor-associated protein-like 1 | 28 | 93 |
| mmu-miR-143-3p | [*Ddr2*](http://www.ncbi.nlm.nih.gov/entrez/query.fcgi?db=gene&cmd=Retrieve&dopt=full_report&list_uids=18214) | discoidin domain receptor family, member 2 | 29 | 92 |
| mmu-miR-143-3p | [*Cops7a*](http://www.ncbi.nlm.nih.gov/entrez/query.fcgi?db=gene&cmd=Retrieve&dopt=full_report&list_uids=26894) | COP9 (constitutive photomorphogenic) homolog, subunit 7a (Arabidopsis thaliana) | 30 | 92 |
| mmu-miR-143-3p | [*Rbm24*](http://www.ncbi.nlm.nih.gov/entrez/query.fcgi?db=gene&cmd=Retrieve&dopt=full_report&list_uids=666794) | RNA binding motif protein 24 | 31 | 92 |
| mmu-miR-143-3p | [*Kras*](http://www.ncbi.nlm.nih.gov/entrez/query.fcgi?db=gene&cmd=Retrieve&dopt=full_report&list_uids=16653) | v-Ki-ras2 Kirsten rat sarcoma viral oncogene homolog | 32 | 92 |
| mmu-miR-143-3p | [*Atp12a*](http://www.ncbi.nlm.nih.gov/entrez/query.fcgi?db=gene&cmd=Retrieve&dopt=full_report&list_uids=192113) | ATPase, H+/K+ transporting, nongastric, alpha polypeptide | 33 | 92 |
| mmu-miR-143-3p | [*Zfp583*](http://www.ncbi.nlm.nih.gov/entrez/query.fcgi?db=gene&cmd=Retrieve&dopt=full_report&list_uids=213011) | zinc finger protein 583 | 34 | 91 |
| mmu-miR-143-3p | [*Klhl20*](http://www.ncbi.nlm.nih.gov/entrez/query.fcgi?db=gene&cmd=Retrieve&dopt=full_report&list_uids=226541) | kelch-like 20 | 35 | 91 |
| mmu-miR-143-3p | [*Map1b*](http://www.ncbi.nlm.nih.gov/entrez/query.fcgi?db=gene&cmd=Retrieve&dopt=full_report&list_uids=17755) | microtubule-associated protein 1B | 36 | 91 |
| mmu-miR-143-3p | [*Ppm1e*](http://www.ncbi.nlm.nih.gov/entrez/query.fcgi?db=gene&cmd=Retrieve&dopt=full_report&list_uids=320472) | protein phosphatase 1E (PP2C domain containing) | 37 | 91 |
| mmu-miR-143-3p | [*Slc7a11*](http://www.ncbi.nlm.nih.gov/entrez/query.fcgi?db=gene&cmd=Retrieve&dopt=full_report&list_uids=26570) | solute carrier family 7 (cationic amino acid transporter, y+ system), member 11 | 38 | 91 |
| mmu-miR-143-3p | [*Necap1*](http://www.ncbi.nlm.nih.gov/entrez/query.fcgi?db=gene&cmd=Retrieve&dopt=full_report&list_uids=67602) | NECAP endocytosis associated 1 | 39 | 90 |
| mmu-miR-143-3p | [*Gigyf2*](http://www.ncbi.nlm.nih.gov/entrez/query.fcgi?db=gene&cmd=Retrieve&dopt=full_report&list_uids=227331) | GRB10 interacting GYF protein 2 | 40 | 90 |
| mmu-miR-143-3p | [*Rdh19*](http://www.ncbi.nlm.nih.gov/entrez/query.fcgi?db=gene&cmd=Retrieve&dopt=full_report&list_uids=216453) | retinol dehydrogenase 19 | 41 | 90 |
| mmu-miR-143-3p | [*Brd2*](http://www.ncbi.nlm.nih.gov/entrez/query.fcgi?db=gene&cmd=Retrieve&dopt=full_report&list_uids=14312) | bromodomain containing 2 | 42 | 90 |
| mmu-miR-143-3p | [*Ifih1*](http://www.ncbi.nlm.nih.gov/entrez/query.fcgi?db=gene&cmd=Retrieve&dopt=full_report&list_uids=71586) | interferon induced with helicase C domain 1 | 43 | 90 |
| mmu-miR-143-3p | [*Larp4*](http://www.ncbi.nlm.nih.gov/entrez/query.fcgi?db=gene&cmd=Retrieve&dopt=full_report&list_uids=207214) | La ribonucleoprotein domain family, member 4 | 44 | 90 |
| mmu-miR-143-3p | [*Arhgap28*](http://www.ncbi.nlm.nih.gov/entrez/query.fcgi?db=gene&cmd=Retrieve&dopt=full_report&list_uids=268970) | Rho GTPase activating protein 28 | 45 | 90 |
| mmu-miR-143-3p | [*Dip2b*](http://www.ncbi.nlm.nih.gov/entrez/query.fcgi?db=gene&cmd=Retrieve&dopt=full_report&list_uids=239667) | DIP2 disco-interacting protein 2 homolog B (Drosophila) | 46 | 90 |
| mmu-miR-143-3p | [*Phf11d*](http://www.ncbi.nlm.nih.gov/entrez/query.fcgi?db=gene&cmd=Retrieve&dopt=full_report&list_uids=219132) | PHD finger protein 11D | 47 | 90 |
| mmu-miR-143-3p | [*Epm2aip1*](http://www.ncbi.nlm.nih.gov/entrez/query.fcgi?db=gene&cmd=Retrieve&dopt=full_report&list_uids=77781) | EPM2A (laforin) interacting protein 1 | 48 | 89 |
| mmu-miR-143-3p | [*Nxph1*](http://www.ncbi.nlm.nih.gov/entrez/query.fcgi?db=gene&cmd=Retrieve&dopt=full_report&list_uids=18231) | neurexophilin 1 | 49 | 89 |
| mmu-miR-143-3p | [*Scamp4*](http://www.ncbi.nlm.nih.gov/entrez/query.fcgi?db=gene&cmd=Retrieve&dopt=full_report&list_uids=56214) | secretory carrier membrane protein 4 | 50 | 89 |
| mmu-miR-143-3p | [*4930524B15Rik*](http://www.ncbi.nlm.nih.gov/entrez/query.fcgi?db=gene&cmd=Retrieve&dopt=full_report&list_uids=67592) | RIKEN cDNA 4930524B15 gene | 51 | 89 |
| mmu-miR-143-3p | [*Atp8a2*](http://www.ncbi.nlm.nih.gov/entrez/query.fcgi?db=gene&cmd=Retrieve&dopt=full_report&list_uids=50769) | ATPase, aminophospholipid transporter-like, class I, type 8A, member 2 | 52 | 89 |
| mmu-miR-143-3p | [*Zfp148*](http://www.ncbi.nlm.nih.gov/entrez/query.fcgi?db=gene&cmd=Retrieve&dopt=full_report&list_uids=22661) | zinc finger protein 148 | 53 | 89 |
| mmu-miR-143-3p | [*Etv6*](http://www.ncbi.nlm.nih.gov/entrez/query.fcgi?db=gene&cmd=Retrieve&dopt=full_report&list_uids=14011) | ets variant gene 6 (TEL oncogene) | 54 | 89 |
| mmu-miR-143-3p | [*Tmem121*](http://www.ncbi.nlm.nih.gov/entrez/query.fcgi?db=gene&cmd=Retrieve&dopt=full_report&list_uids=69195) | transmembrane protein 121 | 55 | 88 |
| mmu-miR-143-3p | [*Whsc1*](http://www.ncbi.nlm.nih.gov/entrez/query.fcgi?db=gene&cmd=Retrieve&dopt=full_report&list_uids=107823) | Wolf-Hirschhorn syndrome candidate 1 (human) | 56 | 88 |
| mmu-miR-143-3p | [*C77370*](http://www.ncbi.nlm.nih.gov/entrez/query.fcgi?db=gene&cmd=Retrieve&dopt=full_report&list_uids=245555) | expressed sequence C77370 | 57 | 88 |
| mmu-miR-143-3p | [*Fam134b*](http://www.ncbi.nlm.nih.gov/entrez/query.fcgi?db=gene&cmd=Retrieve&dopt=full_report&list_uids=66270) | family with sequence similarity 134, member B | 58 | 87 |
| mmu-miR-143-3p | [*Psd3*](http://www.ncbi.nlm.nih.gov/entrez/query.fcgi?db=gene&cmd=Retrieve&dopt=full_report&list_uids=234353) | pleckstrin and Sec7 domain containing 3 | 59 | 87 |
| mmu-miR-143-3p | [*Tmem170b*](http://www.ncbi.nlm.nih.gov/entrez/query.fcgi?db=gene&cmd=Retrieve&dopt=full_report&list_uids=621976) | transmembrane protein 170B | 60 | 87 |
| mmu-miR-143-3p | [*Mar-03*](http://www.ncbi.nlm.nih.gov/entrez/query.fcgi?db=gene&cmd=Retrieve&dopt=full_report&list_uids=320253) | membrane-associated ring finger (C3HC4) 3 | 61 | 87 |
| mmu-miR-143-3p | [*Uqcrfs1*](http://www.ncbi.nlm.nih.gov/entrez/query.fcgi?db=gene&cmd=Retrieve&dopt=full_report&list_uids=66694) | ubiquinol-cytochrome c reductase, Rieske iron-sulfur polypeptide 1 | 62 | 87 |
| mmu-miR-143-3p | [*Erbb4*](http://www.ncbi.nlm.nih.gov/entrez/query.fcgi?db=gene&cmd=Retrieve&dopt=full_report&list_uids=13869) | v-erb-a erythroblastic leukemia viral oncogene homolog 4 (avian) | 63 | 87 |
| mmu-miR-143-3p | [*Bmp5*](http://www.ncbi.nlm.nih.gov/entrez/query.fcgi?db=gene&cmd=Retrieve&dopt=full_report&list_uids=12160) | bone morphogenetic protein 5 | 64 | 87 |
| mmu-miR-143-3p | [*G2e3*](http://www.ncbi.nlm.nih.gov/entrez/query.fcgi?db=gene&cmd=Retrieve&dopt=full_report&list_uids=217558) | G2/M-phase specific E3 ubiquitin ligase | 65 | 86 |
| mmu-miR-143-3p | [*Ppp3r2*](http://www.ncbi.nlm.nih.gov/entrez/query.fcgi?db=gene&cmd=Retrieve&dopt=full_report&list_uids=19059) | protein phosphatase 3, regulatory subunit B, alpha isoform (calcineurin B, type II) | 66 | 86 |
| mmu-miR-143-3p | [*Naa30*](http://www.ncbi.nlm.nih.gov/entrez/query.fcgi?db=gene&cmd=Retrieve&dopt=full_report&list_uids=70646) | N(alpha)-acetyltransferase 30, NatC catalytic subunit | 67 | 86 |
| mmu-miR-143-3p | [*Tmx4*](http://www.ncbi.nlm.nih.gov/entrez/query.fcgi?db=gene&cmd=Retrieve&dopt=full_report&list_uids=52837) | thioredoxin-related transmembrane protein 4 | 68 | 86 |
| mmu-miR-143-3p | [*Gbas*](http://www.ncbi.nlm.nih.gov/entrez/query.fcgi?db=gene&cmd=Retrieve&dopt=full_report&list_uids=14467) | glioblastoma amplified sequence | 69 | 86 |
| mmu-miR-143-3p | [*Mab21l1*](http://www.ncbi.nlm.nih.gov/entrez/query.fcgi?db=gene&cmd=Retrieve&dopt=full_report&list_uids=17116) | mab-21-like 1 (C. elegans) | 70 | 85 |
| mmu-miR-143-3p | [*Itga6*](http://www.ncbi.nlm.nih.gov/entrez/query.fcgi?db=gene&cmd=Retrieve&dopt=full_report&list_uids=16403) | integrin alpha 6 | 71 | 85 |
| mmu-miR-143-3p | [*Six4*](http://www.ncbi.nlm.nih.gov/entrez/query.fcgi?db=gene&cmd=Retrieve&dopt=full_report&list_uids=20474) | sine oculis-related homeobox 4 | 72 | 85 |
| mmu-miR-143-3p | [*Cyfip2*](http://www.ncbi.nlm.nih.gov/entrez/query.fcgi?db=gene&cmd=Retrieve&dopt=full_report&list_uids=76884) | cytoplasmic FMR1 interacting protein 2 | 73 | 85 |
| mmu-miR-143-3p | [*Patl1*](http://www.ncbi.nlm.nih.gov/entrez/query.fcgi?db=gene&cmd=Retrieve&dopt=full_report&list_uids=225929) | protein associated with topoisomerase II homolog 1 (yeast) | 74 | 85 |
| mmu-miR-143-3p | [*Mcf2*](http://www.ncbi.nlm.nih.gov/entrez/query.fcgi?db=gene&cmd=Retrieve&dopt=full_report&list_uids=109904) | mcf.2 transforming sequence | 75 | 85 |
| mmu-miR-143-3p | [*Strn3*](http://www.ncbi.nlm.nih.gov/entrez/query.fcgi?db=gene&cmd=Retrieve&dopt=full_report&list_uids=94186) | striatin, calmodulin binding protein 3 | 76 | 85 |
| mmu-miR-143-3p | [*Strip2*](http://www.ncbi.nlm.nih.gov/entrez/query.fcgi?db=gene&cmd=Retrieve&dopt=full_report&list_uids=320609) | striatin interacting protein 2 | 77 | 85 |
| mmu-miR-143-3p | [*Gfpt1*](http://www.ncbi.nlm.nih.gov/entrez/query.fcgi?db=gene&cmd=Retrieve&dopt=full_report&list_uids=14583) | glutamine fructose-6-phosphate transaminase 1 | 78 | 85 |
| mmu-miR-143-3p | [*Ube2e3*](http://www.ncbi.nlm.nih.gov/entrez/query.fcgi?db=gene&cmd=Retrieve&dopt=full_report&list_uids=22193) | ubiquitin-conjugating enzyme E2E 3 | 79 | 85 |
| mmu-miR-143-3p | [*Egln1*](http://www.ncbi.nlm.nih.gov/entrez/query.fcgi?db=gene&cmd=Retrieve&dopt=full_report&list_uids=112405) | EGL nine homolog 1 (C. elegans) | 80 | 84 |
| mmu-miR-143-3p | [*Msi2*](http://www.ncbi.nlm.nih.gov/entrez/query.fcgi?db=gene&cmd=Retrieve&dopt=full_report&list_uids=76626) | musashi RNA-binding protein 2 | 81 | 84 |
| mmu-miR-143-3p | [*Cd226*](http://www.ncbi.nlm.nih.gov/entrez/query.fcgi?db=gene&cmd=Retrieve&dopt=full_report&list_uids=225825) | CD226 antigen | 82 | 84 |
| mmu-miR-143-3p | [*Slc4a8*](http://www.ncbi.nlm.nih.gov/entrez/query.fcgi?db=gene&cmd=Retrieve&dopt=full_report&list_uids=59033) | solute carrier family 4 (anion exchanger), member 8 | 83 | 83 |
| mmu-miR-143-3p | [*Ssh2*](http://www.ncbi.nlm.nih.gov/entrez/query.fcgi?db=gene&cmd=Retrieve&dopt=full_report&list_uids=237860) | slingshot homolog 2 (Drosophila) | 84 | 83 |
| mmu-miR-143-3p | [*Fam117a*](http://www.ncbi.nlm.nih.gov/entrez/query.fcgi?db=gene&cmd=Retrieve&dopt=full_report&list_uids=215512) | family with sequence similarity 117, member A | 85 | 83 |
| mmu-miR-143-3p | [*Crebzf*](http://www.ncbi.nlm.nih.gov/entrez/query.fcgi?db=gene&cmd=Retrieve&dopt=full_report&list_uids=233490) | CREB/ATF bZIP transcription factor | 86 | 82 |
| mmu-miR-143-3p | [*Slc16a2*](http://www.ncbi.nlm.nih.gov/entrez/query.fcgi?db=gene&cmd=Retrieve&dopt=full_report&list_uids=20502) | solute carrier family 16 (monocarboxylic acid transporters), member 2 | 87 | 82 |
| mmu-miR-143-3p | [*Kcnj12*](http://www.ncbi.nlm.nih.gov/entrez/query.fcgi?db=gene&cmd=Retrieve&dopt=full_report&list_uids=16515) | potassium inwardly-rectifying channel, subfamily J, member 12 | 88 | 82 |
| mmu-miR-143-3p | [*Fgf1*](http://www.ncbi.nlm.nih.gov/entrez/query.fcgi?db=gene&cmd=Retrieve&dopt=full_report&list_uids=14164) | fibroblast growth factor 1 | 89 | 82 |
| mmu-miR-143-3p | [*Lrrc34*](http://www.ncbi.nlm.nih.gov/entrez/query.fcgi?db=gene&cmd=Retrieve&dopt=full_report&list_uids=71827) | leucine rich repeat containing 34 | 90 | 82 |
| mmu-miR-143-3p | [*Hoxa5*](http://www.ncbi.nlm.nih.gov/entrez/query.fcgi?db=gene&cmd=Retrieve&dopt=full_report&list_uids=15402) | homeobox A5 | 91 | 82 |
| mmu-miR-143-3p | [*Frem1*](http://www.ncbi.nlm.nih.gov/entrez/query.fcgi?db=gene&cmd=Retrieve&dopt=full_report&list_uids=329872) | Fras1 related extracellular matrix protein 1 | 92 | 82 |
| mmu-miR-143-3p | [*Lrrc30*](http://www.ncbi.nlm.nih.gov/entrez/query.fcgi?db=gene&cmd=Retrieve&dopt=full_report&list_uids=240131) | leucine rich repeat containing 30 | 93 | 82 |
| mmu-miR-143-3p | [*Phf6*](http://www.ncbi.nlm.nih.gov/entrez/query.fcgi?db=gene&cmd=Retrieve&dopt=full_report&list_uids=70998) | PHD finger protein 6 | 94 | 81 |
| mmu-miR-143-3p | [*Prr3*](http://www.ncbi.nlm.nih.gov/entrez/query.fcgi?db=gene&cmd=Retrieve&dopt=full_report&list_uids=75210) | proline-rich polypeptide 3 | 95 | 81 |
| mmu-miR-143-3p | [*Orc3*](http://www.ncbi.nlm.nih.gov/entrez/query.fcgi?db=gene&cmd=Retrieve&dopt=full_report&list_uids=50793) | origin recognition complex, subunit 3 | 96 | 81 |
| mmu-miR-143-3p | [*Smndc1*](http://www.ncbi.nlm.nih.gov/entrez/query.fcgi?db=gene&cmd=Retrieve&dopt=full_report&list_uids=76479) | survival motor neuron domain containing 1 | 97 | 81 |
| mmu-miR-143-3p | [*Clec7a*](http://www.ncbi.nlm.nih.gov/entrez/query.fcgi?db=gene&cmd=Retrieve&dopt=full_report&list_uids=56644) | C-type lectin domain family 7, member a | 98 | 80 |
| mmu-miR-143-3p | [*Dgkg*](http://www.ncbi.nlm.nih.gov/entrez/query.fcgi?db=gene&cmd=Retrieve&dopt=full_report&list_uids=110197) | diacylglycerol kinase, gamma | 99 | 80 |
| mmu-miR-143-3p | [*Fndc3b*](http://www.ncbi.nlm.nih.gov/entrez/query.fcgi?db=gene&cmd=Retrieve&dopt=full_report&list_uids=72007) | fibronectin type III domain containing 3B | 100 | 80 |
| mmu-miR-143-3p | [*Map9*](http://www.ncbi.nlm.nih.gov/entrez/query.fcgi?db=gene&cmd=Retrieve&dopt=full_report&list_uids=213582) | microtubule-associated protein 9 | 101 | 80 |
| mmu-miR-143-3p | [*Otud4*](http://www.ncbi.nlm.nih.gov/entrez/query.fcgi?db=gene&cmd=Retrieve&dopt=full_report&list_uids=73945) | OTU domain containing 4 | 102 | 80 |
| mmu-miR-143-3p | [*Zdhhc21*](http://www.ncbi.nlm.nih.gov/entrez/query.fcgi?db=gene&cmd=Retrieve&dopt=full_report&list_uids=68268) | zinc finger, DHHC domain containing 21 | 103 | 80 |
| mmu-miR-199b-3p | [*Celsr2*](http://www.ncbi.nlm.nih.gov/entrez/query.fcgi?db=gene&cmd=Retrieve&dopt=full_report&list_uids=53883) | cadherin, EGF LAG seven-pass G-type receptor 2 (flamingo homolog, Drosophila) | 1 | 100 |
| mmu-miR-199b-3p | [*Lin28b*](http://www.ncbi.nlm.nih.gov/entrez/query.fcgi?db=gene&cmd=Retrieve&dopt=full_report&list_uids=380669) | lin-28 homolog B (C. elegans) | 2 | 100 |
| mmu-miR-199b-3p | [*Cd151*](http://www.ncbi.nlm.nih.gov/entrez/query.fcgi?db=gene&cmd=Retrieve&dopt=full_report&list_uids=12476) | CD151 antigen | 3 | 99 |
| mmu-miR-199b-3p | [*Bcar3*](http://www.ncbi.nlm.nih.gov/entrez/query.fcgi?db=gene&cmd=Retrieve&dopt=full_report&list_uids=29815) | breast cancer anti-estrogen resistance 3 | 4 | 99 |
| mmu-miR-199b-3p | [*Klhl3*](http://www.ncbi.nlm.nih.gov/entrez/query.fcgi?db=gene&cmd=Retrieve&dopt=full_report&list_uids=100503085) | kelch-like 3 | 5 | 99 |
| mmu-miR-199b-3p | [*Adamtsl3*](http://www.ncbi.nlm.nih.gov/entrez/query.fcgi?db=gene&cmd=Retrieve&dopt=full_report&list_uids=269959) | ADAMTS-like 3 | 6 | 99 |
| mmu-miR-199b-3p | [*Ankrd44*](http://www.ncbi.nlm.nih.gov/entrez/query.fcgi?db=gene&cmd=Retrieve&dopt=full_report&list_uids=329154) | ankyrin repeat domain 44 | 7 | 99 |
| mmu-miR-199b-3p | [*Acvr2a*](http://www.ncbi.nlm.nih.gov/entrez/query.fcgi?db=gene&cmd=Retrieve&dopt=full_report&list_uids=11480) | activin receptor IIA | 8 | 99 |
| mmu-miR-199b-3p | [*Tmem62*](http://www.ncbi.nlm.nih.gov/entrez/query.fcgi?db=gene&cmd=Retrieve&dopt=full_report&list_uids=96957) | transmembrane protein 62 | 9 | 98 |
| mmu-miR-199b-3p | [*Nlk*](http://www.ncbi.nlm.nih.gov/entrez/query.fcgi?db=gene&cmd=Retrieve&dopt=full_report&list_uids=18099) | nemo like kinase | 10 | 98 |
| mmu-miR-199b-3p | [*Kdm5a*](http://www.ncbi.nlm.nih.gov/entrez/query.fcgi?db=gene&cmd=Retrieve&dopt=full_report&list_uids=214899) | lysine (K)-specific demethylase 5A | 11 | 97 |
| mmu-miR-199b-3p | [*Lrp2*](http://www.ncbi.nlm.nih.gov/entrez/query.fcgi?db=gene&cmd=Retrieve&dopt=full_report&list_uids=14725) | low density lipoprotein receptor-related protein 2 | 12 | 97 |
| mmu-miR-199b-3p | [*Psd2*](http://www.ncbi.nlm.nih.gov/entrez/query.fcgi?db=gene&cmd=Retrieve&dopt=full_report&list_uids=74002) | pleckstrin and Sec7 domain containing 2 | 13 | 97 |
| mmu-miR-199b-3p | [*G3bp2*](http://www.ncbi.nlm.nih.gov/entrez/query.fcgi?db=gene&cmd=Retrieve&dopt=full_report&list_uids=23881) | GTPase activating protein (SH3 domain) binding protein 2 | 14 | 97 |
| mmu-miR-199b-3p | [*Purg*](http://www.ncbi.nlm.nih.gov/entrez/query.fcgi?db=gene&cmd=Retrieve&dopt=full_report&list_uids=75029) | purine-rich element binding protein G | 15 | 97 |
| mmu-miR-199b-3p | [*Nova1*](http://www.ncbi.nlm.nih.gov/entrez/query.fcgi?db=gene&cmd=Retrieve&dopt=full_report&list_uids=664883) | neuro-oncological ventral antigen 1 | 16 | 97 |
| mmu-miR-199b-3p | [*Serpine2*](http://www.ncbi.nlm.nih.gov/entrez/query.fcgi?db=gene&cmd=Retrieve&dopt=full_report&list_uids=20720) | serine (or cysteine) peptidase inhibitor, clade E, member 2 | 17 | 96 |
| mmu-miR-199b-3p | [*Itga3*](http://www.ncbi.nlm.nih.gov/entrez/query.fcgi?db=gene&cmd=Retrieve&dopt=full_report&list_uids=16400) | integrin alpha 3 | 18 | 96 |
| mmu-miR-199b-3p | [*Kdm6a*](http://www.ncbi.nlm.nih.gov/entrez/query.fcgi?db=gene&cmd=Retrieve&dopt=full_report&list_uids=22289) | lysine (K)-specific demethylase 6A | 19 | 96 |
| mmu-miR-199b-3p | [*Fam199x*](http://www.ncbi.nlm.nih.gov/entrez/query.fcgi?db=gene&cmd=Retrieve&dopt=full_report&list_uids=245622) | family with sequence similarity 199, X-linked | 20 | 96 |
| mmu-miR-199b-3p | [*Ppp2r2a*](http://www.ncbi.nlm.nih.gov/entrez/query.fcgi?db=gene&cmd=Retrieve&dopt=full_report&list_uids=71978) | protein phosphatase 2, regulatory subunit B, alpha | 21 | 96 |
| mmu-miR-199b-3p | [*Tppp*](http://www.ncbi.nlm.nih.gov/entrez/query.fcgi?db=gene&cmd=Retrieve&dopt=full_report&list_uids=72948) | tubulin polymerization promoting protein | 22 | 95 |
| mmu-miR-199b-3p | [*Pak4*](http://www.ncbi.nlm.nih.gov/entrez/query.fcgi?db=gene&cmd=Retrieve&dopt=full_report&list_uids=70584) | p21 protein (Cdc42/Rac)-activated kinase 4 | 23 | 95 |
| mmu-miR-199b-3p | [*Nid2*](http://www.ncbi.nlm.nih.gov/entrez/query.fcgi?db=gene&cmd=Retrieve&dopt=full_report&list_uids=18074) | nidogen 2 | 24 | 95 |
| mmu-miR-199b-3p | [*Arhgap20*](http://www.ncbi.nlm.nih.gov/entrez/query.fcgi?db=gene&cmd=Retrieve&dopt=full_report&list_uids=244867) | Rho GTPase activating protein 20 | 25 | 95 |
| mmu-miR-199b-3p | [*Rimklb*](http://www.ncbi.nlm.nih.gov/entrez/query.fcgi?db=gene&cmd=Retrieve&dopt=full_report&list_uids=108653) | ribosomal modification protein rimK-like family member B | 26 | 95 |
| mmu-miR-199b-3p | [*Emc1*](http://www.ncbi.nlm.nih.gov/entrez/query.fcgi?db=gene&cmd=Retrieve&dopt=full_report&list_uids=230866) | ER membrane protein complex subunit 1 | 27 | 95 |
| mmu-miR-199b-3p | [*Pdgfra*](http://www.ncbi.nlm.nih.gov/entrez/query.fcgi?db=gene&cmd=Retrieve&dopt=full_report&list_uids=18595) | platelet derived growth factor receptor, alpha polypeptide | 28 | 95 |
| mmu-miR-199b-3p | [*Pnrc1*](http://www.ncbi.nlm.nih.gov/entrez/query.fcgi?db=gene&cmd=Retrieve&dopt=full_report&list_uids=108767) | proline-rich nuclear receptor coactivator 1 | 29 | 95 |
| mmu-miR-199b-3p | [*Cd2ap*](http://www.ncbi.nlm.nih.gov/entrez/query.fcgi?db=gene&cmd=Retrieve&dopt=full_report&list_uids=12488) | CD2-associated protein | 30 | 95 |
| mmu-miR-199b-3p | [*Zhx1*](http://www.ncbi.nlm.nih.gov/entrez/query.fcgi?db=gene&cmd=Retrieve&dopt=full_report&list_uids=22770) | zinc fingers and homeoboxes 1 | 31 | 95 |
| mmu-miR-199b-3p | [*Etnk1*](http://www.ncbi.nlm.nih.gov/entrez/query.fcgi?db=gene&cmd=Retrieve&dopt=full_report&list_uids=75320) | ethanolamine kinase 1 | 32 | 94 |
| mmu-miR-199b-3p | [*AU040320*](http://www.ncbi.nlm.nih.gov/entrez/query.fcgi?db=gene&cmd=Retrieve&dopt=full_report&list_uids=100317) | expressed sequence AU040320 | 33 | 94 |
| mmu-miR-199b-3p | [*Itgb8*](http://www.ncbi.nlm.nih.gov/entrez/query.fcgi?db=gene&cmd=Retrieve&dopt=full_report&list_uids=320910) | integrin beta 8 | 34 | 94 |
| mmu-miR-199b-3p | [*Fxr1*](http://www.ncbi.nlm.nih.gov/entrez/query.fcgi?db=gene&cmd=Retrieve&dopt=full_report&list_uids=14359) | fragile X mental retardation gene 1, autosomal homolog | 35 | 94 |
| mmu-miR-199b-3p | [*Fubp1*](http://www.ncbi.nlm.nih.gov/entrez/query.fcgi?db=gene&cmd=Retrieve&dopt=full_report&list_uids=51886) | far upstream element (FUSE) binding protein 1 | 36 | 93 |
| mmu-miR-199b-3p | [*Fn1*](http://www.ncbi.nlm.nih.gov/entrez/query.fcgi?db=gene&cmd=Retrieve&dopt=full_report&list_uids=14268) | fibronectin 1 | 37 | 93 |
| mmu-miR-199b-3p | [*Map3k4*](http://www.ncbi.nlm.nih.gov/entrez/query.fcgi?db=gene&cmd=Retrieve&dopt=full_report&list_uids=26407) | mitogen-activated protein kinase kinase kinase 4 | 38 | 92 |
| mmu-miR-199b-3p | [*Ptpn4*](http://www.ncbi.nlm.nih.gov/entrez/query.fcgi?db=gene&cmd=Retrieve&dopt=full_report&list_uids=19258) | protein tyrosine phosphatase, non-receptor type 4 | 39 | 92 |
| mmu-miR-199b-3p | [*Vgll2*](http://www.ncbi.nlm.nih.gov/entrez/query.fcgi?db=gene&cmd=Retrieve&dopt=full_report&list_uids=215031) | vestigial like 2 homolog (Drosophila) | 40 | 91 |
| mmu-miR-199b-3p | [*Ppp1r9a*](http://www.ncbi.nlm.nih.gov/entrez/query.fcgi?db=gene&cmd=Retrieve&dopt=full_report&list_uids=243725) | protein phosphatase 1, regulatory (inhibitor) subunit 9A | 41 | 91 |
| mmu-miR-199b-3p | [*Zbtb18*](http://www.ncbi.nlm.nih.gov/entrez/query.fcgi?db=gene&cmd=Retrieve&dopt=full_report&list_uids=30928) | zinc finger and BTB domain containing 18 | 42 | 91 |
| mmu-miR-199b-3p | [*Aplp2*](http://www.ncbi.nlm.nih.gov/entrez/query.fcgi?db=gene&cmd=Retrieve&dopt=full_report&list_uids=11804) | amyloid beta (A4) precursor-like protein 2 | 43 | 91 |
| mmu-miR-199b-3p | [*Ppp2r5e*](http://www.ncbi.nlm.nih.gov/entrez/query.fcgi?db=gene&cmd=Retrieve&dopt=full_report&list_uids=26932) | protein phosphatase 2, regulatory subunit B', epsilon | 44 | 90 |
| mmu-miR-199b-3p | [*Cnih2*](http://www.ncbi.nlm.nih.gov/entrez/query.fcgi?db=gene&cmd=Retrieve&dopt=full_report&list_uids=12794) | cornichon homolog 2 (Drosophila) | 45 | 90 |
| mmu-miR-199b-3p | [*Tmtc3*](http://www.ncbi.nlm.nih.gov/entrez/query.fcgi?db=gene&cmd=Retrieve&dopt=full_report&list_uids=237500) | transmembrane and tetratricopeptide repeat containing 3 | 46 | 89 |
| mmu-miR-199b-3p | [*Pip5k1b*](http://www.ncbi.nlm.nih.gov/entrez/query.fcgi?db=gene&cmd=Retrieve&dopt=full_report&list_uids=18719) | phosphatidylinositol-4-phosphate 5-kinase, type 1 beta | 47 | 89 |
| mmu-miR-199b-3p | [*Nufip2*](http://www.ncbi.nlm.nih.gov/entrez/query.fcgi?db=gene&cmd=Retrieve&dopt=full_report&list_uids=68564) | nuclear fragile X mental retardation protein interacting protein 2 | 48 | 89 |
| mmu-miR-199b-3p | [*Cdk17*](http://www.ncbi.nlm.nih.gov/entrez/query.fcgi?db=gene&cmd=Retrieve&dopt=full_report&list_uids=237459) | cyclin-dependent kinase 17 | 49 | 88 |
| mmu-miR-199b-3p | [*Fktn*](http://www.ncbi.nlm.nih.gov/entrez/query.fcgi?db=gene&cmd=Retrieve&dopt=full_report&list_uids=246179) | fukutin | 50 | 88 |
| mmu-miR-199b-3p | [*D030056L22Rik*](http://www.ncbi.nlm.nih.gov/entrez/query.fcgi?db=gene&cmd=Retrieve&dopt=full_report&list_uids=225995) | RIKEN cDNA D030056L22 gene | 51 | 88 |
| mmu-miR-199b-3p | [*Zbtb33*](http://www.ncbi.nlm.nih.gov/entrez/query.fcgi?db=gene&cmd=Retrieve&dopt=full_report&list_uids=56805) | zinc finger and BTB domain containing 33 | 52 | 88 |
| mmu-miR-199b-3p | [*Cxxc5*](http://www.ncbi.nlm.nih.gov/entrez/query.fcgi?db=gene&cmd=Retrieve&dopt=full_report&list_uids=67393) | CXXC finger 5 | 53 | 87 |
| mmu-miR-199b-3p | [*Erbb4*](http://www.ncbi.nlm.nih.gov/entrez/query.fcgi?db=gene&cmd=Retrieve&dopt=full_report&list_uids=13869) | v-erb-a erythroblastic leukemia viral oncogene homolog 4 (avian) | 54 | 87 |
| mmu-miR-199b-3p | [*Vldlr*](http://www.ncbi.nlm.nih.gov/entrez/query.fcgi?db=gene&cmd=Retrieve&dopt=full_report&list_uids=22359) | very low density lipoprotein receptor | 55 | 86 |
| mmu-miR-199b-3p | [*Fam60a*](http://www.ncbi.nlm.nih.gov/entrez/query.fcgi?db=gene&cmd=Retrieve&dopt=full_report&list_uids=56306) | family with sequence similarity 60, member A | 56 | 86 |
| mmu-miR-199b-3p | [*Rps6ka6*](http://www.ncbi.nlm.nih.gov/entrez/query.fcgi?db=gene&cmd=Retrieve&dopt=full_report&list_uids=67071) | ribosomal protein S6 kinase polypeptide 6 | 57 | 86 |
| mmu-miR-199b-3p | [*Ccdc85c*](http://www.ncbi.nlm.nih.gov/entrez/query.fcgi?db=gene&cmd=Retrieve&dopt=full_report&list_uids=668158) | coiled-coil domain containing 85C | 58 | 86 |
| mmu-miR-199b-3p | [*Sema3a*](http://www.ncbi.nlm.nih.gov/entrez/query.fcgi?db=gene&cmd=Retrieve&dopt=full_report&list_uids=20346) | sema domain, immunoglobulin domain (Ig), short basic domain, secreted, (semaphorin) 3A | 59 | 86 |
| mmu-miR-199b-3p | [*Pde4b*](http://www.ncbi.nlm.nih.gov/entrez/query.fcgi?db=gene&cmd=Retrieve&dopt=full_report&list_uids=18578) | phosphodiesterase 4B, cAMP specific | 60 | 86 |
| mmu-miR-199b-3p | [*Mal2*](http://www.ncbi.nlm.nih.gov/entrez/query.fcgi?db=gene&cmd=Retrieve&dopt=full_report&list_uids=105853) | mal, T cell differentiation protein 2 | 61 | 86 |
| mmu-miR-199b-3p | [*Cep97*](http://www.ncbi.nlm.nih.gov/entrez/query.fcgi?db=gene&cmd=Retrieve&dopt=full_report&list_uids=74201) | centrosomal protein 97 | 62 | 85 |
| mmu-miR-199b-3p | [*Sp1*](http://www.ncbi.nlm.nih.gov/entrez/query.fcgi?db=gene&cmd=Retrieve&dopt=full_report&list_uids=20683) | trans-acting transcription factor 1 | 63 | 85 |
| mmu-miR-199b-3p | [*Adam10*](http://www.ncbi.nlm.nih.gov/entrez/query.fcgi?db=gene&cmd=Retrieve&dopt=full_report&list_uids=11487) | a disintegrin and metallopeptidase domain 10 | 64 | 85 |
| mmu-miR-199b-3p | [*Taok1*](http://www.ncbi.nlm.nih.gov/entrez/query.fcgi?db=gene&cmd=Retrieve&dopt=full_report&list_uids=216965) | TAO kinase 1 | 65 | 85 |
| mmu-miR-199b-3p | [*Raph1*](http://www.ncbi.nlm.nih.gov/entrez/query.fcgi?db=gene&cmd=Retrieve&dopt=full_report&list_uids=77300) | Ras association (RalGDS/AF-6) and pleckstrin homology domains 1 | 66 | 85 |
| mmu-miR-199b-3p | [*Rbm47*](http://www.ncbi.nlm.nih.gov/entrez/query.fcgi?db=gene&cmd=Retrieve&dopt=full_report&list_uids=245945) | RNA binding motif protein 47 | 67 | 85 |
| mmu-miR-199b-3p | [*Arhgef3*](http://www.ncbi.nlm.nih.gov/entrez/query.fcgi?db=gene&cmd=Retrieve&dopt=full_report&list_uids=71704) | Rho guanine nucleotide exchange factor (GEF) 3 | 68 | 85 |
| mmu-miR-199b-3p | [*Plcb1*](http://www.ncbi.nlm.nih.gov/entrez/query.fcgi?db=gene&cmd=Retrieve&dopt=full_report&list_uids=18795) | phospholipase C, beta 1 | 69 | 84 |
| mmu-miR-199b-3p | [*Wapal*](http://www.ncbi.nlm.nih.gov/entrez/query.fcgi?db=gene&cmd=Retrieve&dopt=full_report&list_uids=218914) | wings apart-like homolog (Drosophila) | 70 | 84 |
| mmu-miR-199b-3p | [*Slc39a10*](http://www.ncbi.nlm.nih.gov/entrez/query.fcgi?db=gene&cmd=Retrieve&dopt=full_report&list_uids=227059) | solute carrier family 39 (zinc transporter), member 10 | 71 | 84 |
| mmu-miR-199b-3p | [*Rfx3*](http://www.ncbi.nlm.nih.gov/entrez/query.fcgi?db=gene&cmd=Retrieve&dopt=full_report&list_uids=19726) | regulatory factor X, 3 (influences HLA class II expression) | 72 | 84 |
| mmu-miR-199b-3p | [*Agbl3*](http://www.ncbi.nlm.nih.gov/entrez/query.fcgi?db=gene&cmd=Retrieve&dopt=full_report&list_uids=76223) | ATP/GTP binding protein-like 3 | 73 | 84 |
| mmu-miR-199b-3p | [*Mdga2*](http://www.ncbi.nlm.nih.gov/entrez/query.fcgi?db=gene&cmd=Retrieve&dopt=full_report&list_uids=320772) | MAM domain containing glycosylphosphatidylinositol anchor 2 | 74 | 84 |
| mmu-miR-199b-3p | [*Iffo2*](http://www.ncbi.nlm.nih.gov/entrez/query.fcgi?db=gene&cmd=Retrieve&dopt=full_report&list_uids=212632) | intermediate filament family orphan 2 | 75 | 84 |
| mmu-miR-199b-3p | [*Zbtb20*](http://www.ncbi.nlm.nih.gov/entrez/query.fcgi?db=gene&cmd=Retrieve&dopt=full_report&list_uids=56490) | zinc finger and BTB domain containing 20 | 76 | 84 |
| mmu-miR-199b-3p | [*Ube2w*](http://www.ncbi.nlm.nih.gov/entrez/query.fcgi?db=gene&cmd=Retrieve&dopt=full_report&list_uids=66799) | ubiquitin-conjugating enzyme E2W (putative) | 77 | 83 |
| mmu-miR-199b-3p | [*Tenm1*](http://www.ncbi.nlm.nih.gov/entrez/query.fcgi?db=gene&cmd=Retrieve&dopt=full_report&list_uids=23963) | teneurin transmembrane protein 1 | 78 | 83 |
| mmu-miR-199b-3p | [*Gm12888*](http://www.ncbi.nlm.nih.gov/entrez/query.fcgi?db=gene&cmd=Retrieve&dopt=full_report&list_uids=545677) | predicted gene 12888 | 79 | 83 |
| mmu-miR-199b-3p | [*Cnot7*](http://www.ncbi.nlm.nih.gov/entrez/query.fcgi?db=gene&cmd=Retrieve&dopt=full_report&list_uids=18983) | CCR4-NOT transcription complex, subunit 7 | 80 | 83 |
| mmu-miR-199b-3p | [*Ktn1*](http://www.ncbi.nlm.nih.gov/entrez/query.fcgi?db=gene&cmd=Retrieve&dopt=full_report&list_uids=16709) | kinectin 1 | 81 | 82 |
| mmu-miR-199b-3p | [*Cpeb4*](http://www.ncbi.nlm.nih.gov/entrez/query.fcgi?db=gene&cmd=Retrieve&dopt=full_report&list_uids=67579) | cytoplasmic polyadenylation element binding protein 4 | 82 | 82 |
| mmu-miR-199b-3p | [*Arl15*](http://www.ncbi.nlm.nih.gov/entrez/query.fcgi?db=gene&cmd=Retrieve&dopt=full_report&list_uids=218639) | ADP-ribosylation factor-like 15 | 83 | 82 |
| mmu-miR-199b-3p | [*Btbd9*](http://www.ncbi.nlm.nih.gov/entrez/query.fcgi?db=gene&cmd=Retrieve&dopt=full_report&list_uids=224671) | BTB (POZ) domain containing 9 | 84 | 82 |
| mmu-miR-199b-3p | [*Pik3cb*](http://www.ncbi.nlm.nih.gov/entrez/query.fcgi?db=gene&cmd=Retrieve&dopt=full_report&list_uids=74769) | phosphatidylinositol 3-kinase, catalytic, beta polypeptide | 85 | 82 |
| mmu-miR-199b-3p | [*Cbll1*](http://www.ncbi.nlm.nih.gov/entrez/query.fcgi?db=gene&cmd=Retrieve&dopt=full_report&list_uids=104836) | Casitas B-lineage lymphoma-like 1 | 86 | 81 |
| mmu-miR-199b-3p | [*Id4*](http://www.ncbi.nlm.nih.gov/entrez/query.fcgi?db=gene&cmd=Retrieve&dopt=full_report&list_uids=15904) | inhibitor of DNA binding 4 | 87 | 81 |
| mmu-miR-199b-3p | [*Mmgt1*](http://www.ncbi.nlm.nih.gov/entrez/query.fcgi?db=gene&cmd=Retrieve&dopt=full_report&list_uids=236792) | membrane magnesium transporter 1 | 88 | 81 |
| mmu-miR-199b-3p | [*Kcmf1*](http://www.ncbi.nlm.nih.gov/entrez/query.fcgi?db=gene&cmd=Retrieve&dopt=full_report&list_uids=74287) | potassium channel modulatory factor 1 | 89 | 81 |
| mmu-miR-199b-3p | [*Pon2*](http://www.ncbi.nlm.nih.gov/entrez/query.fcgi?db=gene&cmd=Retrieve&dopt=full_report&list_uids=330260) | paraoxonase 2 | 90 | 81 |
| mmu-miR-199b-3p | [*Galnt7*](http://www.ncbi.nlm.nih.gov/entrez/query.fcgi?db=gene&cmd=Retrieve&dopt=full_report&list_uids=108150) | UDP-N-acetyl-alpha-D-galactosamine: polypeptide N-acetylgalactosaminyltransferase 7 | 91 | 80 |
| mmu-miR-199b-3p | [*Ggnbp2*](http://www.ncbi.nlm.nih.gov/entrez/query.fcgi?db=gene&cmd=Retrieve&dopt=full_report&list_uids=217039) | gametogenetin binding protein 2 | 92 | 80 |
| mmu-miR-199b-3p | [*Atad1*](http://www.ncbi.nlm.nih.gov/entrez/query.fcgi?db=gene&cmd=Retrieve&dopt=full_report&list_uids=67979) | ATPase family, AAA domain containing 1 | 93 | 80 |
| mmu-miR-199b-3p | [*Ndrg1*](http://www.ncbi.nlm.nih.gov/entrez/query.fcgi?db=gene&cmd=Retrieve&dopt=full_report&list_uids=17988) | N-myc downstream regulated gene 1 | 94 | 80 |
| mmu-miR-199b-3p | [*Itpk1*](http://www.ncbi.nlm.nih.gov/entrez/query.fcgi?db=gene&cmd=Retrieve&dopt=full_report&list_uids=217837) | inositol 1,3,4-triphosphate 5/6 kinase | 95 | 80 |
| mmu-miR-27b-5p | [*Kank1*](http://www.ncbi.nlm.nih.gov/entrez/query.fcgi?db=gene&cmd=Retrieve&dopt=full_report&list_uids=107351) | KN motif and ankyrin repeat domains 1 | 1 | 97 |
| mmu-miR-27b-5p | [*Pitpna*](http://www.ncbi.nlm.nih.gov/entrez/query.fcgi?db=gene&cmd=Retrieve&dopt=full_report&list_uids=18738) | phosphatidylinositol transfer protein, alpha | 2 | 96 |
| mmu-miR-27b-5p | [*Zfp36l1*](http://www.ncbi.nlm.nih.gov/entrez/query.fcgi?db=gene&cmd=Retrieve&dopt=full_report&list_uids=12192) | zinc finger protein 36, C3H type-like 1 | 3 | 95 |
| mmu-miR-27b-5p | [*Trps1*](http://www.ncbi.nlm.nih.gov/entrez/query.fcgi?db=gene&cmd=Retrieve&dopt=full_report&list_uids=83925) | trichorhinophalangeal syndrome I (human) | 4 | 95 |
| mmu-miR-27b-5p | [*Gmcl1*](http://www.ncbi.nlm.nih.gov/entrez/query.fcgi?db=gene&cmd=Retrieve&dopt=full_report&list_uids=23885) | germ cell-less homolog 1 (Drosophila) | 5 | 94 |
| mmu-miR-27b-5p | [*Bicd1*](http://www.ncbi.nlm.nih.gov/entrez/query.fcgi?db=gene&cmd=Retrieve&dopt=full_report&list_uids=12121) | bicaudal D homolog 1 (Drosophila) | 6 | 94 |
| mmu-miR-27b-5p | [*Clint1*](http://www.ncbi.nlm.nih.gov/entrez/query.fcgi?db=gene&cmd=Retrieve&dopt=full_report&list_uids=216705) | clathrin interactor 1 | 7 | 93 |
| mmu-miR-27b-5p | [*Kcnj1*](http://www.ncbi.nlm.nih.gov/entrez/query.fcgi?db=gene&cmd=Retrieve&dopt=full_report&list_uids=56379) | potassium inwardly-rectifying channel, subfamily J, member 1 | 8 | 93 |
| mmu-miR-27b-5p | [*Hnrnph2*](http://www.ncbi.nlm.nih.gov/entrez/query.fcgi?db=gene&cmd=Retrieve&dopt=full_report&list_uids=56258) | heterogeneous nuclear ribonucleoprotein H2 | 9 | 93 |
| mmu-miR-27b-5p | [*Rbm39*](http://www.ncbi.nlm.nih.gov/entrez/query.fcgi?db=gene&cmd=Retrieve&dopt=full_report&list_uids=170791) | RNA binding motif protein 39 | 10 | 92 |
| mmu-miR-27b-5p | [*Cd160*](http://www.ncbi.nlm.nih.gov/entrez/query.fcgi?db=gene&cmd=Retrieve&dopt=full_report&list_uids=54215) | CD160 antigen | 11 | 91 |
| mmu-miR-27b-5p | [*Gda*](http://www.ncbi.nlm.nih.gov/entrez/query.fcgi?db=gene&cmd=Retrieve&dopt=full_report&list_uids=14544) | guanine deaminase | 12 | 91 |
| mmu-miR-27b-5p | [*Nov*](http://www.ncbi.nlm.nih.gov/entrez/query.fcgi?db=gene&cmd=Retrieve&dopt=full_report&list_uids=18133) | nephroblastoma overexpressed gene | 13 | 91 |
| mmu-miR-27b-5p | [*Ets1*](http://www.ncbi.nlm.nih.gov/entrez/query.fcgi?db=gene&cmd=Retrieve&dopt=full_report&list_uids=23871) | E26 avian leukemia oncogene 1, 5' domain | 14 | 91 |
| mmu-miR-27b-5p | [*Dsg1a*](http://www.ncbi.nlm.nih.gov/entrez/query.fcgi?db=gene&cmd=Retrieve&dopt=full_report&list_uids=13510) | desmoglein 1 alpha | 15 | 90 |
| mmu-miR-27b-5p | [*Stc1*](http://www.ncbi.nlm.nih.gov/entrez/query.fcgi?db=gene&cmd=Retrieve&dopt=full_report&list_uids=20855) | stanniocalcin 1 | 16 | 90 |
| mmu-miR-27b-5p | [*Dscaml1*](http://www.ncbi.nlm.nih.gov/entrez/query.fcgi?db=gene&cmd=Retrieve&dopt=full_report&list_uids=114873) | Down syndrome cell adhesion molecule like 1 | 17 | 89 |
| mmu-miR-27b-5p | [*Krtap3-1*](http://www.ncbi.nlm.nih.gov/entrez/query.fcgi?db=gene&cmd=Retrieve&dopt=full_report&list_uids=69473) | keratin associated protein 3-1 | 18 | 87 |
| mmu-miR-27b-5p | [*Stx3*](http://www.ncbi.nlm.nih.gov/entrez/query.fcgi?db=gene&cmd=Retrieve&dopt=full_report&list_uids=20908) | syntaxin 3 | 19 | 86 |
| mmu-miR-27b-5p | [*Prkra*](http://www.ncbi.nlm.nih.gov/entrez/query.fcgi?db=gene&cmd=Retrieve&dopt=full_report&list_uids=23992) | protein kinase, interferon inducible double stranded RNA dependent activator | 20 | 85 |
| mmu-miR-27b-5p | [*Med13*](http://www.ncbi.nlm.nih.gov/entrez/query.fcgi?db=gene&cmd=Retrieve&dopt=full_report&list_uids=327987) | mediator complex subunit 13 | 21 | 85 |
| mmu-miR-27b-5p | [*Ubtf*](http://www.ncbi.nlm.nih.gov/entrez/query.fcgi?db=gene&cmd=Retrieve&dopt=full_report&list_uids=21429) | upstream binding transcription factor, RNA polymerase I | 22 | 85 |
| mmu-miR-27b-5p | [*Zeb2*](http://www.ncbi.nlm.nih.gov/entrez/query.fcgi?db=gene&cmd=Retrieve&dopt=full_report&list_uids=24136) | zinc finger E-box binding homeobox 2 | 23 | 85 |
| mmu-miR-27b-5p | [*Zhx1*](http://www.ncbi.nlm.nih.gov/entrez/query.fcgi?db=gene&cmd=Retrieve&dopt=full_report&list_uids=22770) | zinc fingers and homeoboxes 1 | 24 | 84 |
| mmu-miR-27b-5p | [*Golga5*](http://www.ncbi.nlm.nih.gov/entrez/query.fcgi?db=gene&cmd=Retrieve&dopt=full_report&list_uids=27277) | golgi autoantigen, golgin subfamily a, 5 | 25 | 83 |
| mmu-miR-27b-5p | [*Map3k3*](http://www.ncbi.nlm.nih.gov/entrez/query.fcgi?db=gene&cmd=Retrieve&dopt=full_report&list_uids=26406) | mitogen-activated protein kinase kinase kinase 3 | 26 | 83 |
| mmu-miR-27b-5p | [*Dcaf7*](http://www.ncbi.nlm.nih.gov/entrez/query.fcgi?db=gene&cmd=Retrieve&dopt=full_report&list_uids=71833) | DDB1 and CUL4 associated factor 7 | 27 | 83 |
| mmu-miR-27b-5p | [*Srsf6*](http://www.ncbi.nlm.nih.gov/entrez/query.fcgi?db=gene&cmd=Retrieve&dopt=full_report&list_uids=67996) | serine/arginine-rich splicing factor 6 | 28 | 82 |
| mmu-miR-27b-5p | [*B630005N14Rik*](http://www.ncbi.nlm.nih.gov/entrez/query.fcgi?db=gene&cmd=Retrieve&dopt=full_report&list_uids=101148) | RIKEN cDNA B630005N14 gene | 29 | 80 |
| mmu-miR-27b-5p | [*Atp2b4*](http://www.ncbi.nlm.nih.gov/entrez/query.fcgi?db=gene&cmd=Retrieve&dopt=full_report&list_uids=381290) | ATPase, Ca++ transporting, plasma membrane 4 | 30 | 80 |
| mmu-miR-27b-5p | [*Btbd3*](http://www.ncbi.nlm.nih.gov/entrez/query.fcgi?db=gene&cmd=Retrieve&dopt=full_report&list_uids=228662) | BTB (POZ) domain containing 3 | 31 | 80 |
| mmu-miR-322-5p | [*Luzp1*](http://www.ncbi.nlm.nih.gov/entrez/query.fcgi?db=gene&cmd=Retrieve&dopt=full_report&list_uids=269593) | leucine zipper protein 1 | 1 | 100 |
| mmu-miR-322-5p | [*Pappa*](http://www.ncbi.nlm.nih.gov/entrez/query.fcgi?db=gene&cmd=Retrieve&dopt=full_report&list_uids=18491) | pregnancy-associated plasma protein A | 2 | 100 |
| mmu-miR-322-5p | [*Pafah1b1*](http://www.ncbi.nlm.nih.gov/entrez/query.fcgi?db=gene&cmd=Retrieve&dopt=full_report&list_uids=18472) | platelet-activating factor acetylhydrolase, isoform 1b, subunit 1 | 3 | 100 |
| mmu-miR-322-5p | [*Gpatch8*](http://www.ncbi.nlm.nih.gov/entrez/query.fcgi?db=gene&cmd=Retrieve&dopt=full_report&list_uids=237943) | G patch domain containing 8 | 4 | 100 |
| mmu-miR-322-5p | [*Kif5c*](http://www.ncbi.nlm.nih.gov/entrez/query.fcgi?db=gene&cmd=Retrieve&dopt=full_report&list_uids=16574) | kinesin family member 5C | 5 | 100 |
| mmu-miR-322-5p | [*Nup50*](http://www.ncbi.nlm.nih.gov/entrez/query.fcgi?db=gene&cmd=Retrieve&dopt=full_report&list_uids=18141) | nucleoporin 50 | 6 | 100 |
| mmu-miR-322-5p | [*N4bp1*](http://www.ncbi.nlm.nih.gov/entrez/query.fcgi?db=gene&cmd=Retrieve&dopt=full_report&list_uids=80750) | NEDD4 binding protein 1 | 7 | 100 |
| mmu-miR-322-5p | [*Abtb2*](http://www.ncbi.nlm.nih.gov/entrez/query.fcgi?db=gene&cmd=Retrieve&dopt=full_report&list_uids=99382) | ankyrin repeat and BTB (POZ) domain containing 2 | 8 | 100 |
| mmu-miR-322-5p | [*Akt3*](http://www.ncbi.nlm.nih.gov/entrez/query.fcgi?db=gene&cmd=Retrieve&dopt=full_report&list_uids=23797) | thymoma viral proto-oncogene 3 | 9 | 100 |
| mmu-miR-322-5p | [*Zfhx4*](http://www.ncbi.nlm.nih.gov/entrez/query.fcgi?db=gene&cmd=Retrieve&dopt=full_report&list_uids=80892) | zinc finger homeodomain 4 | 10 | 100 |
| mmu-miR-322-5p | [*Lcor*](http://www.ncbi.nlm.nih.gov/entrez/query.fcgi?db=gene&cmd=Retrieve&dopt=full_report&list_uids=212391) | ligand dependent nuclear receptor corepressor | 11 | 100 |
| mmu-miR-322-5p | [*Ubfd1*](http://www.ncbi.nlm.nih.gov/entrez/query.fcgi?db=gene&cmd=Retrieve&dopt=full_report&list_uids=28018) | ubiquitin family domain containing 1 | 12 | 99 |
| mmu-miR-322-5p | [*Hgf*](http://www.ncbi.nlm.nih.gov/entrez/query.fcgi?db=gene&cmd=Retrieve&dopt=full_report&list_uids=15234) | hepatocyte growth factor | 13 | 99 |
| mmu-miR-322-5p | [*Phf19*](http://www.ncbi.nlm.nih.gov/entrez/query.fcgi?db=gene&cmd=Retrieve&dopt=full_report&list_uids=74016) | PHD finger protein 19 | 14 | 99 |
| mmu-miR-322-5p | [*Btrc*](http://www.ncbi.nlm.nih.gov/entrez/query.fcgi?db=gene&cmd=Retrieve&dopt=full_report&list_uids=12234) | beta-transducin repeat containing protein | 15 | 99 |
| mmu-miR-322-5p | [*Cacul1*](http://www.ncbi.nlm.nih.gov/entrez/query.fcgi?db=gene&cmd=Retrieve&dopt=full_report&list_uids=78832) | CDK2 associated, cullin domain 1 | 16 | 99 |
| mmu-miR-322-5p | [*Srpr*](http://www.ncbi.nlm.nih.gov/entrez/query.fcgi?db=gene&cmd=Retrieve&dopt=full_report&list_uids=67398) | signal recognition particle receptor ('docking protein') | 17 | 99 |
| mmu-miR-322-5p | [*Cdk17*](http://www.ncbi.nlm.nih.gov/entrez/query.fcgi?db=gene&cmd=Retrieve&dopt=full_report&list_uids=237459) | cyclin-dependent kinase 17 | 18 | 99 |
| mmu-miR-322-5p | [*Cdca4*](http://www.ncbi.nlm.nih.gov/entrez/query.fcgi?db=gene&cmd=Retrieve&dopt=full_report&list_uids=71963) | cell division cycle associated 4 | 19 | 99 |
| mmu-miR-322-5p | [*Zc2hc1a*](http://www.ncbi.nlm.nih.gov/entrez/query.fcgi?db=gene&cmd=Retrieve&dopt=full_report&list_uids=67306) | zinc finger, C2HC-type containing 1A | 20 | 99 |
| mmu-miR-322-5p | [*Atf6*](http://www.ncbi.nlm.nih.gov/entrez/query.fcgi?db=gene&cmd=Retrieve&dopt=full_report&list_uids=226641) | activating transcription factor 6 | 21 | 99 |
| mmu-miR-322-5p | [*Myt1l*](http://www.ncbi.nlm.nih.gov/entrez/query.fcgi?db=gene&cmd=Retrieve&dopt=full_report&list_uids=17933) | myelin transcription factor 1-like | 22 | 99 |
| mmu-miR-322-5p | [*Serbp1*](http://www.ncbi.nlm.nih.gov/entrez/query.fcgi?db=gene&cmd=Retrieve&dopt=full_report&list_uids=66870) | serpine1 mRNA binding protein 1 | 23 | 99 |
| mmu-miR-322-5p | [*Ist1*](http://www.ncbi.nlm.nih.gov/entrez/query.fcgi?db=gene&cmd=Retrieve&dopt=full_report&list_uids=71955) | increased sodium tolerance 1 homolog (yeast) | 24 | 99 |
| mmu-miR-322-5p | [*Cpeb2*](http://www.ncbi.nlm.nih.gov/entrez/query.fcgi?db=gene&cmd=Retrieve&dopt=full_report&list_uids=231207) | cytoplasmic polyadenylation element binding protein 2 | 25 | 99 |
| mmu-miR-322-5p | [*Prkar2a*](http://www.ncbi.nlm.nih.gov/entrez/query.fcgi?db=gene&cmd=Retrieve&dopt=full_report&list_uids=19087) | protein kinase, cAMP dependent regulatory, type II alpha | 26 | 99 |
| mmu-miR-322-5p | [*Usp15*](http://www.ncbi.nlm.nih.gov/entrez/query.fcgi?db=gene&cmd=Retrieve&dopt=full_report&list_uids=14479) | ubiquitin specific peptidase 15 | 27 | 99 |
| mmu-miR-322-5p | [*Aff4*](http://www.ncbi.nlm.nih.gov/entrez/query.fcgi?db=gene&cmd=Retrieve&dopt=full_report&list_uids=93736) | AF4/FMR2 family, member 4 | 28 | 99 |
| mmu-miR-322-5p | [*Ncs1*](http://www.ncbi.nlm.nih.gov/entrez/query.fcgi?db=gene&cmd=Retrieve&dopt=full_report&list_uids=14299) | neuronal calcium sensor 1 | 29 | 99 |
| mmu-miR-322-5p | [*Lats1*](http://www.ncbi.nlm.nih.gov/entrez/query.fcgi?db=gene&cmd=Retrieve&dopt=full_report&list_uids=16798) | large tumor suppressor | 30 | 99 |
| mmu-miR-322-5p | [*Ube4b*](http://www.ncbi.nlm.nih.gov/entrez/query.fcgi?db=gene&cmd=Retrieve&dopt=full_report&list_uids=63958) | ubiquitination factor E4B | 31 | 99 |
| mmu-miR-322-5p | [*Erc2*](http://www.ncbi.nlm.nih.gov/entrez/query.fcgi?db=gene&cmd=Retrieve&dopt=full_report&list_uids=238988) | ELKS/RAB6-interacting/CAST family member 2 | 32 | 99 |
| mmu-miR-322-5p | [*Phc3*](http://www.ncbi.nlm.nih.gov/entrez/query.fcgi?db=gene&cmd=Retrieve&dopt=full_report&list_uids=241915) | polyhomeotic-like 3 (Drosophila) | 33 | 99 |
| mmu-miR-322-5p | [*Kl*](http://www.ncbi.nlm.nih.gov/entrez/query.fcgi?db=gene&cmd=Retrieve&dopt=full_report&list_uids=16591) | klotho | 34 | 98 |
| mmu-miR-322-5p | [*Tmem55a*](http://www.ncbi.nlm.nih.gov/entrez/query.fcgi?db=gene&cmd=Retrieve&dopt=full_report&list_uids=72519) | transmembrane protein 55A | 35 | 98 |
| mmu-miR-322-5p | [*Usp14*](http://www.ncbi.nlm.nih.gov/entrez/query.fcgi?db=gene&cmd=Retrieve&dopt=full_report&list_uids=59025) | ubiquitin specific peptidase 14 | 36 | 98 |
| mmu-miR-322-5p | [*Crebl2*](http://www.ncbi.nlm.nih.gov/entrez/query.fcgi?db=gene&cmd=Retrieve&dopt=full_report&list_uids=232430) | cAMP responsive element binding protein-like 2 | 37 | 98 |
| mmu-miR-322-5p | [*Reck*](http://www.ncbi.nlm.nih.gov/entrez/query.fcgi?db=gene&cmd=Retrieve&dopt=full_report&list_uids=53614) | reversion-inducing-cysteine-rich protein with kazal motifs | 38 | 98 |
| mmu-miR-322-5p | [*Ahcyl2*](http://www.ncbi.nlm.nih.gov/entrez/query.fcgi?db=gene&cmd=Retrieve&dopt=full_report&list_uids=74340) | S-adenosylhomocysteine hydrolase-like 2 | 39 | 98 |
| mmu-miR-322-5p | [*Colq*](http://www.ncbi.nlm.nih.gov/entrez/query.fcgi?db=gene&cmd=Retrieve&dopt=full_report&list_uids=382864) | collagen-like tail subunit (single strand of homotrimer) of asymmetric acetylcholinesterase | 40 | 98 |
| mmu-miR-322-5p | [*Cdc25a*](http://www.ncbi.nlm.nih.gov/entrez/query.fcgi?db=gene&cmd=Retrieve&dopt=full_report&list_uids=12530) | cell division cycle 25A | 41 | 98 |
| mmu-miR-322-5p | [*Stxbp3a*](http://www.ncbi.nlm.nih.gov/entrez/query.fcgi?db=gene&cmd=Retrieve&dopt=full_report&list_uids=20912) | syntaxin binding protein 3A | 42 | 98 |
| mmu-miR-322-5p | [*Tlk1*](http://www.ncbi.nlm.nih.gov/entrez/query.fcgi?db=gene&cmd=Retrieve&dopt=full_report&list_uids=228012) | tousled-like kinase 1 | 43 | 98 |
| mmu-miR-322-5p | [*Rad23b*](http://www.ncbi.nlm.nih.gov/entrez/query.fcgi?db=gene&cmd=Retrieve&dopt=full_report&list_uids=19359) | RAD23b homolog (S. cerevisiae) | 44 | 98 |
| mmu-miR-322-5p | [*Cnot6l*](http://www.ncbi.nlm.nih.gov/entrez/query.fcgi?db=gene&cmd=Retrieve&dopt=full_report&list_uids=231464) | CCR4-NOT transcription complex, subunit 6-like | 45 | 98 |
| mmu-miR-322-5p | [*Mfn2*](http://www.ncbi.nlm.nih.gov/entrez/query.fcgi?db=gene&cmd=Retrieve&dopt=full_report&list_uids=170731) | mitofusin 2 | 46 | 98 |
| mmu-miR-322-5p | [*Rasgef1b*](http://www.ncbi.nlm.nih.gov/entrez/query.fcgi?db=gene&cmd=Retrieve&dopt=full_report&list_uids=320292) | RasGEF domain family, member 1B | 47 | 98 |
| mmu-miR-322-5p | [*Usp31*](http://www.ncbi.nlm.nih.gov/entrez/query.fcgi?db=gene&cmd=Retrieve&dopt=full_report&list_uids=76179) | ubiquitin specific peptidase 31 | 48 | 98 |
| mmu-miR-322-5p | [*Fam110c*](http://www.ncbi.nlm.nih.gov/entrez/query.fcgi?db=gene&cmd=Retrieve&dopt=full_report&list_uids=104943) | family with sequence similarity 110, member C | 49 | 98 |
| mmu-miR-322-5p | [*Ddx3x*](http://www.ncbi.nlm.nih.gov/entrez/query.fcgi?db=gene&cmd=Retrieve&dopt=full_report&list_uids=13205) | DEAD/H (Asp-Glu-Ala-Asp/His) box polypeptide 3, X-linked | 50 | 98 |
| mmu-miR-322-5p | [*Rfx3*](http://www.ncbi.nlm.nih.gov/entrez/query.fcgi?db=gene&cmd=Retrieve&dopt=full_report&list_uids=19726) | regulatory factor X, 3 (influences HLA class II expression) | 51 | 98 |
| mmu-miR-322-5p | [*Lrp6*](http://www.ncbi.nlm.nih.gov/entrez/query.fcgi?db=gene&cmd=Retrieve&dopt=full_report&list_uids=16974) | low density lipoprotein receptor-related protein 6 | 52 | 98 |
| mmu-miR-322-5p | [*Mapk8*](http://www.ncbi.nlm.nih.gov/entrez/query.fcgi?db=gene&cmd=Retrieve&dopt=full_report&list_uids=26419) | mitogen-activated protein kinase 8 | 53 | 98 |
| mmu-miR-322-5p | [*Wee1*](http://www.ncbi.nlm.nih.gov/entrez/query.fcgi?db=gene&cmd=Retrieve&dopt=full_report&list_uids=22390) | WEE 1 homolog 1 (S. pombe) | 54 | 98 |
| mmu-miR-322-5p | [*Smad7*](http://www.ncbi.nlm.nih.gov/entrez/query.fcgi?db=gene&cmd=Retrieve&dopt=full_report&list_uids=17131) | SMAD family member 7 | 55 | 98 |
| mmu-miR-322-5p | [*Etnk1*](http://www.ncbi.nlm.nih.gov/entrez/query.fcgi?db=gene&cmd=Retrieve&dopt=full_report&list_uids=75320) | ethanolamine kinase 1 | 56 | 98 |
| mmu-miR-322-5p | [*Bicd1*](http://www.ncbi.nlm.nih.gov/entrez/query.fcgi?db=gene&cmd=Retrieve&dopt=full_report&list_uids=12121) | bicaudal D homolog 1 (Drosophila) | 57 | 98 |
| mmu-miR-322-5p | [*Dync1li2*](http://www.ncbi.nlm.nih.gov/entrez/query.fcgi?db=gene&cmd=Retrieve&dopt=full_report&list_uids=234663) | dynein, cytoplasmic 1 light intermediate chain 2 | 58 | 97 |
| mmu-miR-322-5p | [*Kpna1*](http://www.ncbi.nlm.nih.gov/entrez/query.fcgi?db=gene&cmd=Retrieve&dopt=full_report&list_uids=16646) | karyopherin (importin) alpha 1 | 59 | 97 |
| mmu-miR-322-5p | [*Clspn*](http://www.ncbi.nlm.nih.gov/entrez/query.fcgi?db=gene&cmd=Retrieve&dopt=full_report&list_uids=269582) | claspin | 60 | 97 |
| mmu-miR-322-5p | [*C77370*](http://www.ncbi.nlm.nih.gov/entrez/query.fcgi?db=gene&cmd=Retrieve&dopt=full_report&list_uids=245555) | expressed sequence C77370 | 61 | 97 |
| mmu-miR-322-5p | [*Rab9b*](http://www.ncbi.nlm.nih.gov/entrez/query.fcgi?db=gene&cmd=Retrieve&dopt=full_report&list_uids=319642) | RAB9B, member RAS oncogene family | 62 | 97 |
| mmu-miR-322-5p | [*Rab11fip1*](http://www.ncbi.nlm.nih.gov/entrez/query.fcgi?db=gene&cmd=Retrieve&dopt=full_report&list_uids=75767) | RAB11 family interacting protein 1 (class I) | 63 | 97 |
| mmu-miR-322-5p | [*Fam133b*](http://www.ncbi.nlm.nih.gov/entrez/query.fcgi?db=gene&cmd=Retrieve&dopt=full_report&list_uids=68152) | family with sequence similarity 133, member B | 64 | 97 |
| mmu-miR-322-5p | [*Btg2*](http://www.ncbi.nlm.nih.gov/entrez/query.fcgi?db=gene&cmd=Retrieve&dopt=full_report&list_uids=12227) | B cell translocation gene 2, anti-proliferative | 65 | 97 |
| mmu-miR-322-5p | [*Atp7a*](http://www.ncbi.nlm.nih.gov/entrez/query.fcgi?db=gene&cmd=Retrieve&dopt=full_report&list_uids=11977) | ATPase, Cu++ transporting, alpha polypeptide | 66 | 97 |
| mmu-miR-322-5p | [*Ash1l*](http://www.ncbi.nlm.nih.gov/entrez/query.fcgi?db=gene&cmd=Retrieve&dopt=full_report&list_uids=192195) | ash1 (absent, small, or homeotic)-like (Drosophila) | 67 | 97 |
| mmu-miR-322-5p | [*Zbtb42*](http://www.ncbi.nlm.nih.gov/entrez/query.fcgi?db=gene&cmd=Retrieve&dopt=full_report&list_uids=382639) | zinc finger and BTB domain containing 42 | 68 | 97 |
| mmu-miR-322-5p | [*Slit2*](http://www.ncbi.nlm.nih.gov/entrez/query.fcgi?db=gene&cmd=Retrieve&dopt=full_report&list_uids=20563) | slit homolog 2 (Drosophila) | 69 | 97 |
| mmu-miR-322-5p | [*Lphn1*](http://www.ncbi.nlm.nih.gov/entrez/query.fcgi?db=gene&cmd=Retrieve&dopt=full_report&list_uids=330814) | latrophilin 1 | 70 | 97 |
| mmu-miR-322-5p | [*Socs6*](http://www.ncbi.nlm.nih.gov/entrez/query.fcgi?db=gene&cmd=Retrieve&dopt=full_report&list_uids=54607) | suppressor of cytokine signaling 6 | 71 | 97 |
| mmu-miR-322-5p | [*Cpsf7*](http://www.ncbi.nlm.nih.gov/entrez/query.fcgi?db=gene&cmd=Retrieve&dopt=full_report&list_uids=269061) | cleavage and polyadenylation specific factor 7 | 72 | 97 |
| mmu-miR-322-5p | [*Prr16*](http://www.ncbi.nlm.nih.gov/entrez/query.fcgi?db=gene&cmd=Retrieve&dopt=full_report&list_uids=71373) | proline rich 16 | 73 | 97 |
| mmu-miR-322-5p | [*Polr3f*](http://www.ncbi.nlm.nih.gov/entrez/query.fcgi?db=gene&cmd=Retrieve&dopt=full_report&list_uids=70408) | polymerase (RNA) III (DNA directed) polypeptide F | 74 | 97 |
| mmu-miR-322-5p | [*1700025G04Rik*](http://www.ncbi.nlm.nih.gov/entrez/query.fcgi?db=gene&cmd=Retrieve&dopt=full_report&list_uids=69399) | RIKEN cDNA 1700025G04 gene | 75 | 97 |
| mmu-miR-322-5p | [*Actr2*](http://www.ncbi.nlm.nih.gov/entrez/query.fcgi?db=gene&cmd=Retrieve&dopt=full_report&list_uids=66713) | ARP2 actin-related protein 2 | 76 | 97 |
| mmu-miR-322-5p | [*Pvrl1*](http://www.ncbi.nlm.nih.gov/entrez/query.fcgi?db=gene&cmd=Retrieve&dopt=full_report&list_uids=58235) | poliovirus receptor-related 1 | 77 | 97 |
| mmu-miR-322-5p | [*Atp1b4*](http://www.ncbi.nlm.nih.gov/entrez/query.fcgi?db=gene&cmd=Retrieve&dopt=full_report&list_uids=67821) | ATPase, (Na+)/K+ transporting, beta 4 polypeptide | 78 | 97 |
| mmu-miR-322-5p | [*Arhgdia*](http://www.ncbi.nlm.nih.gov/entrez/query.fcgi?db=gene&cmd=Retrieve&dopt=full_report&list_uids=192662) | Rho GDP dissociation inhibitor (GDI) alpha | 79 | 97 |
| mmu-miR-322-5p | [*Trp53inp2*](http://www.ncbi.nlm.nih.gov/entrez/query.fcgi?db=gene&cmd=Retrieve&dopt=full_report&list_uids=68728) | transformation related protein 53 inducible nuclear protein 2 | 80 | 97 |
| mmu-miR-322-5p | [*Kdsr*](http://www.ncbi.nlm.nih.gov/entrez/query.fcgi?db=gene&cmd=Retrieve&dopt=full_report&list_uids=70750) | 3-ketodihydrosphingosine reductase | 81 | 97 |
| mmu-miR-322-5p | [*Zbtb46*](http://www.ncbi.nlm.nih.gov/entrez/query.fcgi?db=gene&cmd=Retrieve&dopt=full_report&list_uids=72147) | zinc finger and BTB domain containing 46 | 82 | 97 |
| mmu-miR-322-5p | [*Sall1*](http://www.ncbi.nlm.nih.gov/entrez/query.fcgi?db=gene&cmd=Retrieve&dopt=full_report&list_uids=58198) | sal-like 1 (Drosophila) | 83 | 97 |
| mmu-miR-322-5p | [*Tbpl1*](http://www.ncbi.nlm.nih.gov/entrez/query.fcgi?db=gene&cmd=Retrieve&dopt=full_report&list_uids=237336) | TATA box binding protein-like 1 | 84 | 97 |
| mmu-miR-322-5p | [*Rasef*](http://www.ncbi.nlm.nih.gov/entrez/query.fcgi?db=gene&cmd=Retrieve&dopt=full_report&list_uids=242505) | RAS and EF hand domain containing | 85 | 97 |
| mmu-miR-322-5p | [*Rbm6*](http://www.ncbi.nlm.nih.gov/entrez/query.fcgi?db=gene&cmd=Retrieve&dopt=full_report&list_uids=19654) | RNA binding motif protein 6 | 86 | 97 |
| mmu-miR-322-5p | [*Zswim3*](http://www.ncbi.nlm.nih.gov/entrez/query.fcgi?db=gene&cmd=Retrieve&dopt=full_report&list_uids=67538) | zinc finger SWIM-type containing 3 | 87 | 97 |
| mmu-miR-322-5p | [*Insr*](http://www.ncbi.nlm.nih.gov/entrez/query.fcgi?db=gene&cmd=Retrieve&dopt=full_report&list_uids=16337) | insulin receptor | 88 | 97 |
| mmu-miR-322-5p | [*C1qtnf2*](http://www.ncbi.nlm.nih.gov/entrez/query.fcgi?db=gene&cmd=Retrieve&dopt=full_report&list_uids=69183) | C1q and tumor necrosis factor related protein 2 | 89 | 97 |
| mmu-miR-322-5p | [*Fgf9*](http://www.ncbi.nlm.nih.gov/entrez/query.fcgi?db=gene&cmd=Retrieve&dopt=full_report&list_uids=14180) | fibroblast growth factor 9 | 90 | 96 |
| mmu-miR-322-5p | [*Zcchc3*](http://www.ncbi.nlm.nih.gov/entrez/query.fcgi?db=gene&cmd=Retrieve&dopt=full_report&list_uids=67917) | zinc finger, CCHC domain containing 3 | 91 | 96 |
| mmu-miR-322-5p | [*Ept1*](http://www.ncbi.nlm.nih.gov/entrez/query.fcgi?db=gene&cmd=Retrieve&dopt=full_report&list_uids=28042) | ethanolaminephosphotransferase 1 (CDP-ethanolamine-specific) | 92 | 96 |
| mmu-miR-322-5p | [*Slc13a3*](http://www.ncbi.nlm.nih.gov/entrez/query.fcgi?db=gene&cmd=Retrieve&dopt=full_report&list_uids=114644) | solute carrier family 13 (sodium-dependent dicarboxylate transporter), member 3 | 93 | 96 |
| mmu-miR-322-5p | [*Hus1*](http://www.ncbi.nlm.nih.gov/entrez/query.fcgi?db=gene&cmd=Retrieve&dopt=full_report&list_uids=15574) | Hus1 homolog (S. pombe) | 94 | 96 |
| mmu-miR-322-5p | [*Soga1*](http://www.ncbi.nlm.nih.gov/entrez/query.fcgi?db=gene&cmd=Retrieve&dopt=full_report&list_uids=320706) | suppressor of glucose, autophagy associated 1 | 95 | 96 |
| mmu-miR-322-5p | [*Tcaim*](http://www.ncbi.nlm.nih.gov/entrez/query.fcgi?db=gene&cmd=Retrieve&dopt=full_report&list_uids=382117) | T cell activation inhibitor, mitochondrial | 96 | 96 |
| mmu-miR-322-5p | [*Zbtb44*](http://www.ncbi.nlm.nih.gov/entrez/query.fcgi?db=gene&cmd=Retrieve&dopt=full_report&list_uids=235132) | zinc finger and BTB domain containing 44 | 97 | 96 |
| mmu-miR-322-5p | [*Ppm1d*](http://www.ncbi.nlm.nih.gov/entrez/query.fcgi?db=gene&cmd=Retrieve&dopt=full_report&list_uids=53892) | protein phosphatase 1D magnesium-dependent, delta isoform | 98 | 96 |
| mmu-miR-322-5p | [*Fermt2*](http://www.ncbi.nlm.nih.gov/entrez/query.fcgi?db=gene&cmd=Retrieve&dopt=full_report&list_uids=218952) | fermitin family homolog 2 (Drosophila) | 99 | 96 |
| mmu-miR-322-5p | [*Chd2*](http://www.ncbi.nlm.nih.gov/entrez/query.fcgi?db=gene&cmd=Retrieve&dopt=full_report&list_uids=244059) | chromodomain helicase DNA binding protein 2 | 100 | 96 |
| mmu-miR-322-5p | [*Kif21a*](http://www.ncbi.nlm.nih.gov/entrez/query.fcgi?db=gene&cmd=Retrieve&dopt=full_report&list_uids=16564) | kinesin family member 21A | 101 | 96 |
| mmu-miR-322-5p | [*Srek1*](http://www.ncbi.nlm.nih.gov/entrez/query.fcgi?db=gene&cmd=Retrieve&dopt=full_report&list_uids=218543) | splicing regulatory glutamine/lysine-rich protein 1 | 102 | 96 |
| mmu-miR-322-5p | [*Prdm4*](http://www.ncbi.nlm.nih.gov/entrez/query.fcgi?db=gene&cmd=Retrieve&dopt=full_report&list_uids=72843) | PR domain containing 4 | 103 | 96 |
| mmu-miR-322-5p | [*Ipo7*](http://www.ncbi.nlm.nih.gov/entrez/query.fcgi?db=gene&cmd=Retrieve&dopt=full_report&list_uids=233726) | importin 7 | 104 | 96 |
| mmu-miR-322-5p | [*Zfp275*](http://www.ncbi.nlm.nih.gov/entrez/query.fcgi?db=gene&cmd=Retrieve&dopt=full_report&list_uids=27081) | zinc finger protein 275 | 105 | 96 |
| mmu-miR-322-5p | [*Amotl1*](http://www.ncbi.nlm.nih.gov/entrez/query.fcgi?db=gene&cmd=Retrieve&dopt=full_report&list_uids=75723) | angiomotin-like 1 | 106 | 96 |
| mmu-miR-322-5p | [*Cul2*](http://www.ncbi.nlm.nih.gov/entrez/query.fcgi?db=gene&cmd=Retrieve&dopt=full_report&list_uids=71745) | cullin 2 | 107 | 96 |
| mmu-miR-322-5p | [*Hpcal4*](http://www.ncbi.nlm.nih.gov/entrez/query.fcgi?db=gene&cmd=Retrieve&dopt=full_report&list_uids=170638) | hippocalcin-like 4 | 108 | 95 |
| mmu-miR-322-5p | [*Nt5dc3*](http://www.ncbi.nlm.nih.gov/entrez/query.fcgi?db=gene&cmd=Retrieve&dopt=full_report&list_uids=103466) | 5'-nucleotidase domain containing 3 | 109 | 95 |
| mmu-miR-322-5p | [*Crebrf*](http://www.ncbi.nlm.nih.gov/entrez/query.fcgi?db=gene&cmd=Retrieve&dopt=full_report&list_uids=77128) | CREB3 regulatory factor | 110 | 95 |
| mmu-miR-322-5p | [*Phip*](http://www.ncbi.nlm.nih.gov/entrez/query.fcgi?db=gene&cmd=Retrieve&dopt=full_report&list_uids=83946) | pleckstrin homology domain interacting protein | 111 | 95 |
| mmu-miR-322-5p | [*Arih1*](http://www.ncbi.nlm.nih.gov/entrez/query.fcgi?db=gene&cmd=Retrieve&dopt=full_report&list_uids=23806) | ariadne ubiquitin-conjugating enzyme E2 binding protein homolog 1 (Drosophila) | 112 | 95 |
| mmu-miR-322-5p | [*Casr*](http://www.ncbi.nlm.nih.gov/entrez/query.fcgi?db=gene&cmd=Retrieve&dopt=full_report&list_uids=12374) | calcium-sensing receptor | 113 | 95 |
| mmu-miR-322-5p | [*Cpeb3*](http://www.ncbi.nlm.nih.gov/entrez/query.fcgi?db=gene&cmd=Retrieve&dopt=full_report&list_uids=208922) | cytoplasmic polyadenylation element binding protein 3 | 114 | 95 |
| mmu-miR-322-5p | [*Zfp622*](http://www.ncbi.nlm.nih.gov/entrez/query.fcgi?db=gene&cmd=Retrieve&dopt=full_report&list_uids=52521) | zinc finger protein 622 | 115 | 95 |
| mmu-miR-322-5p | [*Aar2*](http://www.ncbi.nlm.nih.gov/entrez/query.fcgi?db=gene&cmd=Retrieve&dopt=full_report&list_uids=68295) | AAR2 splicing factor homolog (S. cerevisiae) | 116 | 95 |
| mmu-miR-322-5p | [*Spred1*](http://www.ncbi.nlm.nih.gov/entrez/query.fcgi?db=gene&cmd=Retrieve&dopt=full_report&list_uids=114715) | sprouty protein with EVH-1 domain 1, related sequence | 117 | 95 |
| mmu-miR-322-5p | [*Dcaf7*](http://www.ncbi.nlm.nih.gov/entrez/query.fcgi?db=gene&cmd=Retrieve&dopt=full_report&list_uids=71833) | DDB1 and CUL4 associated factor 7 | 118 | 95 |
| mmu-miR-322-5p | [*Sypl*](http://www.ncbi.nlm.nih.gov/entrez/query.fcgi?db=gene&cmd=Retrieve&dopt=full_report&list_uids=19027) | synaptophysin-like protein | 119 | 95 |
| mmu-miR-322-5p | [*Pth*](http://www.ncbi.nlm.nih.gov/entrez/query.fcgi?db=gene&cmd=Retrieve&dopt=full_report&list_uids=19226) | parathyroid hormone | 120 | 95 |
| mmu-miR-322-5p | [*8430427H17Rik*](http://www.ncbi.nlm.nih.gov/entrez/query.fcgi?db=gene&cmd=Retrieve&dopt=full_report&list_uids=329540) | RIKEN cDNA 8430427H17 gene | 121 | 95 |
| mmu-miR-322-5p | [*Plekhm3*](http://www.ncbi.nlm.nih.gov/entrez/query.fcgi?db=gene&cmd=Retrieve&dopt=full_report&list_uids=241075) | pleckstrin homology domain containing, family M, member 3 | 122 | 95 |
| mmu-miR-322-5p | [*Caprin1*](http://www.ncbi.nlm.nih.gov/entrez/query.fcgi?db=gene&cmd=Retrieve&dopt=full_report&list_uids=53872) | cell cycle associated protein 1 | 123 | 95 |
| mmu-miR-322-5p | [*Slc4a7*](http://www.ncbi.nlm.nih.gov/entrez/query.fcgi?db=gene&cmd=Retrieve&dopt=full_report&list_uids=218756) | solute carrier family 4, sodium bicarbonate cotransporter, member 7 | 124 | 95 |
| mmu-miR-322-5p | [*Stradb*](http://www.ncbi.nlm.nih.gov/entrez/query.fcgi?db=gene&cmd=Retrieve&dopt=full_report&list_uids=227154) | STE20-related kinase adaptor beta | 125 | 95 |
| mmu-miR-322-5p | [*Dync1i1*](http://www.ncbi.nlm.nih.gov/entrez/query.fcgi?db=gene&cmd=Retrieve&dopt=full_report&list_uids=13426) | dynein cytoplasmic 1 intermediate chain 1 | 126 | 94 |
| mmu-miR-322-5p | [*Nup210*](http://www.ncbi.nlm.nih.gov/entrez/query.fcgi?db=gene&cmd=Retrieve&dopt=full_report&list_uids=54563) | nucleoporin 210 | 127 | 94 |
| mmu-miR-322-5p | [*Cdc37l1*](http://www.ncbi.nlm.nih.gov/entrez/query.fcgi?db=gene&cmd=Retrieve&dopt=full_report&list_uids=67072) | cell division cycle 37-like 1 | 128 | 94 |
| mmu-miR-322-5p | [*Wipi2*](http://www.ncbi.nlm.nih.gov/entrez/query.fcgi?db=gene&cmd=Retrieve&dopt=full_report&list_uids=74781) | WD repeat domain, phosphoinositide interacting 2 | 129 | 94 |
| mmu-miR-322-5p | [*Sowahc*](http://www.ncbi.nlm.nih.gov/entrez/query.fcgi?db=gene&cmd=Retrieve&dopt=full_report&list_uids=268301) | sosondowah ankyrin repeat domain family member C | 130 | 94 |
| mmu-miR-322-5p | [*Il10ra*](http://www.ncbi.nlm.nih.gov/entrez/query.fcgi?db=gene&cmd=Retrieve&dopt=full_report&list_uids=16154) | interleukin 10 receptor, alpha | 131 | 94 |
| mmu-miR-322-5p | [*Ezh1*](http://www.ncbi.nlm.nih.gov/entrez/query.fcgi?db=gene&cmd=Retrieve&dopt=full_report&list_uids=14055) | enhancer of zeste homolog 1 (Drosophila) | 132 | 94 |
| mmu-miR-322-5p | [*Tmem150c*](http://www.ncbi.nlm.nih.gov/entrez/query.fcgi?db=gene&cmd=Retrieve&dopt=full_report&list_uids=231503) | transmembrane protein 150C | 133 | 94 |
| mmu-miR-322-5p | [*Nfatc3*](http://www.ncbi.nlm.nih.gov/entrez/query.fcgi?db=gene&cmd=Retrieve&dopt=full_report&list_uids=18021) | nuclear factor of activated T cells, cytoplasmic, calcineurin dependent 3 | 134 | 94 |
| mmu-miR-322-5p | [*Rab10*](http://www.ncbi.nlm.nih.gov/entrez/query.fcgi?db=gene&cmd=Retrieve&dopt=full_report&list_uids=19325) | RAB10, member RAS oncogene family | 135 | 94 |
| mmu-miR-322-5p | [*Eif3a*](http://www.ncbi.nlm.nih.gov/entrez/query.fcgi?db=gene&cmd=Retrieve&dopt=full_report&list_uids=13669) | eukaryotic translation initiation factor 3, subunit A | 136 | 94 |
| mmu-miR-322-5p | [*Fbxw7*](http://www.ncbi.nlm.nih.gov/entrez/query.fcgi?db=gene&cmd=Retrieve&dopt=full_report&list_uids=50754) | F-box and WD-40 domain protein 7 | 137 | 94 |
| mmu-miR-322-5p | [*Lrrc32*](http://www.ncbi.nlm.nih.gov/entrez/query.fcgi?db=gene&cmd=Retrieve&dopt=full_report&list_uids=434215) | leucine rich repeat containing 32 | 138 | 94 |
| mmu-miR-322-5p | [*Tgfbr3*](http://www.ncbi.nlm.nih.gov/entrez/query.fcgi?db=gene&cmd=Retrieve&dopt=full_report&list_uids=21814) | transforming growth factor, beta receptor III | 139 | 94 |
| mmu-miR-322-5p | [*Plekhh1*](http://www.ncbi.nlm.nih.gov/entrez/query.fcgi?db=gene&cmd=Retrieve&dopt=full_report&list_uids=211945) | pleckstrin homology domain containing, family H (with MyTH4 domain) member 1 | 140 | 94 |
| mmu-miR-322-5p | [*Slc12a1*](http://www.ncbi.nlm.nih.gov/entrez/query.fcgi?db=gene&cmd=Retrieve&dopt=full_report&list_uids=20495) | solute carrier family 12, member 1 | 141 | 94 |
| mmu-miR-322-5p | [*Ret*](http://www.ncbi.nlm.nih.gov/entrez/query.fcgi?db=gene&cmd=Retrieve&dopt=full_report&list_uids=19713) | ret proto-oncogene | 142 | 94 |
| mmu-miR-322-5p | [*Usp42*](http://www.ncbi.nlm.nih.gov/entrez/query.fcgi?db=gene&cmd=Retrieve&dopt=full_report&list_uids=76800) | ubiquitin specific peptidase 42 | 143 | 94 |
| mmu-miR-322-5p | [*Arl2*](http://www.ncbi.nlm.nih.gov/entrez/query.fcgi?db=gene&cmd=Retrieve&dopt=full_report&list_uids=56327) | ADP-ribosylation factor-like 2 | 144 | 94 |
| mmu-miR-322-5p | [*Cpd*](http://www.ncbi.nlm.nih.gov/entrez/query.fcgi?db=gene&cmd=Retrieve&dopt=full_report&list_uids=12874) | carboxypeptidase D | 145 | 94 |
| mmu-miR-322-5p | [*3632451O06Rik*](http://www.ncbi.nlm.nih.gov/entrez/query.fcgi?db=gene&cmd=Retrieve&dopt=full_report&list_uids=67419) | RIKEN cDNA 3632451O06 gene | 146 | 94 |
| mmu-miR-322-5p | [*Epb4.1l4b*](http://www.ncbi.nlm.nih.gov/entrez/query.fcgi?db=gene&cmd=Retrieve&dopt=full_report&list_uids=54357) | erythrocyte protein band 4.1-like 4b | 147 | 94 |
| mmu-miR-322-5p | [*Abhd13*](http://www.ncbi.nlm.nih.gov/entrez/query.fcgi?db=gene&cmd=Retrieve&dopt=full_report&list_uids=68904) | abhydrolase domain containing 13 | 148 | 94 |
| mmu-miR-322-5p | [*B3gnt6*](http://www.ncbi.nlm.nih.gov/entrez/query.fcgi?db=gene&cmd=Retrieve&dopt=full_report&list_uids=272411) | UDP-GlcNAc:betaGal beta-1,3-N-acetylglucosaminyltransferase 6 (core 3 synthase) | 149 | 94 |
| mmu-miR-322-5p | [*Ccne1*](http://www.ncbi.nlm.nih.gov/entrez/query.fcgi?db=gene&cmd=Retrieve&dopt=full_report&list_uids=12447) | cyclin E1 | 150 | 94 |
| mmu-miR-322-5p | [*Kctd8*](http://www.ncbi.nlm.nih.gov/entrez/query.fcgi?db=gene&cmd=Retrieve&dopt=full_report&list_uids=243043) | potassium channel tetramerisation domain containing 8 | 151 | 93 |
| mmu-miR-322-5p | [*Mtmr3*](http://www.ncbi.nlm.nih.gov/entrez/query.fcgi?db=gene&cmd=Retrieve&dopt=full_report&list_uids=74302) | myotubularin related protein 3 | 152 | 93 |
| mmu-miR-322-5p | [*Napb*](http://www.ncbi.nlm.nih.gov/entrez/query.fcgi?db=gene&cmd=Retrieve&dopt=full_report&list_uids=17957) | N-ethylmaleimide sensitive fusion protein attachment protein beta | 153 | 93 |
| mmu-miR-322-5p | [*Arhgap12*](http://www.ncbi.nlm.nih.gov/entrez/query.fcgi?db=gene&cmd=Retrieve&dopt=full_report&list_uids=75415) | Rho GTPase activating protein 12 | 154 | 93 |
| mmu-miR-322-5p | [*Vstm2a*](http://www.ncbi.nlm.nih.gov/entrez/query.fcgi?db=gene&cmd=Retrieve&dopt=full_report&list_uids=211739) | V-set and transmembrane domain containing 2A | 155 | 93 |
| mmu-miR-322-5p | [*Elmsan1*](http://www.ncbi.nlm.nih.gov/entrez/query.fcgi?db=gene&cmd=Retrieve&dopt=full_report&list_uids=238317) | ELM2 and Myb/SANT-like domain containing 1 | 156 | 93 |
| mmu-miR-322-5p | [*5031414D18Rik*](http://www.ncbi.nlm.nih.gov/entrez/query.fcgi?db=gene&cmd=Retrieve&dopt=full_report&list_uids=271221) | RIKEN cDNA 5031414D18 gene | 157 | 93 |
| mmu-miR-322-5p | [*Slc7a2*](http://www.ncbi.nlm.nih.gov/entrez/query.fcgi?db=gene&cmd=Retrieve&dopt=full_report&list_uids=11988) | solute carrier family 7 (cationic amino acid transporter, y+ system), member 2 | 158 | 93 |
| mmu-miR-322-5p | [*Ywhah*](http://www.ncbi.nlm.nih.gov/entrez/query.fcgi?db=gene&cmd=Retrieve&dopt=full_report&list_uids=22629) | tyrosine 3-monooxygenase/tryptophan 5-monooxygenase activation protein, eta polypeptide | 159 | 93 |
| mmu-miR-322-5p | [*Ccnt2*](http://www.ncbi.nlm.nih.gov/entrez/query.fcgi?db=gene&cmd=Retrieve&dopt=full_report&list_uids=72949) | cyclin T2 | 160 | 93 |
| mmu-miR-322-5p | [*Ywhaq*](http://www.ncbi.nlm.nih.gov/entrez/query.fcgi?db=gene&cmd=Retrieve&dopt=full_report&list_uids=22630) | tyrosine 3-monooxygenase/tryptophan 5-monooxygenase activation protein, theta polypeptide | 161 | 93 |
| mmu-miR-322-5p | [*Rims3*](http://www.ncbi.nlm.nih.gov/entrez/query.fcgi?db=gene&cmd=Retrieve&dopt=full_report&list_uids=242662) | regulating synaptic membrane exocytosis 3 | 162 | 93 |
| mmu-miR-322-5p | [*Tmem178b*](http://www.ncbi.nlm.nih.gov/entrez/query.fcgi?db=gene&cmd=Retrieve&dopt=full_report&list_uids=434008) | transmembrane protein 178B | 163 | 93 |
| mmu-miR-322-5p | [*Rnf10*](http://www.ncbi.nlm.nih.gov/entrez/query.fcgi?db=gene&cmd=Retrieve&dopt=full_report&list_uids=50849) | ring finger protein 10 | 164 | 93 |
| mmu-miR-322-5p | [*Eif2b2*](http://www.ncbi.nlm.nih.gov/entrez/query.fcgi?db=gene&cmd=Retrieve&dopt=full_report&list_uids=217715) | eukaryotic translation initiation factor 2B, subunit 2 beta | 165 | 93 |
| mmu-miR-322-5p | [*Erlin2*](http://www.ncbi.nlm.nih.gov/entrez/query.fcgi?db=gene&cmd=Retrieve&dopt=full_report&list_uids=244373) | ER lipid raft associated 2 | 166 | 93 |
| mmu-miR-322-5p | [*Plxna2*](http://www.ncbi.nlm.nih.gov/entrez/query.fcgi?db=gene&cmd=Retrieve&dopt=full_report&list_uids=18845) | plexin A2 | 167 | 93 |
| mmu-miR-322-5p | [*Sfrs18*](http://www.ncbi.nlm.nih.gov/entrez/query.fcgi?db=gene&cmd=Retrieve&dopt=full_report&list_uids=66625) | serine/arginine-rich splicing factor 18 | 168 | 93 |
| mmu-miR-322-5p | [*Slc39a10*](http://www.ncbi.nlm.nih.gov/entrez/query.fcgi?db=gene&cmd=Retrieve&dopt=full_report&list_uids=227059) | solute carrier family 39 (zinc transporter), member 10 | 169 | 93 |
| mmu-miR-322-5p | [*Ptpn4*](http://www.ncbi.nlm.nih.gov/entrez/query.fcgi?db=gene&cmd=Retrieve&dopt=full_report&list_uids=19258) | protein tyrosine phosphatase, non-receptor type 4 | 170 | 93 |
| mmu-miR-322-5p | [*Tmem255a*](http://www.ncbi.nlm.nih.gov/entrez/query.fcgi?db=gene&cmd=Retrieve&dopt=full_report&list_uids=245386) | transmembrane protein 255A | 171 | 92 |
| mmu-miR-322-5p | [*Osbpl7*](http://www.ncbi.nlm.nih.gov/entrez/query.fcgi?db=gene&cmd=Retrieve&dopt=full_report&list_uids=71240) | oxysterol binding protein-like 7 | 172 | 92 |
| mmu-miR-322-5p | [*Sox6*](http://www.ncbi.nlm.nih.gov/entrez/query.fcgi?db=gene&cmd=Retrieve&dopt=full_report&list_uids=20679) | SRY (sex determining region Y)-box 6 | 173 | 92 |
| mmu-miR-322-5p | [*Zfp46*](http://www.ncbi.nlm.nih.gov/entrez/query.fcgi?db=gene&cmd=Retrieve&dopt=full_report&list_uids=22704) | zinc finger protein 46 | 174 | 92 |
| mmu-miR-322-5p | [*Slc2a13*](http://www.ncbi.nlm.nih.gov/entrez/query.fcgi?db=gene&cmd=Retrieve&dopt=full_report&list_uids=239606) | solute carrier family 2 (facilitated glucose transporter), member 13 | 175 | 92 |
| mmu-miR-322-5p | [*Ubn2*](http://www.ncbi.nlm.nih.gov/entrez/query.fcgi?db=gene&cmd=Retrieve&dopt=full_report&list_uids=320538) | ubinuclein 2 | 176 | 92 |
| mmu-miR-322-5p | [*Kif23*](http://www.ncbi.nlm.nih.gov/entrez/query.fcgi?db=gene&cmd=Retrieve&dopt=full_report&list_uids=71819) | kinesin family member 23 | 177 | 92 |
| mmu-miR-322-5p | [*Med1*](http://www.ncbi.nlm.nih.gov/entrez/query.fcgi?db=gene&cmd=Retrieve&dopt=full_report&list_uids=19014) | mediator complex subunit 1 | 178 | 92 |
| mmu-miR-322-5p | [*Kmt2a*](http://www.ncbi.nlm.nih.gov/entrez/query.fcgi?db=gene&cmd=Retrieve&dopt=full_report&list_uids=214162) | lysine (K)-specific methyltransferase 2A | 179 | 92 |
| mmu-miR-322-5p | [*Cbx6*](http://www.ncbi.nlm.nih.gov/entrez/query.fcgi?db=gene&cmd=Retrieve&dopt=full_report&list_uids=494448) | chromobox 6 | 180 | 92 |
| mmu-miR-322-5p | [*Kif1b*](http://www.ncbi.nlm.nih.gov/entrez/query.fcgi?db=gene&cmd=Retrieve&dopt=full_report&list_uids=16561) | kinesin family member 1B | 181 | 92 |
| mmu-miR-322-5p | [*Cdc42ep2*](http://www.ncbi.nlm.nih.gov/entrez/query.fcgi?db=gene&cmd=Retrieve&dopt=full_report&list_uids=104252) | CDC42 effector protein (Rho GTPase binding) 2 | 182 | 92 |
| mmu-miR-322-5p | [*Arhgap5*](http://www.ncbi.nlm.nih.gov/entrez/query.fcgi?db=gene&cmd=Retrieve&dopt=full_report&list_uids=11855) | Rho GTPase activating protein 5 | 183 | 92 |
| mmu-miR-322-5p | [*Akap11*](http://www.ncbi.nlm.nih.gov/entrez/query.fcgi?db=gene&cmd=Retrieve&dopt=full_report&list_uids=219181) | A kinase (PRKA) anchor protein 11 | 184 | 92 |
| mmu-miR-322-5p | [*Clock*](http://www.ncbi.nlm.nih.gov/entrez/query.fcgi?db=gene&cmd=Retrieve&dopt=full_report&list_uids=12753) | circadian locomotor output cycles kaput | 185 | 92 |
| mmu-miR-322-5p | [*Sft2d3*](http://www.ncbi.nlm.nih.gov/entrez/query.fcgi?db=gene&cmd=Retrieve&dopt=full_report&list_uids=67158) | SFT2 domain containing 3 | 186 | 92 |
| mmu-miR-322-5p | [*Gbp2b*](http://www.ncbi.nlm.nih.gov/entrez/query.fcgi?db=gene&cmd=Retrieve&dopt=full_report&list_uids=14468) | guanylate binding protein 2b | 187 | 92 |
| mmu-miR-322-5p | [*Ankib1*](http://www.ncbi.nlm.nih.gov/entrez/query.fcgi?db=gene&cmd=Retrieve&dopt=full_report&list_uids=70797) | ankyrin repeat and IBR domain containing 1 | 188 | 92 |
| mmu-miR-322-5p | [*Cacna2d1*](http://www.ncbi.nlm.nih.gov/entrez/query.fcgi?db=gene&cmd=Retrieve&dopt=full_report&list_uids=12293) | calcium channel, voltage-dependent, alpha2/delta subunit 1 | 189 | 92 |
| mmu-miR-322-5p | [*Ell*](http://www.ncbi.nlm.nih.gov/entrez/query.fcgi?db=gene&cmd=Retrieve&dopt=full_report&list_uids=13716) | elongation factor RNA polymerase II | 190 | 92 |
| mmu-miR-322-5p | [*Med26*](http://www.ncbi.nlm.nih.gov/entrez/query.fcgi?db=gene&cmd=Retrieve&dopt=full_report&list_uids=70625) | mediator complex subunit 26 | 191 | 92 |
| mmu-miR-322-5p | [*Sema6d*](http://www.ncbi.nlm.nih.gov/entrez/query.fcgi?db=gene&cmd=Retrieve&dopt=full_report&list_uids=214968) | sema domain, transmembrane domain (TM), and cytoplasmic domain, (semaphorin) 6D | 192 | 92 |
| mmu-miR-322-5p | [*Ghr*](http://www.ncbi.nlm.nih.gov/entrez/query.fcgi?db=gene&cmd=Retrieve&dopt=full_report&list_uids=14600) | growth hormone receptor | 193 | 92 |
| mmu-miR-322-5p | [*Zmat3*](http://www.ncbi.nlm.nih.gov/entrez/query.fcgi?db=gene&cmd=Retrieve&dopt=full_report&list_uids=22401) | zinc finger matrin type 3 | 194 | 91 |
| mmu-miR-322-5p | [*Sgk1*](http://www.ncbi.nlm.nih.gov/entrez/query.fcgi?db=gene&cmd=Retrieve&dopt=full_report&list_uids=20393) | serum/glucocorticoid regulated kinase 1 | 195 | 91 |
| mmu-miR-322-5p | [*Kbtbd2*](http://www.ncbi.nlm.nih.gov/entrez/query.fcgi?db=gene&cmd=Retrieve&dopt=full_report&list_uids=210973) | kelch repeat and BTB (POZ) domain containing 2 | 196 | 91 |
| mmu-miR-322-5p | [*Seh1l*](http://www.ncbi.nlm.nih.gov/entrez/query.fcgi?db=gene&cmd=Retrieve&dopt=full_report&list_uids=72124) | SEH1-like (S. cerevisiae | 197 | 91 |
| mmu-miR-322-5p | [*Zbtb39*](http://www.ncbi.nlm.nih.gov/entrez/query.fcgi?db=gene&cmd=Retrieve&dopt=full_report&list_uids=320080) | zinc finger and BTB domain containing 39 | 198 | 91 |
| mmu-miR-322-5p | [*Arhgap26*](http://www.ncbi.nlm.nih.gov/entrez/query.fcgi?db=gene&cmd=Retrieve&dopt=full_report&list_uids=71302) | Rho GTPase activating protein 26 | 199 | 91 |
| mmu-miR-322-5p | [*Cd2ap*](http://www.ncbi.nlm.nih.gov/entrez/query.fcgi?db=gene&cmd=Retrieve&dopt=full_report&list_uids=12488) | CD2-associated protein | 200 | 91 |
| mmu-miR-322-5p | [*Traf3*](http://www.ncbi.nlm.nih.gov/entrez/query.fcgi?db=gene&cmd=Retrieve&dopt=full_report&list_uids=22031) | TNF receptor-associated factor 3 | 201 | 91 |
| mmu-miR-322-5p | [*Lurap1l*](http://www.ncbi.nlm.nih.gov/entrez/query.fcgi?db=gene&cmd=Retrieve&dopt=full_report&list_uids=52829) | leucine rich adaptor protein 1-like | 202 | 91 |
| mmu-miR-322-5p | [*Tab3*](http://www.ncbi.nlm.nih.gov/entrez/query.fcgi?db=gene&cmd=Retrieve&dopt=full_report&list_uids=66724) | TGF-beta activated kinase 1/MAP3K7 binding protein 3 | 203 | 91 |
| mmu-miR-322-5p | [*Ccdc6*](http://www.ncbi.nlm.nih.gov/entrez/query.fcgi?db=gene&cmd=Retrieve&dopt=full_report&list_uids=76551) | coiled-coil domain containing 6 | 204 | 91 |
| mmu-miR-322-5p | [*Snrk*](http://www.ncbi.nlm.nih.gov/entrez/query.fcgi?db=gene&cmd=Retrieve&dopt=full_report&list_uids=20623) | SNF related kinase | 205 | 91 |
| mmu-miR-322-5p | [*Zpbp*](http://www.ncbi.nlm.nih.gov/entrez/query.fcgi?db=gene&cmd=Retrieve&dopt=full_report&list_uids=53604) | zona pellucida binding protein | 206 | 91 |
| mmu-miR-322-5p | [*Nf1*](http://www.ncbi.nlm.nih.gov/entrez/query.fcgi?db=gene&cmd=Retrieve&dopt=full_report&list_uids=18015) | neurofibromatosis 1 | 207 | 91 |
| mmu-miR-322-5p | [*Cd274*](http://www.ncbi.nlm.nih.gov/entrez/query.fcgi?db=gene&cmd=Retrieve&dopt=full_report&list_uids=60533) | CD274 antigen | 208 | 91 |
| mmu-miR-322-5p | [*Cbl*](http://www.ncbi.nlm.nih.gov/entrez/query.fcgi?db=gene&cmd=Retrieve&dopt=full_report&list_uids=12402) | Casitas B-lineage lymphoma | 209 | 91 |
| mmu-miR-322-5p | [*Lrig1*](http://www.ncbi.nlm.nih.gov/entrez/query.fcgi?db=gene&cmd=Retrieve&dopt=full_report&list_uids=16206) | leucine-rich repeats and immunoglobulin-like domains 1 | 210 | 91 |
| mmu-miR-322-5p | [*Helz*](http://www.ncbi.nlm.nih.gov/entrez/query.fcgi?db=gene&cmd=Retrieve&dopt=full_report&list_uids=78455) | helicase with zinc finger domain | 211 | 91 |
| mmu-miR-322-5p | [*Hmga1-rs1*](http://www.ncbi.nlm.nih.gov/entrez/query.fcgi?db=gene&cmd=Retrieve&dopt=full_report&list_uids=111241) | high mobility group AT-hook I, related sequence 1 | 212 | 90 |
| mmu-miR-322-5p | [*Pdxk*](http://www.ncbi.nlm.nih.gov/entrez/query.fcgi?db=gene&cmd=Retrieve&dopt=full_report&list_uids=216134) | pyridoxal (pyridoxine, vitamin B6) kinase | 213 | 90 |
| mmu-miR-322-5p | [*Atxn2*](http://www.ncbi.nlm.nih.gov/entrez/query.fcgi?db=gene&cmd=Retrieve&dopt=full_report&list_uids=20239) | ataxin 2 | 214 | 90 |
| mmu-miR-322-5p | [*Col4a3bp*](http://www.ncbi.nlm.nih.gov/entrez/query.fcgi?db=gene&cmd=Retrieve&dopt=full_report&list_uids=68018) | collagen, type IV, alpha 3 (Goodpasture antigen) binding protein | 215 | 90 |
| mmu-miR-322-5p | [*Phka1*](http://www.ncbi.nlm.nih.gov/entrez/query.fcgi?db=gene&cmd=Retrieve&dopt=full_report&list_uids=18679) | phosphorylase kinase alpha 1 | 216 | 90 |
| mmu-miR-322-5p | [*Eya1*](http://www.ncbi.nlm.nih.gov/entrez/query.fcgi?db=gene&cmd=Retrieve&dopt=full_report&list_uids=14048) | eyes absent 1 homolog (Drosophila) | 217 | 90 |
| mmu-miR-322-5p | [*Ccny*](http://www.ncbi.nlm.nih.gov/entrez/query.fcgi?db=gene&cmd=Retrieve&dopt=full_report&list_uids=67974) | cyclin Y | 218 | 90 |
| mmu-miR-322-5p | [*Eda*](http://www.ncbi.nlm.nih.gov/entrez/query.fcgi?db=gene&cmd=Retrieve&dopt=full_report&list_uids=13607) | ectodysplasin-A | 219 | 90 |
| mmu-miR-322-5p | [*Cops7b*](http://www.ncbi.nlm.nih.gov/entrez/query.fcgi?db=gene&cmd=Retrieve&dopt=full_report&list_uids=26895) | COP9 (constitutive photomorphogenic) homolog, subunit 7b (Arabidopsis thaliana) | 220 | 90 |
| mmu-miR-322-5p | [*Ubr3*](http://www.ncbi.nlm.nih.gov/entrez/query.fcgi?db=gene&cmd=Retrieve&dopt=full_report&list_uids=68795) | ubiquitin protein ligase E3 component n-recognin 3 | 221 | 90 |
| mmu-miR-322-5p | [*Wnt7a*](http://www.ncbi.nlm.nih.gov/entrez/query.fcgi?db=gene&cmd=Retrieve&dopt=full_report&list_uids=22421) | wingless-related MMTV integration site 7A | 222 | 90 |
| mmu-miR-322-5p | [*Ythdc1*](http://www.ncbi.nlm.nih.gov/entrez/query.fcgi?db=gene&cmd=Retrieve&dopt=full_report&list_uids=231386) | YTH domain containing 1 | 223 | 90 |
| mmu-miR-322-5p | [*Tbl1xr1*](http://www.ncbi.nlm.nih.gov/entrez/query.fcgi?db=gene&cmd=Retrieve&dopt=full_report&list_uids=81004) | transducin (beta)-like 1X-linked receptor 1 | 224 | 90 |
| mmu-miR-322-5p | [*Rnf138*](http://www.ncbi.nlm.nih.gov/entrez/query.fcgi?db=gene&cmd=Retrieve&dopt=full_report&list_uids=56515) | ring finger protein 138 | 225 | 90 |
| mmu-miR-322-5p | [*Chd6*](http://www.ncbi.nlm.nih.gov/entrez/query.fcgi?db=gene&cmd=Retrieve&dopt=full_report&list_uids=71389) | chromodomain helicase DNA binding protein 6 | 226 | 90 |
| mmu-miR-322-5p | [*B4galt1*](http://www.ncbi.nlm.nih.gov/entrez/query.fcgi?db=gene&cmd=Retrieve&dopt=full_report&list_uids=14595) | UDP-Gal:betaGlcNAc beta 1,4- galactosyltransferase, polypeptide 1 | 227 | 90 |
| mmu-miR-322-5p | [*Suco*](http://www.ncbi.nlm.nih.gov/entrez/query.fcgi?db=gene&cmd=Retrieve&dopt=full_report&list_uids=226551) | SUN domain containing ossification factor | 228 | 89 |
| mmu-miR-322-5p | [*Abl2*](http://www.ncbi.nlm.nih.gov/entrez/query.fcgi?db=gene&cmd=Retrieve&dopt=full_report&list_uids=11352) | v-abl Abelson murine leukemia viral oncogene 2 (arg, Abelson-related gene) | 229 | 89 |
| mmu-miR-322-5p | [*Cc2d1b*](http://www.ncbi.nlm.nih.gov/entrez/query.fcgi?db=gene&cmd=Retrieve&dopt=full_report&list_uids=319965) | coiled-coil and C2 domain containing 1B | 230 | 89 |
| mmu-miR-322-5p | [*Il1rapl1*](http://www.ncbi.nlm.nih.gov/entrez/query.fcgi?db=gene&cmd=Retrieve&dopt=full_report&list_uids=331461) | interleukin 1 receptor accessory protein-like 1 | 231 | 89 |
| mmu-miR-322-5p | [*Pbx3*](http://www.ncbi.nlm.nih.gov/entrez/query.fcgi?db=gene&cmd=Retrieve&dopt=full_report&list_uids=18516) | pre B cell leukemia homeobox 3 | 232 | 89 |
| mmu-miR-322-5p | [*Capza2*](http://www.ncbi.nlm.nih.gov/entrez/query.fcgi?db=gene&cmd=Retrieve&dopt=full_report&list_uids=12343) | capping protein (actin filament) muscle Z-line, alpha 2 | 233 | 89 |
| mmu-miR-322-5p | [*Ano3*](http://www.ncbi.nlm.nih.gov/entrez/query.fcgi?db=gene&cmd=Retrieve&dopt=full_report&list_uids=228432) | anoctamin 3 | 234 | 89 |
| mmu-miR-322-5p | [*AI593442*](http://www.ncbi.nlm.nih.gov/entrez/query.fcgi?db=gene&cmd=Retrieve&dopt=full_report&list_uids=330941) | expressed sequence AI593442 | 235 | 89 |
| mmu-miR-322-5p | [*Sez6l*](http://www.ncbi.nlm.nih.gov/entrez/query.fcgi?db=gene&cmd=Retrieve&dopt=full_report&list_uids=56747) | seizure related 6 homolog like | 236 | 89 |
| mmu-miR-322-5p | [*Atg14*](http://www.ncbi.nlm.nih.gov/entrez/query.fcgi?db=gene&cmd=Retrieve&dopt=full_report&list_uids=100504663) | autophagy related 14 | 237 | 89 |
| mmu-miR-322-5p | [*Hsd17b7*](http://www.ncbi.nlm.nih.gov/entrez/query.fcgi?db=gene&cmd=Retrieve&dopt=full_report&list_uids=15490) | hydroxysteroid (17-beta) dehydrogenase 7 | 238 | 89 |
| mmu-miR-322-5p | [*Has3*](http://www.ncbi.nlm.nih.gov/entrez/query.fcgi?db=gene&cmd=Retrieve&dopt=full_report&list_uids=15118) | hyaluronan synthase 3 | 239 | 89 |
| mmu-miR-322-5p | [*Dnajc16*](http://www.ncbi.nlm.nih.gov/entrez/query.fcgi?db=gene&cmd=Retrieve&dopt=full_report&list_uids=214063) | DnaJ (Hsp40) homolog, subfamily C, member 16 | 240 | 89 |
| mmu-miR-322-5p | [*1700021K19Rik*](http://www.ncbi.nlm.nih.gov/entrez/query.fcgi?db=gene&cmd=Retrieve&dopt=full_report&list_uids=100502698) | RIKEN cDNA 1700021K19 gene | 241 | 89 |
| mmu-miR-322-5p | [*Mgat4a*](http://www.ncbi.nlm.nih.gov/entrez/query.fcgi?db=gene&cmd=Retrieve&dopt=full_report&list_uids=269181) | mannoside acetylglucosaminyltransferase 4, isoenzyme A | 242 | 89 |
| mmu-miR-322-5p | [*Flywch1*](http://www.ncbi.nlm.nih.gov/entrez/query.fcgi?db=gene&cmd=Retrieve&dopt=full_report&list_uids=224613) | FLYWCH-type zinc finger 1 | 243 | 89 |
| mmu-miR-322-5p | [*Cd28*](http://www.ncbi.nlm.nih.gov/entrez/query.fcgi?db=gene&cmd=Retrieve&dopt=full_report&list_uids=12487) | CD28 antigen | 244 | 89 |
| mmu-miR-322-5p | [*Znrf2*](http://www.ncbi.nlm.nih.gov/entrez/query.fcgi?db=gene&cmd=Retrieve&dopt=full_report&list_uids=387524) | zinc and ring finger 2 | 245 | 88 |
| mmu-miR-322-5p | [*Plxnc1*](http://www.ncbi.nlm.nih.gov/entrez/query.fcgi?db=gene&cmd=Retrieve&dopt=full_report&list_uids=54712) | plexin C1 | 246 | 88 |
| mmu-miR-322-5p | [*P2ry4*](http://www.ncbi.nlm.nih.gov/entrez/query.fcgi?db=gene&cmd=Retrieve&dopt=full_report&list_uids=57385) | pyrimidinergic receptor P2Y, G-protein coupled, 4 | 247 | 88 |
| mmu-miR-322-5p | [*Cdc14b*](http://www.ncbi.nlm.nih.gov/entrez/query.fcgi?db=gene&cmd=Retrieve&dopt=full_report&list_uids=218294) | CDC14 cell division cycle 14B | 248 | 88 |
| mmu-miR-322-5p | [*Gne*](http://www.ncbi.nlm.nih.gov/entrez/query.fcgi?db=gene&cmd=Retrieve&dopt=full_report&list_uids=50798) | glucosamine (UDP-N-acetyl)-2-epimerase/N-acetylmannosamine kinase | 249 | 88 |
| mmu-miR-322-5p | [*E2f7*](http://www.ncbi.nlm.nih.gov/entrez/query.fcgi?db=gene&cmd=Retrieve&dopt=full_report&list_uids=52679) | E2F transcription factor 7 | 250 | 88 |
| mmu-miR-322-5p | [*Zbtb34*](http://www.ncbi.nlm.nih.gov/entrez/query.fcgi?db=gene&cmd=Retrieve&dopt=full_report&list_uids=241311) | zinc finger and BTB domain containing 34 | 251 | 88 |
| mmu-miR-322-5p | [*Syt4*](http://www.ncbi.nlm.nih.gov/entrez/query.fcgi?db=gene&cmd=Retrieve&dopt=full_report&list_uids=20983) | synaptotagmin IV | 252 | 88 |
| mmu-miR-322-5p | [*Kcnh1*](http://www.ncbi.nlm.nih.gov/entrez/query.fcgi?db=gene&cmd=Retrieve&dopt=full_report&list_uids=16510) | potassium voltage-gated channel, subfamily H (eag-related), member 1 | 253 | 88 |
| mmu-miR-322-5p | [*Slc16a7*](http://www.ncbi.nlm.nih.gov/entrez/query.fcgi?db=gene&cmd=Retrieve&dopt=full_report&list_uids=20503) | solute carrier family 16 (monocarboxylic acid transporters), member 7 | 254 | 88 |
| mmu-miR-322-5p | [*Inpp5j*](http://www.ncbi.nlm.nih.gov/entrez/query.fcgi?db=gene&cmd=Retrieve&dopt=full_report&list_uids=170835) | inositol polyphosphate 5-phosphatase J | 255 | 88 |
| mmu-miR-322-5p | [*Gpr63*](http://www.ncbi.nlm.nih.gov/entrez/query.fcgi?db=gene&cmd=Retrieve&dopt=full_report&list_uids=81006) | G protein-coupled receptor 63 | 256 | 88 |
| mmu-miR-322-5p | [*Rassf4*](http://www.ncbi.nlm.nih.gov/entrez/query.fcgi?db=gene&cmd=Retrieve&dopt=full_report&list_uids=213391) | Ras association (RalGDS/AF-6) domain family member 4 | 257 | 88 |
| mmu-miR-322-5p | [*Vat1*](http://www.ncbi.nlm.nih.gov/entrez/query.fcgi?db=gene&cmd=Retrieve&dopt=full_report&list_uids=26949) | vesicle amine transport protein 1 homolog (T californica) | 258 | 88 |
| mmu-miR-322-5p | [*Scoc*](http://www.ncbi.nlm.nih.gov/entrez/query.fcgi?db=gene&cmd=Retrieve&dopt=full_report&list_uids=56367) | short coiled-coil protein | 259 | 88 |
| mmu-miR-322-5p | [*Mief1*](http://www.ncbi.nlm.nih.gov/entrez/query.fcgi?db=gene&cmd=Retrieve&dopt=full_report&list_uids=239555) | mitochondrial elongation factor 1 | 260 | 88 |
| mmu-miR-322-5p | [*Man2a2*](http://www.ncbi.nlm.nih.gov/entrez/query.fcgi?db=gene&cmd=Retrieve&dopt=full_report&list_uids=140481) | mannosidase 2, alpha 2 | 261 | 88 |
| mmu-miR-322-5p | [*Mob3b*](http://www.ncbi.nlm.nih.gov/entrez/query.fcgi?db=gene&cmd=Retrieve&dopt=full_report&list_uids=214944) | MOB kinase activator 3B | 262 | 88 |
| mmu-miR-322-5p | [*Mybl1*](http://www.ncbi.nlm.nih.gov/entrez/query.fcgi?db=gene&cmd=Retrieve&dopt=full_report&list_uids=17864) | myeloblastosis oncogene-like 1 | 263 | 88 |
| mmu-miR-322-5p | [*Gcnt3*](http://www.ncbi.nlm.nih.gov/entrez/query.fcgi?db=gene&cmd=Retrieve&dopt=full_report&list_uids=72077) | glucosaminyl (N-acetyl) transferase 3, mucin type | 264 | 88 |
| mmu-miR-322-5p | [*Zfp609*](http://www.ncbi.nlm.nih.gov/entrez/query.fcgi?db=gene&cmd=Retrieve&dopt=full_report&list_uids=214812) | zinc finger protein 609 | 265 | 88 |
| mmu-miR-322-5p | [*Gm12886*](http://www.ncbi.nlm.nih.gov/entrez/query.fcgi?db=gene&cmd=Retrieve&dopt=full_report&list_uids=666921) | predicted gene 12886 | 266 | 87 |
| mmu-miR-322-5p | [*Cecr6*](http://www.ncbi.nlm.nih.gov/entrez/query.fcgi?db=gene&cmd=Retrieve&dopt=full_report&list_uids=94047) | cat eye syndrome chromosome region, candidate 6 | 267 | 87 |
| mmu-miR-322-5p | [*Adrb2*](http://www.ncbi.nlm.nih.gov/entrez/query.fcgi?db=gene&cmd=Retrieve&dopt=full_report&list_uids=11555) | adrenergic receptor, beta 2 | 268 | 87 |
| mmu-miR-322-5p | [*Sesn1*](http://www.ncbi.nlm.nih.gov/entrez/query.fcgi?db=gene&cmd=Retrieve&dopt=full_report&list_uids=140742) | sestrin 1 | 269 | 87 |
| mmu-miR-322-5p | [*Eif1a*](http://www.ncbi.nlm.nih.gov/entrez/query.fcgi?db=gene&cmd=Retrieve&dopt=full_report&list_uids=13664) | eukaryotic translation initiation factor 1A | 270 | 87 |
| mmu-miR-322-5p | [*Sidt2*](http://www.ncbi.nlm.nih.gov/entrez/query.fcgi?db=gene&cmd=Retrieve&dopt=full_report&list_uids=214597) | SID1 transmembrane family, member 2 | 271 | 87 |
| mmu-miR-322-5p | [*Bcl2l2*](http://www.ncbi.nlm.nih.gov/entrez/query.fcgi?db=gene&cmd=Retrieve&dopt=full_report&list_uids=12050) | BCL2-like 2 | 272 | 87 |
| mmu-miR-322-5p | [*Tmem33*](http://www.ncbi.nlm.nih.gov/entrez/query.fcgi?db=gene&cmd=Retrieve&dopt=full_report&list_uids=67878) | transmembrane protein 33 | 273 | 87 |
| mmu-miR-322-5p | [*Vegfa*](http://www.ncbi.nlm.nih.gov/entrez/query.fcgi?db=gene&cmd=Retrieve&dopt=full_report&list_uids=22339) | vascular endothelial growth factor A | 274 | 87 |
| mmu-miR-322-5p | [*Igf2r*](http://www.ncbi.nlm.nih.gov/entrez/query.fcgi?db=gene&cmd=Retrieve&dopt=full_report&list_uids=16004) | insulin-like growth factor 2 receptor | 275 | 87 |
| mmu-miR-322-5p | [*Lhx3*](http://www.ncbi.nlm.nih.gov/entrez/query.fcgi?db=gene&cmd=Retrieve&dopt=full_report&list_uids=16871) | LIM homeobox protein 3 | 276 | 87 |
| mmu-miR-322-5p | [*Entpd7*](http://www.ncbi.nlm.nih.gov/entrez/query.fcgi?db=gene&cmd=Retrieve&dopt=full_report&list_uids=93685) | ectonucleoside triphosphate diphosphohydrolase 7 | 277 | 87 |
| mmu-miR-322-5p | [*Chic1*](http://www.ncbi.nlm.nih.gov/entrez/query.fcgi?db=gene&cmd=Retrieve&dopt=full_report&list_uids=12212) | cysteine-rich hydrophobic domain 1 | 278 | 87 |
| mmu-miR-322-5p | [*Dnajc25*](http://www.ncbi.nlm.nih.gov/entrez/query.fcgi?db=gene&cmd=Retrieve&dopt=full_report&list_uids=72429) | DnaJ (Hsp40) homolog, subfamily C, member 25 | 279 | 87 |
| mmu-miR-322-5p | [*Synj1*](http://www.ncbi.nlm.nih.gov/entrez/query.fcgi?db=gene&cmd=Retrieve&dopt=full_report&list_uids=104015) | synaptojanin 1 | 280 | 87 |
| mmu-miR-322-5p | [*Fmn2*](http://www.ncbi.nlm.nih.gov/entrez/query.fcgi?db=gene&cmd=Retrieve&dopt=full_report&list_uids=54418) | formin 2 | 281 | 87 |
| mmu-miR-322-5p | [*Cobll1*](http://www.ncbi.nlm.nih.gov/entrez/query.fcgi?db=gene&cmd=Retrieve&dopt=full_report&list_uids=319876) | Cobl-like 1 | 282 | 87 |
| mmu-miR-322-5p | [*Abcg4*](http://www.ncbi.nlm.nih.gov/entrez/query.fcgi?db=gene&cmd=Retrieve&dopt=full_report&list_uids=192663) | ATP-binding cassette, sub-family G (WHITE), member 4 | 283 | 87 |
| mmu-miR-322-5p | [*Aqp11*](http://www.ncbi.nlm.nih.gov/entrez/query.fcgi?db=gene&cmd=Retrieve&dopt=full_report&list_uids=66333) | aquaporin 11 | 284 | 86 |
| mmu-miR-322-5p | [*Pnpla6*](http://www.ncbi.nlm.nih.gov/entrez/query.fcgi?db=gene&cmd=Retrieve&dopt=full_report&list_uids=50767) | patatin-like phospholipase domain containing 6 | 285 | 86 |
| mmu-miR-322-5p | [*Raph1*](http://www.ncbi.nlm.nih.gov/entrez/query.fcgi?db=gene&cmd=Retrieve&dopt=full_report&list_uids=77300) | Ras association (RalGDS/AF-6) and pleckstrin homology domains 1 | 286 | 86 |
| mmu-miR-322-5p | [*Bag5*](http://www.ncbi.nlm.nih.gov/entrez/query.fcgi?db=gene&cmd=Retrieve&dopt=full_report&list_uids=70369) | BCL2-associated athanogene 5 | 287 | 86 |
| mmu-miR-322-5p | [*Usp49*](http://www.ncbi.nlm.nih.gov/entrez/query.fcgi?db=gene&cmd=Retrieve&dopt=full_report&list_uids=224836) | ubiquitin specific peptidase 49 | 288 | 86 |
| mmu-miR-322-5p | [*Pwwp2b*](http://www.ncbi.nlm.nih.gov/entrez/query.fcgi?db=gene&cmd=Retrieve&dopt=full_report&list_uids=101631) | PWWP domain containing 2B | 289 | 86 |
| mmu-miR-322-5p | [*Wdr92*](http://www.ncbi.nlm.nih.gov/entrez/query.fcgi?db=gene&cmd=Retrieve&dopt=full_report&list_uids=103784) | WD repeat domain 92 | 290 | 86 |
| mmu-miR-322-5p | [*Arhgef37*](http://www.ncbi.nlm.nih.gov/entrez/query.fcgi?db=gene&cmd=Retrieve&dopt=full_report&list_uids=328967) | Rho guanine nucleotide exchange factor (GEF) 37 | 291 | 86 |
| mmu-miR-322-5p | [*Phf20*](http://www.ncbi.nlm.nih.gov/entrez/query.fcgi?db=gene&cmd=Retrieve&dopt=full_report&list_uids=228829) | PHD finger protein 20 | 292 | 86 |
| mmu-miR-322-5p | [*Usp12*](http://www.ncbi.nlm.nih.gov/entrez/query.fcgi?db=gene&cmd=Retrieve&dopt=full_report&list_uids=22217) | ubiquitin specific peptidase 12 | 293 | 86 |
| mmu-miR-322-5p | [*Zdhhc15*](http://www.ncbi.nlm.nih.gov/entrez/query.fcgi?db=gene&cmd=Retrieve&dopt=full_report&list_uids=108672) | zinc finger, DHHC domain containing 15 | 294 | 86 |
| mmu-miR-322-5p | [*Tuba1a*](http://www.ncbi.nlm.nih.gov/entrez/query.fcgi?db=gene&cmd=Retrieve&dopt=full_report&list_uids=22142) | tubulin, alpha 1A | 295 | 86 |
| mmu-miR-322-5p | [*Rs1*](http://www.ncbi.nlm.nih.gov/entrez/query.fcgi?db=gene&cmd=Retrieve&dopt=full_report&list_uids=20147) | retinoschisis (X-linked, juvenile) 1 (human) | 296 | 86 |
| mmu-miR-322-5p | [*Sptbn2*](http://www.ncbi.nlm.nih.gov/entrez/query.fcgi?db=gene&cmd=Retrieve&dopt=full_report&list_uids=20743) | spectrin beta, non-erythrocytic 2 | 297 | 86 |
| mmu-miR-322-5p | [*Syndig1*](http://www.ncbi.nlm.nih.gov/entrez/query.fcgi?db=gene&cmd=Retrieve&dopt=full_report&list_uids=433485) | synapse differentiation inducing 1 | 298 | 86 |
| mmu-miR-322-5p | [*Galnt1*](http://www.ncbi.nlm.nih.gov/entrez/query.fcgi?db=gene&cmd=Retrieve&dopt=full_report&list_uids=14423) | UDP-N-acetyl-alpha-D-galactosamine:polypeptide N-acetylgalactosaminyltransferase 1 | 299 | 86 |
| mmu-miR-322-5p | [*Tmem183a*](http://www.ncbi.nlm.nih.gov/entrez/query.fcgi?db=gene&cmd=Retrieve&dopt=full_report&list_uids=57439) | transmembrane protein 183A | 300 | 86 |
| mmu-miR-322-5p | [*Cask*](http://www.ncbi.nlm.nih.gov/entrez/query.fcgi?db=gene&cmd=Retrieve&dopt=full_report&list_uids=12361) | calcium/calmodulin-dependent serine protein kinase (MAGUK family) | 301 | 86 |
| mmu-miR-322-5p | [*Ppap2a*](http://www.ncbi.nlm.nih.gov/entrez/query.fcgi?db=gene&cmd=Retrieve&dopt=full_report&list_uids=19012) | phosphatidic acid phosphatase type 2A | 302 | 86 |
| mmu-miR-322-5p | [*Nsg1*](http://www.ncbi.nlm.nih.gov/entrez/query.fcgi?db=gene&cmd=Retrieve&dopt=full_report&list_uids=18196) | neuron specific gene family member 1 | 303 | 86 |
| mmu-miR-322-5p | [*Ip6k1*](http://www.ncbi.nlm.nih.gov/entrez/query.fcgi?db=gene&cmd=Retrieve&dopt=full_report&list_uids=27399) | inositol hexaphosphate kinase 1 | 304 | 86 |
| mmu-miR-322-5p | [*Rnf144b*](http://www.ncbi.nlm.nih.gov/entrez/query.fcgi?db=gene&cmd=Retrieve&dopt=full_report&list_uids=218215) | ring finger protein 144B | 305 | 86 |
| mmu-miR-322-5p | [*Cldn12*](http://www.ncbi.nlm.nih.gov/entrez/query.fcgi?db=gene&cmd=Retrieve&dopt=full_report&list_uids=64945) | claudin 12 | 306 | 86 |
| mmu-miR-322-5p | [*Rarb*](http://www.ncbi.nlm.nih.gov/entrez/query.fcgi?db=gene&cmd=Retrieve&dopt=full_report&list_uids=218772) | retinoic acid receptor, beta | 307 | 85 |
| mmu-miR-322-5p | [*Ppm1e*](http://www.ncbi.nlm.nih.gov/entrez/query.fcgi?db=gene&cmd=Retrieve&dopt=full_report&list_uids=320472) | protein phosphatase 1E (PP2C domain containing) | 308 | 85 |
| mmu-miR-322-5p | [*Tle4*](http://www.ncbi.nlm.nih.gov/entrez/query.fcgi?db=gene&cmd=Retrieve&dopt=full_report&list_uids=21888) | transducin-like enhancer of split 4, homolog of Drosophila E(spl) | 309 | 85 |
| mmu-miR-322-5p | [*Tmtc1*](http://www.ncbi.nlm.nih.gov/entrez/query.fcgi?db=gene&cmd=Retrieve&dopt=full_report&list_uids=387314) | transmembrane and tetratricopeptide repeat containing 1 | 310 | 85 |
| mmu-miR-322-5p | [*Hspa4l*](http://www.ncbi.nlm.nih.gov/entrez/query.fcgi?db=gene&cmd=Retrieve&dopt=full_report&list_uids=18415) | heat shock protein 4 like | 311 | 85 |
| mmu-miR-322-5p | [*Pnp2*](http://www.ncbi.nlm.nih.gov/entrez/query.fcgi?db=gene&cmd=Retrieve&dopt=full_report&list_uids=667034) | purine-nucleoside phosphorylase 2 | 312 | 85 |
| mmu-miR-322-5p | [*Plscr4*](http://www.ncbi.nlm.nih.gov/entrez/query.fcgi?db=gene&cmd=Retrieve&dopt=full_report&list_uids=235527) | phospholipid scramblase 4 | 313 | 85 |
| mmu-miR-322-5p | [*Taf5*](http://www.ncbi.nlm.nih.gov/entrez/query.fcgi?db=gene&cmd=Retrieve&dopt=full_report&list_uids=226182) | TAF5 RNA polymerase II, TATA box binding protein (TBP)-associated factor | 314 | 85 |
| mmu-miR-322-5p | [*Ogt*](http://www.ncbi.nlm.nih.gov/entrez/query.fcgi?db=gene&cmd=Retrieve&dopt=full_report&list_uids=108155) | O-linked N-acetylglucosamine (GlcNAc) transferase (UDP-N-acetylglucosamine:polypeptide-N-acetylglucosaminyl transferase) | 315 | 85 |
| mmu-miR-322-5p | [*Wwp1*](http://www.ncbi.nlm.nih.gov/entrez/query.fcgi?db=gene&cmd=Retrieve&dopt=full_report&list_uids=107568) | WW domain containing E3 ubiquitin protein ligase 1 | 316 | 85 |
| mmu-miR-322-5p | [*Mob4*](http://www.ncbi.nlm.nih.gov/entrez/query.fcgi?db=gene&cmd=Retrieve&dopt=full_report&list_uids=19070) | MOB family member 4, phocein | 317 | 85 |
| mmu-miR-322-5p | [*Zmym2*](http://www.ncbi.nlm.nih.gov/entrez/query.fcgi?db=gene&cmd=Retrieve&dopt=full_report&list_uids=76007) | zinc finger, MYM-type 2 | 318 | 85 |
| mmu-miR-322-5p | [*Kcnab1*](http://www.ncbi.nlm.nih.gov/entrez/query.fcgi?db=gene&cmd=Retrieve&dopt=full_report&list_uids=16497) | potassium voltage-gated channel, shaker-related subfamily, beta member 1 | 319 | 85 |
| mmu-miR-322-5p | [*Pax7*](http://www.ncbi.nlm.nih.gov/entrez/query.fcgi?db=gene&cmd=Retrieve&dopt=full_report&list_uids=18509) | paired box 7 | 320 | 85 |
| mmu-miR-322-5p | [*Zfp592*](http://www.ncbi.nlm.nih.gov/entrez/query.fcgi?db=gene&cmd=Retrieve&dopt=full_report&list_uids=233410) | zinc finger protein 592 | 321 | 85 |
| mmu-miR-322-5p | [*Ccr2*](http://www.ncbi.nlm.nih.gov/entrez/query.fcgi?db=gene&cmd=Retrieve&dopt=full_report&list_uids=12772) | chemokine (C-C motif) receptor 2 | 322 | 85 |
| mmu-miR-322-5p | [*Nlrx1*](http://www.ncbi.nlm.nih.gov/entrez/query.fcgi?db=gene&cmd=Retrieve&dopt=full_report&list_uids=270151) | NLR family member X1 | 323 | 85 |
| mmu-miR-322-5p | [*Lyst*](http://www.ncbi.nlm.nih.gov/entrez/query.fcgi?db=gene&cmd=Retrieve&dopt=full_report&list_uids=17101) | lysosomal trafficking regulator | 324 | 85 |
| mmu-miR-322-5p | [*Reln*](http://www.ncbi.nlm.nih.gov/entrez/query.fcgi?db=gene&cmd=Retrieve&dopt=full_report&list_uids=19699) | reelin | 325 | 84 |
| mmu-miR-322-5p | [*Unc80*](http://www.ncbi.nlm.nih.gov/entrez/query.fcgi?db=gene&cmd=Retrieve&dopt=full_report&list_uids=329178) | unc-80 homolog (C. elegans) | 326 | 84 |
| mmu-miR-322-5p | [*Pcmt1*](http://www.ncbi.nlm.nih.gov/entrez/query.fcgi?db=gene&cmd=Retrieve&dopt=full_report&list_uids=18537) | protein-L-isoaspartate (D-aspartate) O-methyltransferase 1 | 327 | 84 |
| mmu-miR-322-5p | [*Ptpn3*](http://www.ncbi.nlm.nih.gov/entrez/query.fcgi?db=gene&cmd=Retrieve&dopt=full_report&list_uids=545622) | protein tyrosine phosphatase, non-receptor type 3 | 328 | 84 |
| mmu-miR-322-5p | [*Camsap1*](http://www.ncbi.nlm.nih.gov/entrez/query.fcgi?db=gene&cmd=Retrieve&dopt=full_report&list_uids=227634) | calmodulin regulated spectrin-associated protein 1 | 329 | 84 |
| mmu-miR-322-5p | [*Nxph1*](http://www.ncbi.nlm.nih.gov/entrez/query.fcgi?db=gene&cmd=Retrieve&dopt=full_report&list_uids=18231) | neurexophilin 1 | 330 | 84 |
| mmu-miR-322-5p | [*Omg*](http://www.ncbi.nlm.nih.gov/entrez/query.fcgi?db=gene&cmd=Retrieve&dopt=full_report&list_uids=18377) | oligodendrocyte myelin glycoprotein | 331 | 84 |
| mmu-miR-322-5p | [*Klc4*](http://www.ncbi.nlm.nih.gov/entrez/query.fcgi?db=gene&cmd=Retrieve&dopt=full_report&list_uids=74764) | kinesin light chain 4 | 332 | 84 |
| mmu-miR-322-5p | [*Gga3*](http://www.ncbi.nlm.nih.gov/entrez/query.fcgi?db=gene&cmd=Retrieve&dopt=full_report&list_uids=260302) | golgi associated, gamma adaptin ear containing, ARF binding protein 3 | 333 | 84 |
| mmu-miR-322-5p | [*Sema3d*](http://www.ncbi.nlm.nih.gov/entrez/query.fcgi?db=gene&cmd=Retrieve&dopt=full_report&list_uids=108151) | sema domain, immunoglobulin domain (Ig), short basic domain, secreted, (semaphorin) 3D | 334 | 84 |
| mmu-miR-322-5p | [*Otud6b*](http://www.ncbi.nlm.nih.gov/entrez/query.fcgi?db=gene&cmd=Retrieve&dopt=full_report&list_uids=72201) | OTU domain containing 6B | 335 | 84 |
| mmu-miR-322-5p | [*Slc4a8*](http://www.ncbi.nlm.nih.gov/entrez/query.fcgi?db=gene&cmd=Retrieve&dopt=full_report&list_uids=59033) | solute carrier family 4 (anion exchanger), member 8 | 336 | 84 |
| mmu-miR-322-5p | [*Tenm2*](http://www.ncbi.nlm.nih.gov/entrez/query.fcgi?db=gene&cmd=Retrieve&dopt=full_report&list_uids=23964) | teneurin transmembrane protein 2 | 337 | 84 |
| mmu-miR-322-5p | [*Ncapg2*](http://www.ncbi.nlm.nih.gov/entrez/query.fcgi?db=gene&cmd=Retrieve&dopt=full_report&list_uids=76044) | non-SMC condensin II complex, subunit G2 | 338 | 84 |
| mmu-miR-322-5p | [*Atp6v1a*](http://www.ncbi.nlm.nih.gov/entrez/query.fcgi?db=gene&cmd=Retrieve&dopt=full_report&list_uids=11964) | ATPase, H+ transporting, lysosomal V1 subunit A | 339 | 84 |
| mmu-miR-322-5p | [*Slc9a6*](http://www.ncbi.nlm.nih.gov/entrez/query.fcgi?db=gene&cmd=Retrieve&dopt=full_report&list_uids=236794) | solute carrier family 9 (sodium/hydrogen exchanger), member 6 | 340 | 84 |
| mmu-miR-322-5p | [*Cdc42se2*](http://www.ncbi.nlm.nih.gov/entrez/query.fcgi?db=gene&cmd=Retrieve&dopt=full_report&list_uids=72729) | CDC42 small effector 2 | 341 | 84 |
| mmu-miR-322-5p | [*Pcdh9*](http://www.ncbi.nlm.nih.gov/entrez/query.fcgi?db=gene&cmd=Retrieve&dopt=full_report&list_uids=211712) | protocadherin 9 | 342 | 83 |
| mmu-miR-322-5p | [*Idh3a*](http://www.ncbi.nlm.nih.gov/entrez/query.fcgi?db=gene&cmd=Retrieve&dopt=full_report&list_uids=67834) | isocitrate dehydrogenase 3 (NAD+) alpha | 343 | 83 |
| mmu-miR-322-5p | [*Dmtn*](http://www.ncbi.nlm.nih.gov/entrez/query.fcgi?db=gene&cmd=Retrieve&dopt=full_report&list_uids=13829) | dematin actin binding protein | 344 | 83 |
| mmu-miR-322-5p | [*Plxna4*](http://www.ncbi.nlm.nih.gov/entrez/query.fcgi?db=gene&cmd=Retrieve&dopt=full_report&list_uids=243743) | plexin A4 | 345 | 83 |
| mmu-miR-322-5p | [*Arl3*](http://www.ncbi.nlm.nih.gov/entrez/query.fcgi?db=gene&cmd=Retrieve&dopt=full_report&list_uids=56350) | ADP-ribosylation factor-like 3 | 346 | 83 |
| mmu-miR-322-5p | [*Rsbn1*](http://www.ncbi.nlm.nih.gov/entrez/query.fcgi?db=gene&cmd=Retrieve&dopt=full_report&list_uids=229675) | rosbin, round spermatid basic protein 1 | 347 | 83 |
| mmu-miR-322-5p | [*Itgb8*](http://www.ncbi.nlm.nih.gov/entrez/query.fcgi?db=gene&cmd=Retrieve&dopt=full_report&list_uids=320910) | integrin beta 8 | 348 | 83 |
| mmu-miR-322-5p | [*Erc1*](http://www.ncbi.nlm.nih.gov/entrez/query.fcgi?db=gene&cmd=Retrieve&dopt=full_report&list_uids=111173) | ELKS/RAB6-interacting/CAST family member 1 | 349 | 83 |
| mmu-miR-322-5p | [*Dyrk1b*](http://www.ncbi.nlm.nih.gov/entrez/query.fcgi?db=gene&cmd=Retrieve&dopt=full_report&list_uids=13549) | dual-specificity tyrosine-(Y)-phosphorylation regulated kinase 1b | 350 | 83 |
| mmu-miR-322-5p | [*Peli3*](http://www.ncbi.nlm.nih.gov/entrez/query.fcgi?db=gene&cmd=Retrieve&dopt=full_report&list_uids=240518) | pellino 3 | 351 | 83 |
| mmu-miR-322-5p | [*Nuak2*](http://www.ncbi.nlm.nih.gov/entrez/query.fcgi?db=gene&cmd=Retrieve&dopt=full_report&list_uids=74137) | NUAK family, SNF1-like kinase, 2 | 352 | 83 |
| mmu-miR-322-5p | [*Shoc2*](http://www.ncbi.nlm.nih.gov/entrez/query.fcgi?db=gene&cmd=Retrieve&dopt=full_report&list_uids=56392) | soc-2 (suppressor of clear) homolog (C. elegans) | 353 | 83 |
| mmu-miR-322-5p | [*Espn*](http://www.ncbi.nlm.nih.gov/entrez/query.fcgi?db=gene&cmd=Retrieve&dopt=full_report&list_uids=56226) | espin | 354 | 83 |
| mmu-miR-322-5p | [*Il31ra*](http://www.ncbi.nlm.nih.gov/entrez/query.fcgi?db=gene&cmd=Retrieve&dopt=full_report&list_uids=218624) | interleukin 31 receptor A | 355 | 83 |
| mmu-miR-322-5p | [*Rabl3*](http://www.ncbi.nlm.nih.gov/entrez/query.fcgi?db=gene&cmd=Retrieve&dopt=full_report&list_uids=67657) | RAB, member of RAS oncogene family-like 3 | 356 | 83 |
| mmu-miR-322-5p | [*Prkca*](http://www.ncbi.nlm.nih.gov/entrez/query.fcgi?db=gene&cmd=Retrieve&dopt=full_report&list_uids=18750) | protein kinase C, alpha | 357 | 83 |
| mmu-miR-322-5p | [*Tmem135*](http://www.ncbi.nlm.nih.gov/entrez/query.fcgi?db=gene&cmd=Retrieve&dopt=full_report&list_uids=72759) | transmembrane protein 135 | 358 | 83 |
| mmu-miR-322-5p | [*2010012O05Rik*](http://www.ncbi.nlm.nih.gov/entrez/query.fcgi?db=gene&cmd=Retrieve&dopt=full_report&list_uids=66439) | RIKEN cDNA 2010012O05 gene | 359 | 83 |
| mmu-miR-322-5p | [*Csde1*](http://www.ncbi.nlm.nih.gov/entrez/query.fcgi?db=gene&cmd=Retrieve&dopt=full_report&list_uids=229663) | cold shock domain containing E1, RNA binding | 360 | 83 |
| mmu-miR-322-5p | [*Pura*](http://www.ncbi.nlm.nih.gov/entrez/query.fcgi?db=gene&cmd=Retrieve&dopt=full_report&list_uids=19290) | purine rich element binding protein A | 361 | 83 |
| mmu-miR-322-5p | [*Smad5*](http://www.ncbi.nlm.nih.gov/entrez/query.fcgi?db=gene&cmd=Retrieve&dopt=full_report&list_uids=17129) | SMAD family member 5 | 362 | 83 |
| mmu-miR-322-5p | [*9930012K11Rik*](http://www.ncbi.nlm.nih.gov/entrez/query.fcgi?db=gene&cmd=Retrieve&dopt=full_report&list_uids=268759) | RIKEN cDNA 9930012K11 gene | 363 | 83 |
| mmu-miR-322-5p | [*Ogfod3*](http://www.ncbi.nlm.nih.gov/entrez/query.fcgi?db=gene&cmd=Retrieve&dopt=full_report&list_uids=66179) | 2-oxoglutarate and iron-dependent oxygenase domain containing 3 | 364 | 83 |
| mmu-miR-322-5p | [*Ccdc19*](http://www.ncbi.nlm.nih.gov/entrez/query.fcgi?db=gene&cmd=Retrieve&dopt=full_report&list_uids=71870) | coiled-coil domain containing 19 | 365 | 83 |
| mmu-miR-322-5p | [*1810013L24Rik*](http://www.ncbi.nlm.nih.gov/entrez/query.fcgi?db=gene&cmd=Retrieve&dopt=full_report&list_uids=69053) | RIKEN cDNA 1810013L24 gene | 366 | 83 |
| mmu-miR-322-5p | [*Vamp7*](http://www.ncbi.nlm.nih.gov/entrez/query.fcgi?db=gene&cmd=Retrieve&dopt=full_report&list_uids=20955) | vesicle-associated membrane protein 7 | 367 | 83 |
| mmu-miR-322-5p | [*Frmpd1*](http://www.ncbi.nlm.nih.gov/entrez/query.fcgi?db=gene&cmd=Retrieve&dopt=full_report&list_uids=666060) | FERM and PDZ domain containing 1 | 368 | 83 |
| mmu-miR-322-5p | [*Tk2*](http://www.ncbi.nlm.nih.gov/entrez/query.fcgi?db=gene&cmd=Retrieve&dopt=full_report&list_uids=57813) | thymidine kinase 2, mitochondrial | 369 | 82 |
| mmu-miR-322-5p | [*Jade2*](http://www.ncbi.nlm.nih.gov/entrez/query.fcgi?db=gene&cmd=Retrieve&dopt=full_report&list_uids=76901) | jade family PHD finger 2 | 370 | 82 |
| mmu-miR-322-5p | [*Dsel*](http://www.ncbi.nlm.nih.gov/entrez/query.fcgi?db=gene&cmd=Retrieve&dopt=full_report&list_uids=319901) | dermatan sulfate epimerase-like | 371 | 82 |
| mmu-miR-322-5p | [*Hoxa10*](http://www.ncbi.nlm.nih.gov/entrez/query.fcgi?db=gene&cmd=Retrieve&dopt=full_report&list_uids=15395) | homeobox A10 | 372 | 82 |
| mmu-miR-322-5p | [*Tatdn3*](http://www.ncbi.nlm.nih.gov/entrez/query.fcgi?db=gene&cmd=Retrieve&dopt=full_report&list_uids=68972) | TatD DNase domain containing 3 | 373 | 82 |
| mmu-miR-322-5p | [*Tet3*](http://www.ncbi.nlm.nih.gov/entrez/query.fcgi?db=gene&cmd=Retrieve&dopt=full_report&list_uids=194388) | tet methylcytosine dioxygenase 3 | 374 | 82 |
| mmu-miR-322-5p | [*Hoxa3*](http://www.ncbi.nlm.nih.gov/entrez/query.fcgi?db=gene&cmd=Retrieve&dopt=full_report&list_uids=15400) | homeobox A3 | 375 | 82 |
| mmu-miR-322-5p | [*Mtmr4*](http://www.ncbi.nlm.nih.gov/entrez/query.fcgi?db=gene&cmd=Retrieve&dopt=full_report&list_uids=170749) | myotubularin related protein 4 | 376 | 82 |
| mmu-miR-322-5p | [*Klhl18*](http://www.ncbi.nlm.nih.gov/entrez/query.fcgi?db=gene&cmd=Retrieve&dopt=full_report&list_uids=270201) | kelch-like 18 | 377 | 82 |
| mmu-miR-322-5p | [*LOC102643142*](http://www.ncbi.nlm.nih.gov/entrez/query.fcgi?db=gene&cmd=Retrieve&dopt=full_report&list_uids=102643142) | mucin-3A-like | 378 | 82 |
| mmu-miR-322-5p | [*Atp13a3*](http://www.ncbi.nlm.nih.gov/entrez/query.fcgi?db=gene&cmd=Retrieve&dopt=full_report&list_uids=224088) | ATPase type 13A3 | 379 | 82 |
| mmu-miR-322-5p | [*Rspo3*](http://www.ncbi.nlm.nih.gov/entrez/query.fcgi?db=gene&cmd=Retrieve&dopt=full_report&list_uids=72780) | R-spondin 3 homolog (Xenopus laevis) | 380 | 82 |
| mmu-miR-322-5p | [*Lamc1*](http://www.ncbi.nlm.nih.gov/entrez/query.fcgi?db=gene&cmd=Retrieve&dopt=full_report&list_uids=226519) | laminin, gamma 1 | 381 | 82 |
| mmu-miR-322-5p | [*Pnoc*](http://www.ncbi.nlm.nih.gov/entrez/query.fcgi?db=gene&cmd=Retrieve&dopt=full_report&list_uids=18155) | prepronociceptin | 382 | 81 |
| mmu-miR-322-5p | [*Zhx1*](http://www.ncbi.nlm.nih.gov/entrez/query.fcgi?db=gene&cmd=Retrieve&dopt=full_report&list_uids=22770) | zinc fingers and homeoboxes 1 | 383 | 81 |
| mmu-miR-322-5p | [*Rnf217*](http://www.ncbi.nlm.nih.gov/entrez/query.fcgi?db=gene&cmd=Retrieve&dopt=full_report&list_uids=268291) | ring finger protein 217 | 384 | 81 |
| mmu-miR-322-5p | [*Tnfaip8l3*](http://www.ncbi.nlm.nih.gov/entrez/query.fcgi?db=gene&cmd=Retrieve&dopt=full_report&list_uids=244882) | tumor necrosis factor, alpha-induced protein 8-like 3 | 385 | 81 |
| mmu-miR-322-5p | [*Sik1*](http://www.ncbi.nlm.nih.gov/entrez/query.fcgi?db=gene&cmd=Retrieve&dopt=full_report&list_uids=17691) | salt inducible kinase 1 | 386 | 81 |
| mmu-miR-322-5p | [*Lrig2*](http://www.ncbi.nlm.nih.gov/entrez/query.fcgi?db=gene&cmd=Retrieve&dopt=full_report&list_uids=269473) | leucine-rich repeats and immunoglobulin-like domains 2 | 387 | 81 |
| mmu-miR-322-5p | [*Atp2b2*](http://www.ncbi.nlm.nih.gov/entrez/query.fcgi?db=gene&cmd=Retrieve&dopt=full_report&list_uids=11941) | ATPase, Ca++ transporting, plasma membrane 2 | 388 | 81 |
| mmu-miR-322-5p | [*Cacnb2*](http://www.ncbi.nlm.nih.gov/entrez/query.fcgi?db=gene&cmd=Retrieve&dopt=full_report&list_uids=12296) | calcium channel, voltage-dependent, beta 2 subunit | 389 | 81 |
| mmu-miR-322-5p | [*Map2k1*](http://www.ncbi.nlm.nih.gov/entrez/query.fcgi?db=gene&cmd=Retrieve&dopt=full_report&list_uids=26395) | mitogen-activated protein kinase kinase 1 | 390 | 81 |
| mmu-miR-322-5p | [*Akap7*](http://www.ncbi.nlm.nih.gov/entrez/query.fcgi?db=gene&cmd=Retrieve&dopt=full_report&list_uids=432442) | A kinase (PRKA) anchor protein 7 | 391 | 81 |
| mmu-miR-322-5p | [*Siah1a*](http://www.ncbi.nlm.nih.gov/entrez/query.fcgi?db=gene&cmd=Retrieve&dopt=full_report&list_uids=20437) | seven in absentia 1A | 392 | 81 |
| mmu-miR-322-5p | [*Adh5*](http://www.ncbi.nlm.nih.gov/entrez/query.fcgi?db=gene&cmd=Retrieve&dopt=full_report&list_uids=11532) | alcohol dehydrogenase 5 (class III), chi polypeptide | 393 | 80 |
| mmu-miR-322-5p | [*Fktn*](http://www.ncbi.nlm.nih.gov/entrez/query.fcgi?db=gene&cmd=Retrieve&dopt=full_report&list_uids=246179) | fukutin | 394 | 80 |
| mmu-miR-322-5p | [*Lrrc15*](http://www.ncbi.nlm.nih.gov/entrez/query.fcgi?db=gene&cmd=Retrieve&dopt=full_report&list_uids=74488) | leucine rich repeat containing 15 | 395 | 80 |
| mmu-miR-322-5p | [*6430571L13Rik*](http://www.ncbi.nlm.nih.gov/entrez/query.fcgi?db=gene&cmd=Retrieve&dopt=full_report&list_uids=235599) | RIKEN cDNA 6430571L13 gene | 396 | 80 |
| mmu-miR-322-5p | [*Prrc2c*](http://www.ncbi.nlm.nih.gov/entrez/query.fcgi?db=gene&cmd=Retrieve&dopt=full_report&list_uids=226562) | proline-rich coiled-coil 2C | 397 | 80 |
| mmu-miR-322-5p | [*Mettl1*](http://www.ncbi.nlm.nih.gov/entrez/query.fcgi?db=gene&cmd=Retrieve&dopt=full_report&list_uids=17299) | methyltransferase like 1 | 398 | 80 |
| mmu-miR-322-5p | [*Faf2*](http://www.ncbi.nlm.nih.gov/entrez/query.fcgi?db=gene&cmd=Retrieve&dopt=full_report&list_uids=76577) | Fas associated factor family member 2 | 399 | 80 |
| mmu-miR-322-5p | [*Dzip3*](http://www.ncbi.nlm.nih.gov/entrez/query.fcgi?db=gene&cmd=Retrieve&dopt=full_report&list_uids=224170) | DAZ interacting protein 3, zinc finger | 400 | 80 |
| mmu-miR-322-5p | [*Pfas*](http://www.ncbi.nlm.nih.gov/entrez/query.fcgi?db=gene&cmd=Retrieve&dopt=full_report&list_uids=237823) | phosphoribosylformylglycinamidine synthase (FGAR amidotransferase) | 401 | 80 |
| mmu-miR-322-5p | [*Cttnbp2nl*](http://www.ncbi.nlm.nih.gov/entrez/query.fcgi?db=gene&cmd=Retrieve&dopt=full_report&list_uids=80281) | CTTNBP2 N-terminal like | 402 | 80 |
| mmu-miR-322-5p | [*Krtap26-1*](http://www.ncbi.nlm.nih.gov/entrez/query.fcgi?db=gene&cmd=Retrieve&dopt=full_report&list_uids=69533) | keratin associated protein 26-1 | 403 | 80 |
| mmu-miR-322-5p | [*Tmem74b*](http://www.ncbi.nlm.nih.gov/entrez/query.fcgi?db=gene&cmd=Retrieve&dopt=full_report&list_uids=108832) | transmembrane protein 74B | 404 | 80 |
| mmu-miR-322-5p | [*1200014J11Rik*](http://www.ncbi.nlm.nih.gov/entrez/query.fcgi?db=gene&cmd=Retrieve&dopt=full_report&list_uids=66874) | RIKEN cDNA 1200014J11 gene | 405 | 80 |
| mmu-miR-322-5p | [*Nudt4*](http://www.ncbi.nlm.nih.gov/entrez/query.fcgi?db=gene&cmd=Retrieve&dopt=full_report&list_uids=71207) | nudix (nucleoside diphosphate linked moiety X)-type motif 4 | 406 | 80 |
| mmu-miR-322-5p | [*Tmem45b*](http://www.ncbi.nlm.nih.gov/entrez/query.fcgi?db=gene&cmd=Retrieve&dopt=full_report&list_uids=235135) | transmembrane protein 45b | 407 | 80 |
| mmu-miR-322-5p | [*Krtap11-1*](http://www.ncbi.nlm.nih.gov/entrez/query.fcgi?db=gene&cmd=Retrieve&dopt=full_report&list_uids=16693) | keratin associated protein 11-1 | 408 | 80 |
| mmu-miR-322-5p | [*Dvl1*](http://www.ncbi.nlm.nih.gov/entrez/query.fcgi?db=gene&cmd=Retrieve&dopt=full_report&list_uids=13542) | dishevelled, dsh homolog 1 (Drosophila) | 409 | 80 |
| mmu-miR-322-5p | [*Ttll5*](http://www.ncbi.nlm.nih.gov/entrez/query.fcgi?db=gene&cmd=Retrieve&dopt=full_report&list_uids=320244) | tubulin tyrosine ligase-like family, member 5 | 410 | 80 |
| mmu-miR-322-5p | [*Uspl1*](http://www.ncbi.nlm.nih.gov/entrez/query.fcgi?db=gene&cmd=Retrieve&dopt=full_report&list_uids=231915) | ubiquitin specific peptidase like 1 | 411 | 80 |
| mmu-miR-322-5p | [*Stox2*](http://www.ncbi.nlm.nih.gov/entrez/query.fcgi?db=gene&cmd=Retrieve&dopt=full_report&list_uids=71069) | storkhead box 2 | 412 | 80 |
| mmu-miR-322-5p | [*Iars*](http://www.ncbi.nlm.nih.gov/entrez/query.fcgi?db=gene&cmd=Retrieve&dopt=full_report&list_uids=105148) | isoleucine-tRNA synthetase | 413 | 80 |
| mmu-miR-322-5p | [*Kctd1*](http://www.ncbi.nlm.nih.gov/entrez/query.fcgi?db=gene&cmd=Retrieve&dopt=full_report&list_uids=106931) | potassium channel tetramerisation domain containing 1 | 414 | 80 |
| mmu-miR-322-5p | [*Ptprr*](http://www.ncbi.nlm.nih.gov/entrez/query.fcgi?db=gene&cmd=Retrieve&dopt=full_report&list_uids=19279) | protein tyrosine phosphatase, receptor type, R | 415 | 80 |
| mmu-miR-322-5p | [*Epha1*](http://www.ncbi.nlm.nih.gov/entrez/query.fcgi?db=gene&cmd=Retrieve&dopt=full_report&list_uids=13835) | Eph receptor A1 | 416 | 80 |
| mmu-miR-351-5p | [*Fam83h*](http://www.ncbi.nlm.nih.gov/entrez/query.fcgi?db=gene&cmd=Retrieve&dopt=full_report&list_uids=105732) | family with sequence similarity 83, member H | 1 | 99 |
| mmu-miR-351-5p | [*Kcnk10*](http://www.ncbi.nlm.nih.gov/entrez/query.fcgi?db=gene&cmd=Retrieve&dopt=full_report&list_uids=72258) | potassium channel, subfamily K, member 10 | 2 | 99 |
| mmu-miR-351-5p | [*Zswim6*](http://www.ncbi.nlm.nih.gov/entrez/query.fcgi?db=gene&cmd=Retrieve&dopt=full_report&list_uids=67263) | zinc finger SWIM-type containing 6 | 3 | 99 |
| mmu-miR-351-5p | [*Abhd6*](http://www.ncbi.nlm.nih.gov/entrez/query.fcgi?db=gene&cmd=Retrieve&dopt=full_report&list_uids=66082) | abhydrolase domain containing 6 | 4 | 99 |
| mmu-miR-351-5p | [*Enpep*](http://www.ncbi.nlm.nih.gov/entrez/query.fcgi?db=gene&cmd=Retrieve&dopt=full_report&list_uids=13809) | glutamyl aminopeptidase | 5 | 99 |
| mmu-miR-351-5p | [*Bmf*](http://www.ncbi.nlm.nih.gov/entrez/query.fcgi?db=gene&cmd=Retrieve&dopt=full_report&list_uids=171543) | BCL2 modifying factor | 6 | 99 |
| mmu-miR-351-5p | [*Slc39a9*](http://www.ncbi.nlm.nih.gov/entrez/query.fcgi?db=gene&cmd=Retrieve&dopt=full_report&list_uids=328133) | solute carrier family 39 (zinc transporter), member 9 | 7 | 99 |
| mmu-miR-351-5p | [*Ovol1*](http://www.ncbi.nlm.nih.gov/entrez/query.fcgi?db=gene&cmd=Retrieve&dopt=full_report&list_uids=18426) | OVO homolog-like 1 (Drosophila) | 8 | 99 |
| mmu-miR-351-5p | [*Osbpl9*](http://www.ncbi.nlm.nih.gov/entrez/query.fcgi?db=gene&cmd=Retrieve&dopt=full_report&list_uids=100273) | oxysterol binding protein-like 9 | 9 | 99 |
| mmu-miR-351-5p | [*Npl*](http://www.ncbi.nlm.nih.gov/entrez/query.fcgi?db=gene&cmd=Retrieve&dopt=full_report&list_uids=74091) | N-acetylneuraminate pyruvate lyase | 10 | 98 |
| mmu-miR-351-5p | [*Rapgef5*](http://www.ncbi.nlm.nih.gov/entrez/query.fcgi?db=gene&cmd=Retrieve&dopt=full_report&list_uids=217944) | Rap guanine nucleotide exchange factor (GEF) 5 | 11 | 98 |
| mmu-miR-351-5p | [*Sgpl1*](http://www.ncbi.nlm.nih.gov/entrez/query.fcgi?db=gene&cmd=Retrieve&dopt=full_report&list_uids=20397) | sphingosine phosphate lyase 1 | 12 | 98 |
| mmu-miR-351-5p | [*Irf4*](http://www.ncbi.nlm.nih.gov/entrez/query.fcgi?db=gene&cmd=Retrieve&dopt=full_report&list_uids=16364) | interferon regulatory factor 4 | 13 | 98 |
| mmu-miR-351-5p | [*Eif1ad*](http://www.ncbi.nlm.nih.gov/entrez/query.fcgi?db=gene&cmd=Retrieve&dopt=full_report&list_uids=69860) | eukaryotic translation initiation factor 1A domain containing | 14 | 98 |
| mmu-miR-351-5p | [*Nrm*](http://www.ncbi.nlm.nih.gov/entrez/query.fcgi?db=gene&cmd=Retrieve&dopt=full_report&list_uids=106582) | nurim (nuclear envelope membrane protein) | 15 | 98 |
| mmu-miR-351-5p | [*Necab3*](http://www.ncbi.nlm.nih.gov/entrez/query.fcgi?db=gene&cmd=Retrieve&dopt=full_report&list_uids=56846) | N-terminal EF-hand calcium binding protein 3 | 16 | 98 |
| mmu-miR-351-5p | [*Ptpn1*](http://www.ncbi.nlm.nih.gov/entrez/query.fcgi?db=gene&cmd=Retrieve&dopt=full_report&list_uids=19246) | protein tyrosine phosphatase, non-receptor type 1 | 17 | 97 |
| mmu-miR-351-5p | [*Slc43a2*](http://www.ncbi.nlm.nih.gov/entrez/query.fcgi?db=gene&cmd=Retrieve&dopt=full_report&list_uids=215113) | solute carrier family 43, member 2 | 18 | 97 |
| mmu-miR-351-5p | [*Smek1*](http://www.ncbi.nlm.nih.gov/entrez/query.fcgi?db=gene&cmd=Retrieve&dopt=full_report&list_uids=68734) | SMEK homolog 1, suppressor of mek1 (Dictyostelium) | 19 | 97 |
| mmu-miR-351-5p | [*Scn2b*](http://www.ncbi.nlm.nih.gov/entrez/query.fcgi?db=gene&cmd=Retrieve&dopt=full_report&list_uids=72821) | sodium channel, voltage-gated, type II, beta | 20 | 97 |
| mmu-miR-351-5p | [*Casp2*](http://www.ncbi.nlm.nih.gov/entrez/query.fcgi?db=gene&cmd=Retrieve&dopt=full_report&list_uids=12366) | caspase 2 | 21 | 97 |
| mmu-miR-351-5p | [*Slc35a4*](http://www.ncbi.nlm.nih.gov/entrez/query.fcgi?db=gene&cmd=Retrieve&dopt=full_report&list_uids=67843) | solute carrier family 35, member A4 | 22 | 97 |
| mmu-miR-351-5p | [*4632428N05Rik*](http://www.ncbi.nlm.nih.gov/entrez/query.fcgi?db=gene&cmd=Retrieve&dopt=full_report&list_uids=74048) | RIKEN cDNA 4632428N05 gene | 23 | 97 |
| mmu-miR-351-5p | [*Cgn*](http://www.ncbi.nlm.nih.gov/entrez/query.fcgi?db=gene&cmd=Retrieve&dopt=full_report&list_uids=70737) | cingulin | 24 | 97 |
| mmu-miR-351-5p | [*Dynlt3*](http://www.ncbi.nlm.nih.gov/entrez/query.fcgi?db=gene&cmd=Retrieve&dopt=full_report&list_uids=67117) | dynein light chain Tctex-type 3 | 25 | 97 |
| mmu-miR-351-5p | [*Grsf1*](http://www.ncbi.nlm.nih.gov/entrez/query.fcgi?db=gene&cmd=Retrieve&dopt=full_report&list_uids=231413) | G-rich RNA sequence binding factor 1 | 26 | 97 |
| mmu-miR-351-5p | [*Kcns3*](http://www.ncbi.nlm.nih.gov/entrez/query.fcgi?db=gene&cmd=Retrieve&dopt=full_report&list_uids=238076) | potassium voltage-gated channel, delayed-rectifier, subfamily S, member 3 | 27 | 97 |
| mmu-miR-351-5p | [*Nek10*](http://www.ncbi.nlm.nih.gov/entrez/query.fcgi?db=gene&cmd=Retrieve&dopt=full_report&list_uids=674895) | NIMA (never in mitosis gene a)- related kinase 10 | 28 | 97 |
| mmu-miR-351-5p | [*Hif1an*](http://www.ncbi.nlm.nih.gov/entrez/query.fcgi?db=gene&cmd=Retrieve&dopt=full_report&list_uids=319594) | hypoxia-inducible factor 1, alpha subunit inhibitor | 29 | 97 |
| mmu-miR-351-5p | [*Zswim5*](http://www.ncbi.nlm.nih.gov/entrez/query.fcgi?db=gene&cmd=Retrieve&dopt=full_report&list_uids=74464) | zinc finger SWIM-type containing 5 | 30 | 96 |
| mmu-miR-351-5p | [*Samd10*](http://www.ncbi.nlm.nih.gov/entrez/query.fcgi?db=gene&cmd=Retrieve&dopt=full_report&list_uids=229011) | sterile alpha motif domain containing 10 | 31 | 96 |
| mmu-miR-351-5p | [*Slc25a35*](http://www.ncbi.nlm.nih.gov/entrez/query.fcgi?db=gene&cmd=Retrieve&dopt=full_report&list_uids=71998) | solute carrier family 25, member 35 | 32 | 96 |
| mmu-miR-351-5p | [*Pgpep1*](http://www.ncbi.nlm.nih.gov/entrez/query.fcgi?db=gene&cmd=Retrieve&dopt=full_report&list_uids=66522) | pyroglutamyl-peptidase I | 33 | 96 |
| mmu-miR-351-5p | [*Crb2*](http://www.ncbi.nlm.nih.gov/entrez/query.fcgi?db=gene&cmd=Retrieve&dopt=full_report&list_uids=241324) | crumbs homolog 2 (Drosophila) | 34 | 96 |
| mmu-miR-351-5p | [*Sec14l2*](http://www.ncbi.nlm.nih.gov/entrez/query.fcgi?db=gene&cmd=Retrieve&dopt=full_report&list_uids=67815) | SEC14-like 2 (S. cerevisiae) | 35 | 96 |
| mmu-miR-351-5p | [*Cyyr1*](http://www.ncbi.nlm.nih.gov/entrez/query.fcgi?db=gene&cmd=Retrieve&dopt=full_report&list_uids=224405) | cysteine and tyrosine-rich protein 1 | 36 | 96 |
| mmu-miR-351-5p | [*Myo9a*](http://www.ncbi.nlm.nih.gov/entrez/query.fcgi?db=gene&cmd=Retrieve&dopt=full_report&list_uids=270163) | myosin IXa | 37 | 95 |
| mmu-miR-351-5p | [*Zfyve1*](http://www.ncbi.nlm.nih.gov/entrez/query.fcgi?db=gene&cmd=Retrieve&dopt=full_report&list_uids=217695) | zinc finger, FYVE domain containing 1 | 38 | 95 |
| mmu-miR-351-5p | [*Sema4d*](http://www.ncbi.nlm.nih.gov/entrez/query.fcgi?db=gene&cmd=Retrieve&dopt=full_report&list_uids=20354) | sema domain, immunoglobulin domain (Ig), transmembrane domain (TM) and short cytoplasmic domain, (semaphorin) 4D | 39 | 95 |
| mmu-miR-351-5p | [*Sec14l5*](http://www.ncbi.nlm.nih.gov/entrez/query.fcgi?db=gene&cmd=Retrieve&dopt=full_report&list_uids=665119) | SEC14-like 5 (S. cerevisiae) | 40 | 95 |
| mmu-miR-351-5p | [*Kctd21*](http://www.ncbi.nlm.nih.gov/entrez/query.fcgi?db=gene&cmd=Retrieve&dopt=full_report&list_uids=622320) | potassium channel tetramerisation domain containing 21 | 41 | 95 |
| mmu-miR-351-5p | [*Lactb*](http://www.ncbi.nlm.nih.gov/entrez/query.fcgi?db=gene&cmd=Retrieve&dopt=full_report&list_uids=80907) | lactamase, beta | 42 | 95 |
| mmu-miR-351-5p | [*Vps4b*](http://www.ncbi.nlm.nih.gov/entrez/query.fcgi?db=gene&cmd=Retrieve&dopt=full_report&list_uids=20479) | vacuolar protein sorting 4b (yeast) | 43 | 95 |
| mmu-miR-351-5p | [*Cyb561d1*](http://www.ncbi.nlm.nih.gov/entrez/query.fcgi?db=gene&cmd=Retrieve&dopt=full_report&list_uids=72023) | cytochrome b-561 domain containing 1 | 44 | 95 |
| mmu-miR-351-5p | [*Sema4b*](http://www.ncbi.nlm.nih.gov/entrez/query.fcgi?db=gene&cmd=Retrieve&dopt=full_report&list_uids=20352) | sema domain, immunoglobulin domain (Ig), transmembrane domain (TM) and short cytoplasmic domain, (semaphorin) 4B | 45 | 94 |
| mmu-miR-351-5p | [*Tmem161b*](http://www.ncbi.nlm.nih.gov/entrez/query.fcgi?db=gene&cmd=Retrieve&dopt=full_report&list_uids=72745) | transmembrane protein 161B | 46 | 94 |
| mmu-miR-351-5p | [*C77080*](http://www.ncbi.nlm.nih.gov/entrez/query.fcgi?db=gene&cmd=Retrieve&dopt=full_report&list_uids=97130) | expressed sequence C77080 | 47 | 94 |
| mmu-miR-351-5p | [*Rest*](http://www.ncbi.nlm.nih.gov/entrez/query.fcgi?db=gene&cmd=Retrieve&dopt=full_report&list_uids=19712) | RE1-silencing transcription factor | 48 | 94 |
| mmu-miR-351-5p | [*Blzf1*](http://www.ncbi.nlm.nih.gov/entrez/query.fcgi?db=gene&cmd=Retrieve&dopt=full_report&list_uids=66352) | basic leucine zipper nuclear factor 1 | 49 | 94 |
| mmu-miR-351-5p | [*Scn4a*](http://www.ncbi.nlm.nih.gov/entrez/query.fcgi?db=gene&cmd=Retrieve&dopt=full_report&list_uids=110880) | sodium channel, voltage-gated, type IV, alpha | 50 | 94 |
| mmu-miR-351-5p | [*Prdm1*](http://www.ncbi.nlm.nih.gov/entrez/query.fcgi?db=gene&cmd=Retrieve&dopt=full_report&list_uids=12142) | PR domain containing 1, with ZNF domain | 51 | 93 |
| mmu-miR-351-5p | [*Uck2*](http://www.ncbi.nlm.nih.gov/entrez/query.fcgi?db=gene&cmd=Retrieve&dopt=full_report&list_uids=80914) | uridine-cytidine kinase 2 | 52 | 93 |
| mmu-miR-351-5p | [*Dus1l*](http://www.ncbi.nlm.nih.gov/entrez/query.fcgi?db=gene&cmd=Retrieve&dopt=full_report&list_uids=68730) | dihydrouridine synthase 1-like (S. cerevisiae) | 53 | 93 |
| mmu-miR-351-5p | [*Lfng*](http://www.ncbi.nlm.nih.gov/entrez/query.fcgi?db=gene&cmd=Retrieve&dopt=full_report&list_uids=16848) | LFNG O-fucosylpeptide 3-beta-N-acetylglucosaminyltransferase | 54 | 93 |
| mmu-miR-351-5p | [*Trp53inp1*](http://www.ncbi.nlm.nih.gov/entrez/query.fcgi?db=gene&cmd=Retrieve&dopt=full_report&list_uids=60599) | transformation related protein 53 inducible nuclear protein 1 | 55 | 93 |
| mmu-miR-351-5p | [*Klhl25*](http://www.ncbi.nlm.nih.gov/entrez/query.fcgi?db=gene&cmd=Retrieve&dopt=full_report&list_uids=207952) | kelch-like 25 | 56 | 93 |
| mmu-miR-351-5p | [*Sarm1*](http://www.ncbi.nlm.nih.gov/entrez/query.fcgi?db=gene&cmd=Retrieve&dopt=full_report&list_uids=237868) | sterile alpha and HEAT/Armadillo motif containing 1 | 57 | 93 |
| mmu-miR-351-5p | [*Mobp*](http://www.ncbi.nlm.nih.gov/entrez/query.fcgi?db=gene&cmd=Retrieve&dopt=full_report&list_uids=17433) | myelin-associated oligodendrocytic basic protein | 58 | 93 |
| mmu-miR-351-5p | [*Coro2a*](http://www.ncbi.nlm.nih.gov/entrez/query.fcgi?db=gene&cmd=Retrieve&dopt=full_report&list_uids=107684) | coronin, actin binding protein 2A | 59 | 92 |
| mmu-miR-351-5p | [*Cbx7*](http://www.ncbi.nlm.nih.gov/entrez/query.fcgi?db=gene&cmd=Retrieve&dopt=full_report&list_uids=52609) | chromobox 7 | 60 | 92 |
| mmu-miR-351-5p | [*Pafah1b1*](http://www.ncbi.nlm.nih.gov/entrez/query.fcgi?db=gene&cmd=Retrieve&dopt=full_report&list_uids=18472) | platelet-activating factor acetylhydrolase, isoform 1b, subunit 1 | 61 | 92 |
| mmu-miR-351-5p | [*Gm9994*](http://www.ncbi.nlm.nih.gov/entrez/query.fcgi?db=gene&cmd=Retrieve&dopt=full_report&list_uids=100041596) | predicted gene 9994 | 62 | 92 |
| mmu-miR-351-5p | [*Morc2a*](http://www.ncbi.nlm.nih.gov/entrez/query.fcgi?db=gene&cmd=Retrieve&dopt=full_report&list_uids=74522) | microrchidia 2A | 63 | 92 |
| mmu-miR-351-5p | [*Arid3a*](http://www.ncbi.nlm.nih.gov/entrez/query.fcgi?db=gene&cmd=Retrieve&dopt=full_report&list_uids=13496) | AT rich interactive domain 3A (BRIGHT-like) | 64 | 92 |
| mmu-miR-351-5p | [*Smurf1*](http://www.ncbi.nlm.nih.gov/entrez/query.fcgi?db=gene&cmd=Retrieve&dopt=full_report&list_uids=75788) | SMAD specific E3 ubiquitin protein ligase 1 | 65 | 92 |
| mmu-miR-351-5p | [*Speg*](http://www.ncbi.nlm.nih.gov/entrez/query.fcgi?db=gene&cmd=Retrieve&dopt=full_report&list_uids=11790) | SPEG complex locus | 66 | 92 |
| mmu-miR-351-5p | [*Stard13*](http://www.ncbi.nlm.nih.gov/entrez/query.fcgi?db=gene&cmd=Retrieve&dopt=full_report&list_uids=243362) | StAR-related lipid transfer (START) domain containing 13 | 67 | 92 |
| mmu-miR-351-5p | [*2610528J11Rik*](http://www.ncbi.nlm.nih.gov/entrez/query.fcgi?db=gene&cmd=Retrieve&dopt=full_report&list_uids=66451) | RIKEN cDNA 2610528J11 gene | 68 | 92 |
| mmu-miR-351-5p | [*Trps1*](http://www.ncbi.nlm.nih.gov/entrez/query.fcgi?db=gene&cmd=Retrieve&dopt=full_report&list_uids=83925) | trichorhinophalangeal syndrome I (human) | 69 | 92 |
| mmu-miR-351-5p | [*Gpd1*](http://www.ncbi.nlm.nih.gov/entrez/query.fcgi?db=gene&cmd=Retrieve&dopt=full_report&list_uids=14555) | glycerol-3-phosphate dehydrogenase 1 (soluble) | 70 | 92 |
| mmu-miR-351-5p | [*Lrp4*](http://www.ncbi.nlm.nih.gov/entrez/query.fcgi?db=gene&cmd=Retrieve&dopt=full_report&list_uids=228357) | low density lipoprotein receptor-related protein 4 | 71 | 92 |
| mmu-miR-351-5p | [*Atoh8*](http://www.ncbi.nlm.nih.gov/entrez/query.fcgi?db=gene&cmd=Retrieve&dopt=full_report&list_uids=71093) | atonal homolog 8 (Drosophila) | 72 | 92 |
| mmu-miR-351-5p | [*Ptpn7*](http://www.ncbi.nlm.nih.gov/entrez/query.fcgi?db=gene&cmd=Retrieve&dopt=full_report&list_uids=320139) | protein tyrosine phosphatase, non-receptor type 7 | 73 | 92 |
| mmu-miR-351-5p | [*Tmem174*](http://www.ncbi.nlm.nih.gov/entrez/query.fcgi?db=gene&cmd=Retrieve&dopt=full_report&list_uids=68344) | transmembrane protein 174 | 74 | 91 |
| mmu-miR-351-5p | [*Fam53c*](http://www.ncbi.nlm.nih.gov/entrez/query.fcgi?db=gene&cmd=Retrieve&dopt=full_report&list_uids=66306) | family with sequence similarity 53, member C | 75 | 91 |
| mmu-miR-351-5p | [*Klhl24*](http://www.ncbi.nlm.nih.gov/entrez/query.fcgi?db=gene&cmd=Retrieve&dopt=full_report&list_uids=75785) | kelch-like 24 | 76 | 91 |
| mmu-miR-351-5p | [*Dram2*](http://www.ncbi.nlm.nih.gov/entrez/query.fcgi?db=gene&cmd=Retrieve&dopt=full_report&list_uids=67171) | DNA-damage regulated autophagy modulator 2 | 77 | 91 |
| mmu-miR-351-5p | [*Frmd5*](http://www.ncbi.nlm.nih.gov/entrez/query.fcgi?db=gene&cmd=Retrieve&dopt=full_report&list_uids=228564) | FERM domain containing 5 | 78 | 91 |
| mmu-miR-351-5p | [*Rnmt*](http://www.ncbi.nlm.nih.gov/entrez/query.fcgi?db=gene&cmd=Retrieve&dopt=full_report&list_uids=67897) | RNA (guanine-7-) methyltransferase | 79 | 91 |
| mmu-miR-351-5p | [*AU019823*](http://www.ncbi.nlm.nih.gov/entrez/query.fcgi?db=gene&cmd=Retrieve&dopt=full_report&list_uids=270156) | expressed sequence AU019823 | 80 | 91 |
| mmu-miR-351-5p | [*Acer2*](http://www.ncbi.nlm.nih.gov/entrez/query.fcgi?db=gene&cmd=Retrieve&dopt=full_report&list_uids=230379) | alkaline ceramidase 2 | 81 | 91 |
| mmu-miR-351-5p | [*Dennd6a*](http://www.ncbi.nlm.nih.gov/entrez/query.fcgi?db=gene&cmd=Retrieve&dopt=full_report&list_uids=211922) | DENN/MADD domain containing 6A | 82 | 91 |
| mmu-miR-351-5p | [*Rbm20*](http://www.ncbi.nlm.nih.gov/entrez/query.fcgi?db=gene&cmd=Retrieve&dopt=full_report&list_uids=73713) | RNA binding motif protein 20 | 83 | 91 |
| mmu-miR-351-5p | [*Kcnh7*](http://www.ncbi.nlm.nih.gov/entrez/query.fcgi?db=gene&cmd=Retrieve&dopt=full_report&list_uids=170738) | potassium voltage-gated channel, subfamily H (eag-related), member 7 | 84 | 91 |
| mmu-miR-351-5p | [*Mfsd9*](http://www.ncbi.nlm.nih.gov/entrez/query.fcgi?db=gene&cmd=Retrieve&dopt=full_report&list_uids=211798) | major facilitator superfamily domain containing 9 | 85 | 91 |
| mmu-miR-351-5p | [*Bap1*](http://www.ncbi.nlm.nih.gov/entrez/query.fcgi?db=gene&cmd=Retrieve&dopt=full_report&list_uids=104416) | Brca1 associated protein 1 | 86 | 91 |
| mmu-miR-351-5p | [*Arrb1*](http://www.ncbi.nlm.nih.gov/entrez/query.fcgi?db=gene&cmd=Retrieve&dopt=full_report&list_uids=109689) | arrestin, beta 1 | 87 | 91 |
| mmu-miR-351-5p | [*Msrb3*](http://www.ncbi.nlm.nih.gov/entrez/query.fcgi?db=gene&cmd=Retrieve&dopt=full_report&list_uids=320183) | methionine sulfoxide reductase B3 | 88 | 91 |
| mmu-miR-351-5p | [*Galnt14*](http://www.ncbi.nlm.nih.gov/entrez/query.fcgi?db=gene&cmd=Retrieve&dopt=full_report&list_uids=71685) | UDP-N-acetyl-alpha-D-galactosamine:polypeptide N-acetylgalactosaminyltransferase 14 | 89 | 90 |
| mmu-miR-351-5p | [*Anpep*](http://www.ncbi.nlm.nih.gov/entrez/query.fcgi?db=gene&cmd=Retrieve&dopt=full_report&list_uids=16790) | alanyl (membrane) aminopeptidase | 90 | 90 |
| mmu-miR-351-5p | [*C1qtnf1*](http://www.ncbi.nlm.nih.gov/entrez/query.fcgi?db=gene&cmd=Retrieve&dopt=full_report&list_uids=56745) | C1q and tumor necrosis factor related protein 1 | 91 | 90 |
| mmu-miR-351-5p | [*Gm10153*](http://www.ncbi.nlm.nih.gov/entrez/query.fcgi?db=gene&cmd=Retrieve&dopt=full_report&list_uids=100039757) | predicted gene 10153 | 92 | 90 |
| mmu-miR-351-5p | [*Elovl6*](http://www.ncbi.nlm.nih.gov/entrez/query.fcgi?db=gene&cmd=Retrieve&dopt=full_report&list_uids=170439) | ELOVL family member 6, elongation of long chain fatty acids (yeast) | 93 | 90 |
| mmu-miR-351-5p | [*Triap1*](http://www.ncbi.nlm.nih.gov/entrez/query.fcgi?db=gene&cmd=Retrieve&dopt=full_report&list_uids=69076) | TP53 regulated inhibitor of apoptosis 1 | 94 | 90 |
| mmu-miR-351-5p | [*Vdr*](http://www.ncbi.nlm.nih.gov/entrez/query.fcgi?db=gene&cmd=Retrieve&dopt=full_report&list_uids=22337) | vitamin D receptor | 95 | 90 |
| mmu-miR-351-5p | [*Nup210*](http://www.ncbi.nlm.nih.gov/entrez/query.fcgi?db=gene&cmd=Retrieve&dopt=full_report&list_uids=54563) | nucleoporin 210 | 96 | 90 |
| mmu-miR-351-5p | [*Gcnt1*](http://www.ncbi.nlm.nih.gov/entrez/query.fcgi?db=gene&cmd=Retrieve&dopt=full_report&list_uids=14537) | glucosaminyl (N-acetyl) transferase 1, core 2 | 97 | 90 |
| mmu-miR-351-5p | [*Gga2*](http://www.ncbi.nlm.nih.gov/entrez/query.fcgi?db=gene&cmd=Retrieve&dopt=full_report&list_uids=74105) | golgi associated, gamma adaptin ear containing, ARF binding protein 2 | 98 | 90 |
| mmu-miR-351-5p | [*Fam212b*](http://www.ncbi.nlm.nih.gov/entrez/query.fcgi?db=gene&cmd=Retrieve&dopt=full_report&list_uids=109050) | family with sequence similarity 212, member B | 99 | 89 |
| mmu-miR-351-5p | [*Cyp24a1*](http://www.ncbi.nlm.nih.gov/entrez/query.fcgi?db=gene&cmd=Retrieve&dopt=full_report&list_uids=13081) | cytochrome P450, family 24, subfamily a, polypeptide 1 | 100 | 89 |
| mmu-miR-351-5p | [*Cln6*](http://www.ncbi.nlm.nih.gov/entrez/query.fcgi?db=gene&cmd=Retrieve&dopt=full_report&list_uids=76524) | ceroid-lipofuscinosis, neuronal 6 | 101 | 89 |
| mmu-miR-351-5p | [*Psmg3*](http://www.ncbi.nlm.nih.gov/entrez/query.fcgi?db=gene&cmd=Retrieve&dopt=full_report&list_uids=66506) | proteasome (prosome, macropain) assembly chaperone 3 | 102 | 89 |
| mmu-miR-351-5p | [*Gm16515*](http://www.ncbi.nlm.nih.gov/entrez/query.fcgi?db=gene&cmd=Retrieve&dopt=full_report&list_uids=24083) | predicted gene, Gm16515 | 103 | 89 |
| mmu-miR-351-5p | [*Jade2*](http://www.ncbi.nlm.nih.gov/entrez/query.fcgi?db=gene&cmd=Retrieve&dopt=full_report&list_uids=76901) | jade family PHD finger 2 | 104 | 89 |
| mmu-miR-351-5p | [*Cdk16*](http://www.ncbi.nlm.nih.gov/entrez/query.fcgi?db=gene&cmd=Retrieve&dopt=full_report&list_uids=18555) | cyclin-dependent kinase 16 | 105 | 89 |
| mmu-miR-351-5p | [*Atp11a*](http://www.ncbi.nlm.nih.gov/entrez/query.fcgi?db=gene&cmd=Retrieve&dopt=full_report&list_uids=50770) | ATPase, class VI, type 11A | 106 | 88 |
| mmu-miR-351-5p | [*Etv6*](http://www.ncbi.nlm.nih.gov/entrez/query.fcgi?db=gene&cmd=Retrieve&dopt=full_report&list_uids=14011) | ets variant gene 6 (TEL oncogene) | 107 | 88 |
| mmu-miR-351-5p | [*Ndufs4*](http://www.ncbi.nlm.nih.gov/entrez/query.fcgi?db=gene&cmd=Retrieve&dopt=full_report&list_uids=17993) | NADH dehydrogenase (ubiquinone) Fe-S protein 4 | 108 | 88 |
| mmu-miR-351-5p | [*Abhd3*](http://www.ncbi.nlm.nih.gov/entrez/query.fcgi?db=gene&cmd=Retrieve&dopt=full_report&list_uids=106861) | abhydrolase domain containing 3 | 109 | 88 |
| mmu-miR-351-5p | [*Itfg3*](http://www.ncbi.nlm.nih.gov/entrez/query.fcgi?db=gene&cmd=Retrieve&dopt=full_report&list_uids=106581) | integrin alpha FG-GAP repeat containing 3 | 110 | 88 |
| mmu-miR-351-5p | [*Sufu*](http://www.ncbi.nlm.nih.gov/entrez/query.fcgi?db=gene&cmd=Retrieve&dopt=full_report&list_uids=24069) | suppressor of fused homolog (Drosophila) | 111 | 88 |
| mmu-miR-351-5p | [*Alg6*](http://www.ncbi.nlm.nih.gov/entrez/query.fcgi?db=gene&cmd=Retrieve&dopt=full_report&list_uids=320438) | asparagine-linked glycosylation 6 (alpha-1,3,-glucosyltransferase) | 112 | 88 |
| mmu-miR-351-5p | [*Zfp523*](http://www.ncbi.nlm.nih.gov/entrez/query.fcgi?db=gene&cmd=Retrieve&dopt=full_report&list_uids=224656) | zinc finger protein 523 | 113 | 88 |
| mmu-miR-351-5p | [*Tmtc2*](http://www.ncbi.nlm.nih.gov/entrez/query.fcgi?db=gene&cmd=Retrieve&dopt=full_report&list_uids=278279) | transmembrane and tetratricopeptide repeat containing 2 | 114 | 88 |
| mmu-miR-351-5p | [*Itga9*](http://www.ncbi.nlm.nih.gov/entrez/query.fcgi?db=gene&cmd=Retrieve&dopt=full_report&list_uids=104099) | integrin alpha 9 | 115 | 88 |
| mmu-miR-351-5p | [*Mapre2*](http://www.ncbi.nlm.nih.gov/entrez/query.fcgi?db=gene&cmd=Retrieve&dopt=full_report&list_uids=212307) | microtubule-associated protein, RP/EB family, member 2 | 116 | 88 |
| mmu-miR-351-5p | [*Diras1*](http://www.ncbi.nlm.nih.gov/entrez/query.fcgi?db=gene&cmd=Retrieve&dopt=full_report&list_uids=208666) | DIRAS family, GTP-binding RAS-like 1 | 117 | 88 |
| mmu-miR-351-5p | [*Ppat*](http://www.ncbi.nlm.nih.gov/entrez/query.fcgi?db=gene&cmd=Retrieve&dopt=full_report&list_uids=231327) | phosphoribosyl pyrophosphate amidotransferase | 118 | 87 |
| mmu-miR-351-5p | [*Ppm1f*](http://www.ncbi.nlm.nih.gov/entrez/query.fcgi?db=gene&cmd=Retrieve&dopt=full_report&list_uids=68606) | protein phosphatase 1F (PP2C domain containing) | 119 | 87 |
| mmu-miR-351-5p | [*Cntn2*](http://www.ncbi.nlm.nih.gov/entrez/query.fcgi?db=gene&cmd=Retrieve&dopt=full_report&list_uids=21367) | contactin 2 | 120 | 87 |
| mmu-miR-351-5p | [*Suv420h2*](http://www.ncbi.nlm.nih.gov/entrez/query.fcgi?db=gene&cmd=Retrieve&dopt=full_report&list_uids=232811) | suppressor of variegation 4-20 homolog 2 (Drosophila) | 121 | 87 |
| mmu-miR-351-5p | [*Ahrr*](http://www.ncbi.nlm.nih.gov/entrez/query.fcgi?db=gene&cmd=Retrieve&dopt=full_report&list_uids=11624) | aryl-hydrocarbon receptor repressor | 122 | 87 |
| mmu-miR-351-5p | [*Sptb*](http://www.ncbi.nlm.nih.gov/entrez/query.fcgi?db=gene&cmd=Retrieve&dopt=full_report&list_uids=20741) | spectrin beta, erythrocytic | 123 | 87 |
| mmu-miR-351-5p | [*Serpinb9d*](http://www.ncbi.nlm.nih.gov/entrez/query.fcgi?db=gene&cmd=Retrieve&dopt=full_report&list_uids=20726) | serine (or cysteine) peptidase inhibitor, clade B, member 9d | 124 | 87 |
| mmu-miR-351-5p | [*Mamdc2*](http://www.ncbi.nlm.nih.gov/entrez/query.fcgi?db=gene&cmd=Retrieve&dopt=full_report&list_uids=71738) | MAM domain containing 2 | 125 | 87 |
| mmu-miR-351-5p | [*Acads*](http://www.ncbi.nlm.nih.gov/entrez/query.fcgi?db=gene&cmd=Retrieve&dopt=full_report&list_uids=11409) | acyl-Coenzyme A dehydrogenase, short chain | 126 | 87 |
| mmu-miR-351-5p | [*Tmprss13*](http://www.ncbi.nlm.nih.gov/entrez/query.fcgi?db=gene&cmd=Retrieve&dopt=full_report&list_uids=214531) | transmembrane protease, serine 13 | 127 | 87 |
| mmu-miR-351-5p | [*Tnfsf4*](http://www.ncbi.nlm.nih.gov/entrez/query.fcgi?db=gene&cmd=Retrieve&dopt=full_report&list_uids=22164) | tumor necrosis factor (ligand) superfamily, member 4 | 128 | 87 |
| mmu-miR-351-5p | [*Cdc42bpg*](http://www.ncbi.nlm.nih.gov/entrez/query.fcgi?db=gene&cmd=Retrieve&dopt=full_report&list_uids=240505) | CDC42 binding protein kinase gamma (DMPK-like) | 129 | 87 |
| mmu-miR-351-5p | [*Sstr3*](http://www.ncbi.nlm.nih.gov/entrez/query.fcgi?db=gene&cmd=Retrieve&dopt=full_report&list_uids=20607) | somatostatin receptor 3 | 130 | 87 |
| mmu-miR-351-5p | [*Rfxank*](http://www.ncbi.nlm.nih.gov/entrez/query.fcgi?db=gene&cmd=Retrieve&dopt=full_report&list_uids=19727) | regulatory factor X-associated ankyrin-containing protein | 131 | 87 |
| mmu-miR-351-5p | [*Eva1a*](http://www.ncbi.nlm.nih.gov/entrez/query.fcgi?db=gene&cmd=Retrieve&dopt=full_report&list_uids=232146) | eva-1 homolog A (C. elegans) | 132 | 86 |
| mmu-miR-351-5p | [*Ist1*](http://www.ncbi.nlm.nih.gov/entrez/query.fcgi?db=gene&cmd=Retrieve&dopt=full_report&list_uids=71955) | increased sodium tolerance 1 homolog (yeast) | 133 | 86 |
| mmu-miR-351-5p | [*Lin28a*](http://www.ncbi.nlm.nih.gov/entrez/query.fcgi?db=gene&cmd=Retrieve&dopt=full_report&list_uids=83557) | lin-28 homolog A (C. elegans) | 134 | 86 |
| mmu-miR-351-5p | [*Pcgf6*](http://www.ncbi.nlm.nih.gov/entrez/query.fcgi?db=gene&cmd=Retrieve&dopt=full_report&list_uids=71041) | polycomb group ring finger 6 | 135 | 86 |
| mmu-miR-351-5p | [*Nin*](http://www.ncbi.nlm.nih.gov/entrez/query.fcgi?db=gene&cmd=Retrieve&dopt=full_report&list_uids=18080) | ninein | 136 | 86 |
| mmu-miR-351-5p | [*Fam134a*](http://www.ncbi.nlm.nih.gov/entrez/query.fcgi?db=gene&cmd=Retrieve&dopt=full_report&list_uids=227298) | family with sequence similarity 134, member A | 137 | 86 |
| mmu-miR-351-5p | [*Tle3*](http://www.ncbi.nlm.nih.gov/entrez/query.fcgi?db=gene&cmd=Retrieve&dopt=full_report&list_uids=21887) | transducin-like enhancer of split 3, homolog of Drosophila E(spl) | 138 | 86 |
| mmu-miR-351-5p | [*Gtf2h3*](http://www.ncbi.nlm.nih.gov/entrez/query.fcgi?db=gene&cmd=Retrieve&dopt=full_report&list_uids=209357) | general transcription factor IIH, polypeptide 3 | 139 | 86 |
| mmu-miR-351-5p | [*Map3k13*](http://www.ncbi.nlm.nih.gov/entrez/query.fcgi?db=gene&cmd=Retrieve&dopt=full_report&list_uids=71751) | mitogen-activated protein kinase kinase kinase 13 | 140 | 86 |
| mmu-miR-351-5p | [*Klf13*](http://www.ncbi.nlm.nih.gov/entrez/query.fcgi?db=gene&cmd=Retrieve&dopt=full_report&list_uids=50794) | Kruppel-like factor 13 | 141 | 86 |
| mmu-miR-351-5p | [*Ttc7*](http://www.ncbi.nlm.nih.gov/entrez/query.fcgi?db=gene&cmd=Retrieve&dopt=full_report&list_uids=225049) | tetratricopeptide repeat domain 7 | 142 | 86 |
| mmu-miR-351-5p | [*Scarb1*](http://www.ncbi.nlm.nih.gov/entrez/query.fcgi?db=gene&cmd=Retrieve&dopt=full_report&list_uids=20778) | scavenger receptor class B, member 1 | 143 | 85 |
| mmu-miR-351-5p | [*Rbm7*](http://www.ncbi.nlm.nih.gov/entrez/query.fcgi?db=gene&cmd=Retrieve&dopt=full_report&list_uids=67010) | RNA binding motif protein 7 | 144 | 85 |
| mmu-miR-351-5p | [*Syvn1*](http://www.ncbi.nlm.nih.gov/entrez/query.fcgi?db=gene&cmd=Retrieve&dopt=full_report&list_uids=74126) | synovial apoptosis inhibitor 1, synoviolin | 145 | 85 |
| mmu-miR-351-5p | [*Cdh9*](http://www.ncbi.nlm.nih.gov/entrez/query.fcgi?db=gene&cmd=Retrieve&dopt=full_report&list_uids=12565) | cadherin 9 | 146 | 85 |
| mmu-miR-351-5p | [*Ccnj*](http://www.ncbi.nlm.nih.gov/entrez/query.fcgi?db=gene&cmd=Retrieve&dopt=full_report&list_uids=240665) | cyclin J | 147 | 85 |
| mmu-miR-351-5p | [*Csde1*](http://www.ncbi.nlm.nih.gov/entrez/query.fcgi?db=gene&cmd=Retrieve&dopt=full_report&list_uids=229663) | cold shock domain containing E1, RNA binding | 148 | 85 |
| mmu-miR-351-5p | [*Bhlhe41*](http://www.ncbi.nlm.nih.gov/entrez/query.fcgi?db=gene&cmd=Retrieve&dopt=full_report&list_uids=79362) | basic helix-loop-helix family, member e41 | 149 | 85 |
| mmu-miR-351-5p | [*Pla2g2f*](http://www.ncbi.nlm.nih.gov/entrez/query.fcgi?db=gene&cmd=Retrieve&dopt=full_report&list_uids=26971) | phospholipase A2, group IIF | 150 | 85 |
| mmu-miR-351-5p | [*Meis2*](http://www.ncbi.nlm.nih.gov/entrez/query.fcgi?db=gene&cmd=Retrieve&dopt=full_report&list_uids=17536) | Meis homeobox 2 | 151 | 85 |
| mmu-miR-351-5p | [*Myo18a*](http://www.ncbi.nlm.nih.gov/entrez/query.fcgi?db=gene&cmd=Retrieve&dopt=full_report&list_uids=360013) | myosin XVIIIA | 152 | 85 |
| mmu-miR-351-5p | [*Ppp2r5c*](http://www.ncbi.nlm.nih.gov/entrez/query.fcgi?db=gene&cmd=Retrieve&dopt=full_report&list_uids=26931) | protein phosphatase 2, regulatory subunit B', gamma | 153 | 85 |
| mmu-miR-351-5p | [*Dtx4*](http://www.ncbi.nlm.nih.gov/entrez/query.fcgi?db=gene&cmd=Retrieve&dopt=full_report&list_uids=207521) | deltex 4 homolog (Drosophila) | 154 | 84 |
| mmu-miR-351-5p | [*Cacna1b*](http://www.ncbi.nlm.nih.gov/entrez/query.fcgi?db=gene&cmd=Retrieve&dopt=full_report&list_uids=12287) | calcium channel, voltage-dependent, N type, alpha 1B subunit | 155 | 84 |
| mmu-miR-351-5p | [*Usp45*](http://www.ncbi.nlm.nih.gov/entrez/query.fcgi?db=gene&cmd=Retrieve&dopt=full_report&list_uids=77593) | ubiquitin specific petidase 45 | 156 | 84 |
| mmu-miR-351-5p | [*Rabl6*](http://www.ncbi.nlm.nih.gov/entrez/query.fcgi?db=gene&cmd=Retrieve&dopt=full_report&list_uids=227624) | RAB, member of RAS oncogene family-like 6 | 157 | 84 |
| mmu-miR-351-5p | [*Taz*](http://www.ncbi.nlm.nih.gov/entrez/query.fcgi?db=gene&cmd=Retrieve&dopt=full_report&list_uids=66826) | tafazzin | 158 | 84 |
| mmu-miR-351-5p | [*Wars*](http://www.ncbi.nlm.nih.gov/entrez/query.fcgi?db=gene&cmd=Retrieve&dopt=full_report&list_uids=22375) | tryptophanyl-tRNA synthetase | 159 | 84 |
| mmu-miR-351-5p | [*Sema4f*](http://www.ncbi.nlm.nih.gov/entrez/query.fcgi?db=gene&cmd=Retrieve&dopt=full_report&list_uids=20355) | sema domain, immunoglobulin domain (Ig), TM domain, and short cytoplasmic domain | 160 | 84 |
| mmu-miR-351-5p | [*Serpinb5*](http://www.ncbi.nlm.nih.gov/entrez/query.fcgi?db=gene&cmd=Retrieve&dopt=full_report&list_uids=20724) | serine (or cysteine) peptidase inhibitor, clade B, member 5 | 161 | 84 |
| mmu-miR-351-5p | [*Nipal4*](http://www.ncbi.nlm.nih.gov/entrez/query.fcgi?db=gene&cmd=Retrieve&dopt=full_report&list_uids=214112) | NIPA-like domain containing 4 | 162 | 84 |
| mmu-miR-351-5p | [*Dicer1*](http://www.ncbi.nlm.nih.gov/entrez/query.fcgi?db=gene&cmd=Retrieve&dopt=full_report&list_uids=192119) | dicer 1, ribonuclease type III | 163 | 84 |
| mmu-miR-351-5p | [*Daam1*](http://www.ncbi.nlm.nih.gov/entrez/query.fcgi?db=gene&cmd=Retrieve&dopt=full_report&list_uids=208846) | dishevelled associated activator of morphogenesis 1 | 164 | 84 |
| mmu-miR-351-5p | [*Abtb1*](http://www.ncbi.nlm.nih.gov/entrez/query.fcgi?db=gene&cmd=Retrieve&dopt=full_report&list_uids=80283) | ankyrin repeat and BTB (POZ) domain containing 1 | 165 | 83 |
| mmu-miR-351-5p | [*Ubn1*](http://www.ncbi.nlm.nih.gov/entrez/query.fcgi?db=gene&cmd=Retrieve&dopt=full_report&list_uids=170644) | ubinuclein 1 | 166 | 83 |
| mmu-miR-351-5p | [*Cdr2l*](http://www.ncbi.nlm.nih.gov/entrez/query.fcgi?db=gene&cmd=Retrieve&dopt=full_report&list_uids=237988) | cerebellar degeneration-related protein 2-like | 167 | 83 |
| mmu-miR-351-5p | [*Ppme1*](http://www.ncbi.nlm.nih.gov/entrez/query.fcgi?db=gene&cmd=Retrieve&dopt=full_report&list_uids=72590) | protein phosphatase methylesterase 1 | 168 | 83 |
| mmu-miR-351-5p | [*Khnyn*](http://www.ncbi.nlm.nih.gov/entrez/query.fcgi?db=gene&cmd=Retrieve&dopt=full_report&list_uids=219094) | KH and NYN domain containing | 169 | 83 |
| mmu-miR-351-5p | [*Plekhm3*](http://www.ncbi.nlm.nih.gov/entrez/query.fcgi?db=gene&cmd=Retrieve&dopt=full_report&list_uids=241075) | pleckstrin homology domain containing, family M, member 3 | 170 | 83 |
| mmu-miR-351-5p | [*Klc2*](http://www.ncbi.nlm.nih.gov/entrez/query.fcgi?db=gene&cmd=Retrieve&dopt=full_report&list_uids=16594) | kinesin light chain 2 | 171 | 83 |
| mmu-miR-351-5p | [*2310047M10Rik*](http://www.ncbi.nlm.nih.gov/entrez/query.fcgi?db=gene&cmd=Retrieve&dopt=full_report&list_uids=71923) | RIKEN cDNA 2310047M10 gene | 172 | 83 |
| mmu-miR-351-5p | [*Elmsan1*](http://www.ncbi.nlm.nih.gov/entrez/query.fcgi?db=gene&cmd=Retrieve&dopt=full_report&list_uids=238317) | ELM2 and Myb/SANT-like domain containing 1 | 173 | 83 |
| mmu-miR-351-5p | [*Cdc42se1*](http://www.ncbi.nlm.nih.gov/entrez/query.fcgi?db=gene&cmd=Retrieve&dopt=full_report&list_uids=57912) | CDC42 small effector 1 | 174 | 83 |
| mmu-miR-351-5p | [*Scnn1a*](http://www.ncbi.nlm.nih.gov/entrez/query.fcgi?db=gene&cmd=Retrieve&dopt=full_report&list_uids=20276) | sodium channel, nonvoltage-gated 1 alpha | 175 | 83 |
| mmu-miR-351-5p | [*Fbxo10*](http://www.ncbi.nlm.nih.gov/entrez/query.fcgi?db=gene&cmd=Retrieve&dopt=full_report&list_uids=269529) | F-box protein 10 | 176 | 83 |
| mmu-miR-351-5p | [*P2rx4*](http://www.ncbi.nlm.nih.gov/entrez/query.fcgi?db=gene&cmd=Retrieve&dopt=full_report&list_uids=18438) | purinergic receptor P2X, ligand-gated ion channel 4 | 177 | 82 |
| mmu-miR-351-5p | [*Arid3b*](http://www.ncbi.nlm.nih.gov/entrez/query.fcgi?db=gene&cmd=Retrieve&dopt=full_report&list_uids=56380) | AT rich interactive domain 3B (BRIGHT-like) | 178 | 82 |
| mmu-miR-351-5p | [*Rap1a*](http://www.ncbi.nlm.nih.gov/entrez/query.fcgi?db=gene&cmd=Retrieve&dopt=full_report&list_uids=109905) | RAS-related protein-1a | 179 | 82 |
| mmu-miR-351-5p | [*Ncln*](http://www.ncbi.nlm.nih.gov/entrez/query.fcgi?db=gene&cmd=Retrieve&dopt=full_report&list_uids=103425) | nicalin homolog (zebrafish) | 180 | 82 |
| mmu-miR-351-5p | [*Tgoln1*](http://www.ncbi.nlm.nih.gov/entrez/query.fcgi?db=gene&cmd=Retrieve&dopt=full_report&list_uids=22134) | trans-golgi network protein | 181 | 82 |
| mmu-miR-351-5p | [*Itga7*](http://www.ncbi.nlm.nih.gov/entrez/query.fcgi?db=gene&cmd=Retrieve&dopt=full_report&list_uids=16404) | integrin alpha 7 | 182 | 82 |
| mmu-miR-351-5p | [*Zfp385a*](http://www.ncbi.nlm.nih.gov/entrez/query.fcgi?db=gene&cmd=Retrieve&dopt=full_report&list_uids=29813) | zinc finger protein 385A | 183 | 82 |
| mmu-miR-351-5p | [*0610010F05Rik*](http://www.ncbi.nlm.nih.gov/entrez/query.fcgi?db=gene&cmd=Retrieve&dopt=full_report&list_uids=71675) | RIKEN cDNA 0610010F05 gene | 184 | 81 |
| mmu-miR-351-5p | [*Lrrc71*](http://www.ncbi.nlm.nih.gov/entrez/query.fcgi?db=gene&cmd=Retrieve&dopt=full_report&list_uids=74485) | leucine rich repeat containing 71 | 185 | 81 |
| mmu-miR-351-5p | [*Dis3l2*](http://www.ncbi.nlm.nih.gov/entrez/query.fcgi?db=gene&cmd=Retrieve&dopt=full_report&list_uids=208718) | DIS3 mitotic control homolog (S. cerevisiae)-like 2 | 186 | 81 |
| mmu-miR-351-5p | [*Sh3tc2*](http://www.ncbi.nlm.nih.gov/entrez/query.fcgi?db=gene&cmd=Retrieve&dopt=full_report&list_uids=225608) | SH3 domain and tetratricopeptide repeats 2 | 187 | 81 |
| mmu-miR-351-5p | [*Kif24*](http://www.ncbi.nlm.nih.gov/entrez/query.fcgi?db=gene&cmd=Retrieve&dopt=full_report&list_uids=109242) | kinesin family member 24 | 188 | 81 |
| mmu-miR-351-5p | [*Mtus1*](http://www.ncbi.nlm.nih.gov/entrez/query.fcgi?db=gene&cmd=Retrieve&dopt=full_report&list_uids=102103) | mitochondrial tumor suppressor 1 | 189 | 81 |
| mmu-miR-351-5p | [*Sertad3*](http://www.ncbi.nlm.nih.gov/entrez/query.fcgi?db=gene&cmd=Retrieve&dopt=full_report&list_uids=170742) | SERTA domain containing 3 | 190 | 80 |
| mmu-miR-351-5p | [*Ptpn18*](http://www.ncbi.nlm.nih.gov/entrez/query.fcgi?db=gene&cmd=Retrieve&dopt=full_report&list_uids=19253) | protein tyrosine phosphatase, non-receptor type 18 | 191 | 80 |
| mmu-miR-351-5p | [*Fam131b*](http://www.ncbi.nlm.nih.gov/entrez/query.fcgi?db=gene&cmd=Retrieve&dopt=full_report&list_uids=76156) | family with sequence similarity 131, member B | 192 | 80 |
| mmu-miR-351-5p | [*Atp1b4*](http://www.ncbi.nlm.nih.gov/entrez/query.fcgi?db=gene&cmd=Retrieve&dopt=full_report&list_uids=67821) | ATPase, (Na+)/K+ transporting, beta 4 polypeptide | 193 | 80 |
| mmu-miR-351-5p | [*Atg4d*](http://www.ncbi.nlm.nih.gov/entrez/query.fcgi?db=gene&cmd=Retrieve&dopt=full_report&list_uids=235040) | autophagy related 4D, cysteine peptidase | 194 | 80 |
| mmu-miR-351-5p | [*Tjap1*](http://www.ncbi.nlm.nih.gov/entrez/query.fcgi?db=gene&cmd=Retrieve&dopt=full_report&list_uids=74094) | tight junction associated protein 1 | 195 | 80 |
| mmu-miR-351-5p | [*Il16*](http://www.ncbi.nlm.nih.gov/entrez/query.fcgi?db=gene&cmd=Retrieve&dopt=full_report&list_uids=16170) | interleukin 16 | 196 | 80 |
| mmu-miR-351-5p | [*Cpsf6*](http://www.ncbi.nlm.nih.gov/entrez/query.fcgi?db=gene&cmd=Retrieve&dopt=full_report&list_uids=432508) | cleavage and polyadenylation specific factor 6 | 197 | 80 |
| mmu-miR-351-5p | [*Iqce*](http://www.ncbi.nlm.nih.gov/entrez/query.fcgi?db=gene&cmd=Retrieve&dopt=full_report&list_uids=74239) | IQ motif containing E | 198 | 80 |
| mmu-miR-181c-5p | [*Prox1*](http://www.ncbi.nlm.nih.gov/entrez/query.fcgi?db=gene&cmd=Retrieve&dopt=full_report&list_uids=19130) | prospero homeobox 1 | 1 | 100 |
| mmu-miR-181c-5p | [*Tnpo1*](http://www.ncbi.nlm.nih.gov/entrez/query.fcgi?db=gene&cmd=Retrieve&dopt=full_report&list_uids=238799) | transportin 1 | 2 | 100 |
| mmu-miR-181c-5p | [*Ythdc2*](http://www.ncbi.nlm.nih.gov/entrez/query.fcgi?db=gene&cmd=Retrieve&dopt=full_report&list_uids=240255) | YTH domain containing 2 | 3 | 100 |
| mmu-miR-181c-5p | [*Fign*](http://www.ncbi.nlm.nih.gov/entrez/query.fcgi?db=gene&cmd=Retrieve&dopt=full_report&list_uids=60344) | fidgetin | 4 | 100 |
| mmu-miR-181c-5p | [*Rps6ka3*](http://www.ncbi.nlm.nih.gov/entrez/query.fcgi?db=gene&cmd=Retrieve&dopt=full_report&list_uids=110651) | ribosomal protein S6 kinase polypeptide 3 | 5 | 100 |
| mmu-miR-181c-5p | [*Fmnl2*](http://www.ncbi.nlm.nih.gov/entrez/query.fcgi?db=gene&cmd=Retrieve&dopt=full_report&list_uids=71409) | formin-like 2 | 6 | 100 |
| mmu-miR-181c-5p | [*Phf20l1*](http://www.ncbi.nlm.nih.gov/entrez/query.fcgi?db=gene&cmd=Retrieve&dopt=full_report&list_uids=239510) | PHD finger protein 20-like 1 | 7 | 100 |
| mmu-miR-181c-5p | [*Gpr22*](http://www.ncbi.nlm.nih.gov/entrez/query.fcgi?db=gene&cmd=Retrieve&dopt=full_report&list_uids=73010) | G protein-coupled receptor 22 | 8 | 100 |
| mmu-miR-181c-5p | [*Pi4k2b*](http://www.ncbi.nlm.nih.gov/entrez/query.fcgi?db=gene&cmd=Retrieve&dopt=full_report&list_uids=67073) | phosphatidylinositol 4-kinase type 2 beta | 9 | 100 |
| mmu-miR-181c-5p | [*Kmt2a*](http://www.ncbi.nlm.nih.gov/entrez/query.fcgi?db=gene&cmd=Retrieve&dopt=full_report&list_uids=214162) | lysine (K)-specific methyltransferase 2A | 10 | 100 |
| mmu-miR-181c-5p | [*Greb1l*](http://www.ncbi.nlm.nih.gov/entrez/query.fcgi?db=gene&cmd=Retrieve&dopt=full_report&list_uids=381157) | growth regulation by estrogen in breast cancer-like | 11 | 100 |
| mmu-miR-181c-5p | [*Sfmbt1*](http://www.ncbi.nlm.nih.gov/entrez/query.fcgi?db=gene&cmd=Retrieve&dopt=full_report&list_uids=54650) | Scm-like with four mbt domains 1 | 12 | 100 |
| mmu-miR-181c-5p | [*Spry4*](http://www.ncbi.nlm.nih.gov/entrez/query.fcgi?db=gene&cmd=Retrieve&dopt=full_report&list_uids=24066) | sprouty homolog 4 (Drosophila) | 13 | 100 |
| mmu-miR-181c-5p | [*Ddx3x*](http://www.ncbi.nlm.nih.gov/entrez/query.fcgi?db=gene&cmd=Retrieve&dopt=full_report&list_uids=13205) | DEAD/H (Asp-Glu-Ala-Asp/His) box polypeptide 3, X-linked | 14 | 99 |
| mmu-miR-181c-5p | [*Ssx2ip*](http://www.ncbi.nlm.nih.gov/entrez/query.fcgi?db=gene&cmd=Retrieve&dopt=full_report&list_uids=99167) | synovial sarcoma, X breakpoint 2 interacting protein | 15 | 99 |
| mmu-miR-181c-5p | [*Gm14440*](http://www.ncbi.nlm.nih.gov/entrez/query.fcgi?db=gene&cmd=Retrieve&dopt=full_report&list_uids=100503353) | predicted gene 14440 | 16 | 99 |
| mmu-miR-181c-5p | [*Nova1*](http://www.ncbi.nlm.nih.gov/entrez/query.fcgi?db=gene&cmd=Retrieve&dopt=full_report&list_uids=664883) | neuro-oncological ventral antigen 1 | 17 | 99 |
| mmu-miR-181c-5p | [*Nek7*](http://www.ncbi.nlm.nih.gov/entrez/query.fcgi?db=gene&cmd=Retrieve&dopt=full_report&list_uids=59125) | NIMA (never in mitosis gene a)-related expressed kinase 7 | 18 | 99 |
| mmu-miR-181c-5p | [*Crebrf*](http://www.ncbi.nlm.nih.gov/entrez/query.fcgi?db=gene&cmd=Retrieve&dopt=full_report&list_uids=77128) | CREB3 regulatory factor | 19 | 99 |
| mmu-miR-181c-5p | [*Dnajc13*](http://www.ncbi.nlm.nih.gov/entrez/query.fcgi?db=gene&cmd=Retrieve&dopt=full_report&list_uids=235567) | DnaJ (Hsp40) homolog, subfamily C, member 13 | 20 | 99 |
| mmu-miR-181c-5p | [*Spire1*](http://www.ncbi.nlm.nih.gov/entrez/query.fcgi?db=gene&cmd=Retrieve&dopt=full_report&list_uids=68166) | spire homolog 1 (Drosophila) | 21 | 99 |
| mmu-miR-181c-5p | [*Btbd3*](http://www.ncbi.nlm.nih.gov/entrez/query.fcgi?db=gene&cmd=Retrieve&dopt=full_report&list_uids=228662) | BTB (POZ) domain containing 3 | 22 | 99 |
| mmu-miR-181c-5p | [*Chic1*](http://www.ncbi.nlm.nih.gov/entrez/query.fcgi?db=gene&cmd=Retrieve&dopt=full_report&list_uids=12212) | cysteine-rich hydrophobic domain 1 | 23 | 99 |
| mmu-miR-181c-5p | [*Specc1l*](http://www.ncbi.nlm.nih.gov/entrez/query.fcgi?db=gene&cmd=Retrieve&dopt=full_report&list_uids=74392) | sperm antigen with calponin homology and coiled-coil domains 1-like | 24 | 99 |
| mmu-miR-181c-5p | [*Cops2*](http://www.ncbi.nlm.nih.gov/entrez/query.fcgi?db=gene&cmd=Retrieve&dopt=full_report&list_uids=12848) | COP9 (constitutive photomorphogenic) homolog, subunit 2 (Arabidopsis thaliana) | 25 | 99 |
| mmu-miR-181c-5p | [*1700066M21Rik*](http://www.ncbi.nlm.nih.gov/entrez/query.fcgi?db=gene&cmd=Retrieve&dopt=full_report&list_uids=73467) | RIKEN cDNA 1700066M21 gene | 26 | 99 |
| mmu-miR-181c-5p | [*Clip1*](http://www.ncbi.nlm.nih.gov/entrez/query.fcgi?db=gene&cmd=Retrieve&dopt=full_report&list_uids=56430) | CAP-GLY domain containing linker protein 1 | 27 | 99 |
| mmu-miR-181c-5p | [*Prtg*](http://www.ncbi.nlm.nih.gov/entrez/query.fcgi?db=gene&cmd=Retrieve&dopt=full_report&list_uids=235472) | protogenin homolog (Gallus gallus) | 28 | 99 |
| mmu-miR-181c-5p | [*Gfpt1*](http://www.ncbi.nlm.nih.gov/entrez/query.fcgi?db=gene&cmd=Retrieve&dopt=full_report&list_uids=14583) | glutamine fructose-6-phosphate transaminase 1 | 29 | 98 |
| mmu-miR-181c-5p | [*Gskip*](http://www.ncbi.nlm.nih.gov/entrez/query.fcgi?db=gene&cmd=Retrieve&dopt=full_report&list_uids=66787) | GSK3B interacting protein | 30 | 98 |
| mmu-miR-181c-5p | [*Tulp4*](http://www.ncbi.nlm.nih.gov/entrez/query.fcgi?db=gene&cmd=Retrieve&dopt=full_report&list_uids=68842) | tubby like protein 4 | 31 | 98 |
| mmu-miR-181c-5p | [*E2f5*](http://www.ncbi.nlm.nih.gov/entrez/query.fcgi?db=gene&cmd=Retrieve&dopt=full_report&list_uids=13559) | E2F transcription factor 5 | 32 | 98 |
| mmu-miR-181c-5p | [*Esr1*](http://www.ncbi.nlm.nih.gov/entrez/query.fcgi?db=gene&cmd=Retrieve&dopt=full_report&list_uids=13982) | estrogen receptor 1 (alpha) | 33 | 98 |
| mmu-miR-181c-5p | [*Rbm46*](http://www.ncbi.nlm.nih.gov/entrez/query.fcgi?db=gene&cmd=Retrieve&dopt=full_report&list_uids=633285) | RNA binding motif protein 46 | 34 | 98 |
| mmu-miR-181c-5p | [*Prkcd*](http://www.ncbi.nlm.nih.gov/entrez/query.fcgi?db=gene&cmd=Retrieve&dopt=full_report&list_uids=18753) | protein kinase C, delta | 35 | 98 |
| mmu-miR-181c-5p | [*Zfp800*](http://www.ncbi.nlm.nih.gov/entrez/query.fcgi?db=gene&cmd=Retrieve&dopt=full_report&list_uids=627049) | zinc finger protein 800 | 36 | 98 |
| mmu-miR-181c-5p | [*Rnf145*](http://www.ncbi.nlm.nih.gov/entrez/query.fcgi?db=gene&cmd=Retrieve&dopt=full_report&list_uids=74315) | ring finger protein 145 | 37 | 98 |
| mmu-miR-181c-5p | [*Tgfbr1*](http://www.ncbi.nlm.nih.gov/entrez/query.fcgi?db=gene&cmd=Retrieve&dopt=full_report&list_uids=21812) | transforming growth factor, beta receptor I | 38 | 98 |
| mmu-miR-181c-5p | [*Osbpl3*](http://www.ncbi.nlm.nih.gov/entrez/query.fcgi?db=gene&cmd=Retrieve&dopt=full_report&list_uids=71720) | oxysterol binding protein-like 3 | 39 | 98 |
| mmu-miR-181c-5p | [*Rnmt*](http://www.ncbi.nlm.nih.gov/entrez/query.fcgi?db=gene&cmd=Retrieve&dopt=full_report&list_uids=67897) | RNA (guanine-7-) methyltransferase | 40 | 98 |
| mmu-miR-181c-5p | [*Zfp781*](http://www.ncbi.nlm.nih.gov/entrez/query.fcgi?db=gene&cmd=Retrieve&dopt=full_report&list_uids=331188) | zinc finger protein 781 | 41 | 98 |
| mmu-miR-181c-5p | [*Ap1s3*](http://www.ncbi.nlm.nih.gov/entrez/query.fcgi?db=gene&cmd=Retrieve&dopt=full_report&list_uids=252903) | adaptor-related protein complex AP-1, sigma 3 | 42 | 98 |
| mmu-miR-181c-5p | [*Mtf2*](http://www.ncbi.nlm.nih.gov/entrez/query.fcgi?db=gene&cmd=Retrieve&dopt=full_report&list_uids=17765) | metal response element binding transcription factor 2 | 43 | 98 |
| mmu-miR-181c-5p | [*Larp4*](http://www.ncbi.nlm.nih.gov/entrez/query.fcgi?db=gene&cmd=Retrieve&dopt=full_report&list_uids=207214) | La ribonucleoprotein domain family, member 4 | 44 | 98 |
| mmu-miR-181c-5p | [*Cdyl*](http://www.ncbi.nlm.nih.gov/entrez/query.fcgi?db=gene&cmd=Retrieve&dopt=full_report&list_uids=12593) | chromodomain protein, Y chromosome-like | 45 | 98 |
| mmu-miR-181c-5p | [*Ppip5k2*](http://www.ncbi.nlm.nih.gov/entrez/query.fcgi?db=gene&cmd=Retrieve&dopt=full_report&list_uids=227399) | diphosphoinositol pentakisphosphate kinase 2 | 46 | 98 |
| mmu-miR-181c-5p | [*Elmsan1*](http://www.ncbi.nlm.nih.gov/entrez/query.fcgi?db=gene&cmd=Retrieve&dopt=full_report&list_uids=238317) | ELM2 and Myb/SANT-like domain containing 1 | 47 | 98 |
| mmu-miR-181c-5p | [*Slitrk1*](http://www.ncbi.nlm.nih.gov/entrez/query.fcgi?db=gene&cmd=Retrieve&dopt=full_report&list_uids=76965) | SLIT and NTRK-like family, member 1 | 48 | 98 |
| mmu-miR-181c-5p | [*Epc2*](http://www.ncbi.nlm.nih.gov/entrez/query.fcgi?db=gene&cmd=Retrieve&dopt=full_report&list_uids=227867) | enhancer of polycomb homolog 2 (Drosophila) | 49 | 98 |
| mmu-miR-181c-5p | [*Taf9b*](http://www.ncbi.nlm.nih.gov/entrez/query.fcgi?db=gene&cmd=Retrieve&dopt=full_report&list_uids=407786) | TAF9B RNA polymerase II, TATA box binding protein (TBP)-associated factor | 50 | 98 |
| mmu-miR-181c-5p | [*Cblb*](http://www.ncbi.nlm.nih.gov/entrez/query.fcgi?db=gene&cmd=Retrieve&dopt=full_report&list_uids=208650) | Casitas B-lineage lymphoma b | 51 | 98 |
| mmu-miR-181c-5p | [*Bend3*](http://www.ncbi.nlm.nih.gov/entrez/query.fcgi?db=gene&cmd=Retrieve&dopt=full_report&list_uids=331623) | BEN domain containing 3 | 52 | 98 |
| mmu-miR-181c-5p | [*Klhl29*](http://www.ncbi.nlm.nih.gov/entrez/query.fcgi?db=gene&cmd=Retrieve&dopt=full_report&list_uids=208439) | kelch-like 29 | 53 | 98 |
| mmu-miR-181c-5p | [*Cpd*](http://www.ncbi.nlm.nih.gov/entrez/query.fcgi?db=gene&cmd=Retrieve&dopt=full_report&list_uids=12874) | carboxypeptidase D | 54 | 98 |
| mmu-miR-181c-5p | [*Itsn2*](http://www.ncbi.nlm.nih.gov/entrez/query.fcgi?db=gene&cmd=Retrieve&dopt=full_report&list_uids=20403) | intersectin 2 | 55 | 98 |
| mmu-miR-181c-5p | [*Palb2*](http://www.ncbi.nlm.nih.gov/entrez/query.fcgi?db=gene&cmd=Retrieve&dopt=full_report&list_uids=233826) | partner and localizer of BRCA2 | 56 | 98 |
| mmu-miR-181c-5p | [*Pdap1*](http://www.ncbi.nlm.nih.gov/entrez/query.fcgi?db=gene&cmd=Retrieve&dopt=full_report&list_uids=231887) | PDGFA associated protein 1 | 57 | 98 |
| mmu-miR-181c-5p | [*Gm14436*](http://www.ncbi.nlm.nih.gov/entrez/query.fcgi?db=gene&cmd=Retrieve&dopt=full_report&list_uids=100190996) | predicted gene 14436 | 58 | 97 |
| mmu-miR-181c-5p | [*Tspan13*](http://www.ncbi.nlm.nih.gov/entrez/query.fcgi?db=gene&cmd=Retrieve&dopt=full_report&list_uids=66109) | tetraspanin 13 | 59 | 97 |
| mmu-miR-181c-5p | [*Klf6*](http://www.ncbi.nlm.nih.gov/entrez/query.fcgi?db=gene&cmd=Retrieve&dopt=full_report&list_uids=23849) | Kruppel-like factor 6 | 60 | 97 |
| mmu-miR-181c-5p | [*Mpp5*](http://www.ncbi.nlm.nih.gov/entrez/query.fcgi?db=gene&cmd=Retrieve&dopt=full_report&list_uids=56217) | membrane protein, palmitoylated 5 (MAGUK p55 subfamily member 5) | 61 | 97 |
| mmu-miR-181c-5p | [*Klf15*](http://www.ncbi.nlm.nih.gov/entrez/query.fcgi?db=gene&cmd=Retrieve&dopt=full_report&list_uids=66277) | Kruppel-like factor 15 | 62 | 97 |
| mmu-miR-181c-5p | [*Ube2b*](http://www.ncbi.nlm.nih.gov/entrez/query.fcgi?db=gene&cmd=Retrieve&dopt=full_report&list_uids=22210) | ubiquitin-conjugating enzyme E2B | 63 | 97 |
| mmu-miR-181c-5p | [*Ccp110*](http://www.ncbi.nlm.nih.gov/entrez/query.fcgi?db=gene&cmd=Retrieve&dopt=full_report&list_uids=101565) | centriolar coiled coil protein 110 | 64 | 97 |
| mmu-miR-181c-5p | [*Gse1*](http://www.ncbi.nlm.nih.gov/entrez/query.fcgi?db=gene&cmd=Retrieve&dopt=full_report&list_uids=382034) | genetic suppressor element 1 | 65 | 97 |
| mmu-miR-181c-5p | [*Gpsm1*](http://www.ncbi.nlm.nih.gov/entrez/query.fcgi?db=gene&cmd=Retrieve&dopt=full_report&list_uids=67839) | G-protein signalling modulator 1 (AGS3-like, C. elegans) | 66 | 97 |
| mmu-miR-181c-5p | [*Spice1*](http://www.ncbi.nlm.nih.gov/entrez/query.fcgi?db=gene&cmd=Retrieve&dopt=full_report&list_uids=212514) | spindle and centriole associated protein 1 | 67 | 97 |
| mmu-miR-181c-5p | [*Lrrc32*](http://www.ncbi.nlm.nih.gov/entrez/query.fcgi?db=gene&cmd=Retrieve&dopt=full_report&list_uids=434215) | leucine rich repeat containing 32 | 68 | 97 |
| mmu-miR-181c-5p | [*Ptbp3*](http://www.ncbi.nlm.nih.gov/entrez/query.fcgi?db=gene&cmd=Retrieve&dopt=full_report&list_uids=230257) | polypyrimidine tract binding protein 3 | 69 | 97 |
| mmu-miR-181c-5p | [*Ksr1*](http://www.ncbi.nlm.nih.gov/entrez/query.fcgi?db=gene&cmd=Retrieve&dopt=full_report&list_uids=16706) | kinase suppressor of ras 1 | 70 | 97 |
| mmu-miR-181c-5p | [*Cdc40*](http://www.ncbi.nlm.nih.gov/entrez/query.fcgi?db=gene&cmd=Retrieve&dopt=full_report&list_uids=71713) | cell division cycle 40 | 71 | 97 |
| mmu-miR-181c-5p | [*Zic3*](http://www.ncbi.nlm.nih.gov/entrez/query.fcgi?db=gene&cmd=Retrieve&dopt=full_report&list_uids=22773) | zinc finger protein of the cerebellum 3 | 72 | 97 |
| mmu-miR-181c-5p | [*Sec24a*](http://www.ncbi.nlm.nih.gov/entrez/query.fcgi?db=gene&cmd=Retrieve&dopt=full_report&list_uids=77371) | Sec24 related gene family, member A (S. cerevisiae) | 73 | 97 |
| mmu-miR-181c-5p | [*D430041D05Rik*](http://www.ncbi.nlm.nih.gov/entrez/query.fcgi?db=gene&cmd=Retrieve&dopt=full_report&list_uids=241589) | RIKEN cDNA D430041D05 gene | 74 | 97 |
| mmu-miR-181c-5p | [*Lmo1*](http://www.ncbi.nlm.nih.gov/entrez/query.fcgi?db=gene&cmd=Retrieve&dopt=full_report&list_uids=109594) | LIM domain only 1 | 75 | 97 |
| mmu-miR-181c-5p | [*Ythdf3*](http://www.ncbi.nlm.nih.gov/entrez/query.fcgi?db=gene&cmd=Retrieve&dopt=full_report&list_uids=229096) | YTH domain family 3 | 76 | 97 |
| mmu-miR-181c-5p | [*Slc4a10*](http://www.ncbi.nlm.nih.gov/entrez/query.fcgi?db=gene&cmd=Retrieve&dopt=full_report&list_uids=94229) | solute carrier family 4, sodium bicarbonate cotransporter-like, member 10 | 77 | 97 |
| mmu-miR-181c-5p | [*Nr6a1*](http://www.ncbi.nlm.nih.gov/entrez/query.fcgi?db=gene&cmd=Retrieve&dopt=full_report&list_uids=14536) | nuclear receptor subfamily 6, group A, member 1 | 78 | 97 |
| mmu-miR-181c-5p | [*Slc25a36*](http://www.ncbi.nlm.nih.gov/entrez/query.fcgi?db=gene&cmd=Retrieve&dopt=full_report&list_uids=192287) | solute carrier family 25, member 36 | 79 | 97 |
| mmu-miR-181c-5p | [*Man2a1*](http://www.ncbi.nlm.nih.gov/entrez/query.fcgi?db=gene&cmd=Retrieve&dopt=full_report&list_uids=17158) | mannosidase 2, alpha 1 | 80 | 97 |
| mmu-miR-181c-5p | [*Hipk3*](http://www.ncbi.nlm.nih.gov/entrez/query.fcgi?db=gene&cmd=Retrieve&dopt=full_report&list_uids=15259) | homeodomain interacting protein kinase 3 | 81 | 97 |
| mmu-miR-181c-5p | [*Entpd6*](http://www.ncbi.nlm.nih.gov/entrez/query.fcgi?db=gene&cmd=Retrieve&dopt=full_report&list_uids=12497) | ectonucleoside triphosphate diphosphohydrolase 6 | 82 | 96 |
| mmu-miR-181c-5p | [*Cnot6l*](http://www.ncbi.nlm.nih.gov/entrez/query.fcgi?db=gene&cmd=Retrieve&dopt=full_report&list_uids=231464) | CCR4-NOT transcription complex, subunit 6-like | 83 | 96 |
| mmu-miR-181c-5p | [*Mb21d2*](http://www.ncbi.nlm.nih.gov/entrez/query.fcgi?db=gene&cmd=Retrieve&dopt=full_report&list_uids=239796) | Mab-21 domain containing 2 | 84 | 96 |
| mmu-miR-181c-5p | [*Rbbp7*](http://www.ncbi.nlm.nih.gov/entrez/query.fcgi?db=gene&cmd=Retrieve&dopt=full_report&list_uids=245688) | retinoblastoma binding protein 7 | 85 | 96 |
| mmu-miR-181c-5p | [*Wwc2*](http://www.ncbi.nlm.nih.gov/entrez/query.fcgi?db=gene&cmd=Retrieve&dopt=full_report&list_uids=52357) | WW, C2 and coiled-coil domain containing 2 | 86 | 96 |
| mmu-miR-181c-5p | [*Pcdhac2*](http://www.ncbi.nlm.nih.gov/entrez/query.fcgi?db=gene&cmd=Retrieve&dopt=full_report&list_uids=353237) | protocadherin alpha subfamily C, 2 | 87 | 96 |
| mmu-miR-181c-5p | [*Acsl4*](http://www.ncbi.nlm.nih.gov/entrez/query.fcgi?db=gene&cmd=Retrieve&dopt=full_report&list_uids=50790) | acyl-CoA synthetase long-chain family member 4 | 88 | 96 |
| mmu-miR-181c-5p | [*Cpne2*](http://www.ncbi.nlm.nih.gov/entrez/query.fcgi?db=gene&cmd=Retrieve&dopt=full_report&list_uids=234577) | copine II | 89 | 96 |
| mmu-miR-181c-5p | [*Lox*](http://www.ncbi.nlm.nih.gov/entrez/query.fcgi?db=gene&cmd=Retrieve&dopt=full_report&list_uids=16948) | lysyl oxidase | 90 | 96 |
| mmu-miR-181c-5p | [*Cyp7a1*](http://www.ncbi.nlm.nih.gov/entrez/query.fcgi?db=gene&cmd=Retrieve&dopt=full_report&list_uids=13122) | cytochrome P450, family 7, subfamily a, polypeptide 1 | 91 | 96 |
| mmu-miR-181c-5p | [*Zfp280d*](http://www.ncbi.nlm.nih.gov/entrez/query.fcgi?db=gene&cmd=Retrieve&dopt=full_report&list_uids=235469) | zinc finger protein 280D | 92 | 96 |
| mmu-miR-181c-5p | [*Thrb*](http://www.ncbi.nlm.nih.gov/entrez/query.fcgi?db=gene&cmd=Retrieve&dopt=full_report&list_uids=21834) | thyroid hormone receptor beta | 93 | 96 |
| mmu-miR-181c-5p | [*Zfp120*](http://www.ncbi.nlm.nih.gov/entrez/query.fcgi?db=gene&cmd=Retrieve&dopt=full_report&list_uids=104348) | zinc finger protein 120 | 94 | 96 |
| mmu-miR-181c-5p | [*Nus1*](http://www.ncbi.nlm.nih.gov/entrez/query.fcgi?db=gene&cmd=Retrieve&dopt=full_report&list_uids=52014) | nuclear undecaprenyl pyrophosphate synthase 1 homolog (S. cerevisiae) | 95 | 96 |
| mmu-miR-181c-5p | [*Rad21*](http://www.ncbi.nlm.nih.gov/entrez/query.fcgi?db=gene&cmd=Retrieve&dopt=full_report&list_uids=19357) | RAD21 homolog (S. pombe) | 96 | 95 |
| mmu-miR-181c-5p | [*Mier3*](http://www.ncbi.nlm.nih.gov/entrez/query.fcgi?db=gene&cmd=Retrieve&dopt=full_report&list_uids=218613) | mesoderm induction early response 1, family member 3 | 97 | 95 |
| mmu-miR-181c-5p | [*Txndc12*](http://www.ncbi.nlm.nih.gov/entrez/query.fcgi?db=gene&cmd=Retrieve&dopt=full_report&list_uids=66073) | thioredoxin domain containing 12 (endoplasmic reticulum) | 98 | 95 |
| mmu-miR-181c-5p | [*Tcerg1*](http://www.ncbi.nlm.nih.gov/entrez/query.fcgi?db=gene&cmd=Retrieve&dopt=full_report&list_uids=56070) | transcription elongation regulator 1 (CA150) | 99 | 95 |
| mmu-miR-181c-5p | [*Mas1*](http://www.ncbi.nlm.nih.gov/entrez/query.fcgi?db=gene&cmd=Retrieve&dopt=full_report&list_uids=17171) | MAS1 oncogene | 100 | 95 |
| mmu-miR-181c-5p | [*Clasp1*](http://www.ncbi.nlm.nih.gov/entrez/query.fcgi?db=gene&cmd=Retrieve&dopt=full_report&list_uids=76707) | CLIP associating protein 1 | 101 | 95 |
| mmu-miR-181c-5p | [*Fam122b*](http://www.ncbi.nlm.nih.gov/entrez/query.fcgi?db=gene&cmd=Retrieve&dopt=full_report&list_uids=78755) | family with sequence similarity 122, member B | 102 | 95 |
| mmu-miR-181c-5p | [*Stxbp6*](http://www.ncbi.nlm.nih.gov/entrez/query.fcgi?db=gene&cmd=Retrieve&dopt=full_report&list_uids=217517) | syntaxin binding protein 6 (amisyn) | 103 | 95 |
| mmu-miR-181c-5p | [*Sfrs18*](http://www.ncbi.nlm.nih.gov/entrez/query.fcgi?db=gene&cmd=Retrieve&dopt=full_report&list_uids=66625) | serine/arginine-rich splicing factor 18 | 104 | 95 |
| mmu-miR-181c-5p | [*Cntn4*](http://www.ncbi.nlm.nih.gov/entrez/query.fcgi?db=gene&cmd=Retrieve&dopt=full_report&list_uids=269784) | contactin 4 | 105 | 95 |
| mmu-miR-181c-5p | [*Gabra1*](http://www.ncbi.nlm.nih.gov/entrez/query.fcgi?db=gene&cmd=Retrieve&dopt=full_report&list_uids=14394) | gamma-aminobutyric acid (GABA) A receptor, subunit alpha 1 | 106 | 95 |
| mmu-miR-181c-5p | [*Naa50*](http://www.ncbi.nlm.nih.gov/entrez/query.fcgi?db=gene&cmd=Retrieve&dopt=full_report&list_uids=72117) | N(alpha)-acetyltransferase 50, NatE catalytic subunit | 107 | 94 |
| mmu-miR-181c-5p | [*Tada2b*](http://www.ncbi.nlm.nih.gov/entrez/query.fcgi?db=gene&cmd=Retrieve&dopt=full_report&list_uids=231151) | transcriptional adaptor 2B | 108 | 94 |
| mmu-miR-181c-5p | [*Snn*](http://www.ncbi.nlm.nih.gov/entrez/query.fcgi?db=gene&cmd=Retrieve&dopt=full_report&list_uids=20621) | stannin | 109 | 94 |
| mmu-miR-181c-5p | [*Sun1*](http://www.ncbi.nlm.nih.gov/entrez/query.fcgi?db=gene&cmd=Retrieve&dopt=full_report&list_uids=77053) | Sad1 and UNC84 domain containing 1 | 110 | 94 |
| mmu-miR-181c-5p | [*Usp33*](http://www.ncbi.nlm.nih.gov/entrez/query.fcgi?db=gene&cmd=Retrieve&dopt=full_report&list_uids=170822) | ubiquitin specific peptidase 33 | 111 | 94 |
| mmu-miR-181c-5p | [*Dock4*](http://www.ncbi.nlm.nih.gov/entrez/query.fcgi?db=gene&cmd=Retrieve&dopt=full_report&list_uids=238130) | dedicator of cytokinesis 4 | 112 | 94 |
| mmu-miR-181c-5p | [*Rnf34*](http://www.ncbi.nlm.nih.gov/entrez/query.fcgi?db=gene&cmd=Retrieve&dopt=full_report&list_uids=80751) | ring finger protein 34 | 113 | 94 |
| mmu-miR-181c-5p | [*Lrba*](http://www.ncbi.nlm.nih.gov/entrez/query.fcgi?db=gene&cmd=Retrieve&dopt=full_report&list_uids=80877) | LPS-responsive beige-like anchor | 114 | 94 |
| mmu-miR-181c-5p | [*Fbxo33*](http://www.ncbi.nlm.nih.gov/entrez/query.fcgi?db=gene&cmd=Retrieve&dopt=full_report&list_uids=70611) | F-box protein 33 | 115 | 94 |
| mmu-miR-181c-5p | [*Zfp97*](http://www.ncbi.nlm.nih.gov/entrez/query.fcgi?db=gene&cmd=Retrieve&dopt=full_report&list_uids=22759) | zinc finger protein 97 | 116 | 94 |
| mmu-miR-181c-5p | [*Grm5*](http://www.ncbi.nlm.nih.gov/entrez/query.fcgi?db=gene&cmd=Retrieve&dopt=full_report&list_uids=108071) | glutamate receptor, metabotropic 5 | 117 | 94 |
| mmu-miR-181c-5p | [*Zfp960*](http://www.ncbi.nlm.nih.gov/entrez/query.fcgi?db=gene&cmd=Retrieve&dopt=full_report&list_uids=449000) | zinc finger protein 960 | 118 | 94 |
| mmu-miR-181c-5p | [*Esm1*](http://www.ncbi.nlm.nih.gov/entrez/query.fcgi?db=gene&cmd=Retrieve&dopt=full_report&list_uids=71690) | endothelial cell-specific molecule 1 | 119 | 94 |
| mmu-miR-181c-5p | [*Trak1*](http://www.ncbi.nlm.nih.gov/entrez/query.fcgi?db=gene&cmd=Retrieve&dopt=full_report&list_uids=67095) | trafficking protein, kinesin binding 1 | 120 | 94 |
| mmu-miR-181c-5p | [*Atp2b1*](http://www.ncbi.nlm.nih.gov/entrez/query.fcgi?db=gene&cmd=Retrieve&dopt=full_report&list_uids=67972) | ATPase, Ca++ transporting, plasma membrane 1 | 121 | 94 |
| mmu-miR-181c-5p | [*Lhx9*](http://www.ncbi.nlm.nih.gov/entrez/query.fcgi?db=gene&cmd=Retrieve&dopt=full_report&list_uids=16876) | LIM homeobox protein 9 | 122 | 93 |
| mmu-miR-181c-5p | [*Ppp3r1*](http://www.ncbi.nlm.nih.gov/entrez/query.fcgi?db=gene&cmd=Retrieve&dopt=full_report&list_uids=19058) | protein phosphatase 3, regulatory subunit B, alpha isoform (calcineurin B, type I) | 123 | 93 |
| mmu-miR-181c-5p | [*Usp42*](http://www.ncbi.nlm.nih.gov/entrez/query.fcgi?db=gene&cmd=Retrieve&dopt=full_report&list_uids=76800) | ubiquitin specific peptidase 42 | 124 | 93 |
| mmu-miR-181c-5p | [*Adam11*](http://www.ncbi.nlm.nih.gov/entrez/query.fcgi?db=gene&cmd=Retrieve&dopt=full_report&list_uids=11488) | a disintegrin and metallopeptidase domain 11 | 125 | 93 |
| mmu-miR-181c-5p | [*Unc80*](http://www.ncbi.nlm.nih.gov/entrez/query.fcgi?db=gene&cmd=Retrieve&dopt=full_report&list_uids=329178) | unc-80 homolog (C. elegans) | 126 | 93 |
| mmu-miR-181c-5p | [*Afg3l2*](http://www.ncbi.nlm.nih.gov/entrez/query.fcgi?db=gene&cmd=Retrieve&dopt=full_report&list_uids=69597) | AFG3(ATPase family gene 3)-like 2 (yeast) | 127 | 93 |
| mmu-miR-181c-5p | [*Fam3c*](http://www.ncbi.nlm.nih.gov/entrez/query.fcgi?db=gene&cmd=Retrieve&dopt=full_report&list_uids=27999) | family with sequence similarity 3, member C | 128 | 93 |
| mmu-miR-181c-5p | [*Gpd1l*](http://www.ncbi.nlm.nih.gov/entrez/query.fcgi?db=gene&cmd=Retrieve&dopt=full_report&list_uids=333433) | glycerol-3-phosphate dehydrogenase 1-like | 129 | 93 |
| mmu-miR-181c-5p | [*Arnt2*](http://www.ncbi.nlm.nih.gov/entrez/query.fcgi?db=gene&cmd=Retrieve&dopt=full_report&list_uids=11864) | aryl hydrocarbon receptor nuclear translocator 2 | 130 | 93 |
| mmu-miR-181c-5p | [*Ipo8*](http://www.ncbi.nlm.nih.gov/entrez/query.fcgi?db=gene&cmd=Retrieve&dopt=full_report&list_uids=320727) | importin 8 | 131 | 92 |
| mmu-miR-181c-5p | [*Crim1*](http://www.ncbi.nlm.nih.gov/entrez/query.fcgi?db=gene&cmd=Retrieve&dopt=full_report&list_uids=50766) | cysteine rich transmembrane BMP regulator 1 (chordin like) | 132 | 92 |
| mmu-miR-181c-5p | [*En1*](http://www.ncbi.nlm.nih.gov/entrez/query.fcgi?db=gene&cmd=Retrieve&dopt=full_report&list_uids=13798) | engrailed 1 | 133 | 92 |
| mmu-miR-181c-5p | [*Rps6kb1*](http://www.ncbi.nlm.nih.gov/entrez/query.fcgi?db=gene&cmd=Retrieve&dopt=full_report&list_uids=72508) | ribosomal protein S6 kinase, polypeptide 1 | 134 | 92 |
| mmu-miR-181c-5p | [*Ino80d*](http://www.ncbi.nlm.nih.gov/entrez/query.fcgi?db=gene&cmd=Retrieve&dopt=full_report&list_uids=227195) | INO80 complex subunit D | 135 | 92 |
| mmu-miR-181c-5p | [*Ralgapb*](http://www.ncbi.nlm.nih.gov/entrez/query.fcgi?db=gene&cmd=Retrieve&dopt=full_report&list_uids=228850) | Ral GTPase activating protein, beta subunit (non-catalytic) | 136 | 92 |
| mmu-miR-181c-5p | [*Dnajc21*](http://www.ncbi.nlm.nih.gov/entrez/query.fcgi?db=gene&cmd=Retrieve&dopt=full_report&list_uids=78244) | DnaJ (Hsp40) homolog, subfamily C, member 21 | 137 | 92 |
| mmu-miR-181c-5p | [*Sgpp1*](http://www.ncbi.nlm.nih.gov/entrez/query.fcgi?db=gene&cmd=Retrieve&dopt=full_report&list_uids=81535) | sphingosine-1-phosphate phosphatase 1 | 138 | 92 |
| mmu-miR-181c-5p | [*Tbc1d1*](http://www.ncbi.nlm.nih.gov/entrez/query.fcgi?db=gene&cmd=Retrieve&dopt=full_report&list_uids=57915) | TBC1 domain family, member 1 | 139 | 92 |
| mmu-miR-181c-5p | [*Sec24c*](http://www.ncbi.nlm.nih.gov/entrez/query.fcgi?db=gene&cmd=Retrieve&dopt=full_report&list_uids=218811) | Sec24 related gene family, member C (S. cerevisiae) | 140 | 92 |
| mmu-miR-181c-5p | [*Kcna4*](http://www.ncbi.nlm.nih.gov/entrez/query.fcgi?db=gene&cmd=Retrieve&dopt=full_report&list_uids=16492) | potassium voltage-gated channel, shaker-related subfamily, member 4 | 141 | 92 |
| mmu-miR-181c-5p | [*Cdc42bpa*](http://www.ncbi.nlm.nih.gov/entrez/query.fcgi?db=gene&cmd=Retrieve&dopt=full_report&list_uids=226751) | CDC42 binding protein kinase alpha | 142 | 92 |
| mmu-miR-181c-5p | [*Hoxa11*](http://www.ncbi.nlm.nih.gov/entrez/query.fcgi?db=gene&cmd=Retrieve&dopt=full_report&list_uids=15396) | homeobox A11 | 143 | 92 |
| mmu-miR-181c-5p | [*Zfp951*](http://www.ncbi.nlm.nih.gov/entrez/query.fcgi?db=gene&cmd=Retrieve&dopt=full_report&list_uids=626391) | zinc finger protein 951 | 144 | 92 |
| mmu-miR-181c-5p | [*Carf*](http://www.ncbi.nlm.nih.gov/entrez/query.fcgi?db=gene&cmd=Retrieve&dopt=full_report&list_uids=241066) | calcium response factor | 145 | 92 |
| mmu-miR-181c-5p | [*Srsf7*](http://www.ncbi.nlm.nih.gov/entrez/query.fcgi?db=gene&cmd=Retrieve&dopt=full_report&list_uids=225027) | serine/arginine-rich splicing factor 7 | 146 | 92 |
| mmu-miR-181c-5p | [*Ap1g1*](http://www.ncbi.nlm.nih.gov/entrez/query.fcgi?db=gene&cmd=Retrieve&dopt=full_report&list_uids=11765) | adaptor protein complex AP-1, gamma 1 subunit | 147 | 92 |
| mmu-miR-181c-5p | [*Hmbs*](http://www.ncbi.nlm.nih.gov/entrez/query.fcgi?db=gene&cmd=Retrieve&dopt=full_report&list_uids=15288) | hydroxymethylbilane synthase | 148 | 92 |
| mmu-miR-181c-5p | [*C77370*](http://www.ncbi.nlm.nih.gov/entrez/query.fcgi?db=gene&cmd=Retrieve&dopt=full_report&list_uids=245555) | expressed sequence C77370 | 149 | 92 |
| mmu-miR-181c-5p | [*Sbno1*](http://www.ncbi.nlm.nih.gov/entrez/query.fcgi?db=gene&cmd=Retrieve&dopt=full_report&list_uids=243272) | sno, strawberry notch homolog 1 (Drosophila) | 150 | 91 |
| mmu-miR-181c-5p | [*Fktn*](http://www.ncbi.nlm.nih.gov/entrez/query.fcgi?db=gene&cmd=Retrieve&dopt=full_report&list_uids=246179) | fukutin | 151 | 91 |
| mmu-miR-181c-5p | [*Lyrm1*](http://www.ncbi.nlm.nih.gov/entrez/query.fcgi?db=gene&cmd=Retrieve&dopt=full_report&list_uids=73919) | LYR motif containing 1 | 152 | 91 |
| mmu-miR-181c-5p | [*Gdap1*](http://www.ncbi.nlm.nih.gov/entrez/query.fcgi?db=gene&cmd=Retrieve&dopt=full_report&list_uids=14545) | ganglioside-induced differentiation-associated-protein 1 | 153 | 91 |
| mmu-miR-181c-5p | [*Nek10*](http://www.ncbi.nlm.nih.gov/entrez/query.fcgi?db=gene&cmd=Retrieve&dopt=full_report&list_uids=674895) | NIMA (never in mitosis gene a)- related kinase 10 | 154 | 91 |
| mmu-miR-181c-5p | [*Kank1*](http://www.ncbi.nlm.nih.gov/entrez/query.fcgi?db=gene&cmd=Retrieve&dopt=full_report&list_uids=107351) | KN motif and ankyrin repeat domains 1 | 155 | 91 |
| mmu-miR-181c-5p | [*Derl1*](http://www.ncbi.nlm.nih.gov/entrez/query.fcgi?db=gene&cmd=Retrieve&dopt=full_report&list_uids=67819) | Der1-like domain family, member 1 | 156 | 91 |
| mmu-miR-181c-5p | [*Prom2*](http://www.ncbi.nlm.nih.gov/entrez/query.fcgi?db=gene&cmd=Retrieve&dopt=full_report&list_uids=192212) | prominin 2 | 157 | 91 |
| mmu-miR-181c-5p | [*Cdon*](http://www.ncbi.nlm.nih.gov/entrez/query.fcgi?db=gene&cmd=Retrieve&dopt=full_report&list_uids=57810) | cell adhesion molecule-related/down-regulated by oncogenes | 158 | 91 |
| mmu-miR-181c-5p | [*Cpeb4*](http://www.ncbi.nlm.nih.gov/entrez/query.fcgi?db=gene&cmd=Retrieve&dopt=full_report&list_uids=67579) | cytoplasmic polyadenylation element binding protein 4 | 159 | 91 |
| mmu-miR-181c-5p | [*Zfp36l2*](http://www.ncbi.nlm.nih.gov/entrez/query.fcgi?db=gene&cmd=Retrieve&dopt=full_report&list_uids=12193) | zinc finger protein 36, C3H type-like 2 | 160 | 91 |
| mmu-miR-181c-5p | [*C2cd5*](http://www.ncbi.nlm.nih.gov/entrez/query.fcgi?db=gene&cmd=Retrieve&dopt=full_report&list_uids=74741) | C2 calcium-dependent domain containing 5 | 161 | 91 |
| mmu-miR-181c-5p | [*Ctdspl*](http://www.ncbi.nlm.nih.gov/entrez/query.fcgi?db=gene&cmd=Retrieve&dopt=full_report&list_uids=69274) | CTD (carboxy-terminal domain, RNA polymerase II, polypeptide A) small phosphatase-like | 162 | 91 |
| mmu-miR-181c-5p | [*Twistnb*](http://www.ncbi.nlm.nih.gov/entrez/query.fcgi?db=gene&cmd=Retrieve&dopt=full_report&list_uids=28071) | TWIST neighbor | 163 | 91 |
| mmu-miR-181c-5p | [*Gata6*](http://www.ncbi.nlm.nih.gov/entrez/query.fcgi?db=gene&cmd=Retrieve&dopt=full_report&list_uids=14465) | GATA binding protein 6 | 164 | 91 |
| mmu-miR-181c-5p | [*Grik2*](http://www.ncbi.nlm.nih.gov/entrez/query.fcgi?db=gene&cmd=Retrieve&dopt=full_report&list_uids=14806) | glutamate receptor, ionotropic, kainate 2 (beta 2) | 165 | 91 |
| mmu-miR-181c-5p | [*Med26*](http://www.ncbi.nlm.nih.gov/entrez/query.fcgi?db=gene&cmd=Retrieve&dopt=full_report&list_uids=70625) | mediator complex subunit 26 | 166 | 91 |
| mmu-miR-181c-5p | [*Ago2*](http://www.ncbi.nlm.nih.gov/entrez/query.fcgi?db=gene&cmd=Retrieve&dopt=full_report&list_uids=239528) | argonaute RISC catalytic subunit 2 | 167 | 90 |
| mmu-miR-181c-5p | [*Tnfrsf11b*](http://www.ncbi.nlm.nih.gov/entrez/query.fcgi?db=gene&cmd=Retrieve&dopt=full_report&list_uids=18383) | tumor necrosis factor receptor superfamily, member 11b (osteoprotegerin) | 168 | 90 |
| mmu-miR-181c-5p | [*Mfap3l*](http://www.ncbi.nlm.nih.gov/entrez/query.fcgi?db=gene&cmd=Retrieve&dopt=full_report&list_uids=71306) | microfibrillar-associated protein 3-like | 169 | 90 |
| mmu-miR-181c-5p | [*Cxadr*](http://www.ncbi.nlm.nih.gov/entrez/query.fcgi?db=gene&cmd=Retrieve&dopt=full_report&list_uids=13052) | coxsackie virus and adenovirus receptor | 170 | 90 |
| mmu-miR-181c-5p | [*Rabgef1*](http://www.ncbi.nlm.nih.gov/entrez/query.fcgi?db=gene&cmd=Retrieve&dopt=full_report&list_uids=56715) | RAB guanine nucleotide exchange factor (GEF) 1 | 171 | 90 |
| mmu-miR-181c-5p | [*Rlf*](http://www.ncbi.nlm.nih.gov/entrez/query.fcgi?db=gene&cmd=Retrieve&dopt=full_report&list_uids=109263) | rearranged L-myc fusion sequence | 172 | 90 |
| mmu-miR-181c-5p | [*Anapc16*](http://www.ncbi.nlm.nih.gov/entrez/query.fcgi?db=gene&cmd=Retrieve&dopt=full_report&list_uids=52717) | anaphase promoting complex subunit 16 | 173 | 90 |
| mmu-miR-181c-5p | [*Ddx55*](http://www.ncbi.nlm.nih.gov/entrez/query.fcgi?db=gene&cmd=Retrieve&dopt=full_report&list_uids=67848) | DEAD (Asp-Glu-Ala-Asp) box polypeptide 55 | 174 | 90 |
| mmu-miR-181c-5p | [*Pcdha8*](http://www.ncbi.nlm.nih.gov/entrez/query.fcgi?db=gene&cmd=Retrieve&dopt=full_report&list_uids=353235) | protocadherin alpha 8 | 175 | 90 |
| mmu-miR-181c-5p | [*Pcdha12*](http://www.ncbi.nlm.nih.gov/entrez/query.fcgi?db=gene&cmd=Retrieve&dopt=full_report&list_uids=192164) | protocadherin alpha 12 | 176 | 90 |
| mmu-miR-181c-5p | [*Pcdha3*](http://www.ncbi.nlm.nih.gov/entrez/query.fcgi?db=gene&cmd=Retrieve&dopt=full_report&list_uids=192163) | protocadherin alpha 3 | 177 | 90 |
| mmu-miR-181c-5p | [*Pcdha9*](http://www.ncbi.nlm.nih.gov/entrez/query.fcgi?db=gene&cmd=Retrieve&dopt=full_report&list_uids=192161) | protocadherin alpha 9 | 178 | 90 |
| mmu-miR-181c-5p | [*Pcdha1*](http://www.ncbi.nlm.nih.gov/entrez/query.fcgi?db=gene&cmd=Retrieve&dopt=full_report&list_uids=116731) | protocadherin alpha 1 | 179 | 90 |
| mmu-miR-181c-5p | [*Pcdha11*](http://www.ncbi.nlm.nih.gov/entrez/query.fcgi?db=gene&cmd=Retrieve&dopt=full_report&list_uids=12942) | protocadherin alpha 11 | 180 | 90 |
| mmu-miR-181c-5p | [*Pcdha5*](http://www.ncbi.nlm.nih.gov/entrez/query.fcgi?db=gene&cmd=Retrieve&dopt=full_report&list_uids=12941) | protocadherin alpha 5 | 181 | 90 |
| mmu-miR-181c-5p | [*Pcdha7*](http://www.ncbi.nlm.nih.gov/entrez/query.fcgi?db=gene&cmd=Retrieve&dopt=full_report&list_uids=12939) | protocadherin alpha 7 | 182 | 90 |
| mmu-miR-181c-5p | [*Pcdha6*](http://www.ncbi.nlm.nih.gov/entrez/query.fcgi?db=gene&cmd=Retrieve&dopt=full_report&list_uids=12937) | protocadherin alpha 6 | 183 | 90 |
| mmu-miR-181c-5p | [*Pcdha4*](http://www.ncbi.nlm.nih.gov/entrez/query.fcgi?db=gene&cmd=Retrieve&dopt=full_report&list_uids=12936) | protocadherin alpha 4 | 184 | 90 |
| mmu-miR-181c-5p | [*Pcdhac1*](http://www.ncbi.nlm.nih.gov/entrez/query.fcgi?db=gene&cmd=Retrieve&dopt=full_report&list_uids=353236) | protocadherin alpha subfamily C, 1 | 185 | 90 |
| mmu-miR-181c-5p | [*Rbm26*](http://www.ncbi.nlm.nih.gov/entrez/query.fcgi?db=gene&cmd=Retrieve&dopt=full_report&list_uids=74213) | RNA binding motif protein 26 | 186 | 90 |
| mmu-miR-181c-5p | [*Lpcat1*](http://www.ncbi.nlm.nih.gov/entrez/query.fcgi?db=gene&cmd=Retrieve&dopt=full_report&list_uids=210992) | lysophosphatidylcholine acyltransferase 1 | 187 | 90 |
| mmu-miR-181c-5p | [*Pcdha2*](http://www.ncbi.nlm.nih.gov/entrez/query.fcgi?db=gene&cmd=Retrieve&dopt=full_report&list_uids=353234) | protocadherin alpha 2 | 188 | 90 |
| mmu-miR-181c-5p | [*Lonrf2*](http://www.ncbi.nlm.nih.gov/entrez/query.fcgi?db=gene&cmd=Retrieve&dopt=full_report&list_uids=381338) | LON peptidase N-terminal domain and ring finger 2 | 189 | 90 |
| mmu-miR-181c-5p | [*Pkd1l1*](http://www.ncbi.nlm.nih.gov/entrez/query.fcgi?db=gene&cmd=Retrieve&dopt=full_report&list_uids=171395) | polycystic kidney disease 1 like 1 | 190 | 90 |
| mmu-miR-181c-5p | [*Akt3*](http://www.ncbi.nlm.nih.gov/entrez/query.fcgi?db=gene&cmd=Retrieve&dopt=full_report&list_uids=23797) | thymoma viral proto-oncogene 3 | 191 | 90 |
| mmu-miR-181c-5p | [*Prrg1*](http://www.ncbi.nlm.nih.gov/entrez/query.fcgi?db=gene&cmd=Retrieve&dopt=full_report&list_uids=546336) | proline rich Gla (G-carboxyglutamic acid) 1 | 192 | 90 |
| mmu-miR-181c-5p | [*St8sia3*](http://www.ncbi.nlm.nih.gov/entrez/query.fcgi?db=gene&cmd=Retrieve&dopt=full_report&list_uids=20451) | ST8 alpha-N-acetyl-neuraminide alpha-2,8-sialyltransferase 3 | 193 | 90 |
| mmu-miR-181c-5p | [*Per2*](http://www.ncbi.nlm.nih.gov/entrez/query.fcgi?db=gene&cmd=Retrieve&dopt=full_report&list_uids=18627) | period circadian clock 2 | 194 | 90 |
| mmu-miR-181c-5p | [*Ss18l1*](http://www.ncbi.nlm.nih.gov/entrez/query.fcgi?db=gene&cmd=Retrieve&dopt=full_report&list_uids=269397) | synovial sarcoma translocation gene on chromosome 18-like 1 | 195 | 89 |
| mmu-miR-181c-5p | [*Mbtps2*](http://www.ncbi.nlm.nih.gov/entrez/query.fcgi?db=gene&cmd=Retrieve&dopt=full_report&list_uids=270669) | membrane-bound transcription factor peptidase, site 2 | 196 | 89 |
| mmu-miR-181c-5p | [*Acsl1*](http://www.ncbi.nlm.nih.gov/entrez/query.fcgi?db=gene&cmd=Retrieve&dopt=full_report&list_uids=14081) | acyl-CoA synthetase long-chain family member 1 | 197 | 89 |
| mmu-miR-181c-5p | [*Carm1*](http://www.ncbi.nlm.nih.gov/entrez/query.fcgi?db=gene&cmd=Retrieve&dopt=full_report&list_uids=59035) | coactivator-associated arginine methyltransferase 1 | 198 | 89 |
| mmu-miR-181c-5p | [*3110047P20Rik*](http://www.ncbi.nlm.nih.gov/entrez/query.fcgi?db=gene&cmd=Retrieve&dopt=full_report&list_uids=319807) | RIKEN cDNA 3110047P20 gene | 199 | 89 |
| mmu-miR-181c-5p | [*Oxsm*](http://www.ncbi.nlm.nih.gov/entrez/query.fcgi?db=gene&cmd=Retrieve&dopt=full_report&list_uids=71147) | 3-oxoacyl-ACP synthase, mitochondrial | 200 | 89 |
| mmu-miR-181c-5p | [*Ttl*](http://www.ncbi.nlm.nih.gov/entrez/query.fcgi?db=gene&cmd=Retrieve&dopt=full_report&list_uids=69737) | tubulin tyrosine ligase | 201 | 89 |
| mmu-miR-181c-5p | [*Birc6*](http://www.ncbi.nlm.nih.gov/entrez/query.fcgi?db=gene&cmd=Retrieve&dopt=full_report&list_uids=12211) | baculoviral IAP repeat-containing 6 | 202 | 89 |
| mmu-miR-181c-5p | [*Gatm*](http://www.ncbi.nlm.nih.gov/entrez/query.fcgi?db=gene&cmd=Retrieve&dopt=full_report&list_uids=67092) | glycine amidinotransferase (L-arginine:glycine amidinotransferase) | 203 | 89 |
| mmu-miR-181c-5p | [*Fnip2*](http://www.ncbi.nlm.nih.gov/entrez/query.fcgi?db=gene&cmd=Retrieve&dopt=full_report&list_uids=329679) | folliculin interacting protein 2 | 204 | 89 |
| mmu-miR-181c-5p | [*Sox6*](http://www.ncbi.nlm.nih.gov/entrez/query.fcgi?db=gene&cmd=Retrieve&dopt=full_report&list_uids=20679) | SRY (sex determining region Y)-box 6 | 205 | 89 |
| mmu-miR-181c-5p | [*Me2*](http://www.ncbi.nlm.nih.gov/entrez/query.fcgi?db=gene&cmd=Retrieve&dopt=full_report&list_uids=107029) | malic enzyme 2, NAD(+)-dependent, mitochondrial | 206 | 89 |
| mmu-miR-181c-5p | [*Mtmr12*](http://www.ncbi.nlm.nih.gov/entrez/query.fcgi?db=gene&cmd=Retrieve&dopt=full_report&list_uids=268783) | myotubularin related protein 12 | 207 | 89 |
| mmu-miR-181c-5p | [*Lmbrd2*](http://www.ncbi.nlm.nih.gov/entrez/query.fcgi?db=gene&cmd=Retrieve&dopt=full_report&list_uids=320506) | LMBR1 domain containing 2 | 208 | 89 |
| mmu-miR-181c-5p | [*Ankrd13c*](http://www.ncbi.nlm.nih.gov/entrez/query.fcgi?db=gene&cmd=Retrieve&dopt=full_report&list_uids=433667) | ankyrin repeat domain 13c | 209 | 89 |
| mmu-miR-181c-5p | [*Map1b*](http://www.ncbi.nlm.nih.gov/entrez/query.fcgi?db=gene&cmd=Retrieve&dopt=full_report&list_uids=17755) | microtubule-associated protein 1B | 210 | 89 |
| mmu-miR-181c-5p | [*Cnksr3*](http://www.ncbi.nlm.nih.gov/entrez/query.fcgi?db=gene&cmd=Retrieve&dopt=full_report&list_uids=215748) | Cnksr family member 3 | 211 | 89 |
| mmu-miR-181c-5p | [*Ercc5*](http://www.ncbi.nlm.nih.gov/entrez/query.fcgi?db=gene&cmd=Retrieve&dopt=full_report&list_uids=22592) | excision repair cross-complementing rodent repair deficiency, complementation group 5 | 212 | 88 |
| mmu-miR-181c-5p | [*Yipf6*](http://www.ncbi.nlm.nih.gov/entrez/query.fcgi?db=gene&cmd=Retrieve&dopt=full_report&list_uids=77929) | Yip1 domain family, member 6 | 213 | 88 |
| mmu-miR-181c-5p | [*Ywhag*](http://www.ncbi.nlm.nih.gov/entrez/query.fcgi?db=gene&cmd=Retrieve&dopt=full_report&list_uids=22628) | tyrosine 3-monooxygenase/tryptophan 5-monooxygenase activation protein, gamma polypeptide | 214 | 88 |
| mmu-miR-181c-5p | [*Wdr82*](http://www.ncbi.nlm.nih.gov/entrez/query.fcgi?db=gene&cmd=Retrieve&dopt=full_report&list_uids=77305) | WD repeat domain containing 82 | 215 | 88 |
| mmu-miR-181c-5p | [*Jazf1*](http://www.ncbi.nlm.nih.gov/entrez/query.fcgi?db=gene&cmd=Retrieve&dopt=full_report&list_uids=231986) | JAZF zinc finger 1 | 216 | 88 |
| mmu-miR-181c-5p | [*Galnt16*](http://www.ncbi.nlm.nih.gov/entrez/query.fcgi?db=gene&cmd=Retrieve&dopt=full_report&list_uids=108760) | UDP-N-acetyl-alpha-D-galactosamine:polypeptide N-acetylgalactosaminyltransferase 16 | 217 | 88 |
| mmu-miR-181c-5p | [*Dock7*](http://www.ncbi.nlm.nih.gov/entrez/query.fcgi?db=gene&cmd=Retrieve&dopt=full_report&list_uids=67299) | dedicator of cytokinesis 7 | 218 | 88 |
| mmu-miR-181c-5p | [*Slc25a37*](http://www.ncbi.nlm.nih.gov/entrez/query.fcgi?db=gene&cmd=Retrieve&dopt=full_report&list_uids=67712) | solute carrier family 25, member 37 | 219 | 88 |
| mmu-miR-181c-5p | [*B3galt5*](http://www.ncbi.nlm.nih.gov/entrez/query.fcgi?db=gene&cmd=Retrieve&dopt=full_report&list_uids=93961) | UDP-Gal:betaGlcNAc beta 1,3-galactosyltransferase, polypeptide 5 | 220 | 88 |
| mmu-miR-181c-5p | [*4921524J17Rik*](http://www.ncbi.nlm.nih.gov/entrez/query.fcgi?db=gene&cmd=Retrieve&dopt=full_report&list_uids=66714) | RIKEN cDNA 4921524J17 gene | 221 | 88 |
| mmu-miR-181c-5p | [*Mfsd6*](http://www.ncbi.nlm.nih.gov/entrez/query.fcgi?db=gene&cmd=Retrieve&dopt=full_report&list_uids=98682) | major facilitator superfamily domain containing 6 | 222 | 88 |
| mmu-miR-181c-5p | [*Dlg2*](http://www.ncbi.nlm.nih.gov/entrez/query.fcgi?db=gene&cmd=Retrieve&dopt=full_report&list_uids=23859) | discs, large homolog 2 (Drosophila) | 223 | 88 |
| mmu-miR-181c-5p | [*Clec10a*](http://www.ncbi.nlm.nih.gov/entrez/query.fcgi?db=gene&cmd=Retrieve&dopt=full_report&list_uids=17312) | C-type lectin domain family 10, member A | 224 | 88 |
| mmu-miR-181c-5p | [*Creb1*](http://www.ncbi.nlm.nih.gov/entrez/query.fcgi?db=gene&cmd=Retrieve&dopt=full_report&list_uids=12912) | cAMP responsive element binding protein 1 | 225 | 88 |
| mmu-miR-181c-5p | [*Hsp90b1*](http://www.ncbi.nlm.nih.gov/entrez/query.fcgi?db=gene&cmd=Retrieve&dopt=full_report&list_uids=22027) | heat shock protein 90, beta (Grp94), member 1 | 226 | 88 |
| mmu-miR-181c-5p | [*Prrc2c*](http://www.ncbi.nlm.nih.gov/entrez/query.fcgi?db=gene&cmd=Retrieve&dopt=full_report&list_uids=226562) | proline-rich coiled-coil 2C | 227 | 88 |
| mmu-miR-181c-5p | [*Mtx3*](http://www.ncbi.nlm.nih.gov/entrez/query.fcgi?db=gene&cmd=Retrieve&dopt=full_report&list_uids=382793) | metaxin 3 | 228 | 88 |
| mmu-miR-181c-5p | [*Pdik1l*](http://www.ncbi.nlm.nih.gov/entrez/query.fcgi?db=gene&cmd=Retrieve&dopt=full_report&list_uids=230809) | PDLIM1 interacting kinase 1 like | 229 | 88 |
| mmu-miR-181c-5p | [*Cenpi*](http://www.ncbi.nlm.nih.gov/entrez/query.fcgi?db=gene&cmd=Retrieve&dopt=full_report&list_uids=102920) | centromere protein I | 230 | 88 |
| mmu-miR-181c-5p | [*Fam179b*](http://www.ncbi.nlm.nih.gov/entrez/query.fcgi?db=gene&cmd=Retrieve&dopt=full_report&list_uids=328108) | family with sequence similarity 179, member B | 231 | 88 |
| mmu-miR-181c-5p | [*Rad21l*](http://www.ncbi.nlm.nih.gov/entrez/query.fcgi?db=gene&cmd=Retrieve&dopt=full_report&list_uids=668929) | RAD21-like (S. pombe) | 232 | 88 |
| mmu-miR-181c-5p | [*Adamts1*](http://www.ncbi.nlm.nih.gov/entrez/query.fcgi?db=gene&cmd=Retrieve&dopt=full_report&list_uids=11504) | a disintegrin-like and metallopeptidase (reprolysin type) with thrombospondin type 1 motif, 1 | 233 | 87 |
| mmu-miR-181c-5p | [*Mfsd1*](http://www.ncbi.nlm.nih.gov/entrez/query.fcgi?db=gene&cmd=Retrieve&dopt=full_report&list_uids=66868) | major facilitator superfamily domain containing 1 | 234 | 87 |
| mmu-miR-181c-5p | [*Eif4a2*](http://www.ncbi.nlm.nih.gov/entrez/query.fcgi?db=gene&cmd=Retrieve&dopt=full_report&list_uids=13682) | eukaryotic translation initiation factor 4A2 | 235 | 87 |
| mmu-miR-181c-5p | [*Gigyf1*](http://www.ncbi.nlm.nih.gov/entrez/query.fcgi?db=gene&cmd=Retrieve&dopt=full_report&list_uids=57330) | GRB10 interacting GYF protein 1 | 236 | 87 |
| mmu-miR-181c-5p | [*G3bp2*](http://www.ncbi.nlm.nih.gov/entrez/query.fcgi?db=gene&cmd=Retrieve&dopt=full_report&list_uids=23881) | GTPase activating protein (SH3 domain) binding protein 2 | 237 | 87 |
| mmu-miR-181c-5p | [*Dynll2*](http://www.ncbi.nlm.nih.gov/entrez/query.fcgi?db=gene&cmd=Retrieve&dopt=full_report&list_uids=68097) | dynein light chain LC8-type 2 | 238 | 87 |
| mmu-miR-181c-5p | [*2310067B10Rik*](http://www.ncbi.nlm.nih.gov/entrez/query.fcgi?db=gene&cmd=Retrieve&dopt=full_report&list_uids=71947) | RIKEN cDNA 2310067B10 gene | 239 | 87 |
| mmu-miR-181c-5p | [*Rassf2*](http://www.ncbi.nlm.nih.gov/entrez/query.fcgi?db=gene&cmd=Retrieve&dopt=full_report&list_uids=215653) | Ras association (RalGDS/AF-6) domain family member 2 | 240 | 87 |
| mmu-miR-181c-5p | [*Adarb1*](http://www.ncbi.nlm.nih.gov/entrez/query.fcgi?db=gene&cmd=Retrieve&dopt=full_report&list_uids=110532) | adenosine deaminase, RNA-specific, B1 | 241 | 87 |
| mmu-miR-181c-5p | [*Mapk1*](http://www.ncbi.nlm.nih.gov/entrez/query.fcgi?db=gene&cmd=Retrieve&dopt=full_report&list_uids=26413) | mitogen-activated protein kinase 1 | 242 | 87 |
| mmu-miR-181c-5p | [*Pde5a*](http://www.ncbi.nlm.nih.gov/entrez/query.fcgi?db=gene&cmd=Retrieve&dopt=full_report&list_uids=242202) | phosphodiesterase 5A, cGMP-specific | 243 | 87 |
| mmu-miR-181c-5p | [*Lclat1*](http://www.ncbi.nlm.nih.gov/entrez/query.fcgi?db=gene&cmd=Retrieve&dopt=full_report&list_uids=225010) | lysocardiolipin acyltransferase 1 | 244 | 87 |
| mmu-miR-181c-5p | [*Kcnq5*](http://www.ncbi.nlm.nih.gov/entrez/query.fcgi?db=gene&cmd=Retrieve&dopt=full_report&list_uids=226922) | potassium voltage-gated channel, subfamily Q, member 5 | 245 | 87 |
| mmu-miR-181c-5p | [*Baz2b*](http://www.ncbi.nlm.nih.gov/entrez/query.fcgi?db=gene&cmd=Retrieve&dopt=full_report&list_uids=407823) | bromodomain adjacent to zinc finger domain, 2B | 246 | 86 |
| mmu-miR-181c-5p | [*Sim1*](http://www.ncbi.nlm.nih.gov/entrez/query.fcgi?db=gene&cmd=Retrieve&dopt=full_report&list_uids=20464) | single-minded homolog 1 (Drosophila) | 247 | 86 |
| mmu-miR-181c-5p | [*Nipal4*](http://www.ncbi.nlm.nih.gov/entrez/query.fcgi?db=gene&cmd=Retrieve&dopt=full_report&list_uids=214112) | NIPA-like domain containing 4 | 248 | 86 |
| mmu-miR-181c-5p | [*Ubp1*](http://www.ncbi.nlm.nih.gov/entrez/query.fcgi?db=gene&cmd=Retrieve&dopt=full_report&list_uids=22221) | upstream binding protein 1 | 249 | 86 |
| mmu-miR-181c-5p | [*Tbpl1*](http://www.ncbi.nlm.nih.gov/entrez/query.fcgi?db=gene&cmd=Retrieve&dopt=full_report&list_uids=237336) | TATA box binding protein-like 1 | 250 | 86 |
| mmu-miR-181c-5p | [*Pdgfra*](http://www.ncbi.nlm.nih.gov/entrez/query.fcgi?db=gene&cmd=Retrieve&dopt=full_report&list_uids=18595) | platelet derived growth factor receptor, alpha polypeptide | 251 | 86 |
| mmu-miR-181c-5p | [*Hoxa1*](http://www.ncbi.nlm.nih.gov/entrez/query.fcgi?db=gene&cmd=Retrieve&dopt=full_report&list_uids=15394) | homeobox A1 | 252 | 86 |
| mmu-miR-181c-5p | [*Hhip*](http://www.ncbi.nlm.nih.gov/entrez/query.fcgi?db=gene&cmd=Retrieve&dopt=full_report&list_uids=15245) | Hedgehog-interacting protein | 253 | 86 |
| mmu-miR-181c-5p | [*Mlf1*](http://www.ncbi.nlm.nih.gov/entrez/query.fcgi?db=gene&cmd=Retrieve&dopt=full_report&list_uids=17349) | myeloid leukemia factor 1 | 254 | 86 |
| mmu-miR-181c-5p | [*Golga1*](http://www.ncbi.nlm.nih.gov/entrez/query.fcgi?db=gene&cmd=Retrieve&dopt=full_report&list_uids=76899) | golgi autoantigen, golgin subfamily a, 1 | 255 | 86 |
| mmu-miR-181c-5p | [*Zbtb4*](http://www.ncbi.nlm.nih.gov/entrez/query.fcgi?db=gene&cmd=Retrieve&dopt=full_report&list_uids=75580) | zinc finger and BTB domain containing 4 | 256 | 86 |
| mmu-miR-181c-5p | [*Zbtb43*](http://www.ncbi.nlm.nih.gov/entrez/query.fcgi?db=gene&cmd=Retrieve&dopt=full_report&list_uids=71834) | zinc finger and BTB domain containing 43 | 257 | 86 |
| mmu-miR-181c-5p | [*Ehd4*](http://www.ncbi.nlm.nih.gov/entrez/query.fcgi?db=gene&cmd=Retrieve&dopt=full_report&list_uids=98878) | EH-domain containing 4 | 258 | 86 |
| mmu-miR-181c-5p | [*Mboat2*](http://www.ncbi.nlm.nih.gov/entrez/query.fcgi?db=gene&cmd=Retrieve&dopt=full_report&list_uids=67216) | membrane bound O-acyltransferase domain containing 2 | 259 | 86 |
| mmu-miR-181c-5p | [*Nr3c1*](http://www.ncbi.nlm.nih.gov/entrez/query.fcgi?db=gene&cmd=Retrieve&dopt=full_report&list_uids=14815) | nuclear receptor subfamily 3, group C, member 1 | 260 | 85 |
| mmu-miR-181c-5p | [*Mybl1*](http://www.ncbi.nlm.nih.gov/entrez/query.fcgi?db=gene&cmd=Retrieve&dopt=full_report&list_uids=17864) | myeloblastosis oncogene-like 1 | 261 | 85 |
| mmu-miR-181c-5p | [*Api5*](http://www.ncbi.nlm.nih.gov/entrez/query.fcgi?db=gene&cmd=Retrieve&dopt=full_report&list_uids=11800) | apoptosis inhibitor 5 | 262 | 85 |
| mmu-miR-181c-5p | [*Fam83b*](http://www.ncbi.nlm.nih.gov/entrez/query.fcgi?db=gene&cmd=Retrieve&dopt=full_report&list_uids=208994) | family with sequence similarity 83, member B | 263 | 85 |
| mmu-miR-181c-5p | [*Klhl42*](http://www.ncbi.nlm.nih.gov/entrez/query.fcgi?db=gene&cmd=Retrieve&dopt=full_report&list_uids=232539) | kelch-like 42 | 264 | 85 |
| mmu-miR-181c-5p | [*Ncoa2*](http://www.ncbi.nlm.nih.gov/entrez/query.fcgi?db=gene&cmd=Retrieve&dopt=full_report&list_uids=17978) | nuclear receptor coactivator 2 | 265 | 85 |
| mmu-miR-181c-5p | [*Wasl*](http://www.ncbi.nlm.nih.gov/entrez/query.fcgi?db=gene&cmd=Retrieve&dopt=full_report&list_uids=73178) | Wiskott-Aldrich syndrome-like (human) | 266 | 85 |
| mmu-miR-181c-5p | [*Zfp825*](http://www.ncbi.nlm.nih.gov/entrez/query.fcgi?db=gene&cmd=Retrieve&dopt=full_report&list_uids=235956) | zinc finger protein 825 | 267 | 85 |
| mmu-miR-181c-5p | [*AF529169*](http://www.ncbi.nlm.nih.gov/entrez/query.fcgi?db=gene&cmd=Retrieve&dopt=full_report&list_uids=209743) | cDNA sequence AF529169 | 268 | 85 |
| mmu-miR-181c-5p | [*Prnd*](http://www.ncbi.nlm.nih.gov/entrez/query.fcgi?db=gene&cmd=Retrieve&dopt=full_report&list_uids=26434) | prion protein dublet | 269 | 85 |
| mmu-miR-181c-5p | [*Wdr7*](http://www.ncbi.nlm.nih.gov/entrez/query.fcgi?db=gene&cmd=Retrieve&dopt=full_report&list_uids=104082) | WD repeat domain 7 | 270 | 85 |
| mmu-miR-181c-5p | [*E2f7*](http://www.ncbi.nlm.nih.gov/entrez/query.fcgi?db=gene&cmd=Retrieve&dopt=full_report&list_uids=52679) | E2F transcription factor 7 | 271 | 85 |
| mmu-miR-181c-5p | [*Slc2a3*](http://www.ncbi.nlm.nih.gov/entrez/query.fcgi?db=gene&cmd=Retrieve&dopt=full_report&list_uids=20527) | solute carrier family 2 (facilitated glucose transporter), member 3 | 272 | 85 |
| mmu-miR-181c-5p | [*Ppp1r3c*](http://www.ncbi.nlm.nih.gov/entrez/query.fcgi?db=gene&cmd=Retrieve&dopt=full_report&list_uids=53412) | protein phosphatase 1, regulatory (inhibitor) subunit 3C | 273 | 85 |
| mmu-miR-181c-5p | [*Slc35f3*](http://www.ncbi.nlm.nih.gov/entrez/query.fcgi?db=gene&cmd=Retrieve&dopt=full_report&list_uids=210027) | solute carrier family 35, member F3 | 274 | 85 |
| mmu-miR-181c-5p | [*Ept1*](http://www.ncbi.nlm.nih.gov/entrez/query.fcgi?db=gene&cmd=Retrieve&dopt=full_report&list_uids=28042) | ethanolaminephosphotransferase 1 (CDP-ethanolamine-specific) | 275 | 85 |
| mmu-miR-181c-5p | [*Kmt2c*](http://www.ncbi.nlm.nih.gov/entrez/query.fcgi?db=gene&cmd=Retrieve&dopt=full_report&list_uids=231051) | lysine (K)-specific methyltransferase 2C | 276 | 85 |
| mmu-miR-181c-5p | [*Gm21949*](http://www.ncbi.nlm.nih.gov/entrez/query.fcgi?db=gene&cmd=Retrieve&dopt=full_report&list_uids=100505386) | predicted gene, 21949 | 277 | 84 |
| mmu-miR-181c-5p | [*Grik3*](http://www.ncbi.nlm.nih.gov/entrez/query.fcgi?db=gene&cmd=Retrieve&dopt=full_report&list_uids=14807) | glutamate receptor, ionotropic, kainate 3 | 278 | 84 |
| mmu-miR-181c-5p | [*Mkrn1*](http://www.ncbi.nlm.nih.gov/entrez/query.fcgi?db=gene&cmd=Retrieve&dopt=full_report&list_uids=54484) | makorin, ring finger protein, 1 | 279 | 84 |
| mmu-miR-181c-5p | [*Trim2*](http://www.ncbi.nlm.nih.gov/entrez/query.fcgi?db=gene&cmd=Retrieve&dopt=full_report&list_uids=80890) | tripartite motif-containing 2 | 280 | 84 |
| mmu-miR-181c-5p | [*Zfand6*](http://www.ncbi.nlm.nih.gov/entrez/query.fcgi?db=gene&cmd=Retrieve&dopt=full_report&list_uids=65098) | zinc finger, AN1-type domain 6 | 281 | 84 |
| mmu-miR-181c-5p | [*Zfp869*](http://www.ncbi.nlm.nih.gov/entrez/query.fcgi?db=gene&cmd=Retrieve&dopt=full_report&list_uids=66869) | zinc finger protein 869 | 282 | 84 |
| mmu-miR-181c-5p | [*Igf2bp2*](http://www.ncbi.nlm.nih.gov/entrez/query.fcgi?db=gene&cmd=Retrieve&dopt=full_report&list_uids=319765) | insulin-like growth factor 2 mRNA binding protein 2 | 283 | 84 |
| mmu-miR-181c-5p | [*Tsc22d2*](http://www.ncbi.nlm.nih.gov/entrez/query.fcgi?db=gene&cmd=Retrieve&dopt=full_report&list_uids=72033) | TSC22 domain family, member 2 | 284 | 84 |
| mmu-miR-181c-5p | [*Ccnk*](http://www.ncbi.nlm.nih.gov/entrez/query.fcgi?db=gene&cmd=Retrieve&dopt=full_report&list_uids=12454) | cyclin K | 285 | 84 |
| mmu-miR-181c-5p | [*Timp3*](http://www.ncbi.nlm.nih.gov/entrez/query.fcgi?db=gene&cmd=Retrieve&dopt=full_report&list_uids=21859) | tissue inhibitor of metalloproteinase 3 | 286 | 84 |
| mmu-miR-181c-5p | [*Sep-08*](http://www.ncbi.nlm.nih.gov/entrez/query.fcgi?db=gene&cmd=Retrieve&dopt=full_report&list_uids=20362) | septin 8 | 287 | 84 |
| mmu-miR-181c-5p | [*Cysltr1*](http://www.ncbi.nlm.nih.gov/entrez/query.fcgi?db=gene&cmd=Retrieve&dopt=full_report&list_uids=58861) | cysteinyl leukotriene receptor 1 | 288 | 84 |
| mmu-miR-181c-5p | [*Zdhhc7*](http://www.ncbi.nlm.nih.gov/entrez/query.fcgi?db=gene&cmd=Retrieve&dopt=full_report&list_uids=102193) | zinc finger, DHHC domain containing 7 | 289 | 84 |
| mmu-miR-181c-5p | [*Psap*](http://www.ncbi.nlm.nih.gov/entrez/query.fcgi?db=gene&cmd=Retrieve&dopt=full_report&list_uids=19156) | prosaposin | 290 | 84 |
| mmu-miR-181c-5p | [*Etnk1*](http://www.ncbi.nlm.nih.gov/entrez/query.fcgi?db=gene&cmd=Retrieve&dopt=full_report&list_uids=75320) | ethanolamine kinase 1 | 291 | 84 |
| mmu-miR-181c-5p | [*Phactr1*](http://www.ncbi.nlm.nih.gov/entrez/query.fcgi?db=gene&cmd=Retrieve&dopt=full_report&list_uids=218194) | phosphatase and actin regulator 1 | 292 | 84 |
| mmu-miR-181c-5p | [*Chrm3*](http://www.ncbi.nlm.nih.gov/entrez/query.fcgi?db=gene&cmd=Retrieve&dopt=full_report&list_uids=12671) | cholinergic receptor, muscarinic 3, cardiac | 293 | 84 |
| mmu-miR-181c-5p | [*Elavl4*](http://www.ncbi.nlm.nih.gov/entrez/query.fcgi?db=gene&cmd=Retrieve&dopt=full_report&list_uids=15572) | ELAV (embryonic lethal, abnormal vision, Drosophila)-like 4 (Hu antigen D) | 294 | 84 |
| mmu-miR-181c-5p | [*Sowaha*](http://www.ncbi.nlm.nih.gov/entrez/query.fcgi?db=gene&cmd=Retrieve&dopt=full_report&list_uids=237761) | sosondowah ankyrin repeat domain family member A | 295 | 84 |
| mmu-miR-181c-5p | [*Ube3c*](http://www.ncbi.nlm.nih.gov/entrez/query.fcgi?db=gene&cmd=Retrieve&dopt=full_report&list_uids=100763) | ubiquitin protein ligase E3C | 296 | 84 |
| mmu-miR-181c-5p | [*Ino80*](http://www.ncbi.nlm.nih.gov/entrez/query.fcgi?db=gene&cmd=Retrieve&dopt=full_report&list_uids=68142) | INO80 homolog (S. cerevisiae) | 297 | 83 |
| mmu-miR-181c-5p | [*Nfat5*](http://www.ncbi.nlm.nih.gov/entrez/query.fcgi?db=gene&cmd=Retrieve&dopt=full_report&list_uids=54446) | nuclear factor of activated T cells 5 | 298 | 83 |
| mmu-miR-181c-5p | [*Ppp1r2*](http://www.ncbi.nlm.nih.gov/entrez/query.fcgi?db=gene&cmd=Retrieve&dopt=full_report&list_uids=66849) | protein phosphatase 1, regulatory (inhibitor) subunit 2 | 299 | 83 |
| mmu-miR-181c-5p | [*Mtpn*](http://www.ncbi.nlm.nih.gov/entrez/query.fcgi?db=gene&cmd=Retrieve&dopt=full_report&list_uids=14489) | myotrophin | 300 | 83 |
| mmu-miR-181c-5p | [*Abtb2*](http://www.ncbi.nlm.nih.gov/entrez/query.fcgi?db=gene&cmd=Retrieve&dopt=full_report&list_uids=99382) | ankyrin repeat and BTB (POZ) domain containing 2 | 301 | 83 |
| mmu-miR-181c-5p | [*Ssr1*](http://www.ncbi.nlm.nih.gov/entrez/query.fcgi?db=gene&cmd=Retrieve&dopt=full_report&list_uids=107513) | signal sequence receptor, alpha | 302 | 83 |
| mmu-miR-181c-5p | [*Cdh8*](http://www.ncbi.nlm.nih.gov/entrez/query.fcgi?db=gene&cmd=Retrieve&dopt=full_report&list_uids=12564) | cadherin 8 | 303 | 83 |
| mmu-miR-181c-5p | [*9830147E19Rik*](http://www.ncbi.nlm.nih.gov/entrez/query.fcgi?db=gene&cmd=Retrieve&dopt=full_report&list_uids=208111) | RIKEN cDNA 9830147E19 gene | 304 | 83 |
| mmu-miR-181c-5p | [*Bai3*](http://www.ncbi.nlm.nih.gov/entrez/query.fcgi?db=gene&cmd=Retrieve&dopt=full_report&list_uids=210933) | brain-specific angiogenesis inhibitor 3 | 305 | 83 |
| mmu-miR-181c-5p | [*Rras2*](http://www.ncbi.nlm.nih.gov/entrez/query.fcgi?db=gene&cmd=Retrieve&dopt=full_report&list_uids=66922) | related RAS viral (r-ras) oncogene homolog 2 | 306 | 83 |
| mmu-miR-181c-5p | [*Tmem151b*](http://www.ncbi.nlm.nih.gov/entrez/query.fcgi?db=gene&cmd=Retrieve&dopt=full_report&list_uids=210573) | transmembrane protein 151B | 307 | 83 |
| mmu-miR-181c-5p | [*Plcl2*](http://www.ncbi.nlm.nih.gov/entrez/query.fcgi?db=gene&cmd=Retrieve&dopt=full_report&list_uids=224860) | phospholipase C-like 2 | 308 | 83 |
| mmu-miR-181c-5p | [*Synpr*](http://www.ncbi.nlm.nih.gov/entrez/query.fcgi?db=gene&cmd=Retrieve&dopt=full_report&list_uids=72003) | synaptoporin | 309 | 83 |
| mmu-miR-181c-5p | [*Tnfaip1*](http://www.ncbi.nlm.nih.gov/entrez/query.fcgi?db=gene&cmd=Retrieve&dopt=full_report&list_uids=21927) | tumor necrosis factor, alpha-induced protein 1 (endothelial) | 310 | 82 |
| mmu-miR-181c-5p | [*Cacnb2*](http://www.ncbi.nlm.nih.gov/entrez/query.fcgi?db=gene&cmd=Retrieve&dopt=full_report&list_uids=12296) | calcium channel, voltage-dependent, beta 2 subunit | 311 | 82 |
| mmu-miR-181c-5p | [*Nptxr*](http://www.ncbi.nlm.nih.gov/entrez/query.fcgi?db=gene&cmd=Retrieve&dopt=full_report&list_uids=73340) | neuronal pentraxin receptor | 312 | 82 |
| mmu-miR-181c-5p | [*Pam*](http://www.ncbi.nlm.nih.gov/entrez/query.fcgi?db=gene&cmd=Retrieve&dopt=full_report&list_uids=18484) | peptidylglycine alpha-amidating monooxygenase | 313 | 82 |
| mmu-miR-181c-5p | [*Trnau1ap*](http://www.ncbi.nlm.nih.gov/entrez/query.fcgi?db=gene&cmd=Retrieve&dopt=full_report&list_uids=71787) | tRNA selenocysteine 1 associated protein 1 | 314 | 82 |
| mmu-miR-181c-5p | [*Rspo2*](http://www.ncbi.nlm.nih.gov/entrez/query.fcgi?db=gene&cmd=Retrieve&dopt=full_report&list_uids=239405) | R-spondin 2 homolog (Xenopus laevis) | 315 | 82 |
| mmu-miR-181c-5p | [*Dip2c*](http://www.ncbi.nlm.nih.gov/entrez/query.fcgi?db=gene&cmd=Retrieve&dopt=full_report&list_uids=208440) | DIP2 disco-interacting protein 2 homolog C (Drosophila) | 316 | 82 |
| mmu-miR-181c-5p | [*Atxn3*](http://www.ncbi.nlm.nih.gov/entrez/query.fcgi?db=gene&cmd=Retrieve&dopt=full_report&list_uids=110616) | ataxin 3 | 317 | 82 |
| mmu-miR-181c-5p | [*Ppp1r12b*](http://www.ncbi.nlm.nih.gov/entrez/query.fcgi?db=gene&cmd=Retrieve&dopt=full_report&list_uids=329251) | protein phosphatase 1, regulatory (inhibitor) subunit 12B | 318 | 82 |
| mmu-miR-181c-5p | [*Proser1*](http://www.ncbi.nlm.nih.gov/entrez/query.fcgi?db=gene&cmd=Retrieve&dopt=full_report&list_uids=212127) | proline and serine rich 1 | 319 | 82 |
| mmu-miR-181c-5p | [*Zfp317*](http://www.ncbi.nlm.nih.gov/entrez/query.fcgi?db=gene&cmd=Retrieve&dopt=full_report&list_uids=244713) | zinc finger protein 317 | 320 | 82 |
| mmu-miR-181c-5p | [*Dennd1b*](http://www.ncbi.nlm.nih.gov/entrez/query.fcgi?db=gene&cmd=Retrieve&dopt=full_report&list_uids=329260) | DENN/MADD domain containing 1B | 321 | 82 |
| mmu-miR-181c-5p | [*Cecr2*](http://www.ncbi.nlm.nih.gov/entrez/query.fcgi?db=gene&cmd=Retrieve&dopt=full_report&list_uids=330409) | cat eye syndrome chromosome region, candidate 2 | 322 | 82 |
| mmu-miR-181c-5p | [*Heca*](http://www.ncbi.nlm.nih.gov/entrez/query.fcgi?db=gene&cmd=Retrieve&dopt=full_report&list_uids=380629) | headcase homolog (Drosophila) | 323 | 82 |
| mmu-miR-181c-5p | [*Pbx1*](http://www.ncbi.nlm.nih.gov/entrez/query.fcgi?db=gene&cmd=Retrieve&dopt=full_report&list_uids=18514) | pre B cell leukemia homeobox 1 | 324 | 82 |
| mmu-miR-181c-5p | [*Slc9a3*](http://www.ncbi.nlm.nih.gov/entrez/query.fcgi?db=gene&cmd=Retrieve&dopt=full_report&list_uids=105243) | solute carrier family 9 (sodium/hydrogen exchanger), member 3 | 325 | 82 |
| mmu-miR-181c-5p | [*Ankmy2*](http://www.ncbi.nlm.nih.gov/entrez/query.fcgi?db=gene&cmd=Retrieve&dopt=full_report&list_uids=217473) | ankyrin repeat and MYND domain containing 2 | 326 | 82 |
| mmu-miR-181c-5p | [*Zfp212*](http://www.ncbi.nlm.nih.gov/entrez/query.fcgi?db=gene&cmd=Retrieve&dopt=full_report&list_uids=232784) | Zinc finger protein 212 | 327 | 82 |
| mmu-miR-181c-5p | [*Hic2*](http://www.ncbi.nlm.nih.gov/entrez/query.fcgi?db=gene&cmd=Retrieve&dopt=full_report&list_uids=58180) | hypermethylated in cancer 2 | 328 | 82 |
| mmu-miR-181c-5p | [*Msi2*](http://www.ncbi.nlm.nih.gov/entrez/query.fcgi?db=gene&cmd=Retrieve&dopt=full_report&list_uids=76626) | musashi RNA-binding protein 2 | 329 | 81 |
| mmu-miR-181c-5p | [*Pbx3*](http://www.ncbi.nlm.nih.gov/entrez/query.fcgi?db=gene&cmd=Retrieve&dopt=full_report&list_uids=18516) | pre B cell leukemia homeobox 3 | 330 | 81 |
| mmu-miR-181c-5p | [*Ano1*](http://www.ncbi.nlm.nih.gov/entrez/query.fcgi?db=gene&cmd=Retrieve&dopt=full_report&list_uids=101772) | anoctamin 1, calcium activated chloride channel | 331 | 81 |
| mmu-miR-181c-5p | [*Sin3b*](http://www.ncbi.nlm.nih.gov/entrez/query.fcgi?db=gene&cmd=Retrieve&dopt=full_report&list_uids=20467) | transcriptional regulator, SIN3B (yeast) | 332 | 81 |
| mmu-miR-181c-5p | [*Zdhhc17*](http://www.ncbi.nlm.nih.gov/entrez/query.fcgi?db=gene&cmd=Retrieve&dopt=full_report&list_uids=320150) | zinc finger, DHHC domain containing 17 | 333 | 81 |
| mmu-miR-181c-5p | [*Fndc3a*](http://www.ncbi.nlm.nih.gov/entrez/query.fcgi?db=gene&cmd=Retrieve&dopt=full_report&list_uids=319448) | fibronectin type III domain containing 3A | 334 | 81 |
| mmu-miR-181c-5p | [*Med12l*](http://www.ncbi.nlm.nih.gov/entrez/query.fcgi?db=gene&cmd=Retrieve&dopt=full_report&list_uids=329650) | mediator complex subunit 12-like | 335 | 81 |
| mmu-miR-181c-5p | [*Nlk*](http://www.ncbi.nlm.nih.gov/entrez/query.fcgi?db=gene&cmd=Retrieve&dopt=full_report&list_uids=18099) | nemo like kinase | 336 | 81 |
| mmu-miR-181c-5p | [*Ankrd27*](http://www.ncbi.nlm.nih.gov/entrez/query.fcgi?db=gene&cmd=Retrieve&dopt=full_report&list_uids=245886) | ankyrin repeat domain 27 (VPS9 domain) | 337 | 81 |
| mmu-miR-181c-5p | [*Foxp1*](http://www.ncbi.nlm.nih.gov/entrez/query.fcgi?db=gene&cmd=Retrieve&dopt=full_report&list_uids=108655) | forkhead box P1 | 338 | 81 |
| mmu-miR-181c-5p | [*Gpx8*](http://www.ncbi.nlm.nih.gov/entrez/query.fcgi?db=gene&cmd=Retrieve&dopt=full_report&list_uids=69590) | glutathione peroxidase 8 (putative) | 339 | 81 |
| mmu-miR-181c-5p | [*Diap1*](http://www.ncbi.nlm.nih.gov/entrez/query.fcgi?db=gene&cmd=Retrieve&dopt=full_report&list_uids=13367) | diaphanous homolog 1 (Drosophila) | 340 | 81 |
| mmu-miR-181c-5p | [*Glrb*](http://www.ncbi.nlm.nih.gov/entrez/query.fcgi?db=gene&cmd=Retrieve&dopt=full_report&list_uids=14658) | glycine receptor, beta subunit | 341 | 81 |
| mmu-miR-181c-5p | [*Rassf8*](http://www.ncbi.nlm.nih.gov/entrez/query.fcgi?db=gene&cmd=Retrieve&dopt=full_report&list_uids=71323) | Ras association (RalGDS/AF-6) domain family (N-terminal) member 8 | 342 | 81 |
| mmu-miR-181c-5p | [*Uri1*](http://www.ncbi.nlm.nih.gov/entrez/query.fcgi?db=gene&cmd=Retrieve&dopt=full_report&list_uids=19777) | URI1, prefoldin-like chaperone | 343 | 81 |
| mmu-miR-181c-5p | [*Cbx4*](http://www.ncbi.nlm.nih.gov/entrez/query.fcgi?db=gene&cmd=Retrieve&dopt=full_report&list_uids=12418) | chromobox 4 | 344 | 80 |
| mmu-miR-181c-5p | [*Itga2*](http://www.ncbi.nlm.nih.gov/entrez/query.fcgi?db=gene&cmd=Retrieve&dopt=full_report&list_uids=16398) | integrin alpha 2 | 345 | 80 |
| mmu-miR-181c-5p | [*Plau*](http://www.ncbi.nlm.nih.gov/entrez/query.fcgi?db=gene&cmd=Retrieve&dopt=full_report&list_uids=18792) | plasminogen activator, urokinase | 346 | 80 |
| mmu-miR-181c-5p | [*Fam83a*](http://www.ncbi.nlm.nih.gov/entrez/query.fcgi?db=gene&cmd=Retrieve&dopt=full_report&list_uids=239463) | family with sequence similarity 83, member A | 347 | 80 |
| mmu-miR-181c-5p | [*Pcnp*](http://www.ncbi.nlm.nih.gov/entrez/query.fcgi?db=gene&cmd=Retrieve&dopt=full_report&list_uids=76302) | PEST proteolytic signal containing nuclear protein | 348 | 80 |
| mmu-miR-181c-5p | [*Syncrip*](http://www.ncbi.nlm.nih.gov/entrez/query.fcgi?db=gene&cmd=Retrieve&dopt=full_report&list_uids=56403) | synaptotagmin binding, cytoplasmic RNA interacting protein | 349 | 80 |
| mmu-miR-181c-5p | [*Rlim*](http://www.ncbi.nlm.nih.gov/entrez/query.fcgi?db=gene&cmd=Retrieve&dopt=full_report&list_uids=19820) | ring finger protein, LIM domain interacting | 350 | 80 |
| mmu-miR-181c-5p | [*Asah2*](http://www.ncbi.nlm.nih.gov/entrez/query.fcgi?db=gene&cmd=Retrieve&dopt=full_report&list_uids=54447) | N-acylsphingosine amidohydrolase 2 | 351 | 80 |
| mmu-miR-181c-5p | [*Ankrd44*](http://www.ncbi.nlm.nih.gov/entrez/query.fcgi?db=gene&cmd=Retrieve&dopt=full_report&list_uids=329154) | ankyrin repeat domain 44 | 352 | 80 |
| mmu-miR-181c-5p | [*Cpsf6*](http://www.ncbi.nlm.nih.gov/entrez/query.fcgi?db=gene&cmd=Retrieve&dopt=full_report&list_uids=432508) | cleavage and polyadenylation specific factor 6 | 353 | 80 |
| mmu-miR-181c-5p | [*Lats1*](http://www.ncbi.nlm.nih.gov/entrez/query.fcgi?db=gene&cmd=Retrieve&dopt=full_report&list_uids=16798) | large tumor suppressor | 354 | 80 |
| mmu-miR-181c-5p | [*Agfg1*](http://www.ncbi.nlm.nih.gov/entrez/query.fcgi?db=gene&cmd=Retrieve&dopt=full_report&list_uids=15463) | ArfGAP with FG repeats 1 | 355 | 80 |
| mmu-miR-181c-5p | [*Metap1*](http://www.ncbi.nlm.nih.gov/entrez/query.fcgi?db=gene&cmd=Retrieve&dopt=full_report&list_uids=75624) | methionyl aminopeptidase 1 | 356 | 80 |
| mmu-miR-181c-5p | [*Tgfbi*](http://www.ncbi.nlm.nih.gov/entrez/query.fcgi?db=gene&cmd=Retrieve&dopt=full_report&list_uids=21810) | transforming growth factor, beta induced | 357 | 80 |
| mmu-miR-181c-5p | [*Sfr1*](http://www.ncbi.nlm.nih.gov/entrez/query.fcgi?db=gene&cmd=Retrieve&dopt=full_report&list_uids=67788) | SWI5 dependent recombination repair 1 | 358 | 80 |
| mmu-miR-181c-5p | [*Kcnh1*](http://www.ncbi.nlm.nih.gov/entrez/query.fcgi?db=gene&cmd=Retrieve&dopt=full_report&list_uids=16510) | potassium voltage-gated channel, subfamily H (eag-related), member 1 | 359 | 80 |
| mmu-miR-181c-5p | [*Akirin1*](http://www.ncbi.nlm.nih.gov/entrez/query.fcgi?db=gene&cmd=Retrieve&dopt=full_report&list_uids=68050) | akirin 1 | 360 | 80 |
| mmu-miR-181c-5p | [*Aftph*](http://www.ncbi.nlm.nih.gov/entrez/query.fcgi?db=gene&cmd=Retrieve&dopt=full_report&list_uids=216549) | aftiphilin | 361 | 80 |
| mmu-miR-181c-5p | [*Pi15*](http://www.ncbi.nlm.nih.gov/entrez/query.fcgi?db=gene&cmd=Retrieve&dopt=full_report&list_uids=94227) | peptidase inhibitor 15 | 362 | 80 |
| mmu-miR-181c-5p | [*Evx1*](http://www.ncbi.nlm.nih.gov/entrez/query.fcgi?db=gene&cmd=Retrieve&dopt=full_report&list_uids=14028) | even skipped homeotic gene 1 homolog | 363 | 80 |
| mmu-miR-181c-5p | [*Slc18a2*](http://www.ncbi.nlm.nih.gov/entrez/query.fcgi?db=gene&cmd=Retrieve&dopt=full_report&list_uids=214084) | solute carrier family 18 (vesicular monoamine), member 2 | 364 | 80 |
