# Supplementary material for: The small non-coding RNA profile of mouse oocytes is modified during aging
Source: Aging (Albany NY). 2019 May 24;11(10):2968–97. doi: 10.18632/aging.101947 (PMC6555462; doi:10.18632/aging.101947)
Supplement: Supplementary Table 6 [file aging-11-101947-s007.pdf]

Supplementary Figures

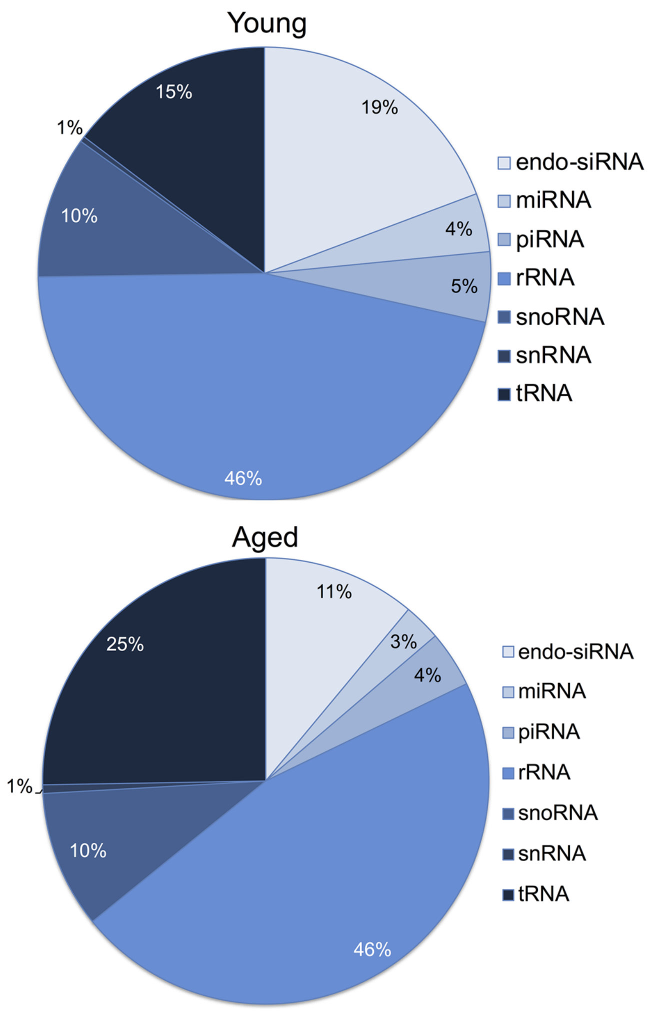

Supplementary Figure S1. The small RNA signatures of young and aged oocytes. Following filtering and normalisation, sncRNA reads were mapped to known and miRNA, endo-siRNA, piRNA, rRNA, snoRNA, snRNA, and tRNA fragments from RNAcentral. Pie chart was generated to illustrate the number of total sequences aligning to each small RNA category.

5' UTR

```
KIFC1  ----------------------------ATTCAAAAGTGGCGGGTGTGGCTGATTGGCCT 32
KIFC5B GAAGTGCGTTGACTGCGCGGAGGTTGAAATTCAAAAGTGACTGGTGTGGCGGATTGGCTT 60
                                   *********** * ******** ******* *

KIFC1  GGCTCCTGGA----------CAGCCTGGCTTCGGCGTTTTGCGGCGCGAGCCCTTGAGAG 82
KIFC5B GGCCGATTGGCCTGGCGAATTGGCCTGGCTCCGACGTTTTGCGGCGCGAGCCCTTGAGAG 120
       ***   * *             ******** ** **************************

KIFC1  GACGCGGGTCTCATCGTCCTGCCTGCTGCCTTTGCACC-CTTTCTGTGCGGACCACCATG 141
KIFC5B GACGCGGGTCTCGTCACCCTGCCTGCTGCCTTTGCATCCCTTCCTGTGCGGACCACCATG 180
       ************ **  ******************* * *** *****************

KIFC1  GACGTGCAGGCGCAGAGGCCACCTTTGTTGGAAGTGAAGAGGAACGTAGAACTGAAGGCA 201
KIFC5B GACGTGCAGGCGCAGAGAAAG---GGAAGGGAAGGGAAGAGGAACGTAGAACTGAAGGCG 237
       *****************            ***** ************************

KIFC1  GCCCTGGTGAAGTCCTCCTCCCGAGTGCCCCTGTCAGCAAGCAGGCTCAAGAGGGGTCCT 261
KIFC5B GCCCTGGTCAAGTCCTCCTCCCGACTGCCCCTGTCAGCAAGCAGCCTCAAGAGGGGTCCT 297
       ******** *************** ******************* ***************

KIFC1  GACCAGATGGAGGATGCCTTGGAGCCTGCAAAGAAACGGACACGAGTCATGGGTGCAGTG 321
KIFC5B GACCAGATGGAGGATGCCTTGGAGCCTGCAAAGAAACGGACACGAGTCATGGGTGCAGTG 357
       ************************************************************

KIFC1  ACCAAAGTTGACACATCCCGTCCCAGAGGACCACTCCTCAGCACAGTGTCACAGACCCAG 381
KIFC5B ACCAAAGTTGACACATCCCGTCCCAGAGGACCACTCCTCAGCACAGTGTCACAGACCCAG 417
       ************************************************************

KIFC1  GGCCACACTGCAGCTCAGAAAGGCCCTAAGAAGACAGGACCTCGTGGGTGCTCTGCTATT 441
KIFC5B GGCCACAATGCAGCTCAGAAAGGCCCTAAGAAGACAGGACCTGGTGGGTGCTCTGCTGTT 477
       ******* ********************************** ************** **

KIFC1  GGTACAGTGCTGAGGAGCCAGAAGCCAGTTCCCGCTGCTCCTGCCCAGAAGCCTGGCACA 501
KIFC5B GGTACAGTGCTGAGGAGCCAGAAGCCAGCTCCCGCTGCTCCTGC---GAAGCCTGGCACA 534
       **************************** ***************   *************

KIFC1  TCCACTGCTCCTGTGGTGGTAGGGAAGAGAGCTGGCAAACGCCCTGCCTGGGACCTGAAG 561
KIFC5B TCCACTGCTCCTGTGGTGGTAGGGAAGAGAGCTGGCAAACGCCCTGCCTGGGACCTGAAG 594
       ************************************************************

KIFC1  GGCCAGTTGTGTGACCTCAATGAAGAGTTGAAACGCTATCGGGAGAAGACTCAAACGCTG 621
KIFC5B GGCCAGTTGTGTGACCTCAATGAAGAGTTGAAACGCTATCGGGAGAAGACTGAAACGCTG 654
       *************************************************** ********

KIFC1  GAACTGGAGAACCGGGGTCTTCGGGAGCAACTCAGAGAGGTCCAGGAGCAGGCCACGACC 681
KIFC5B GAACTGGAGAACCGGGGTCTTCGGGAGCAACTCAGAGAGGTCCAGGAGCAGGCCACGACC 714
       ************************************************************

KIFC1  CTGGGGACAGAGCGGAACACCCTGGAAGGGGAGCTGGCCAGTGTACGCAGCCGAGCTGAG 741
KIFC5B CTGGGGACAGAGCGGAACACCCTGGAAGGGGAGCTGGCCAGTGTACGCAGCCGAGCTGAG 774
       ************************************************************

KIFC1  CAGGACCAGCAGAGGCTGGAGACGCTGAGTGCCCGTGTCTTGGAGCTGGAGGAATGTCTG 801
KIFC5B CAGGACCAGCAGAGGCTGGAGACGCTGAGTGCCCGTGTCTTGGAGCTGGAGGAATGTCTG 834
       ************************************************************

KIFC1  GGTACCAGGGAAAGGCTGCTTCAGGAGCTTCAGGGAGAGCGGCTGCAATTGCAGGAGGAG 861
KIFC5B GGTACCAGGGAAAGGCTGCTTCAGGAGCTTCAGGGAGAGCGGCTGCAATTGCAGGAGGAG 894
       ************************************************************

KIFC1  CGGAGCACACTGAGCACCCAGCTGGAGGAGCAGGAGAGGAGGTTTCAGGCCACAGAAGCA 921
KIFC5B CGGAGCACACTGAGCACCCAACTGGAGGAGCAGGAGAGGAGGTTTCAGGCCACAGAAGCA 954
       ******************** ***************************************

KIFC1  GCTCTGTCAAGCAGCCAAGAAGAGGTGGTGTGTCTTCGGCAGAAGACTGAAGCCCAGGTG 981
KIFC5B GCTCTGTCAAGCAGCCAAGAAGAGGTGGTGTGTCTTCGGCAGAAGACTGAAGCCCAGGTG 1014
       ************************************************************

KIFC1  ACCTTACTGGCTGAGCAAGGAGACCGGCTCTATGGGTTAGAGATGGAGCGGCGACGACTC 1041
KIFC5B ACCTTACTGGCTGAGCAAGGAGACCGGCTCTATGGGTTAGAGATGGAGCGGCGGCGACTC 1074
```

Supplementary Figure S2 (Part 1). mRNA sequence alignment of Kifc1 and Kicf5b. Location of siRNA targeting sites, 5' and 3' untranslated regions are as marked.

```
       RNA9867
KIFC1  CACAACCAGCTGCAGGAACTGAAGGGCAATATCCGGGTGTTCTGCCGCGTGCGCCCTGTC 1101
KIFC5B CACAACCAGCTGCAGGAACTGAAGGGCAATATCCGGGTGTTCTGCCGCGTGCGCCCTGTC 1134
       ************************************************************
KIFC1  CTCGAAGGGGAATCCACTCCATCTCCTGGCTTCCTCGTGTTTCCTCCTGGCCCTGCTGGA 1161
KIFC5B CTCGAAGGGGAATCCACTCCATCTCCTGGCTTCCTCGTGTTTCCTCCTGGCCCTGCTGGA 1194
       ************************************************************
KIFC1  CCCTCTGATCCCCCGACGGGCCTTAGCCTCTCACGATCTGATGATCGGCGCTCCACCCTG 1221
KIFC5B CCCTCTGATCGCCCGACGGGCCTTAGCCTCTCACGATCTGATGATCGGCGCTCCACCCTG 1254
       ********** *************************************************
KIFC1  ACTGGGGCCCCGGCCCCCACTGTCCGCCATGATTTCTCCTTTGATCGGGTGTTCCCGCCG 1281
KIFC5B ACTGGGGCCCCGGCACCCACTGTCCGCCATGATTTCTCCTTTGATCGGGTGTTCCCGCCG 1314
       ************** *********************************************
KIFC1  GGAAGCAAGCAGGAGGAAGTGTTTGAGGAGATCGCCATGCTTGTCCAGTCAGCACTGGAT 1341
KIFC5B GGAAGCAAGCAGGAGGAAGTGTTTGAGGAGATCGCCATGCTTGTCCAGTCAGCACTAGAT 1374
       ******************************************************** ***
KIFC1  GGCTACCCTGTGTGCATTTTTGCCTATGGACAGACAGGCAGTGGCAAGACCTTCACTATG 1401
KIFC5B GGCTACCCTGTGTGCATTTTTGCCTATGGACAGACAGGCAGTGGCAAGACCTTCACTATG 1434
       ************************************************************
KIFC1  GAAGGAGGGCCTAGGGGAGACCCCCAATTGGAAGGGCTGATCCCTCGGGCCATGCGGCAT 1461
KIFC5B GAAGGAGGGCCTAGGGGAGACCCCCAATTGGCAGGGCTGATCCCTCGGGCCATGCGGCAT 1494
       ******************************* ****************************
KIFC1  CTGTTCTCTGTGGCCCAGGAGATGAGCGGCCAGGGCTGGACATACAGTTTTGTGGCGAGT 1521
KIFC5B CTGTTCTCTGTGGCCCAGGAGATGAGCGGCCAGGGCTGGACATACAGTTTTGTGGCGAGT 1554
       ************************************************************
KIFC1  TACGTAGAGATCTACAATGAGACCGTTCGAGACCTGCTAGCTACTGGGCCCCGCAAGGGA 1581 RNA9878
KIFC5B TACGTAGAGATCTACAATGAGACCGTTCGAGACCTGCTAGCTACTGGGCCCCGCAAGGGA 1614 RNA9879
       ************************************************************
KIFC1  CAAGGGGGCGAGTGTGAGATCCGTCGGGCAAGCCCAGGAAGTGAGGAGCTTACTGTCACC 1641
KIFC5B CAAGGGGGCGAGTGCGAGATCCGTCGGGCAAGCCCAGGGAGTGAGGAGCTTACTGTCACC 1674
       ************** *********************** *********************
KIFC1  AATGCCCGCTATGTCCCTGTTTCCTGTGAGAAAGAGGTGGAGGCCCTGCTCCATTTGGCT 1701
KIFC5B AATGCCCGCTATGTCCCTGTTTCCTGTGAGAAAGAGGTGGAGGCCCTGCTCCATTTGGCT 1734
       ************************************************************
KIFC1  CATCAGAACCGGGCTGTGGCCCACACTGCCCAAAATAAGAGATCATCACGCAGTCATAGT 1761
KIFC5B CACCAGAACCGGGCTGTGGCCCACACTGCCCAGAATAAGAGATCATCACGCAGTCATAGT 1794
       ** ***************************** ***************************
KIFC1  GTGTTCCAGCTGCAGATTTCTGGAGAGCATGCAGCTCGGGGCCTGCAGTGTGGCGCTCCG 1821
KIFC5B GTGTTCCAGCTGCAGATTTCTGGAGAGCATGCAGCCCGGGGCCTGCAGTGTGGCGCTCCC 1854
       *********************************** *********************** 
KIFC1  CTCAACCTTGTGGACCTAGCTGGGAGTGAGCGGCTAGACCCTGGCTTACACCTAGGCCCT 1881
KIFC5B CTCAACCTTGTGGACCTAGCTGGGAGTGAGCGGCTAGACCCTGGCTTACCCCTAGGCCCT 1914
       ************************************************* **********
KIFC1  GGGGAGCGTGATCGTCTTCGGGAGACACAGGCCATTAACAGCAGTCTGTCTACACTGGGA 1941
KIFC5B GGGGAGCGTGATCGTCTTCGGGAGACACAGGCCATTAACAGCAGTCTGTCTACATTGGGA 1974
       ****************************************************** *****
KIFC1  CTGGTCATAATGGCCCTGAGCAATAAGGAGTCCCACGTGCCTTACCGAAACAGCAAGCTC 2001
KIFC5B CTGGTCATAATGGCCCTGAGCAATAAGGAGTCCCACGTGCCTTACCGAAACAGCAAGCTC 2034
       ************************************************************
KIFC1  ACCTACTTGCTGCAGAACTCTCTGGGTGGCAGTGCCAAGATGCTTATGTTTGTGAATATT 2061
KIFC5B ACCTACTTGCTGCAGAACTCTCTGGGTGGCAGTGCCAAGATGCTTATGTTTGTGAATATT 2094
       ************************************************************
KIFC1  TCTCCTCTGGAAGAGAATGTCTCCGAGTCTCTGAATTCACTACGCTTTGCTTCCAAGGTG 2121
KIFC5B TCTCCTCTGGAAGAGAATGTCTCCGAGTCTCTGAATTCACTACGCTTTGCTTCCAAGGTG 2154
       ************************************************************
```

Supplementary Figure S2 (Part 2). mRNA sequence alignment of Kifc1 and Kicf5b. Location of siRNA targeting sites, 5’ and 3’ untranslated regions are as marked.

```
KIFC1   AACCAGTGTGTCATTGGTACTGCTCAGGCTAATAAGAAGTGAAGTCGGATCCAGAGCCTG 2181
KIFC5B  AACCAGTGTGTCATTGGTACTGCTCAGGCTAATAAGAAGTGAAGTCGGATCCAGAGC--- 2211
        *********************************************************

KIFC1   ATTCCCTTGCAAGCCAGTGCGCATGCGTCTCTGTTTTAGTGTGTA----TTCGGTGGGG 2237
KIFC5B  ----CCTTGCAAGCCAGTGCGCATGCGTCTCTGTTTTAGTGTGTATTTATTCGGTGGGG 2267
            ****************************************    ***********

KIFC1   TGGGTGGGAGTTGAGACATGATTTTATTGGGTGAACAATATTTATTATGTAATCAACTAT 2297
KIFC5B  TGGGTGGGAGTTGAGACATGATATTACTGGG-------TATTTATTATGTAATAAAGAAT 2320
        ********************** *** ****       *************** **  **

KIFC1   AAAT----AAAGAATAATCTGTTGGTTAAAA---------AAAAAA-------------- 2330
KIFC5B  AATCTTTTGTATGTTAATCCTTTTCTTAATCTTAGATTTTAAAAAATTGTGTGCAGTTGT 2380
        **         *    *****   **   ****             ******

KIFC1   ------------------------------------------------------------ 2330
KIFC5B  TTTGCCTGAGTGTATATGCATGCACCATGTGCATGTCTCCTGCTCCAGTGAGTTCAGAAC 2440

KIFC1   ------------------------------------------------------------ 2330
KIFC5B  AGGGCTTCAGGTCCTTCAAGAACTGGGATTATGGAATACATGTGGGTCCTCTGCAAAATC 2500

KIFC1   ------------------------------------------------------------ 2330
KIFC5B  AGTCAGTGCTCTTAACCGCCCAGCCATCTTCCCAGCCCGGCCCTTGTTACAGCTCTAAAC 2560

KIFC1   ------------------------------------------------------------ 2330
KIFC5B  GGTTTTATTAACTTAACGGTTTTAGCTGCCAAAGGAAGGTATTGTAAATAAATACTGTAA 2620

KIFC1   -------  2330
KIFC5B  GCACGTA  2627
```

3' UTR

Supplementary Figure S2 (Part 3). mRNA sequence alignment of Kifc1 and Kicf5b. Location of siRNA targeting sites, 5' and 3' untranslated regions are as marked.

```
KIFC1  MDVQAQRPPLLEVKRNVELKAALVKSSSRVPLSASRLKRGPDQMEDALEPAKKRTRVMGA 60
KIFC5B MDVQAQRKG-REGKRNVELKAALVKSSSRLPLSASSLKRGPDQMEDALEPAKKRTRVMGA 59
       *******    * ****************:***** ************************

KIFC1  VTKVDTSRPRGPLLSTVSQTQGHTAAQKGPKKTGPRGCSAIGTVLRSQKPVPAAPAQKPG 120
KIFC5B VTKVDTSRPRGPLLSTVSQTQGHNAAQKGPKKTGPGGCSAVGTVLRSQKPAPAAPA-KPG 118
       ***********************.*********** ****:*********.***** ***

KIFC1  TSTAPVVVGKRAGKRPAWDLKGQLCDLNEELKRYREKTQTLELENRGLREQLREVQEQAT 180
KIFC5B TSTAPVVVGKRAGKRPAWDLKGQLCDLNEELKRYREKTETLELENRGLREQLREVQEQAT 178
       **************************************:*********************

KIFC1  TLGTERNTLEGELASVRSRAEQDQQRLETLSARVLELEECLGTRERLLQELQGERLQLQE 240
KIFC5B TLGTERNTLEGELASVRSRAEQDQQRLETLSARVLELEECLGTRERLLQELQGERLQLQE 238
       ************************************************************

KIFC1  ERSTLSTQLEEQERRFQATEAALSSSQEEVVCLRQKTEAQVTLLAEQGDRLYGLEMERRR 300
KIFC5B ERSTLSTQLEEQERRFQATEAALSSSQEEVVCLRQKTEAQVTLLAEQGDRLYGLEMERRR 298
       ************************************************************

KIFC1  LHNQLQELKGNIRVFCRVRPVLEGESTPSPGFLVFPPGPAGPSDPPTGLSLSRSDDRRST 360
KIFC5B LHNQLQELKGNIRVFCRVRPVLEGESTPSPGFLVFPPGPAGPSDRPTGLSLSRSDDRRST 358
       ******************************************** ***************

KIFC1  LTGAPAPTVRHDFSFDRVFPPGSKQEEVFEEIAMLVQSALDGYPVCIFAYGQTGSGKTFT 420
KIFC5B LTGAPAPTVRHDFSFDRVFPPGSKQEEVFEEIAMLVQSALDGYPVCIFAYGQTGSGKTFT 418
       ************************************************************

KIFC1  MEGGPRGDPQLEGLIPRAMRHLFSVAQEMSGQGWTYSFVASYVEIYNETVRDLLATGPRK 480
KIFC5B MEGGPRGDPQLAGLIPRAMRHLFSVAQEMSGQGWTYSFVASYVEIYNETVRDLLATGPRK 478
       *********** ************************************************

KIFC1  GQGGECEIRRASPGSEELTVTNARYVPVSCEKEVEALLHLAHQNRAVAHTAQNKRSSRSH 540
KIFC5B GQGGECEIRRASPGSEELTVTNARYVPVSCEKEVEALLHLAHQNRAVAHTAQNKRSSRSH 538
       ************************************************************

KIFC1  SVFQLQISGEHAARGLQCGAPLNLVDLAGSERLDPGLHLGPGERDRLRETQAINSSLSTL 600
KIFC5B SVFQLQISGEHAARGLQCGAPLNLVDLAGSERLDPGLPLGPGERDRLRETQAINSSLSTL 598
       ************************************* **********************

KIFC1  GLVIMALSNKESHVPYRNSKLTYLLQNSLGGSAKMLMFVNISPLEENVSESLNSLRFASK 660
KIFC5B GLVIMALSNKESHVPYRNSKLTYLLQNSLGGSAKMLMFVNISPLEENVSESLNSLRFASK 658
       ************************************************************

KIFC1  VNQCVIGTAQANKK 674
KIFC5B VNQCVIGTAQANKK 672
       **************
```

Supplementary Figure S3. Protein sequence alignment of Kifc1 and Kicf5b.

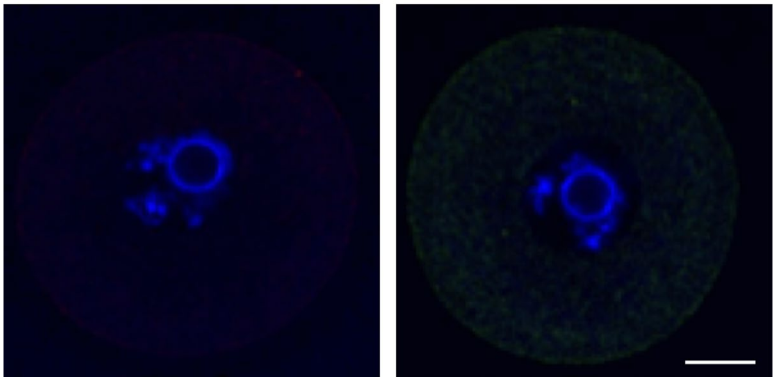

Supplementary Figure S4. Antibody specificity. Primary antibodies were substituted with the appropriate concentration of either anti-rabbit (red) or anti-mouse (green) IgG in negative control groups for immunocytochemistry. Oocytes were counterstained with the nuclear stain Hoechst 33342 (blue) and viewed using confocal microscopy. Scale bar = 20 μm.

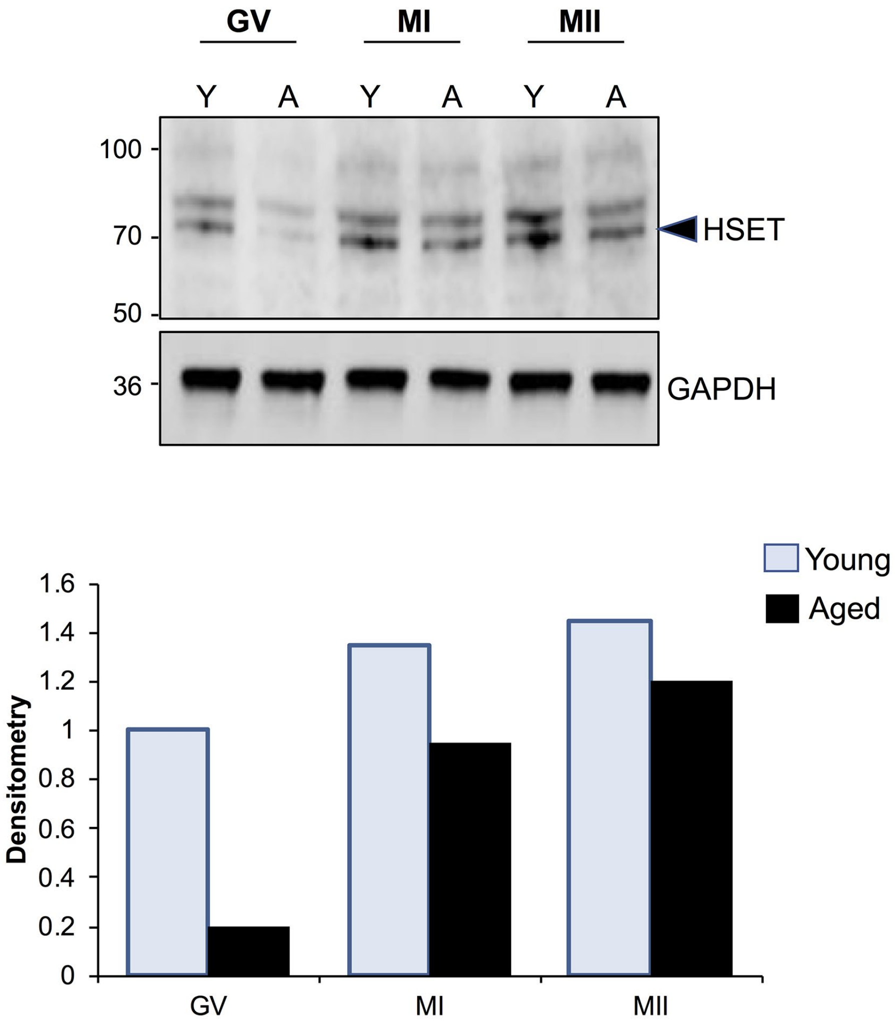

Supplementary Figure S5. Anti-HSET immunoblot in young and aged oocytes. Immunoblotting with anti-HSET antibodies revealed a predominant band at the appropriate molecular weight of approximately 74 kDa in cell lysates prepared from isolated GV, MI and MII stage oocytes of young (Y) and aged (A) animals. Densitometric analysis of the labeling intensity of the HSET band relative to that of the GAPDH loading control, revealed an age-dependent decrease in the relative abundance of HSET in lysates of aged GV, MI and, to a lesser extent, MII stage oocytes. This experiment was repeated once with each lane being loaded with total protein lysates prepared from the equivalent of 100 oocytes (i.e. ~ 2 µg protein / lane).
